# Supplementary material for: Isotopologues of potassium 2,2,2-trifluoroethoxide for applications in positron emission tomography and beyond
Source: Nat Commun. 2024 Jul 10;15:5798. doi: 10.1038/s41467-024-49975-7 (PMC11237122; doi:10.1038/s41467-024-49975-7)
Supplement: Supplementary file 1 — Supplementary Information [file 41467_2024_49975_MOESM1_ESM.pdf]

Supplementary Information for

Isotopologues of Potassium 2,2,2-Trifluoroethoxide for Applications in Positron Emission Tomography and Beyond

Qunchao Zhao, Sanjay Telu*, Susovan Jana, Cheryl L. Morse, Victor W. Pike*

Molecular Imaging Branch, National Institute of Mental Health, National Institutes of Health, Building 10, Rm B3C346, 10 Center Drive, Bethesda, MD 20892, USA.

*Corresponding authors: sanjay.telu@nih.gov; pikev@mail.nih.gov

Contents

Supplementary Notes S2

Supplementary Methods S3

1. Optimization of 2,2,2-trifluoroethanol synthesis S3
2. The synthesis of precursors and standards. S4
3. General HPLC methods S48
4. Syntheses of [^{11}C]fluoroform and [^{18}F]fluoroform S49
5. General procedure for producing [^{11}C/^{18}F]2,2,2-trifluoroethanol S52
6. General procedure for radio-2,2,2-trifluoroethoxylation. S55
7. HPLC analysis of ^{11}C- and ^{18}F- labeled products. S61

Supplementary Abbreviations S243

Supplementary NMR spectra S244

Supplementary References S377

Supplementary Notes

Fluoroform was purchased from Sigma-Aldrich. Other commercially available chemicals, reagents, and solvents were purchased primarily from Sigma-Aldrich, Alfa Aesar, TCI, AmBeed, and Chemscene, and used as received. ^{1}H (400.13 MHz), ^{13}C (100.62 MHz), and ^{19}F (376.47 MHz) NMR spectra were recorded on an Avance 400 instrument (Bruker) at RT in deuterated solvents. All spectra are reported with chemical shifts in parts per million. NMR data are reported as follows: chemical shift (δ (ppm), multiplicity (*s* = singlet, *d* = doublet, *t* = triplet, *q* = quartet, *quint* = quintet, *sept* = septet, *dd* = doublet of doublets, *dt* = doublet of triplets, *dq* = doublet of quartets, *qd* = quartet of doublets, *td* = triplet of doublets, *tt* = triplet of triplets, *ddd* = doublet of doublet of doublets, *dtd* = doublet of triplet of doublets, *tdd* = triplet of doublet of doublets, *m* = multiplet, app = apparent), coupling constants (*J* in Hz), and integration. TMS (δ = 0 ppm) was used as an internal standard for ^{1}H and ^{13}C-NMR spectroscopy and 1,1,1-trifluorobenzene (δ = –62 ppm) as internal standard for ^{19}F-NMR spectroscopy. NMR data were processed using MestReNova. HRMS data (ESI-TOF) were obtained at the Bioorganic Chemistry Laboratory of NIDDK (NIH). Melting points were measured with a digital SMP20 (Stuart) melting point apparatus. Radiochemistry was performed in a lead-shielded hot-cell for protection of personnel from radiation. HPLC analyses were performed on Shimadzu HPLC systems, which were also coupled with UV absorbance and radioactivity detectors. The HPLC chromatograms were processed using GraphPad Prism 8. Flash chromatography was performed on silica gel; eluent compositions are reported as *v*/*v*.

Supplementary Methods

1. Optimization of 2,2,2-trifluoroethanol synthesis.

1.1. Optimization.

Paraformaldehyde (*y* mmol) and *t*-BuOK (*z* mmol), followed by DMF (3.0 mL) were added. to a round-bottomed flask equipped with a magnetic stirrer bar under argon. Then fluoroform (*x* mmol in DMF; 0.3 M) was added. The flask was capped loosely, and the contents stirred at RT for *t* h. The reaction was then quenched with water (0.5 mL). The yield of 2,2,2-trifluoroethoxide (δ –76.3 ppm) from fluoroform (δ –78.9 ppm) was determined by ^{19}F NMR spectroscopy on the crude reaction mixture.

Supplementary Table 1. Optimization of the yield of 2,2,2-trifluoroethanol from the reaction of fluoroform with paraformaldehyde.

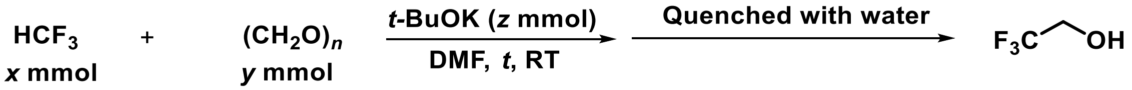

Entry	*x*	*y*	*z*	*t* (h)	Yield (%)
1	0.9	0.3	0.3	2	41
2	0.9	0.3	0.6	2	4
3	0.9	0.3	0.9	2	trace
4	0.6	0.3	0.3	2	21
5	0.3	0.3	0.3	2	33
6	0.15	0.3	0.3	2	68
7	0.1	0.3	0.3	2	95
8	0.1	0.4	0.3	2	75
9	0.1	0.15	0.3	2	12
10	0.1	0.1	0.3	2	12
11	0.1	0.3	0.3	3	96
12	0.1	0.3	0.3	1	91
13	0.1	0.3	0.3	0.5	88
14[a]	0.1	0.3	0.3	3	2
15[b]	0.1	0.3	0.3	3	trace
16[c]	0.1	0.3	0.3	3	trace
17[d]	0.1	0.3	0.3	3	trace
18[e]	0.1	0.3	0.3	3	trace

[a]*t*-BuONa as the base; [b]NaH as the base; [c]DMA as the solvent; [d]THF as the solvent; [e]DMSO as the solvent

1.2. One-pot 2,2,2-trifluoroethoxylation of 2-chloropyrimidine (1).

To a round-bottomed flask equipped with a magnetic stirrer bar was added paraformaldehyde (0.5 mmol) and *t*-BuOK (0.5 mmol), followed by DMF (3 mL) under

argon. A DMF solution (0.6 mL) of fluoroform (0.17 mmol; 0.3 M) was added and the solution was stirred at RT for 3 h. 2-Chloropyrimidine (0.5 mmol) in DMF (0.5 M) was then added and the reaction mixture was stirred either at RT or at 60 °C for another 2 h. The reaction was then quenched with water (0.5 mL). The yield of 2-(2,2,2-trifluoroethoxy)pyrimidine (**1**) (δ –73.8 ppm) was determined by ^{19}F NMR spectroscopy on the crude reaction mixture.

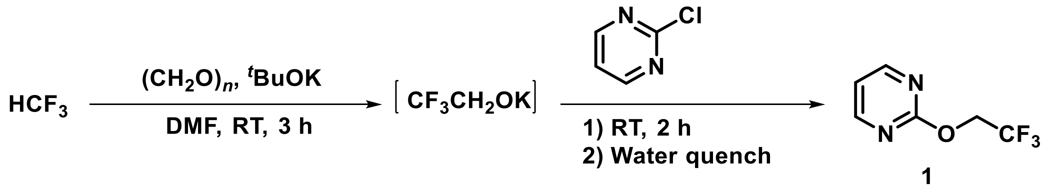

1

71% yield by ^{19}F NMR

Supplementary Figure 1. One-pot 2,2,2-trifluoroethoxylation of 2-chloropyrimidine to give **1**.

1.3. One-pot 2,2,2-trifluoroethoxylation of naphthalen-1-yl iodonium-(2,2-dimethyl-1,3-dioxane-4,6-dione)ylide (2).

The procedure used for the 2,2,2-trifluoroethoxylation of 2-chloropyrimidine was used instead with naphthalen-1-yl iodonium-(2,2-dimethyl-1,3-dioxane-4,6-dione)ylide (0.5 mmol). The yield of 1-(2,2,2-trifluoroethoxy)naphthalene (**2**) (δ –73.5 ppm) was determined by ^{19}F-NMR spectroscopy.

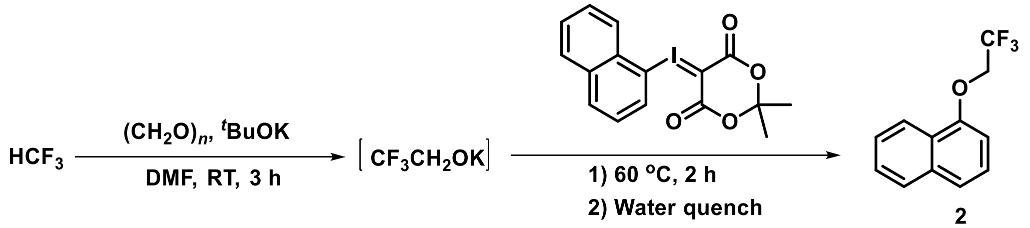

2

63% yield by ^{19}F NMR

Supplementary Figure 2. One-pot 2,2,2-trifluoroethoxylation of naphthalen-1-yl iodonium-(2,2-dimethyl-1,3-dioxane-4,6-dione)ylide to give **2.**

2. The synthesis of precursors and standards.

2.1. General procedure 1 for the synthesis of heterocycle precursors and standards.

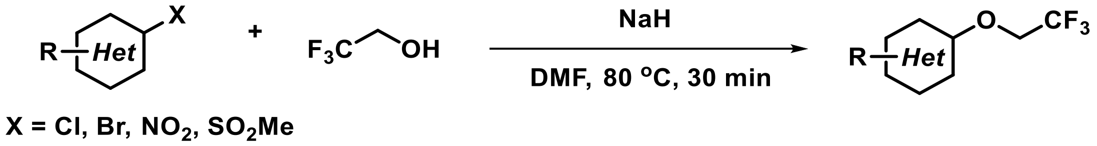

Supplementary Figure 3. General procedure 1 for the synthesis of 2,2,2-trifluoroethyl heterocyclic ethers.

To a round-bottomed flask equipped with a magnetic stirrer bar was added dry NaH (1 equiv.) and DMF (0.5 M) under argon. 2,2,2-Trifluoroethanol (2 equiv.) was then added dropwise, and the reaction mixture was stirred at RT for 5 min under argon. Precursor (1 equiv.) was then added and stirred vigorously at 80 ^{o}C for 30 min. After completion of the reaction, as assessed with TLC, the reaction mixture was cooled to RT, poured onto water, and extracted with DCM (2 × 25 mL). The organic phase was dried (Mg_2SO_4) and concentrated *in vacuo*. The residue was purified by silica gel flash chromatography using hexane-ethyl acetate to afford the corresponding 2,2,2-trifluoroethyl heterocyclic ether.

2-(2,2,2-Trifluoroethoxy)pyrimidine (1).

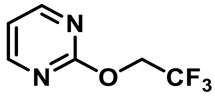

General procedure 1 with 2-chloropyrimidine on a 2.0 mmol scale followed by flash chromatography (ethyl acetate/*n*-hexane,1: 4) gave **1** as a colorless oil (274 mg, 77% yield). ^{1}H NMR (400 MHz, $CDCl_3$) *δ* (ppm): 8.54 (*d*, *J* = 4.8 Hz, 2H), 7.04 (*t*, *J* = 4.8 Hz, 1H), 4.79 (*q*, *J* = 8.4 Hz, 2H); ^{19}F NMR (376 MHz, $CDCl_3$) *δ* (ppm): –73.85 (*t*, *J* = 8.2 Hz, 3F); ^{13}C NMR (101 MHz, $CDCl_3$) *δ* (ppm): 163.85, 159.70, 123.39 (*q*, *J* = 277.6 Hz), 116.52, 63.57 (*q*, *J* = 36.5 Hz). MS (ESI): 179.1 $[M+H]^+$. HRMS (ESI) for $C_6H_6N_2OF_3$ $[M+H]^+$: calc'd: 179.0432; found: 179.0430.

6-(2,2,2-Trifluoroethoxy)nicotinaldehyde (3).

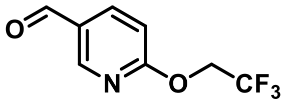

General procedure 1 with 6-chloronicotinaldehyde on a 2.0 mmol scale followed by flash chromatography (ethyl acetate/*n*-hexane, 1: 5) gave **3** as a pale-yellow solid (353 mg, 86% yield). Mp: 52–54 ^{o}C. ^{1}H NMR (400 MHz, $CDCl_3$) *δ* (ppm): 9.99 (*s*, 1H), 8.63 (*d*, *J* = 2.2 Hz, 1H), 8.14 (*dd*, *J* = 8.6, 2.2 Hz, 1H), 6.98 (*d*, *J* = 8.6 Hz, 1H), 4.85 (*q*, *J* = 8.4 Hz, 2H); ^{19}F NMR (376 MHz, $CDCl_3$) *δ* (ppm): –73.77 (*t*, *J* = 8.3 Hz, 3F); ^{13}C NMR (101 MHz, $CDCl_3$): *δ* (ppm) 189.36, 165.30, 152.10, 138.70, 128.12, 123.51 (*q*, *J* = 277.4 Hz), 112.32, 62.91 (*q*, *J* = 36.3 Hz). MS (ESI): 206.0 $[M+H]^+$. HRMS (ESI) for $C_8H_7NO_2F_3$ $[M+H]^+$: calc'd: 206.0429; found: 206.0426.

5-Bromo-2-(2,2,2-trifluoroethoxy)pyridine (4).

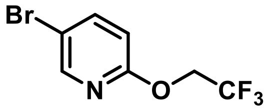

General procedure 1 with 5-bromo-2-nitropyridine on a 2.0 mmol scale followed by flash chromatography (ethyl acetate/*n*-hexane, 1: 10) gave **4** as a colorless oil (342 mg, 67% yield). ^{1}H NMR (400 MHz, $CDCl_3$) *δ* (ppm): 8.19 (*d*, *J* = 2.4 Hz, 1H), 7.72 (*dd*, *J* = 8.7, 2.5 Hz, 1H), 6.78 (*d*, *J* = 8.7

Hz, 1H), 4.72 (*q*, *J* = 8.5 Hz, 2H); ^{19}F NMR (376 MHz, $CDCl_3$) *δ* (ppm): –73.84 (*t*, *J* = 8.6 Hz, 3F); ^{13}C NMR (101 MHz, $CDCl_3$) *δ* (ppm): 160.78, 147.49, 142.06, 123.73 (*q*, *J* = 277.5 Hz), 113.56, 112.93, 62.57 (*q*, *J* = 36.1 Hz). (Known compound).[1]

4,6-Dimethyl-2-(2,2,2-trifluoroethoxy)nicotinonitrile (5).

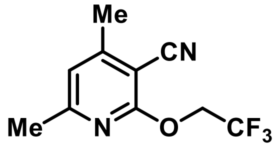

General procedure 1 with 2-chloro-3-cyano-4,6-dimethylpyridine on a 1.0 mmol scale followed by flash chromatography (ethyl acetate/*n*-hexane, 1: 10) gave **5** as a white solid (201 mg, 87% yield). Mp: 54–56 °C. ^{1}H NMR (400 MHz, $CDCl_3$) *δ* (ppm): 6.80 (*s*, 1H), 4.85 (*q*, *J* = 8.4 Hz, 2H), 2.47 (*s*, 3H), 2.45 (*s*, 3H); ^{19}F NMR (376 MHz, $CDCl_3$) *δ* (ppm) –73.71 (*t*, *J* = 8.2 Hz, 3F); ^{13}C NMR (101 MHz, $CDCl_3$) *δ* (ppm): 161.84, 160.71, 155.41, 123.39 (*q*, *J* = 277.6 Hz), 119.32, 114.09, 94.53, 62.64 (*q*, *J* = 36.6 Hz), 24.52, 20.26. MS (ESI): 231.1 [*M*+H]$^+$. HRMS (ESI) for $C_{10}H_{10}N_2OF_3$ [*M*+H]$^+$: calc'd: 231.0745; found: 231.0742.

3-Benzyl-6–bromo-2-(2,2,2-trifluoroethoxy)quinoline (6).

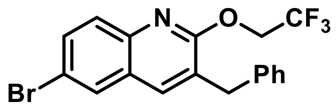

General procedure 1 with 3-benzyl-6-bromo-2-chloroquinoline on a 1.0 mmol scale followed by flash chromatography (ethyl acetate/*n*-hexane, 1: 20) gave **6** as a white solid (313 mg, 79% yield). Mp: 90–92 °C .^{1}H NMR (400 MHz, $CDCl_3$) *δ* (ppm): 7.77 (*d*, *J* = 1.7 Hz, 1H), 7.70–7.56 (*m*, 3H), 7.37–7.28 (*m*, 2H), 7.26–7.21 (*m*, 3H), 4.88 (*q*, *J* = 8.5 Hz, 2H), 4.04 (*s*, 2H); ^{19}F NMR (376 MHz, $CDCl_3$) *δ* (ppm): –73.46 (*t*, *J* = 8.7 Hz, 3F); ^{13}C NMR (101 MHz, $CDCl_3$) *δ* (ppm): 158.78, 143.58, 138.53, 136.91, 132.58, 129.45, 129.37, 128.83, 127.42, 126.91, 126.82, 123.95 (*q*, *J* = 277.3 Hz), 118.27, 62.71 (*q*, *J* = 36.1 Hz), 36.21. MS (ESI): 396.0 [*M*+H]$^+$. HRMS (ESI) for $C_{18}H_{14}NOF_3Br$ [*M*+H]$^+$: calc'd: 396.0211; found: 396.0208.

6-Methoxy-1-(2,2,2-trifluoroethoxy)isoquinoline (7).

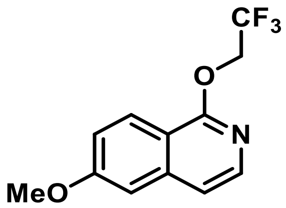

General procedure 1 with 1-chloro-6-methoxyisoquinoline on a 2.0 mmol scale followed by flash chromatography (ethyl acetate/*n*-hexane, 1: 4) gave **7** as a white solid (473 mg, 92% yield). Mp: 80–82 °C. ^{1}H NMR (400 MHz, $CDCl_3$) *δ* (ppm): 8.16 (*d*, *J* = 9.1 Hz, 1H), 7.90 (*d*, *J* = 5.9 Hz, 1H), 7.19 (*dd*, *J* = 12.8, 4.0 Hz, 2H), 7.03 (*d*, *J* = 2.2 Hz, 1H), 4.92 (*q*, *J* = 8.6 Hz, 2H), 3.93 (*s*, 3H); ^{19}F NMR (376 MHz, $CDCl_3$) *δ* (ppm): –73.67 (*t*, *J* = 8.3 Hz, 3F); ^{13}C NMR (101 MHz, $CDCl_3$) *δ* (ppm): 161.68, 158.62, 140.48, 139.96, 125.92, 124.09 (*q*, *J* = 277.3 Hz), 119.44, 116.22, 114.23, 104.86, 62.56 (*q*, *J* = 36.0 Hz), 55.63. MS

(ESI): 258.1 [*M*+H]$^+$. HRMS (ESI) for $C_{12}H_{11}NO_2F_3$ [*M*+H]$^+$: calc'd: 258.0742; found: 258.0740.

3-(2,2,2-Trifluoroethoxy)-1,2-benzisothiazole (8).

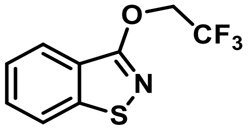

General procedure 1 with 3-chloro-1,2-benzisothiazole on a 1.0 mmol scale followed by flash chromatography (ethyl acetate/*n*-hexane, 1: 10) gave **8** as a colorless oil (178 mg, 76% yield). ^{1}H NMR (400 MHz, $CDCl_3$) δ (ppm): 7.97 (*d*, *J* = 8.1 Hz, 1H), 7.79 (*d*, *J* = 8.2 Hz, 1H), 7.56 (*t*, *J* = 7.6 Hz, 1H), 7.43 (*t*, *J* = 7.5 Hz, 1H), 4.95 (*q*, *J* = 8.3 Hz, 2H); ^{19}F NMR (376 MHz, $CDCl_3$) δ (ppm): –73.91 (*t*, *J* = 8.3 Hz, 3F); ^{13}C NMR (101 MHz, $CDCl_3$) δ (ppm): 160.95, 152.42, 129.29, 125.04, 124.54, 123.41 (*q*, *J* = 277.3 Hz), 123.24, 120.43, 65.00 (*q*, *J* = 36.5 Hz). MS (ESI): 234.0 [*M*+H]$^+$. HRMS (ESI) for $C_9H_7NOF_3S$ [*M*+H]$^+$: calc'd: 234.0200; found: 234.0197.

***tert*-Butyl 2-(2,2,2-trifluoroethoxy)-7,8-dihydropyrido[4,3-*d*]pyrimidine-6(5*H*)-carboxylate (9).**

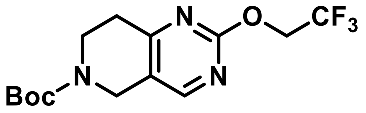

General procedure 1 with *tert*-butyl 2-(methylsulfonyl)-7,8-dihydropyrido[4,3-*d*]pyrimidine-6(5*H*)-carboxylate on a 1.0 mmol scale followed by flash chromatography (ethyl acetate/*n*-hexane, 1: 4) gave **9** as a white solid (301 mg, 90% yield). Mp: 65–67 ^{o}C. ^{1}H NMR (400 MHz, $CDCl_3$) δ (ppm): 8.24 (*s*, 1H), 4.75 (*q*, *J* = 8.4 Hz, 1H), 4.51 (*s*, 1H), 3.70 (*t*, *J* = 6.0 Hz, 1H), 2.86 (*t*, *J* = 5.9 Hz, 1H), 1.45 (*s*, 1H); ^{19}F NMR (376 MHz, $CDCl_3$) δ (ppm): –73.86 (*t*, *J* = 8.3 Hz); ^{13}C NMR (101 MHz, $CDCl_3$) δ (ppm): 166.73, 162.33, 156.93, 154.65, 123.37 (*q*, *J* = 277.8 Hz), 122.14, 80.76, 63.49 (*q*, *J* = 36.3 Hz), 42.22, 40.85, 31.81, 28.48. MS (ESI): 334.2 [*M*+H]$^+$. HRMS (ESI) for $C_{14}H_{19}N_3O_3F_3$ [*M*+H]$^+$: calc'd: 334.1379; found: 334.1376.

4-Phenyl-2-(2,2,2-trifluoroethoxy)quinazoline (10).

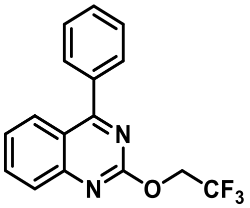

General procedure 1 with methyl 2-chloro-3-quinolinecarboxylate on a 1.0 mmol scale followed by flash chromatography (ethyl acetate/*n*-hexane, 1: 10) gave **10** as a white solid (273 mg, 90% yield). Mp: 86–88 ^{o}C. ^{1}H NMR (400 MHz, $CDCl_3$) δ (ppm): 8.07 (*d*, *J* = 8.4 Hz, 1H), 7.90 (*d*, *J* = 8.4 Hz, 1H), 7.84 (*t, J* = 7.6 Hz, 1H), 7.80–7.75 (*m*, 2H), 7.62–7.51 (*m*, 3H), 7.45 (*t*, *J* = 7.6 Hz, 1H), 5.00 (*q*, *J* = 8.5 Hz, 2H); ^{19}F NMR (376 MHz, $CDCl_3$) δ (ppm): –73.49 (*t*, *J* = 8.5 Hz, 3F); ^{13}C NMR (101 MHz, $CDCl_3$) δ (ppm): 172.76, 160.39, 152.87,

136.65, 134.67, 130.57, 130.19, 128.75, 127.79, 127.50, 125.84, 123.63 (*q*, *J* = 277.7 Hz), 120.72, 63.69 (*q*, *J* = 36.2 Hz). MS (ESI): 305.1 [*M*+H]$^+$. HRMS (ESI) for $C_{16}H_{12}N_2OF_3$ [*M*+H]$^+$: calc'd: 305.0902; found: 305.0903.

4-(2,2,2-Trifluoroethoxy)furo[2,3-*d*]pyrimidine (11).

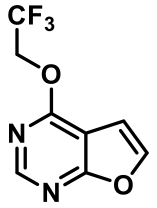

General procedure 1 with 4-chlorofuro[2,3-*d*]pyrimidine on a 1.0 mmol scale followed by flash chromatography (ethyl acetate/petroleum ether, 1: 4) gave **11** as a colorless oil (193 mg, 89% yield). ^{1}H NMR (400 MHz, $CDCl_3$) δ (ppm): 8.56 (*s*, 1H), 7.65 (*d*, *J* = 2.5 Hz, 1H), 6.88 (*d*, *J* = 2.5 Hz, 1H), 4.97 (*q*, *J* = 8.4 Hz, 2H); ^{19}F NMR (376 MHz, $CDCl_3$) δ (ppm): –73.69 (*t*, *J* = 8.2 Hz, 3F); ^{13}C NMR (101 MHz, $CDCl_3$) δ (ppm): 168.74, 162.29, 152.81, 143.81, 123.38 (*q*, *J* = 277.4 Hz), 104.80, 103.36, 62.86 (*q*, *J* = 36.7 Hz). MS (ESI): 219.0 [*M*+H]$^+$. HRMS (ESI) for $C_8H_6N_2O_2F_3$ [*M*+H]$^+$: calc'd: 219.0381; found: 219.0380.

3-Phenyl-6-(2,2,2-trifluoroethoxy)pyridazine (12)

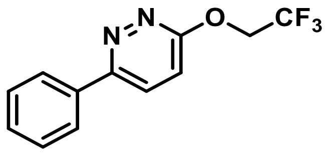

General procedure 1 with 3-chloro-6-phenylpyridazine on a 1.0 mmol scale followed by flash chromatography (ethyl acetate/*n*-hexane, 1: 10) gave **12** as a white solid (132 mg, 52% yield). Mp: 132–134 ^{o}C.^{1}H NMR (400 MHz, $CDCl_3$) δ (ppm): 7.99 (*dd*, *J* = 7.6, 1.5 Hz, 2H), 7.86 (*d*, *J* = 9.2 Hz, 1H), 7.64–7.39 (*m*, 3H), 7.18 (*d*, *J* = 9.2 Hz, 1H), 5.00 (*q*, *J* = 8.4 Hz, 2H); ^{19}F NMR (376 MHz, $CDCl_3$) δ (ppm): –73.80 (t, *J* = 8.7 Hz, 3F); ^{13}C NMR (101 MHz, $CDCl_3$) δ (ppm): 162.64, 156.86, 135.88, 130.02, 129.22, 128.09, 126.87, 123.52 (*q*, *J* = 277.2 Hz), 117.86, 63.56 (*q*, *J* = 36.5 Hz). MS (ESI): 255.1 [*M*+H]$^+$. HRMS (ESI) for $C_{12}H_{10}N_2OF_3$ [*M*+H]$^+$: calc'd: 255.0745; found: 255.0744.

2,4-Diphenyl-6-(2,2,2-trifluoroethoxy)-1,3,5-triazine (13).

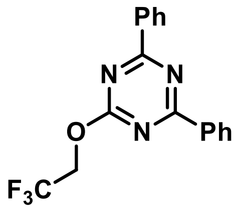

General procedure 1 with 2-bromo-4,6-diphenyl-1,3,5-triazine on a 0.5 mmol scale followed by flash chromatography (ethyl acetate/*n*-hexane, 1: 20) gave **13** as a white solid (123 mg, 74% yield). Mp: 118–120 ^{o}C. ^{1}H NMR (400 MHz, $CDCl_3$) δ (ppm): 8.69–8.56 (*m*, 4H), 7.66–7.58 (*m*, 2H), 7.58–7.47 (*m*, 4H), 5.02 (*q*, *J* = 8.3 Hz, 2H); ^{19}F NMR (376 MHz, $CDCl_3$) δ (ppm): –73.33 (*t*, *J* = 8.2 Hz, 3F); ^{13}C NMR (101 MHz, $CDCl_3$) δ (ppm): 174.24, 170.66, 135.23, 133.36, 129.39, 128.89, 123.24 (*q*, *J* = 277.8 Hz), 63.60 (*q*, *J* = 36.9 Hz). MS (ESI): 332.1 [*M*+H]$^+$. HRMS (ESI) for $C_{17}H_{13}N_3OF_3$ [*M*+H]$^+$: calc'd: 332.1011; found: 332.1005.

2,7-Bis(2,2,2-trifluoroethoxy)-1,8-naphthyridine (14).

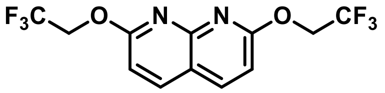

General procedure 1 with 2-chloro-7-(2,2,2-trifluoroethoxy)-1,8-naphthyridine on a 0.5 mmol scale followed by flash chromatography (ethyl acetate/petroleum ether, 1: 5) gave **14** as a white solid (110 mg, 67% yield). Mp: 165–167 ^{o}C. ^{1}H NMR (400 MHz, $CDCl_3$) *δ* (ppm): 8.04 (*d*, *J* = 8.6 Hz, 2H), 7.01 (*d*, *J* = 8.6 Hz, 2H), 4.97 (*q*, *J* = 8.5 Hz, 4H); ^{19}F NMR (376 MHz, $CDCl_3$) *δ* (ppm): –73.70 (*t*, *J* = 8.7 Hz, 3F); ^{13}C NMR (101 MHz, $CDCl_3$) *δ* (ppm): 163.30, 153.60, 139.96, 123.77 (*q*, *J* = 277.1 Hz), 116.71, 111.46, 63.05 (*q*, *J* = 36.1 Hz). MS (ESI): 327.1 [*M*+H]$^{+}$. HRMS (ESI) for $C_{12}H_9N_2O_2F_6$ [*M*+H]$^{+}$: calc'd: 327.0568; found: 327.0564.

4-Chloro-1-isobutyl-1*H*–imidazo[4,5-*c*]quinoline.

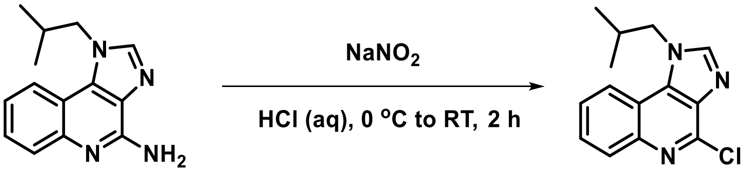

A dried round bottomed flask equipped with a stirrer bar was loaded with Imiquimod (1-isobutyl-1*H*-imidazo[4,5-*c*]quinolin-4-amine; 721 mg, 3.0 mmol, 1 equiv.), followed by concentrated hydrochloric acid (11 mL). The flask was cooled to 0 ^{o}C, and then a cold (0 ^{o}C) aqueous solution (10 mL) of $NaNO_2$ (3.45 g, 50 mmol, 16.7 equiv.) was added in three portions. The mixture was stirred at bath temperature for 30 min and then at RT for 2 h. Water (50 mL) and DCM (50 mL) were then added to the reaction mixture. The organic layer was separated off, washed with brine, dried (Mg_2SO_4), and concentrated in *vacuo*. The residue was purified by silica gel chromatography (ethyl acetate/DCM, 1: 1) to give the title compound as a white solid (343 mg, 44% yield). Mp: 140–142 ^{o}C. ^{1}H NMR (400 MHz, $CDCl_3$) *δ* (ppm): 8.09 (*dd*, *J* = 8.3, 1.2 Hz, 1H), 7.98 (*dd*, *J* = 8.1, 1.3 Hz, 1H), 7.86 (*s*, 1H), 7.59 (*dtd*, *J* = 15.0, 7.1, 1.4 Hz, 2H), 4.28 (*d*, *J* = 7.4 Hz, 2H), 2.27 (*sept*, *J* = 13.8, 6.9 Hz, 1H), 0.99 (*d*, *J* = 6.7 Hz, 6H); ^{13}C NMR (101 MHz, $CDCl_3$) *δ* (ppm): 144.28, 144.10, 135.26, 134.04, 130.12, 128.05, 126.99, 120.18, 117.75, 55.37, 28.91, 19.88. MS (ESI): 260.1 [*M*+H]$^{+}$. HRMS (ESI) for $C_{14}H_{15}N_3Cl$ [*M*+H]$^{+}$: calc'd: 260.0955; found: 260.0950.

1-Isobutyl-4-(2,2,2-trifluoroethoxy)-1*H*-imidazo[4,5-c]quinoline (15).

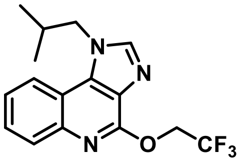

General procedure 1 with 4-chloro-1-isobutyl-1*H*–imidazo[4,5-*c*]quinoline (prepared as described immediately above) on a 1.0 mmol scale followed by flash chromatography (ethyl acetate/*n*-hexane, 2: 1)

gave **15** as a white solid (179 mg, 55% yield). Mp: 136–138 °C. ^{1}H NMR (400 MHz, $CDCl_3$) δ (ppm): 7.98 (*d*, *J* = 8.4 Hz, 1H), 7.82 (*s*, 1H), 7.59 (*ddd*, *J* = 8.4, 7.2, 1.2 Hz, 1H), 7.53–7.41 (*m*, 1H), 5.13 (*q*, *J* = 8.6 Hz, 2H), 4.29 (*d*, *J* = 7.4 Hz, 2H), 2.32 (*sept*, *J* = 13.8, 6.9 Hz, 1H), 1.01 (*d*, *J* = 6.7 Hz, 6H); ^{19}F NMR (376 MHz, $CDCl_3$) δ (ppm): –73.12 (t, *J* = 8.8 Hz, 3F); ^{13}C NMR (101 MHz, $CDCl_3$) δ (ppm): 153.44, 143.73, 142.82, 135.22, 128.98, 128.50, 127.72, 124.89, 123.94 (*q*, *J* = 277.6 Hz), 120.18, 117.15, 62.04 (*q*, *J* = 36.2 Hz), 55.21, 28.98, 19.90. MS (ESI): 324.1 [*M*+H]$^+$. HRMS (ESI) for $C_{16}H_{17}N_3OF_3$ [*M*+H]$^+$: calc'd: 324.1324; found: 324.1324.

9-(Tetrahydro-2*H*-pyran-2-yl)-6-(2,2,2-trifluoroethoxy)-9*H*-purine (16).

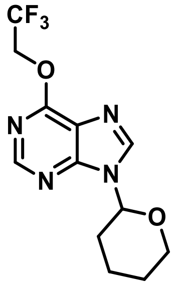

General procedure 1 with 6-chloro-9-(tetrahydro-2*H*-pyran-2-yl)-9*H*-purine on a 1.0 mmol scale followed by flash chromatography (ethyl acetate/*n*-hexane, 1: 2) gave **16** as a white solid (287 mg, 95% yield). Mp: 113–115 °C. ^{1}H NMR (400 MHz, $CDCl_3$) δ (ppm): 8.53 (*s*, 1H), 8.19 (*s*, 1H), 5.76 (*dd*, *J* = 10.1, 2.5 Hz, 1H), 5.27–4.85 (*m*, 2H), 4.16 (*dd*, *J* = 12.7, 2.9 Hz, 1H), 3.77 (*td*, *J* = 11.6, 2.7 Hz, 1H), 2.18–1.95 (*m*, 3H), 1.87–1.59 (*m*, 3H); ^{19}F NMR (376 MHz, $CDCl_3$) δ (ppm): –73.40 (*t*, *J* = 8.6 Hz, 3F); ^{13}C NMR (101 MHz, $CDCl_3$) δ (ppm): 158.90, 152.32, 151.75, 141.12, 123.35 (*q*, *J* = 277.9 Hz), 121.36, 82.45, 69.03, 62.51 (*q*, *J* = 36.9 Hz), 32.01, 25.01, 22.92. MS (ESI): 303.1 [*M*+H]$^+$. HRMS (ESI) for $C_{12}H_{14}N_4O_2F_3$ [*M*+H]$^+$: calc'd: 303.1069; found: 303.1068.

***tert*-Butyl 3,6-dichloropicolinate.**

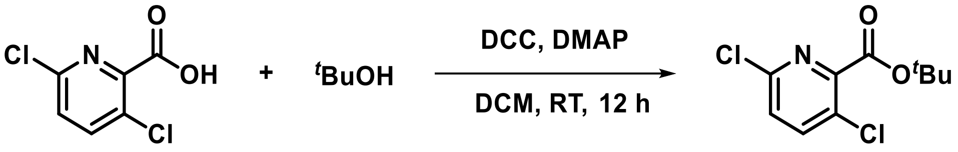

To a dried round-bottomed flask equipped with a stirrer bar were added Clopyralid (3,6-dichloropicolinic acid; 960 mg, 5.0 mmol, 1 equiv.), *tert*-butanol (740 mg, 10.0 mmol, 2 equiv.), DCC (1.55 g, 7.5 mmol, 1.5 equiv.), and DMAP (916 mg, 7.5 mmol, 1.5 equiv.). DCM (30 mL) was then added. The reaction mixture was stirred for 12 h at RT and then water (50 mL) and DCM (50 mL) were added. The organic phase was washed with brine, dried (Mg_2SO_4), and concentrated *in vacuo*. The residue was purified with silica gel chromatography (ethyl acetate/*n*-hexane, 1: 20) to give the title compound (1.17 g, 92% yield) as a white solid. Mp: 61–63 °C. ^{1}H NMR (400 MHz, $CDCl_3$) δ (ppm): 7.69 (*d*, *J* =

8.5 Hz, 1H), 7.33 (*d*, *J* = 8.5 Hz, 1H), 1.61 (*s*, 9H); ^{13}C NMR (101 MHz, $CDCl_3$) *δ* (ppm): 162.89, 149.88, 149.19, 140.78, 128.41, 126.68, 84.47, 28.22. MS (ESI): 270.0 [*M*+Na]$^+$. HRMS (ESI) for $C_{10}H_{11}NO_2Cl_2Na$ [*M*+Na]$^+$: calc'd: 270.0065; found: 270.0062.

***tert*-Butyl 3-chloro-6-(2,2,2-trifluoroethoxy)picolinate (17).**

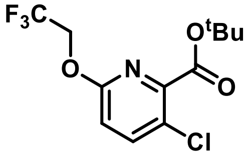

General procedure 1 with *tert*-butyl 3,6-dichloropicolinate (prepared as described immediately above) on a 1.0 mmol scale followed by flash chromatography (ethyl acetate/*n*-hexane, 1: 20) afforded **17** as a colorless oil (128 mg, 41% yield). ^{1}H NMR (400 MHz, $CDCl_3$) *δ* (ppm): 7.66 (*d*, *J* = 8.8 Hz, 1H), 6.89 (*d*, *J* = 8.8 Hz, 1H), 4.75 (*q*, *J* = 8.5 Hz, 2H), 1.62 (*s*, 9H); ^{19}F NMR (376 MHz, $CDCl_3$) *δ* (ppm): –73.80 (*t*, *J* = 8.2 Hz, 3F); ^{13}C NMR (101 MHz, $CDCl_3$) *δ* (ppm): 163.47, 159.39, 145.91, 141.53, 123.42 (*q*, *J* = 277.2 Hz), 123.34, 113.98, 83.67, 62.76 (*q*, *J* = 36.2 Hz), 28.07.

6-Bromo-2-methyl-3,4'-bipyridine-5-carbonitrile.

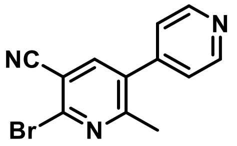

Phosphorus oxybromide (4.30 g, 15 mmol, 3 equiv.) was dissolved in anhydrous MeCN (15 mL). Milrinone (2-methyl-6-oxo-1,6-dihydro-[3,4'-bipyridine]-5-carbonitrile; 1.06 g, 5.0 mmol, 1 equiv.) and DMF (365 mg, 5.0 mmol, 1 equiv.) were added under argon and the resultant mixture was stirred at 85 ^{o}C for 12 h. The reaction mixture was cooled to RT, poured onto ice-water, and basified with saturated sodium carbonate solution until the *p*H was between 8 and 9. The mixture was extracted with DCM (2 × 15 mL), washed with brine, dried (Mg_2SO_4), and concentrated under reduced pressure. The residue was purified by silica gel chromatography (ethyl acetate/*n*-hexane: 8: 1) to give the title compound as a white solid (692 g, 51% yield). Mp: 141–143 ^{o}C. ^{1}H NMR (400 MHz, $CDCl_3$) *δ* (ppm): 8.73 (*d*, *J* = 4.5 Hz, 2H), 7.75 (*s*, 1H), 7.24 (*d*, *J* = 4.5 Hz, 2H), 2.55 (*s*, 3H); ^{13}C NMR (101 MHz, $CDCl_3$) *δ* (ppm): 161.38, 150.63, 144.35, 142.46, 142.40, 133.91, 123.54, 115.67, 111.79, 23.77. MS (ESI): 274.0 [*M*+H]$^+$. HRMS (ESI) for $C_{12}H_9N_3Br$ [*M*+H]$^+$: calc'd: 273.9980; found: 273.9976.

2-Methyl-6-(2,2,2-trifluoroethoxy)-3,4'-bipyridine-5-carbonitrile (18).

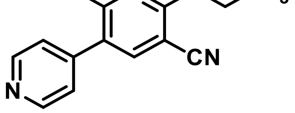

General procedure 1 with 6-bromo-2-methyl-3,4'-bipyridine-5-carbonitrile (prepared as described immediately above) on 0.5 mmol scale followed by flash chromatography (ethyl acetate/*n*-

hexane, 8: 1) gave **18** as a white solid (112 mg, 76% yield). Mp: 135–137 ^oC. ^1H NMR (400 MHz, $CDCl_3$) δ (ppm): 8.70 (*d*, *J* = 5.8 Hz, 2H), 7.79 (*s*, 1H), 7.22 (*d*, *J* = 5.8 Hz, 2H), 4.92 (*q*, *J* = 8.3 Hz, 2H), 2.48 (*s*, 3H); ^{19}F NMR (376 MHz, $CDCl_3$) δ (ppm): –73.56 (*t, J* = 8.2 Hz, 3F); ^{13}C NMR (101 MHz, $CDCl_3$) δ (ppm): 160.50, 159.27, 150.47, 145.21, 144.11, 129.48, 123.98, 123.19 (q, *J* = 277.8 Hz), 114.06, 94.77, 62.90 (*q*, *J* = 36.8 Hz), 23.63. MS (ESI): 294.1 $[M+H]^+$. HRMS (ESI) for $C_{14}H_{11}N_3OF_3$ $[M+H]^+$: calc'd: 294.0854; found: 294.0857.

6,7-Bis(2-methoxyethoxy)-4-(2,2,2-trifluoroethoxy)quinazoline (19).

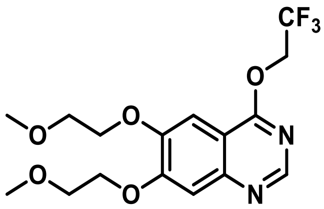

General procedure 1 with 4-chloro-6,7-bis(2-methoxyethoxy)quinazoline on a 0.5 mmol scale followed by flash chromatography (ethyl acetate/petroleum ether, 9: 1) gave **19** as a white solid (123 mg, 65% yield). Mp: 93–95 ^oC. ^1H NMR (400 MHz, $CDCl_3$) δ (ppm): 8.60 (*s*, 1H), 7.33 (*s*, 1H), 7.23 (*s*, 1H), 4.94 (*q*, *J* = 8.4 Hz, 2H), 4.32–4.16 (*m*, 4H), 3.84 (*m*, 4H), 3.46–3.45 (*m*, 6H); ^{19}F NMR (376 MHz, $CDCl_3$) δ (ppm): –73.54 (*t*, *J* = 8.2 Hz, 3F); ^{13}C NMR (101 MHz, $CDCl_3$) δ (ppm): 163.55, 155.64, 152.35, 149.83, 149.09, 123.54 (*q*, *J* = 277.3 Hz), 110.26, 107.89, 102.48, 70.80, 70.57, 68.90, 68.64, 62.57 (*q*, *J* = 36.5 Hz), 59.47, 59.44. MS (ESI): 377.2 $[M+H]^+$. HRMS (ESI) for $C_{16}H_{20}N_2O_5F_3$ $[M+H]^+$: calc'd: 377.1324; found: 377.1328.

2-Fluoro-5-(3-((5-(4-fluorophenyl)thiophen-2-yl)methyl)-4-methylphenyl)pyridine.

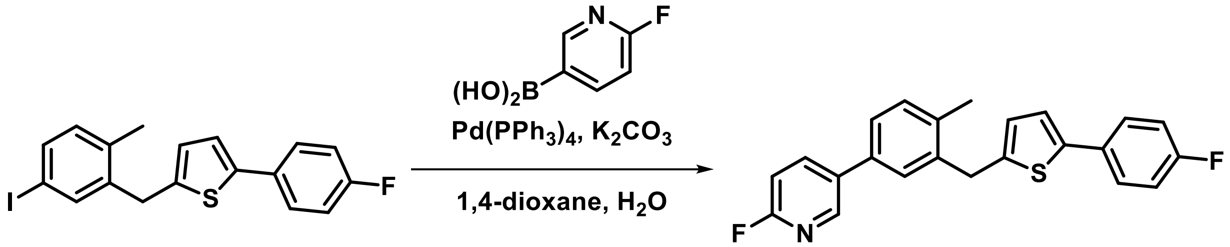

A mixture of 2-fluoropyridine-5-boronic (845 mg, 6.0 mmol, 1.2 equiv.), 2-(4-fluorophenyl)-5-[(5-iodo-2-methylphenyl)methyl]thiophene (2.04 g, 5.0 mmol, 1 equiv.), $Pd(PPh_3)_4$ (289 mg, 0.25 mmol, 5 mol%), K_2CO_3 (2.07 g, 15 mmol, 3 equiv.) in 1,4–dioxane (25 mL) and water (5 mL) was degassed for 15 min under argon. The reaction mixture was then heated at 100 ^oC overnight. The reaction mixture was cooled to RT and diluted with DCM (300 mL). The organic layer was washed with water (200 mL), dried (Mg_2SO_4), filtered, and concentrated. The residue was purified by silica gel chromatography (ethyl acetate/*n*-hexane, 1: 10) to give the title compound as a white solid (1.12 g, 59% yield). Mp: 118–

120 ^oC.^1H NMR (400 MHz, $CDCl_3$) δ (ppm): 8.41 (*d*, *J* = 2.4 Hz, 1H), 7.95 (*td*, *J* = 8.1, 2.6 Hz, 1H), 7.56–7.44 (*m*, 2H), 7.43–7.33 (*m*, 2H), 7.29 (*d*, *J* = 7.8 Hz, 1H), 7.11–6.93 (*m*, 4H), 6.72 (*d*, *J* = 3.6 Hz, 1H), 4.20 (*s*, 2H), 2.39 (*s*, 3H); ^{19}F NMR (376 MHz, $CDCl_3$) δ (ppm): –70.83 (*d*, *J* = 6.0 Hz, 1F), –111.96 to –121.23 (m, 1F); ^{13}C NMR (101 MHz, $CDCl_3$) δ (ppm): 163.96 (*d*, *J* = 83.7 Hz), 161.54 (*d*, *J* = 91.6 Hz), 145.85 (*d*, *J* = 14.7 Hz), 142.97, 141.96, 139.73 (*d*, *J* = 7.7 Hz), 139.32, 136.77, 134.85, 134.78 (*d*, *J* = 4.8 Hz), 131.50, 130.92 (*d*, *J* = 3.2 Hz), 128.21, 127.32 (*d*, *J* = 8.0 Hz), 126.33, 125.67, 122.91, 115.92 (*d*, *J* = 21.7 Hz), 109.54 (d, *J* = 37.5 Hz), 34.34, 19.41. MS (ESI): 378.1 $[M+H]^+$. HRMS (ESI) for $C_{23}H_{18}NF_2S$ $[M+H]^+$: calc'd: 378.1128; found: 378.1123.

5-(3-((5-(4-Fluorophenyl)thiophen-2-yl)methyl)-4-methylphenyl)-2-(2,2,2-trifluoroethoxy) pyridine (20).

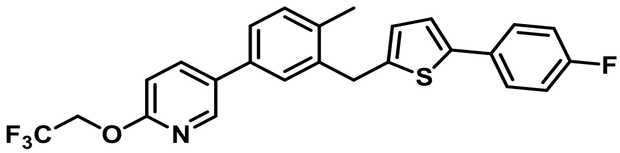

General procedure 1 with 2-fluoro-5-(3-((5-(4-fluorophenyl)thiophen-2-yl)methyl)-4-methylphenyl)pyridine (prepared as described immediately above) on 0.5 mmol scale followed by flash chromatography (ethyl acetate/*n*-hexane, 1: 10) gave **20** as a white solid (201 mg, 88% yield). Mp: 98–100 ^oC ^1H NMR (400 MHz, $CDCl_3$) δ (ppm): 8.25 (*d*, *J* = 2.0 Hz, 1H), 7.74 (*dd*, *J* = 8.5, 2.2 Hz, 1H), 7.38 (*dd*, *J* = 8.5, 5.3 Hz, 2H), 7.33–7.22 (*m*, 2H), 7.22–7.13 (*m*, 1H), 6.93 (dd, *J* = 14.7, 6.0 Hz, 3H), 6.82 (*d*, *J* = 8.6 Hz, 1H), 6.62 (*d*, *J* = 3.3 Hz, 1H), 4.71 (*q*, *J* = 8.6 Hz, 2H), 4.09 (*s*, 2H), 2.28 (*s*, 3H); ^{19}F NMR (376 MHz, $CDCl_3$) δ (ppm): –73.78 (*t*, *J* = 8.6 Hz, 3F), –103.87 to –127.09 (m, 1F); ^{13}C NMR (101 MHz, $CDCl_3$) δ (ppm): 162.33 (*d*, *J* = 246.9 Hz), 161.21, 144.67, 143.16, 141.93, 139.20, 138.22, 136.18, 135.60, 131.69, 131.42, 130.98 (*d*, *J* = 3.2 Hz), 128.01, 127.33 (*d*, *J* = 7.9 Hz), 126.31, 125.44, 123.96 (*q*, *J* = 277.6 Hz), 122.92, 115.93 (*d*, *J* = 21.7 Hz), 111.14, 62.40 (*q*, *J* = 35.8 Hz), 34.38, 19.39. MS (ESI): 458.1 $[M+H]^+$. HRMS (ESI) for $C_{25}H_{20}NOF_4S$ $[M+H]^+$: calc'd: 458.1202; found: 458.1208.

***N*,2,3-Trimethyl-*N*-(2-(2,2,2-trifluoroethoxy)pyrimidin-4-yl)-2*H*-indazol-6-amine (21).**

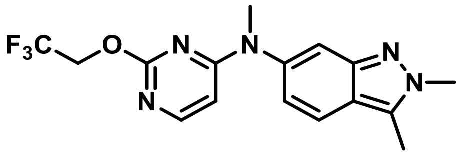

General procedure 1 with *N*-(2-chloropyrimidin-4-yl)-*N*,2,3-trimethyl-2*H*-indazol-6-amine on a 1.0 mmol scale followed by flash chromatography (ethyl acetate) gave **21** as a white solid (301 mg, 86% yield). Mp: 145–147 ^oC. ^1H NMR (400 MHz, $CDCl_3$) δ (ppm): 7.79 (*d*, *J* = 6.0 Hz, 1H), 7.61 (*d*, *J* = 8.8 Hz, 1H), 7.45 (*d*, *J* = 1.2 Hz, 1H), 6.82

(*dd*, J = 8.8, 1.7 Hz, 1H), 6.00 (*d*, J = 6.0 Hz, 1H), 4.76 (*q*, J = 8.6 Hz, 2H), 4.10 (*s*, 3H), 3.50 (*s*, 3H), 2.61 (*s*, 3H); ^{19}F NMR (376 MHz, $CDCl_3$) δ (ppm): –73.57 (*t*, J = 8.2 Hz, 3F); ^{13}C NMR (101 MHz, $CDCl_3$) δ (ppm): 164.41, 163.58, 156.10, 147.98, 142.11, 132.31, 123.67 (*q*, J = 277.9 Hz), 121.95, 120.28, 120.04, 114.88, 100.71, 63.06 (*q*, J = 36.0 Hz), 38.26, 37.67, 10.09. MS (ESI): 352.1 [*M*+H]$^+$. HRMS (ESI) for $C_{16}H_{17}N_5OF_3$ [*M*+H]$^+$: calc'd: 352.1385; found: 352.1380.

6-Bromo-8-cyclopentyl-5-methyl-2-(2,2,2-trifluoroethoxy)pyrido[2,3-*d*]pyrimidin-7(8*H*)-one (22).

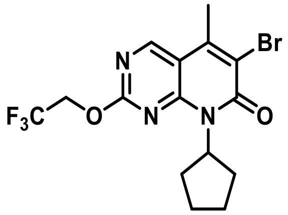

General procedure 1 with 6-bromo-2–chloro-8-cyclopentyl-5-methylpyrido[2,3-*d*]pyrimidin-7(8*H*)-one on a 0.5 mmol scale followed by flash chromatography (ethyl acetate/*n*-hexane, 1: 4) gave 22 as a white solid (158 mg, 78% yield). Mp: 205–207 °C. ^{1}H NMR (400 MHz, $CDCl_3$) δ (ppm): 8.84 (*s*, 1H), 6.66–5.64 (*m*, 1H), 4.87 (*q*, J = 8.2 Hz, 2H), 2.64 (*s*, 3H), 2.26–2.20 (*m*, 2H), 2.16–2.02 (*m*, 2H), 1.93–1.88 (*m*, 2H), 1.76–1.59 (*m*, 2H); ^{19}F NMR (376 MHz, $CDCl_3$) δ (ppm): –73.52 (*t*, J = 8.3 Hz, 3F); ^{13}C NMR (101 MHz, $CDCl_3$) δ (ppm): 162.78, 158.68, 157.45, 155.92, 143.06, 123.19 (*q*, J = 277.9 Hz), 120.23, 110.72, 64.04 (*q*, J = 36.6 Hz), 55.49, 28.53, 26.17, 18.56. MS (ESI): 406.0 [*M*+H]$^+$. HRMS (ESI) for $C_{15}H_{16}N_3O_2F_3{}^{79}Br$ [*M*+H]$^+$: calc'd: 406.0378; found: 406.0374.

2.2. Syntheses of benzenoid precursors and standards.

2.2.1. General procedure 2 for diaryliodonium precursors synthesis.[2]

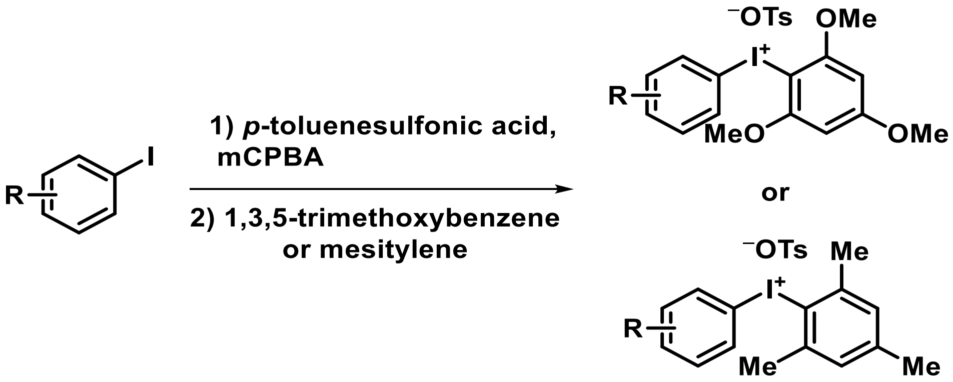

Supplementary Figure 4. General procedure 2 for the synthesis of diaryliodonium salts.

Aryl(trimethoxyphenyl)iodonium or aryl(trimethylphenyl)iodonium tosylates were prepared by known literature procedures.[2] In a closed reaction vessel, iodoarene (1 equiv.) was dissolved in acetonitrile (1 mL/mmol of iodoarene). *p*-Toluenesulfonic acid

monohydrate (1 equiv.) was then added in one portion, followed by *m*CPBA (1 equiv.) in one portion. The reaction mixture was stirred vigorously at 75 ^oC. After 30 min, the flask was raised from the oil bath and 1,3,5-trimethoxybenzene or 1,3,5-trimethylbenzene (1 equiv.) was added in one portion. The flask was returned to the oil bath and stirring was continued at 75 ^oC for 5 min (in the case of 1,3,5–trimethylbenzene, the second stage of the reaction was allowed to proceed for 3 h). The reaction was removed from heat and triturated with diethyl ether until precipitation ceased. The precipitate was isolated by vacuum filtration and washed with diethyl ether to give a white or light beige solid. Diaryliodonium salts were isolated in moderate yield. If needed, precursors were purified via recrystallization in a minimal amount of methanol and crystallized by addition of diethyl ether.

2.2.2. General procedure 3 for iodonium ylide precursors synthesis.[3]

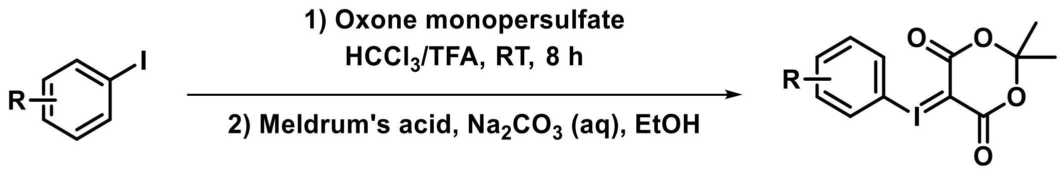

Supplementary Figure 5. General procedure 3 for the synthesis of aryliodonium ylides.

A solution of iodoarene (1 equiv.) in chloroform (1 mL/mmol of iodoarene) was treated with trifluoroacetic acid (1.1 mL/mmol) followed by oxone monopersulfate (1.6 equiv.). The heterogeneous mixture was stirred at RT for 8 h and then concentrated under reduced pressure. The residue was suspended in ethanol (4 mL/mmol) and treated with Meldrum's acid (1 equiv.) and a solution of 10% sodium carbonate in water, which was used to adjust the *p*H to ~10. The reaction was stirred at RT for 3 h and then diluted with water and extracted with DCM. The combined organic layers were dried over anhydrous sodium sulfate, filtered, and concentrated. The residue was recrystallized in a minimal amount of methanol and diethyl ether to give the corresponding aryliodonium ylide.

2.2.3. General procedure 4 for synthesis of reference compounds.[4]

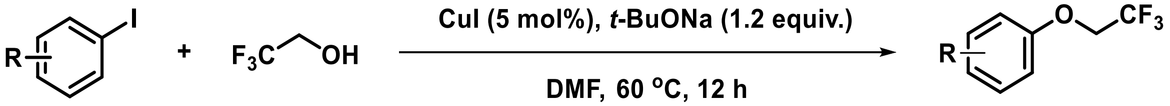

Supplementary Figure 6. General procedure 4 for the synthesis of 2,2,2-trifluoroethyl aryl ethers.

The iodoarene (1 equiv.), CuI (5 mol%), and *t*-BuONa (1.2 equiv.) were placed in a round-bottomed flask with a magnetic stirrer bar. DMF (1 M) and 2,2,2-trifluoroethanol (2 equiv.) were added under argon. The reaction mixture was stirred at 60 °C for 12 h, then quenched with water, and diluted with DCM (30 mL). The organic layer was washed with brine, dried (Mg_2SO_4) and concentrated *in vacuo*. The residue was purified by silica gel flash chromatography to afford the corresponding 2,2,2-trifluoroethyl aryl ether.

2.2.4. General procedure 5 for synthesis of reference compounds.

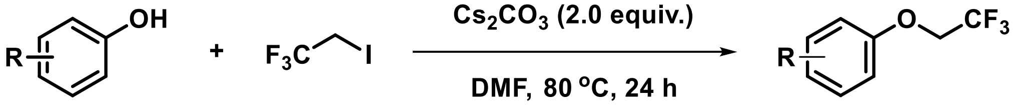

Supplementary Figure 7. General procedure 5 for the synthesis of 2,2,2-trifluoroethyl aryl ethers.

Hydroxy precursor (1 equiv.), Cs_2CO_3 (2 equiv.) 2-iodo-1,1,1-trifluoroethane (3 equiv.) and DMF (0.2 M) were added under argon to the reaction vessel. The reaction vessel was capped loosely and stirred vigorously at 80 °C for 12 h. The mixture was cooled to RT, poured onto water, and extracted with DCM (2 × 15 mL). The organic phase was dried (Mg_2SO_4) and concentrated *in vacuo*. The residue was then purified by silica gel flash chromatography to afford the corresponding 2,2,2-trifluoroethyl aryl ether.

(4-Benzyloxyphenyl)(2,4,6-trimethoxyphenyl)iodonium tosylate.

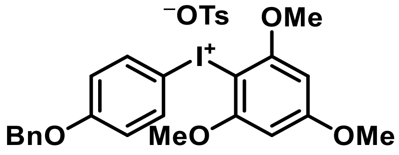

General procedure 2 on a 3.0 mmol scale gave the title compound as a white solid (1.27 g, 65% yield). Mp: 212–214 °C. ^{1}H NMR (400 MHz, DMSO-d_6) δ (ppm): 7.84 (*d*, J = 9.0 Hz, 2H), 7.47 (*d*, J = 8.1 Hz, 2H), 7.44–7.29 (*m*, 5H), 7.09 (*t*, J = 9.0 Hz, 4H), 6.45 (*s*, 2H), 5.14 (*s*, 2H), 3.94 (*s*, 6H), 3.86 (*s*, 3H), 2.28 (*s*, 3H); ^{13}C NMR (101 MHz, DMSO-d_6) δ (ppm): 165.98, 160.60, 159.23, 145.83, 137.49, 136.45, 136.17, 128.48, 128.06, 127.99, 127.78, 125.47, 117.96, 105.30, 91.97, 87.57, 69.58, 57.27, 56.11, 20.74. MS (ESI): 477.0 [*M*–OTs]$^+$. HRMS (ESI) for $C_{22}H_{22}O_4I$ [M–OTs]$^+$: calc'd: 477.0563; found: 477.0564.

1-(Benzyloxy)-4-(2,2,2-trifluoroethoxy)benzene (23).

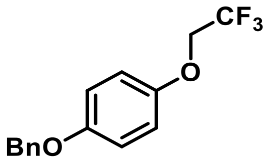

General procedure 4 on a 2.0 mmol scale followed by flash chromatography (ethyl acetate/*n*-hexane, 1: 20) gave **23** as a white solid (167 mg, 30% yield). ^{1}H NMR (400 MHz, $CDCl_3$) δ (ppm): 7.52–

7.31 (*m*, 5H), 6.97–6.90 (*m*, 4H), 5.05 (*s*, 2H), 4.31 (*q*, *J* = 8.2 Hz, 2H); ^{19}F NMR (376 MHz, $CDCl_3$) *δ* (ppm): –74.04 (*t*, *J* = 8.2 Hz, 3F); ^{13}C NMR (101 MHz, $CDCl_3$) *δ* (ppm): 154.48, 152.03, 137.19, 128.79, 128.19, 127.66, 123.63 (*q*, *J* = 278.4 Hz), 116.54, 116.12, 70.77, 67.05 (*q*, *J* = 35.3 Hz). (Known compound).[5]

(*p*-Tolyl)iodonium-(2,2-dimethyl-1,3-dioxane-4,6-dione)ylide.

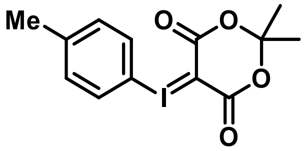

General procedure 3 on a 6.0 mmol scale gave the title compound as a white solid (630 mg, 29% yield). Mp: dec.135 °C. ^{1}H NMR (400 MHz, DMSO–d_6) *δ* (ppm): 7.67 (*d*, *J* = 8.2 Hz, 2H), 7.26 (*d*, *J* = 8.2 Hz, 2H), 2.33 (*s*, 3H), 1.55 (*s*, 6H); ^{13}C NMR (101 MHz, DMSO–d_6) *δ* (ppm): 162.83, 140.79, 132.64, 131.55, 112.87, 102.63, 58.03, 25.57, 20.69. MS (ESI): 743.0 [2*M*+Na]$^+$. HRMS (ESI) for $C_{26}H_{26}O_8I_2Na$ [2*M*+Na]$^+$: calc'd: 742.9615; found: 742.9614.

1-Methyl-4-(2,2,2-trifluoroethoxy)benzene (24).

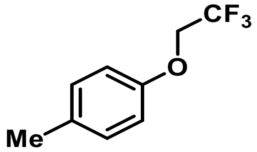

General procedure 4 on a 2.0 mmol scale followed by flash chromatography (*n*-hexane) gave **24** as a colorless oil (201 mg, 53% yield). ^{1}H NMR (400 MHz, $CDCl_3$) *δ* (ppm): 7.13 (*d*, *J* = 8.2 Hz, 2H), 6.86 (*d*, *J* = 8.2 Hz, 2H), 4.33 (*q*, *J* = 8.2 Hz, 2H), 2.32 (*s*, 3H); ^{19}F NMR (376 MHz, $CDCl_3$) *δ* (ppm): –74.05 (*t*, *J* = 8.3 Hz, 3F); ^{13}C NMR (101 MHz, $CDCl_3$) *δ* (ppm): 155.61, 132.16, 130.37, 123.66 (*q*, *J* = 278.0 Hz), 115.07, 66.32 (*q*, *J* = 35.4 Hz), 20.69. (Known compound).[6]

(4-Chlorophenyl)(2,4,6-trimethoxyphenyl)iodonium tosylate.

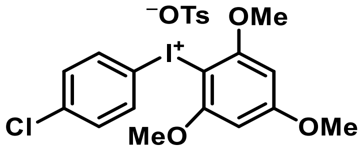

General procedure 2 on a 3.0 mmol scale gave the title compound as a white solid (1.34 g, 77% yield). Mp: 226–230 °C. ^{1}H NMR (400 MHz, DMSO-d_6) *δ* (ppm): 7.91 (*d*, *J* = 8.7 Hz, 2H), 7.53 (*d*, *J* = 8.7 Hz, 2H), 7.47 (*d*, *J* = 8.1 Hz, 2H), 7.10 (*d*, *J* = 7.9 Hz, 2H), 6.47 (*s*, 2H), 3.94 (*s*, 6H), 3.87 (*s*, 3H), 2.28 (*s*, 3H); ^{13}C NMR (101 MHz, DMSO-d_6) *δ* (ppm): 166.27, 159.33, 145.74, 137.56, 136.67, 136.11, 131.49, 128.02, 125.47, 113.92, 92.08, 87.21, 57.35, 56.18, 20.76. MS (ESI): 405.0 [*M*–OTs]$^+$. HRMS (ESI) for $C_{15}H_{15}O_3ClI$ [*M*–OTs]$^+$: calc'd: 404.9754; found: 404.9758.

1-Chloro-4-(2,2,2-trifluoroethoxy)benzene (25).

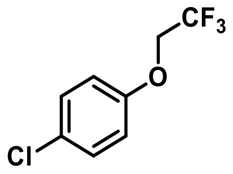

General procedure 4 on a 2.0 mmol scale followed by flash chromatography (*n*-hexane) gave **25** as a white solid (408 mg, 92% yield). ^{1}H NMR (400 MHz, $CDCl_3$) δ (ppm): 7.28 (*d*, *J* = 8.6 Hz, 2H), 6.88 (*d*, *J* = 8.6 Hz, 2H), 4.32 (*q*, *J* = 8.1 Hz, 2H); ^{19}F NMR (376 MHz, $CDCl_3$) δ (ppm): –73.95 (*t*, *J* = 8.1 Hz, 3F); ^{13}C NMR (101 MHz, $CDCl_3$) δ (ppm): 156.19, 129.89, 127.85, 123.38 (*q*, *J* = 278.0 Hz), 116.52, 66.33 (*q*, *J* = 35.8 Hz). (Known compound).[7]

(2-Bromophenyl)(2,4,6-trimethoxyphenyl)iodonium tosylate.

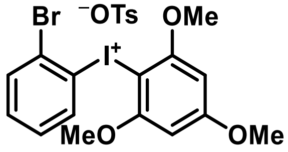

General procedure 2 on a 3.0 mmol scale gave the title compound as a white solid (1.42 g, 76% yield). Mp: 142–145 ºC. ^{1}H NMR (400 MHz, CD_3CN) δ (ppm): 7.90 (*d*, *J* = 7.9 Hz, 1H), 7.75 (*d*, *J* = 7.9 Hz, 1H), 7.46–7.43 (*m*, 3H), 7.32 (*t*, *J* = 7.7 Hz, 1H), 7.11 (*d, J* = 8.0 Hz, 2H), 6.29 (*s*, 2H), 3.87 (*s*, 6H), 3.85 (*s*, 3H), 2.32 (*s*, 3H); ^{13}C NMR (101 MHz, CD_3CN) δ (ppm): 168.31, 161.21, 145.17, 140.26, 139.13, 134.71, 134.60, 131.09, 129.46, 127.08, 126.68, 121.12, 93.03, 86.44, 58.04, 57.09, 21.37. MS (ESI): 450.9 [*M*–OTs]$^+$. HRMS (ESI) for $C_{15}H_{15}O_3BrI$ [*M*–OTs]$^+$: calc'd: 448.9249; found: 48.9254.

1-Bromo-2-(2,2,2-trifluoroethoxy)benzene (26).

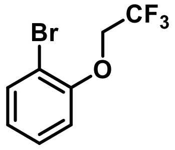

General procedure 5 on a 3.0 mmol scale followed by flash chromatography (*n*-hexane) gave **26** as a colorless oil (246 mg, 32% yield). ^{1}H NMR (400 MHz, $CDCl_3$) δ (ppm): 7.58 (*d*, *J* = 7.9 Hz, 1H), 7.29 (*t*, *J* = 7.8 Hz, 1H), 6.98–6.94 (*m*, 2H), 4.40 (*q*, *J* = 8.1 Hz, 2H); ^{19}F NMR (376 MHz, $CDCl_3$) δ (ppm): –73.90 (*t*, *J* = 8.0 Hz, 3F); ^{13}C NMR (101 MHz, $CDCl_3$) δ (ppm): 154.40, 134.12, 128.85, 124.44, 123.34 (*q*, *J* = 278.4 Hz), 115.48, 113.37, 67.46 (*q*, *J* = 35.8 Hz). (Known compound).[8]

1-Iodo-4-(2,2,2-trifluoroethoxy)benzene (27).

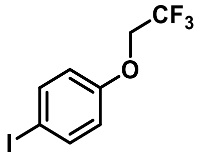

General procedure 5 on a 3.0 mmol scale followed by flash chromatography (*n*-hexane) gave **27** as a white solid (367 mg, 41% yield). ^{1}H NMR (400 MHz, $CDCl_3$) δ (ppm): 7.61 (*d*, *J* = 8.6 Hz, 2H), 6.72 (*d*, *J* = 8.7 Hz, 2H), 4.32 (*q*, *J* = 8.1 Hz, 2H); ^{19}F NMR (376 MHz, $CDCl_3$) δ (ppm): –73.91 (*t*, *J* = 8.1 Hz, 3F); ^{13}C NMR (101 MHz, $CDCl_3$) δ (ppm): 157.48, 138.80, 123.35 (*q*, *J* = 278.1 Hz), 117.45, 85.26, 66.06 (*q*, *J* = 35.9 Hz). (Known compound).[9]

(4-Methylbenzenesulfonate)(2,4,6-trimethoxyphenyl)iodonium tosylate.

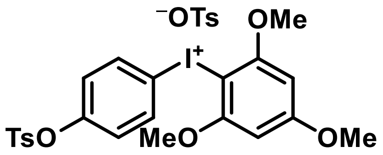

General procedure 2 on a 5.0 mmol scale gave the title compound as a white solid (2.54 g, 71% yield). Mp: 191–192 °C. ^{1}H NMR (400 MHz, DMSO) δ (ppm): 7.93 (*d*, *J* = 8.9 Hz, 2H), 7.73 (*d*, *J* = 8.3 Hz, 2H), 7.47 (*dd*, *J* = 7.9, 6.6 Hz, 4H), 7.12 (*dd*, *J* = 17.2, 8.4 Hz, 4H), 6.46 (*s*, 2H), 3.91 (*s*, 6H), 3.87 (*s*, 3H), 2.41 (*s*, 3H), 2.28 (*s*, 3H); ^{13}C NMR (101 MHz, DMSO) δ (ppm): 166.28, 159.32, 150.84, 146.24, 145.71, 137.60, 136.47, 130.93, 130.38, 128.20, 128.03, 125.48, 125.17, 113.86, 92.10, 87.17, 57.33, 56.17, 21.17, 20.76. MS (ESI): 541.0 [*M*–OTs]$^+$. HRMS (ESI) for $C_{22}H_{22}O_6SI$ [*M*–OTs]$^+$: calc'd: 541.0182; found: 541.0184.

4-(2,2,2-Trifluoroethoxy)phenyl 4-methylbenzenesulfonate (28).

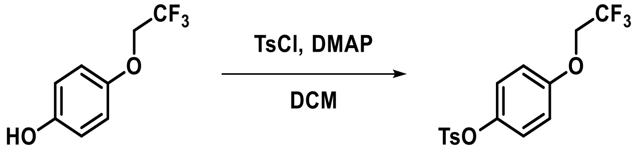

To a round-bottomed flask with a magnetic stirrer bar was added 4-(2,2,2-trifluoroethoxy)phenol (96 mg, 0.5 mmol, 1.0 equiv.), DMAP (122 mg, 1.0 mmol, 2.0 equiv.) and DCM (3 mL) under argon. Tosyl chloride (114 mg, 0.6 mmol, 1.2 equiv.) in DCM (1 mL) was added dropwise at 0 °C and the reaction mixture was stirred at RT for 12 h. After completion of the reaction, the reaction mixture was poured onto water and extracted with DCM (2 × 15 mL). The organic phase was separated, dried over Mg_2SO_4, and concentrated *in vacuo*. The residue was purified by flash chromatography (ethyl acetate/*n*-hexane, 1: 10) to give **28** as a colorless oil (156 mg, 90% yield). Mp: 75–77 °C. ^{1}H NMR (400 MHz, $CDCl_3$) δ (ppm): 7.67 (*d*, *J* = 8.3 Hz, 1H), 7.30 (*d*, *J* = 8.0 Hz, 2H), 6.9. 8–6.87 (*m*, 2H), 6.87–6.76 (*m*, 2H), 4.30 (*q*, *J* = 8.1 Hz, 2H), 2.44 (*s*, 3H); ^{19}F NMR (376 MHz, $CDCl_3$) δ (ppm): –73.96 (t, *J* = 8.0 Hz, 3F); ^{13}C NMR (101 MHz, $CDCl_3$) δ (ppm): 156.04, 145.71, 144.51, 132.24, 129.97, 128.66, 123.87, 123.31 (*q*, *J* = 278.0 Hz), 115.87, 66.21 (*q*, *J* = 35.8 Hz), 21.82.

(4-Nitrophenyl)iodonium-(2,2-dimethyl-1,3-dioxane-4,6–dione)ylide.

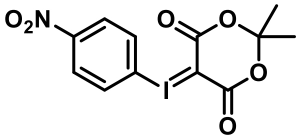

General procedure 3 on a 3.0 mmol scale gave the title compound as a white solid (895 mg, 38% yield). Mp: dec. 131 °C. ^{1}H NMR (400 MHz, DMSO-d_6) δ (ppm): 8.28 (*d*, *J* = 8.4 Hz, 2H), 8.05 (*d*, *J*

= 8.4 Hz, 2H), 1.59 (*s*, 6H); ^{13}C NMR (101 MHz, DMSO-d_6) *δ* (ppm): 162.93, 148.91, 133.51, 125.63, 122.94, 103.04, 58.45, 25.63. MS (ESI): 804.9 [2*M*+Na]$^+$. HRMS (ESI) for $C_{24}H_{20}N_2O_{12}I_2Na$ [2*M*+Na]$^+$: calc'd: 804.9003; found: 804.9008.

1-Nitro-4-(2,2,2-trifluoroethoxy)benzene (29).

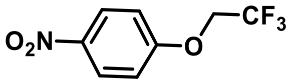

General procedure 4 on a 2.0 mmol scale followed by flash chromatography (ethyl acetate/*n*-hexane, 1: 10) gave **29** as a pale-yellow solid (408 mg, 92% yield). ^{1}H NMR (400 MHz, CDCl$_3$) *δ* (ppm): 8.25 (*d*, *J* = 8.9 Hz, 2H), 7.04 (*d*, *J* = 8.9 Hz, 2H), 4.46 (*q*, *J* = 7.8 Hz, 2H); ^{19}F NMR (376 MHz, CDCl$_3$) *δ* (ppm): –73.72 (*t*, *J* = 8.0 Hz, 3F); ^{13}C NMR (101 MHz, CDCl$_3$) *δ* (ppm): 161.96, 143.04, 126.26, 122.99 (*q*, *J* = 278.1 Hz), 115.06, 65.98 (*q*, *J* = 36.5 Hz). (Known compound).[10]

(4-Ethoxycarbonylphenyl)(mesityl)iodonium tosylate.

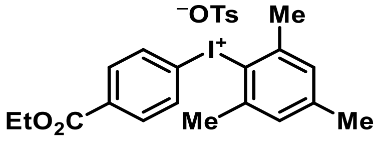

General procedure 2 on a 5.0 mmol scale gave the title compound as a white solid (1.98 g, 70% yield). Mp: 165–167 ^{o}C. ^{1}H NMR (400 MHz, CDCl$_3$) *δ* (ppm): 7.86 (*d*, *J* = 8.2 Hz, 2H), 7.75 (*d*, *J* = 8.2 Hz, 2H), 7.37 (*d*, *J* = 7.8 Hz, 2H), 6.97–6.95 (*m*, 2H), 4.32 (*q*, *J* = 7.1 Hz, 2H), 2.53 (*s*, 6H), 2.28 (*s*, 6H), 1.33 (*t*, *J* = 7.1 Hz, 3H); ^{13}C NMR (101 MHz, CDCl$_3$) *δ* (ppm): 165.24, 143.54, 142.45, 142.38, 139.50, 133.23, 132.78, 132.14, 129.89, 128.52, 126.02, 122.57, 118.73, 61.69, 27.17, 21.40, 21.21, 14.33. MS (ESI): 395.0 [*M*–OTs]$^+$. HRMS (ESI) for $C_{18}H_{20}O_2I$ [*M*–OTs]$^+$: calc'd: 395.0508; found: 395.0508.

Ethyl 4-(2,2,2-trifluoroethoxy)benzoate (30).

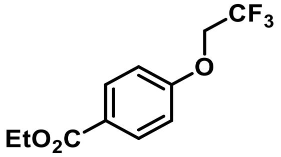

General procedure 4 on a 2.0 mmol scale followed by flash chromatography (ethyl acetate/*n*-hexane, 1: 20) gave **30** as a white solid (412 mg, 83% yield). ^{1}H NMR (400 MHz, CDCl$_3$) *δ* (ppm): 8.02 (*d*, *J* = 8.5 Hz, 2H), 6.95 (*d*, *J* = 8.5 Hz, 2H), 4.67–4.07 (*m*, 4H), 1.38 (*t*, *J* = 7.1 Hz, 2H); ^{19}F NMR (376 MHz, CDCl$_3$) *δ* (ppm): –73.90 (*t*, *J* = 8.1 Hz, 3F); ^{13}C NMR (101 MHz, CDCl$_3$) *δ* (ppm): 166.14, 160.85, 131.91, 125.02, 123.30 (*q*, *J* = 277.9 Hz), 114.51, 65.72 (*d*, *J* = 36.2 Hz), 61.08 (*s*), 14.51. (Known compound).[6]

(4-Cyanophenyl)(2,4,6-trimethoxyphenyl)iodonium tosylate.

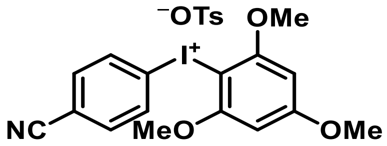

General procedure 2 on a 3.0 mmol scale gave the title compound as a pink solid (0.73 g, 43% yield). Mp: dec. 201 ^{o}C. ^{1}H NMR (400 MHz, DMSO-d_6) *δ* (ppm): 8.06 (*d*, *J* = 8.4

Hz, 2H), 7.90 (*d*, *J* = 8.4 Hz, 2H), 7.47 (*d*, *J* = 8.0 Hz, 2H), 7.11 (*d*, *J* = 7.8 Hz, 2H), 6.48 (*s*, 2H), 3.93 (*s*, 6H), 3.88 (*s*, 3H), 2.28 (*s*, 3H); ^{13}C NMR (101 MHz, DMSO-d_6) δ (ppm): 166.49, 159.43, 145.57, 137.68, 134.81, 128.07, 125.48, 120.96, 117.54, 114.09, 92.18, 86.99, 57.40, 56.23, 20.78. MS (ESI): 396.0 [*M*–OTs]$^+$. HRMS (ESI) for $C_{16}H_{15}NO_3I$ [*M*–OTs]$^+$: calc'd: 396.0097; found: 396.0093.

4-(2,2,2-Trifluoroethoxy)benzonitrile (31).

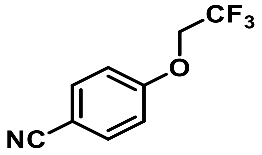

General procedure 4 on a 2.0 mmol scale followed by flash chromatography (ethyl acetate/*n*-hexane, 1: 10) gave **31** as a white solid (330 mg, 82% yield). ^{1}H NMR (400 MHz, $CDCl_3$) δ (ppm): 7.63 (*d*, *J* = 8.2 Hz, 2H), 7.01 (*d*, *J* = 8.2 Hz, 2H), 4.41 (*q*, *J* = 7.9 Hz, 2H); ^{19}F NMR (376 MHz, $CDCl_3$) δ (ppm): –73.77 (*t*, *J* = 8.2 Hz, 3F); ^{13}C NMR (101 MHz, $CDCl_3$) δ (ppm): 160.40, 134.40, 123.05 (*q*, *J* = 278.0 Hz), 118.74, 115.67, 106.25, 65.68 (*q*, *J* = 36.4 Hz). (Known compound).[10]

(3-Cyanophenyl) (2,4,6-trimethoxyphenyl)iodonium tosylate.

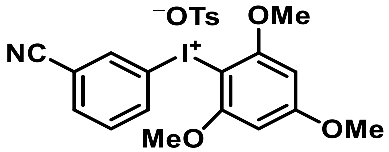

General procedure 2 on a 3.0 mmol scale gave the title compound as a white solid (1.13 g, 66% yield). Mp: 212–214 ^{o}C. ^{1}H NMR (400 MHz, $CDCl_3$) δ (ppm): 8.25 (*d*, *J* = 8.3 Hz, 1H), 7.91 (*s*, 1H), 7.64 (*d*, *J* = 7.8 Hz, 1H), 7.52 (*d*, *J* = 8.1 Hz, 2H), 7.39 (*t*, *J* = 8.0 Hz, 1H), 7.06 (*d*, *J* = 8.0 Hz, 2H), 6.12 (*s*, 2H), 3.83 (*s*, 9H), 2.32 (*s*, 3H); ^{13}C NMR (101 MHz, $CDCl_3$) δ (ppm): 167.18, 160.53, 142.86, 139.57, 138.95, 136.79, 134.34, 131.56, 128.66, 126.11, 116.99, 116.12, 114.78, 91.84, 86.06, 57.18, 56.11, 21.47. MS (ESI): 396.0 [*M*–OTs]$^+$. HRMS (ESI) for $C_{16}H_{15}NO_3I$ [M–OTs]$^+$: calc'd: 396.0097; found: 396.0097.

3-(2,2,2-Trifluoroethoxy)benzonitrile (32).

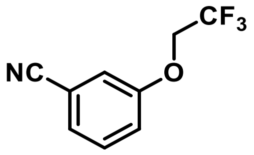

General procedure 4 on a 2.0 mmol scale followed by flash chromatography (ethyl acetate/*n*-hexane, 1: 10) gave **32** as a white solid (235 mg, 58% yield). ^{1}H NMR (400 MHz, $CDCl_3$) δ (ppm): 7.49–7.40 (*m*, 1H), 7.34 (*d*, *J* = 7.6 Hz, 1H), 7.23–7.15 (*m*, 2H), 4.39 (*q*, *J* = 8.0 Hz, 2H); ^{19}F NMR (376 MHz, $CDCl_3$) δ (ppm): –73.86 (*t*, *J* = 8.1 Hz, 3F); ^{13}C NMR (101 MHz, $CDCl_3$) δ (ppm): 157.51, 130.95, 126.50, 123.13 (*q*, *J* = 278.0 Hz), 120.14, 118.28, 118.23, 113.80, 66.05 (*q*, *J* = 36.1 Hz). (Known compound).[11]

(3-Chloro-4-(methoxycarbonyl)phenyl)(2,4,6-trimethoxyphenyl) tosylate.

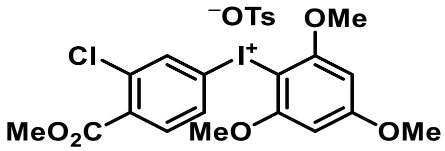

General procedure 2 on a 3.0 mmol scale gave the title compound as a white solid (1.53 g, 81% yield). Mp: 214–216 ^{o}C. ^{1}H NMR (400 MHz, $CDCl_3$) δ (ppm): 8.27 (*d*, *J* = 2.3 Hz, 1H), 7.96 (*dd*, *J* = 8.7, 2.3 Hz, 1H), 7.52 (*d*, *J* = 8.1 Hz, 2H), 7.29 (*d*, *J* = 8.7 Hz, 1H), 7.03 (*d*, *J* = 7.9 Hz, 2H), 6.11 (*s*, 2H), 3.87 (*s*, 3H), 3.84 (*s*, 6H), 3.82 (*s*, 3H), 2.30 (*s*, 3H); ^{13}C NMR (101 MHz, $CDCl_3$) δ (ppm): 167.11, 164.16, 160.50, 142.91, 139.45, 138.43, 137.50, 137.28, 133.63, 132.08, 128.56, 126.10, 112.96, 91.78, 85.54, 57.11, 56.13, 53.01, 21.46. MS (ESI): 463.0 [*M*–OTs]$^{+}$. HRMS (ESI) for $C_{17}H_{17}O_5ClI$ [*M*–OTs]$^{+}$: calc'd: 462.9809; found: 462.9814.

Methyl 2-chloro-5-(2,2,2-trifluoroethoxy)benzoate (33).

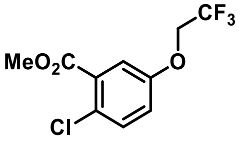

General procedure 4 on a 2.0 mmol scale followed by flash chromatography (ethyl acetate/*n*-hexane, 1: 20) gave **33** as a white solid (230 mg, 43% yield).^{1}H NMR (400 MHz, $CDCl_3$) δ (ppm): 7.38 (*s*, 1H), 7.37 (*d*, *J* = 5.8 Hz, 1H), 7.02 (*dd*, *J* = 8.8, 3.2 Hz, 1H), 4.36 (*q*, *J* = 8.0 Hz, 2H), 3.92 (*s*, 3H); ^{19}F NMR (376 MHz, $CDCl_3$) δ (ppm): –73.93 (*t*, *J* = 8.1 Hz, 3F); ^{13}C NMR (101 MHz, $CDCl_3$) δ (ppm): 165.63, 155.76, 132.48, 131.03, 127.35, 123.21 (*d*, *J* = 278.0 Hz), 120.00, 117.36, 66.28 (*q*, *J* = 36.1 Hz), 52.78. (Known compound).[12]

Naphthalen-1-yl(2,4,6-trimethoxyphenyl)iodonium tosylate.

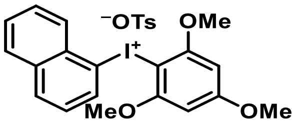

General procedure 2 on a 3.0 mmol scale gave the title compound as a white solid (1.23 g, 69% yield). Mp: 189–191 ^{o}C. ^{1}H NMR (400 MHz, DMSO-*d*$_6$) δ (ppm): 8.47 (*d*, *J* = 6.7 Hz, 1H), 8.28–8.13 (*m*, 2H), 8.02 (*d*, *J* = 8.1 Hz, 1H), 7.87–7.77 (m, 1H), 7.70 (*t*, *J* = 7.2 Hz, 1H), 7.56 (*t*, *J* = 7.8 Hz, 1H), 7.47 (*d*, *J* = 8.0 Hz, 2H), 7.10 (*d*, *J* = 7.8 Hz, 2H), 6.39 (*s*, 2H), 3.95 (s, 6H), 3.80 (*s*, 3H), 2.28 (*s*, 3H); ^{13}C NMR (101 MHz, DMSO-*d*$_6$) δ (ppm): 165.92, 159.36, 145.83, 137.49, 137.42, 134.01, 132.99, 130.98, 129.21, 129.19, 129.17, 128.00, 127.75, 127.37, 125.47, 119.24, 92.01, 87.00, 57.18, 56.06, 20.75. MS (ESI): 421.0 [*M*–OTs]$^{+}$. HRMS (ESI) for $C_{19}H_{18}O_3I$ [*M*–OTs]$^{+}$: calc'd: 421.0301; found: 421.0300.

1-(2,2,2-Trifluoroethoxy)naphthalene (2).

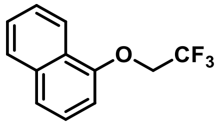

General procedure 4 on a 2.0 mmol scale followed by flash chromatography (*n*-hexane) gave **2** as a white solid (312 mg, 69% yield). ^{1}H NMR (400 MHz, $CDCl_3$) δ (ppm): 8.53–8.22 (*m*, 1H), 7.95–7.77 (*m*, 1H), 7.58–7.56 (*m*, 3H), 7.40 (*t*, J = 8.0 Hz, 1H), 6.79 (*d*, J = 7.6 Hz, 1H), 4.53 (*q*, J = 8.0 Hz, 2H); ^{19}F NMR (376 MHz, $CDCl_3$) δ (ppm): –73.71 (*t*, J = 8.0 Hz, 3F); ^{13}C NMR (101 MHz, $CDCl_3$) δ (ppm): 153.31, 134.78, 127.72, 127.07, 126.07, 125.58, 123.71 (*q*, J = 277.9 Hz), 122.42, 121.98, 105.62, 66.23 (*q*, J = 35.8 Hz). (Known compound).[10]

Naphthalen-2-yl(2,4,6-trimethoxyphenyl)iodonium tosylate.

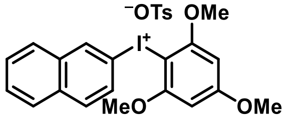

General procedure 2 on a 3.0 mmol scale gave the title compound as a white solid (1.26 g, 71% yield). Mp: 217–220 ºC. ^{1}H NMR (400 MHz, DMSO-d_6) δ (ppm): 8.65 (*d*, J = 1.3 Hz, 1H), 8.12–8.02 (*m*, 1H), 8.00 (*d*, J = 8.9 Hz, 2H), 7.91 (*dd*, J = 8.8, 1.8 Hz, 1H), 7.71–7.58 (*m*, 2H), 7.47 (*d*, J = 8.1 Hz, 2H), 7.09 (*d*, J = 7.9 Hz, 2H), 6.46 (*s*, 2H), 3.97 (*s*, 6H), 3.85 (*s*, 3H), 2.27 (*s*, 3H); ^{13}C NMR (101 MHz, DMSO-d_6) δ (ppm): 166.11, 159.39, 145.76, 137.53, 135.16, 133.77, 133.20, 131.13, 129.81, 128.62, 128.15, 128.00, 127.98, 127.70, 125.47, 113.07, 92.07, 87.12, 57.33, 56.13, 20.75. MS (ESI): 421.0 [*M*–OTs]$^+$. HRMS (ESI) for $C_{19}H_{18}O_3I$ [*M*–OTs]$^+$: calc'd: 421.0301; found: 421.0302.

2-(2,2,2-Trifluoroethoxy)naphthalene (34).

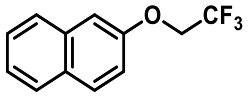

General procedure 4 on a 2.0 mmol scale followed by flash chromatography (*n*-hexane) gave **34** as a white solid (253 mg, 56% yield). ^{1}H NMR (400 MHz, $CDCl_3$) δ (ppm): 7.91–7.68 (*m*, 3H), 7.54 (*t*, J = 7.5 Hz, 1H), 7.46 (*t*, J = 7.5 Hz, 1H), 7.26 (*dd*, J = 9.0, 2.0 Hz, 1H), 7.18 (*s*, 1H), 4.49 (*q*, J = 8.1 Hz, 2H); ^{19}F NMR (376 MHz, $CDCl_3$) δ (ppm): –73.73 (*t*, J = 8.1 Hz, 3F); ^{13}C NMR (101 MHz, $CDCl_3$) δ (ppm): 155.43, 134.31, 130.17, 129.87, 127.95, 127.12, 126.97, 124.72, 123.61 (*q*, J = 277.9 Hz), 118.49, 107.69, 65.90 (*q*, J = 35.7 Hz). (Known compound).[10]

(Quinolin-6-yl)iodonium-(2,2-dimethyl-1,3-dioxane-4,6-dione)ylide.

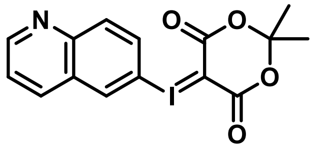

General procedure 3 on a 5.0 mmol scale gave the title compound as a white solid (640 mg, 32% yield). Mp: dec. 165 ºC. ^{1}H NMR (400 MHz, DMSO-d_6) δ (ppm): 9.01 (*dd*, J = 4.2, 1.6 Hz, 1H), 8.74–8.37 (*m*, 2H), 8.27–7.88 (*m*, 2H), 7.65 (*dd*, J = 8.4, 4.2 Hz, 1H), 1.57 (*s*, 6H);

^{13}C NMR (101 MHz, DMSO-d_6) δ (ppm): 162.92, 152.56, 147.60, 136.27, 133.41, 132.32, 131.78, 128.81, 122.69, 113.88, 102.80, 58.49, 25.58. (Known compound).[3]

6-(2,2,2-Trifluoroethoxy)quinoline (35).

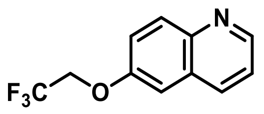

General procedure 4 on a 1.0 mmol scale followed by flash chromatography (ethyl acetate/*n*-hexane, 1: 10) gave **35** as a white solid (86 mg, 38% yield). Mp: 76–78 °C. ^1H NMR (400 MHz, $CDCl_3$) δ (ppm): 8.81 (*dd*, J = 4.2, 1.6 Hz, 1H), 8.20–7.81 (*m*, 2H), 7.39 (*ddd*, J = 20.8, 8.8, 3.5 Hz, 2H), 7.07 (*d*, J = 2.8 Hz, 1H), 4.46 (*q*, J = 8.0 Hz, 2H); ^{19}F NMR (376 MHz, $CDCl_3$) δ (ppm): –73.73 (*t*, J = 8.1 Hz, 3F); ^{13}C NMR (101 MHz, $CDCl_3$) δ (ppm): 155.47, 149.11, 145.14, 135.13, 131.74, 129.06, 123.41 (*q*, J = 277.9 Hz), 121.91, 121.90, 107.23, 66.04 (*q*, J = 36.0 Hz). MS (ESI): 228.1 [2*M*+Na]$^+$. HRMS (ESI) for $C_{24}H_{20}N_2O_{12}I_2Na$ [2*M*+Na]$^+$: calc'd: 228.0636; found: 228.0636.

(*R*)-5-((4-Chloro-3-(4-((tetrahydrofuran-3-yl)oxy)benzoyl)phenyl) iodonium-(2,2-dimethyl-1,3-dioxane-4,6-dione)ylide.

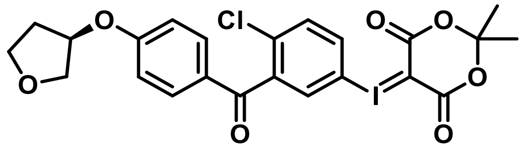

General procedure 3 on a 5.0 mmol scale gave the title compound as a pale–yellow solid (860 mg, 30% yield). Mp: dec. 140 °C. ^1H NMR (400 MHz, $CDCl_3$) δ (ppm): 7.96–7.76 (*m*, 2H), 7.68 (*d*, J = 8.8 Hz, 2H), 7.44 (*d*, J = 8.6 Hz, 1H), 6.88 (*d*, J = 8.9 Hz, 2H), 4.99 (*dd*, J = 5.8, 4.2 Hz, 1H), 4.11–3.93 (*m*, 3H), 3.89 (*t*d, J = 8.4, 4.3 Hz, 1H), 2.25 (*td*, J = 14.3, 8.3 Hz, 1H), 2.17–2.03 (*m*, 1H), 1.63 (*s*, 6H); ^{13}C NMR (101 MHz, $CDCl_3$) δ (ppm): 191.07, 163.86, 162.85, 141.85, 135.57, 135.33, 133.36, 133.30, 132.90, 128.35, 115.62, 111.97, 104.95, 78.12, 73.08, 67.34, 56.72, 33.12, 26.02 .

(*R*)-(2-Chloro-5-(2,2,2-trifluoroethoxy)phenyl)(4-((tetrahydrofuran-3-yl)oxy)phenyl)methanone (36).

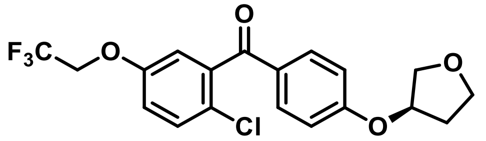

General procedure 4 on a 1.0 mmol scale followed by flash chromatography (ethyl acetate/*n*-hexane, 1: 10) gave **36** as a white solid (232 mg, 58% yield). Mp: 91–93 °C. ^1H NMR (400 MHz, $CDCl_3$) δ (ppm): 7.77 (*d*, J = 8.8 Hz, 2H), 7.38 (*d*, J = 8.8 Hz, 1H), 7.01 (*dd*, J = 8.8, 3.0 Hz, 1H), 6.97–6.80 (*m*, 3H), 5.00 (*td*, J = 4.2, 2.2 Hz, 1H), 4.35 (*q*, J = 8.0 Hz, 2H), 4.21–3.95 (*m*, 3H), 3.91 (*td*, J = 8.4, 4.4 Hz, 1H), 2.26 (*dtd*, J =

14.4, 8.3, 6.2 Hz, 1H), 2.19–2.00 (*m*, 1H); ^{19}F NMR (376 MHz, $CDCl_3$) *δ* (ppm): –73.85 (*t*, *J* = 8.2 Hz, 3F); ^{13}C NMR (101 MHz, $CDCl_3$) *δ* (ppm): 193.04, 162.36, 156.05, 140.23, 132.77, 131.40, 129.29, 124.54, 123.21 (*q*, *J* = 278.1 Hz), 117.94, 115.35, 115.17, 78.01, 73.15, 67.37, 66.29 (*q*, *J* = 36.0 Hz), 33.18. MS (ESI): 401.1 [*M*+H]$^+$. HRMS (ESI) for $C_{19}H_{17}O_4F_3Cl$ [2*M*+Na]$^+$: calc'd: 401.0767; found: 401.0773.

3-(2-Chloro-6-2,2,2-trifluoroethoxy)phenyl)-5-methylisoxazole-4-carbonitrile (37).

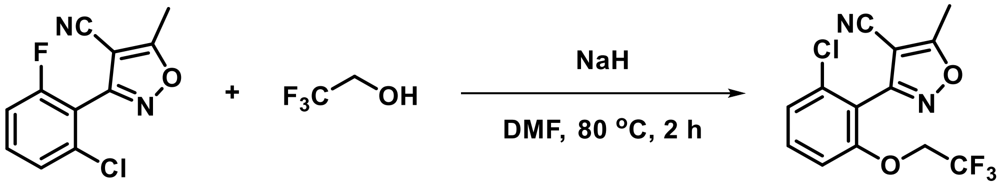

To a round bottomed flask equipped with a magnetic stirrer bar was added dry NaH (27 mg, 1.0 mmol, 2 equiv.) and DMF (2 mL, 0.5 M) under argon, and then 2,2,2-trifluoroethanol (100 mg, 1.0 mmol, 2 equiv.) in DMF (1 mL) at 0 °C. The reaction mixture was stirred for 20 min under argon and (3-(5-(2-fluorophenyl)-1,2,4-oxadiazol-3-yl)phenyl)methanol (237 mg, 1.0 mmol, 1 equiv.) in DMF (2 mL) was added dropwise at RT. The reaction flask was capped loosely and stirred vigorously at 80 °C for 2 h. The reaction mixture was then poured onto water and extracted with DCM (2 × 15 mL). The organic phase was dried (Mg_2SO_4) and concentrated *in vacuo*. The residue was purified by silica gel flash chromatography (ethyl acetate/*n*-hexane, 1: 4) to give **37** as a colorless oil (235 mg, 74% yield). ^{1}H NMR (400 MHz, $CDCl_3$) *δ* (ppm): 7.36 (*t, J* = 8.3 Hz, 1H), 7.18 (*d*, *J* = 8.5 Hz, 1H), 6.86 (*d*, *J* = 8.4 Hz, 1H), 4.32 (*q*, *J* = 7.9 Hz, 2H), 2.63 (*s*, 3H); ^{19}F NMR (376 MHz, $CDCl_3$) *δ* (ppm): –73.76 (*t*, *J* = 8.2 Hz, 3F); ^{13}C NMR (101 MHz, $CDCl_3$) *δ* (ppm): 177.15, 157.33, 156.57, 135.71, 132.73, 124.48, 122.90 (*q*, *J* = 278.3 Hz), 116.23, 111.49, 110.71, 93.77, 66.87 (*q*, *J* = 36.3 Hz), 12.69.

(3-(5-(2-Fluorophenyl)-1,2,4-oxadiazol-3-yl)phenyl)methanol.

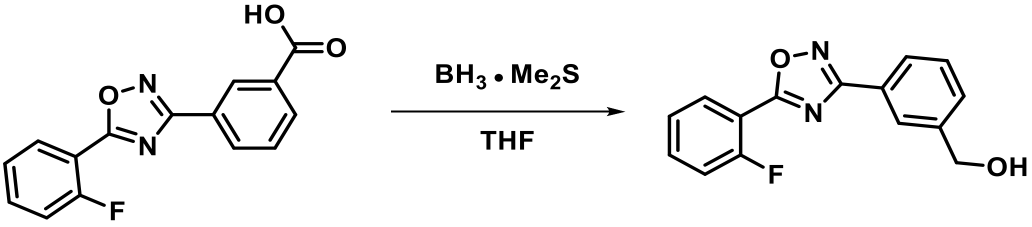

Ataluren (2.84 g, 10 mmol, 1.0 equiv.) was dissolved in THF (20 mL) and $BH_3 \cdot Me_2S$ (1.52 g, 20 mmol, 2.0 equiv.) was added dropwise at 0 °C under argon. The mixture was stirred at RT for 12 h and then quenched with methanol, washed with saturated NH_4Cl, and extracted with EtOAc (2 × 20 mL). The organic layer was dried (Mg_2SO_4), filtered, and concentrated under reduced pressure. The residue was purified by silica gel chromatography (ethyl acetate-*n*-hexane, 1: 2) to give the title compound as a white solid (2.34 g, 87% yield). Mp: 102–104 °C. ^1H NMR (400 MHz, $CDCl_3$) δ (ppm): 8.26–8.11 (*m*, 2H), 8.06 (*d*, *J* = 7.4 Hz, 1H), 7.64–7.54 (*m*, 1H), 7.49 (*dt*, *J* = 15.1, 7.6 Hz, 2H), 7.29 (*dt*, *J* = 19.4, 7.8 Hz, 2H), 4.76 (*s*, 2H), 2.49 (*s*, 1H); ^{19}F NMR (376 MHz, $CDCl_3$) δ (ppm): –108.25 (*m*, 1F), ^{13}C NMR (101 MHz, $CDCl_3$) δ (ppm): 172.91 (*d*, *J* = 4.4 Hz), 168.74, 160.90 (*d*, *J* = 260.5 Hz), 141.97, 134.80 (*d*, *J* = 8.7 Hz), 131.08, 129.92, 129.27, 126.99, 126.86, 126.12, 124.87 (*d*, *J* = 3.7 Hz), 117.31 (*d*, *J* = 21.1 Hz), 112.91 (*d*, *J* = 11.5 Hz), 64.93. MS (ESI): 271.1 $[M+H]^+$. HRMS (ESI) for $C_{15}H_{12}N_2O_2F$ $[M+H]^+$: calc'd: 271.0883; found: 271.0884.

(3-(5-(2-(2,2,2-trifluoroethoxy)phenyl)-1,2,4-oxadiazol-3-yl)phenyl)methanol (38).

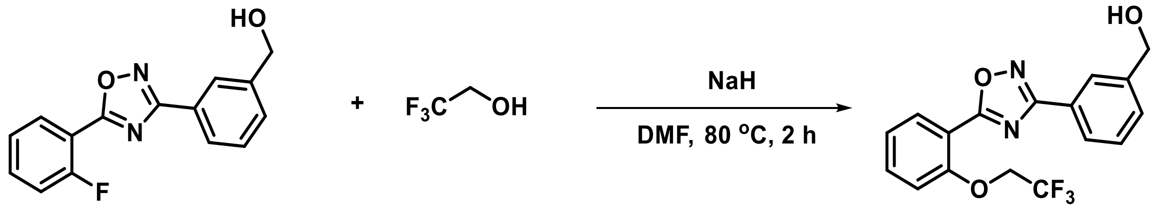

To a round bottomed flask equipped with a magnetic stirrer bar was added dry NaH (27 mg, 1.0 mmol, 2 equiv.) and DMF (2 mL, 0.5 M) under argon, followed by 2,2,2-trifluoroethanol (100 mg, 1.0 mmol, 2 equiv.) in DMF (1 mL) at 0 °C. The reaction mixture was stirred for 20 min under argon. (3-(5-(2-Fluorophenyl)-1,2,4-oxadiazol-3-yl)phenyl)methanol (135 mg, 1.0 mmol, 1 equiv.) in DMF (2 mL) was then added dropwise at RT. The reaction flask was loosely capped and stirred vigorously at 80 °C for 2 h. The reaction mixture was poured onto water and extracted with DCM (2 ×15 mL). The organic phase was dried (Mg_2SO_4), filtered, and concentrated *in vacuo*. The residue was purified by silica gel flash chromatography (ethyl acetate/*n*-hexane, 1: 1) to give **38** as a white solid (117 mg, 67% yield). Mp: 100–102 °C.^1H NMR (400 MHz, CD_3CN) δ (ppm): 8.14 (*dd*, *J* = 7.8, 1.7 Hz, 2H), 8.10 (s, 1H), 8.00 (*dt*, *J* = 6.5, 1.9 Hz, 1H), 7.63 (*ddd*, *J* = 8.6, 7.5, 1.7 Hz, 1H), 7.55–7.44 (*m*, 2H), 7.32–7.13 (*m*, 1H), 5.04–4.37 (*m*, 4H), 3.44 (*t*, *J* =

5.9 Hz, 1H); ^{19}F NMR (376 MHz, CD_3CN) δ (ppm): –74.45 (*t*, J = 8.3 Hz, 3F); ^{13}C NMR (101 MHz, CD_3CN) δ (ppm): 175.57, 169.26, 157.05, 144.24, 135.55, 132.57, 130.54, 130.08, 127.95, 126.84, 126.34, 124.80 (*q*, J = 277.1 Hz), 123.94, 115.83, 115.19, 67.38 (*q*, J = 35.4 Hz), 64.37. MS (ESI): 351.1 $[M+H]^+$. HRMS (ESI) for $C_{17}H_{14}N_2O_3F_3$ $[M+H]^+$: calc'd: 351.0957; found: 351.0963.

2.3. The synthesis of alkyl precursors and standards.

2.3.1. General procedure 6 for precursors synthesis.

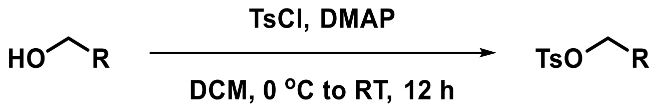

Supplementary Figure 8. General procedure 6 for the synthesis of alkyl 4–methylbenzenesulfonates.

To a round-bottomed flask equipped with a magnetic stirrer bar was added the alcohol (RCH_2OH; 1.0 equiv.), DMAP (1.5 equiv.), and DCM (20 mL) under argon. Tosyl chloride (1.2 equiv.) in DCM (3 mL) was then added dropwise at 0 °C. The reaction mixture was then stirred at RT for 12 h, poured onto water, and extracted with DCM (2 × 15 mL). The organic phase was dried (Mg_2SO_4) and concentrated *in vacuo*. The residue was purified by silica gel flash chromatography to give the desired alkyl 4-methylbenzenesulfonate in moderate to high yield.

2.3.2. General procedure 7 for synthesis of reference standards.

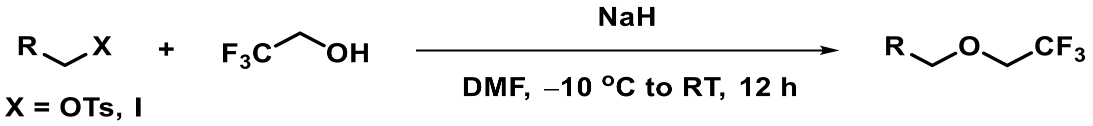

Supplementary Figure 9. General procedure 7 for the synthesis of 2,2,2-trifluoroethyl alkyl ethers.

To a round-bottomed flask equipped with a magnetic stirrer bar was added dry NaH (2 equiv.) and DMF (0.5 M) under argon, followed by 2,2,2-trifluoroethanol (2.0 equiv.) in DMF (1.0 mL) at 0 °C. The reaction mixture was stirred for 20 min under argon. An alkyl *p*-toluenesulfonate or iodoalkane (1 equiv.) in DMF (1 mL) was then added dropwise and the reaction temperature was kept under –10 °C. The reaction flask was loosely capped. The were contents stirred vigorously below –10 °C for 1 h, warmed to RT, and stirred for another 10 h. The reaction mixture was then poured into water and extracted with DCM (2 × 15 mL). The organic phase was dried (Mg_2SO_4), filtered and concentrated *in vacuo*.

The residue was purified by silica gel flash chromatography to give the corresponding 2,2,2-trifluoroethyl alkyl ether.

3-(1,3-Dioxoisoindolin-2-yl)propyl 4-methylbenzenesulfonate.

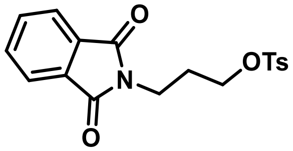

General procedure 6 with 2-(3-hydroxypropyl)isoindoline-1,3-dione on 5.0 mmol followed by flash chromatography (ethyl acetate/*n*-hexane, 1: 2) gave the title compound as a white solid (1.58 g, 88% yield). Mp: 105–107 °C. ^{1}H NMR (400 MHz, $CDCl_3$) *δ* (ppm): 7.80 (*dt*, *J* = 7.0, 3.5 Hz, 2H), 7.76 (*d*, *J* = 8.3 Hz, 2H), 7.73–7.65 (*m*, 2H), 7.31 (*d*, *J* = 8.0 Hz, 2H), 4.09 (*t*, *J* = 6.3 Hz, 2H), 3.72 (*t*, *J* = 7.0 Hz, 2H), 2.42 (*s*, 3H), 2.04 (*quint*, *J* = 6.5 Hz, 2H); ^{13}C NMR (101 MHz, $CDCl_3$) *δ* (ppm): 168.23, 145.00, 134.21, 132.99, 132.11, 130.02, 128.11, 123.47, 67.94, 34.76, 28.14, 21.81. MS (ESI): 360.1 [*M*+H]$^+$. HRMS (ESI) for $C_{18}H_{18}NO_5S$ [*M*+H]$^+$: calc'd: 360.0906; found: 360.0903.

2-(3-(2,2,2-Trifluoroethoxy)propyl)isoindoline-1,3-dione (39).

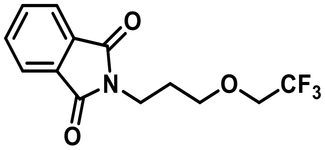

General procedure 7 with 3-(1,3-dioxoisoindolin-2-yl)propyl 4-methylbenzenesulfonate on a 1.0 mmol scale followed by flash chromatography (ethyl acetate/*n*-hexane, 1: 4) gave **39** as a colorless oil (64 mg, 22% yield). ^{1}H NMR (400 MHz, $CDCl_3$) *δ* (ppm): 7.84–7.72 (*m*, 2H), 7.70–7.54 (*m*, 2H), 3.72 (*dt*, *J* = 13.6, 7.7 Hz, 4H), 3.59 (*t*, *J* = 5.9 Hz, 2H), 1.92 (*quint*, *J* = 6.4 Hz, 2H); ^{19}F NMR (376 MHz, $CDCl_3$) *δ* (ppm): –74.16 (*t*, *J* = 8.6 Hz, 3F); ^{13}C NMR (101 MHz, $CDCl_3$) *δ* (ppm): 168.59, 134.12, 132.31, 124.11 (*q*, *J* = 279.6 Hz), 123.38, 70.51, 68.68 (*q*, *J* = 34.0 Hz), 35.34, 28.85. MS (ESI): 288.1 [*M*+H]$^+$. HRMS (ESI) for $C_{13}H_{13}NO_3F_3$ [*M*+H]$^+$: calc'd: 288.0848; found: 288.0848.

2-(2-(Benzyloxy)ethoxy)ethyl 4-methylbenzenesulfonate.

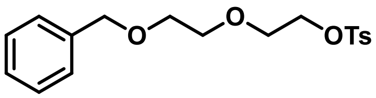

General procedure 6 with 2-[2-(benzyloxy)ethoxy]ethanol on a 5.0 mmol scale followed by flash chromatography (ethyl acetate/*n*-hexane, 1: 3) gave the title product as a colorless oil (1.61 g, 92% yield). ^{1}H NMR (400 MHz, $CDCl_3$) *δ* (ppm): 7.79 (*d*, *J* = 8.3 Hz, 2H), 7.50–7.00 (*m*, 7H), 4.52 (*s*, 2H), 4.27–4.04 (*m*, 2H), 3.84–3.64 (*m*, 2H), 3.63–3.41 (*m*, 2H), 2.41 (*s*, 3H); ^{13}C NMR (101 MHz, $CDCl_3$) *δ* (ppm): 144.92, 138.28, 133.13, 129.95, 128.53, 128.10, 127.85, 127.78, 73.40, 70.94, 69.50, 69.42, 68.82, 21.76. MS (ESI): 351.1 [*M*+H]$^+$. HRMS (ESI) for $C_{18}H_{23}O_5S$ [*M*+H]$^+$: calc'd: 351.1266; found: 351.1266.

((2-(2-(2,2,2-Trifluoroethoxy)ethoxy)ethoxy)methyl)benzene (40).

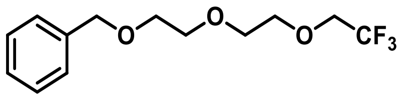

General procedure 7 with 2-(2-(benzyloxy)ethoxy)ethyl 4-methylbenzenesulfonate on a 1.0 mmol scale followed by flash chromatography (ethyl acetate-*n*-hexane, 1: 4) gave **40** as a colorless oil (234 mg, 84% yield). ^{1}H NMR (400 MHz, $CDCl_3$) δ (ppm): 7.75–6.75 (*m*, 5H), 4.56 (*s*, 2H), 3.90 (*q*, J = 8.8 Hz, 2H), 3.83–3.73 (*m*, 2H), 3.73–3.57 (*m*, 6H); ^{19}F NMR (376 MHz, $CDCl_3$) δ (ppm): –74.30 (*t*, J = 8.8 Hz, 3F); ^{13}C NMR (101 MHz, $CDCl_3$) δ (ppm): 138.39, 128.54, 127.89, 127.80, 124.23 (*q*, J = 279.6 Hz), 73.41, 72.13, 70.95, 70.87, 69.57, 68.94 (*q*, J = 33.9 Hz). MS (ESI): 279.1 $[M+H]^+$. HRMS (ESI) for $C_{13}H_{18}O_3F_3$ $[M+H]^+$: calc'd: 279.1208; found: 279.1207.

7,8-Dimethoxy-3-(3-(2,2,2-trifluoroethoxy)propyl)-1*H*-benzo[*d*]azepin-2(3*H*)-one (41).

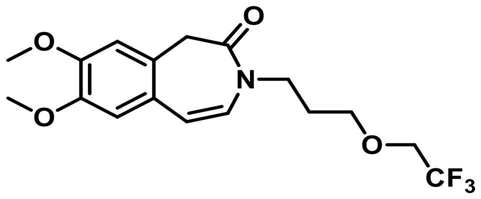

To a round-bottomed flask equipped with a magnetic stirrer bar was added dry NaH (53 mg, 2.0 mmol, 2.0 equiv.) and DMF (4.0 mL, 0.5 M) under argon, followed by 2,2,2-trifluoroethanol (200 mg, 2.0 mmol, 2 equiv. in 1 mL DMF) at 0 ^{o}C. The reaction mixture was stirred for 20 min under argon. 3-(3-Iodopropyl)-7,8-dimethoxy-1*H*-benzo[*d*]azepin-2(3*H*)-one (387 mg, 1.0 mmol, 1 equiv.) in DMF (2 mL) was then added dropwise at 0 ^{o}C. The reaction flask was capped loosely, and the contents were stirred vigorously at 0 ^{o}C for 1 h, warmed to 80 ^{o}C, and stirred for another 12 h. The reaction mixture was then poured onto water and extracted with DCM (2 $\times$15 mL). The organic phase was dried (Mg_2SO_4) and concentrated *in vacuo*. The residue was purified by silica gel flash chromatography (ethyl acetate/*n*-hexane, 1: 4) to give **41** as a colorless oil (172 mg, 48% yield). ^{1}H NMR (400 MHz, $CDCl_3$) δ (ppm): 6.77 (*s*, 1H), 6.72 (s, 1H), 6.33 (*d*, J = 9.1 Hz, 1H), 6.20 (*d*, J = 9.1 Hz, 1H), 3.88 (*s*, 3H), 3.87 (*s*, 3H), 3.72 (*q*, J = 8.8 Hz, 2H), 3.65 (*t*, J = 6.8 Hz, 2H), 3.47 (*t*, J = 6.0 Hz, 2H), 3.42 (*s*, 2H), 1.81 (*quint*, J = 6.4 Hz, 2H); ^{19}F NMR (376 MHz, $CDCl_3$) δ (ppm): –74.13 (*t*, J = 8.8 Hz, 3F); ^{13}C NMR (101 MHz, $CDCl_3$) δ (ppm): 167.86, 150.05, 148.22, 128.89, 126.51, 124.92, 124.13 (*q*, J = 279.6 Hz), 117.08, 111.35, 109.60, 69.75, 68.45 (*q*, J = 33.9 Hz), 56.13, 45.34, 43.37, 28.67. MS (ESI): 360.2 $[M+H]^+$. HRMS (ESI) for $C_{17}H_{21}NO_4F_3$ $[M+H]^+$: calc'd: 360.1423; found: 360.1425.

4-Methyl-2-((2,2,2-trifluoroethoxy)methyl)quinazoline (42).

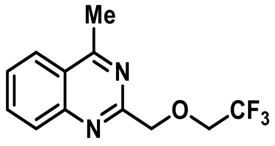

To a round-bottomed flask equipped with a magnetic stirrer bar was added dry NaH (53 mg, 2.0 mmol, 2.0 equiv.) and DMF (4.0 mL, 0.5 M) under argon, followed by 2,2,2-trifluoroethanol (200 mg, 2.0 mmol, 2.0 equiv.) in DMF (1.0 mL) at 0 °C. The reaction mixture was stirred for 20 min under argon. 2-(Chloromethyl)-4-methylquinazoline (193 mg, 1.0 mmol, 1.0 equiv.) in DMF (2.0 mL) was then added dropwise at 0 °C. The flask was capped loosely, and the contents stirred vigorously at 0 °C for 1 h and then 80 °C for 2 h. The reaction mixture was then poured onto water and extracted with DCM (2 × 15 mL). The organic phase was dried (Mg_2SO_4) and concentrated *in vacuo*. The residue was purified by flash chromatography (ethyl acetate-*n*-hexane, 1: 2) to give **42** as a white solid (238 mg, 93% yield). Mp: 75–77 °C. ^1H NMR (400 MHz, $CDCl_3$) δ (ppm): 8.14–7.92 (m, 2H), 7.84 (*ddd*, *J* = 8.4, 7.0, 1.3 Hz, 1H), 7.59 (*ddd*, *J* = 8.2, 7.0, 1.1 Hz, 2H), 4.98 (*s*, 2H), 4.13 (*q*, *J* = 8.8 Hz, 2H), 2.91 (*s*, 3H); ^{19}F NMR (376 MHz, $CDCl_3$) δ (ppm): –73.89 (*t*, *J* = 8.7 Hz, 3F); ^{13}C NMR (101 MHz, $CDCl_3$) δ (ppm): 169.13, 161.19, 149.78, 134.01, 129.06, 127.72, 125.12, 124.25 (*q*, *J* = 279.7 Hz), 123.35, 75.22, 68.58 (*q*, *J* = 34.0 Hz), 21.88. MS (ESI): 257.1 $[M+H]^+$. HRMS (ESI) for $C_{12}H_{12}N_2OF_3$ $[M+H]^+$: calc'd: 257.0902; found: 257.0901.

2-(4-Chloro-2-methylphenoxy)ethan-1-ol.

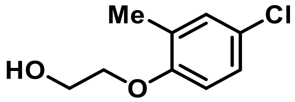

2-Methyl-4-chlorophenoxyacetic acid (2.01 g, 10 mmol, 1 equiv.) was dissolved in THF (20 mL) and $BH_3 \cdot Me_2S$ (1.52 g, 20 mmol, 2 equiv.) was added dropwise at 0 °C under argon. The mixture was stirred at RT for 12 h, quenched with methanol, washed with saturated aq. NH_4Cl, and extracted with ethyl acetate (2 × 20 mL). The organic phase was dried (Mg_2SO_4), filtered, and concentrated under reduced pressure. The residue was purified by silica gel chromatography (ethyl acetate-*n*-hexane, 1: 2) to give the title compound as a white solid (1.47 g, 79% yield). Mp: 56–58 °C. ^1H NMR (400 MHz, $CDCl_3$) δ (ppm): 7.11–7.02 (*m*, 2H), 6.72 (*d*, *J* = 8.4 Hz, 1H), 4.18–4.00 (*m*, 2H), 3.98 (*d*, *J* = 8.1 Hz, 2H), 2.20 (*s*, 4H); ^{13}C NMR (101 MHz, $CDCl_3$) δ (ppm): 155.50, 130.70, 128.88, 126.59, 125.68, 112.54, 69.86, 61.67, 16.28.

2-(4-Chloro-2-methylphenoxy)ethyl 4-methylbenzenesulfonate

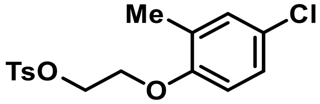

General procedure 6 with 2-(4-chloro-2-'methylphenoxy)ethan-1-ol on a 5.0 mmol followed by flash chromatography (ethyl acetate/*n*-hexane, 1: 3) gave the title compound as a colorless oil (1.56 g, 92% yield). Mp: 58–60 °C. ^1H NMR (400 MHz, $CDCl_3$) δ (ppm): 7.80 (*d*, *J* = 8.3 Hz, 2H), 7.32 (*d*, *J* =

8.0 Hz, 2H), 7.15–6.92 (*m*, 2H), 6.60 (*d*, *J* = 8.5 Hz, 1H), 4.50–4.27 (*m*, 2H), 4.25–3.95 (*m*, 2H), 2.44 (*s*, 3H), 2.09 (*s*, 3H); ^{13}C NMR (101 MHz, $CDCl_3$) *δ* (ppm): 154.93, 145.22, 133.03, 130.78, 130.08, 129.19, 128.10, 126.50, 126.01, 112.39, 68.26, 65.98, 21.84, 16.13. MS (ESI): 358.1 $[M+NH_4]^+$. HRMS (ESI) for $C_{16}H_{21}NO_4SCl$ $[M+NH_4]^+$: calc'd: 358.0880; found: 358.0880.

4-Chloro-2-methyl-1-(2-(2,2,2-trifluoroethoxy)ethoxy)benzene (43).

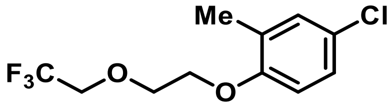

General procedure 7 with 2-(4-chloro-2-methylphenoxy)ethyl 4-methylbenzenesulfonate on a 1.0 mmol scale followed by flash chromatography (ethyl acetate/*n*-hexane, 1: 10) gave **43** as a colorless oil (183 mg, 68% yield). ^{1}H NMR (400 MHz, $CDCl_3$) *δ* (ppm): 7.11 (*dt*, *J* = 8.6, 2.3 Hz, 2H), 6.72 (*d*, *J* = 8.5 Hz, 1H), 4.12 (*dd*, *J* = 5.5, 3.7 Hz, 2H), 4.05–3.83 (*m*, 4H), 2.21 (*s*, 3H); ^{19}F NMR (376 MHz, $CDCl_3$) *δ* (ppm): –74.40 (*t*, *J* = 8.8 Hz, 3F); ^{13}C NMR (101 MHz, $CDCl_3$) *δ* (ppm): 155.52, 130.77, 129.08, 126.59, 125.79, 124.14 (*q*, *J* = 279.7 Hz), 112.46, 71.29, 69.19 (*q*, *J* = 33.9 Hz), 68.18, 16.27 .

3-(Benzo[*d*][1,3]dioxol-5-yl)-2-methylpropan-1-ol.

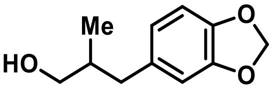

Helional (2-methyl-3-(3,4-methylenedioxyphenyl)propionaldehyde; 1.92 g, 10 mmol, 1 equiv.) was added over a period of 2 min at 0 ^{o}C to a suspension of $NaBH_4$ (0.76 g, 20 mmol, 2 equiv.) in ethanol (200 proof, 20 mL), The reaction mixture was stirred for 6 h at RT, quenched with ice–cold aq. HCl (1 M), and extracted with DCM (2 × 15 mL). The organic phase was dried (Mg_2SO_4) and then concentrated *in vacuo*. The residue was purified by silica gel flash chromatography (ethyl acetate/*n*-hexane, 1: 4) to give the product as a colorless oil (1.73 g, 89% yield). ^{1}H NMR (400 MHz, $CDCl_3$) *δ* (ppm): 6.72 (*d*, *J* = 7.9 Hz, 1H), 6.67 (*d*, *J* = 1.6 Hz, 1H), 6.61 (*dd*, *J* = 7.9, 1.6 Hz, 1H), 5.91 (*s*, 2H), 3.48 (*qd*, *J* = 10.6, 5.9 Hz, 1H), 2.67 (*dd*, *J* = 13.6, 6.4 Hz, 1H), 2.34 (*dd*, *J* = 13.6, 8.0 Hz, 1H), 1.97–1.77 (*m*, 1H), 1.56 (*s*, 1H), 0.90 (*d*, *J* = 6.8 Hz, 3H); ^{13}C NMR (101 MHz, $CDCl_3$) *δ* (ppm): 147.68, 145.83, 134.60, 122.07, 109.64, 108.21, 100.93, 67.69, 39.57, 38.07, 16.58.

3-(Benzo[*d*][1,3]dioxol-5-yl)-2-methylpropyl 4-methylbenzenesulfonate.

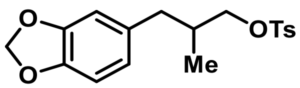

General procedure 6 with 3-(benzo[*d*][1,3]dioxol-5-yl)-2-methylpropan-1-ol on a 5.0 mmol followed by flash chromatography (ethyl acetate/*n*-hexane, 1: 4) gave the title compound as a colorless oil

(1.38 g, 79% yield). ^{1}H NMR (400 MHz, $CDCl_3$) δ (ppm): 7.78 (*d*, *J* = 8.3 Hz, 2H), 7.34 (*d*, *J* = 8.1 Hz, 2H), 6.66 (*d*, *J* = 7.8 Hz, 1H), 6.59–6.38 (*m*, 2H), 5.90 (s, 2H), 3.95–3.72 (*m*, 2H), 2.58 (*dd*, *J* = 13.7, 6.7 Hz, 1H), 2.45 (*s*, 3H), 2.33 (*dd*, *J* = 13.7, 7.7 Hz, 1H), 2.00 (*td*, *J* = 13.0, 6.7 Hz, 1H), 0.88 (*d*, *J* = 6.8 Hz, 3H); ^{13}C NMR (101 MHz, $CDCl_3$) δ (ppm): 147.74, 146.07, 144.93, 133.23, 133.12, 130.03, 128.10, 122.14, 109.52, 108.26, 101.00, 74.20, 38.85, 35.18, 21.83, 16.39. MS (ESI): 371.1 [*M*+Na]$^+$. HRMS (ESI) for $C_{18}H_{20}O_5SNa$ [*M*+Na]$^+$: calc'd: 371.0929; found: 371.0934.

5-(2-Methyl-3-(2,2,2-trifluoroethoxy)propyl)benzo[*d*][1,3]dioxole (44).

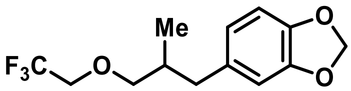

To a round-bottomed flask equipped with a magnetic stirrer bar was added dry NaH (53 mg, 2.0 mmol, 2 equiv.) and DMF (4 mL, 0.5 M) under argon followed by adding 2,2,2-trifluoroethanol (200 mg, 2.0 mmol, 2 equiv.) in DMF (1.0 mL) at 0 °C. The reaction mixture was stirred for 20 min under argon. 3-(Benzo[*d*][1,3]dioxol-5-yl)-2-methylpropyl 4-methylbenzenesulfonate (348 mg, 1.0 mmol, 1 equiv.) in DMF (2 mL) was added dropwise at 0 °C, The flask was loosely capped, stirred vigorously at 0 °C for 1 h, and heated at 80 °C for 12 h. The reaction mixture was then poured onto water and extracted with DCM (2 × 15 mL). The organic phase was dried (Mg_2SO_4) and concentrated *in vacuo*. Silica gel flash chromatography (ethyl acetate/*n*-hexane, 1: 10) of the residue gave **44** as a colorless oil (173 mg, 63% yield). ^{1}H NMR (400 MHz, $CDCl_3$) δ (ppm): 6.73 (*d*, *J* = 7.9 Hz, 1H), 6.65 (*d*, *J* = 1.6 Hz, 1H), 6.60 (*dd*, *J* = 7.9, 1.6 Hz, 1H), 5.92 (*s*, 2H), 3.79 (*q*, *J* = 8.8 Hz, 1H), 3.53–3.30 (m, 2H), 2.67 (*dd*, *J* = 13.6, 6.4 Hz, 1H), 2.37 (*dd*, *J* = 13.6, 7.8 Hz, 1H), 1.99 (*dq*, *J* = 12.9, 6.4 Hz, 1H), 0.92 (*d*, *J* = 6.8 Hz, 3H); ^{19}F NMR (376 MHz, $CDCl_3$) δ (ppm): –74.12 (*d*, *J* = 8.7 Hz, 3F); ^{13}C NMR (101 MHz, $CDCl_3$) δ (ppm): 147.72, 145.92, 134.23, 124.31 (*q*, *J* = 279.7 Hz), 122.20, 109.69, 108.23, 100.97, 77.19, 68.74 (*q*, *J* = 33.8 Hz), 39.37, 35.81, 16.72.

1-(((2*R*,4*R*)-2-(2,4-Difluorophenyl)-4-((2,2,2-trifluoroethoxy)methyl)tetrahydrofuran-2-yl)methyl)-1*H*-1,2,4-triazole (45).

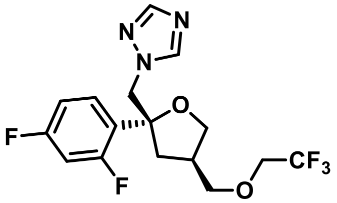

General procedure 7 with ((3*S*,5*R*)-5-((1*H*-1,2,4-triazol-1-yl)methyl)-5-(2,4-difluorophenyl)tetrahydrofuran-3-yl)methyl 4-methylbenzenesulfonate on a 0.5 mmol scale followed by flash chromatography (ethyl acetate/DCM: 3: 1) gave **45** as a colorless oil (87 mg, 46% yield). ^{1}H NMR (400 MHz, $CDCl_3$) δ (ppm): 8.07 (*s*, 1H), 7.79

(*s*, 1H), 7.33 (*td*, *J* = 8.7, 6.6 Hz, 1H), 6.81 (*tdd*, *J* = 11.1, 8.4, 2.5 Hz, 2H), 4.53 (*dd*, *J* = 46.3, 14.4 Hz, 2H), 4.20–3.88 (*m*, 1H), 3.69 (*ddd*, *J* = 15.2, 13.0, 7.5 Hz, 3H), 3.40 (*dd*, *J* = 8.9, 5.3 Hz, 1H), 3.33–3.21 (*m*, 1H), 2.67–2.21 (*m*, 2H), 1.93 (*td*, *J* = 11.6, 4.8 Hz, 1H); ^{19}F NMR (376 MHz, $CDCl_3$) *δ* (ppm): –74.14 (*t*, *J* = 8.4 Hz, 3F), –109.02 (*dd*, *J* = 18.7, 9.6 Hz, 1F), –109.93 – –111.27 (*m*, 1F); ^{13}C NMR (101 MHz, $CDCl_3$) *δ* (ppm): 162.97 (*dd*, *J* = 249.6, 11.9 Hz), 159.16 (*dd*, *J* = 247.5, 11.9 Hz), 151.26, 144.74, 128.75 (*dd*, *J* = 9.5, 5.5 Hz), 125.47 (*dd*, *J* = 13.1, 3.7 Hz), 123.99 (*q*, *J* = 279.7 Hz), 111.49 (*dd*, *J* = 20.7, 3.5 Hz), 104.79 (*t*, *J* = 26.0 Hz), 84.15 (*d*, *J* = 4.3 Hz), 73.60, 70.72, 68.68 (*q*, *J* = 33.9 Hz), 56.05 (*d*, *J* = 4.1 Hz), 39.16, 37.40 (*d*, *J* = 3.5 Hz). MS (ESI): 378.1 [*M*+H]$^+$. HRMS (ESI) for $C_{16}H_{17}N_3O_2F_5$ [*M*+H]$^+$: calc'd: 378.1241; found: 378.1238.

1-(((2*R*,4*R*)-2-(2,4-Dichlorophenyl)-4-((2,2,2-trifluoroethoxy)methyl)-1,3-dioxolan-2-yl)methyl)-1*H*-imidazole (46).

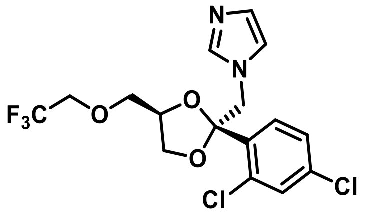

General procedure 7 with ((2*R*,4*S*)-2-((1*H*-imidazol-1-yl)methyl)-2-(2,4-dichlorophenyl)-1,3-dioxolan-4-yl)methyl 4-methylbenzenesulfonate on a 1.0 mmol scale followed by flash chromatography (ethyl acetate) gave **46** as a colorless oil (345 mg, 84% yield). ^{1}H NMR (400 MHz, $CDCl_3$) *δ* (ppm): 7.53 (*d*, *J* = 8.4 Hz, 1H), 7.47 (*s*, 1H), 7.43 (*d*, *J* = 2.0 Hz, 1H), 7.22 (*dd*, *J* = 8.4, 2.0 Hz, 1H), 6.98 (*s*, 1H), 6.94 (*s*, 1H), 4.41 (*dd*, *J* = 49.1, 14.8 Hz, 2H), 4.24–4.07 (*m*, 1H), 3.94–3.63 (*m*, 3H), 3.56 (*dd*, *J* = 8.4, 5.2 Hz, 1H), 3.27 (*dd*, *J* = 10.0, 6.0 Hz, 1H), 3.17 (*dd*, *J* = 10.0, 5.5 Hz, 1H); ^{19}F NMR (376 MHz, $CDCl_3$) *δ* (ppm): –74.22 (*t*, *J* = 8.4 Hz, 3F); ^{13}C NMR (101 MHz, $CDCl_3$) *δ* (ppm): 138.91, 136.08, 134.62, 133.09, 131.53, 129.71, 128.67, 127.40, 123.91 (*q*, *J* = 279.6 Hz), 121.25, 108.27, 75.19, 72.32, 68.81 (*q*, *J* = 34.3 Hz), 67.14, 51.33. MS (ESI): 411.1 [*M*+H]$^+$. HRMS (ESI) for $C_{16}H_{16}N_2O_3F_3Cl_2$ [*M*+H]$^+$: calc'd: 411.0490; found: 411.0494.

(3-(5-(2-Methoxyphenyl)-1,2,4-oxadiazol-3-yl)phenyl)methanol.

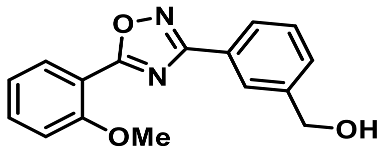

To a round-bottomed flask equipped with a magnetic stirrer bar was added dry NaH (267 mg, 10.0 mmol, 2 equiv.) and DMF (20 mL) under argon, followed by addition of MeOH (2 mL) at 0 °C. The reaction mixture was stirred for 20 min under argon. (3-(5-(2-Fluorophenyl)-1,2,4-oxadiazol-3-yl)phenyl)methanol (1.35 g, 5.0 mmol, 1.0 equiv.) in DMF (3 mL) was then added dropwise at RT. The flask was loosely capped, and the contents stirred vigorously at 80 °C for 12 h. The reaction mixture was then poured onto

water and extracted with DCM (2 × 15 mL). The organic phase was dried (Mg_2SO_4) and concentrated *in vacuo*. The residue was purified by silica gel flash chromatography (ethyl acetate/*n*-hexane, 1: 2) to give the title compound as a white solid (901 mg, 64% yield). Mp: 98–100 °C. ^{1}H NMR (400 MHz, $CDCl_3$) δ (ppm): 8.37–7.96 (*m*, 3H), 7.72–7.38 (*m*, 3H), 7.22–6.86 (*m*, 2H), 4.78 (*d*, J = 5.9 Hz, 2H), 4.00 (*s*, 3H), 2.06 (*t*, J = 6.0 Hz, 1H); ^{13}C NMR (101 MHz, $CDCl_3$) δ (ppm): 175.36, 168.42, 158.73, 141.85, 134.34, 131.86, 129.71, 129.26, 127.54, 126.94, 126.20, 120.98, 113.65, 112.32, 65.13, 56.27. MS (ESI): 283.1 $[M+H]^+$. HRMS (ESI) for $C_{16}H_{15}N_2O_3$ $[M+H]^+$: calc'd: 283.1083; found: 283.1078. .

3-(3-(Bromomethyl)phenyl)-5-(2-methoxyphenyl)-1,2,4-oxadiazole.

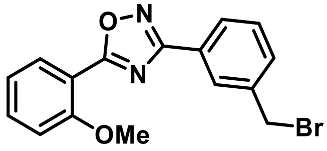

(3-(5-(2-Methoxyphenyl)-1,2,4-oxadiazol-3-yl)phenyl)methanol (0.85 g, 3.0 mmol, 1 equiv.) was dissolved in anhydrous DCM (20 mL) and PBr_3 (0.97 g, 3.6 mmol, 1.2 equiv.) was added dropwise at 0 °C. The mixture was stirred at RT for 2 h, quenched with saturated aq. $NaHCO_3$ solution, and extracted with DCM (2 × 15 mL). The organic layer was separated off, dried (Mg_2SO_4), filtered, and concentrated under reduced pressure. The residue was purified by silica gel chromatography (ethyl acetate/*n*-hexane, 1/5) to give the title compound as a white solid (0.59 g, 57% yield). Mp: 137–139 °C. ^{1}H NMR (400 MHz, $CDCl_3$) δ (ppm): 8.22 (*d*, J = 1.6 Hz, 1H), 8.19–8.05 (*m*, 2H), 7.69–7.52 (*m*, 2H), 7.49 (*t*, J = 7.7 Hz, 1H), 7.18–7.01 (*m*, 2H), 4.57 (*s*, 2H), 4.01 (*s*, 3H); ^{13}C NMR (101 MHz, $CDCl_3$) δ (ppm): 175.49, 168.08, 158.77, 138.74, 134.40, 131.89, 131.85, 129.58, 128.31, 127.97, 127.70, 121.01, 113.64, 112.35, 56.30, 32.93. MS (ESI): 345.0 $[M+H]^+$. HRMS (ESI) for $C_{16}H_{14}N_2O_2{}^{79}Br$ $[M+H]^+$: calc'd: 345.0239; found: 345.0242.

5-(2-Methoxyphenyl)-3-(3-((2,2,2-trifluoroethoxy)methyl)phenyl)-1,2,4-oxadiazole (47).

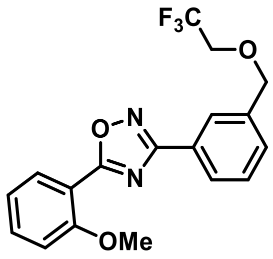

To a round-bottomed flask equipped with a magnetic stirrer bar was added dry NaH (27 mg, 1.0 mmol, 2 equiv.) and DMF (1 mL, 0.5 M) under argon, followed by 2,2,2-trifluoroethanol (100 mg, 1.0 mmol, 2.0 equiv.) in DMF (1 mL) at 0 °C. The reaction mixture was stirred for 20 min under argon. 3-(3-(Bromomethyl)phenyl)-5-(2-methoxyphenyl)-1,2,4-oxadiazole (173 mg, 0.5 mmol, 1 equiv.) in DMF (2 mL) was then added dropwise at RT. The flask was capped loosely capped, and the contents stirred vigorously at 80 °C for 2 h. The reaction mixture was then poured onto water and

extracted with DCM (2 × 15 mL). The organic phase was separated off, dried (Mg_2SO_4), filtered, and concentrated *in vacuo*. The residue was purified by silica gel flash chromatography (ethyl acetate/*n*-hexane, 1: 5) to give **47** as a white solid (147 mg, 81% yield). Mp: 66–68 °C. ^{1}H NMR (400 MHz, $CDCl_3$) *δ* (ppm): 8.34–7.90 (*m*, 3H), 7.90–7.43 (*m*, 3H), 7.20–6.74 (*m*, 2H), 4.75 (*s*, 2H), 3.99 (*s*, 3H), 3.88 (*q*, *J* = 8.7 Hz, 2H); ^{19}F NMR (376 MHz, $CDCl_3$) *δ* (ppm): –73.82 (*t*, *J* = 8.8 Hz, 3F); ^{13}C NMR (101 MHz, $CDCl_3$) *δ* (ppm): 175.42, 168.21, 158.70, 137.50, 134.33, 131.79, 130.46, 129.35, 127.70, 127.54, 127.01, 124.21 (q, *J* = 279.3 Hz), 120.92, 113.56, 112.28, 73.86, 67.54 (*q*, *J* = 34.2 Hz), 56.17. MS (ESI): 365.1 [*M*+H]$^+$. HRMS (ESI) for $C_{18}H_{16}N_2O_3F_3$ [*M*+H]$^+$: calc'd: 365.1113; found: 365.1117.

2-Cyclopropyl-4-(4-fluorophenyl)-3-((2,2,2-trifluoroethoxy)methyl)quinoline (48).

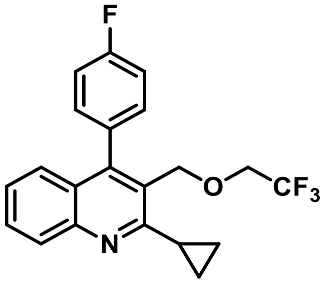

To a round-bottomed flask equipped with a magnetic stirrer bar was added dry NaH (53 mg, 2.0 mmol, 2 equiv.) and DMF (4 mL, 0.5 M) under argon, followed by 2,2,2-trifluoroethanol (200 mg, 2.0 mmol, 2 equiv.) in DMF (1 mL) at 0 °C. The reaction mixture was stirred for 20 min under argon. 3-(Bromomethyl)-2-cyclopropyl-4-(4-fluorophenyl)quinoline (356 mg, 1.0 mmol, 1 equiv.) in DMF (2 mL) was then added dropwise at RT. The flask was capped loosely and stirred vigorously at 80 °C for 2 h. The reaction mixture was then poured onto water and extracted with DCM (2 x 15 mL). The organic phase was separated, dried (Mg_2SO_4), filtered, and concentrated *in vacuo*. Flash chromatography (ethyl acetate/*n*-hexane, 1: 4) of the residue gave **48** as a colorless oil (278 mg, 74% yield). ^{1}H NMR (400 MHz, $CDCl_3$) *δ* (ppm): 7.97 (*d*, *J* = 8.4 Hz, 1H), 7.61 (*ddd*, *J* = 8.4, 5.6, 2.6 Hz, 1H), 7.38–7.26 (*m*, 4H), 7.22 (*dt*, *J* = 8.7, 2.4 Hz, 2H), 4.67 (*s*, 2H), 3.75 (*q*, *J* = 8.7 Hz, 2H), 2.48 (*tt*, *J* = 8.1, 4.9 Hz, 1H), 1.46–1.26 (*m*, 2H), 1.19– 0.89 (*m*, 2H); ^{19}F NMR (376 MHz, $CDCl_3$) *δ* (ppm): –73.69 (*t*, *J* = 8.8 Hz, 3F), –105.26 – –117.40 (*m*, 1F); ^{13}C NMR (101 MHz, $CDCl_3$) *δ* (ppm): 162.90 (*d*, *J* = 247.7 Hz), 162.62, 147.90, 132.29 (*d*, *J* = 3.5 Hz), 131.67 (*d*, *J* = 8.0 Hz), 129.68, 129.21, 126.66, 126.12, 125.93, 125.78, 124.11 (*q*, *J* = 279.7 Hz), 115.61 (*d*, *J* = 21.5 Hz), 69.09, 67.94 (*q*, *J* = 34.2 Hz), 14.73, 9.90. MS (ESI): 376.1 [*M*+H]$^+$. HRMS (ESI) for $C_{21}H_{18}NOF_4$ [*M*+H]$^+$: calc'd: 376.1325; found: 376.1328.

2-(1-Benzyl-1*H*-indazol-3-yloxy)ethanol.

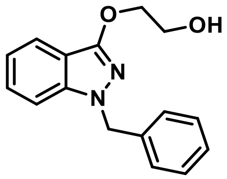

Bendazac (2-((1-benzyl-1*H*-indazol-3-yl)oxy)acetic acid; 2.82 g, 10 mmol, 1 equiv.) was dissolved in THF (20 mL) and $BH_3·Me_2S$ (1.52 g, 20 mmol, 2 equiv.) was added dropwise at 0 °C under argon. The mixture was stirred at RT for 12 h and then quenched with MeOH, washed with saturated aq. NH_4Cl, and extracted with ethyl acetate (2 × 20 mL). The organic layer was dried (Mg_2SO_4), filtered, and concentrated under reduced pressure. The residue was purified with silica gel chromatography (ethyl acetate/*n*-hexane, 1/3) to give the title compound as a white solid (2.34 g, 87% yield). Mp: 89–91 °C. ^{1}H NMR (400 MHz, $CDCl_3$) *δ* (ppm): 7.58 (*d*, *J* = 8.1 Hz, 1H), 7.29–7.13 (*m*, 4H), 7.09 (*t*, *J* = 7.2 Hz, 3H), 6.96 (*t*, *J* = 7.5 Hz, 1H), 5.28 (*s*, 2H), 4.66–4.28 (*m*, 2H), 3.92 (*d*, *J* = 3.1 Hz, 2H), 3.17 (*t*, *J* = 5.3 Hz, 1H); ^{13}C NMR (101 MHz, $CDCl_3$) *δ* (ppm): 156.13, 141.84, 137.31, 128.83, 127.89, 127.81, 127.30, 120.30, 119.61, 113.00, 109.14, 71.59, 62.49, 52.55. MS (ESI): 269.1 [*M*+H]$^+$. HRMS (ESI) for $C_{16}H_{17}N_2O_2$ [*M*+H]$^+$: calc'd: 269.1290; found: 269.1290.

2-(1-Benzyl-1*H*-indazol-3-yloxy)ethyl 4-methylbenzenesulfonate.

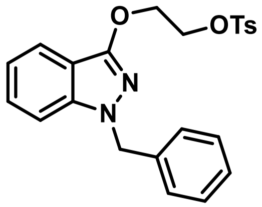

General procedure 6 with 2-(1-benzyl-1*H*-indazol-3-yloxy)ethanol on a 5.0 mmol followed by flash chromatography (ethyl acetate/*n*-hexane, 1: 3) gave the title compound as a white solid (1.75 g, 83% yield). Mp: 61–63 °C. ^{1}H NMR (400 MHz, $CDCl_3$) *δ* (ppm): 7.68 (*d*, *J* = 8.0 Hz, 2H), 7.44 (*d*, *J* = 8.1 Hz, 1H), 7.20 (*dt*, *J* = 17.2, 8.8 Hz, 4H), 7.07 (*dd*, *J* = 14.8, 7.2 Hz, 5H), 6.94 (*t*, *J* = 7.5 Hz, 1H), 5.23 (*s*, 2H), 4.59–4.44 (*m*, 2H), 4.42–4.30 (*m*, 2H), 2.23 (*s*, 3H); ^{13}C NMR (101 MHz, $CDCl_3$) *δ* (ppm): 155.06, 144.89, 141.70, 137.45, 133.02, 129.90, 128.79, 128.11, 127.76, 127.67, 127.23, 120.20, 119.46, 112.82, 108.99, 68.34, 66.17, 52.47, 21.73. MS (ESI): 423.1 [*M*+H]$^+$. HRMS (ESI) for $C_{23}H_{23}N_2O_4S$ [*M*+H]$^+$: calc'd: 423.1379; found: 423.1378.

1-Benzyl-3-(2-(2,2,2-trifluoroethoxy)ethoxy)-1*H*-indazole (49).

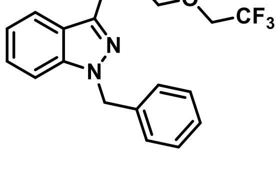

General procedure 7 with 2-(1-benzyl-1*H*-indazol-3-yloxy)ethyl 4-methylbenzenesulfonate on a 1.0 mmol scale followed by flash chromatography (ethyl acetate/*n*-hexane, 1: 4) gave **49** as a colorless oil (178 mg, 51% yield). ^{1}H NMR (400 MHz, $CDCl_3$) *δ* (ppm): 7.59 (*d*, *J* = 8.1 Hz, 1H), 7.40–7.12 (*m*, 4H), 7.08 (*t*, *J* = 7.3 Hz, 3H), 6.95 (*t*, *J* = 7.5 Hz, 1H), 5.29 (*s*, 2H), 4.48 (*dd*, *J* = 4.9, 3.8 Hz, 2H), 4.10–3.94 (*m*, 2H), 3.88 (*q*, *J* =

8.7 Hz, 2H); ^{19}F NMR (376 MHz, $CDCl_3$) δ (ppm): –74.21 (*t*, *J* = 8.8 Hz, 3F); ^{13}C NMR (101 MHz, $CDCl_3$) δ (ppm): 155.72, 141.85, 137.58, 128.79, 127.73, 127.69, 127.19, 124.22 (*q*, *J* = 279.7 Hz), 120.26, 119.49, 113.08, 109.07, 71.22, 69.03 (*q*, *J* = 33.9 Hz), 68.36, 52.52. MS (ESI): 351.1 $[M+H]^+$. HRMS (ESI) for $C_{18}H_{18}N_2O_2F_3$ $[M+H]^+$: calc'd: 351.1320; found: 351.1323.

((2*R*,3*R*,4*S*,5*R*,6*S*)-3,4,5,6-tetrakis(benzyloxy)tetrahydro-2*H*-pyran-2-yl)methyl 4-methylbenzenesulfonate.

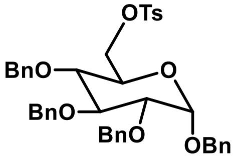

General procedure 6 with ((2*R*,3*R*,4*S*,5*R*,6*S*)-3,4,5,6-tetrakis(benzyloxy)tetrahydro-2*H*-pyran-2-yl)methanol on a 4.0 mmol scale followed by flash chromatography (ethyl acetate/*n*-hexane, 1: 3) gave the title compound as a colorless oil (2.34 g, 84% yield). ^{1}H NMR (400 MHz, $CDCl_3$) δ (ppm): 7.68 (*d*, *J* = 8.3 Hz, 2H), 7.37–7.13 (m, 20H), 7.07 (*dd, J* = 7.0, 2.4 Hz, 2H), 4.91 (*d*, *J* = 10.9 Hz, 1H), 4.84–4.61 (*m*, 3H), 4.54 (*dd*, *J* = 13.8, 12.2 Hz, 2H), 4.48–4.29 (*m*, 3H), 4.11 (*dd*, *J* = 10.6, 4.2 Hz, 1H), 4.06–3.86 (*m*, 2H), 3.71 (*ddd*, *J* = 10.1, 4.1, 1.9 Hz, 1H), 3.46–3.28 (*m*, 2H), 2.31 (*s*, 3H); ^{13}C NMR (101 MHz, $CDCl_3$) δ (ppm): 145.00, 138.82, 138.17, 137.95, 137.10, 133.11, 129.98, 128.63, 128.60, 128.58, 128.19, 128.17, 128.13, 128.10, 128.05, 128.03, 127.82, 95.67, 82.03, 79.93, 77.15, 75.86, 75.23, 73.22, 69.59, 68.97, 68.70, 21.81. MS (ESI): 712.3 $[M+NH_4]^+$. HRMS (ESI) for $C_{41}H_{46}NO_8S$ $[M+NH_4]^+$: calc'd: 712.2944; found: 712.2946.

(2*S*,3*R*,4*S*,5*R*,6*R*)-2,3,4,5-tetrakis(benzyloxy)-6-((2,2,2-trifluoroethoxy)methyl)tetrahydro-2*H*-pyran (50).

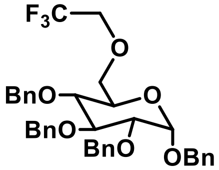

General procedure 7 with ((2*R*,3*R*,4*S*,5*R*,6*S*)-3,4,5,6-tetrakis(benzyloxy)tetrahydro-2*H*-pyran-2-yl)methyl 4-methylbenzenesulfonate on a 0.5 mmol scale followed by flash chromatography (ethyl acetate/*n*-hexane, 1: 4) gave **50** as a white solid (240 mg, 77% yield). Mp: 85–87 ^{o}C. ^{1}H NMR (400 MHz, $CDCl_3$) δ (ppm): 7.55–7.27 (*m*, 20H), 5.08 (*d*, *J* = 10.8 Hz, 1H), 4.97 (*d*, *J* = 10.9 Hz, 1H), 4.90 (*dd*, *J* = 7.2, 3.6 Hz, 2H), 4.74 (*d*, *J* = 12.0 Hz, 2H), 4.64 (*dd*, *J* = 19.5, 11.4 Hz, 3H), 4.12 (*t*, *J* = 9.3 Hz, 1H), 4.02–3.73 (*m*, 4H), 3.75–3.53 (*m*, 3H); ^{19}F NMR (376 MHz, $CDCl_3$) δ (ppm): –73.87 (*t*, *J* = 8.7 Hz, 3F); ^{13}C NMR (101 MHz, $CDCl_3$) δ (ppm): 138.93, 138.38, 138.29, 137.25, 128.63, 128.57, 128.12, 128.09, 127.97, 127.77, 124.11 (*q*, *J* = 279.7 Hz), 95.81, 82.23, 80.14, 77.42,

75.91, 75.23, 73.23, 71.14, 70.62, 69.49, 69.08 (*q*, *J* = 34.1 Hz). MS (ESI): 640.3 [*M*+H]$^+$. HRMS (ESI) for $C_{36}H_{41}NO_6F_3$ [*M*+H]$^+$: calc'd: 640.2886; found: 640.2892.

5-(5-(Hydroxymethyl)-4-methylthiazol-2-yl)-2-isobutoxybenzonitrile

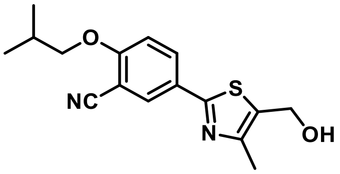

Febuxostat (2-(3-cyano-4-isobutoxyphenyl)-4-methylthiazole-5-carboxylic acid; 1.58 g, 5.0 mmol, 1 equiv.) was dissolved in THF (20 mL) and $BH_3{\cdot}Me_2S$ (0.76 g, 10 mmol, 2 equiv.) was added dropwise at 0 ^oC under argon. The mixture was stirred at RT for 12 h, quenched with MeOH, washed with saturated aq. NH_4Cl, and extracted with ethyl acetate (2 × 20 mL). The organic layer was dried (Mg_2SO_4), filtered, and concentrated under reduced pressure. The residue was purified by silica gel chromatography (ethyl acetate/*n*-hexane, 1: 1) to give the title compound as a white solid (1.15 g, 74% yield). Mp: 106–108 ^oC. ^1H NMR (400 MHz, $CDCl_3$) δ (ppm): 8.02 (*d*, *J* = 2.3 Hz, 1H), 7.95 (*dd*, *J* = 8.8, 2.3 Hz, 1H), 6.94 (*d*, *J* = 8.9 Hz, 1H), 4.78 (*s*, 2H), 3.84 (*d*, *J* = 6.5 Hz, 2H), 2.86 (*s*, 1H), 2.38 (*s*, 3H), 2.16 (*sept*, *J* = 13.3, 6.7 Hz, 1H), 1.06 (*d*, *J* = 6.7 Hz, 6H); ^{13}C NMR (101 MHz, $CDCl_3$) δ (ppm): 163.60, 161.89, 150.41, 132.23, 131.77, 131.56, 126.86, 115.87, 112.70, 102.72, 75.74, 56.79, 28.32, 19.22, 15.20. MS (ESI): 303.1 [*M*+H]$^+$. HRMS (ESI) for $C_{16}H_{19}N_2O_2S$ [*M*+H]$^+$: calc'd: 303.1167; found: 303.1172.

5-(5-(Bromomethyl)-4-methylthiazol-2-yl)-2-isobutoxybenzonitrile.

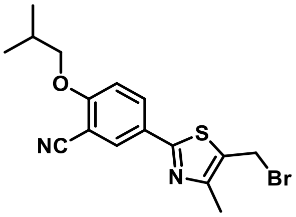

5-(5-(Hydroxymethyl)-4-methylthiazol-2-yl)-2-isobutoxybenzonitrile (0.91 g, 3.0 mmol, 1 equiv.) was dissolved in anhydrous DCM (20 mL) and PBr_3 (0.34 mL, 0.97 g, 3.6 mmol, 1.2 equiv.) was added dropwise at 0 ^oC. The mixture was stirred at RT for 2 h , quenched with saturated aq. $NaHCO_3$ solution, and extracted with DCM (2 × 15 mL). The organic layer was dried (Mg_2SO_4), filtered, and concentrated under reduced pressure. The residue was purified by flash chromatography (ethyl acetate/*n*-hexane, 1: 3) to give the title compound as a white solid (0.73 g, 67% yield). Mp: 96–98 ^oC. ^1H NMR (400 MHz, $CDCl_3$) δ (ppm): 8.09 (*d*, *J* = 2.2 Hz, 1H), 8.01 (*dd*, *J* = 8.8, 2.3 Hz, 1H), 6.98 (*d*, *J* = 8.9 Hz, 1H), 4.70 (*s*, 2H), 3.88 (*d*, *J* = 6.5 Hz, 2H), 2.43 (*s*, 3H), 2.19 (*sept*, *J* = 13.3, 6.7 Hz, 1H), 1.08 (*d*, *J* = 6.7 Hz, 6H); ^{13}C NMR (101 MHz, $CDCl_3$) δ (ppm): 164.24, 162.16, 153.25, 132.29, 131.77, 128.39, 126.67, 115.75, 112.76, 103.01, 75.81, 28.35, 23.86, 19.26, 15.25. MS (ESI): 365.0 [*M*+H]$^+$. HRMS (ESI) for $C_{16}H_{18}N_2OS^{79}Br$ [*M*+H]$^+$: calc'd: 365.0323; found: 365.0329.

2-Isobutoxy-5-(4-methyl-5-((2,2,2-trifluoroethoxy)methyl)thiazol-2-yl)benzonitrile (51).

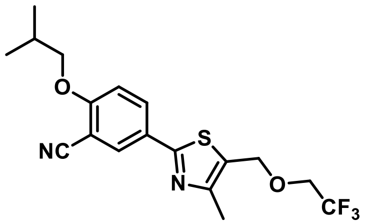

To a round-bottomed flask with a magnetic stirrer bar was added dry NaH (53 mg, 2.0 mmol, 2 equiv.) and DMF (2 mL, 0.5 M) under argon, followed by 2,2,2-trifluoroethanol (200 mg, 2.0 mmol, 2 equiv.) in DMF (1 mL) at 0 °C. The reaction mixture was stirred for 20 min under argon. 5-(5-(Bromomethyl)-4-methylthiazol-2-yl)-2-isobutoxybenzonitrile (183 mg, 1.0 mmol, 1 equiv.) in DMF (2 mL) was then added dropwise at R. The reaction was loosely capped and stirred vigorously at 80 °C for 2 h. The reaction mixture was poured onto water and extracted with DCM. The organic phase was dried (Mg_2SO_4), filtered, and concentrated *in vacuo*. Flash chromatography (ethyl acetate/*n*-hexane, 1: 3) of the residue gave **51** as a white solid (132 mg, 69% yield). Mp: 86–88 °C .^{1}H NMR (400 MHz, $CDCl_3$) *δ* (ppm): 8.10 (*d*, *J* = 2.3 Hz, 1H), 8.02 (*dd*, *J* = 8.8, 2.3 Hz, 1H), 6.98 (*d*, *J* = 8.9 Hz, 1H), 4.81 (*s*, 2H), 4.07–3.52 (*m*, 4H), 2.45 (*s*, 3H), 2.19 (dp, *J* = 13.3, 6.7 Hz, 1H), 1.08 (*d*, *J* = 6.7 Hz, 6H); ^{19}F NMR (376 MHz, $CDCl_3$) *δ* (ppm): –73.72 (*t*, *J* = 9.2 Hz, 3F); ^{13}C NMR (101 MHz, $CDCl_3$) *δ* (ppm): 164.69, 162.10, 152.74, 132.32, 131.78, 126.78, 126.54, 124.08 (*q*, *J* = 279.3 Hz), 115.80, 112.74, 102.97, 75.80, 67.02 (*q*, *J* = 34.3 Hz), 65.45, 28.36, 19.25, 15.36. MS (ESI): 385.1 $[M+H]^+$. HRMS (ESI) for $C_{18}H_{20}N_2O_2F_3S$ $[M+H]^+$: calc'd: 385.1198; found: 385.1203.

***N*-(2,6-Diethylphenyl)-*N*-(2-propoxyethyl)-2-(2,2,2-trifluoroethoxy)acetamide (52).**

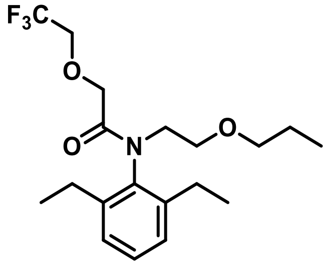

To a round-bottomed flask equipped with a magnetic stirrer bar was added Cs_2CO_3 (652 mg, 2.0 mmol, 2 equiv.) and DMF (4 mL) under argon. The reaction mixture was stirred for 5 min. Pretilachlor (2-chloro-*N*-(2,6-diethylphenyl)-*N*-(2-propoxyethyl)acetamide; 312 mg, 1.0 mmol, 1 equiv.) was then added under argon. The reaction mixture was stirred vigorously at RT for 12 h, poured onto water, and extracted with DCM (2 × 15 mL). The organic phase was dried (Mg_2SO_4), filtered, and concentrated *in vacuo*. The residue was purified by flash chromatography (ethyl acetate/*n*-hexane, 1: 10) to afford the product as a colorless oil (301 mg, 80% yield). ^{1}H NMR (400 MHz, $CDCl_3$) *δ* (ppm): 7.25 (*t, J* = 7.7 Hz, 1H), 7.13 (*d*, *J* = 7.7 Hz, 2H), 3.90 (*q*, *J* = 8.8 Hz, 2H), 3.74–3.64 (*m*, 4H), 3.53 (*t*, *J* = 6.1 Hz, 2H), 3.25 (*t*, *J* = 6.7 Hz, 2H), 2.64–2.30 (*m*, 4H), 1.50–1.36 (*m*, 2H), 1.18 (*t*, *J* = 7.5 Hz, 6H), 0.78 (*t*, *J* = 7.4 Hz, 3H);

^{19}F NMR (376 MHz, $CDCl_3$) δ (ppm): –74.33 (*t*, *J* = 8.8 Hz, 3F); ^{13}C NMR (101 MHz, $CDCl_3$) δ (ppm): 169.44, 141.54, 137.20, 129.26, 126.99, 124.01 (*q*, *J* = 279.6 Hz), 72.85, 69.53, 68.53 (*q*, *J* = 34.2 Hz), 67.52, 49.69, 23.37, 22.95, 14.28, 10.63. MS (ESI): 376.2 $[M+H]^+$. HRMS (ESI) for $C_{19}H_{29}NO_3F_3$ $[M+H]^+$: calc'd: 376.2100; found: 376.2107.

3-(4,5-Diphenyloxazol-2-yl)propan-1-ol.

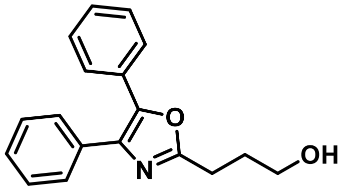

Oxaprozin (3-(4,5-diphenyloxazol-2-yl)propanoic acid; 2.93 g, 10 mmol, 1 equiv.) was dissolved in THF (20 mL) and $BH_3{\cdot}Me_2S$ (1.52 g, 20 mmol, 2 equiv.) was added dropwise at 0 °C under argon. The mixture was stirred at RT for 12 h, quenched with MeOH, and washed with saturated aq. NH_4Cl. The aqueous phase was further extracted with ethyl acetate (2 × 15 mL). The combined organic phase was dried (Mg_2SO_4), filtered and concentrated under reduced pressure. The residue was purified by flash chromatography (ethyl acetate/n-hexane, 1: 2) to give the title compound as a white solid (2.01 g, 72% yield). Mp: 98–100 °C. ^1H NMR (400 MHz, $CDCl_3$) δ (ppm): 7.60 (*dd*, *J* = 20.6, 7.1 Hz, 4H), 7.43–7.28 (*m*, 6H), 3.80 (*t*, *J* = 5.6 Hz, 2H), 3.11 (*s*, 1H), 2.99 (*t*, *J* = 7.1 Hz, 2H), 2.19–2.02 (*m*, 2H); ^{13}C NMR (101 MHz, $CDCl_3$) δ (ppm): 163.72, 145.51, 135.01, 132.48, 129.14, 128.85, 128.77, 128.67, 128.29, 128.06, 126.67, 62.21, 29.66, 25.58. MS (ESI): 280.1 $[M+H]^+$. HRMS (ESI) for $C_{18}H_{18}NO_2$ $[M+H]^+$: calc'd: 280.1338; found: 280.1337.

3-(4,5-Diphenyloxazol-2-yl)propyl 4-methylbenzenesulfonate.

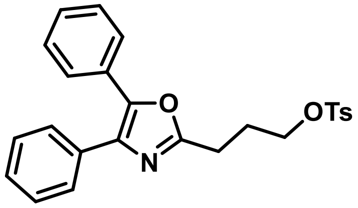

General procedure 6 from 3-(4,5-diphenyloxazol-2-yl)propan-1-ol on a 5.0 mmol scale followed by flash chromatography (ethyl acetate/n-hexane, 1: 2) gave the title compound as a colorless oil (1.93 g, 89% yield). ^1H NMR (400 MHz, $CDCl_3$) δ (ppm): 7.81 (*d*, *J* = 8.2 Hz, 2H), 7.59 (*dd*, *J* = 16.0, 7.1 Hz, 4H), 7.43–7.32 (*m*, 6H), 7.30 (*d*, *J* = 8.2 Hz, 2H), 4.25 (*t*, *J* = 6.1 Hz, 2H), 2.93 (*t*, *J* = 7.3 Hz, 2H), 2.39 (*s*, 3H), 2.25 (*p*, *J* = 6.7 Hz, 2H); ^{13}C NMR (101 MHz, $CDCl_3$) δ (ppm): 161.91, 145.55, 145.01, 135.22, 133.06, 132.59, 130.04, 129.09, 128.86, 128.75, 128.69, 128.28, 128.09, 128.04, 126.62, 69.34, 26.30, 24.27, 21.79. MS (ESI): 434.1 $[M+H]^+$. HRMS (ESI) for $C_{25}H_{24}NO_4S$ $[M+H]^+$: calc'd: 434.1426; found: 434.1431.

4,5-Diphenyl-2-(3-(2,2,2-trifluoroethoxy)propyl)oxazole (53).

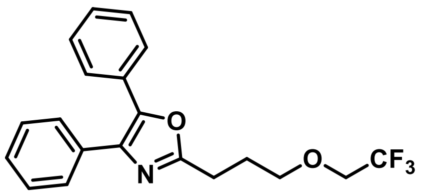

General procedure 7 with 3-(4,5-diphenyloxazol-2-yl)propyl 4-methylbenzenesulfonate on a 1.0 mmol scale followed by flash chromatography (ethyl acetate/*n*-hexane, 1: 4) gave **53** as a colorless oil (280 mg, 78% yield). ^{1}H NMR (400 MHz, $CDCl_3$) δ (ppm): 7.62 (*dd*, *J* = 25.1, 6.9 Hz, 4H), 7.45–7.28 (*m*, 6H), 3.85 (*q*, *J* = 8.7 Hz, 2H), 3.77 (*t, J* = 6.1 Hz, 2H), 2.98 (*t*, *J* = 7.5 Hz, 2H), 2.50–1.99 (*m*, 2H); ^{19}F NMR (376 MHz, $CDCl_3$) δ (ppm): –74.15 (*t*, *J* = 8.8 Hz, 3F); ^{13}C NMR (101 MHz, $CDCl_3$) δ (ppm): 162.95, 145.45, 135.28, 132.76, 129.26, 128.82, 128.75, 128.58, 128.21, 128.09, 126.62, 124.21 (*q*, *J* = 279.6 Hz), 71.70, 68.58 (*q*, *J* = 34.0 Hz), 27.16, 24.83. MS (ESI): 362.1 [*M*+H]$^+$. HRMS (ESI) for $C_{20}H_{19}NO_2F_3$ [*M*+H]$^+$: calc'd: 362.1368; found: 362.1370.

(*Z*)-2-(4-(1,2-Diphenylbuta-1,3-dien-1-yl)phenoxy)ethan-1-ol.

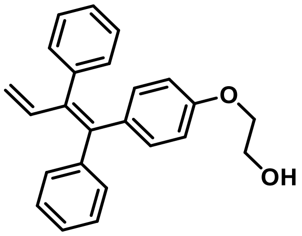

Ospemifene ((Z)-2-(4-(4-chloro-1,2-diphenylbut-1-en-1-yl)phenoxy)ethan-1-ol; 2.27 g, 6.0 mmol, 1 equiv.) and DMF (30 mL) were added to a round-bottomed flask equipped with a magnetic stirrer bar under argon. Then potassium *tert*-butoxide (1.35 g, 12 mmol, 2 equiv.) was added at 0 ^{o}C and the reaction mixture was stirred at RT for 12 h. The reaction mixture was then poured onto water and extracted with DCM (2 × 15 mL). The organic phase was dried (Mg_2SO_4), filtered, and concentrated *in vacuo*. The residue was purified by silica gel flash chromatography (ethyl acetate/*n*-hexane, 1: 4) to give the title compound as a white solid (1.95 g, 95% yield). Mp: 114–116 ^{o}C. ^{1}H NMR (400 MHz, $CDCl_3$) δ (ppm): 7.41–7.08 (*m*, 10H), 6.83–6.64 (*m*, 3H), 6.61–6.49 (*m*, 2H), 5.12 (*dd*, *J* = 10.7, 1.7 Hz, 1H), 4.90 (*dd*, *J* = 17.3, 1.7 Hz, 1H), 3.93 (*dd*, *J* = 5.1, 3.7 Hz, 2H), 3.89–3.65 (*m*, 2H), 2.05 (*t*, *J* = 6.1 Hz, 1H); ^{13}C NMR (101 MHz, $CDCl_3$) δ (ppm): 157.06, 142.72, 141.91, 140.35, 138.76, 138.54, 135.74, 132.41, 131.59, 131.14, 128.13, 128.06, 127.37, 126.65, 117.71, 113.52, 69.06, 61.59. MS (ESI): 343.2 [*M*+H]$^+$. HRMS (ESI) for $C_{24}H_{23}O_2$ [*M*+H]$^+$: calc'd: 343.1698; found: 343.1704.

(*Z*)-2-(4-(1,2-Diphenylbuta-1,3-dien-1-yl)phenoxy)ethyl 4-methylbenzenesulfonate.

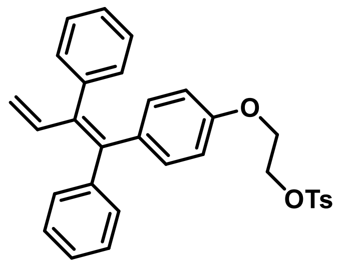

General procedure 6 with (*Z*)-2-(4-(1,2-diphenylbuta-1,3-dien-1-yl)phenoxy)ethan-1-ol on a 5.0 mmol scale followed by flash chromatography (ethyl acetate/*n*-hexane, 1: 4) gave the title compound as a pale–yellow solid (1.93 g, 78% yield). Mp: 52–54 °C. ^{1}H NMR (400 MHz, $CDCl_3$) *δ* (ppm): 7.76 (*d, J* = 8.0 Hz, 2H), 7.45–7.01 (*m*, 12H), 6.90–6.55 (*m*, 3H), 6.41 (*d*, *J* = 8.4 Hz, 2H), 5.11 (*d*, *J* = 10.8 Hz, 1H), 4.89 (*d*, *J* = 17.2 Hz, 1H), 4.49–4.13 (*m*, 2H), 3.97–3.99 (*m*, 2H), 2.40 (*s*, 3H); ^{13}C NMR (101 MHz, $CDCl_3$) *δ* (ppm): 156.41, 145.06, 142.60, 141.77, 140.23, 138.66, 138.59, 135.94, 132.98, 132.30, 131.53, 131.07, 129.99, 128.14, 128.11, 128.03, 127.36, 126.66, 117.77, 113.48, 68.26, 65.26, 21.79. MS (ESI): 514.2 [*M*+NH_4]$^+$. HRMS (ESI) for $C_{31}H_{32}NO_4S$ [*M*+ NH_4]$^+$: calc'd: 514.2052; found: 514.2045.

(*Z*)-(1-(4-(2-(2,2,2-trifluoroethoxy)ethoxy)phenyl)buta-1,3-diene-1,2-di-yl)dibenzene (54).

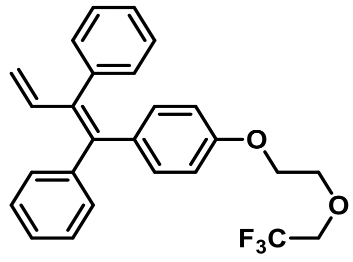

General procedure 7 with (*Z*)-2-(4-(1,2-diphenylbuta-1,3-dien-1-yl)phenoxy)ethyl 4-methylbenzenesulfonate on a 0.5 mmol scale followed by flash chromatography (ethyl acetate/*n*-hexane, 1: 20) gave **54** as a colorless oil (84 mg, 40% yield). ^{1}H NMR (400 MHz, $CDCl_3$) *δ* (ppm): 7.41–7.01 (*m*, 10H), 6.86–6.64 (*m*, 3H), 6.60–6.44 (*m*, 2H), 5.12 (*dd*, *J* = 10.7, 1.7 Hz, 1H), 4.90 (*dd*, *J* = 17.3, 1.7 Hz, 1H), 4.01 (*dd*, *J* = 5.5, 3.6 Hz, 2H), 3.96–3.73 (*m*, 4H); ^{19}F NMR (376 MHz, $CDCl_3$) *δ* (ppm): –74.27 (*t*, *J* = 8.8 Hz, 3F); ^{13}C NMR (101 MHz, $CDCl_3$) *δ* (ppm): 156.95, 142.74, 141.94, 140.36, 138.77, 138.59, 135.81, 132.41, 131.62, 131.16, 128.15, 128.07, 127.38, 126.67, 124.14 (*q*, *J* = 279.8 Hz), 117.72, 113.58, 71.24, 69.11 (*q*, *J* = 34.1 Hz), 67.36 . MS (ESI): 425.2 [*M*+H]$^+$. HRMS (ESI) for $C_{26}H_{24}O_2F_3$ [*M*+H]$^+$: calc'd: 425.1728; found: 425.1728.

(*E*)-2-(2-(4-(3,5-Dimethoxystyryl)phenoxy)ethoxy)ethanol.

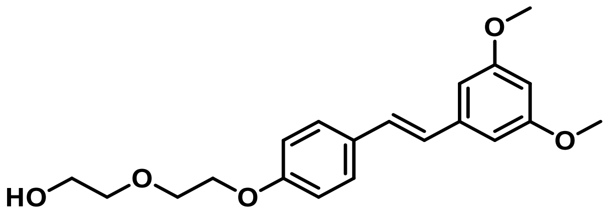

Pterostilbene (4-[(1*E*)-2-(3,5-dimethoxyphenyl)ethenyl]phenol; 2.56 g, 10.0 mmol, 1 equiv.), Cs_2CO_3 (4.89 g, 15.0 mmol, 1.5 equiv.) and DMF (30 mL) were added to a round-bottomed flask equipped with a magnetic stirrer bar under argon. The

reaction mixture was stirred at 60 °C for 12 h. The reaction mixture was then poured onto water and extracted with DCM (2 × 15 mL). The organic phase was dried (Mg_2SO_4), filtered, and concentrated *in vacuo*. The residue was purified by flash chromatography (ethyl acetate/*n*-hexane, 2: 1) to give the title compound as a colorless oil (1.41 g, 41% yield). ^{1}H NMR (400 MHz, $CDCl_3$) *δ* (ppm): 7.43 (*d*, *J* = 8.7 Hz, 2H), 7.10–6.76 (*m*, 4H), 6.65 (*d*, *J* = 2.2 Hz, 2H), 6.38 (*t*, *J* = 2.2 Hz, 1H), 4.24–4.09 (*m*, 2H), 3.87 (*dd*, *J* = 5.4, 4.0 Hz, 2H), 3.82 (*s*, 6H), 3.80–3.74 (*m*, 2H), 3.70–3.64 (*m*, 2H), 2.33 (*t*, *J* = 6.1 Hz, 1H); ^{13}C NMR (101 MHz, $CDCl_3$) *δ* (ppm): 161.13, 158.62, 139.81, 130.43, 128.81, 127.99, 126.92, 114.99, 104.52, 99.82, 72.79, 69.82, 67.62, 61.95, 55.52. MS (ESI): 345.2 [*M*+H]$^+$. HRMS (ESI) for $C_{20}H_{25}O_5$ [*M*+H]$^+$: calc'd: 345.1702; Found: 345.1708.

(*E*)-2-(2-(4-(3,5-Dimethoxystyryl)phenoxy)ethoxy)ethyl 4-methylbenzenesulfonate

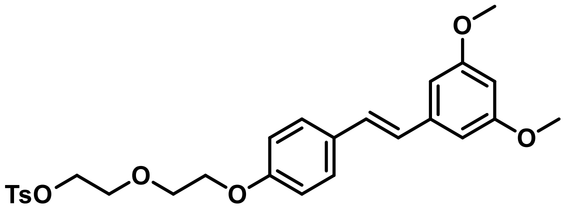

General procedure 6 with (*E*)-2-(2-(4-(3,5-dimethoxystyryl)phenoxy)ethoxy)ethanol on a 4.0 mmol followed by flash chromatography (ethyl acetate/*n*-hexane, 1: 1) gave the title compound as a colorless oil (1.62 g, 81% yield). ^{1}H NMR (400 MHz, $CDCl_3$) *δ* (ppm): 7.80 (*d*, *J* = 8.3 Hz, 2H), 7.43 (*d*, *J* = 8.7 Hz, 2H), 7.30 (*d*, *J* = 8.1 Hz, 2H), 7.12–6.81 (*m*, 4H), 6.65 (*d*, *J* = 2.2 Hz, 2H), 6.38 (*t*, *J* = 2.2 Hz, 1H), 4.28–4.15 (*m*, 2H), 4.14–3.97 (*m*, 2H), 3.83 (*s*, 6H), 3.82–3.73 (*m*, 4H), 2.41 (*s*, 3H); ^{13}C NMR (101 MHz, $CDCl_3$) *δ* (ppm): 161.16, 158.63, 145.01, 139.82, 133.15, 130.41, 130.00, 128.84, 128.17, 127.98, 126.93, 114.99, 104.54, 99.84, 70.05, 69.44, 69.10, 67.62, 55.55, 21.81. MS (ESI): 499.2 [*M*+NH_4]$^+$. HRMS (ESI) for $C_{27}H_{31}O_7S$ [*M*+NH_4]$^+$: calc'd: 499.1790; found: 499.1786.

(*E*)-1,3-Dimethoxy-5-(4-(2-(2-(2,2,2-trifluoroethoxy)ethoxy)ethoxy)styryl)benzene (55).

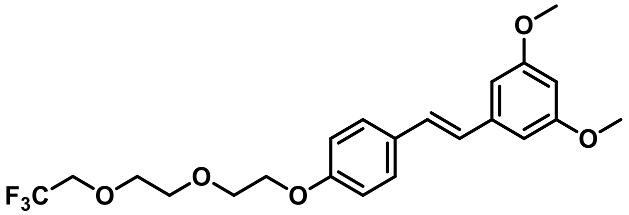

General procedure 7 with (*E*)-2-(2-(4-(3,5-dimethoxystyryl)phenoxy)ethoxy)ethyl 4-methylbenzenesulfonate on a 0.5 mmol scale followed by flash chromatography (ethyl acetate/*n*-hexane, 1: 2) gave **55** as a colorless oil (185 mg, 87% yield). ^{1}H NMR (400 MHz, $CDCl_3$) *δ* (ppm): 7.44 (*d*, *J* = 8.7 Hz, 2H), 6.96 (*dt*, *J* = 13.1, 10.3 Hz, 4H), 6.66 (*d*, *J* = 2.2 Hz, 2H), 6.39 (*t*, *J* = 2.2 Hz, 1H), 4.24–4.09 (*m*, 2H), 3.92 (*q*, *J* = 8.8 Hz, 2H), 3.87 (*dd*, *J* = 5.4, 4.1 Hz, 2H), 3.84–3.79 (*m*, 8H), 3.79–3.72 (*m*, 2H); ^{19}F NMR (376 MHz,

$CDCl_3$) δ (ppm): –74.23 (*t*, *J* = 8.8 Hz, 3F); ^{13}C NMR (101 MHz, $CDCl_3$) δ (ppm): 161.15, 158.72, 139.84, 130.34, 128.86, 127.97, 126.86, 124.22 (*q*, *J* = 279.6 Hz), 115.00, 104.52, 99.81, 72.14, 71.06, 70.04, 68.98 (*q*, *J* = 33.9 Hz), 67.61, 55.51.

(*R*)-3-fluoro-4-(4-(1-hydroxypropan-2-yloxy)phenoxy)benzonitrile.

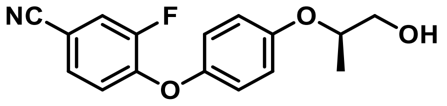

To a round-bottomed flask equipped with a magnetic stirrer bar was added $CaCl_2$ (666 mg, 6.0 mmol, 1 equiv.) and ethanol (200 proof, 30 mL), followed by $NaBH_4$ (681 mg, 18 mmol, 3 equiv.) at 0 °C. The reaction mixture was stirred for 10 min. Butyl (*R*)-2-(4-(4-cyano-2-fluorophenoxy)phenoxy)propanoate (2.14 g, 6.0 mmol, 1 equiv.) was then added and the reaction mixture was stirred at 60 °C for 3 h. The reaction mixture was quenched with water and extracted with DCM (2 × 15 mL). The organic phase was dried (Mg_2SO_4), filtered, and concentrated *in vacuo*. The residue was purified by flash chromatography (ethyl acetate/*n*-hexane, 1: 2) to give the title compound as a white solid (1.21 g, 70% yield). Mp: 86–88 °C. ^1H NMR (400 MHz, $CDCl_3$) δ (ppm): 7.44 (*dd*, *J* = 10.2, 1.9 Hz, 1H), 7.36–7.28 (*m*, 1H), 7.04–6.91 (*m*, 4H), 6.87 (*t*, *J* = 8.3 Hz, 1H), 4.46 (*m*, , 1H), 3.91–3.56 (*m*, 2H), 2.19 (*s*, 1H), 1.28 (*d*, *J* = 6.2 Hz, 3H); ^{19}F NMR (376 MHz, $CDCl_3$) δ (ppm): –130.13 (*t*, *J* = 8.9 Hz, 1F); ^{13}C NMR (101 MHz, $CDCl_3$) δ (ppm): 155.37, 152.24 (*d*, *J* = 303.6 Hz), 151.04 (*d*, *J* = 41.2 Hz), 148.34, 129.52 (*d*, *J* = 4.1 Hz), 121.30, 120.70 (*d*, *J* = 21.2 Hz), 118.59 (*d*, *J* = 1.8 Hz), 117.85 (*d*, *J* = 2.5 Hz), 117.75, 106.05 (*d*, *J* = 8.2 Hz), 75.72, 66.35, 15.95.

(*R*)-2-(4-(4-Cyano-2-fluorophenoxy)phenoxy)propyl 4-methylbenzenesulfonate.

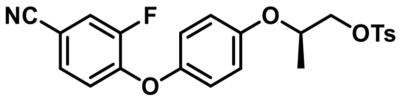

General procedure 6 with (*R*)-3-fluoro-4-(4-(1-hydroxypropan-2-yloxy)phenoxy)benzonitrile on a 4.0 mmol scale followed by flash chromatography (ethyl acetate/*n*-hexane, 1: 3) gave the title product as a colorless oil (1.53 g, 86% yield). Mp: 80–82 °C; ^1H NMR (400 MHz, $CDCl_3$) δ (ppm): 7.78 (*d*, *J* = 8.3 Hz, 2H), 7.45 (*dd*, *J* = 10.2, 1.9 Hz, 1H), 7.34 (*dd*, *J* = 8.5, 3.4 Hz, 3H), 7.03–6.91 (m, 2H), 6.91–6.77 (*m*, 3H), 4.65–4.43 (*m*, 1H), 4.12 (*ddd*, *J* = 14.9, 10.6, 5.2 Hz, 2H), 2.44 (*s*, 3H), 1.31 (*d*, *J* = 6.3 Hz, 3H); ^{19}F NMR (376 MHz, $CDCl_3$) δ (ppm): –130.06 (*t*, *J* = 9.0 Hz, 1F); ^{13}C NMR (101 MHz, $CDCl_3$) δ (ppm): 154.87 (*s*), 152.54 (*d*, *J* = 251.7 Hz), 150.69 (*d*, *J* = 10.6 Hz), 148.65, 145.21, 132.97, 130.07, 129.55 (d, *J* = 3.8 Hz), 128.13, 121.23, 120.74 (d, *J* = 21.2 Hz), 118.72 (d, *J* = 1.6 Hz), 117.85 (d, *J* =

2.4 Hz), 117.80, 106.20 (d, J = 8.1 Hz), 72.50, 71.89, 21.83, 16.68. MS (ESI): 459.1 $[M+NH_4]^+$. HRMS (ESI) for $C_{23}H_{24}N_2O_5FS$ $[M+NH_4]^+$: calc'd: 459.1390; found: 459.1398.

(*R*)-3-fluoro-4-(4-(1-(2,2,2-trifluoroethoxy)propan-2-yloxy)phenoxy)benzonitrile (56).

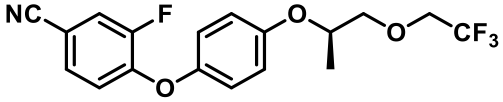

General procedure 7 with (*R*)-2-(4-(4-cyano-2-fluorophenoxy)phenoxy)propyl 4-methylbenzenesulfonate on a 0.5 mmol scale followed by flash chromatography (ethyl acetate/*n*-hexane, 1: 10) gave **56** as a colorless oil (78 mg, 42% yield). ^{1}H NMR (400 MHz, $CDCl_3$) δ (ppm): 7.44 (*dd*, J = 10.2, 1.9 Hz, 1H), 7.39–7.30 (*m*, 1H), 7.08–6.92 (*m*, 4H), 6.87 (*t*, J = 8.3 Hz, 1H), 4.64–4.35 (*m*, 1H), 4.09–3.86 (*m*, 2H), 3.84–3.69 (*m*, 2H), 1.33 (*d*, J = 6.3 Hz, 3H); ^{19}F NMR (376 MHz, $CDCl_3$) δ (ppm): –74.30 (*t*, J = 9.1 Hz, 3F); –130.21 (d, J = 9.7 Hz, 1F); ^{13}C NMR (101 MHz, $CDCl_3$) δ (ppm): 155.37, 152.27 (*d*, J = 302.7 Hz), 151.07 (*d*, J = 40.1 Hz), 148.41, 129.53 (*d*, J = 4.0 Hz), 124.11 (*q*, J = 279.8 Hz), 121.30, 120.70 (*d*, J = 21.2 Hz), 118.61 (*d*, J = 1.6 Hz), 117.88 (d, J = 2.4 Hz), 117.82, 106.09 (*d,* J = 8.1 Hz), 75.82, 74.29, 69.18 (*q*, J = 34.2 Hz), 16.67 .

2.4. Synthesis of PS13 analogue 57 and precursor for radiolabeling.

2-(1,5-Bis(4-methoxyphenyl)-1*H*-1,2,4-triazol-3-yloxy)ethanol.

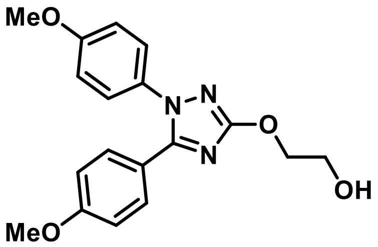

1,5-Bis(4-methoxyphenyl)-1*H*-1,2,4-triazol-3-ol (1.49 g, 5.0 mmol, 1 equiv.), 2-bromoethanol (1.25 g, 10 mmol, 2 equiv.) and Cs_2CO_3 (3.26 g, 10 mmol, 2 equiv.) were added to a round-bottomed flask equipped with a magnetic stirrer bar. The flask was thrice evacuated and backfilled with pure argon. DMF (20 mL) was added from a syringe and the mixture was stirred for 12 h at 80 ^oC under argon. Water (30 mL) was added. The mixture was extracted with DCM (2 × 15 mL), washed with brine, dried (Mg_2SO_4), and concentrated under reduced pressure. The residue was purified by flash chromatography (ethyl acetate/*n*-hexane, 3: 1) to give the title compound (1.23 g, 72% yield) as a white solid. Mp: 104–106 ^oC. ^{1}H NMR (400 MHz, $CDCl_3$) δ (ppm): 7.38–7.26 (*m*, 2H), 7.25–7.12 (m, 2H), 6.95–6.80 (*m*, 2H), 6.80–6.67 (*m*, 2H), 4.50–4.28 (*m*, 2H), 3.90 (*d*, J = 3.3 Hz, 2H), 3.77 (*s*, 3H), 3.73 (*s*, 3H), 3.11 (*s*, 1H); ^{13}C NMR (101 MHz, $CDCl_3$) δ (ppm): 167.66, 161.08, 159.91, 153.22, 131.40, 130.43,

127.26, 120.03, 114.76, 114.11, 71.74, 62.03, 55.74, 55.49. MS (ESI): 274.0 [*M*+H]$^+$. HRMS (ESI) for $C_{18}H_{20}N_3O_4$ [*M*+H]$^+$: calc'd: 342.1454; found: 342.1457.

2-(1,5-Bis(4-methoxyphenyl)-1*H*-1,2,4-triazol-3-yloxy)ethyl 4-methylbenzenesulfonate.

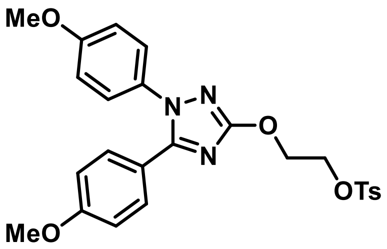

General procedure 6 with 2-(1,5-bis(4-methoxyphenyl)-1*H*-1,2,4-triazol-3-yloxy)ethanol on a 3.0 mmol scale followed by flash chromatography (ethyl acetate/*n*-hexane, 1: 1) gave the title compound as a colorless oil (1.39 g, 93% yield). ^{1}H NMR (400 MHz, $CDCl_3$) δ (ppm): 7.82 (*d*, *J* = 8.3 Hz, 2H), 7.43–7.35 (*m*, 2H), 7.32 (*d*, *J* = 8.0 Hz, 2H), 7.25–7.17 (*m*, 2H), 6.96–6.86 (*m*, 2H), 6.86–6.76 (*m*, 2H), 4.52 (*dd*, *J* = 5.6, 3.8 Hz, 2H), 4.41 (*dd*, *J* = 5.5, 3.8 Hz, 2H), 3.84 (*s*, 3H), 3.80 (*s*, 3H), 2.40 (*s*, 3H); ^{13}C NMR (101 MHz, $CDCl_3$) δ (ppm): 167.10, 161.14, 159.98, 153.35, 145.12, 133.07, 131.50, 130.48, 130.11, 128.32, 127.28, 120.18, 114.83, 114.16, 68.00, 66.85, 55.83, 55.57, 21.91. MS (ESI): 496.1 [*M*+H]$^+$. HRMS (ESI) for $C_{25}H_{26}N_3O_6S$ [*M*+H]$^+$: calc'd: 496.1542; found: 496.1537.

General procedure 8.

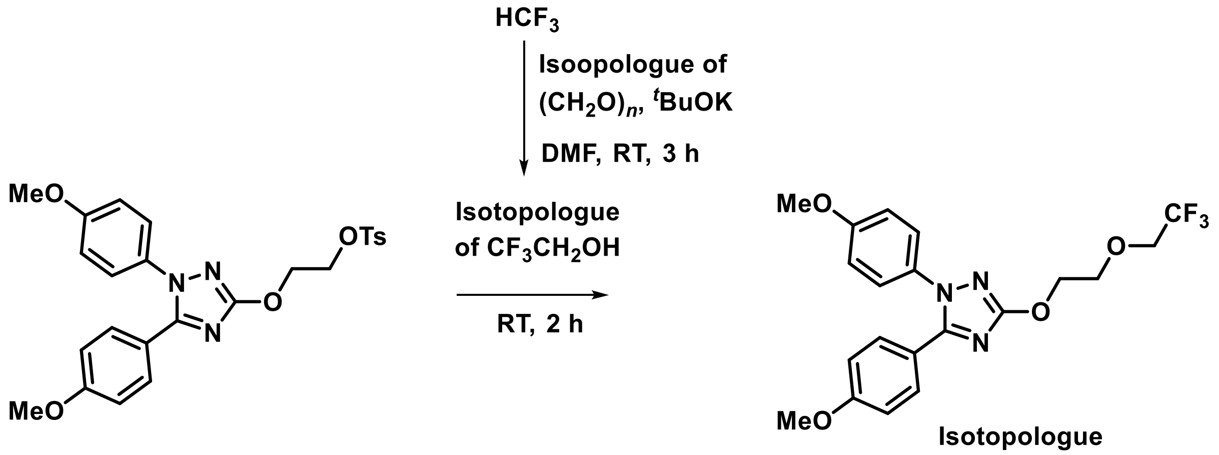

Supplementary Figure 10. General procedure 8 for the synthesis of PS13 analogues.

1,5-Bis(4-methoxyphenyl)-3-(2-(2,2,2-trifluoroethoxy)ethoxy)-1*H*-1,2,4-triazole.

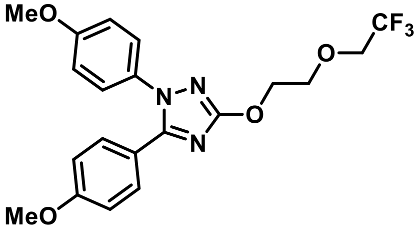

The synthesis of this compound exemplifies general procedure 8, as follows. To a round-bottomed flask equipped with a magnetic stirrer bar was added paraformaldehyde (15 mg, 0.5 mmol, 3.0 equiv.) and *t*-BuOK (56 mg, 0.5 mmol, 3.0 equiv.), and then DMF (3

mL) under argon. A DMF solution of fluoroform (0.3 M, 0.17 mmol, 0.6 mL) was added and the reaction was stirred at RT for 3 h. 2-(1,5-Bis(4-methoxyphenyl)-1*H*-1,2,4-triazol-3-yloxy)ethyl 4-methylbenzenesulfonate (248 mg, 0.5 mmol, 3 equiv.) was added and the reaction stirred for another 2 h. Water (2 mL) was added and the mixture was extracted with DCM (2 × 5 mL), washed with brine, dried (Mg_2SO_4), and concentrated under reduced pressure. The residue was purified by flash chromatography (ethyl acetate/*n*-hexane, 1: 2) to give the product (52 mg, 72% yield) as a colorless oil. ^{1}H NMR (400 MHz, $CDCl_3$) δ (ppm): 7.50–7.35 (*m*, 2H), 7.35–7.17 (*m*, 2H), 6.99–6.87 (*m*, 2H), 6.87–6.69 (*m*, 2H), 4.64–4.46 (*m*, 2H), 4.12–3.91 (*m*, 4H), 3.85 (*s*, 3H), 3.81 (*s*, 3H); ^{19}F NMR (376 MHz, $CDCl_3$) δ (ppm): –74.34 (*t*, *J* = 8.6 Hz, 3F); ^{13}C NMR (101 MHz, $CDCl_3$) δ (ppm): 167.49, 161.05, 159.90, 153.35, 131.53, 130.45, 127.28, 124.18 (*q*, *J* = 279.5 Hz), 120.23, 114.76, 114.09, 71.04, 69.18 (*q*, *J* = 33.9 Hz), 69.08, 55.74, 55.48. MS (ESI): 424.2 [*M*+H]$^+$. HRMS (ESI) for $C_{20}H_{21}N_3O_4F_3$ [*M*+H]$^+$: calc'd: 424.1484; found: 424.1478.

1,5-bis(4-methoxyphenyl)-3-(2-(2,2,2-trifluoroethoxy-1,1-*d*$_2$)ethoxy)-1*H*-1,2,4-triazole.

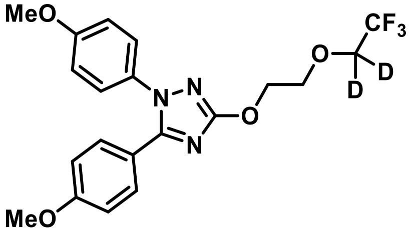

General procedure 8 with paraformaldehyde-*d*$_2$ ((CD_2O)$_n$) instead of paraformaldehyde followed by flash chromatography (ethyl acetate/*n*-hexane, 1: 2) gave the title compound as a colorless oil (35 mg, 48% yield). ^{1}H NMR (400 MHz, $CDCl_3$) δ (ppm): 7.51–7.37 (*m*, 2H), 7.37–7.14 (*m*, 2H), 7.01–6.86 (*m*, 2H), 6.86–6.74 (*m*, 2H), 4.59–4.43 (*m*, 2H), 4.10–3.94 (*m*, 2H), 3.84 (*s*, 3H), 3.80 (*s*, 3H); ^{19}F NMR (376 MHz, $CDCl_3$) δ (ppm): –74.49 (*s*, 3F); ^{13}C NMR (101 MHz, $CDCl_3$) δ (ppm): 167.47, 161.06, 159.91, 153.36, 131.53, 130.46, 127.29, 124.17 (*q*, *J* = 279.6 Hz), 120.21, 114.77, 114.10, 70.97, 69.12, 55.76, 55.50. MS (ESI): 426.2 [M+H]$^+$. HRMS (ESI) for $C_{20}{}^1H_{19}{}^2H_2N_3O_4F_3$ [*M*+H]$^{+:}$ calc'd: 426.1610; found: 426.1604.

1,5-Bis(4-methoxyphenyl)-3-(2-(2,2,2-trifluoroethoxy-1-^{13}C)ethoxy)-1*H*-1,2,4-triazole.

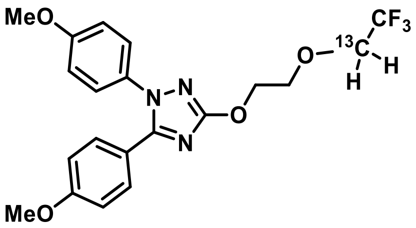

General procedure 8 with [^{13}C]paraformaldehyde (($^{13}CH_2O$)$_n$) instead of paraformaldehyde followed by flash chromatography (ethyl acetate/*n*-hexane, 1: 2) to give the title compound as a colorless oil (40 mg, 56%

yield). ^{1}H NMR (400 MHz, $CDCl_3$) δ (ppm): 7.50–7.36 (*m*, 2H), 7.35–7.16 (*m*, 2H), 7.00–6.87 (*m*, 2H), 6.87–6.67 (*m*, 2H), 4.65–4.45 (*m*, 2H), 4.17 (*q*, *J* = 8.7 Hz, 1H), 4.08–3.96 (*m*, 2H), 3.84–3.78 (*m*, 7H); ^{19}F NMR (376 MHz, $CDCl_3$) δ (ppm): –68.59 to –81.62 (*m*, 3F); ^{13}C NMR (101 MHz, $CDCl_3$) δ (ppm): 167.48, 161.05, 159.90, 153.36, 131.53, 130.46, 127.29, 120.22, 114.77, 114.09, 71.04, 69.20 (*q*, *J* = 34.2 Hz), 55.76, 55.50. MS (ESI): 425.2 $[M+H]^+$. HRMS (ESI) for $^{12}C_{19}{}^{13}C_1H_{21}N_3O_4F_3$ $[M+H]^+$: calc'd: 425.1518; found: 425.1515.

3. General HPLC methods

Column: Luna C18(2) (10 μm, 250 × 4.6 mm; i.d. Phenomenex).

The detection wavelength used for HPLC measurements was 254 nm.

Radioactivity was measured using an in-line Flow-Count PMT radioactivity detector (Eckert & Ziegler)).

3.1. HPLC condition A

Solvent A: H_2O, Solvent B: MeCN

Flow rate: 2 mL/min

0–1 min: 40% B

1–3 min: 40% to 60% B

3–6 min: 60% to 95% B

6–12 min: 95% B

3.2. HPLC condition B

Solvent A: H_2O, Solvent B: MeCN

Flow rate = 2 mL/min

0–1 min: 30% B

1–5 min: 30% to 50% B

5–10 min: 50% to 90% B

10–12 min: 90% B

3.3. HPLC condition C

Solvent A: H_2O, Solvent B: MeCN

Flow rate = 2 mL/min

0–1 min: 30% B

1–15 min: 30% to 80% B

3.4. HPLC condition D

Solvent A: H_2O, Solvent B: MeCN

Flow rate = 2 mL/min

0–1 min: 30% B

1–5 min: 30% to 50% B

5–10 min: 90% B

10–15 min: 90% B

3.5. HPLC condition E

Solvent A: H_2O, Solvent B: MeCN

Flow rate = 2 mL/min

0–5 min: 30% B

5–10 min: 30% to 50% B

10–12 min: 50% to 90% B

4. Syntheses of [^{11}C]fluoroform and [^{18}F]fluoroform

4.1. General procedure for the preparation of [^{11}C]fluoroform [13]

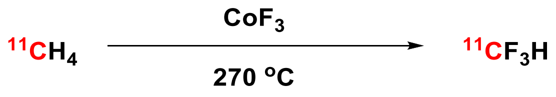

Supplementary Figure 11. The preparation of [^{11}C]fluoroform from [^{11}C]methane.

4.1.1. The preparation of [^{11}C]methane.

[^{11}C]Methane was produced by the ^{14}N(p,α)^{11}C nuclear reaction by irradiation of nitrogen gas (164 psi) containing hydrogen (10%) with a proton beam (16.5 MeV; 5–45 *μ*A) generated with a PETtrace 200 cyclotron (GE Healthcare) for 3 to 10 min.

4.1.2. The preparation of [^{11}C]fluoroform.

The apparatus for [^{11}C]fluoroform synthesis is shown in Supplementary Figure 12. [^{11}C]Methane (3.7–5.5 GBq) was trapped by passing gas from the irradiated cyclotron

target through both cooled U-traps and out into a bag for the safe collection of the nitrogen-hydrogen gas mixture and any untrapped radioactivity. When radioactivity in the cooled Porapak [^{11}C]methane trap had maximized, as indicated by a nearby radiation detector, the liquid argon coolant was removed. The trap was then allowed to warm to RT under a controlled helium flow (20 mL/min) to direct and pass [^{11}C]methane through a heated CoF_3 column, through the cooled HF trap, and into the cooled [^{11}C]fluoroform collection trap. This transfer generally took about 15 min. Then [^{11}C]fluoroform trap was put in warm water (60 ^{o}C) for another 35-45 s to release [^{11}C]fluoroform into a vial containing DMF (0.8 mL) at –40 ^{o}C. The yield of [^{11}C]fluoroform from [^{11}C]methane was usually 35–55% accompanied by 10–50% [^{11}C]fluoromethane. This solution was used for further reactions. The yields of the reaction products are based on the [^{11}C]fluoroform and calculated by decay-correction to the beginning of HPLC analysis. We confirmed that all the radioactivity injected onto the HPLC column was fully recovered.

4.1.3. HPLC analysis of [^{11}C]fluoroform.

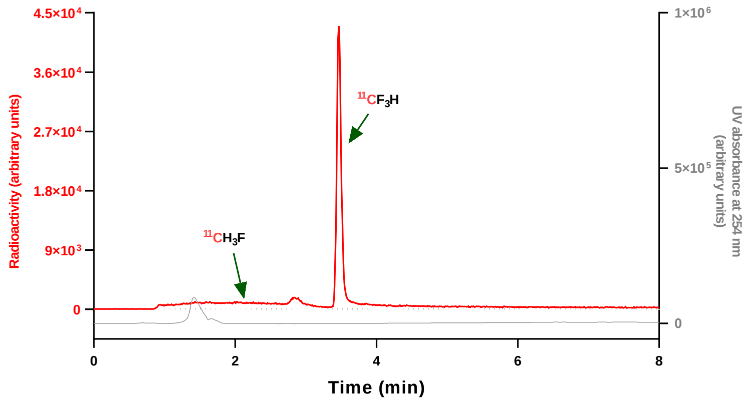

Supplementary Figure 12. Analytical HPLC chromatogram for [^{11}C]fluoroform in collection solvent. [^{11}C]Fluoroform solution in DMF (0.8 mL) was analyzed with HPLC condition A. HPLC yields of [^{11}C]fluoroform were around 50–85% and the byproduct of this production was [^{11}C]fluoromethane.

4.2. General procedure for the preparation of [^{18}F]fluoroform.[14]

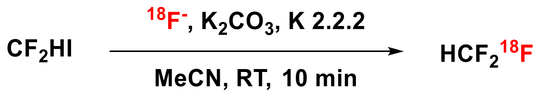

Supplementary Figure 13. The preparation of [^{18}F]fluoroform from difluoroiodomethane.

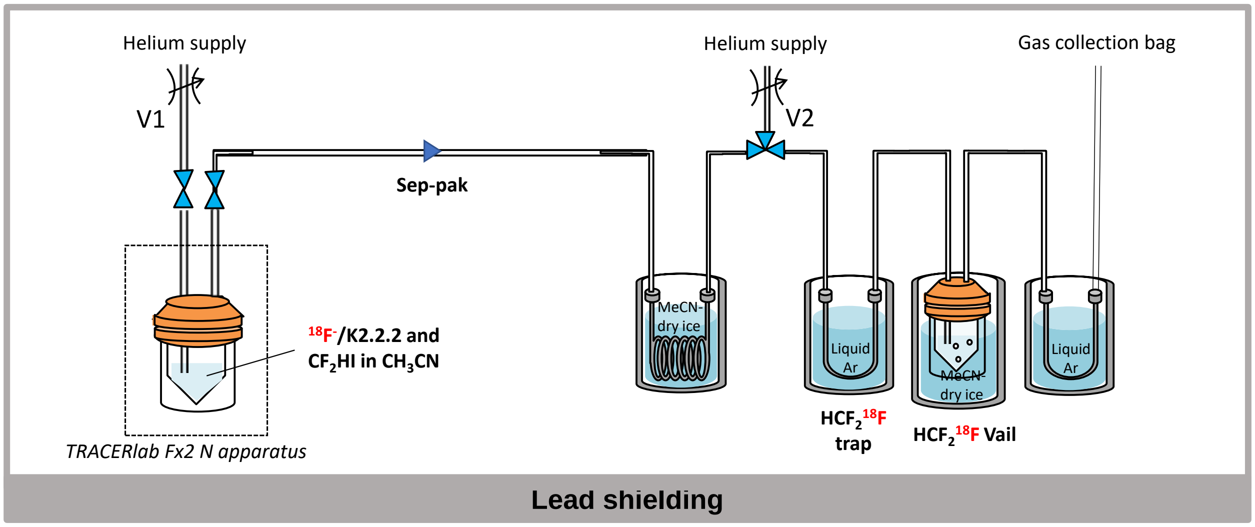

Supplementary Figure 14. Apparatus for [^{18}F]fluoroform synthesis from difluoroiodomethane.

4.2.1. Preparation of [^{18}F]fluoroform.

The apparatus for [^{18}F]fluoroform synthesis is depicted in Supplementary Figure 15. [^{18}F]Fluoride ion was produced on a cyclotron (PETtrace; GE Healthcare) according to the ^{18}O(p,n)^{18}F reaction by irradiating ^{18}O-enriched water (3 mL, 98 atom%) with a beam of protons (35 MeV; 35-45 μA) for at least 45 min. [^{18}F]Fluoroforrm was synthesized within a fully automated apparatus (TRACERlab^{TM} FX2 NFX2N; GE Healthcare). Thus, [^{18}F]fluoride ion (3.72–11.1 GBq) in [^{18}O]water (200–400 μL) and a solution (100 μL) containing K_2CO_3 (3.4 μmol) plus K 2.2.2 (13.6 μmol) were loaded into a glass vial. MeCN (2 mL) was added, and the solvent was azeotropically removed at 80–100 ^{o}C under a stream of nitrogen gas that was vented to vacuum. This step was repeated after a second addition of MeCN (2 mL). A solution of difluoroiodomethane (8.0 mg, 45 μmol) in anhydrous acetonitrile (1.0 mL) was then added to the dried [^{18}F]fluoride-K_2CO_3/K 2.2.2 complex, sealed, and heated at 35 °C for 10 min. [^{18}F]Fluoroform was flushed out of the vial with helium (20 mL/min) and into the [^{18}F]fluoroform trap. The transfer generally required 5 min. Then the [^{18}F]fluoroform trap was put in warm water (60 ^{o}C) for another

35 s to release [^{18}F]fluoroform into a vial containing DMF (0.8 mL) and finally [^{18}F]fluoroform solvent (35–65% yield from dried [^{18}F]fluoride). The solution in the vial was used for further reactions.

4.2.2. HPLC analysis of [^{18}F]fluoroform.

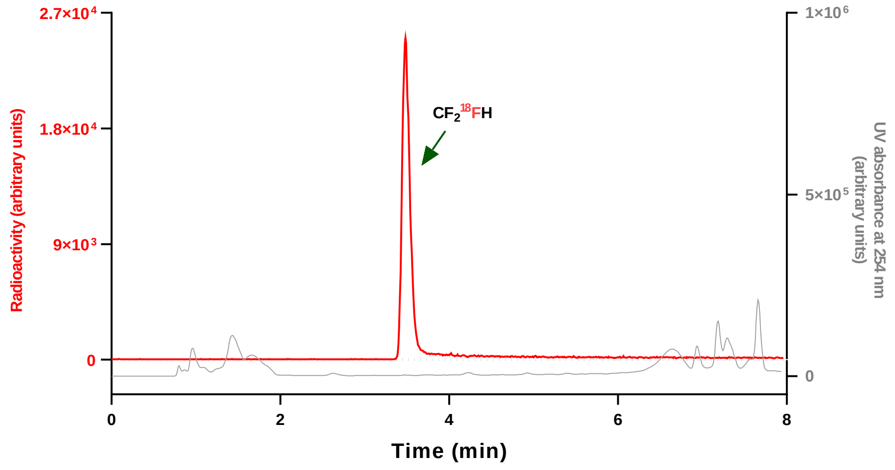

Supplementary Figure 15. Analytical HPLC chromatogram for [^{18}F]fluoroform in collection solvent. [^{18}F]Fluoroform in DMF (0.8 mL) was analyzed using HPLC condition A. HPLC yields of [^{18}F]fluoroform were quantitative (>99% yield).

5. General procedure for producing [^{11}C/^{18}F]2,2,2-trifluoroethanol.

5.1. Optimization of the preparation of [^{11}C]2,2,2-trifluoroethanol.

Procedure

To a 10-mL vial contained within a glovebox was added *t*-BuOK and paraformaldehyde, followed by DMF (6 mL). The *t*-BuOK-paraformaldehyde mixture in DMF was kept in the glovebox until further use i.e., just before adding to [^{11}C]fluoroform (~ 30 min). A V-vial (1 mL) containing the DMF reagent mixture (200 μL) was closed with a septum-sealed cap and removed from the glovebox. [^{11}C]Fluoroform (37–296 MBq) in DMF (50–300 μL) was added to the vial, mixed by shaking, and left at RT for *t* min. The reaction was quenched by addition of water (100 μL). Then the reaction mixture was analyzed with radio-HPLC using HPLC condition A. Results are shown in Supplementary Table 2.

Supplementary Table 2. Optimization of the preparation of [^{11}C]2,2,2-trifluoroethanol from [^{11}C]fluoroform.

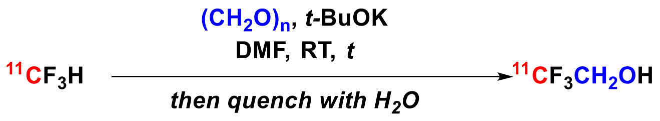

Entry	n [$(CH_2O)_n$]: n [*t*-BuOK]	n [^{t}BuOK] (μmol)	*t* (min)	Radiochemical yield (%)[a]
1	1: 3	50	2	84 ± 10
2	1: 3	50	3	88 ± 1
3	1: 3	50	4	94 ± 0
4	1: 3	50	5	95 ± 1
5	1: 1	50	4	97 ± 2
6	1: 2	50	4	> 99 (n > 5)
7	1: 4	50	4	73 ± 0
8	1: 2	40	4	96 ± 0
9	1: 2	30	4	91 ± 1

[a] Decay-corrected. n = 2 unless otherwise stated.

5.2. General procedure for the preparation of [^{11}C]2,2,2-trifluoroethanol and [^{18}F]2,2,2-trifluoroethanol.

5.2.1. General procedure A.

To a 10-mL vial within a glovebox was added *t*-BuOK (112.2 mg, 1.0 mmol) and paraformaldehyde ($(CH_2O)_n$; 15.0 mg, 0.5 mmol), followed by DMF (6 mL). The DMF mixture of *t*-BuOK-$(CH_2O)_n$ was stored in the glovebox for about 30 min. A V-vial (1 mL) containing of DMF reagent mixture (200 μL) was closed with a septum-sealed cap and removed from the glovebox. [^{11}C]Fluoroform or [^{18}F]fluoroform (37–296 MBq) in DMF (50–300 μL) was added to the vial, mixed by shaking, and left at RT for 4 min. The reaction was quenched with water (100 μL) and then the mixture analyzed by HPLC. The corresponding products, $^{11}CF_3CH_2OH$ or $CF_2{}^{18}FCH_2OH$, were formed in high yields (more than 95% in most cases).

5.2.2. HPLC analysis for [^{11}C]2,2,2-trifluoroethanol.

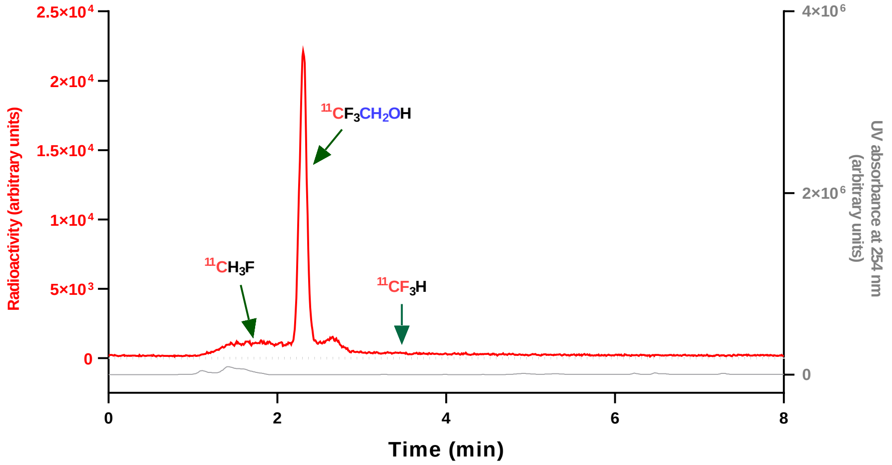

Supplementary Figure 16. Analytical HPLC chromatogram for crude [^{11}C]2,2,2-trifluoroethanol reaction mixture. [^{11}C]2,2,2-trifluoroethanol was synthesized according to general procedure 1 and analyzed with HPLC condition A. HPLC yields of 2,2,2-trifluoroethanol were high (>99%) based on consuming [^{11}C]fluoroform (in most cases within 4 min).

5.2.3. HPLC analysis for [^{18}F]2,2,2-trifluoroethanol.

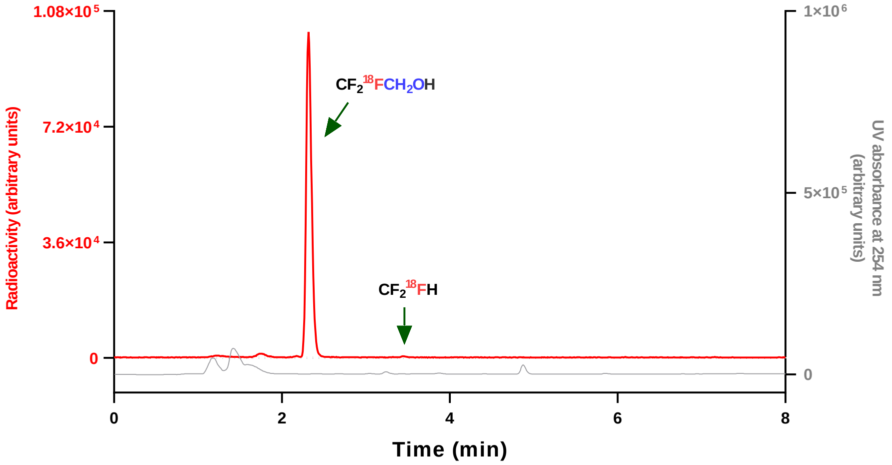

Supplementary Figure 17. Analytical HPLC chromatogram for crude [^{11}C]2,2,2-trifluoroethanol. Reaction mixture. [^{11}C]2,2,2-trifluoroethanol was synthesized according to general procedure 1 and analyzed with HPLC condition A. HPLC yields of [^{11}C]2,2,2-trifluoroethanol. were high (> 95%) (in most cases within 4 min).

5.3. General procedure for the preparation of [^{11}C/^{18}F]2,2,2-trifluoroethanol isotopologues.

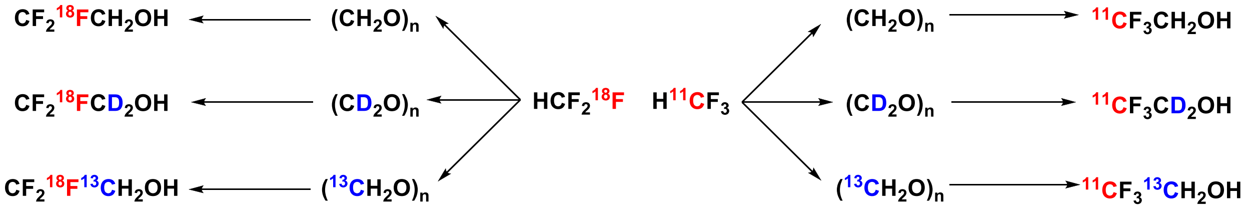

Supplementary Figure 18. The preparation of [^{11}C/^{18}F]2,2,2-trifluoroethanol isotopologues from radiolabeled fluoroform and isotopically labeled paraformaldehyde.

General procedure 1 was used for preparing $^{11}CF_3CD_2OH$, $CF_2{}^{18}FCD_2OH$, $^{11}CF_3{}^{13}CH_2OH$ and $CF_2{}^{18}F^{13}CH_2OH$ from the appropriately isotopically labeled paraformaldehyde. Deuterated paraformaldehyde $(CD_2O)_n$ was used for the preparations of $^{11}CF_3CD_2OH$ and $CF_2{}^{18}FCD_2OH$. [^{13}C]Paraformaldehyde (($^{13}CH_2O$))$_n$ was used for the preparations of $^{11}CF_3{}^{13}CH_2OH$ and $CF_2{}^{18}F^{13}CH_2OH$. The different [^{11}C/^{18}F]2,2,2-trifluoroethanol isotopologues were obtained in high yields (> 95% within 4 min).

6. General procedure for radio-2,2,2-trifluoroethoxylation.

6.1 The preparation of ^{11}C- or ^{18}F- labeled 2,2,2-trifluoroethyl heterocyclic ethers.

General procedure B.

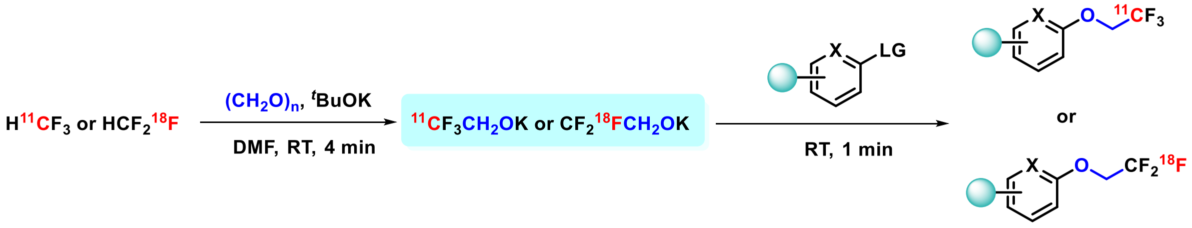

Supplementary Figure 19. The preparation of radiolabeled 2,2,2-trifluoroethyl heterocyclic ethers.

To a 10-mL vial within a glovebox, was added *t*-BuOK (112.2 mg, 1.0 mmol) and $(CH_2O)_n$ (15.0 mg, 0.5 mmol), followed by DMF (6 mL). The *t*-BuOK-$(CH_2O)_n$ mixture in DMF was kept in the glovebox until further use (~ 30 min). A V-vial (1 mL) containing this reagent mixture in DMF (200 μL) was closed with a septum-sealed cap, removed from the glovebox, and taken to the hot-cell for further reaction. [^{11}C]Fluoroform or [^{18}F]fluoroform (37–296 MBq) in DMF (50–300 μL) was added to the vial, mixed by shaking, and left at RT for 4 min. A DMF solution of heterocyclic precursor (50 μmol, 200 μL) was added to

the reaction vial and the reaction mixture was allowed to react at RT for another 1 min. The reaction was quenched with water (100 μL) and the reaction mixture was analyzed with radio-HPLC.

6.2. Optimization of the preparation of ^{11}C- or ^{18}F- labeled 2,2,2-trifluoroethyl aryl ethers.

6.2.1. Optimization.

Supplementary Table 3. Optimization of the preparation of [^{11}C]1–(2,2,2-trifluoroethoxy)naphthalene.

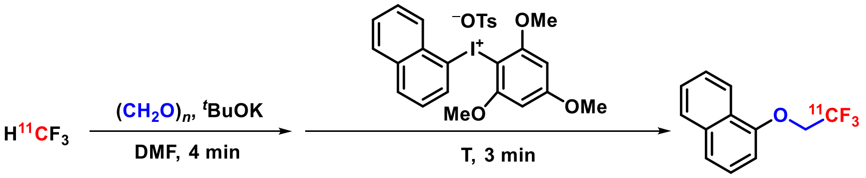

Entry	substrate (μmol)	T (°C)	Radiochemical yield (%)[a]
1	55	rt	65 ± 7
2	55	60	87 ± 4 (*n* = 3)
3	55	80	85 ± 1
4	55	100	85 ± 0
5	30	60	49 ± 9
6	15	60	< 1

[a] Decay-corrected, *n* = 2 unless otherwise statted

6.2.2. General procedure C.

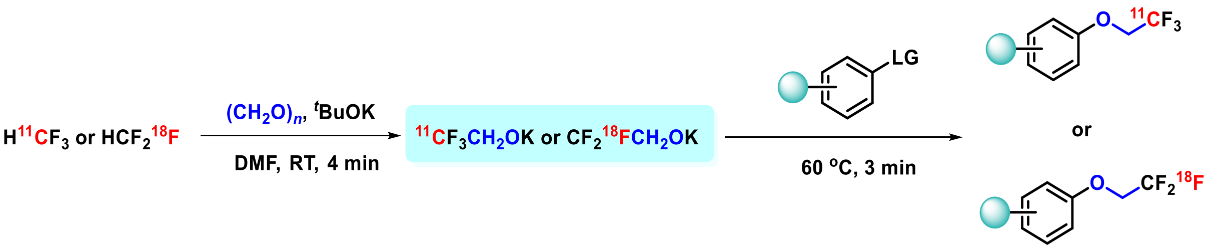

Supplementary Figure 20. The preparation of radiolabeled 2,2,2-trifluoroethyl aryl ethers.

Within a glovebox, to a 10 mL vial was added *t*-BuOK (112.2 mg, 1.0 mmol) and $(CH_2O)_n$ (15.0 mg, 0.5 mmol), followed by DMF (6 mL). The *t*-BuOK-$(CH_2O)_n$ mixture in DMF was kept in the glovebox until further use (~ 30 min). A V-vial (1 mL) containing this reagent mixture in DMF (200 μL) was closed with a septum-sealed cap, removed from the glovebox, and taken to hot cell for further reaction. [^{11}C]Fluoroform or [^{18}F]fluoroform (37–296 MBq) in DMF (50–300 μL) was added to the vial, mixed by shaking, and left at RT for

4 min. A DMF solution of benzenoid precursor, TMP iodonium salt or iodonium ylide (55 μmol, in 200 μL) was,added to the vial and the reaction mixture was heated at 60 °C for 3 min. The reaction was then quenched with water (100 μL) and the reaction mixture was analyzed with radio-HPLC.

6.3. Optimization of the preparation of ^{11}C- or ^{18}F- labeled 2,2,2-trifluoroethyl alkyl ethers.

6.3.1. Optimization.

Supplementary Table 4. Optimization of the preparation of a ^{11}C-labeled 2,2,2-trifluoroethyl alkyl ether.

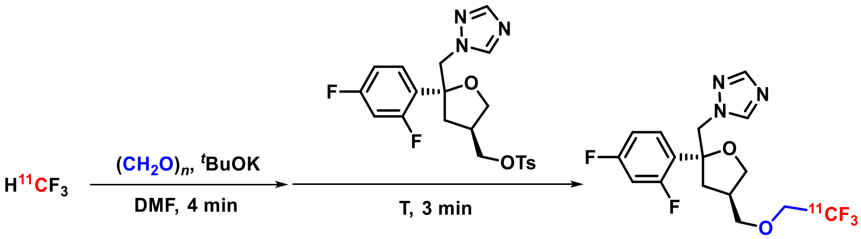

Entry	*n* [^{t}BuOK] (μmol)	T (°C)	Radiochemical yield (%)[a]
1	55	rt	52 ± 2
2	55	40	72 ± 3
3	55	60	89 ± 6 (*n* =3)
4	55	80	80 ± 9
5	30	60	15 ± 1
6	15	60	2 ± 1

[a] Decay-corrected. *n* = 2 unless otherwise stated

6.3.2. General procedure D.

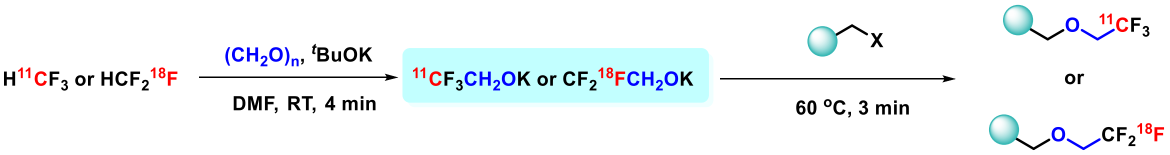

Supplementary Figure 21. The preparation of radiolabeled 2,2,2-trifluoroethyl alkyl ethers.

To a 10-mL vial within a glovebox, was added *t*-BuOK (112.2 mg, 1.0 mmol) and $(CH_2O)_n$ (15.0 mg, 0.5 mmol), followed by DMF (6 mL). The *t*-BuOK-$(CH_2O)_n$ mixture in DMF was kept in the glovebox until further use (~ 30 min). A V-vial (1 mL) containing this reagent mixture in DMF (200 μL) was closed with a septum-sealed cap, removed from the glovebox, and taken to a hot-cell for further reaction. [^{11}C]Fluoroform or [^{18}F]fluoroform (37–296 MBq) in DMF (50–300 μL) was added to the vial, mixed by shaking, and left at

RT for 4 min. A DMF solution (200 μL) of alkyl precursor (55 μmol) was added and the reaction mixture heated at 60 °C for another 3 min. The reaction was quenched with water (100 μL) and the reaction mixture analyzed with radio-HPLC.

6.4. The preparation of radiolabeled PS13 analogues.

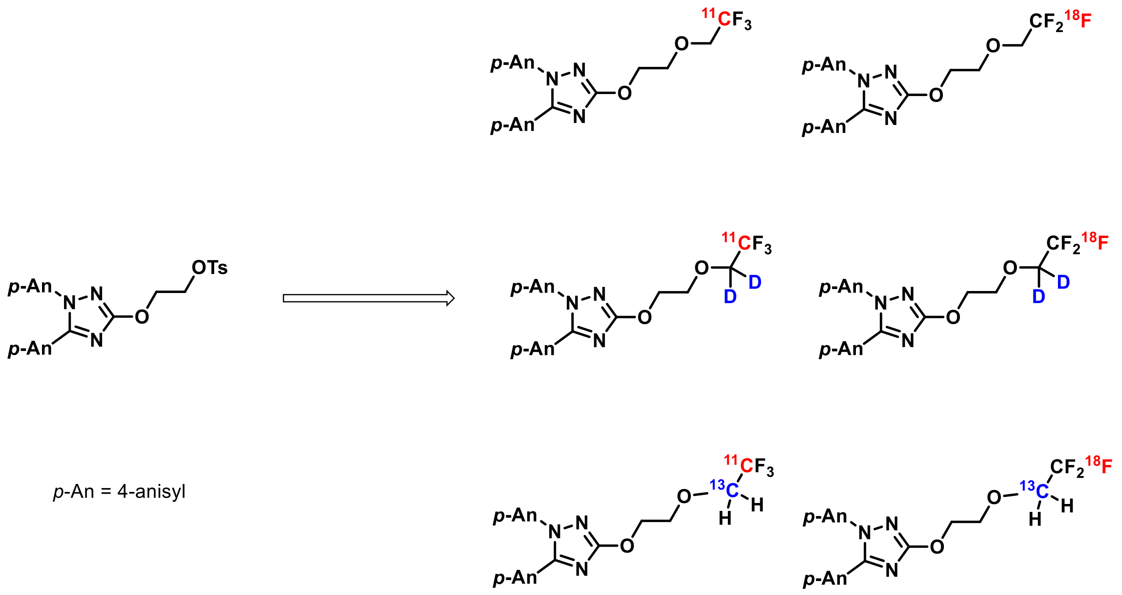

Supplementary Figure 22. The preparation of radiolabeled PS13 analogues. These followed general procedure D using different isotopic paraformaldehydes. For the preparation of d_2-labled products, $(CD_2O)_n$ was used instead of $(CH_2O)_n$. For the preparation of ^{13}C-labeled products, $(^{13}CH_2O)_n$ was used instead of $(CH_2O)_n$. All the radioactive product identities were confirmed by LC-MS analysis of non–radioactive carrier after complete decay.

6.5. Determination of the molar activity for [^{11}C]1.

The molar activity of [^{11}C]**1** was measured by HPLC determination of mass of carrier in a sample of analyte of known radioactivity amount from a calibrated UV absorbance response curve for reference **1**. The standard mass calibration curve for **1** [Y axis = UV area, X axis = mass (ng)] was created from the UV-absorbance at 254 nm from HPLC analyses of solutions of **1** with known dilutions. For a molar activity determination, [^{11}C]**1** from the labeling reaction was purified by HPLC and collected.

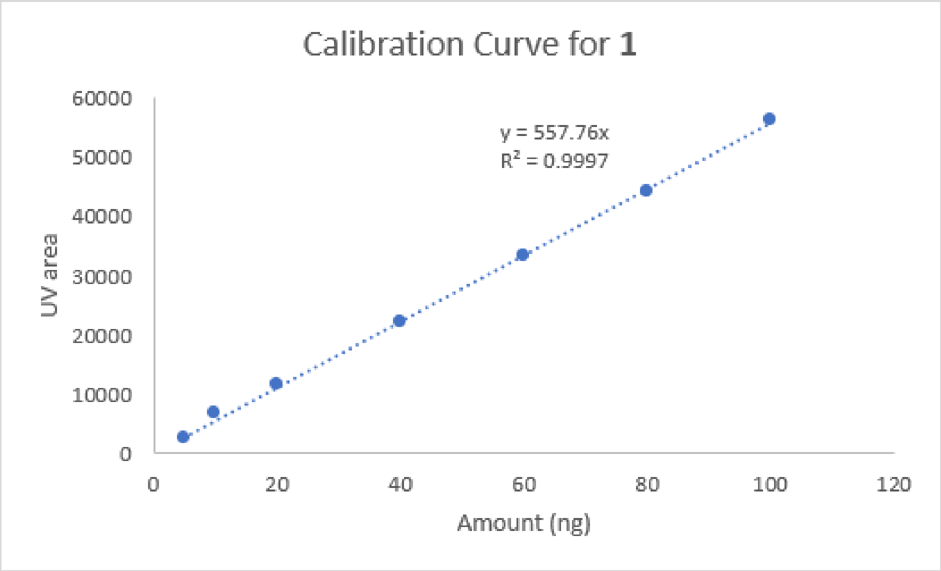

Amount (ng)	UV area
5	2465
10	6645
20	11714
40	22196
60	33386
80	44140
100	56051

Supplementary Figure 23. Calibration curve for 2-(2,2,2-trifluoroethoxy)pyrimidine **1** to determine molar activity.

A known activity of radiochemically pure [^{11}C]**1** was analyzed with HPLC using the same conditions used for creating the calibration curve. The standard curve was used to calculate mass and moles of carrier in [^{11}C]**1**. Starting with about 10 GBq of cyclotron-produced [^{11}C]methane, produced from a 10 μA × 10 min cyclotron irradiation, [^{11}C]**1** was obtained with a molar activity of 60 GBq/μmol, corrected to the end of radionuclide production.

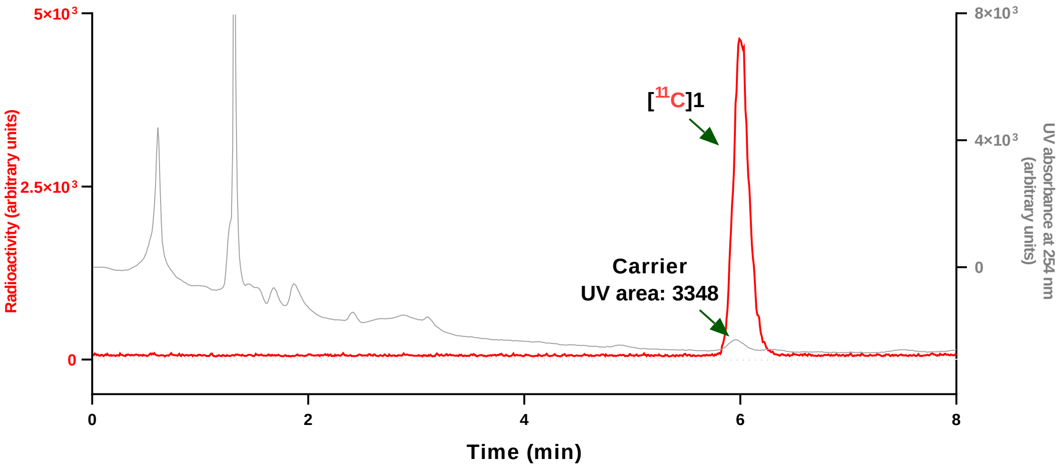

Supplementary Figure 24. Analytical HPLC chromatogram for QC injection of [^{11}C]**1.**

6.6. Determination of the molar activity for [^{18}F]1.

A known activity of radiochemically pure [^{18}F]**1** was analyzed with HPLC using the same conditions used for creating the calibration curve (Supplementary Figure 24). The standard curve was used to calculate mass and moles of carrier in [^{18}F]**1**. Starting with about 200 mCi [^{18}F]Fluoride ion, [^{18}F]**1** was obtained with a molar activity of 1.3 GBq/μmol, corrected to the end of radionuclide production. The molar activity is consistent with the literature reported.[14]

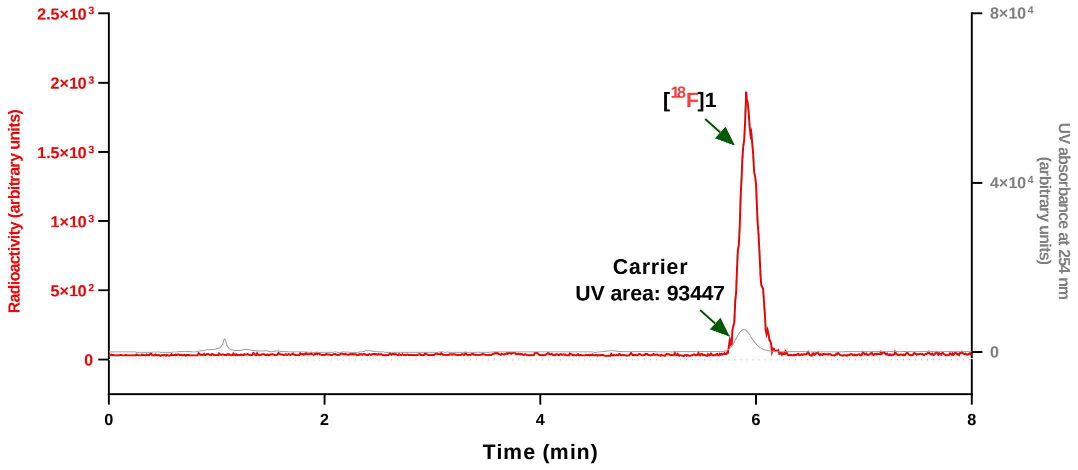

Supplementary Figure 25. Analytical HPLC chromatogram for QC injection of [^{18}F]**1.**

7. HPLC analysis of ^{11}C- and ^{18}F- labeled products.

7.1. HPLC analysis of ^{11}C-labeled compounds.

HPLC analysis of [^{11}C]1

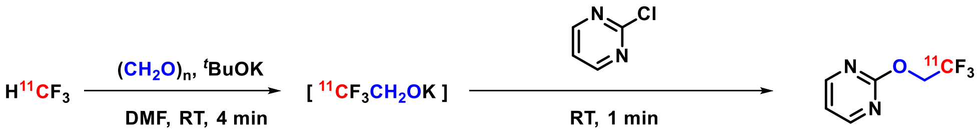

[^{11}C]1

Prepared following general procedure B and analyzed using HPLC condition C.

HPLC condition C

Column: Luna C18(2) (10 μm, 250 × 4.6 mm; i.d. Phenomenex)

Solvent A: H_2O, Solvent B: MeCN

Flow rate = 2 mL/min

0–1 min: 30% B

1–15 min: 30% to 80% B

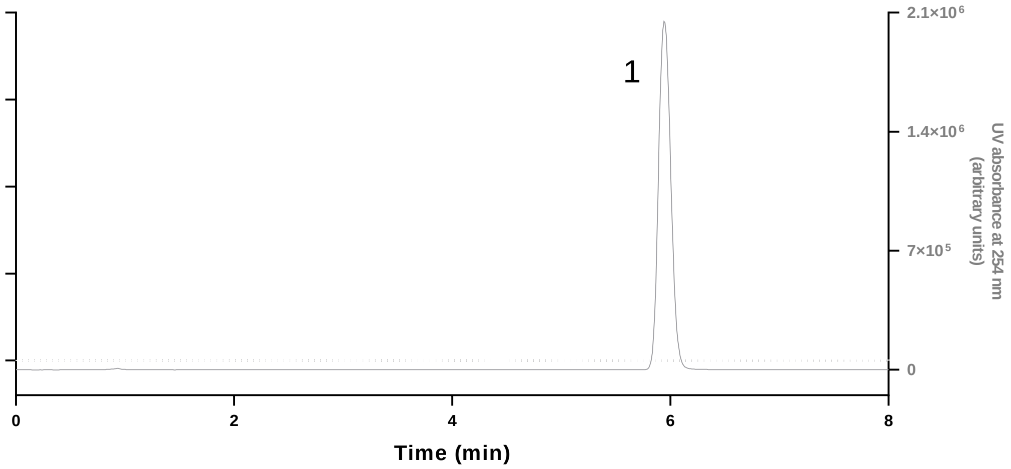

Supplementary Figure 26. Analytical HPLC chromatogram for **1.**

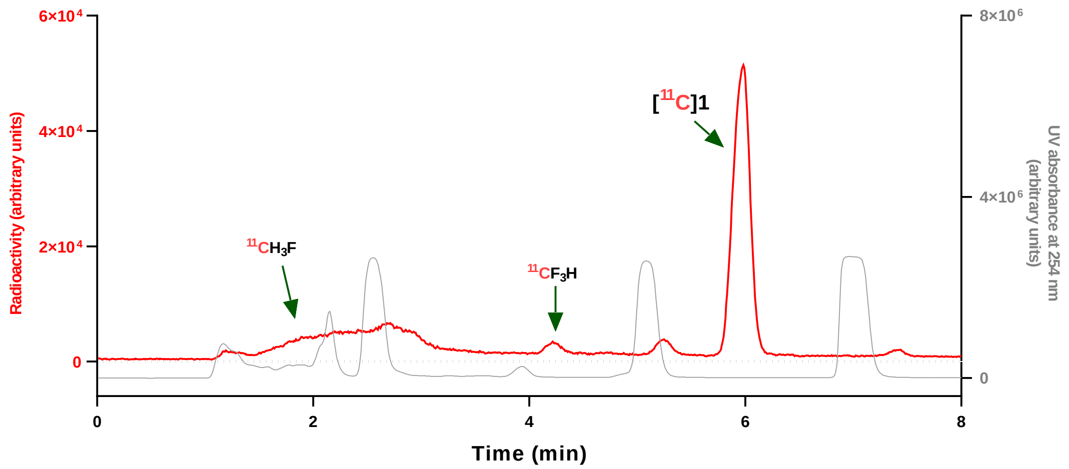

Supplementary Figure 27. Analytical HPLC chromatogram for reaction mixture.

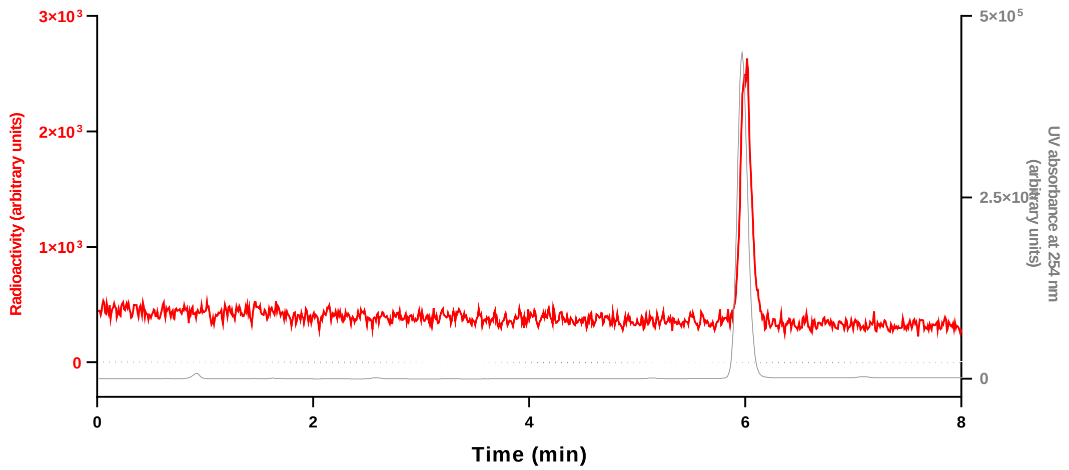

Supplementary Figure 28. Analytical HPLC chromatogram for [^{11}C]**1** collected from the above HPLC analysis when co–injected with **1.**

Run	Retention time (min)	Yield (decay-corrected) (%)	Mean yield ± SD (%)	Isolated yield (%)
1	5.607	88	90 ± 2	83
2	5.606	89		
3	5.701	92		

HPLC analysis of [^{11}C]2

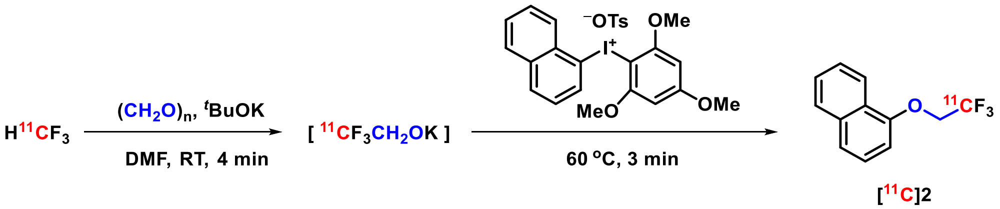

Prepared following general procedure B and analyzed using HPLC condition A.

HPLC condition A

Column: Luna C18(2) (10 μm, 250 × 4.6 mm; i.d. Phenomenex)

Solvent A: H_2O, Solvent B: MeCN

Flow rate: 2 mL/min

0–1 min: 40% B

1–3 min: 40% to 60% B

3–6 min: 60% to 95% B

6–12 min: 95% B

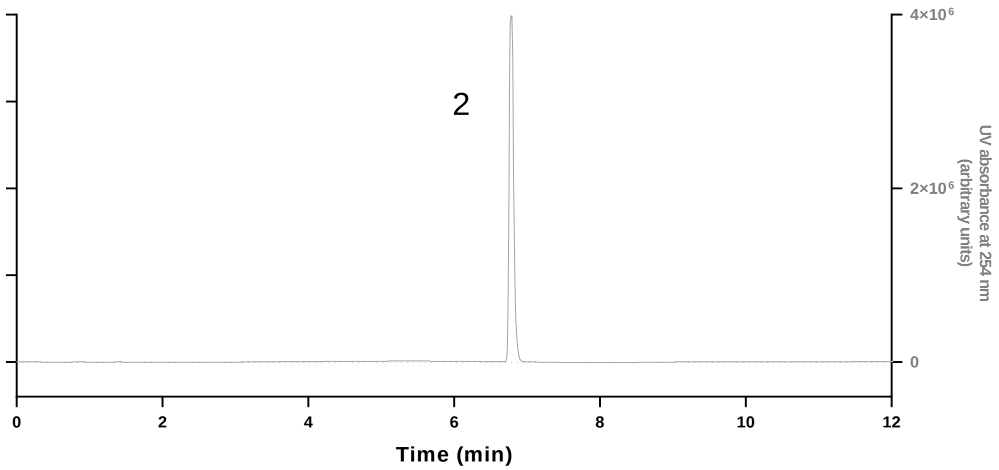

Supplementary Figure 29. Analytical HPLC chromatogram for **2.**

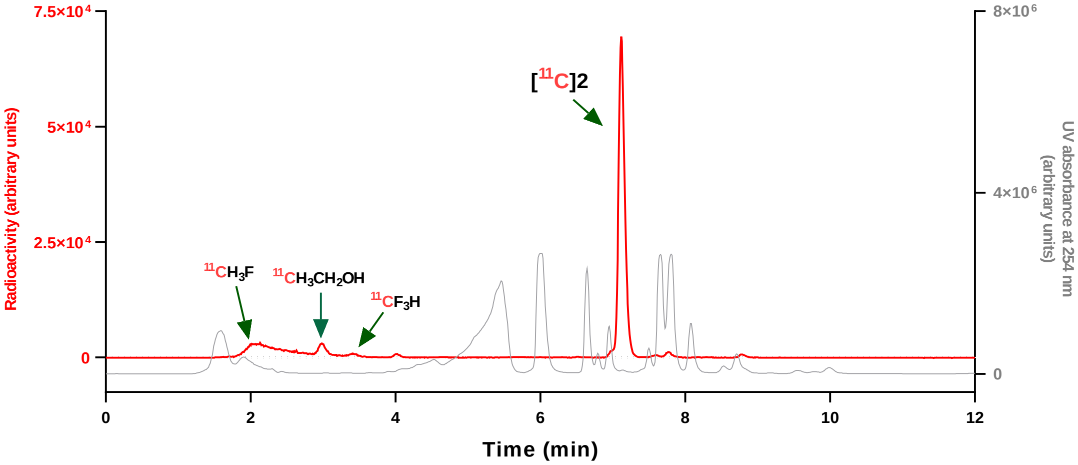

Supplementary Figure 30. Analytical HPLC chromatogram for reaction mixture.

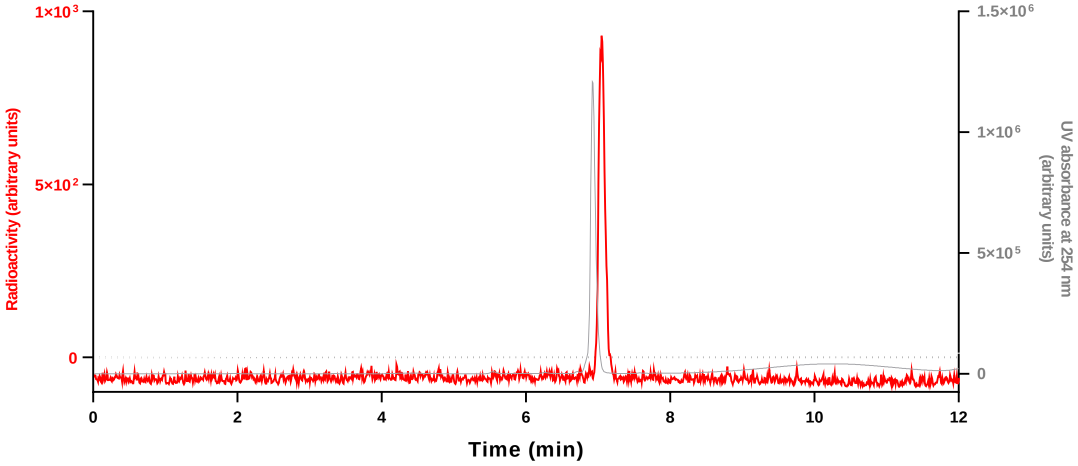

Supplementary Figure 31. Analytical HPLC chromatogram for [^{11}C]**2** collected from the above HPLC analysis when co–injected with **2.**

Run	Retention time (min)	Yield (decay-corrected) (%)	Mean yield ± SD (%)	Isolated yield (%)
1	7.088	89	87 ± 4	73
2	7.118	89		
3	7.023	83		

HPLC analysis of [^{11}C]3

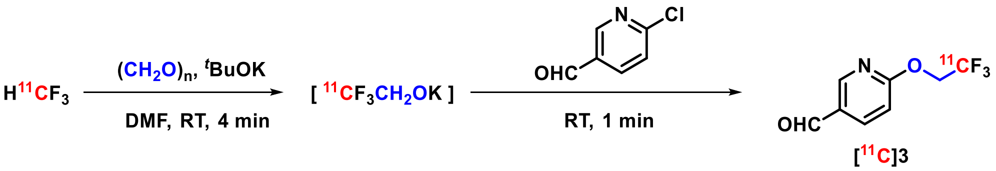

Prepared following general procedure B and analyzed using HPLC condition B.

HPLC condition B

Column: Luna C18(2) (10 μm, 250 × 4.6 mm; i.d. Phenomenex)

Solvent A: H_2O, Solvent B: MeCN

Flow rate = 2 mL/min

0–1 min: 30% B

1–5 min: 30% to 50% B

5–10 min: 50% to 90% B

10–12 min: 90% B

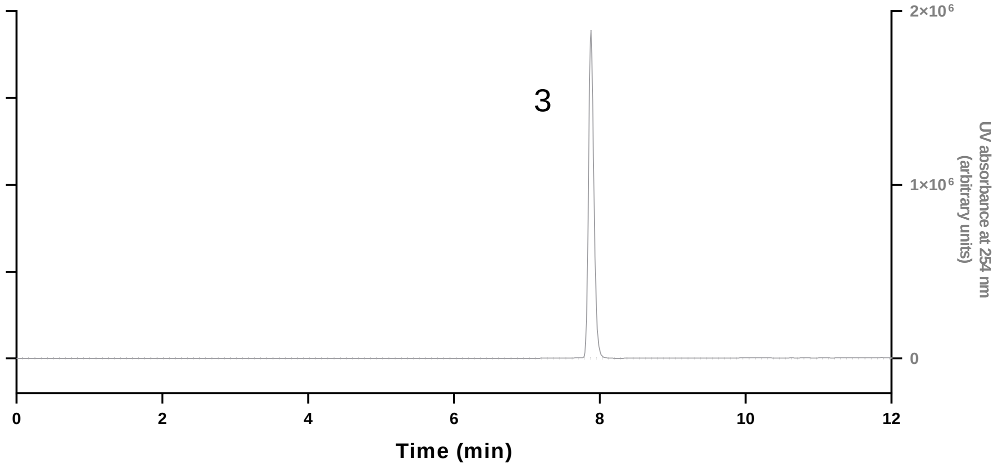

Supplementary Figure 32. Analytical HPLC chromatogram for **3.**

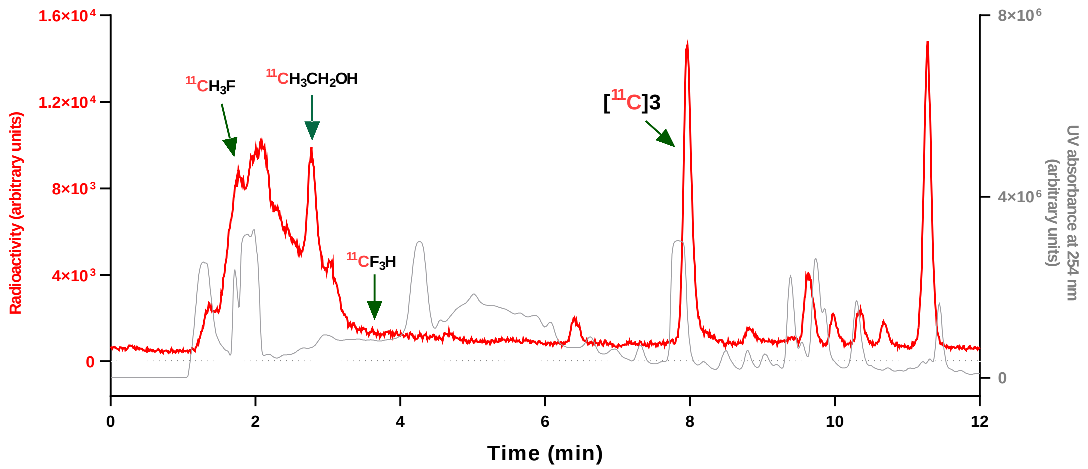

Supplementary Figure 33. Analytical HPLC chromatogram for reaction mixture.

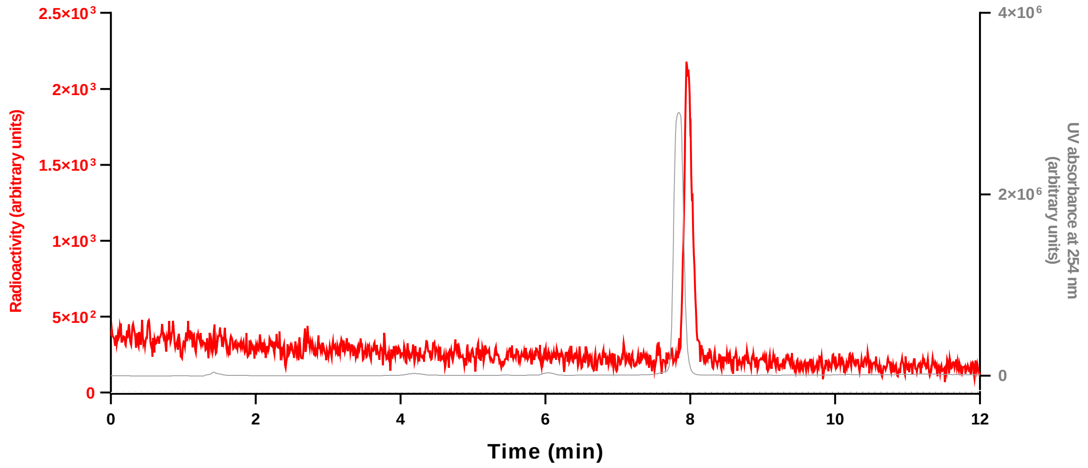

Supplementary Figure 34. Analytical HPLC chromatogram for [^{11}C]**3** collected from the above HPLC analysis when co–injected with **3.**

Run	Retention time (min)	Yield (decay-corrected) (%)	Mean yield ± SD (%)	Isolated yield (%)
1	7.966	20	28 ± 9	22
2	7.967	37		
3	7.964	26		

HPLC analysis of [^{11}C]4

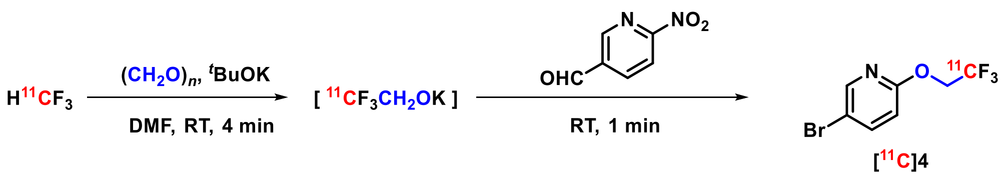

Prepared following general procedure B and analyzed using HPLC condition A.

HPLC condition A

Column: Luna C18(2) (10 μm, 250 × 4.6 mm; i.d. Phenomenex)

Solvent A: H_2O, Solvent B: MeCN

Flow rate: 2 mL/min

0–1 min: 40% B

1–3 min: 40% to 60% B

3–6 min: 60% to 95% B

6–12 min: 95% B

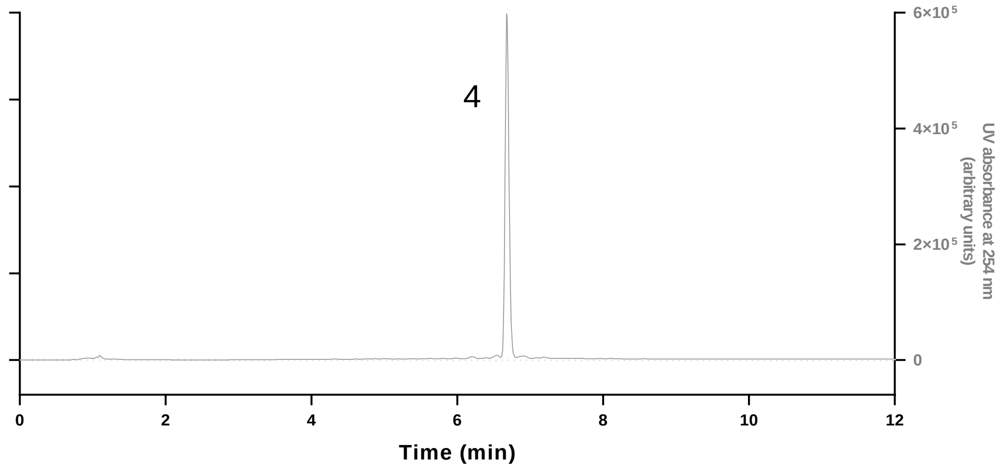

Supplementary Figure 35. Analytical HPLC chromatogram for **4.**

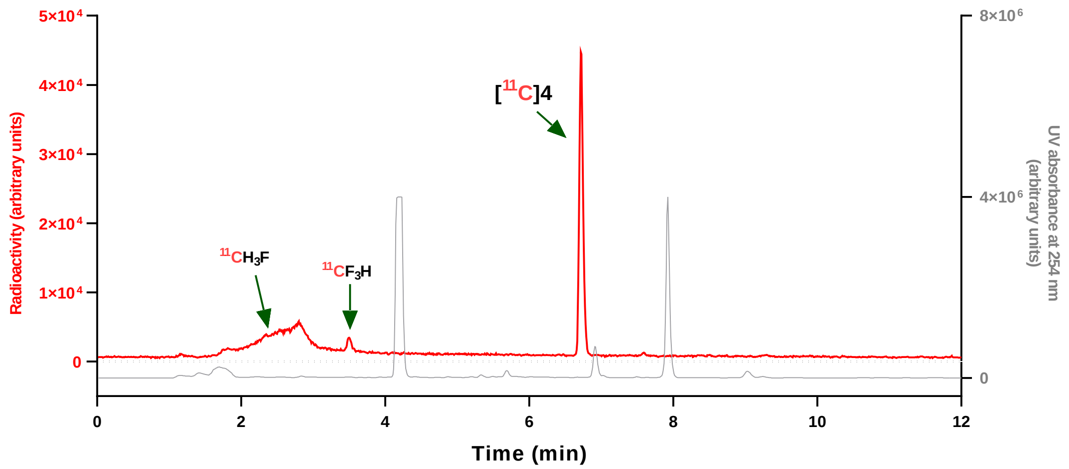

Supplementary Figure 36. Analytical HPLC chromatogram for reaction mixture.

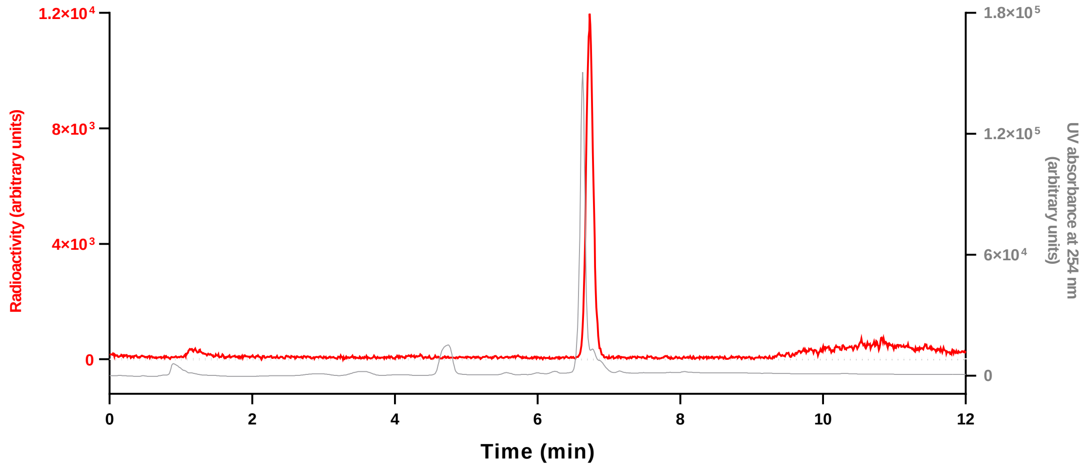

Supplementary Figure 37. Analytical HPLC chromatogram for [^{11}C]**4** collected from the above HPLC analysis when co–injected with **4.**

Run	Retention time (min)	Yield (decay corrected) (%)	Mean yield ± SD (%)	Isolated yield (%)
1	6.727	92	94 ± 2	86
2	6.716	95		
3	6.720	95		

HPLC analysis of [^{11}C]5

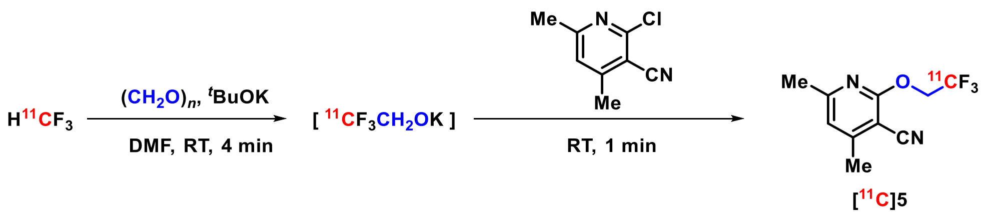

Prepared following general procedure B and analyzed using HPLC condition B.

HPLC condition B

Column: Luna C18(2) (10 μm, 250 × 4.6 mm; i.d. Phenomenex)

Solvent A: H_2O, Solvent B: MeCN

Flow rate = 2 mL/min

0–1 min: 30% B

1–5 min: 30% to 50% B

5–10 min: 50% to 90% B

10–12 min: 90% B

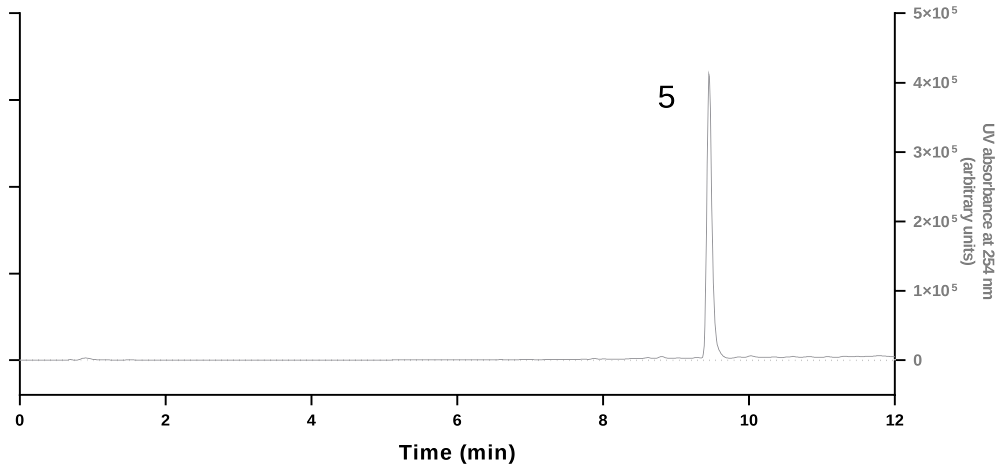

Supplementary Figure 38. Analytical HPLC chromatogram for **5.**

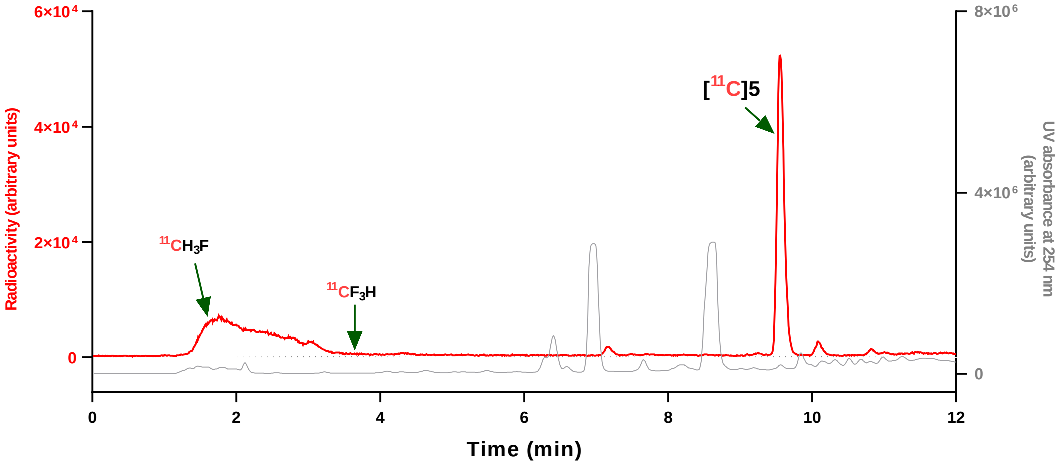

Supplementary Figure 39. Analytical HPLC chromatogram for reaction mixture.

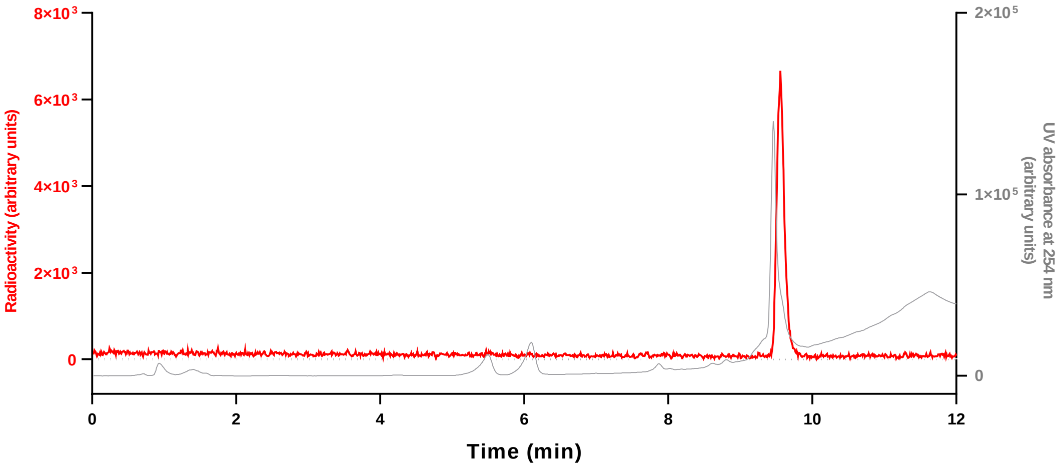

Supplementary Figure 40. Analytical HPLC chromatogram for [^{11}C]**5** collected from the above HPLC analysis when co–injected with **5.**

Run	Retention time (min)	Yield (decay corrected) (%)	Mean yield ± SD (%)	Isolated yield (%)
1	9.564	81	86 ± 5	77
2	9.577	88		
3	9.555	90		

HPLC analysis of [^{11}C]6

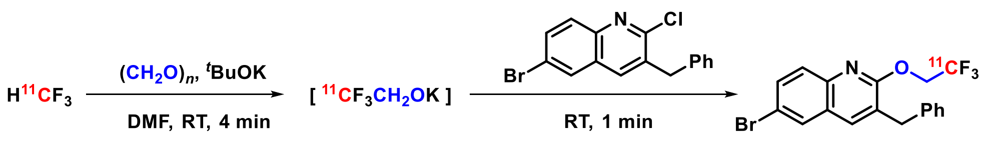

Prepared following general procedure B and analyzed using HPLC condition A.

HPLC condition A

Column: Luna C18(2) (10 μm, 250 × 4.6 mm; i.d. Phenomenex)

Solvent A: H_2O, Solvent B: MeCN

Flow rate: 2 mL/min

0–1 min: 40% B

1–3 min: 40% to 60% B

3–6 min: 60% to 95% B

6–12 min: 95% B

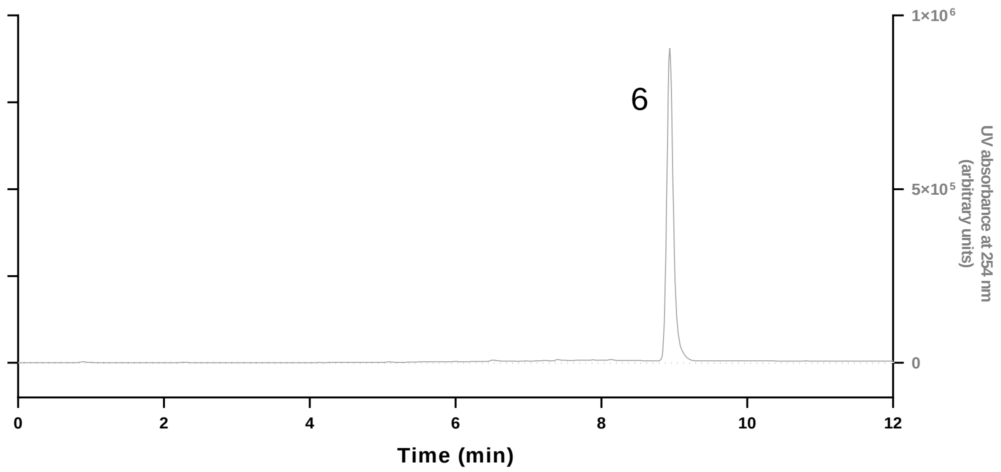

Supplementary Figure 41. Analytical HPLC chromatogram for **6.**

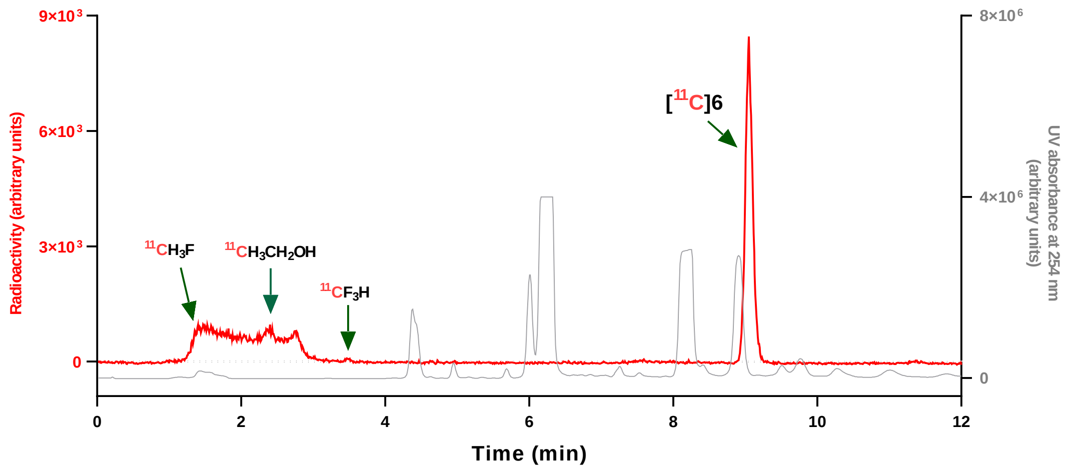

Supplementary Figure 42. Analytical HPLC chromatogram for reaction mixture.

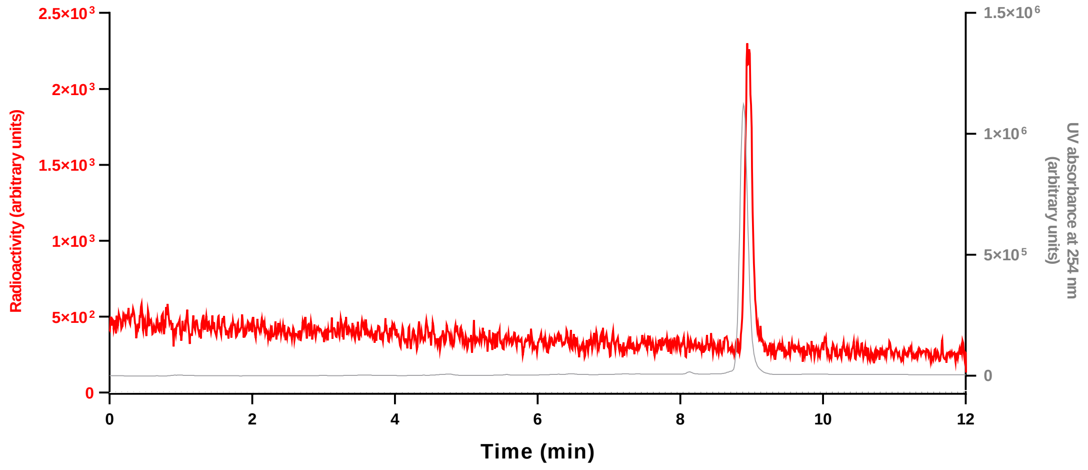

Supplementary Figure 43. Analytical HPLC chromatogram for [^{11}C]**6** collected from the above HPLC analysis when co–injected with **6.**

Run	Retention time (min)	Yield (decay-corrected) (%)	Mean yield ± SD (%)	Isolated yield (%)
1	8.966	88	87 ± 1	86
2	9.057	87		
3	9.049	87		

HPLC analysis of [^{11}C]7

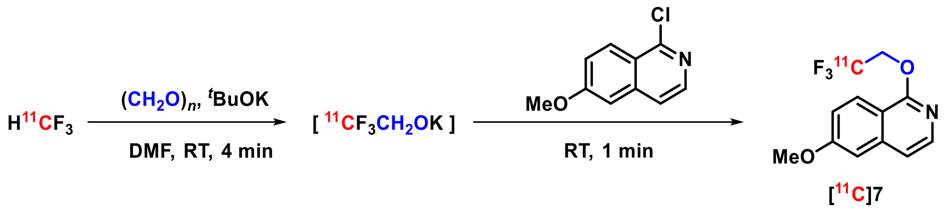

Prepared following general procedure B and analyzed using HPLC condition A.

HPLC condition A

Column: Luna C18(2) (10 μm, 250 × 4.6 mm; i.d. Phenomenex)

Solvent A: H_2O, Solvent B: MeCN

Flow rate: 2 mL/min

0–1 min: 40% B

1–3 min: 40% to 60% B

3–6 min: 60% to 95% B

6–12 min: 95% B

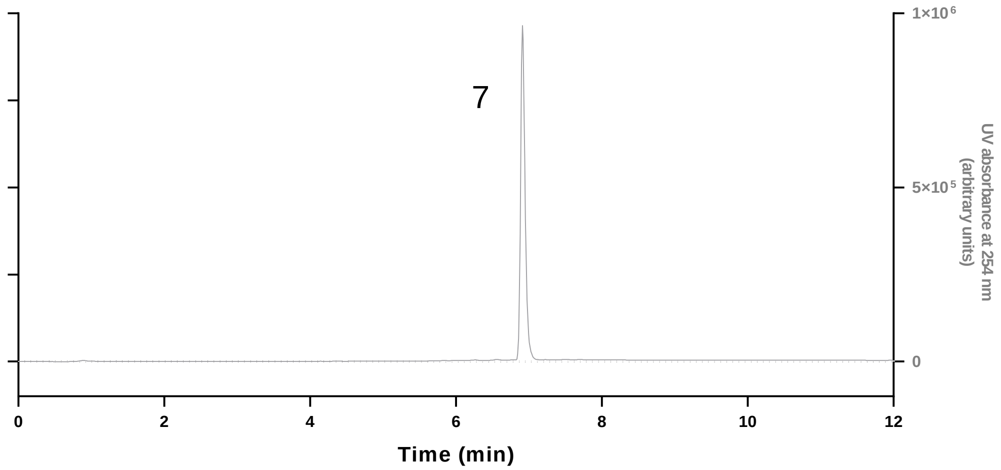

Supplementary Figure 44. Analytical HPLC chromatogram for **7.**

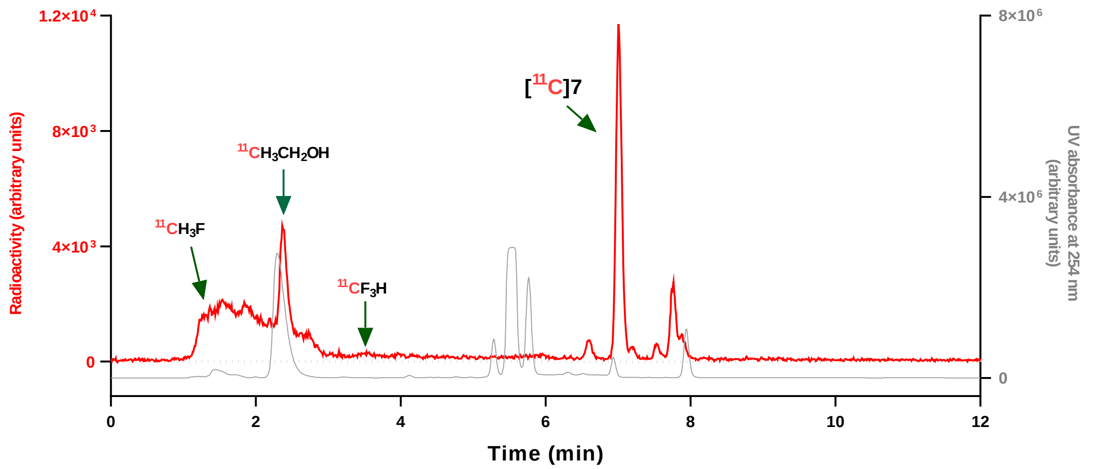

Supplementary Figure 45. Analytical HPLC chromatogram for reaction mixture.

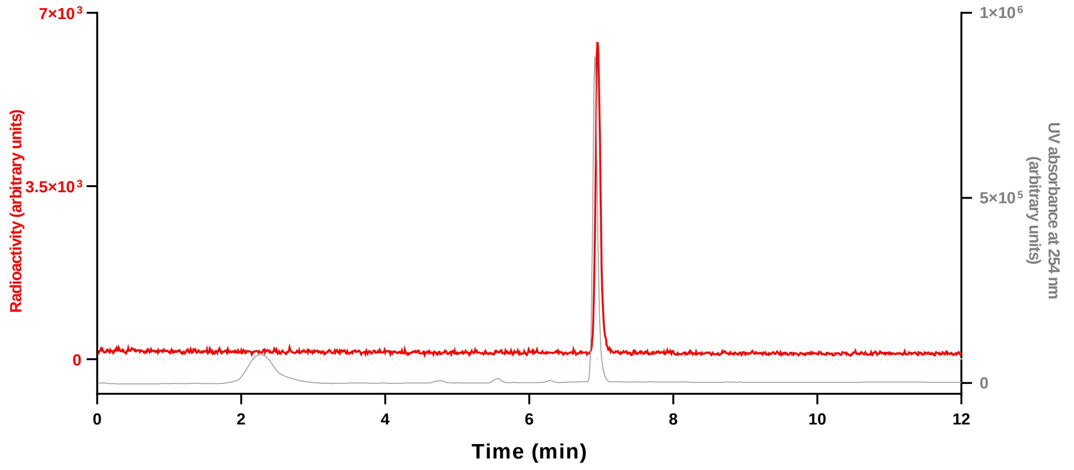

Supplementary Figure 46. Analytical HPLC chromatogram for [^{11}C]**7** collected from the above HPLC analysis when co–injected with **7.**

Run	Retention time (min)	Yield (decay-corrected) (%)	Mean yield ± SD (%)	Isolated yield (%)
1	7.011	41	45 ± 5	31
2	7.013	44		
3	7.009	50		

HPLC analysis of [^{11}C]8

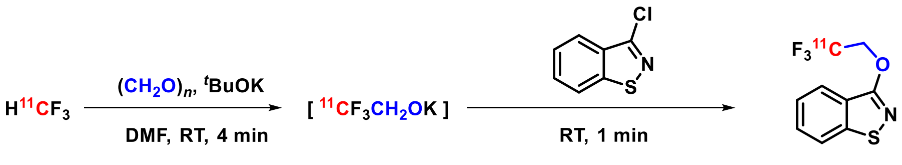

Prepared following general procedure B and analyzed using HPLC condition A.

HPLC condition A

Column: Luna C18(2) (10 μm, 250 × 4.6 mm; i.d. Phenomenex)

Solvent A: H_2O, Solvent B: MeCN

Flow rate: 2 mL/min

0–1 min: 40% B

1–3 min: 40% to 60% B

3–6 min: 60% to 95% B

6–12 min: 95% B

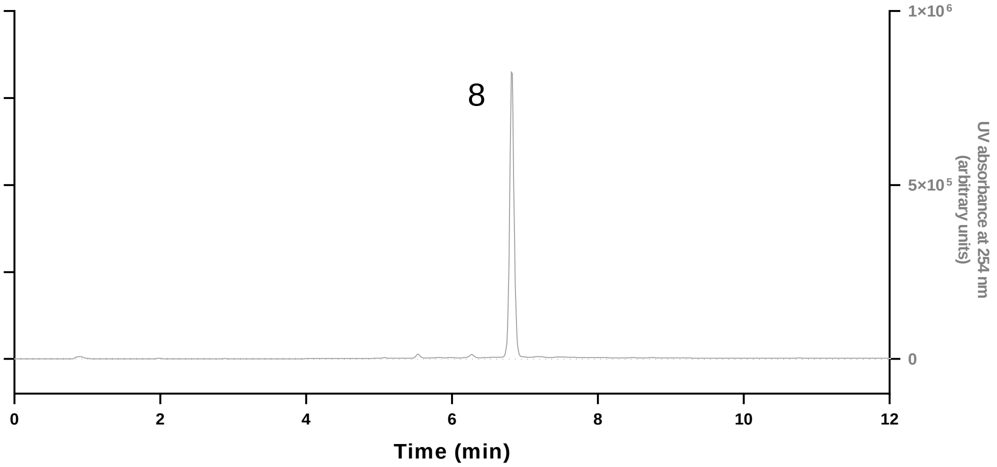

Supplementary Figure 47. Analytical HPLC chromatogram for **8.**

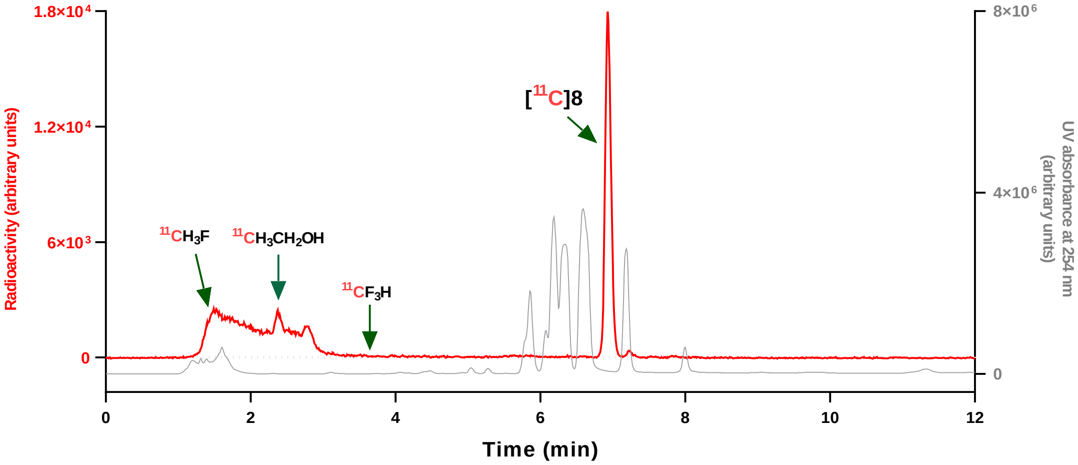

Supplementary Figure 48. Analytical HPLC chromatogram for reaction mixture.

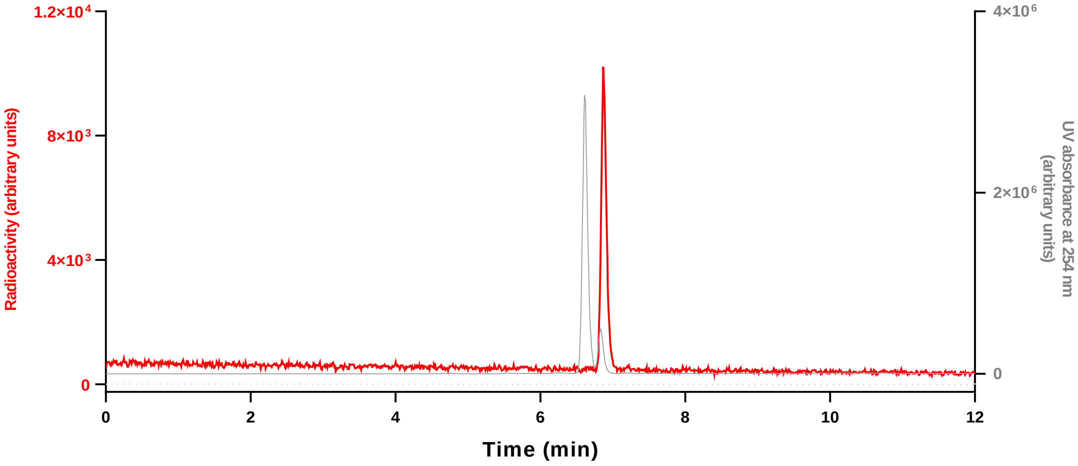

Supplementary Figure 49. Analytical HPLC chromatogram for [^{11}C]**8** collected from the above HPLC analysis when co–injected with **8.**

Run	Retention time (min)	Yield (decay-corrected) (%)	Mean yield ± SD (%)	Isolated yield (%)
1	6.891	78	80 ± 5	82
2	6.932	85		
3	6.940	76		

HPLC analysis of [^{11}C]9

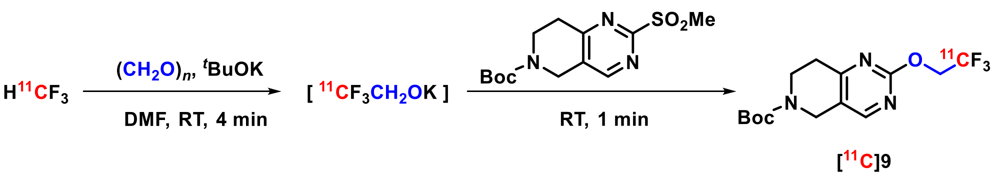

Prepared following general procedure B and analyzed using HPLC condition A.

HPLC condition A

Column: Luna C18(2) (10 μm, 250 × 4.6 mm; i.d. Phenomenex)

Solvent A: H_2O, Solvent B: MeCN

Flow rate: 2 mL/min

0–1 min: 40% B

1–3 min: 40% to 60% B

3–6 min: 60% to 95% B

6–12 min: 95% B

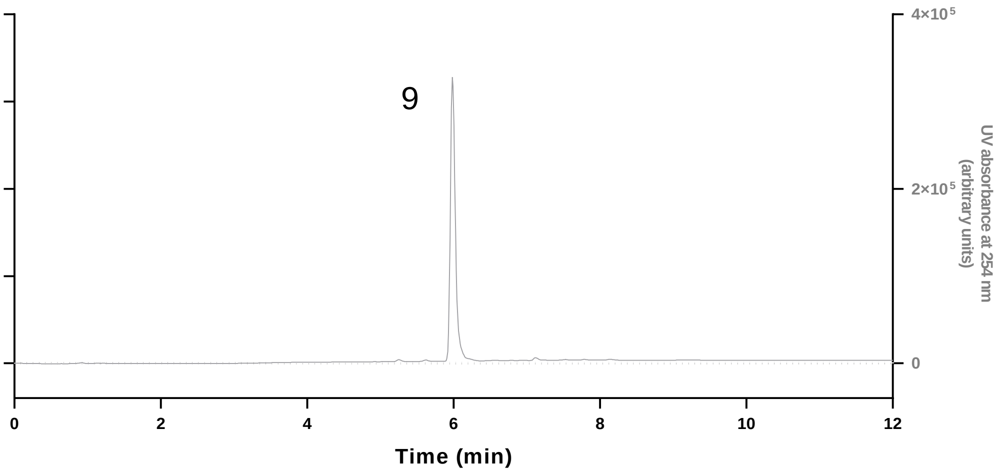

Supplementary Figure 50. Analytical HPLC chromatogram for **9.**

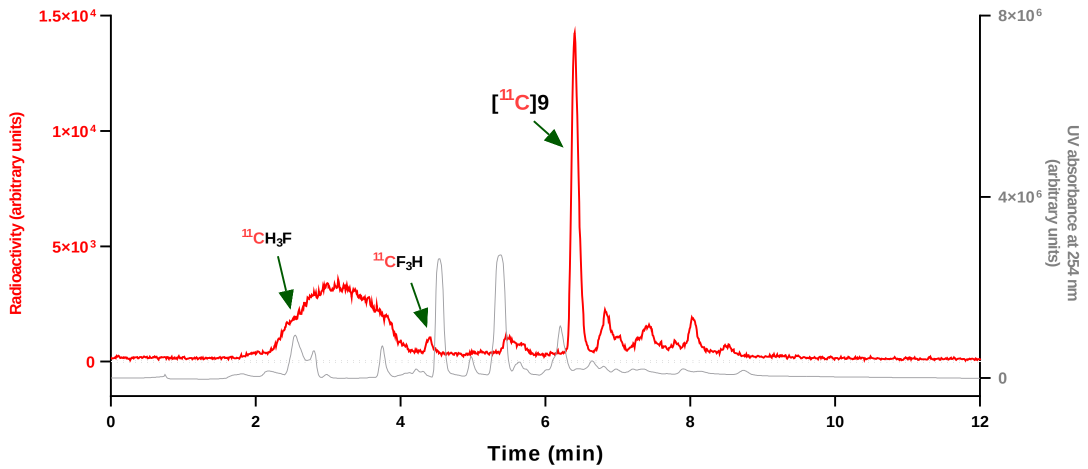

Supplementary Figure 51. Analytical HPLC chromatogram for reaction mixture.

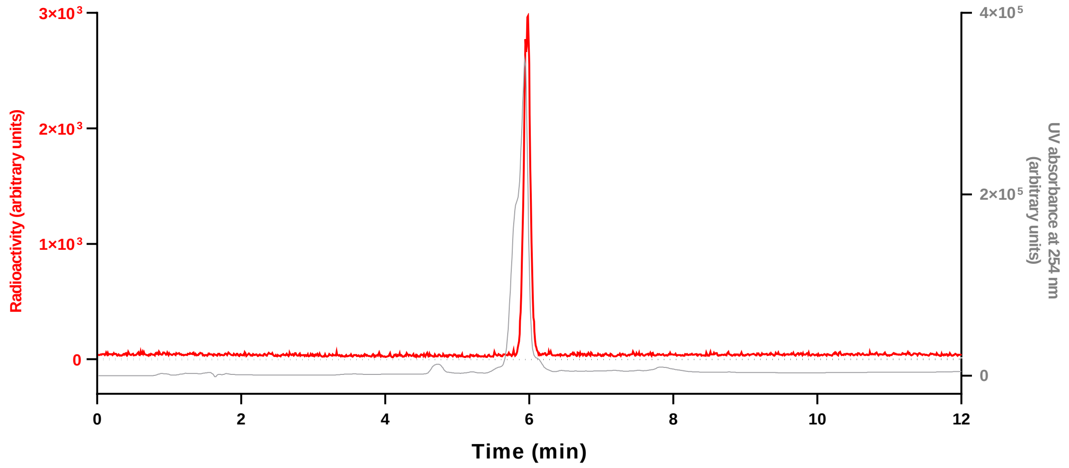

Supplementary Figure 52. Analytical HPLC chromatogram for [^{11}C]**9** collected from the above HPLC analysis when co–injected with **9.**

Run	Retention time (min)	Yield (decay-corrected) (%)	Mean yield ± SD (%)	Isolated yield (%)
1	6.442	45	46 ± 2	41
2	6.405	48		
3	6.068	44		

HPLC analysis of [^{11}C]10

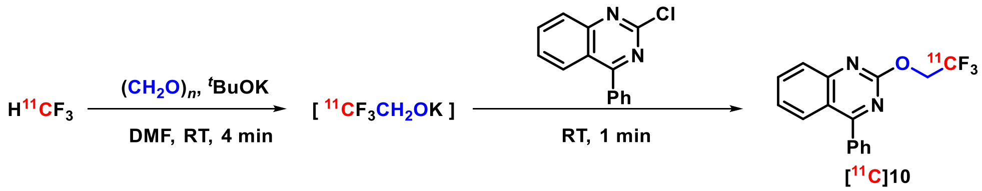

Prepared following general procedure B and analyzed using HPLC condition A.

HPLC condition A

Column: Luna C18(2) (10 μm, 250 × 4.6 mm; i.d. Phenomenex)

Solvent A: H_2O, Solvent B: MeCN

Flow rate: 2 mL/min

0–1 min: 40% B

1–3 min: 40% to 60% B

3–6 min: 60% to 95% B

6–12 min: 95% B

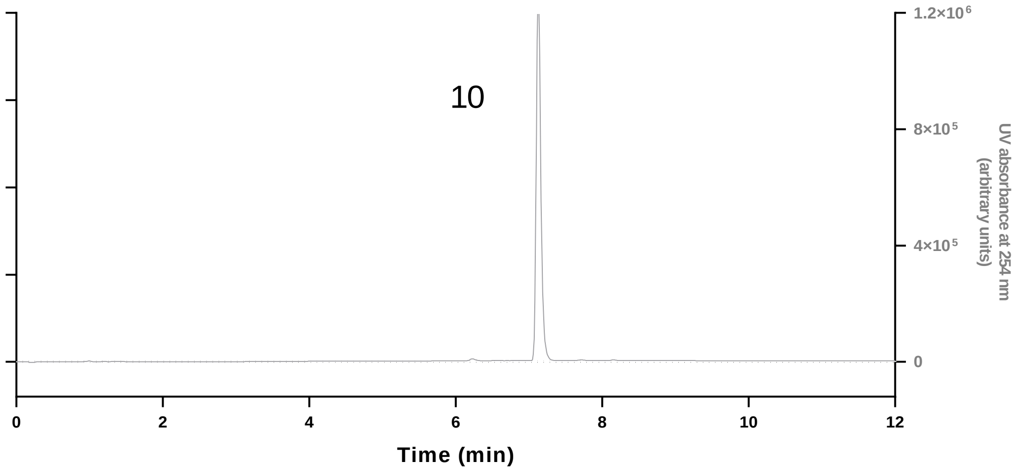

Supplementary Figure 53. Analytical HPLC chromatogram for **10.**

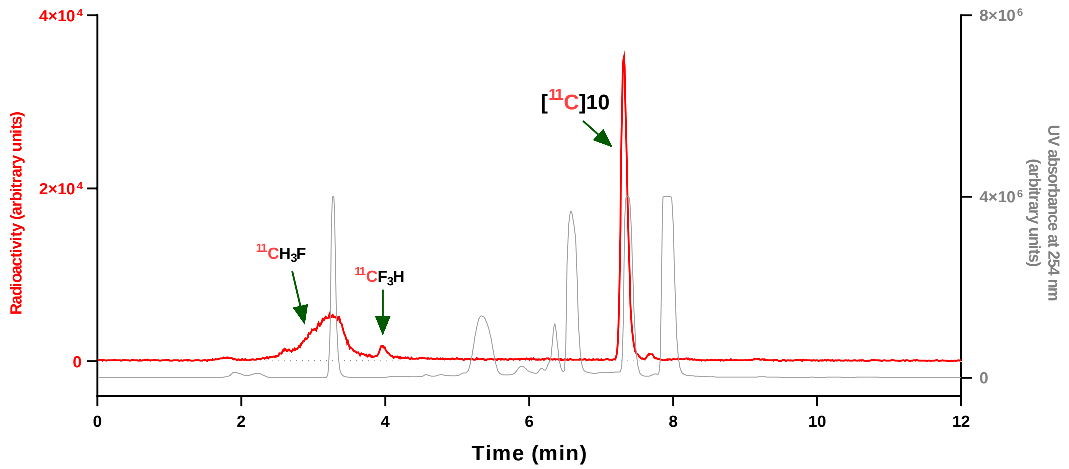

Supplementary Figure 54. Analytical HPLC chromatogram for reaction mixture.

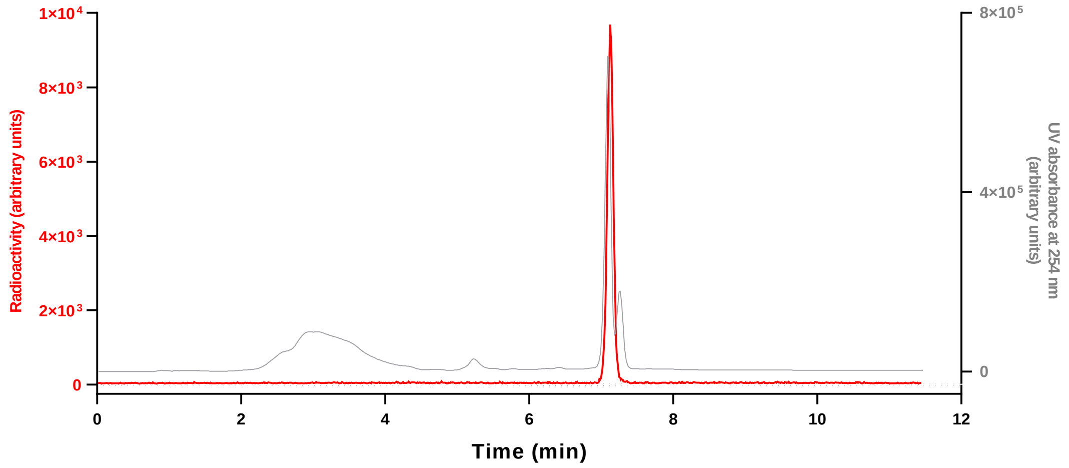

Supplementary Figure 55. Analytical HPLC chromatogram for [^{11}C]**10** collected from the above HPLC analysis when co–injected with **10.**

Run	Retention time (min)	Yield (decay-corrected) (%)	Mean yield ± SD (%)	Isolated yield (%)
1	7.309	88	92 ± 4	77
2	7.314	95		
3	7.381	93		

HPLC analysis of [^{11}C]11

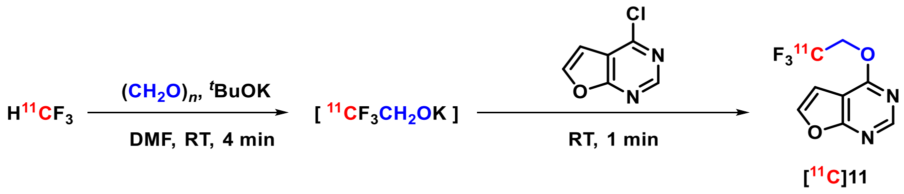

Prepared following general procedure B and analyzed using HPLC condition A.

HPLC condition A

Column: Luna C18(2) (10 μm, 250 × 4.6 mm; i.d. Phenomenex)

Solvent A: H_2O, Solvent B: MeCN

Flow rate: 2 mL/min

0–1 min: 40% B

1–3 min: 40% to 60% B

3–6 min: 60% to 95% B

6–12 min: 95% B

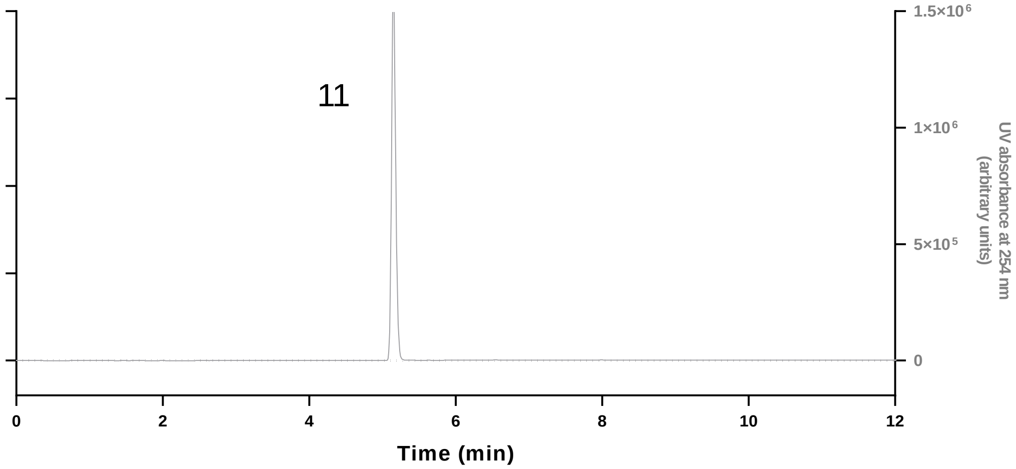

Supplementary Figure 56. Analytical HPLC chromatogram for **11.**

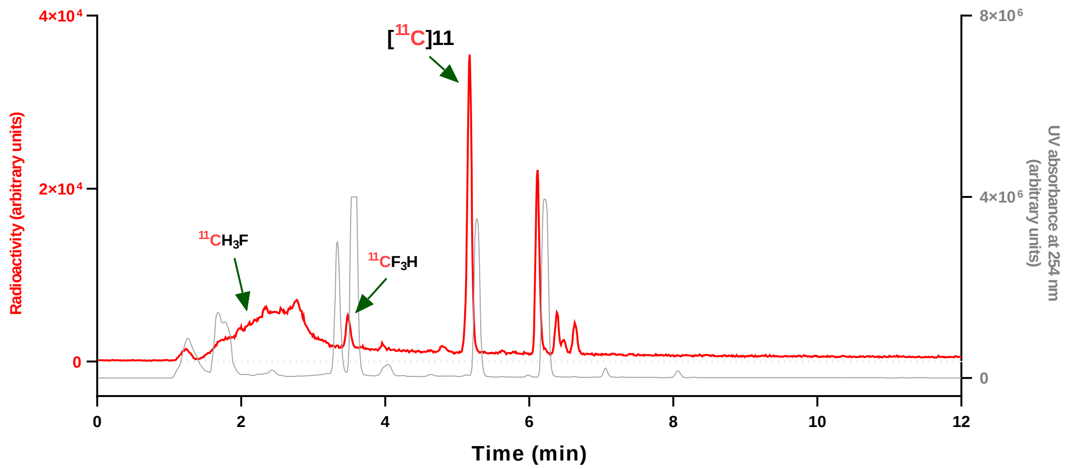

Supplementary Figure 57. Analytical HPLC chromatogram for reaction mixture.

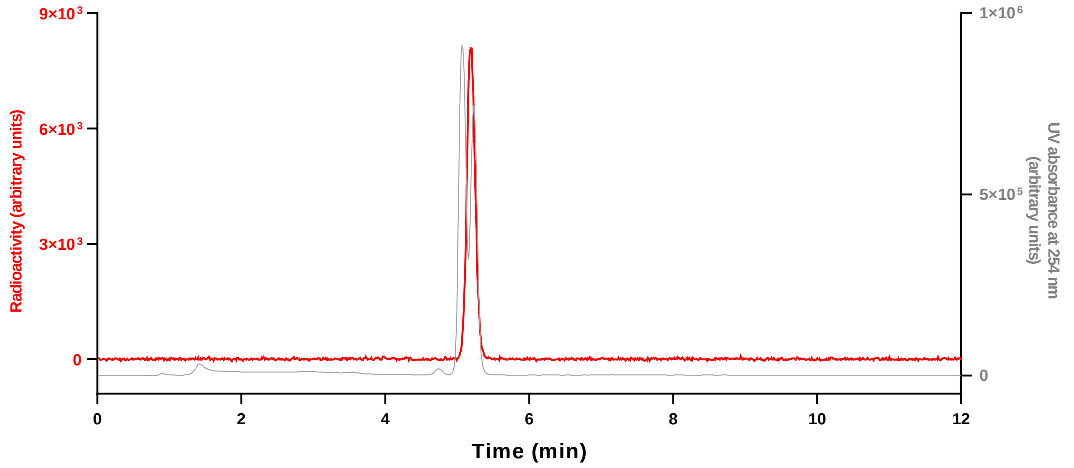

Supplementary Figure 58. Analytical HPLC chromatogram for [^{11}C]**11** collected from the above HPLC analysis when co–injected with **11.**

Run	Retention time (min)	Yield (decay-corrected) (%)	Mean yield ± SD (%)	Isolated yield (%)
1	5.185	28	41 ± 12	41
2	5.172	49		
3	5.173	47		

HPLC analysis of [^{11}C]12

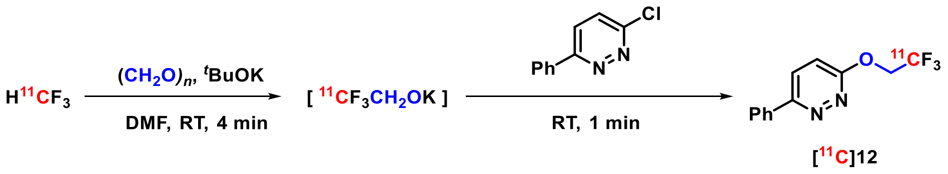

Prepared following general procedure B and analyzed using HPLC condition A.

HPLC condition A

Column: Luna C18(2) (10 μm, 250 × 4.6 mm; i.d. Phenomenex)

Solvent A: H_2O, Solvent B: MeCN

Flow rate: 2 mL/min

0–1 min: 40% B

1–3 min: 40% to 60% B

3–6 min: 60% to 95% B

6–12 min: 95% B

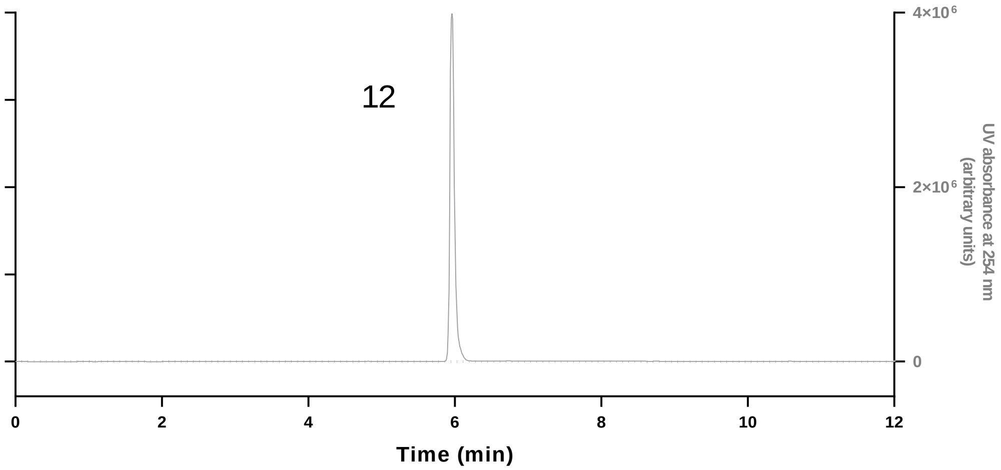

Supplementary Figure 59. Analytical HPLC chromatogram for **12.**

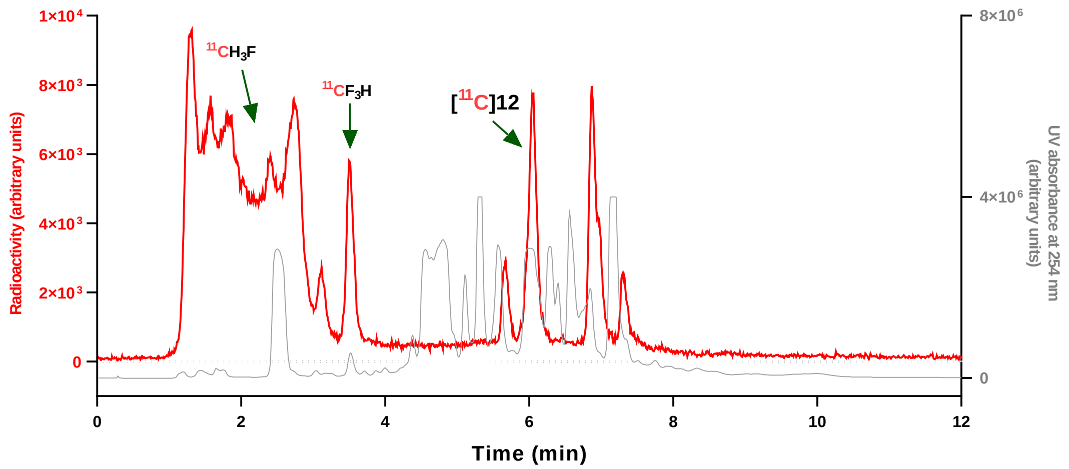

Supplementary Figure 60. Analytical HPLC chromatogram for reaction mixture.

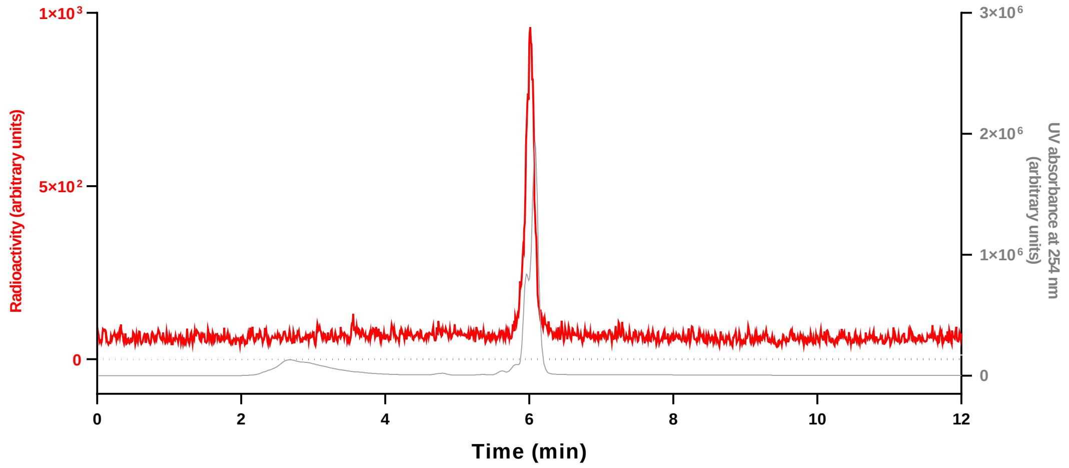

Supplementary Figure 61. Analytical HPLC chromatogram for [^{11}C]**12** collected from the above HPLC analysis when co–injected with **12.**

Run	Retention time (min)	Yield (decay-corrected) (%)	Mean yield ± SD (%)	Isolated yield (%)
1	6.062	21	21 ± 4	20
2	6.056	25		
3	6.057	17		

HPLC analysis of [^{11}C]13

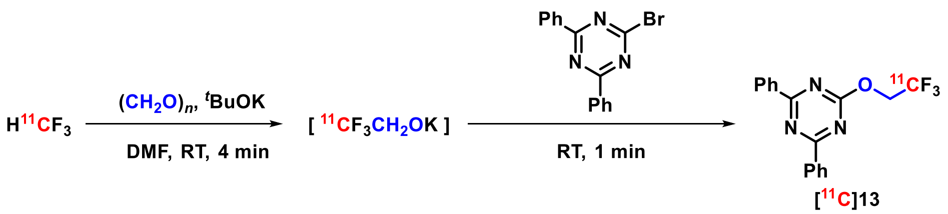

Prepared following general procedure B and analyzed using HPLC condition A.

HPLC condition A

Column: Luna C18(2) (10 μm, 250 × 4.6 mm; i.d. Phenomenex)

Solvent A: H_2O, Solvent B: MeCN

Flow rate: 2 mL/min

0–1 min: 40% B

1–3 min: 40% to 60% B

3–6 min: 60% to 95% B

6–12 min: 95% B

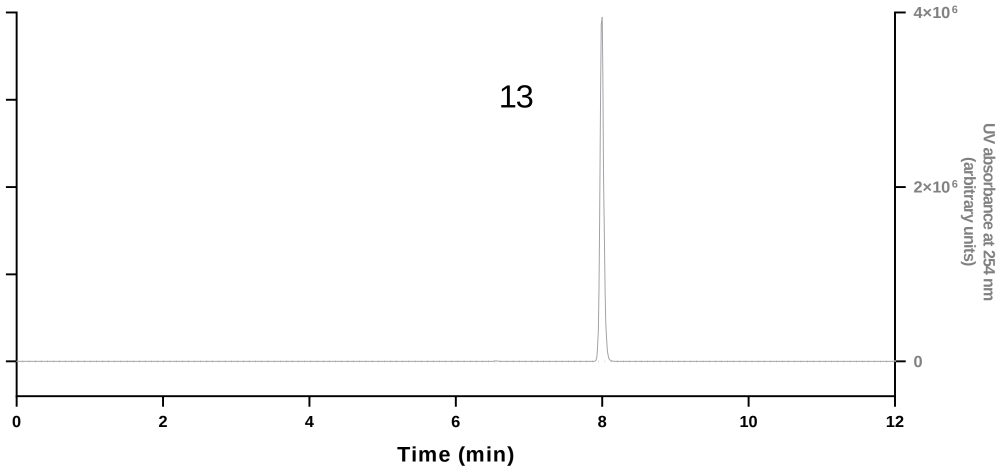

Supplementary Figure 62. Analytical HPLC chromatogram for **13.**

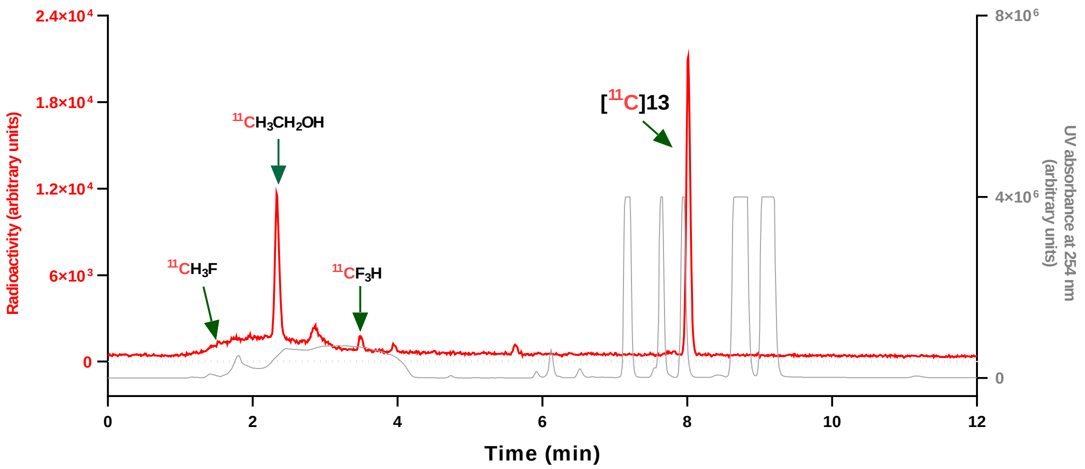

Supplementary Figure 63. Analytical HPLC chromatogram for reaction mixture.

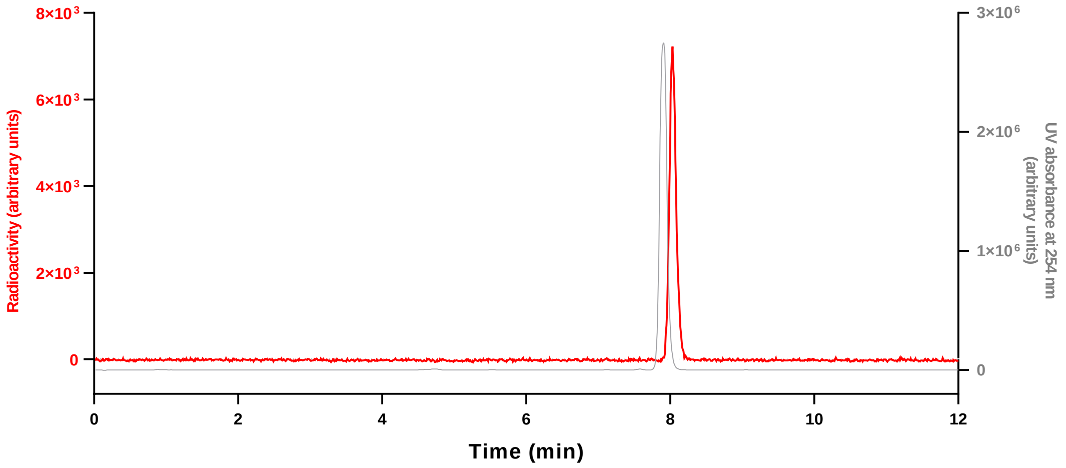

Supplementary Figure 64. Analytical HPLC chromatogram for [11C]**13** collected from the above the HPLC analysis when co–injected with **13.**

Run	Retention time (min)	Yield (decay-corrected) (%)	Mean yield ± SD (%)	Isolated yield (%)
1	8.014	54	53 ± 1	42
2	8.011	54		
3	8.015	52		

HPLC analysis of [^{11}C]14

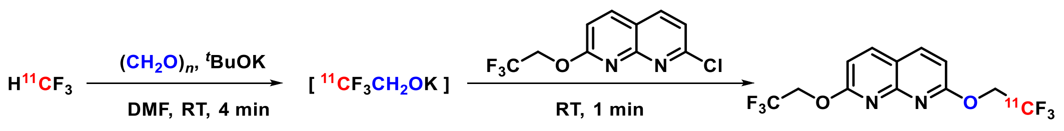

[^{11}C]14

Prepared following general procedure B and analyzed using HPLC condition A.

HPLC condition A

Column: Luna C18(2) (10 μm, 250 × 4.6 mm; i.d. Phenomenex)

Solvent A: H_2O, Solvent B: MeCN

Flow rate: 2 mL/min

0–1 min: 40% B

1–3 min: 40% to 60% B

3–6 min: 60% to 95% B

6–12 min: 95% B

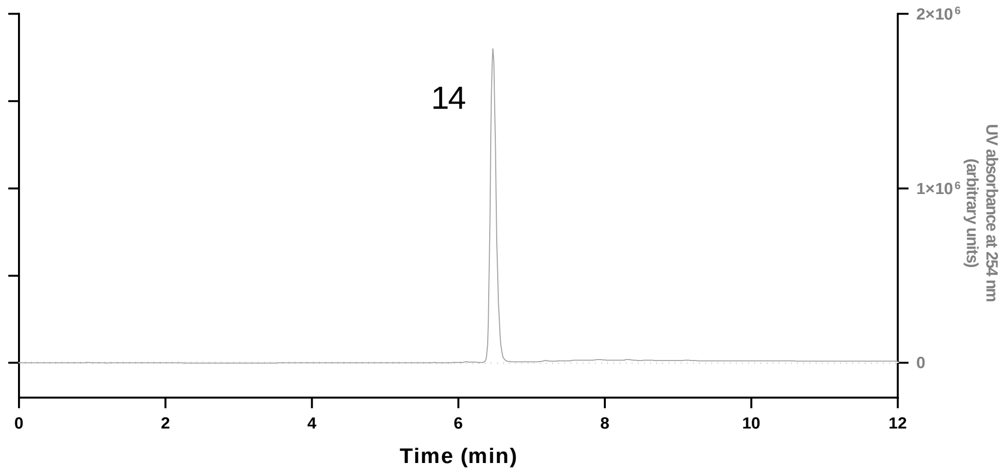

Supplementary Figure 65. Analytical HPLC chromatogram for **14.**

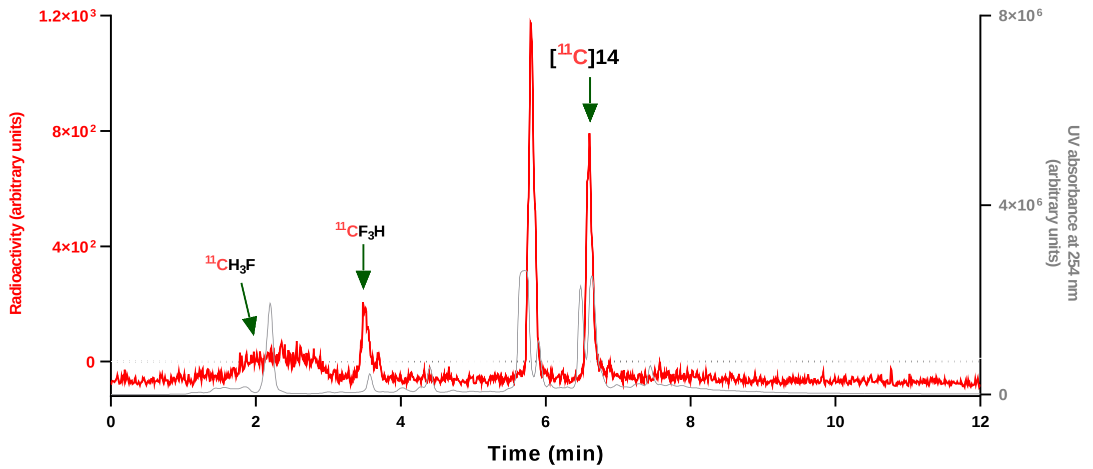

Supplementary Figure 66. Analytical HPLC chromatogram for reaction mixture.

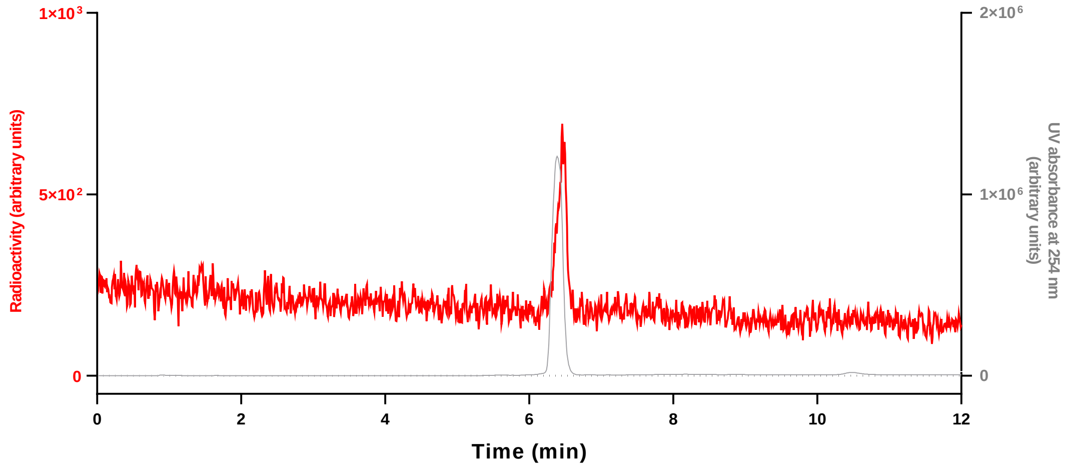

Supplementary Figure 67. Analytical HPLC chromatogram for [^{11}C]**14** collected from the above HPLC analysis when co–injected with **14.**

Run	Retention time (min)	Yield (decay-corrected) (%)	Mean yield ± SD (%)	Isolated yield (%)
1	6.607	37	35 ± 2	29
2	6.608	34		
3	6.499	35		

HPLC analysis of [^{11}C]15

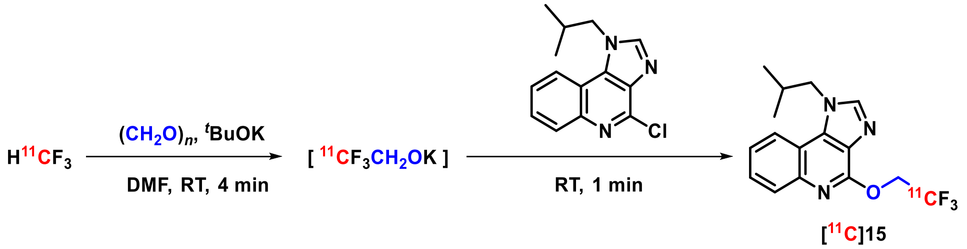

Prepared following general procedure B and analyzed using HPLC condition A.

HPLC condition A

Column: Luna C18(2) (10 μm, 250 × 4.6 mm; i.d. Phenomenex)

Solvent A: H_2O, Solvent B: MeCN

Flow rate: 2 mL/min

0–1 min: 40% B

1–3 min: 40% to 60% B

3–6 min: 60% to 95% B

6–12 min: 95% B

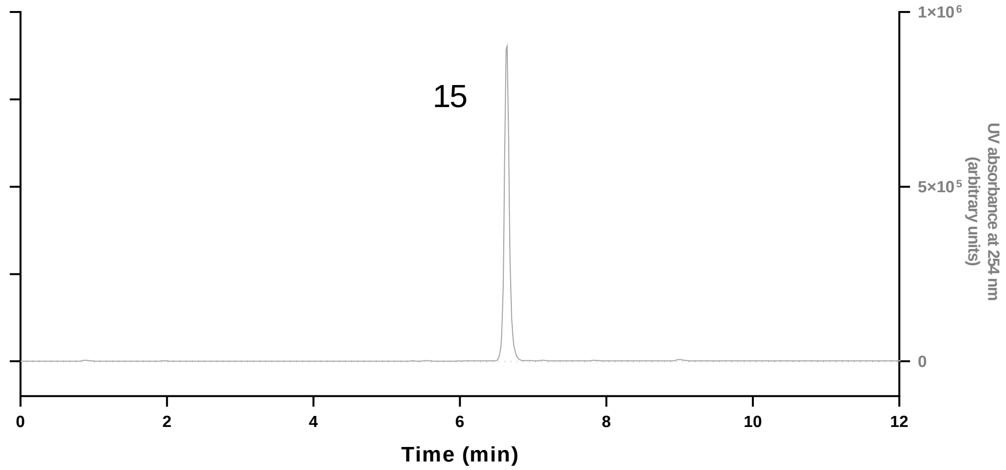

Supplementary Figure 68. Analytical HPLC chromatogram for **15.**

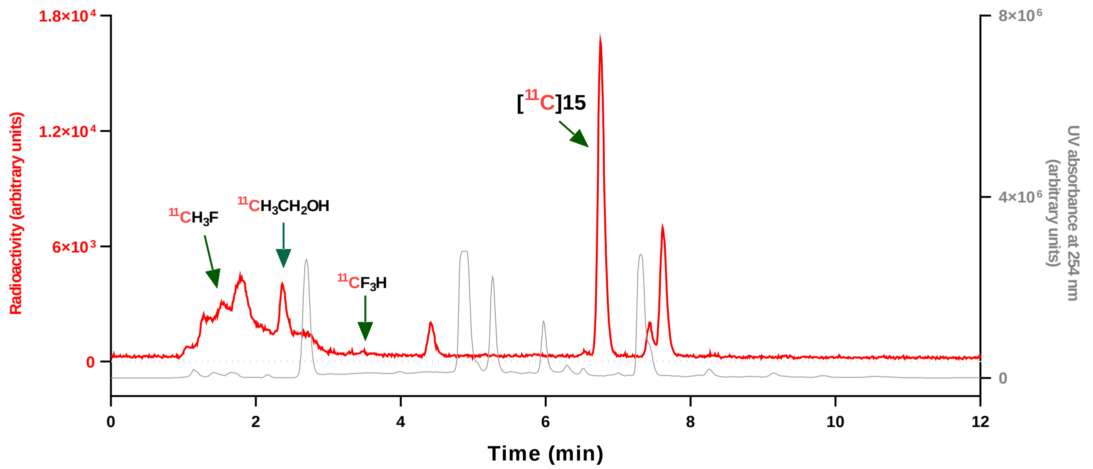

Supplementary Figure 69. Analytical HPLC chromatogram for reaction mixture.

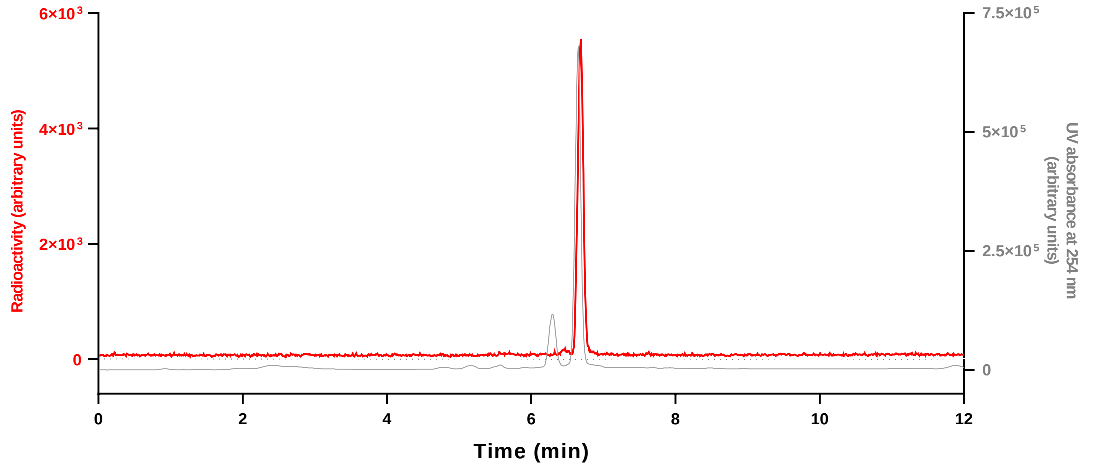

Supplementary Figure 70. Analytical HPLC chromatogram for [^{11}C]**15** collected from the above HPLC analysis when co–injected with **15.**

Run	Retention time (min)	Yield (decay-corrected) (%)	Mean yield ± SD (%)	Isolated yield (%)
1	6.759	37	43 ± 8	33
2	6.763	40		
3	6.761	52		

HPLC analysis of [^{11}C]16

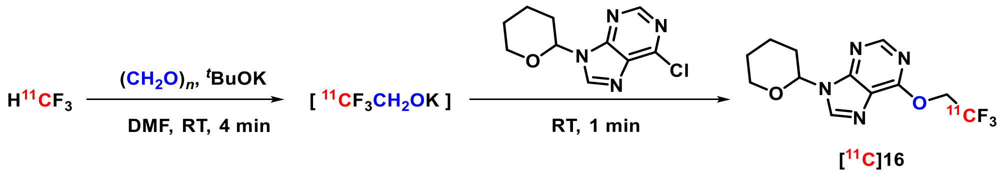

Prepared following general procedure B and analyzed using HPLC condition B.

HPLC condition B

Column: Luna C18(2) (10 μm, 250 × 4.6 mm; i.d. Phenomenex)

Solvent A: H_2O, Solvent B: MeCN

Flow rate = 2 mL/min

0–1 min: 30% B

1–5 min: 30% to 50% B

5–10 min: 50% to 90% B

10–12 min: 90% B

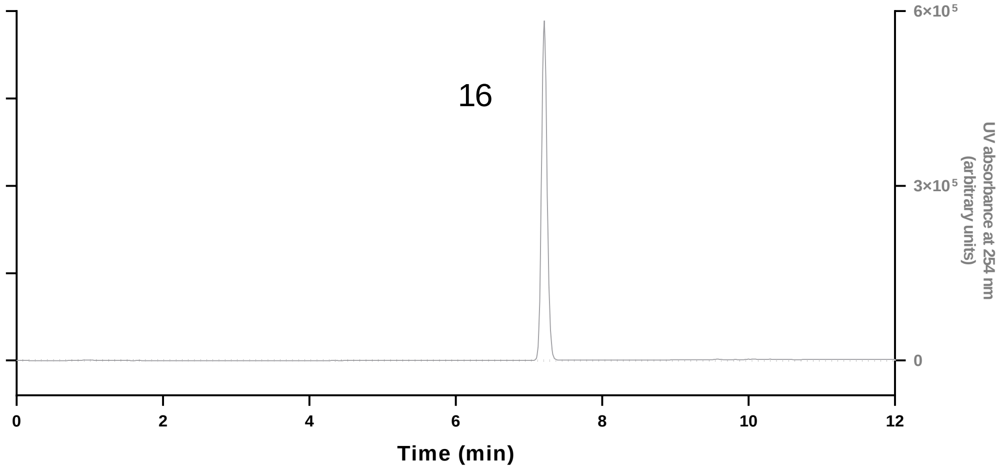

Supplementary Figure 71. Analytical HPLC chromatogram for **16.**

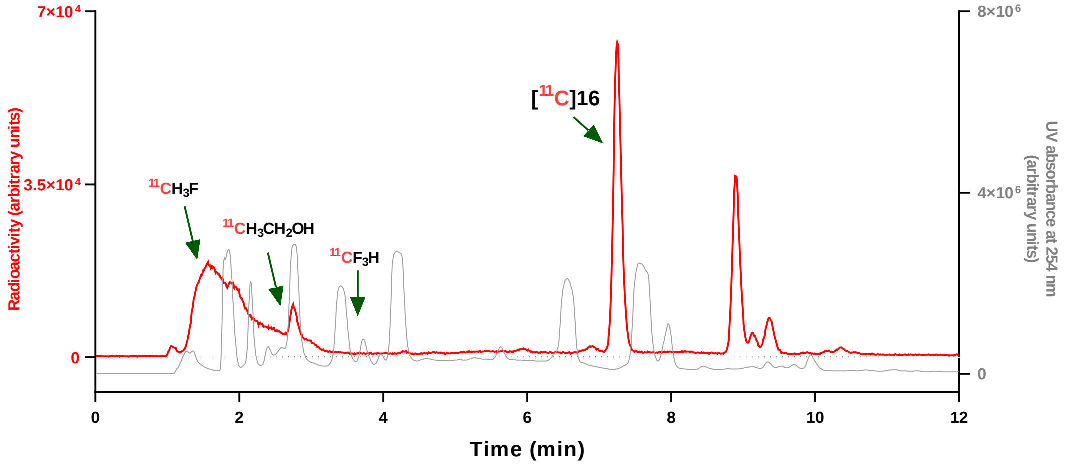

Supplementary Figure 72. Analytical HPLC chromatogram for reaction mixture.

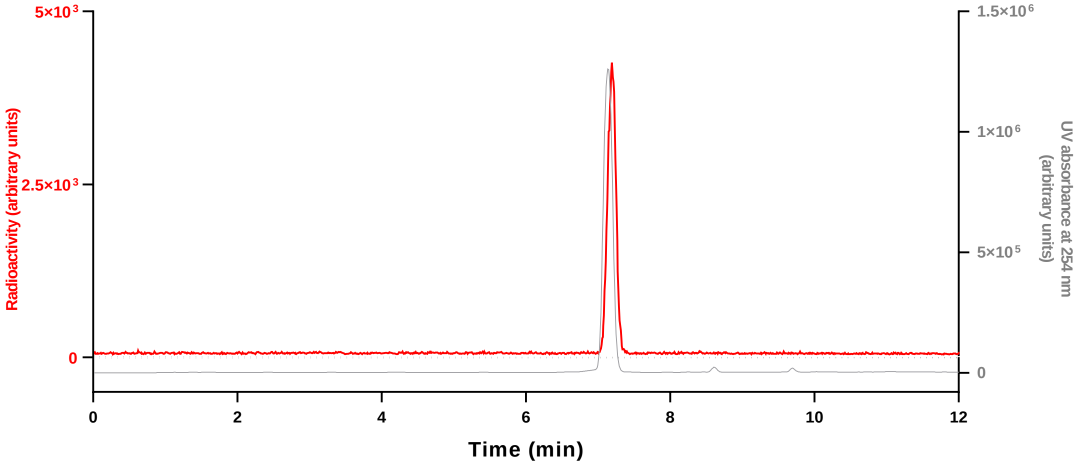

Supplementary Figure 73. Analytical HPLC chromatogram for [^{11}C]**16** collected from the above HPLC analysis when co–injected with **16.**

Run	Retention time (min)	Yield (decay-corrected) (%)	Mean yield ± SD (%)	Isolated yield (%)
1	7.252	47	51 ± 4	38
2	7.227	55		
3	7.221	50		

HPLC analysis of [^{11}C]17

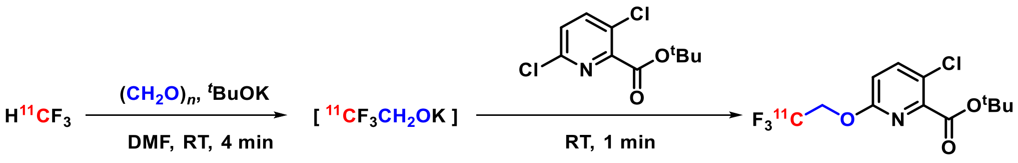

[^{11}C]17

Prepared following general procedure B and analyzed using HPLC condition A.

HPLC condition A

Column: Luna C18(2) (10 μm, 250 × 4.6 mm; i.d. Phenomenex)

Solvent A: H_2O, Solvent B: MeCN

Flow rate: 2 mL/min

0–1 min: 40% B

1–3 min: 40% to 60% B

3–6 min: 60% to 95% B

6–12 min: 95% B

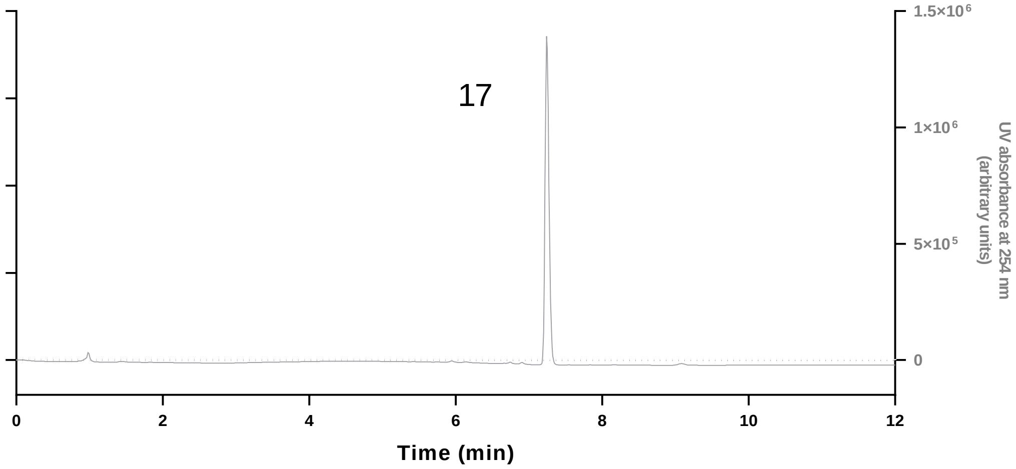

Supplementary Figure 74. Analytical HPLC chromatogram for **17.**

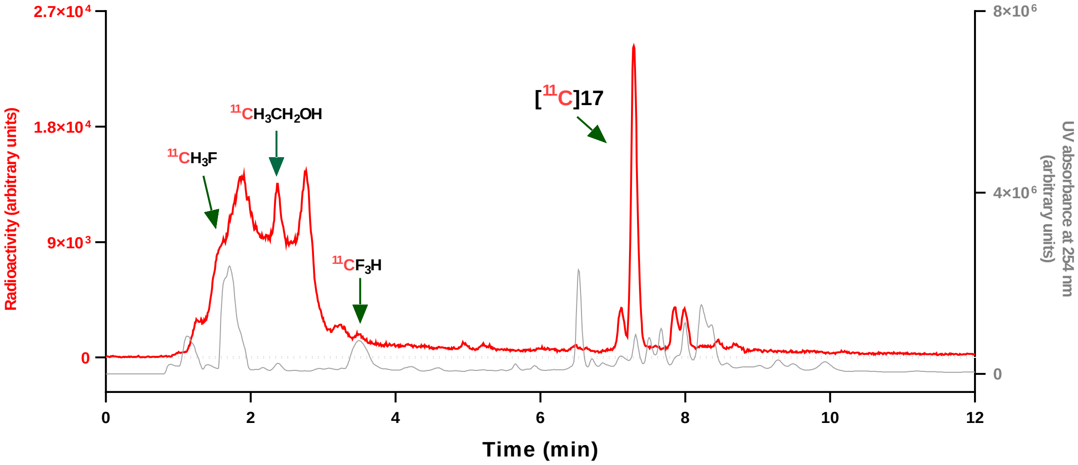

Supplementary Figure 75. Analytical HPLC chromatogram for reaction mixture.

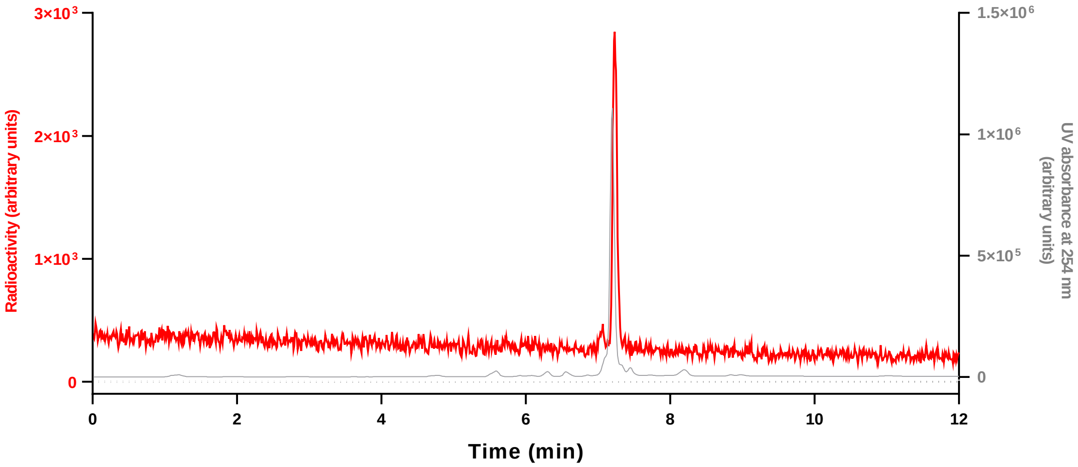

Supplementary Figure 76. Analytical HPLC chromatogram for [^{11}C]**17** collected from the above HPLC analysis when co–injected with **17.**

Run	Retention time (min)	Yield (decay-corrected) (%)	Mean yield ± SD (%)	Isolated yield (%)
1	7.291	37	34 ± 6	31
2	7.252	37		
3	7.246	27		

HPLC analysis of [^{11}C]18

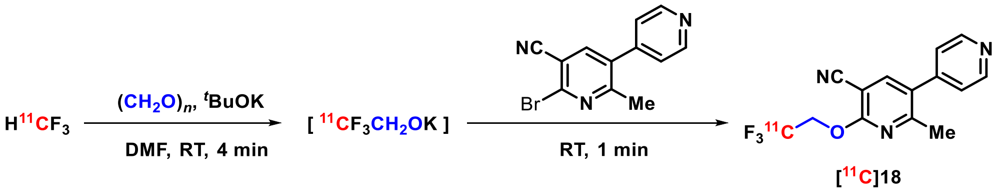

Prepared following general procedure B and analyzed using HPLC condition A.

HPLC condition A

Column: Luna C18(2) (10 μm, 250 × 4.6 mm; i.d. Phenomenex)

Solvent A: H_2O, Solvent B: MeCN

Flow rate: 2 mL/min

0–1 min: 40% B

1–3 min: 40% to 60% B

3–6 min: 60% to 95% B

6–12 min: 95% B

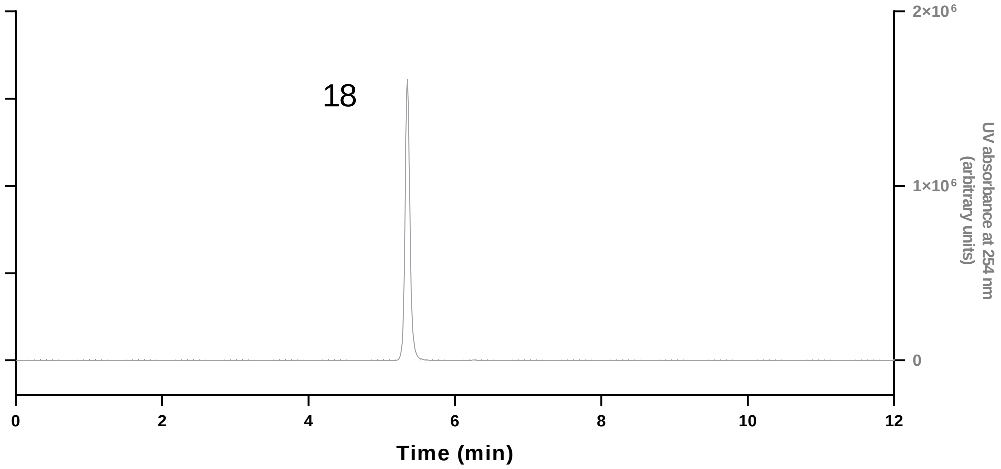

Supplementary Figure 77. Analytical HPLC chromatogram for **18.**

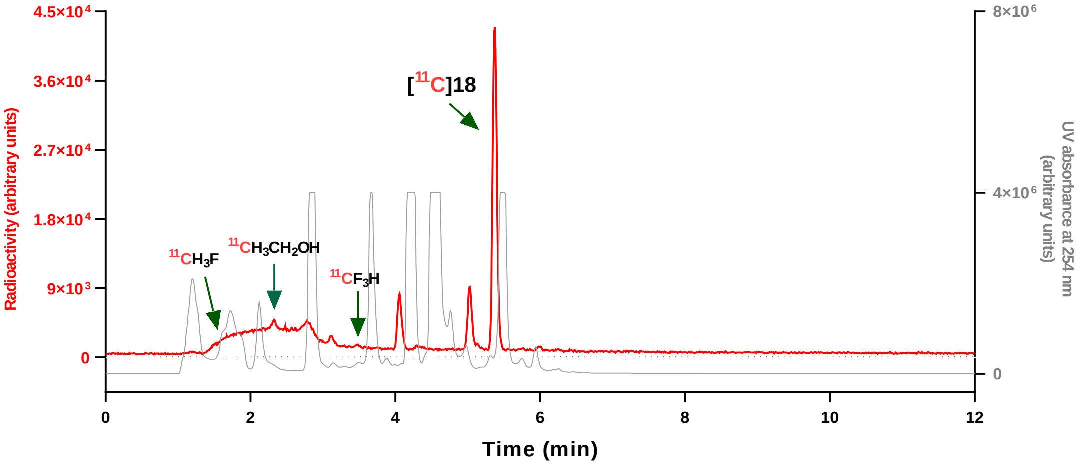

Supplementary Figure 78. Analytical HPLC chromatogram for reaction mixture.

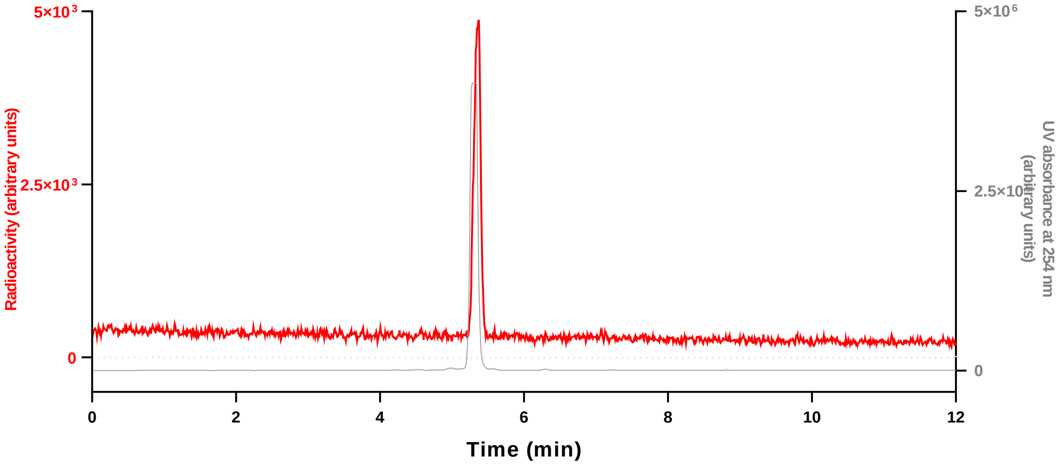

Supplementary Figure 79. Analytical HPLC chromatogram for [^{11}C]**18** collected from the above HPLC analysis when co–injected with **18.**

Run	Retention time (min)	Yield (decay-corrected) (%)	Mean yield ± SD (%)	Isolated yield (%)
1	5.372	61	57 ± 5	48
2	5.470	58		
3	5.470	52		

HPLC analysis of [^{11}C]19

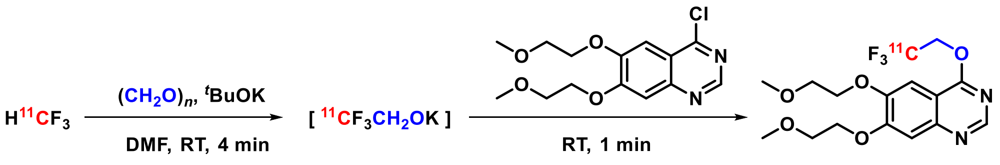

Prepared following general procedure B and analyzed using HPLC condition A.

HPLC condition A

Column: Luna C18(2) (10 μm, 250 × 4.6 mm; i.d. Phenomenex)

Solvent A: H_2O, Solvent B: MeCN

Flow rate: 2 mL/min

0–1 min: 40% B

1–3 min: 40% to 60% B

3–6 min: 60% to 95% B

6–12 min: 95% B

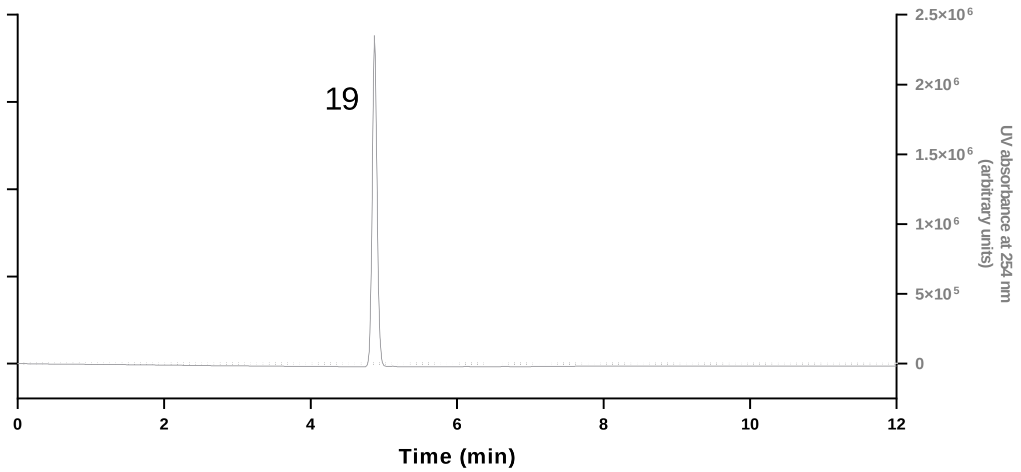

Supplementary Figure 80. Analytical HPLC chromatogram for **19.**

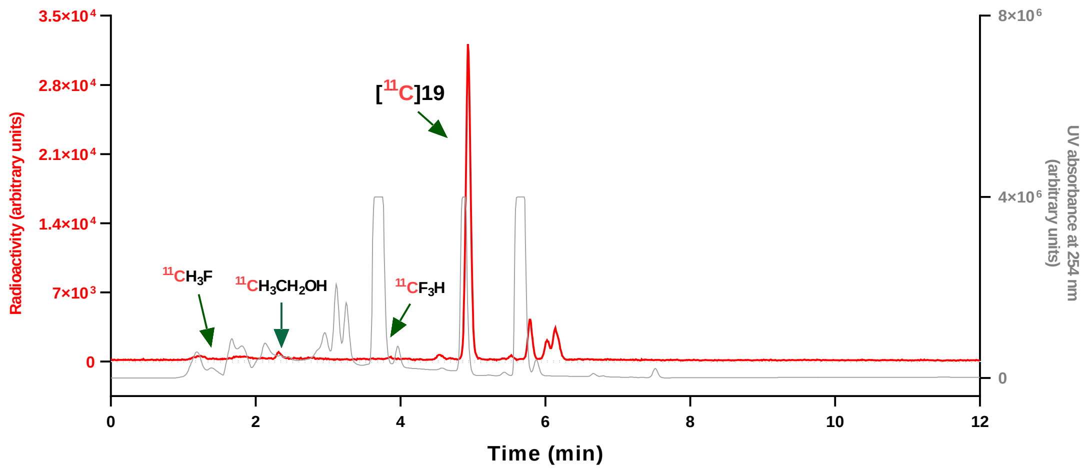

Supplementary Figure 81. Analytical HPLC chromatogram for reaction mixture.

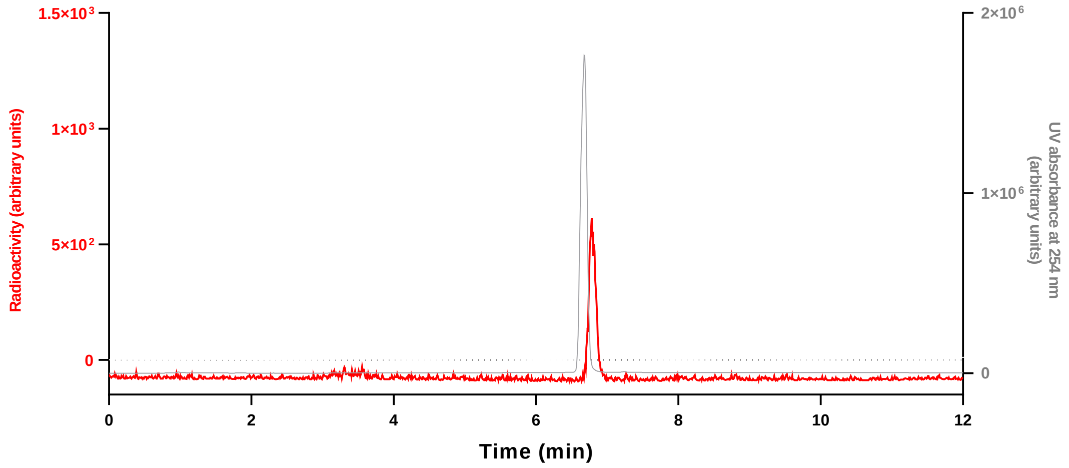

Supplementary Figure 82. Analytical HPLC chromatogram for [^{11}C]**19** collected from the above HPLC analysis when co–injected with **19.**

Run	Retention time (min)	Yield (decay-corrected) (%)	Mean yield ± SD (%)	Isolated yield (%)
1	4.932	70	68 ± 4	56
2	4.935	71		
3	4.518	63		

HPLC analysis of [^{11}C]20

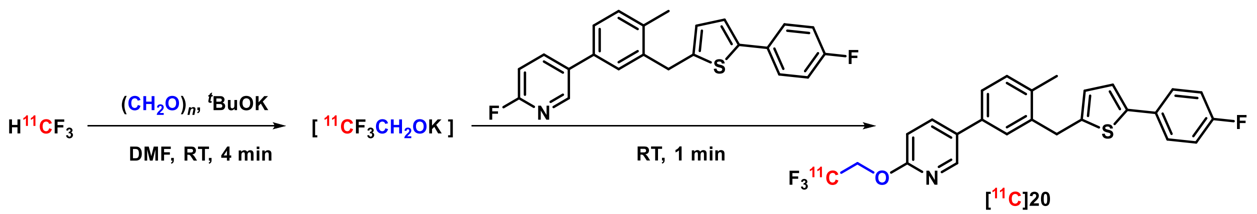

Prepared following general procedure B and analyzed using HPLC condition A.

HPLC condition A

Column: Luna C18(2) (10 μm, 250 × 4.6 mm; i.d. Phenomenex)

Solvent A: H_2O, Solvent B: MeCN

Flow rate: 2 mL/min

0–1 min: 40% B

1–3 min: 40% to 60% B

3–6 min: 60% to 95% B

6–12 min: 95% B

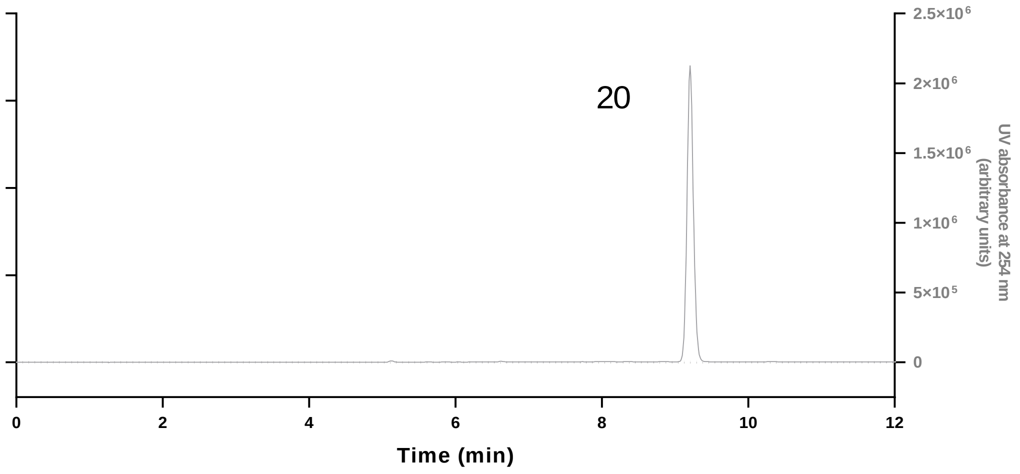

Supplementary Figure 83. Analytical HPLC chromatogram for **20.**

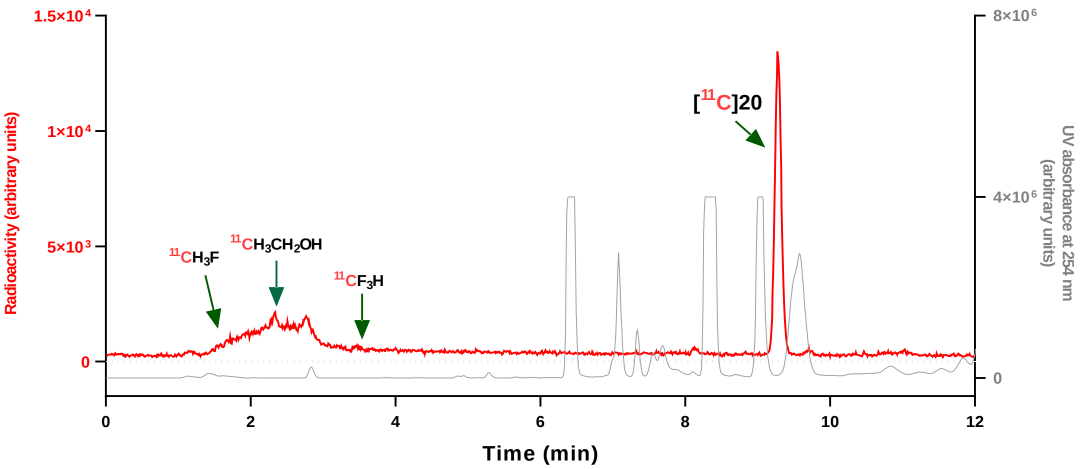

Supplementary Figure 84. Analytical HPLC chromatogram for reaction mixture.

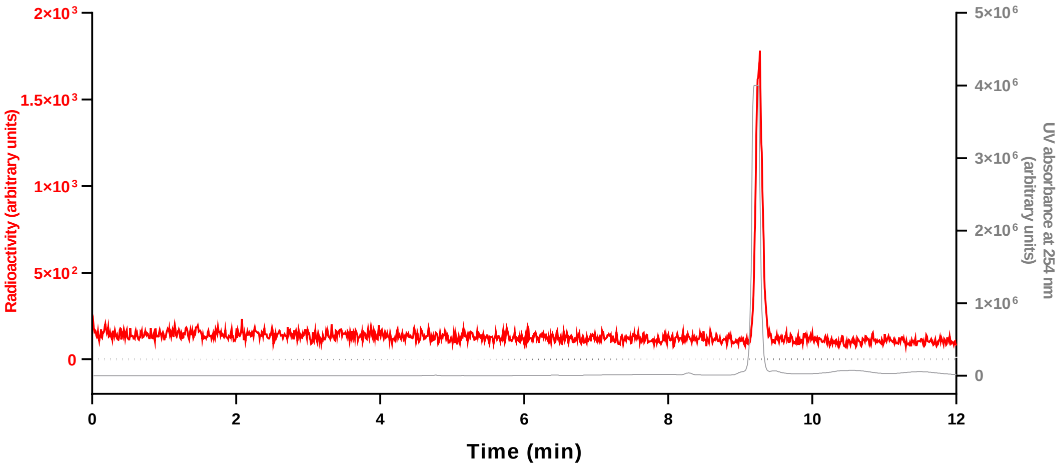

Supplementary Figure 85. Analytical HPLC chromatogram for [11C]**20** collected from the above HPLC analysis when co–injected with **20.**

Run	Retention time (min)	Yield (decay-corrected) (%)	Mean yield ± SD (%)	Isolated yield (%)
1	9.278	79	74 ± 4	61
2	9.277	72		
3	9.273	72		

HPLC analysis of [^{11}C]21

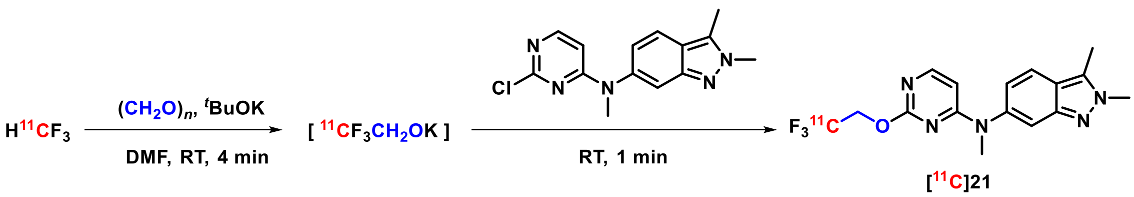

Prepared following general procedure B and analyzed using HPLC condition A.

HPLC condition A

Column: Luna C18(2) (10 μm, 250 × 4.6 mm; i.d. Phenomenex)

Solvent A: H_2O, Solvent B: MeCN

Flow rate: 2 mL/min

0–1 min: 40% B

1–3 min: 40% to 60% B

3–6 min: 60% to 95% B

6–12 min: 95% B

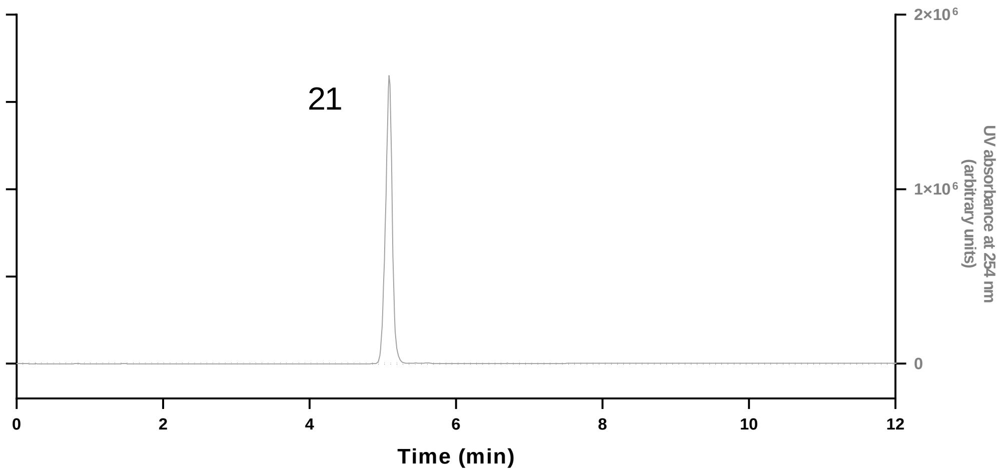

Supplementary Figure 86. Analytical HPLC chromatogram for **21.**

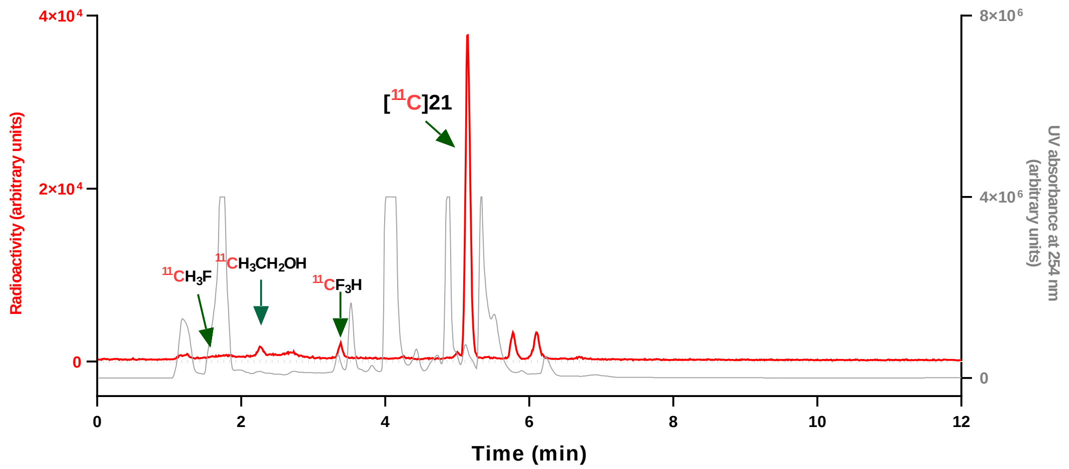

Supplementary Figure 87. Analytical HPLC chromatogram for reaction mixture.

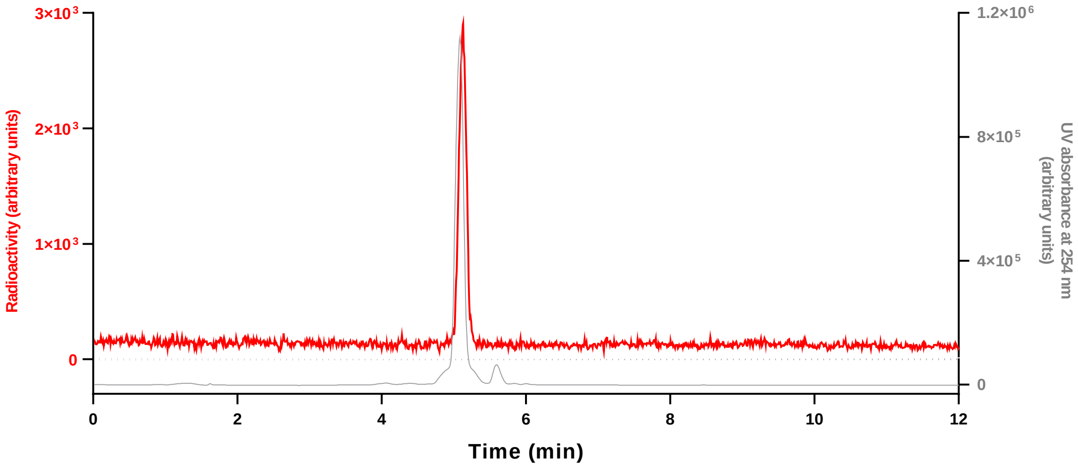

Supplementary Figure 88. Analytical HPLC chromatogram for [^{11}C]**21** collected from the above HPLC analysis when co–injected with **21.**

Run	Retention time (min)	Yield (decay-corrected) (%)	Mean yield ± SD (%)	Isolated yield (%)
1	5.146	75	73 ± 2	64
2	5.149	72		
3	5.146	71		

HPLC analysis of [^{11}C]22

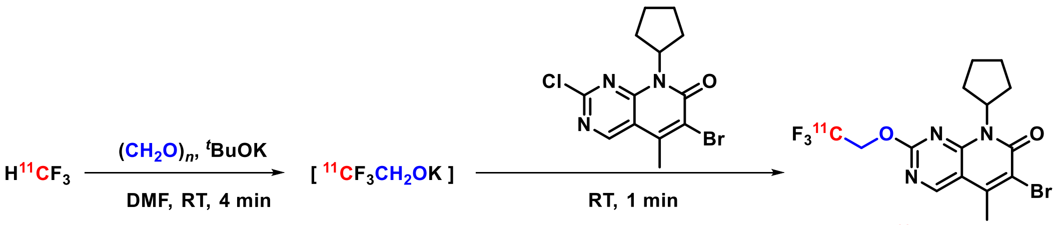

Prepared following general procedure B and analyzed using HPLC condition A.

HPLC condition A

Column: Luna C18(2) (10 μm, 250 × 4.6 mm; i.d. Phenomenex)

Solvent A: H_2O, Solvent B: MeCN

Flow rate: 2 mL/min

0–1 min: 40% B

1–3 min: 40% to 60% B

3–6 min: 60% to 95% B

6–12 min: 95% B

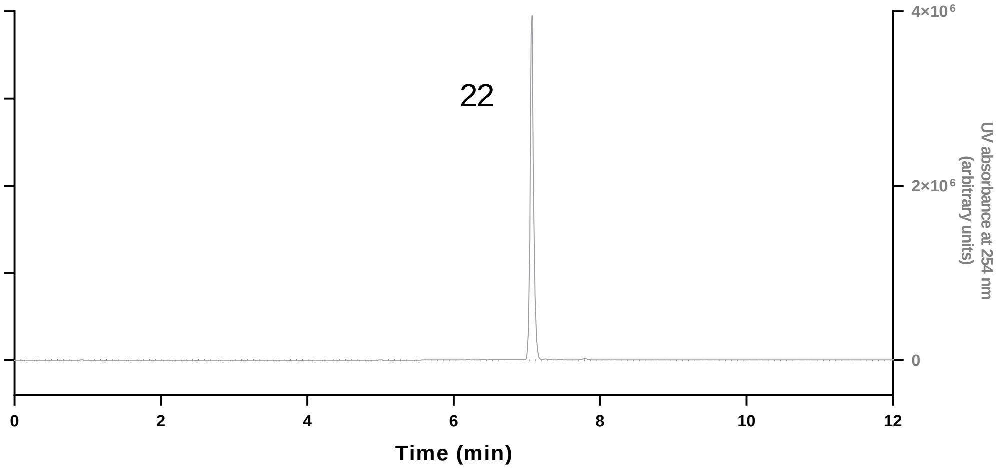

Supplementary Figure 89. Analytical HPLC chromatogram for **22.**

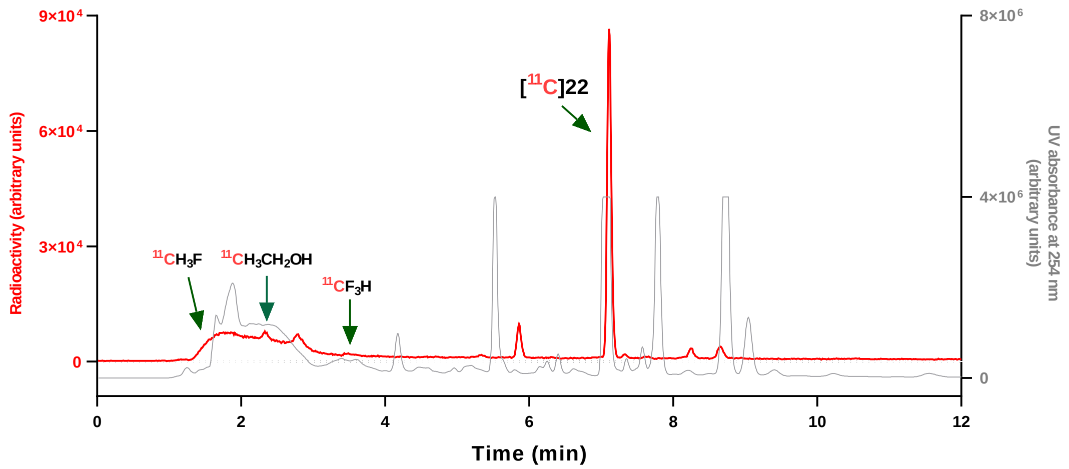

Supplementary Figure 90. Analytical HPLC chromatogram for reaction mixture.

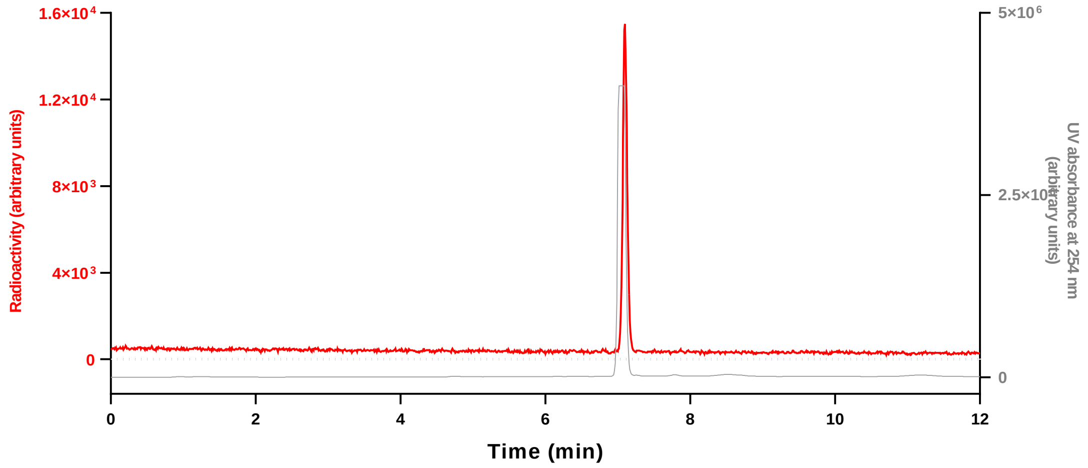

Supplementary Figure 91. Analytical HPLC chromatogram for [^{11}C]**22** collected from the above HPLC analysis when co–injected with **22.**

Run	Retention time (min)	Yield (decay-corrected) (%)	Mean yield ± SD (%)	Isolated yield (%)
1	7.104	65	67 ± 7	73
2	7.111	75		
3	7.104	62		

HPLC analysis of [^{11}C]23

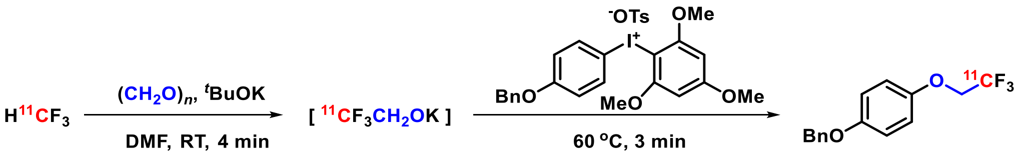

[^{11}C]23

Prepared following general procedure B and analyzed using HPLC condition A.

HPLC condition A

Column: Luna C18(2) (10 μm, 250 × 4.6 mm; i.d. Phenomenex)

Solvent A: H_2O, Solvent B: MeCN

Flow rate: 2 mL/min

0–1 min: 40% B

1–3 min: 40% to 60% B

3–6 min: 60% to 95% B

6–12 min: 95% B

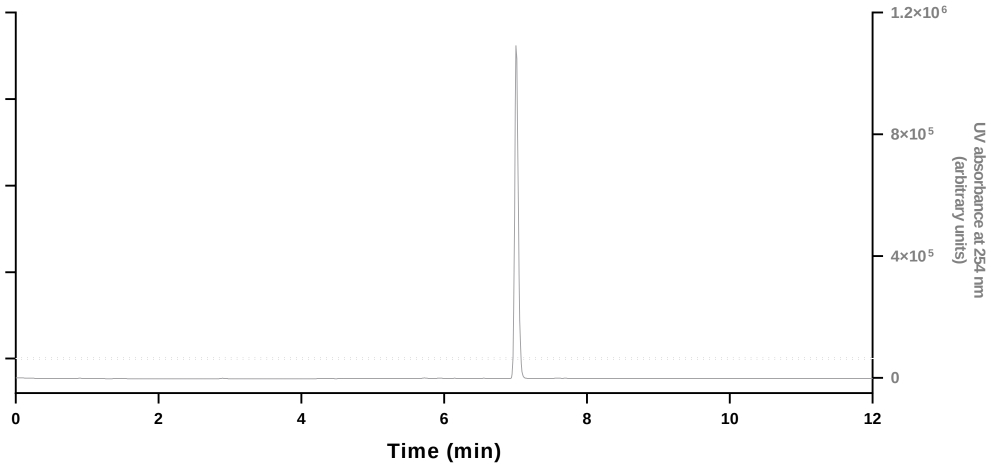

Supplementary Figure 92. Analytical HPLC chromatogram for **23.**

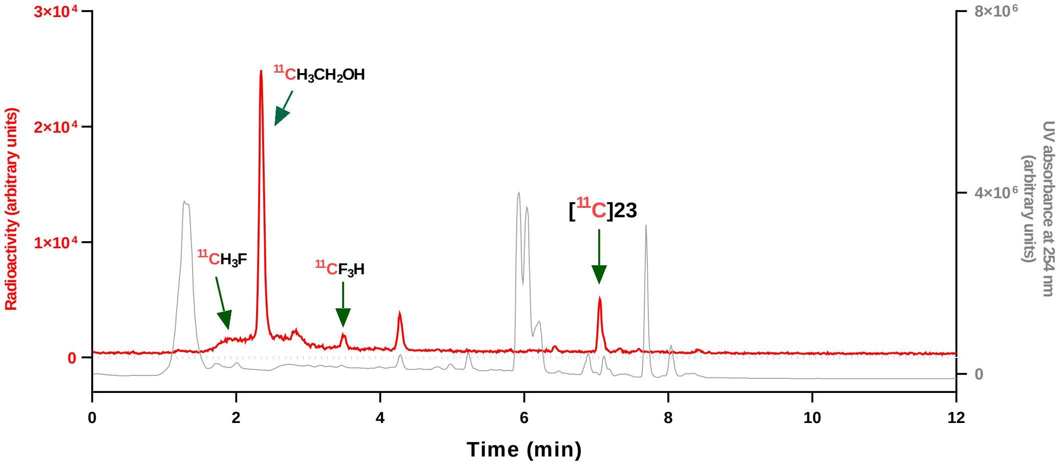

Supplementary Figure 93. Analytical HPLC chromatogram for reaction mixture.

Run	Retention time (min)	Yield (decay-corrected) (%)	Mean yield ± SD (%)
1	7.044	14	12 ± 2
2	7.052	13	
3	7.052	10	

HPLC analysis of [^{11}C]24

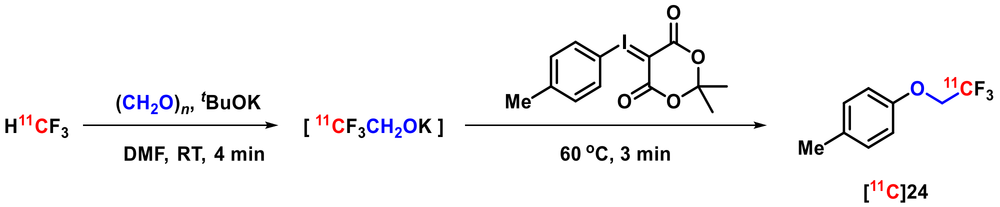

Prepared following general procedure B and analyzed using HPLC condition A.

HPLC condition A

Column: Luna C18(2) (10 μm, 250 × 4.6 mm; i.d. Phenomenex)

Solvent A: H_2O, Solvent B: MeCN

Flow rate: 2 mL/min

0–1 min: 40% B

1–3 min: 40% to 60% B

3–6 min: 60% to 95% B

6–12 min: 95% B

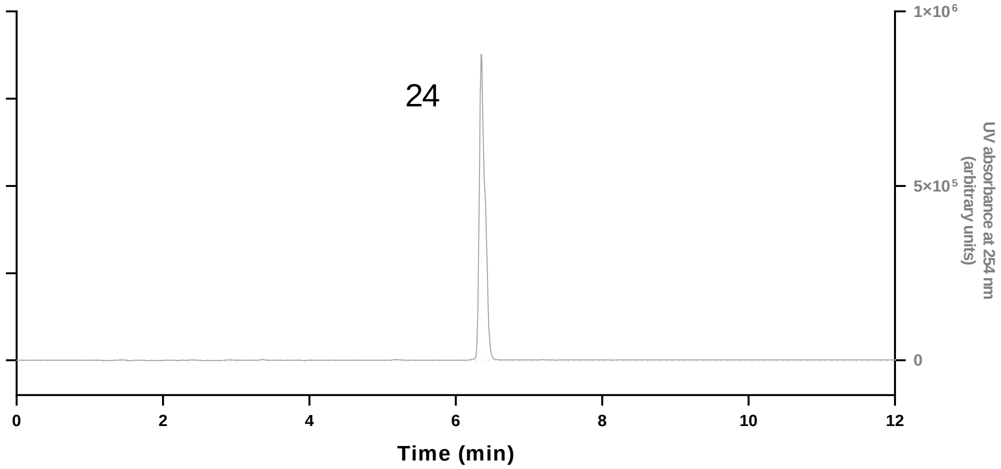

Supplementary Figure 94. Analytical HPLC chromatogram for **24.**

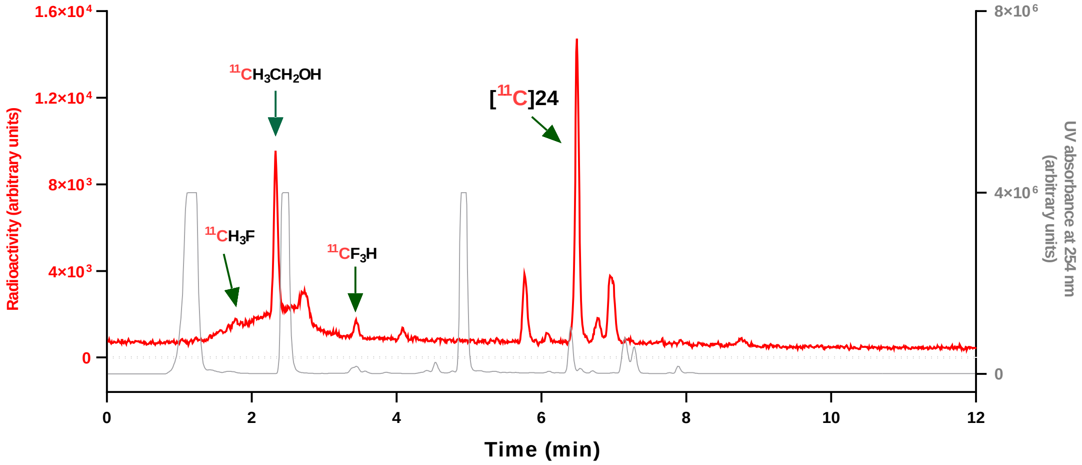

Supplementary Figure 95. Analytical HPLC chromatogram for reaction mixture.

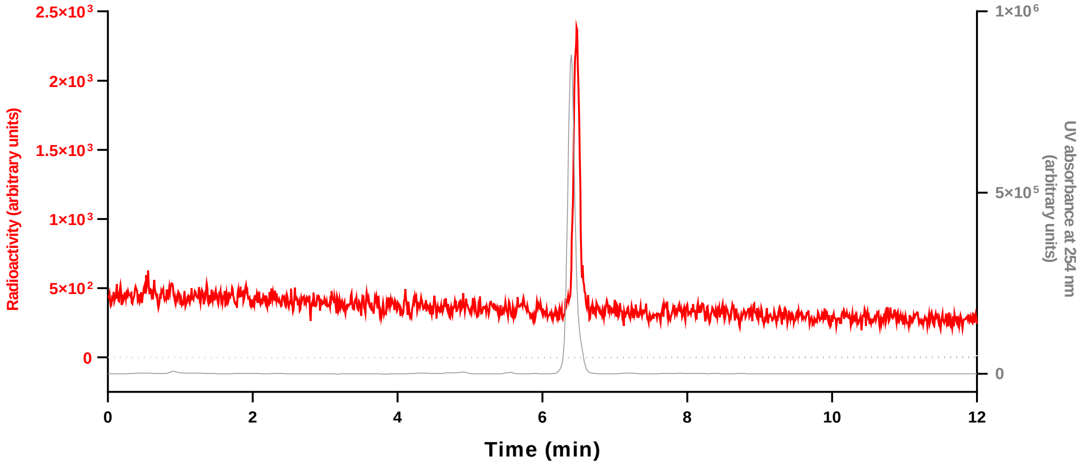

Supplementary Figure 96. Analytical HPLC chromatogram for [^{11}C]**24** collected from the above HPLC analysis when co–injected with **24.**

Run	Retention time (min)	Yield (decay-corrected) (%)	Mean yield ± SD (%)	Isolated yield (%)
1	6.560	44	36 ± 9	35
2	6.560	36		
3	6.495	27		

HPLC analysis of [^{11}C]25

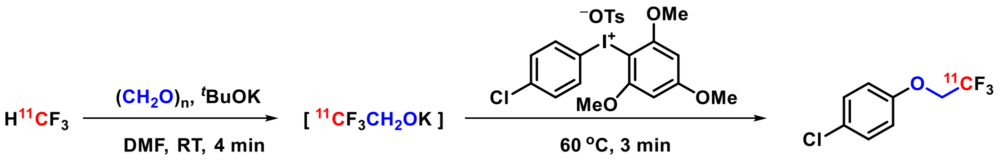

[^{11}C]25

Prepared following general procedure B and analyzed using HPLC condition A.

HPLC condition A

Column: Luna C18(2) (10 μm, 250 × 4.6 mm; i.d. Phenomenex)

Solvent A: H_2O, Solvent B: MeCN

Flow rate: 2 mL/min

0–1 min: 40% B

1–3 min: 40% to 60% B

3–6 min: 60% to 95% B

6–12 min: 95% B

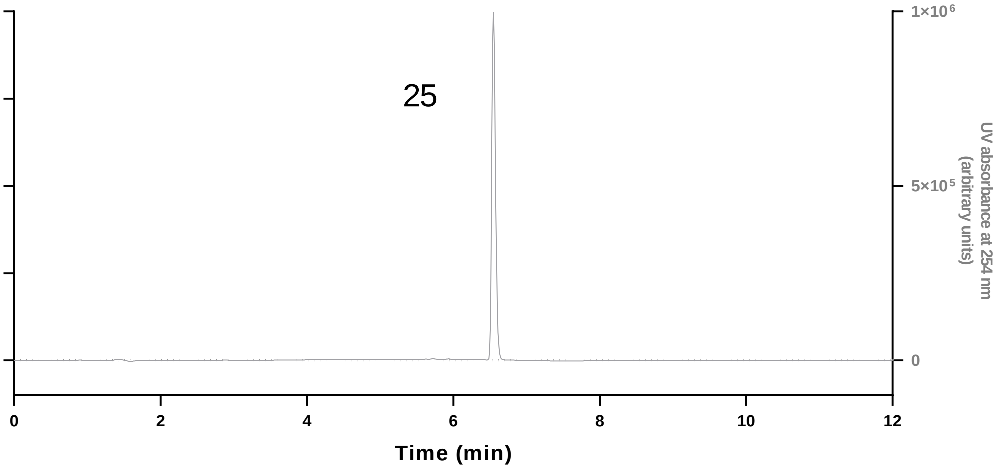

Supplementary Figure 97. Analytical HPLC chromatogram for **25.**

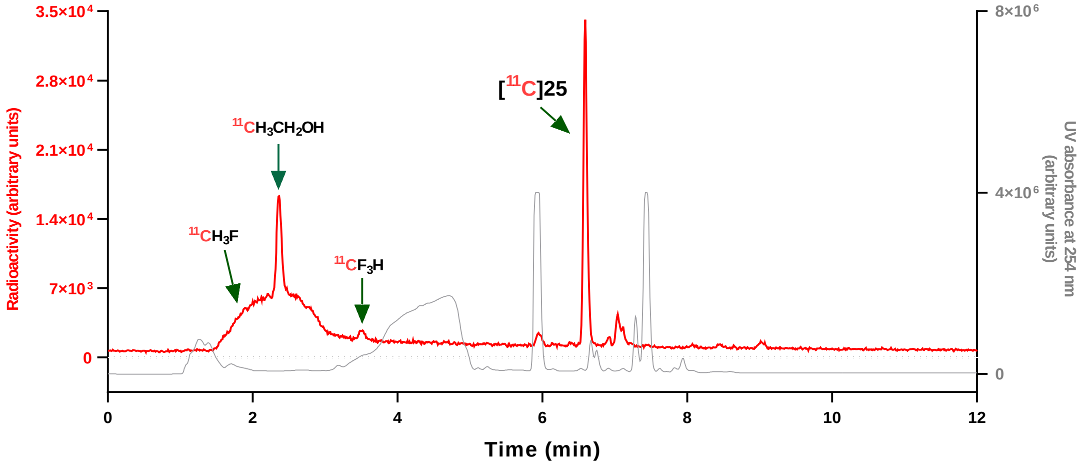

Supplementary Figure 98. Analytical HPLC chromatogram for reaction mixture.

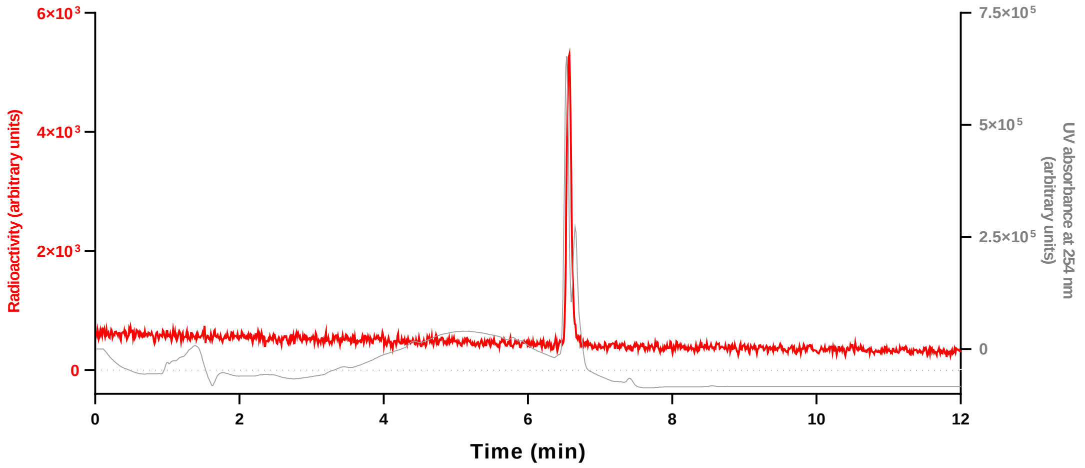

Supplementary Figure 99. Analytical HPLC chromatogram for [^{11}C]**25** collected from the above HPLC analysis when co–injected with **25.**

Run	Retention time (min)	Yield (decay-corrected) (%)	Mean yield ± SD (%)	Isolated yield (%)
1	6.591	49	49 ± 1	47
2	6.591	50		
3	6.594	48		

HPLC analysis of [^{11}C]26

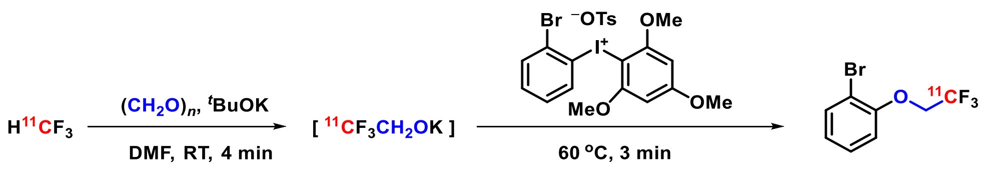

[^{11}C]26

Prepared following general procedure B and analyzed using HPLC condition A.

HPLC condition A

Column: Luna C18(2) (10 μm, 250 × 4.6 mm; i.d. Phenomenex)

Solvent A: H_2O, Solvent B: MeCN

Flow rate: 2 mL/min

0–1 min: 40% B

1–3 min: 40% to 60% B

3–6 min: 60% to 95% B

6–12 min: 95% B

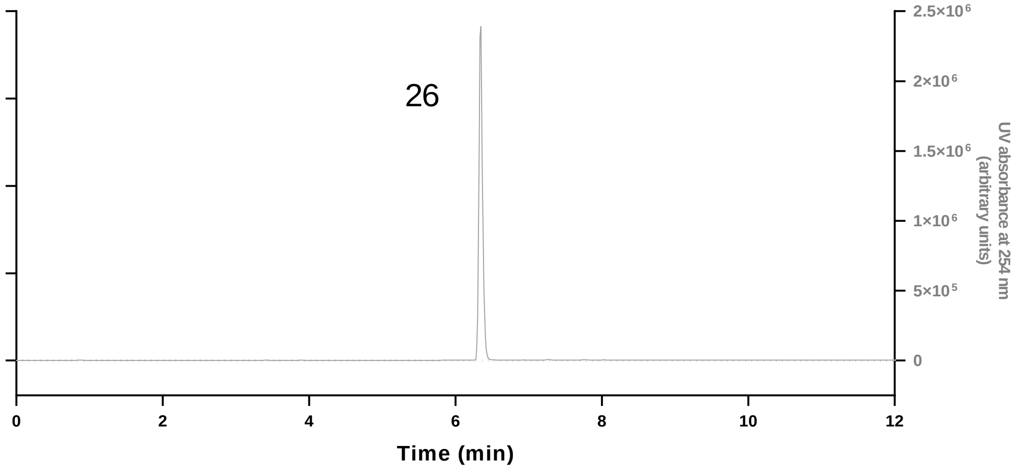

Supplementary Figure 100. Analytical HPLC chromatogram for **26.**

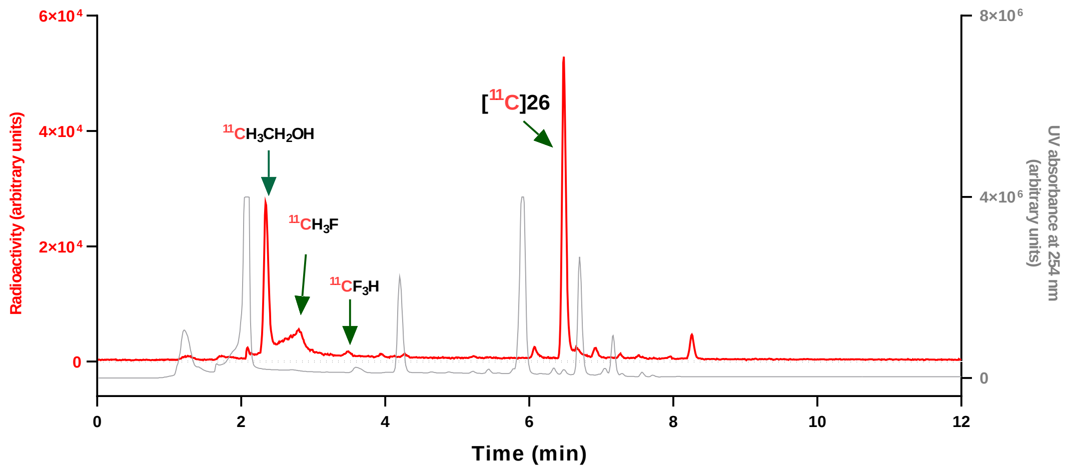

Supplementary Figure 101. Analytical HPLC chromatogram for reaction mixture.

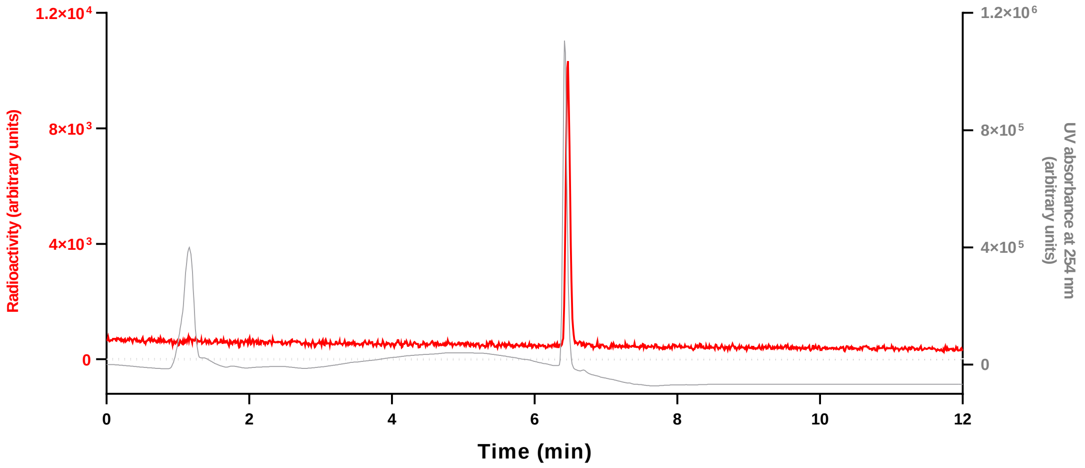

Supplementary Figure 102. Analytical HPLC chromatogram for [^{11}C]**26** collected from the above HPLC analysis when co–injected with **26.**

Run	Retention time (min)	Yield (decay-corrected) (%)	Mean yield ± SD (%)	Isolated yield (%)
1	6.481	56	50 ± 5	38
2	6.488	46		
3	6.485	49		

HPLC analysis of [^{11}C]27

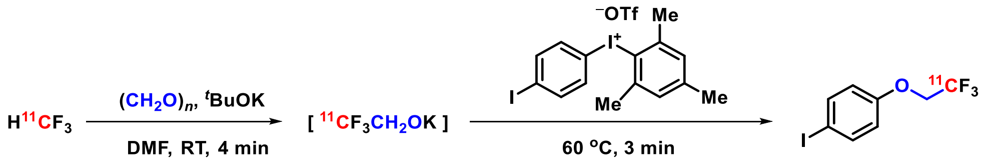

[^{11}C]27

Prepared following general procedure B and analyzed using HPLC condition A.

HPLC condition A

Column: Luna C18(2) (10 μm, 250 × 4.6 mm; i.d. Phenomenex)

Solvent A: H_2O, Solvent B: MeCN

Flow rate: 2 mL/min

0–1 min: 40% B

1–3 min: 40% to 60% B

3–6 min: 60% to 95% B

6–12 min: 95% B

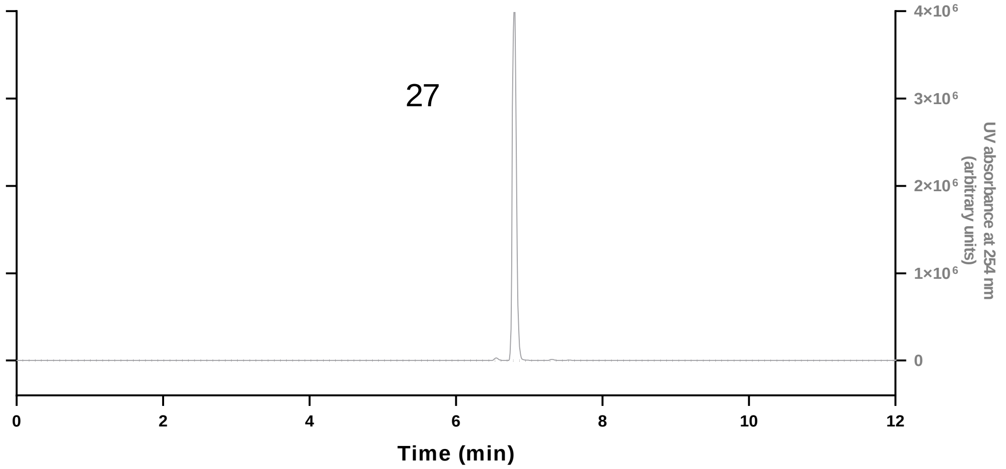

Supplementary Figure 103. Analytical HPLC chromatogram for **27.**

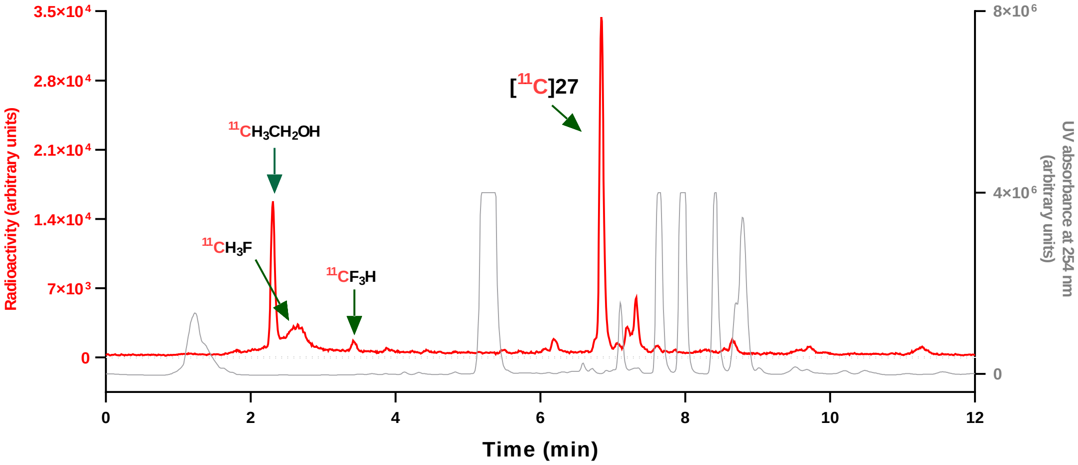

Supplementary Figure 104. Analytical HPLC chromatogram for reaction mixture.

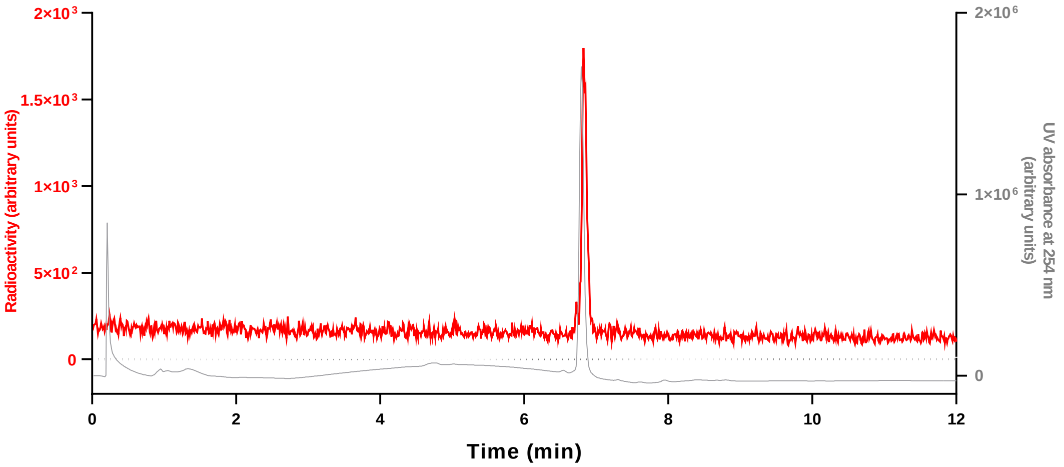

Supplementary Figure 105. Analytical HPLC chromatogram for [^{11}C]**27** collected from the above HPLC analysis when co–injected with **27.**

Run	Retention time (min)	Yield (decay-corrected) (%)	Mean yield ± SD (%)	Isolated yield (%)
1	6.837	48	47 ± 3	42
2	6.845	49		
3	6.842	44		

HPLC analysis of [^{11}C]28

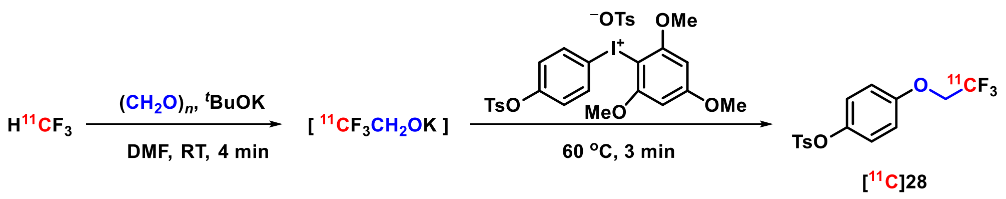

Prepared following general procedure B and analyzed using HPLC condition A.

HPLC condition A

Column: Luna C18(2) (10 μm, 250 × 4.6 mm; i.d. Phenomenex)

Solvent A: H_2O, Solvent B: MeCN

Flow rate: 2 mL/min

0–1 min: 40% B

1–3 min: 40% to 60% B

3–6 min: 60% to 95% B

6–12 min: 95% B

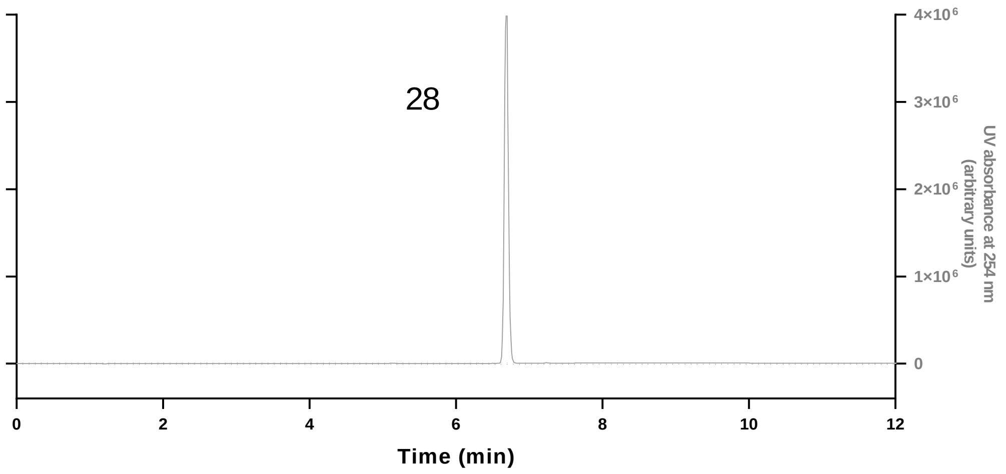

Supplementary Figure 106. Analytical HPLC chromatogram for **28.**

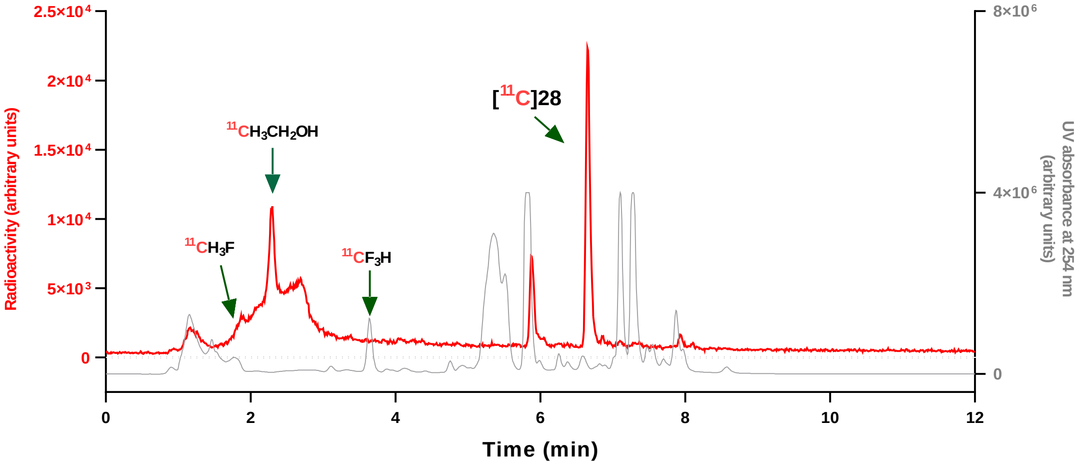

Supplementary Figure 107. Analytical HPLC chromatogram for reaction mixture.

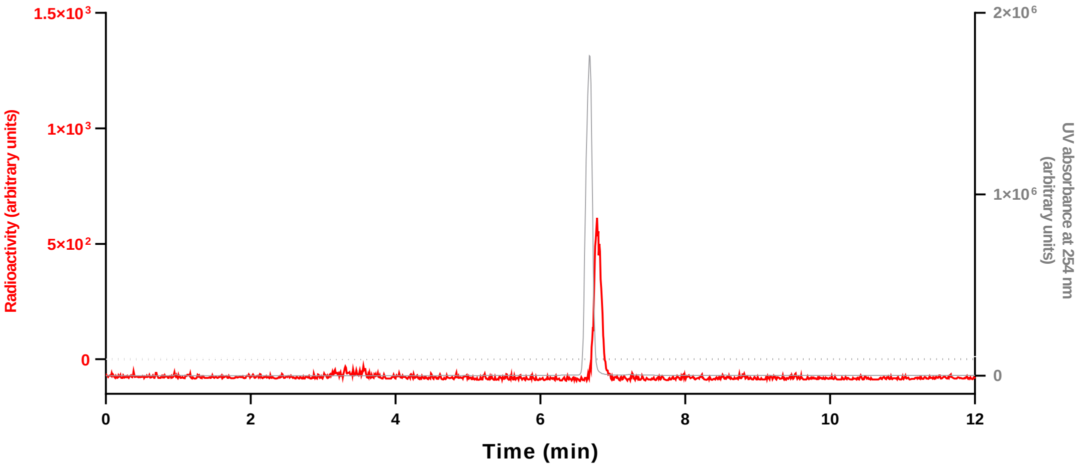

Supplementary Figure 108. Analytical HPLC chromatogram for [^{11}C]**28** collected from the above HPLC analysis when co–injected with **28.**

Run	Retention time (min)	Yield (decay-corrected) (%)	Mean yield ± SD (%)	Isolated yield (%)
1	6.730	34	33 ± 1	25
2	6.654	32		
3	6.622	33		

HPLC analysis of [^{11}C]29

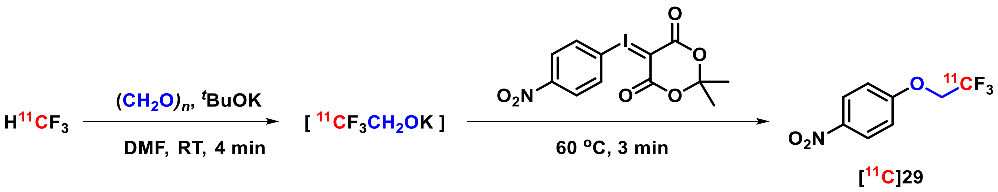

Prepared following general procedure B and analyzed using HPLC condition A.

HPLC condition A

Column: Luna C18(2) (10 μm, 250 × 4.6 mm; i.d. Phenomenex)

Solvent A: H_2O, Solvent B: MeCN

Flow rate: 2 mL/min

0–1 min: 40% B

1–3 min: 40% to 60% B

3–6 min: 60% to 95% B

6–12 min: 95% B

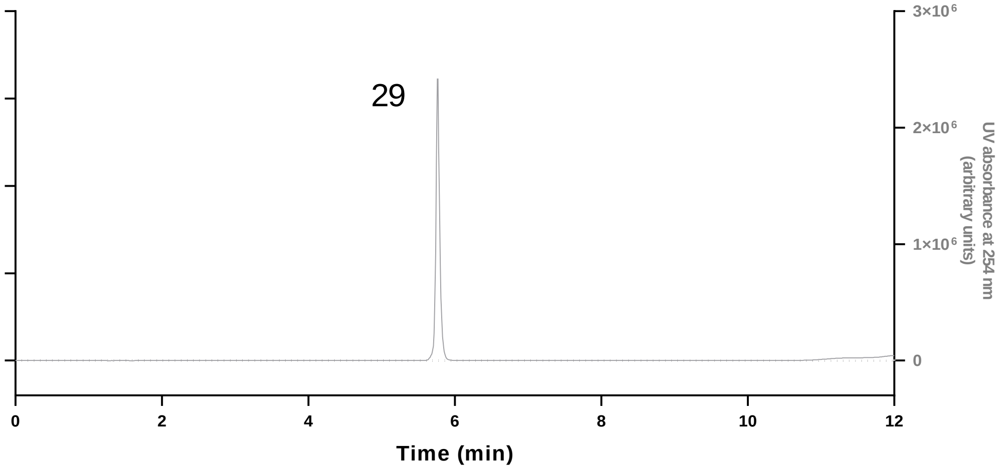

Supplementary Figure 109. Analytical HPLC chromatogram for **29.**

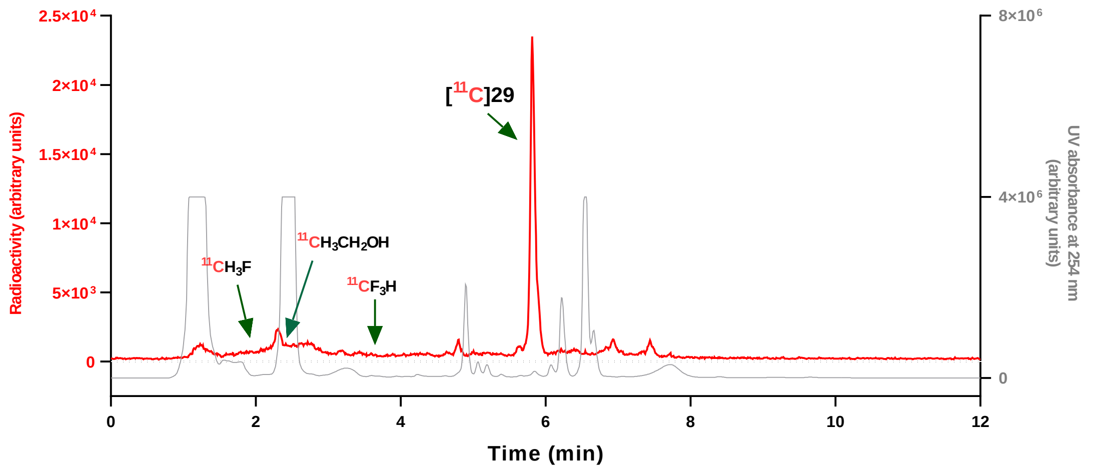

Supplementary Figure 110. Analytical HPLC chromatogram for reaction mixture.

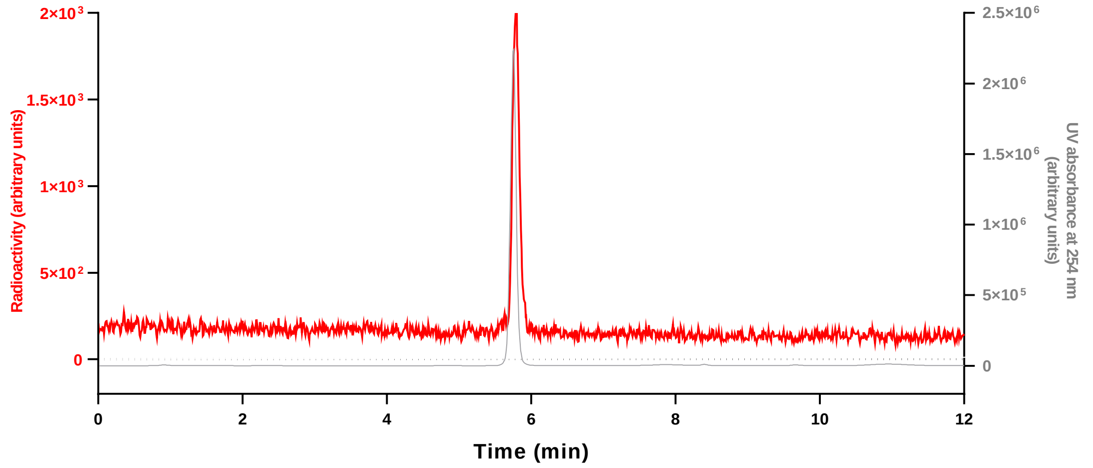

Supplementary Figure 111. Analytical HPLC chromatogram for [11C]**29** collected from the above HPLC analysis when co–injected with **29.**

Run	Retention time (min)	Yield (decay-corrected) (%)	Mean yield ± SD (%)	Isolated yield (%)
1	5.807	71	71 ± 1	57
2	5.817	72		
3	5.820	71		

HPLC analysis of [^{11}C]30

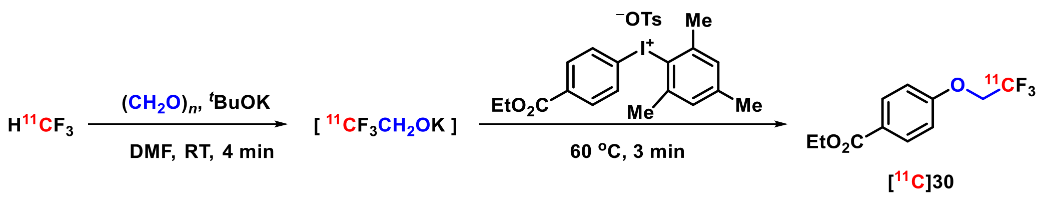

Prepared following general procedure B and analyzed using HPLC condition A.

HPLC condition A

Column: Luna C18(2) (10 μm, 250 × 4.6 mm; i.d. Phenomenex)

Solvent A: H_2O, Solvent B: MeCN

Flow rate: 2 mL/min

0–1 min: 40% B

1–3 min: 40% to 60% B

3–6 min: 60% to 95% B

6–12 min: 95% B

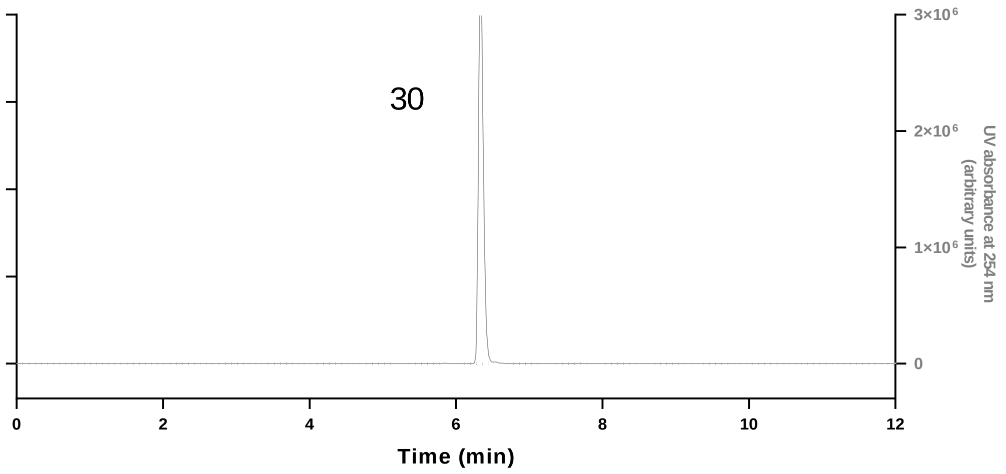

Supplementary Figure 112. Analytical HPLC chromatogram for **30.**

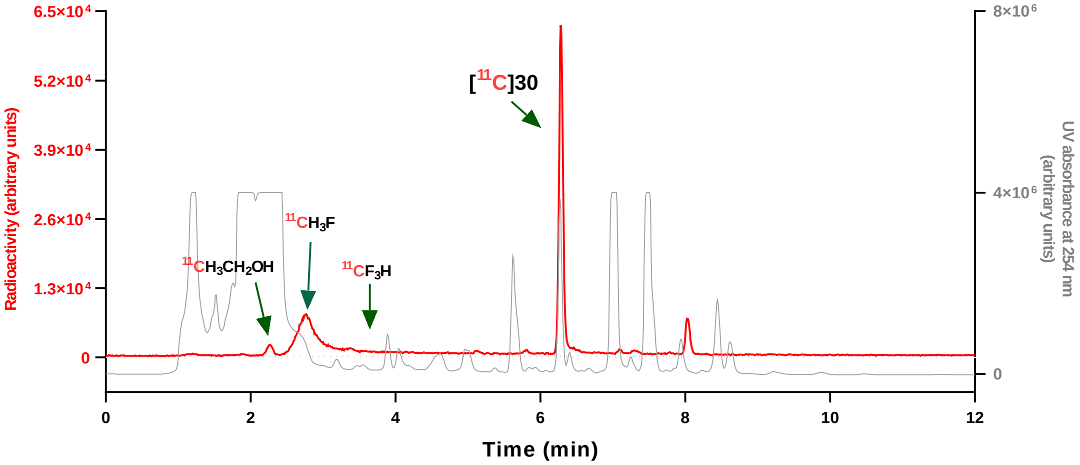

Supplementary Figure 113. Analytical HPLC chromatogram for reaction mixture.

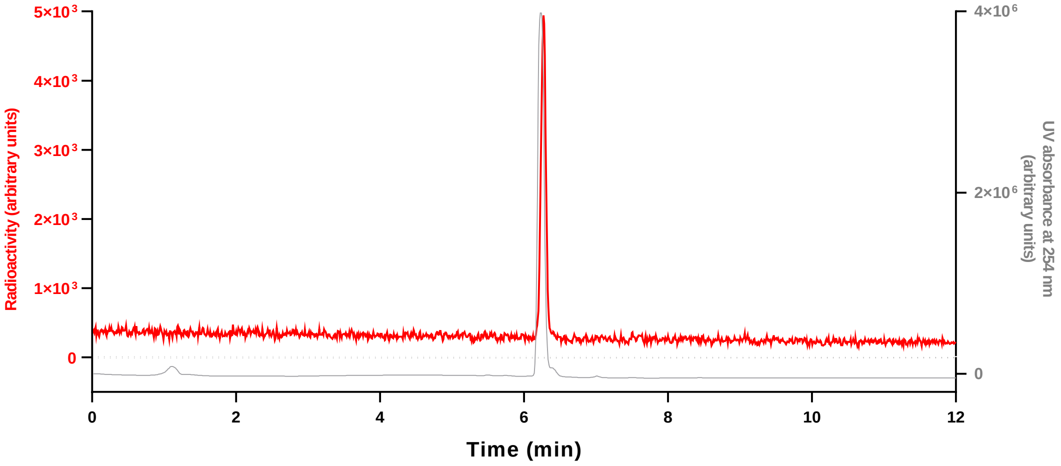

Supplementary Figure 114. Analytical HPLC chromatogram for [^{11}C]**30** collected from the above HPLC analysis when co–injected with **30.**

Run	Retention time (min)	Yield (decay-corrected) (%)	Mean yield ± SD (%)	Isolated yield (%)
1	6.285	86	86 ± 1	74
2	6.281	86		
3	6.450	85		

HPLC analysis of [^{11}C]31

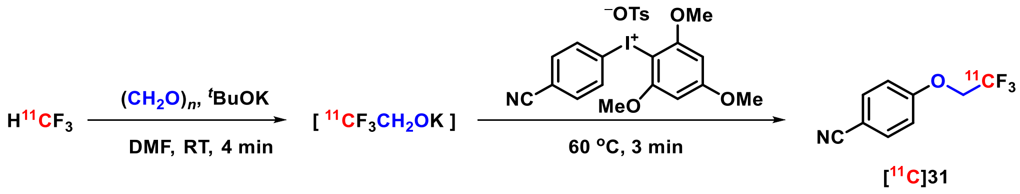

Prepared following general procedure B and analyzed using HPLC condition A.

HPLC condition A

Column: Luna C18(2) (10 μm, 250 × 4.6 mm; i.d. Phenomenex)

Solvent A: H_2O, Solvent B: MeCN

Flow rate: 2 mL/min

0–1 min: 40% B

1–3 min: 40% to 60% B

3–6 min: 60% to 95% B

6–12 min: 95% B

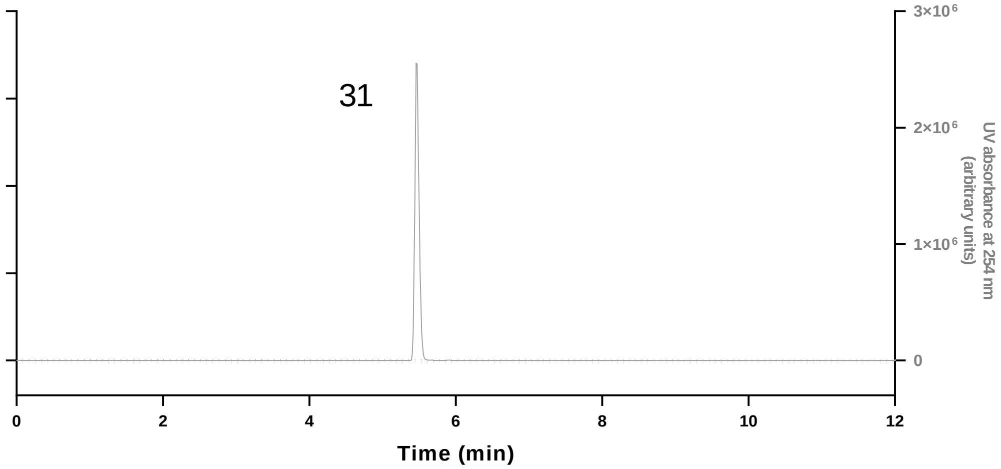

Supplementary Figure 115. Analytical HPLC chromatogram for **31.**

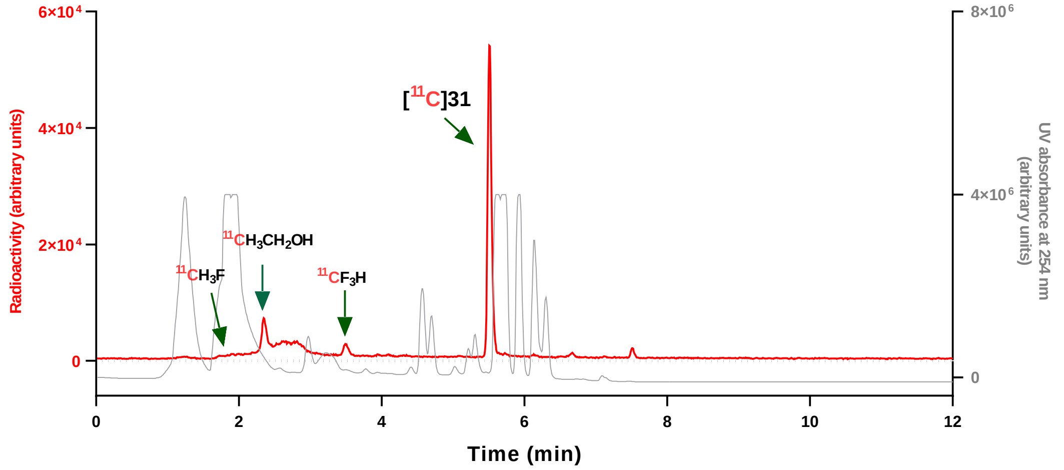

Supplementary Figure 116. Analytical HPLC chromatogram for reaction mixture.

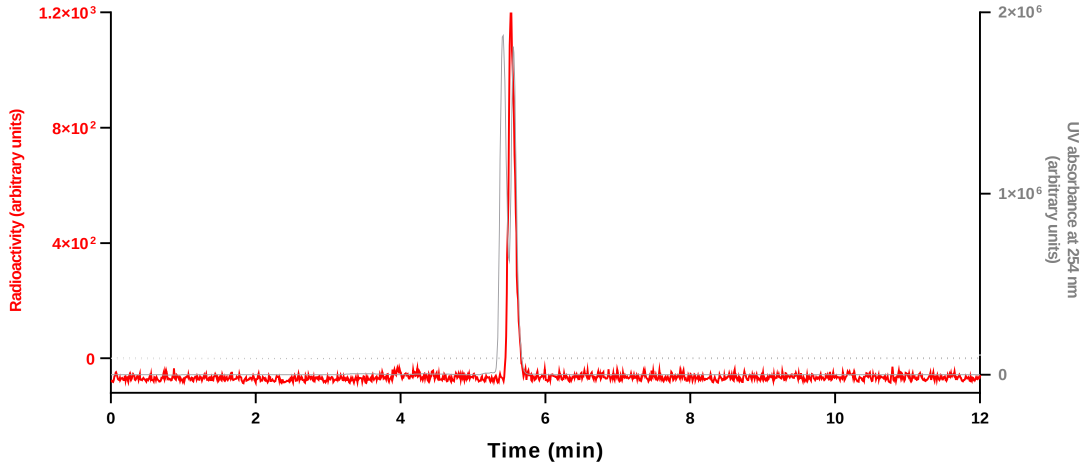

Supplementary Figure 117. Analytical HPLC chromatogram for [^{11}C]**31** collected from the above HPLC analysis when co-injected with **31.**

Run	Retention time (min)	Yield (decay-corrected) (%)	Mean yield ± SD (%)	Isolated yield (%)
1	5.518	77	80 ± 3	73
2	5.512	81		
3	5.550	82		

HPLC analysis of [^{11}C]32

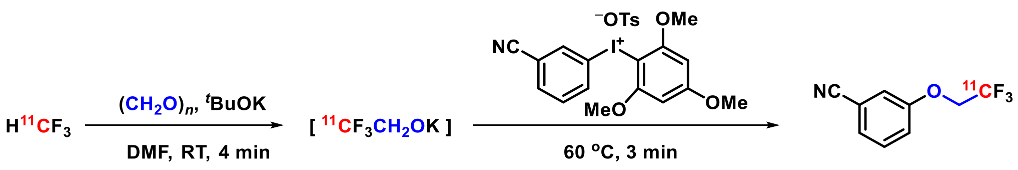

[^{11}C]32

Prepared following general procedure B and analyzed using HPLC condition A.

HPLC condition A

Column: Luna C18(2) (10 μm, 250 × 4.6 mm; i.d. Phenomenex)

Solvent A: H_2O, Solvent B: MeCN

Flow rate: 2 mL/min

0–1 min: 40% B

1–3 min: 40% to 60% B

3–6 min: 60% to 95% B

6–12 min: 95% B

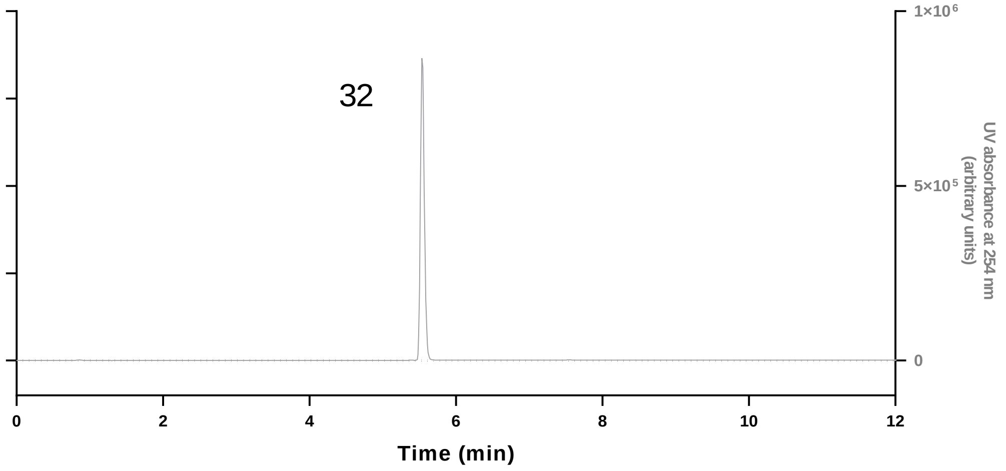

Supplementary Figure 118. Analytical HPLC chromatogram for **32.**

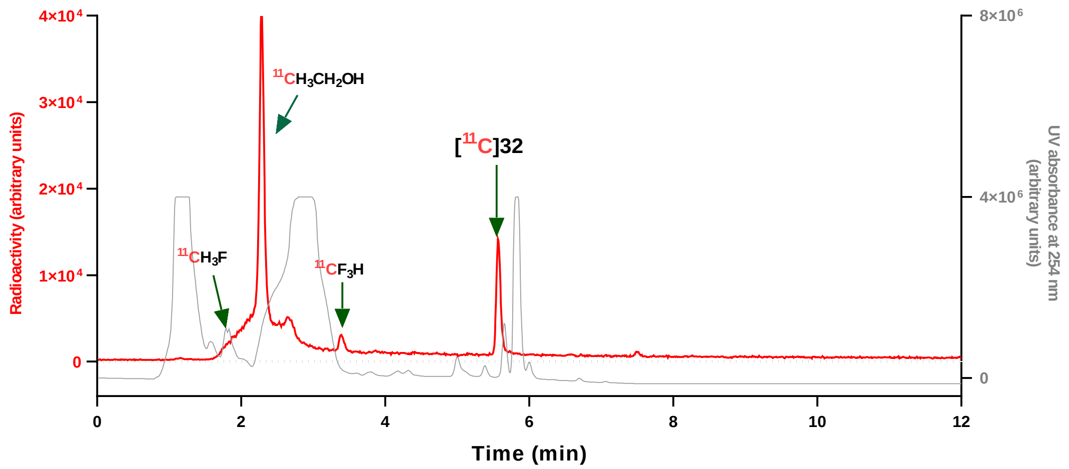

Supplementary Figure 119. Analytical HPLC chromatogram for reaction mixture.

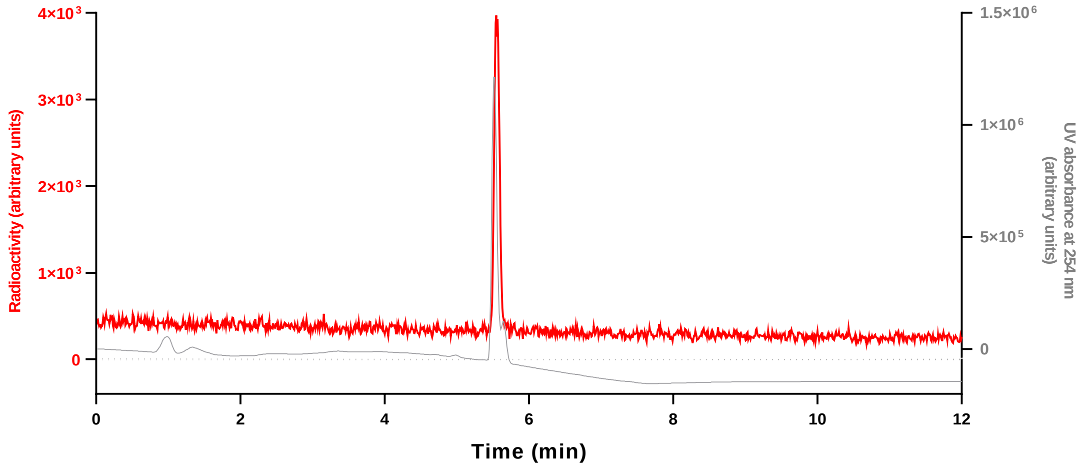

Supplementary Figure 120. Analytical HPLC chromatogram for [^{11}C]**32** collected from the above HPLC analysis when co–injected with **32.**

Run	Retention time (min)	Yield (decay-corrected) (%)	Mean yield ± SD (%)	Isolated yield (%)
1	5.579	22	22 ± 1	23
2	5.576	21		
3	5.570	22		

HPLC analysis of [^{11}C]33

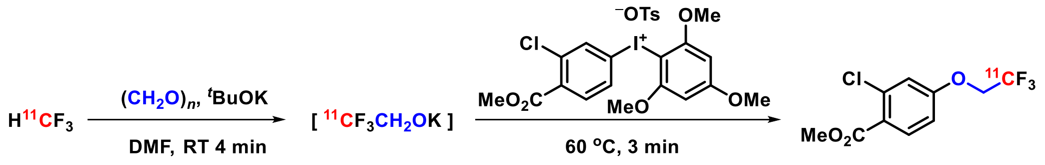

[^{11}C]33

Prepared following general procedure B and analyzed using HPLC condition A.

HPLC condition A

Column: Luna C18(2) (10 μm, 250 × 4.6 mm; i.d. Phenomenex)

Solvent A: H_2O, Solvent B: MeCN

Flow rate: 2 mL/min

0–1 min: 40% B

1–3 min: 40% to 60% B

3–6 min: 60% to 95% B

6–12 min: 95% B

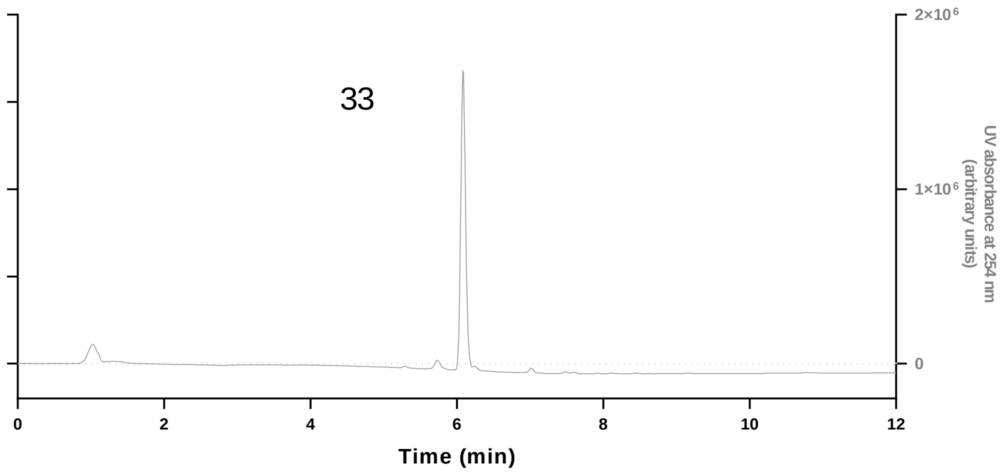

Supplementary Figure 121. Analytical HPLC chromatogram for **33.**

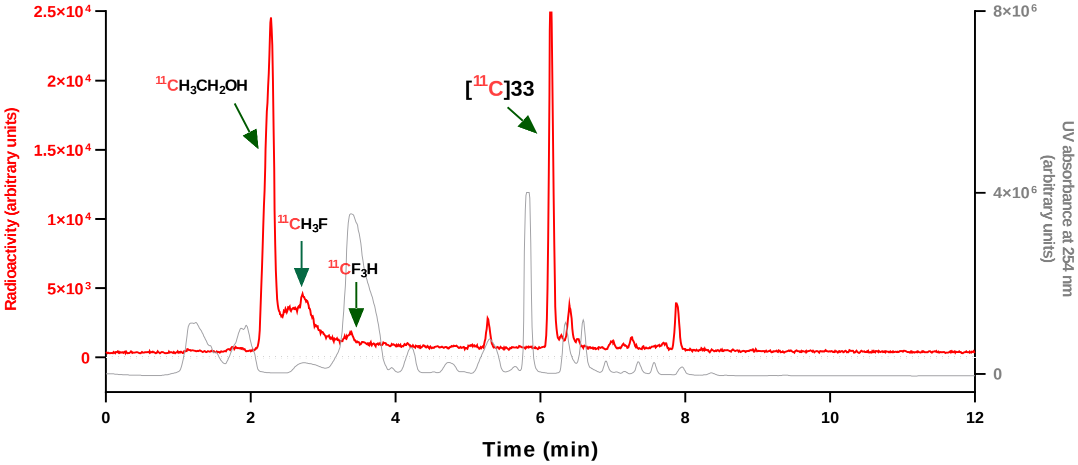

Supplementary Figure 122. Analytical HPLC chromatogram for reaction mixture.

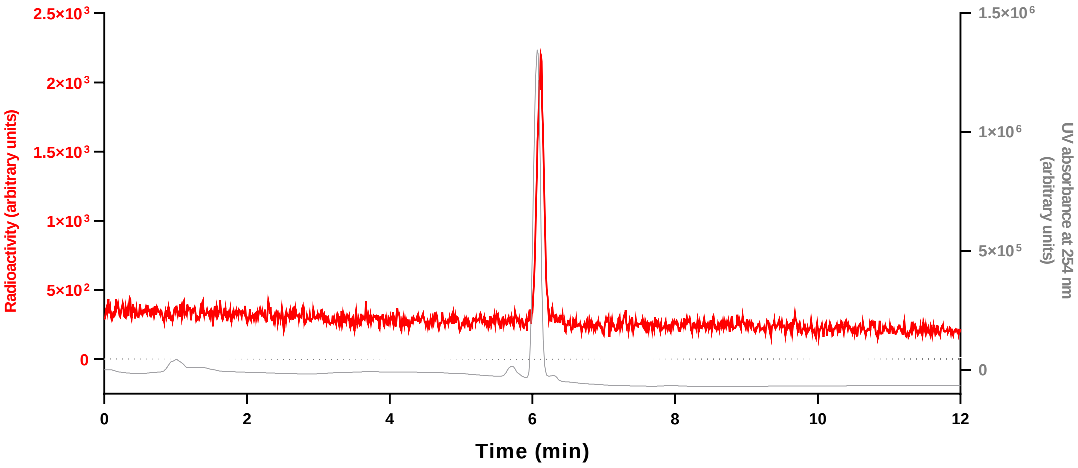

Supplementary Figure 123. Analytical HPLC chromatogram for [^{11}C]**33** collected from the above HPLC analysis when co–injected with **33.**

Run	Retention time (min)	Yield (decay-corrected) (%)	Mean yield ± SD (%)	Isolated yield (%)
1	6.144	33	32 ± 2	30
2	6.147	32		
3	6.148	30		

HPLC analysis of [^{11}C]34

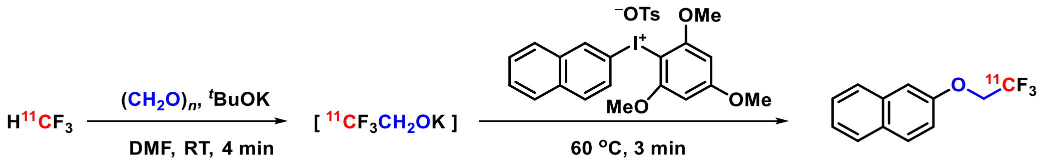

[^{11}C]34

Prepared following general procedure B and analyzed using HPLC condition A.

HPLC condition A

Column: Luna C18(2) (10 μm, 250 × 4.6 mm; i.d. Phenomenex)

Solvent A: H_2O, Solvent B: MeCN

Flow rate: 2 mL/min

0–1 min: 40% B

1–3 min: 40% to 60% B

3–6 min: 60% to 95% B

6–12 min: 95% B

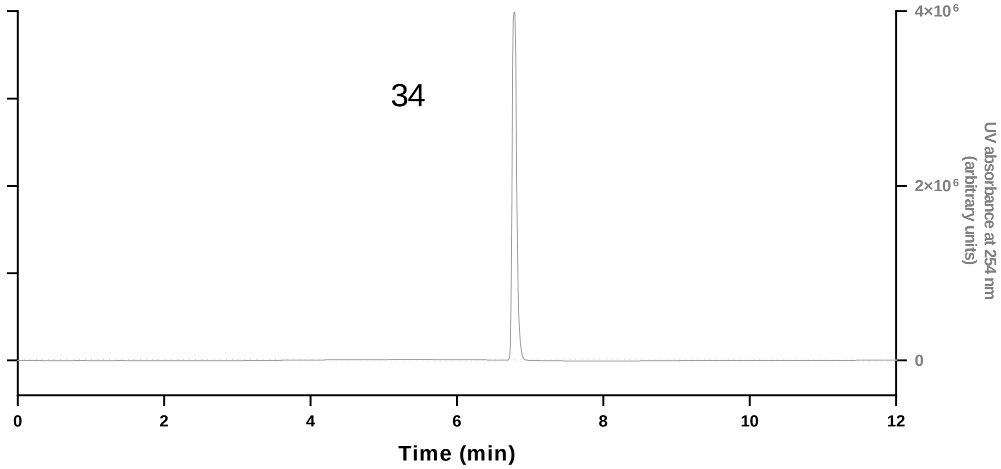

Supplementary Figure 124. Analytical HPLC chromatogram for **34.**

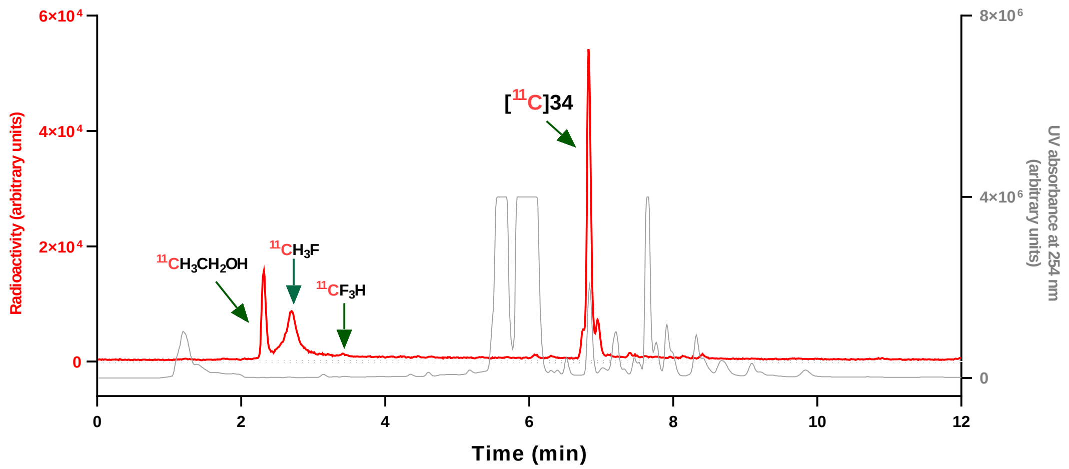

Supplementary Figure 125. Analytical HPLC chromatogram for reaction mixture.

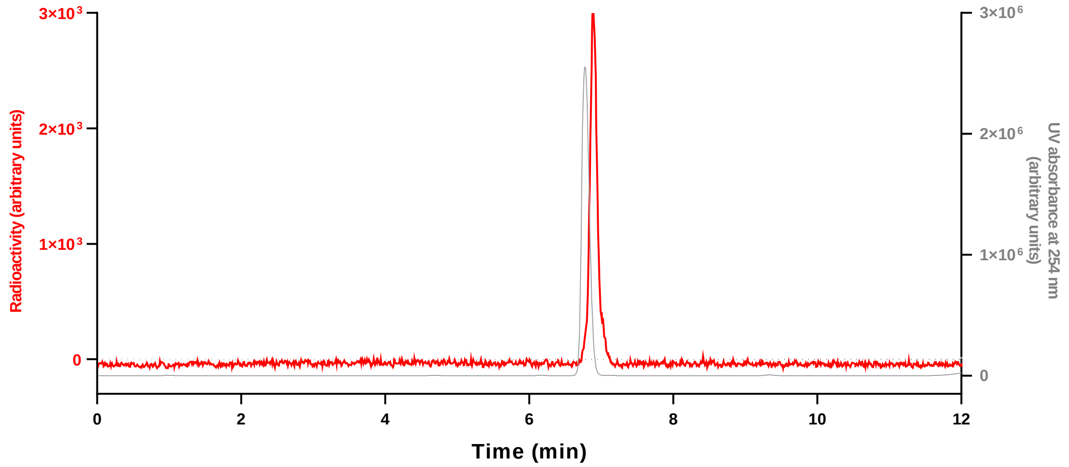

Supplementary Figure 126. Analytical HPLC chromatogram for [^{11}C]**34** collected from the above HPLC analysis when co–injected with **34.**

Run	Retention time (min)	Yield (decay-corrected) (%)	Mean yield ± SD (%)	Isolated yield (%)
1	6.827	69	68 ± 4	69
2	6.809	64		
3	6.899	71		

HPLC analysis of [^{11}C]35

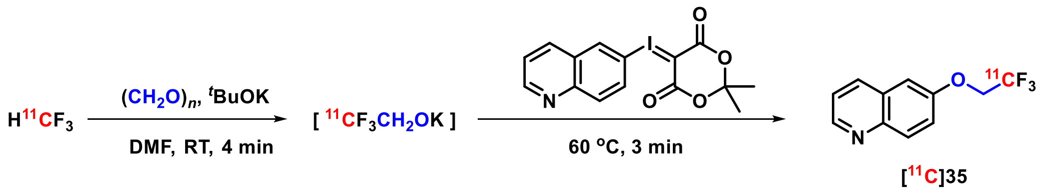

Prepared following general procedure B and analyzed using HPLC condition A.

HPLC condition A

Column: Luna C18(2) (10 μm, 250 × 4.6 mm; i.d. Phenomenex)

Solvent A: H_2O, Solvent B: MeCN

Flow rate: 2 mL/min

0–1 min: 40% B

1–3 min: 40% to 60% B

3–6 min: 60% to 95% B

6–12 min: 95% B

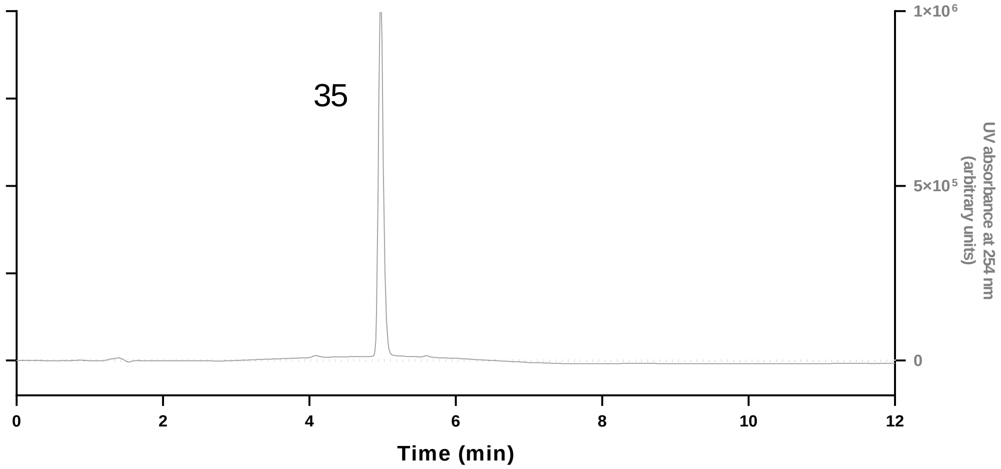

Supplementary Figure 127. Analytical HPLC chromatogram for **35.**

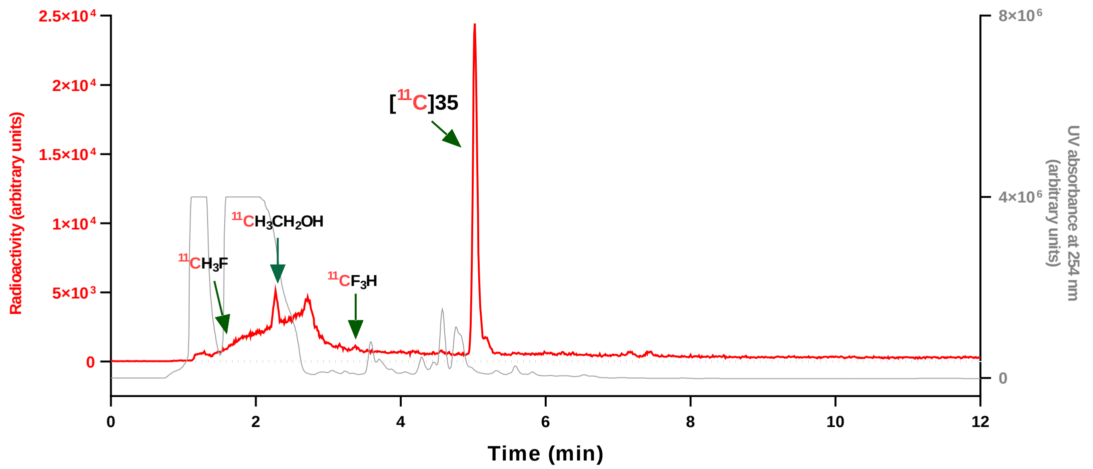

Supplementary Figure 128. Analytical HPLC chromatogram for reaction mixture.

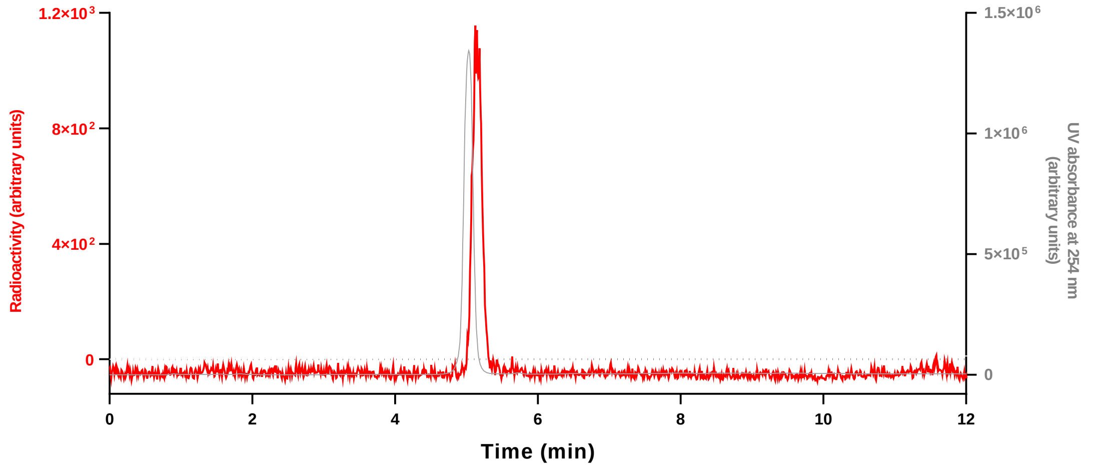

Supplementary Figure 129. Analytical HPLC chromatogram for [^{11}C]**35** collected from the above HPLC analysis when co–injected with **35.**

Run	Retention time (min)	Yield (decay-corrected) (%)	Mean yield ± SD (%)	Isolated yield (%)
1	5.024	75	73 ± 2	67
2	5.027	73		
3	5.027	71		

HPLC analysis of [^{11}C]36

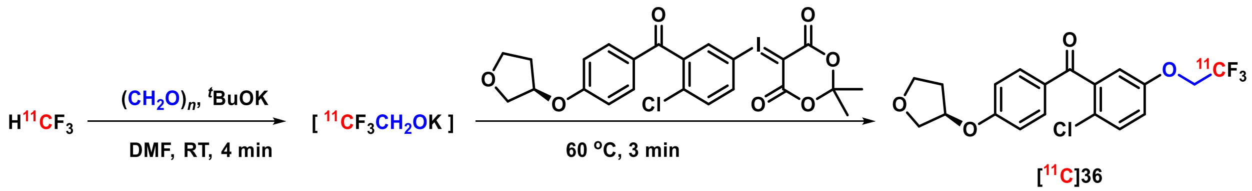

Prepared following general procedure B and analyzed using HPLC condition A.

HPLC condition A

Column: Luna C18(2) (10 μm, 250 × 4.6 mm; i.d. Phenomenex)

Solvent A: H_2O, Solvent B: MeCN

Flow rate: 2 mL/min

0–1 min: 40% B

1–3 min: 40% to 60% B

3–6 min: 60% to 95% B

6–12 min: 95% B

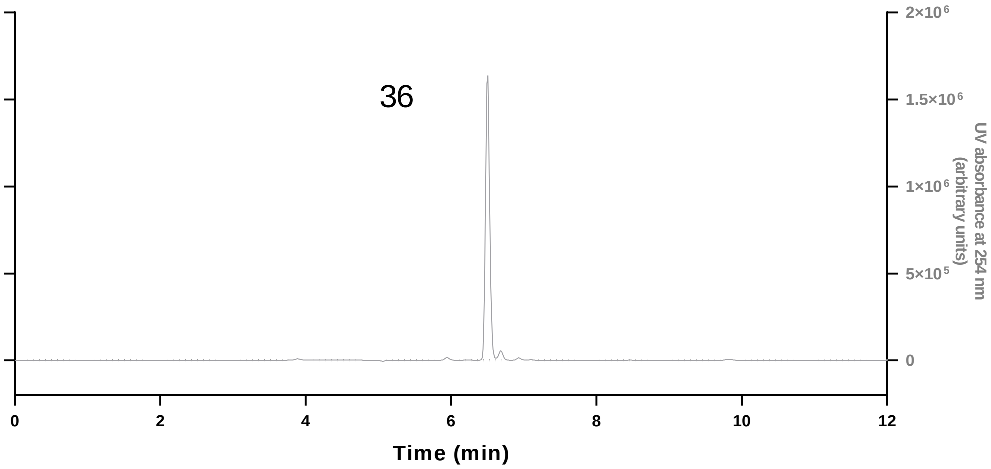

Supplementary Figure 130. Analytical HPLC chromatogram for **36.**

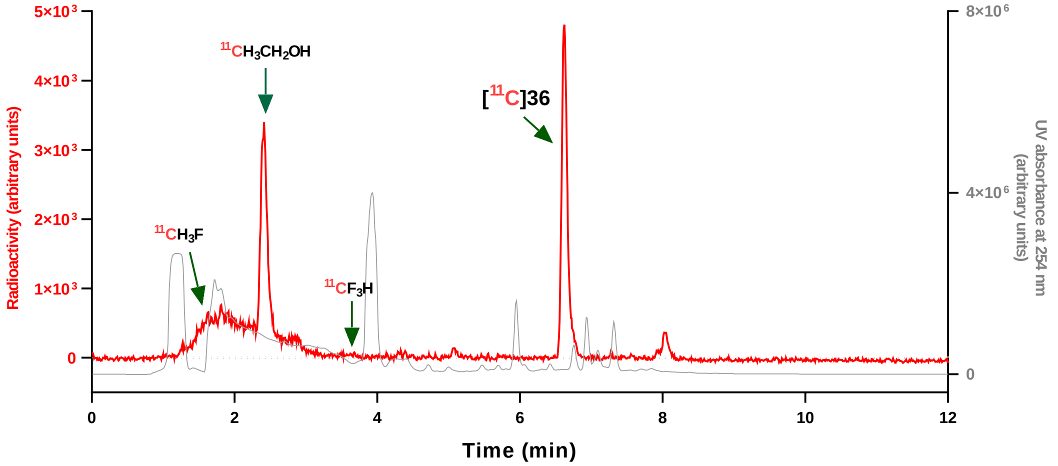

Supplementary Figure 131. Analytical HPLC chromatogram for reaction mixture.

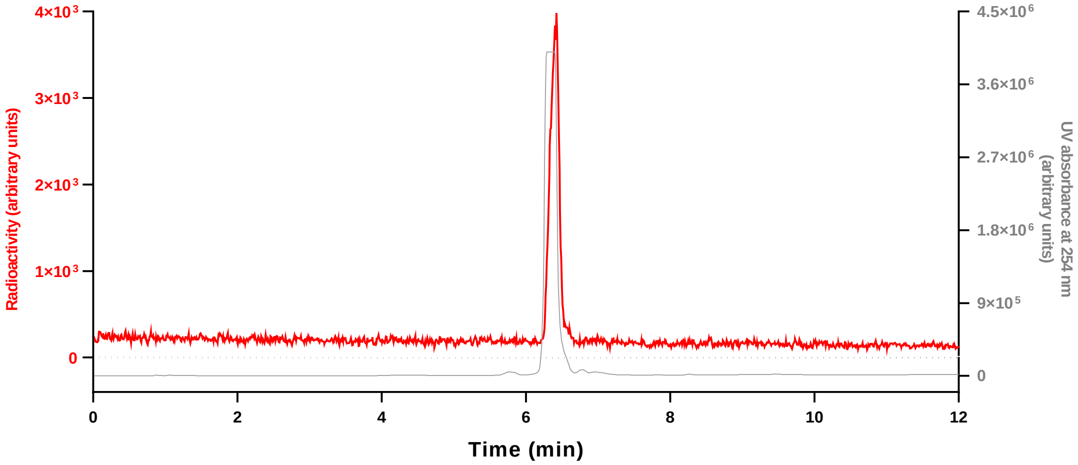

Supplementary Figure 132. Analytical HPLC chromatogram for [11C]**36** collected from the above HPLC analysis when co–injected with **36.**

Run	Retention time (min)	Yield (decay-corrected) (%)	Mean yield ± SD (%)	Isolated yield (%)
1	6.621	46	47 ± 2	42
2	6.617	46		
3	6.623	49		

HPLC analysis of [^{11}C]37

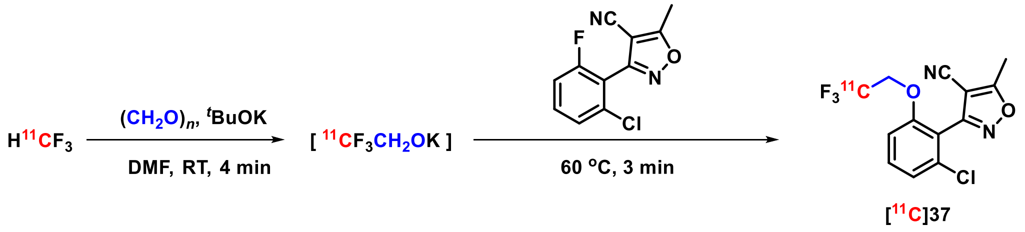

Prepared following general procedure B and analyzed using HPLC condition A.

HPLC condition A

Column: Luna C18(2) (10 μm, 250 × 4.6 mm; i.d. Phenomenex)

Solvent A: H_2O, Solvent B: MeCN

Flow rate: 2 mL/min

0–1 min: 40% B

1–3 min: 40% to 60% B

3–6 min: 60% to 95% B

6–12 min: 95% B

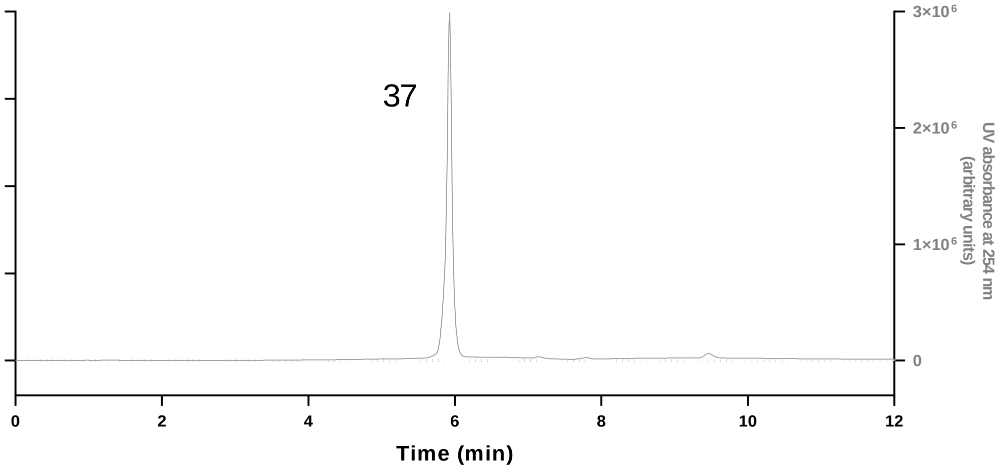

Supplementary Figure 133. Analytical HPLC chromatogram for **37.**

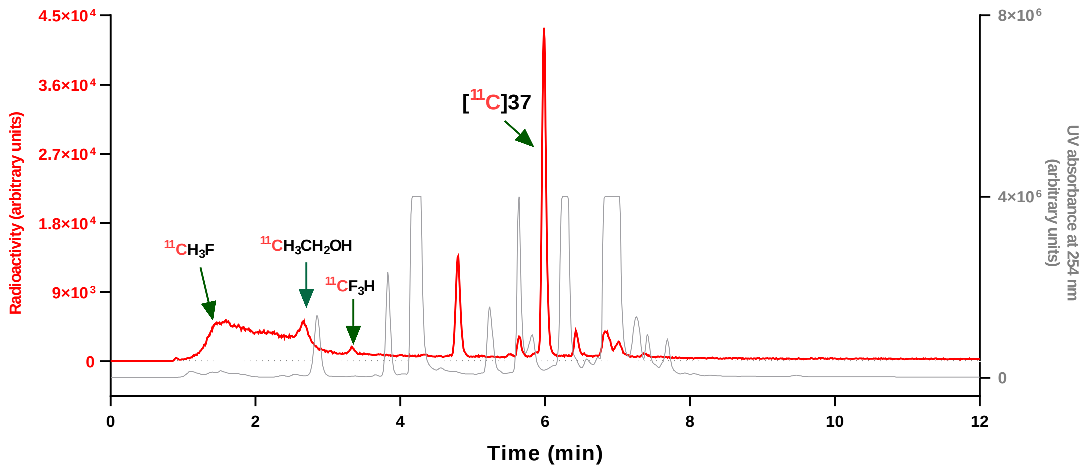

Supplementary Figure 134. Analytical HPLC chromatogram for reaction mixture.

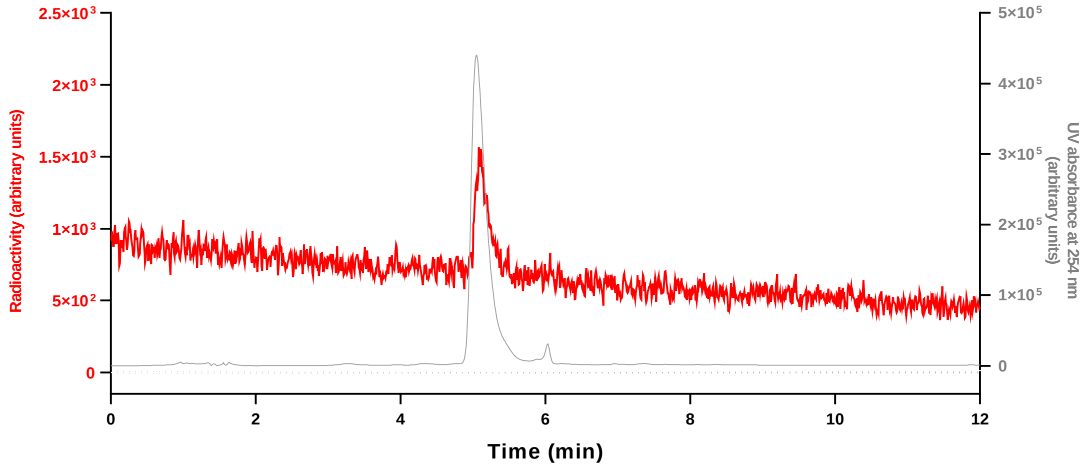

Supplementary Figure 135. Analytical HPLC chromatogram for [^{11}C]**37** collected from the above HPLC analysis when co–injected with **37.**

Run	Retention time (min)	Yield (decay-corrected) (%)	Mean yield ± SD (%)	Isolated yield (%)
1	5.985	49	44 ± 10	36
2	6.082	33		
3	6.076	51		

HPLC analysis of [^{11}C]38

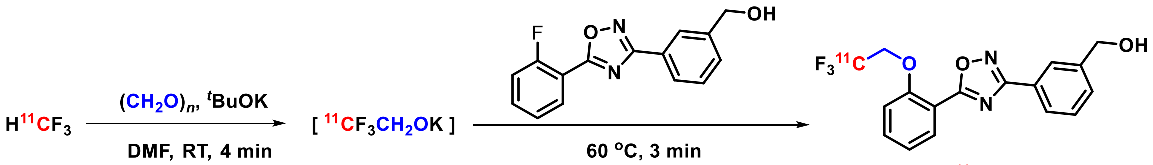

Prepared following general procedure B and analyzed using HPLC condition A.

HPLC condition A

Column: Luna C18(2) (10 μm, 250 × 4.6 mm; i.d. Phenomenex)

Solvent A: H_2O, Solvent B: MeCN

Flow rate: 2 mL/min

0–1 min: 40% B

1–3 min: 40% to 60% B

3–6 min: 60% to 95% B

6–12 min: 95% B

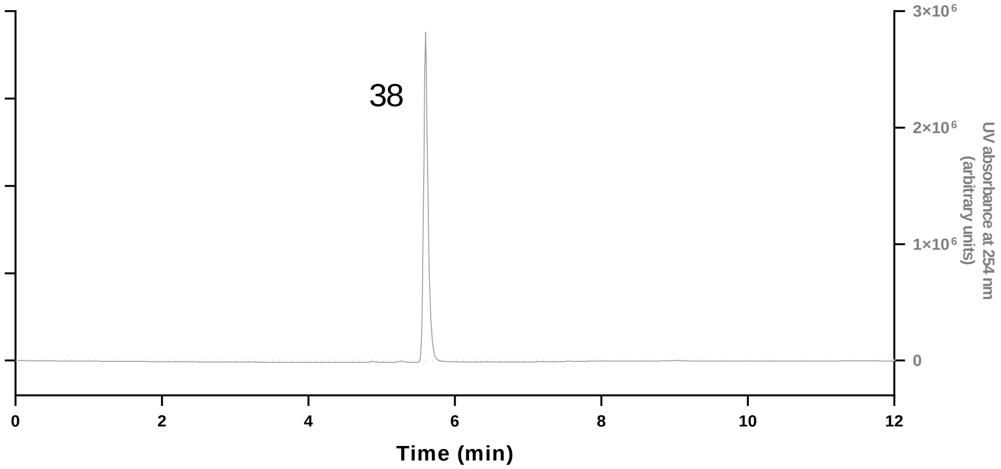

Supplementary Figure 136. Analytical HPLC chromatogram for **38.**

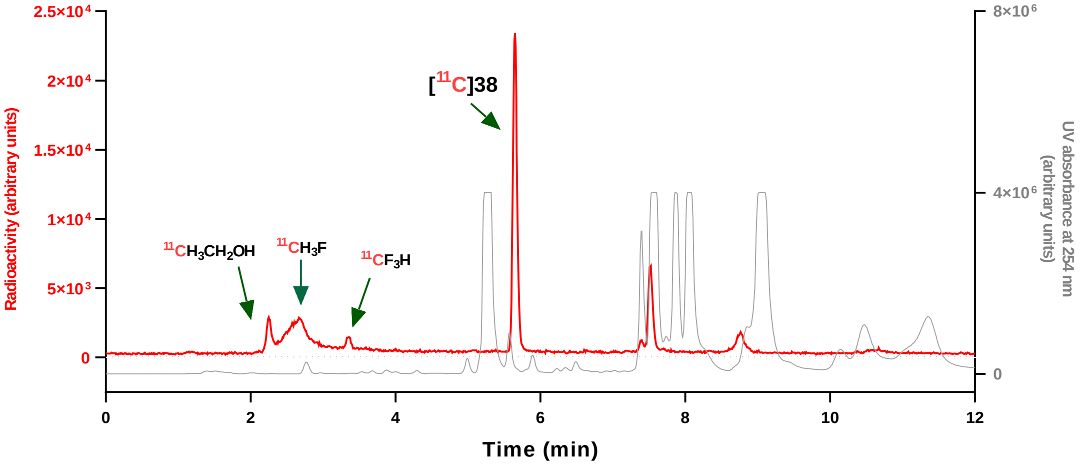

Supplementary Figure 137. Analytical HPLC chromatogram for reaction mixture.

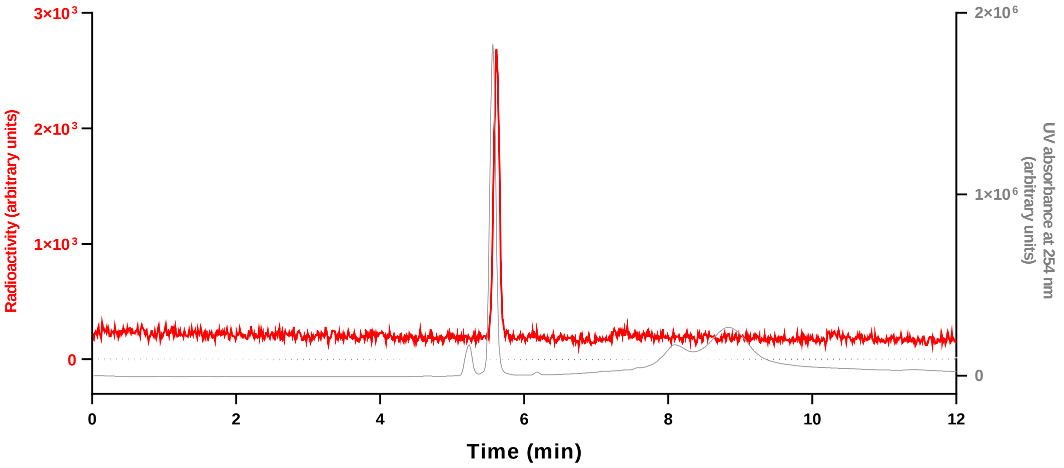

Supplementary Figure 138. Analytical HPLC chromatogram for [^{11}C]**38** collected from the above HPLC analysis when co–injected with **38.**

Run	Retention time (min)	Yield (decay-corrected) (%)	Mean yield ± SD (%)	Isolated yield (%)
1	5.649	60	60 ± 1	57
2	5.737	61		
3	5.756	60		

HPLC analysis of [^{11}C]39

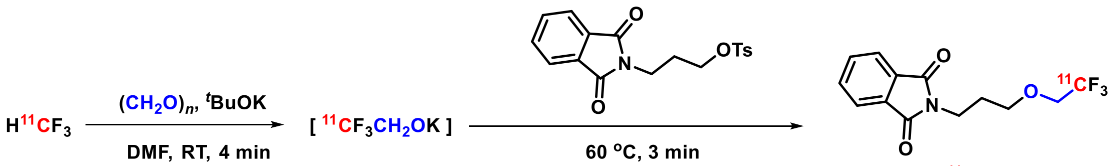

Prepared following general procedure B and analyzed using HPLC condition A.

HPLC condition A

Column: Luna C18(2) (10 μm, 250 × 4.6 mm; i.d. Phenomenex)

Solvent A: H_2O, Solvent B: MeCN

Flow rate: 2 mL/min

0–1 min: 40% B

1–3 min: 40% to 60% B

3–6 min: 60% to 95% B

6–12 min: 95% B

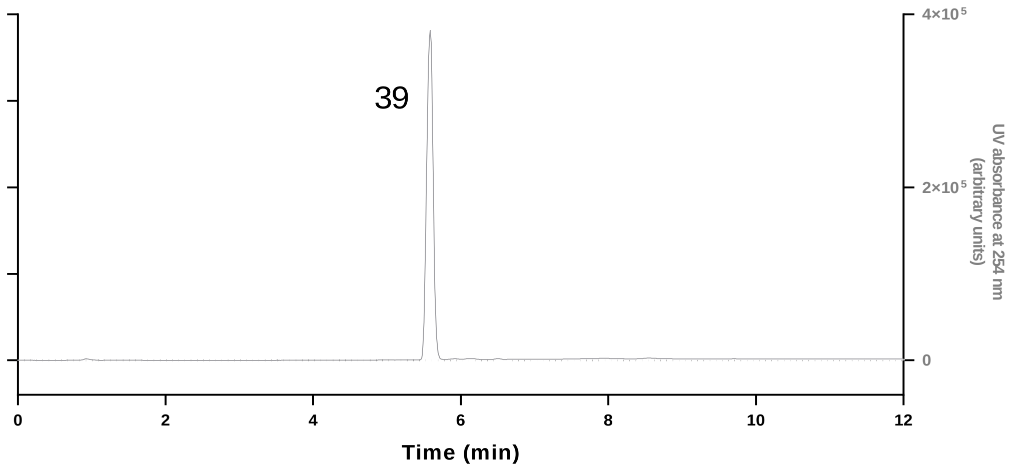

Supplementary Figure 139. Analytical HPLC chromatogram for **39.**

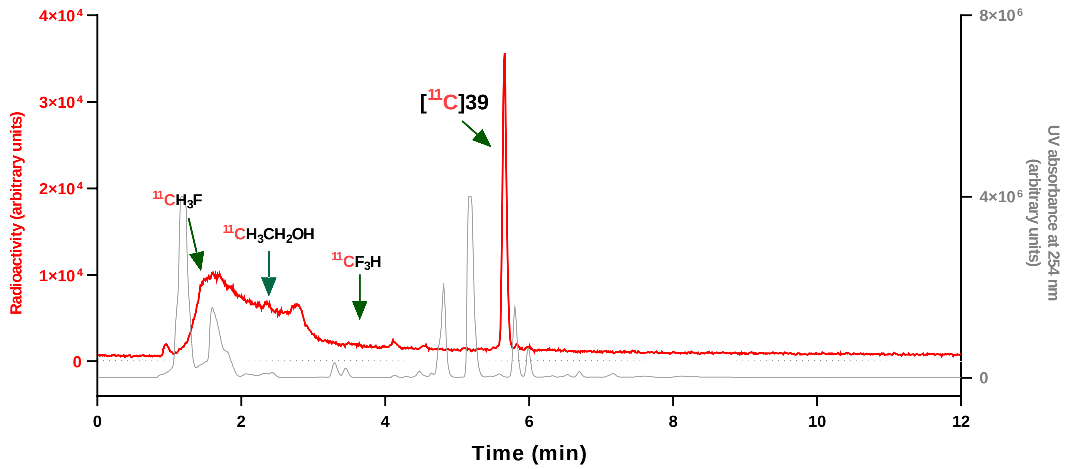

Supplementary Figure 140. Analytical HPLC chromatogram for reaction mixture.

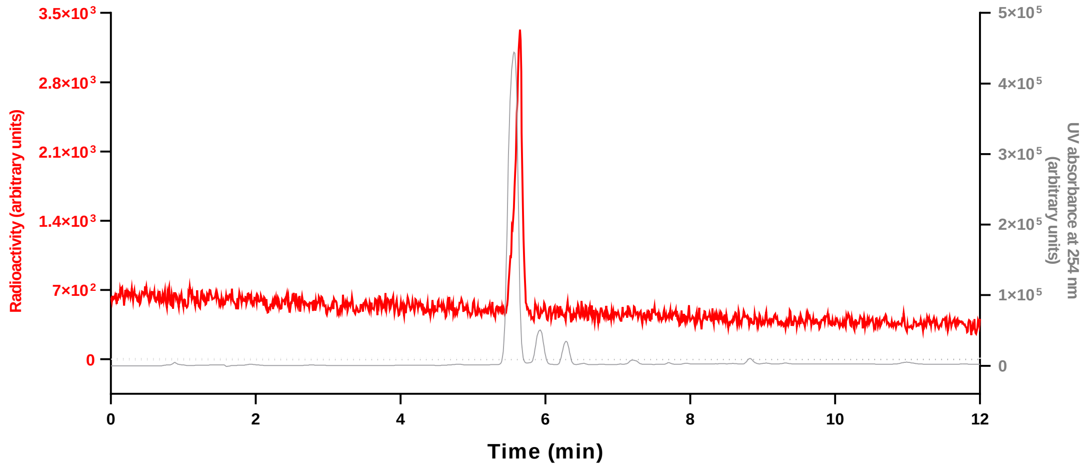

Supplementary Figure 141. Analytical HPLC chromatogram for [^{11}C]**39** collected from the above HPLC analysis when co–injected with **39.**

Run	Retention time (min)	Yield (decay-corrected) (%)	Mean yield ± SD (%)	Isolated yield (%)
1	5.656	34	31 ± 3	26
2	5.655	31		
3	5.646	29		

HPLC analysis of [^{11}C]40

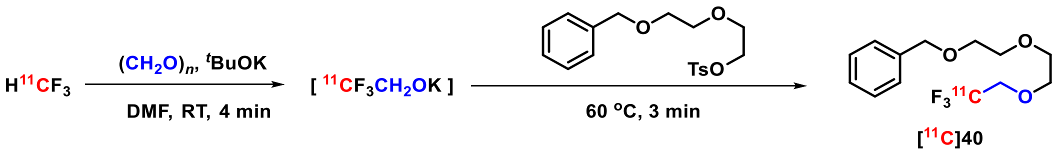

Prepared following general procedure B and analyzed using HPLC condition A.

HPLC condition A

Column: Luna C18(2) (10 μm, 250 × 4.6 mm; i.d. Phenomenex)

Solvent A: H_2O, Solvent B: MeCN

Flow rate: 2 mL/min

0–1 min: 40% B

1–3 min: 40% to 60% B

3–6 min: 60% to 95% B

6–12 min: 95% B

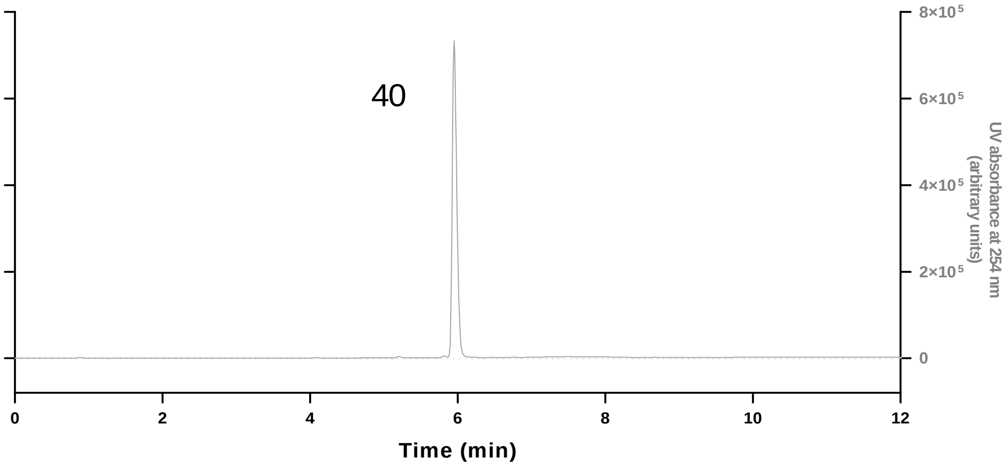

Supplementary Figure 142. Analytical HPLC chromatogram for **40.**

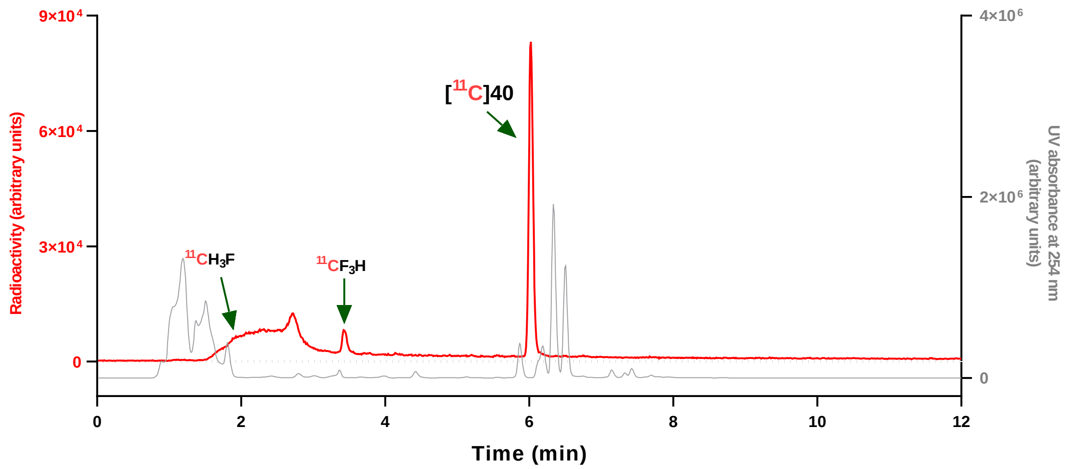

Supplementary Figure 143. Analytical HPLC chromatogram for reaction mixture.

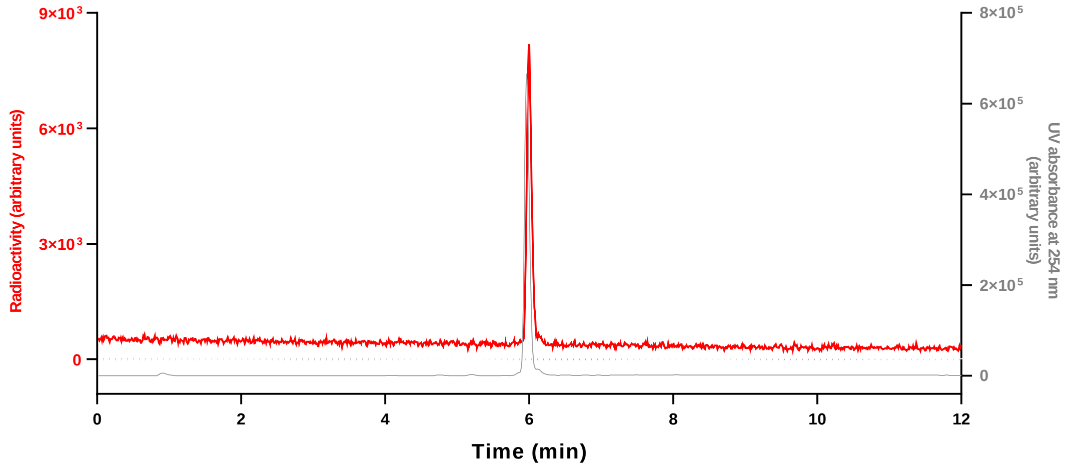

Supplementary Figure 144. Analytical HPLC chromatogram for [^{11}C]**40** collected from the above HPLC analysis when co–injected with **40.**

Run	Retention time (min)	Yield (decay-corrected) (%)	Mean yield ± SD (%)	Isolated yield (%)
1	6.023	90	89 ± 4	78
2	6.028	92		
3	6.046	85		

HPLC analysis of [^{11}C]41

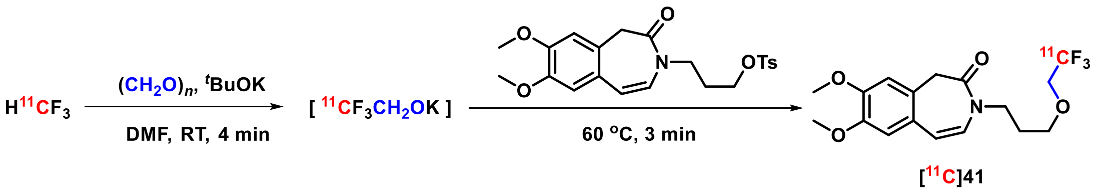

Prepared following general procedure B and analyzed using HPLC condition A.

HPLC condition A

Column: Luna C18(2) (10 μm, 250 × 4.6 mm; i.d. Phenomenex)

Solvent A: H_2O, Solvent B: MeCN

Flow rate: 2 mL/min

0–1 min: 40% B

1–3 min: 40% to 60% B

3–6 min: 60% to 95% B

6–12 min: 95% B

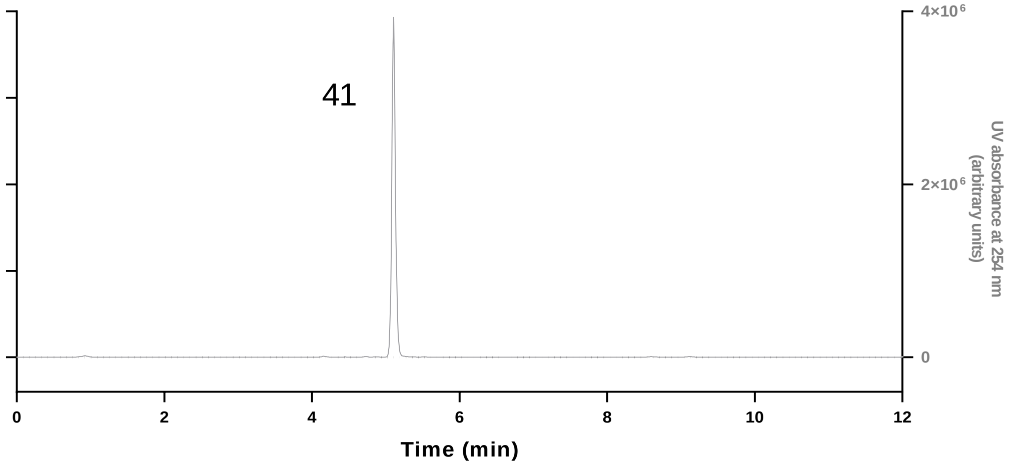

Supplementary Figure 145. Analytical HPLC chromatogram for **41.**

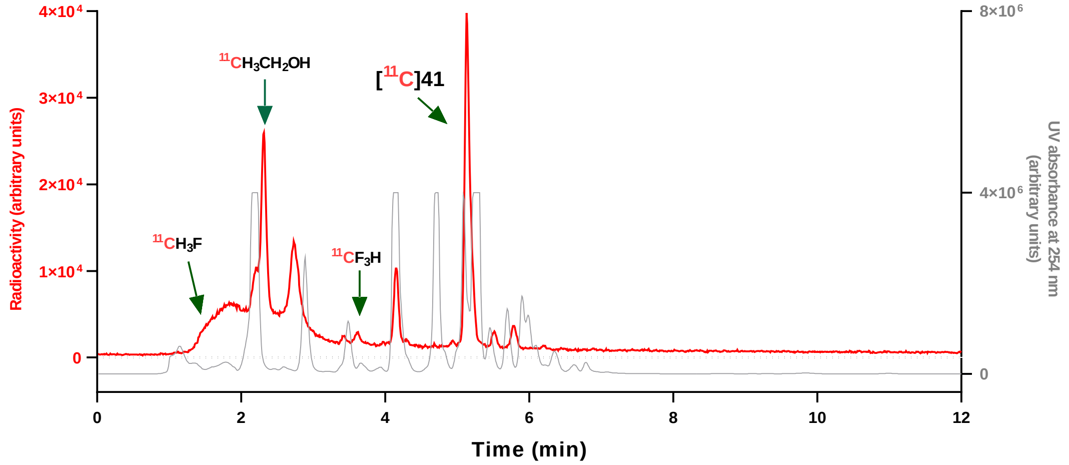

Supplementary Figure 146. Analytical HPLC chromatogram for reaction mixture.

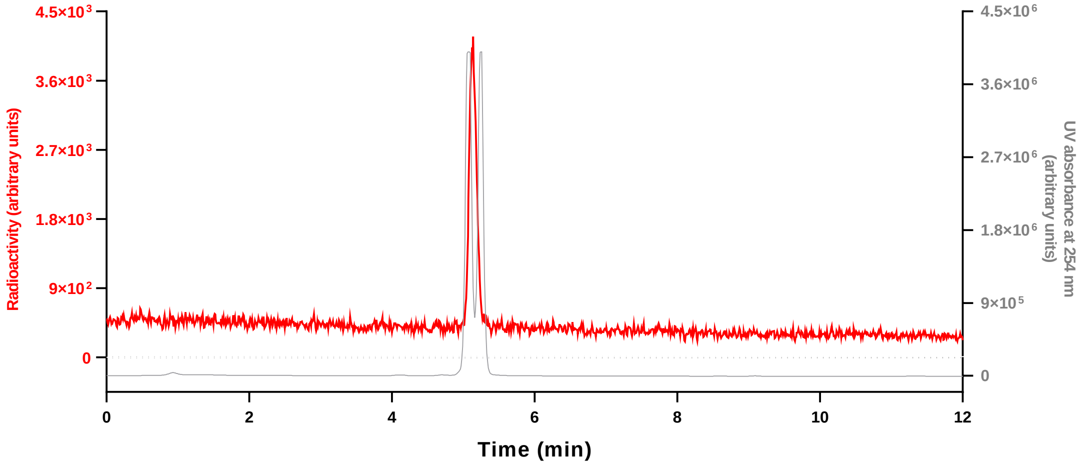

Supplementary Figure 147. Analytical HPLC chromatogram for [^{11}C]**41** collected from the above HPLC analysis when co–injected with **41.**

Run	Retention time (min)	Yield (decay-corrected) (%)	Mean yield ± SD (%)	Isolated yield (%)
1	5.136	46	45 ± 1	41
2	5.134	44		
3	5.133	44		

HPLC analysis of [^{11}C]42

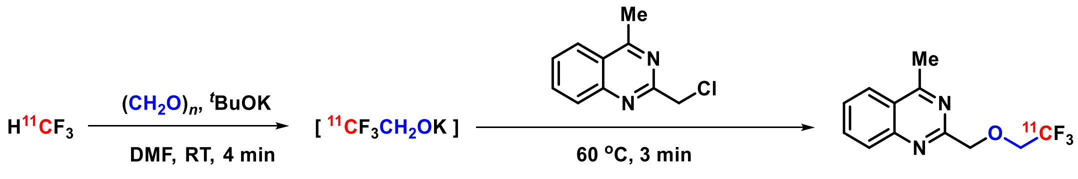

[^{11}C]42

Prepared following general procedure B and analyzed using HPLC condition A.

HPLC condition A

Column: Luna C18(2) (10 μm, 250 × 4.6 mm; i.d. Phenomenex)

Solvent A: H_2O, Solvent B: MeCN

Flow rate: 2 mL/min

0–1 min: 40% B

1–3 min: 40% to 60% B

3–6 min: 60% to 95% B

6–12 min: 95% B

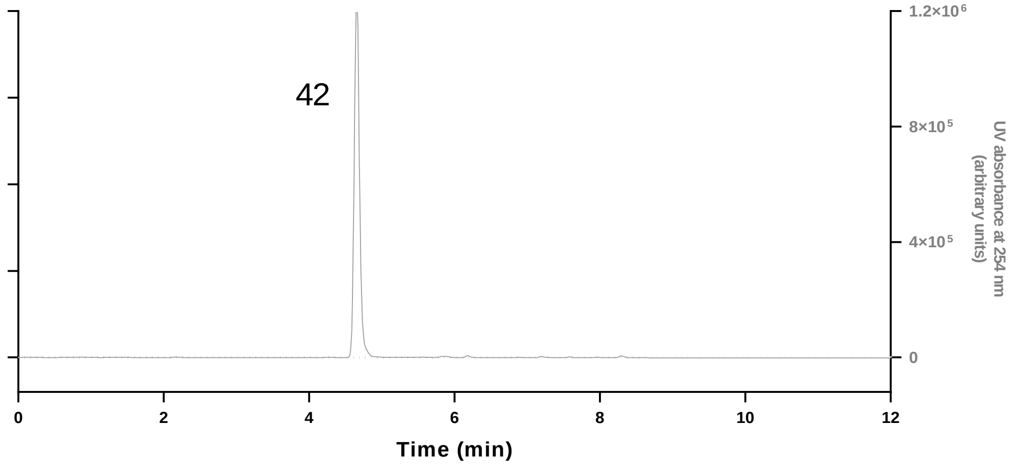

Supplementary Figure 148. Analytical HPLC chromatogram for **42.**

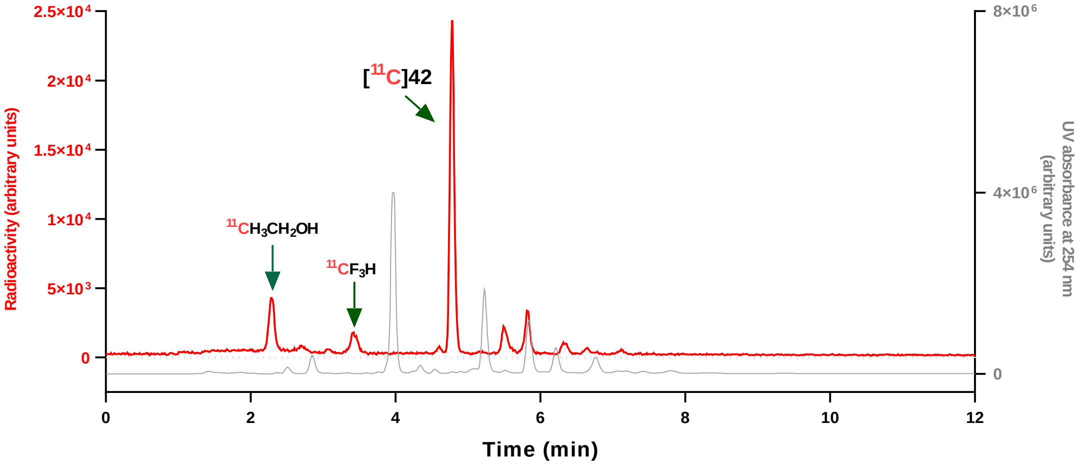

Supplementary Figure 149. Analytical HPLC chromatogram for reaction mixture.

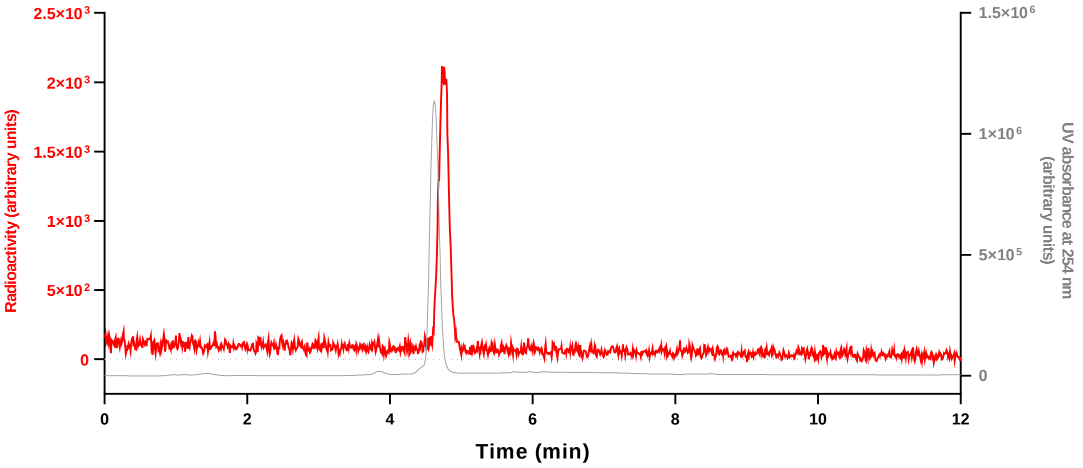

Supplementary Figure 150. Analytical HPLC chromatogram for [^{11}C]**42** collected from the above HPLC analysis when co–injected with **42.**

Run	Retention time (min)	Yield (decay-corrected) (%)	Mean yield ± SD (%)	Isolated yield (%)
1	4.782	57	53 ± 4	60
2	4.782	54		
3	4.786	49		

HPLC analysis of [^{11}C]43

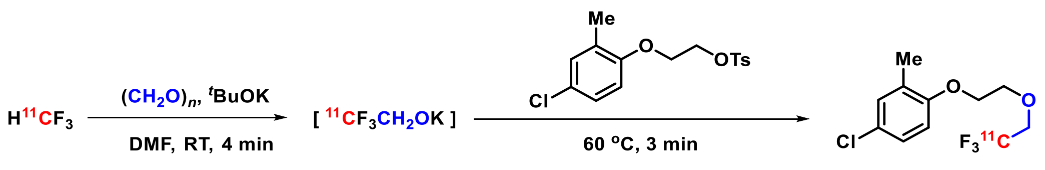

Prepared following general procedure B and analyzed using HPLC condition A.

HPLC condition A

Column: Luna C18(2) (10 μm, 250 × 4.6 mm; i.d. Phenomenex)

Solvent A: H_2O, Solvent B: MeCN

Flow rate: 2 mL/min

0–1 min: 40% B

1–3 min: 40% to 60% B

3–6 min: 60% to 95% B

6–12 min: 95% B

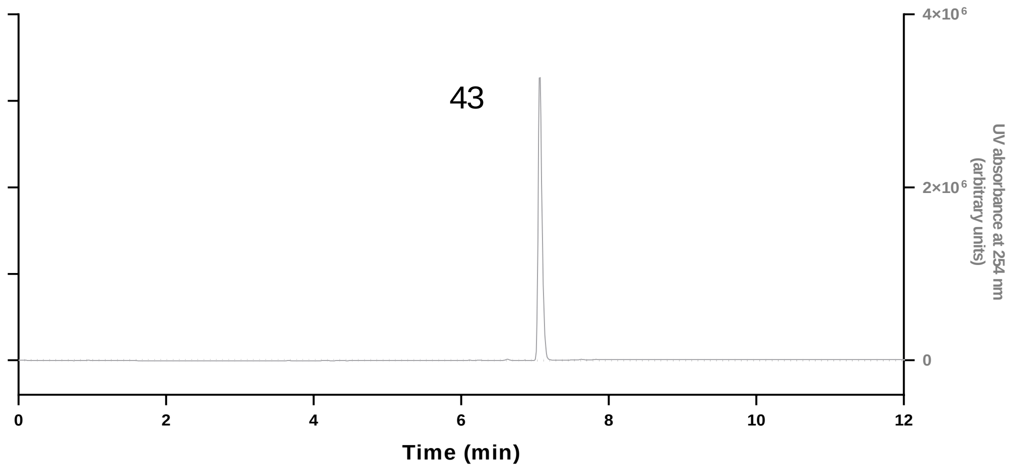

Supplementary Figure 151. Analytical HPLC chromatogram for **43.**

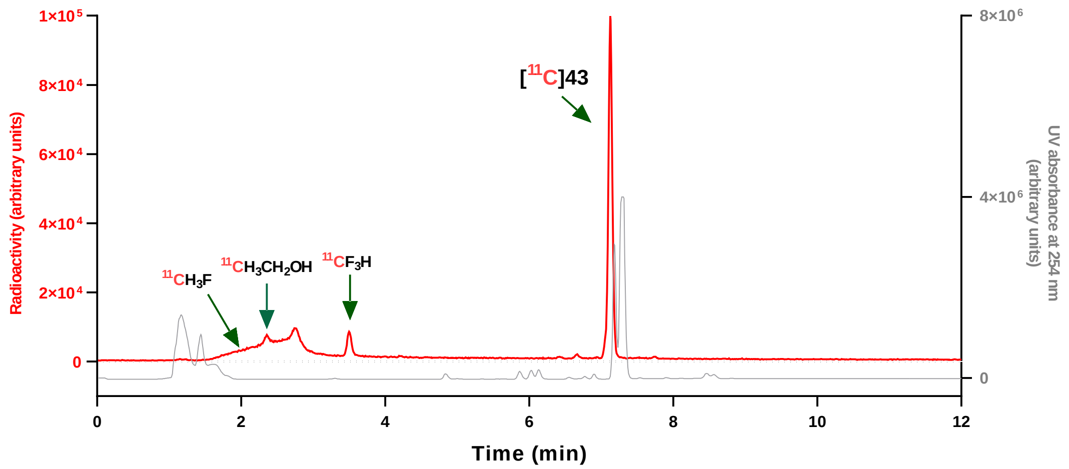

Supplementary Figure 152. Analytical HPLC chromatogram for reaction mixture.

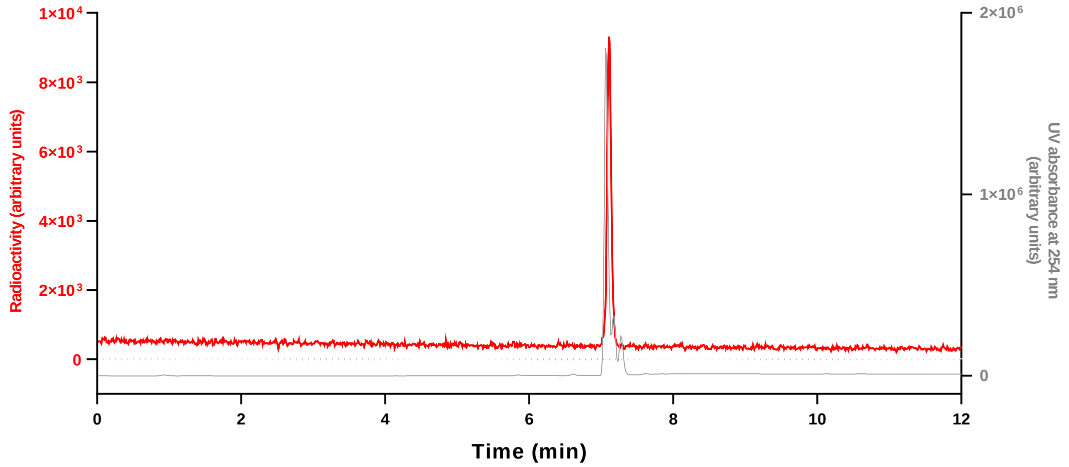

Supplementary Figure 153. Analytical HPLC chromatogram for [^{11}C]**43** collected from the above HPLC analysis when co–injected with **43.**

Run	Retention time (min)	Yield (decay-corrected) (%)	Mean yield ± SD (%)	Isolated yield (%)
1	7.127	86	85 ± 1	74
2	7.128	85		
3	7.132	85		

HPLC analysis of [^{11}C]44

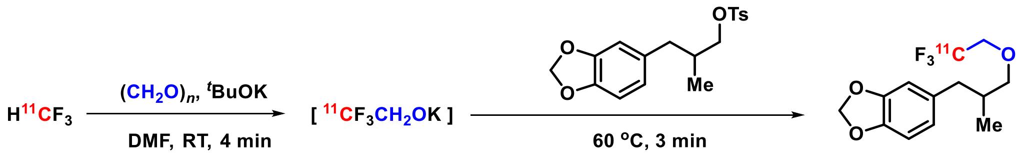

[^{11}C]44

Prepared following general procedure B and analyzed using HPLC condition A.

HPLC condition A

Column: Luna C18(2) (10 μm, 250 × 4.6 mm; i.d. Phenomenex)

Solvent A: H_2O, Solvent B: MeCN

Flow rate: 2 mL/min

0–1 min: 40% B

1–3 min: 40% to 60% B

3–6 min: 60% to 95% B

6–12 min: 95% B

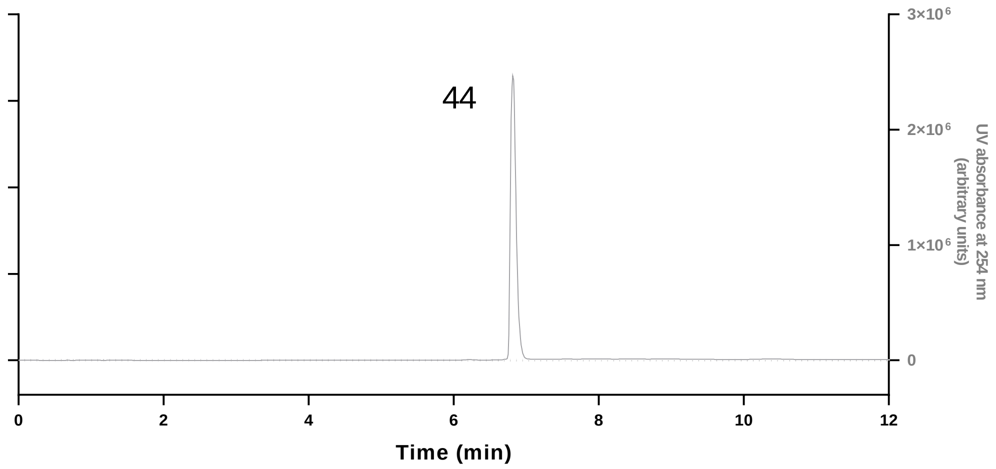

Supplementary Figure 154. Analytical HPLC chromatogram for **44.**

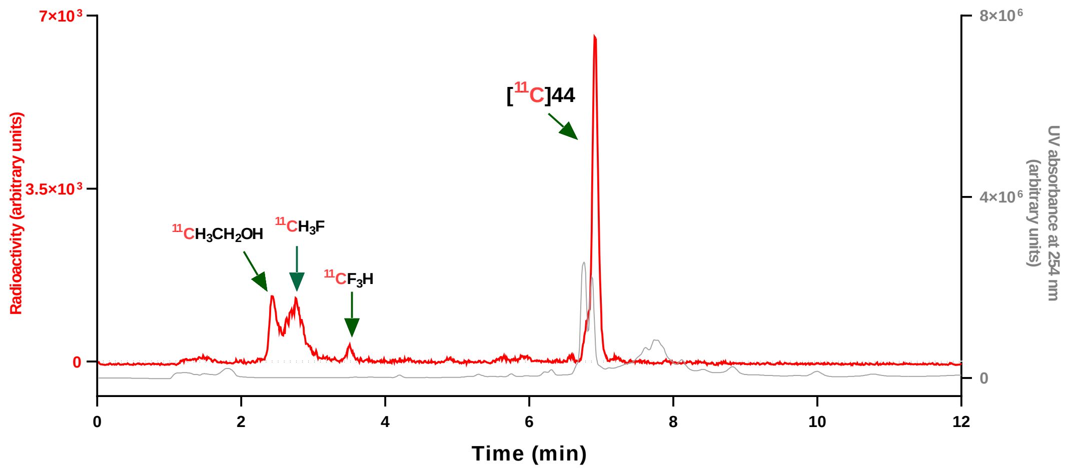

Supplementary Figure 155. Analytical HPLC chromatogram for reaction mixture.

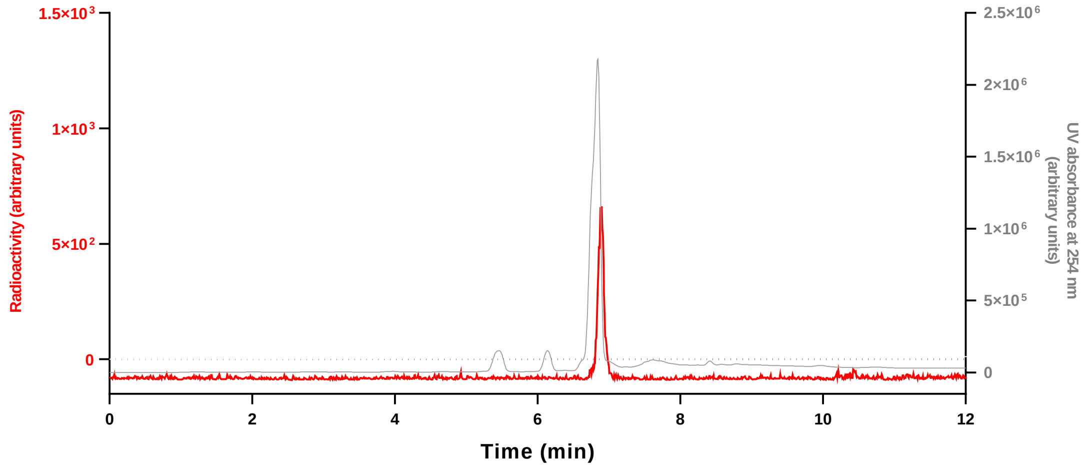

Supplementary Figure 156. Analytical HPLC chromatogram for [^{11}C]**44** collected from the above HPLC analysis when co–injected with **44.**

Run	Retention time (min)	Yield (decay-corrected) (%)	Mean yield ± SD (%)	Isolated yield (%)
1	6.912	60	53 ± 7	44
2	6.866	51		
3	6.854	47		

HPLC analysis of [^{11}C]45

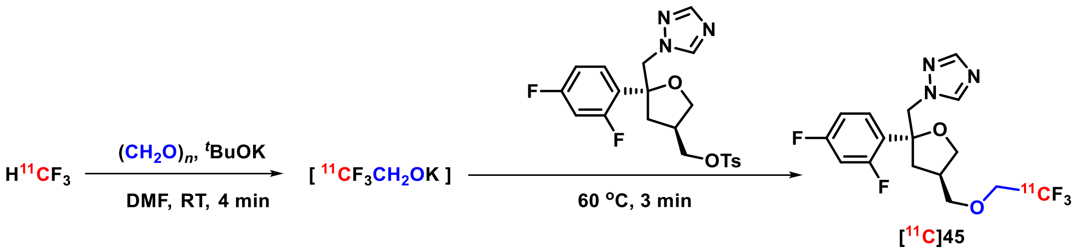

Prepared following general procedure B and analyzed using HPLC condition A.

HPLC condition A

Column: Luna C18(2) (10 μm, 250 × 4.6 mm; i.d. Phenomenex)

Solvent A: H_2O, Solvent B: MeCN

Flow rate: 2 mL/min

0–1 min: 40% B

1–3 min: 40% to 60% B

3–6 min: 60% to 95% B

6–12 min: 95% B

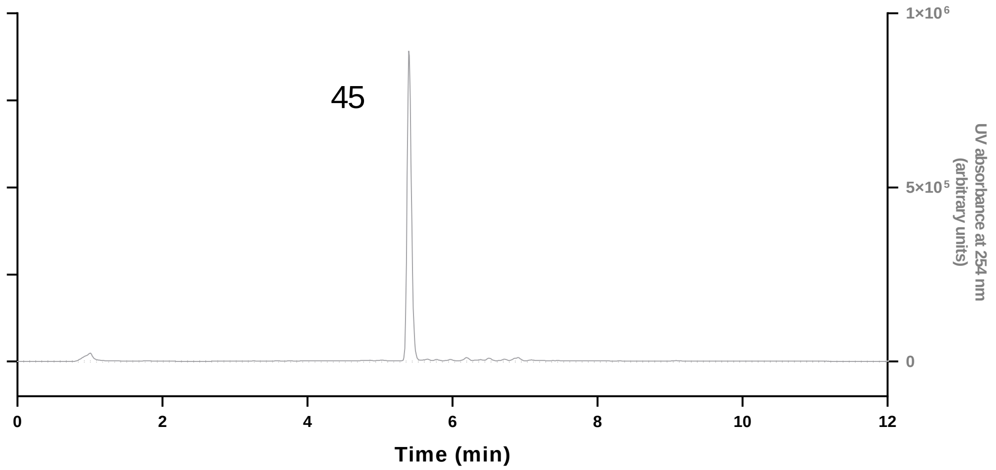

Supplementary Figure 157. Analytical HPLC chromatogram for **45.**

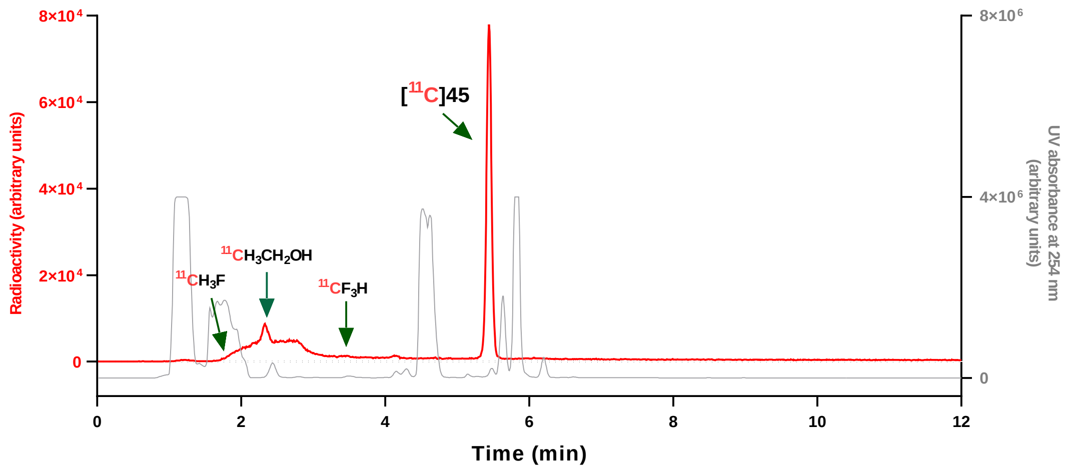

Supplementary Figure 158. Analytical HPLC chromatogram for reaction mixture.

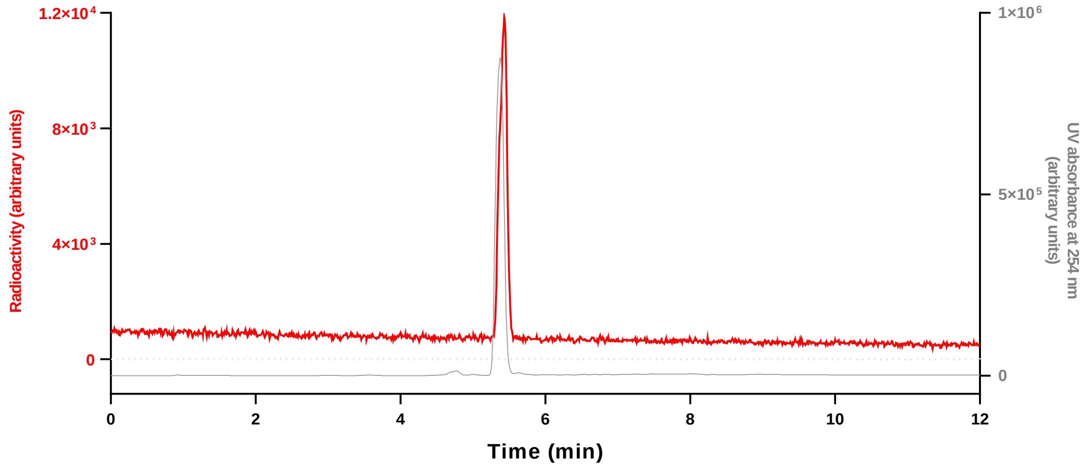

Supplementary Figure 159. Analytical HPLC chromatogram for [^{11}C]**45** collected from the above HPLC analysis when co–injected with **45.**

Run	Retention time (min)	Yield (decay-corrected) (%)	Mean yield ± SD (%)	Isolated yield (%)
1	5.443	91	89 ± 6	90
2	5.528	93		
3	5.459	82		

HPLC analysis of [^{11}C]46

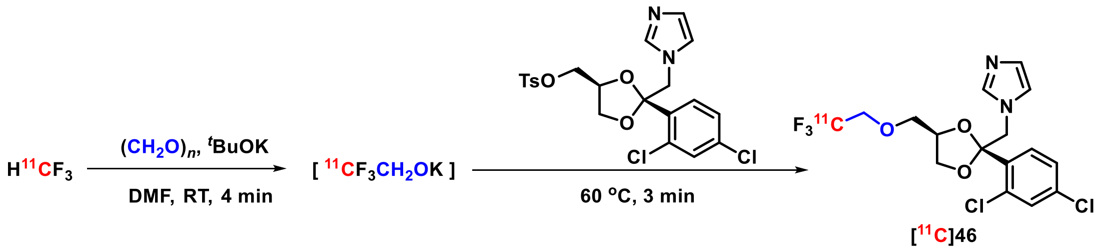

Prepared following general procedure B and analyzed using HPLC condition A.

HPLC condition A

Column: Luna C18(2) (10 μm, 250 × 4.6 mm; i.d. Phenomenex)

Solvent A: H_2O, Solvent B: MeCN

Flow rate: 2 mL/min

0–1 min: 40% B

1–3 min: 40% to 60% B

3–6 min: 60% to 95% B

6–12 min: 95% B

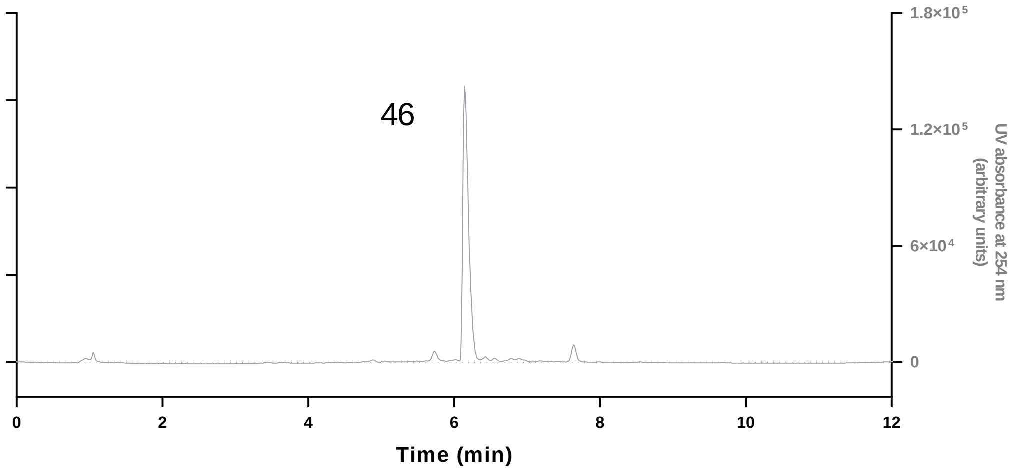

Supplementary Figure 160. Analytical HPLC chromatogram for **46.**

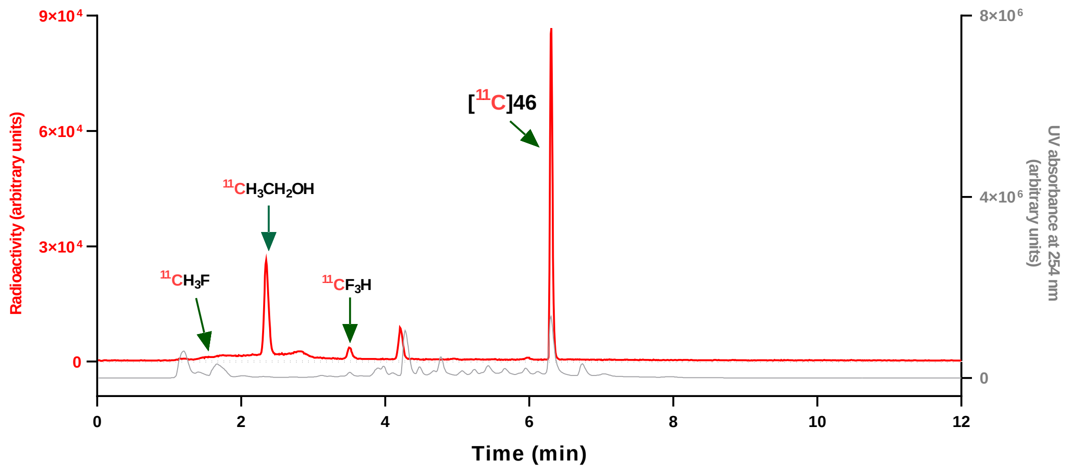

Supplementary Figure 161. Analytical HPLC chromatogram for reaction mixture.

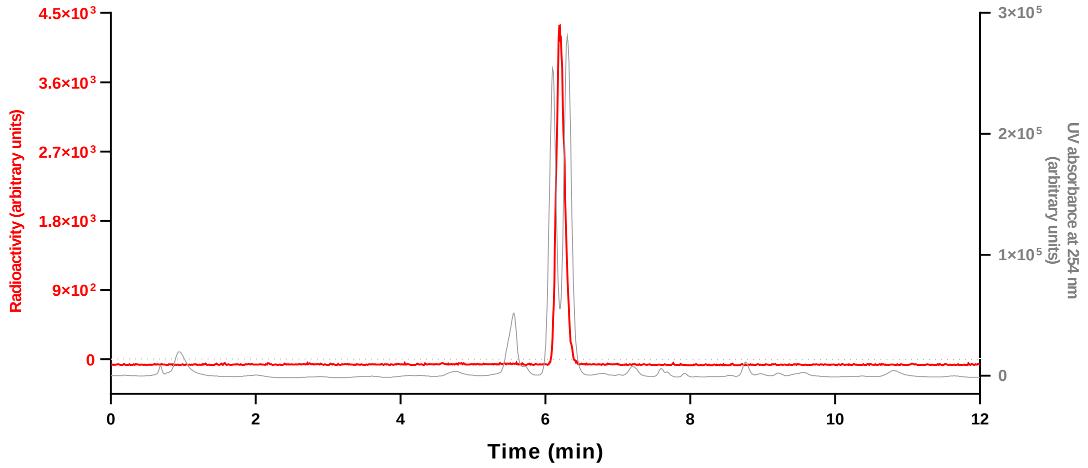

Supplementary Figure 162. Analytical HPLC chromatogram for [^{11}C]**46** collected from the above HPLC analysis when co–injected with **46.**

Run	Retention time (min)	Yield (decay-corrected) (%)	Mean yield ± SD (%)	Isolated yield (%)
1	6.252	50	56 ± 5	52
2	6.305	58		
3	6.286	59		

HPLC analysis of [^{11}C]47

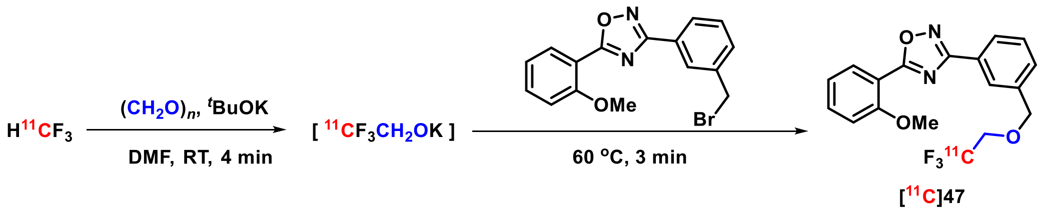

Prepared following general procedure B and analyzed using HPLC condition A.

HPLC condition A

Column: Luna C18(2) (10 μm, 250 × 4.6 mm; i.d. Phenomenex)

Solvent A: H_2O, Solvent B: MeCN

Flow rate: 2 mL/min

0–1 min: 40% B

1–3 min: 40% to 60% B

3–6 min: 60% to 95% B

6–12 min: 95% B

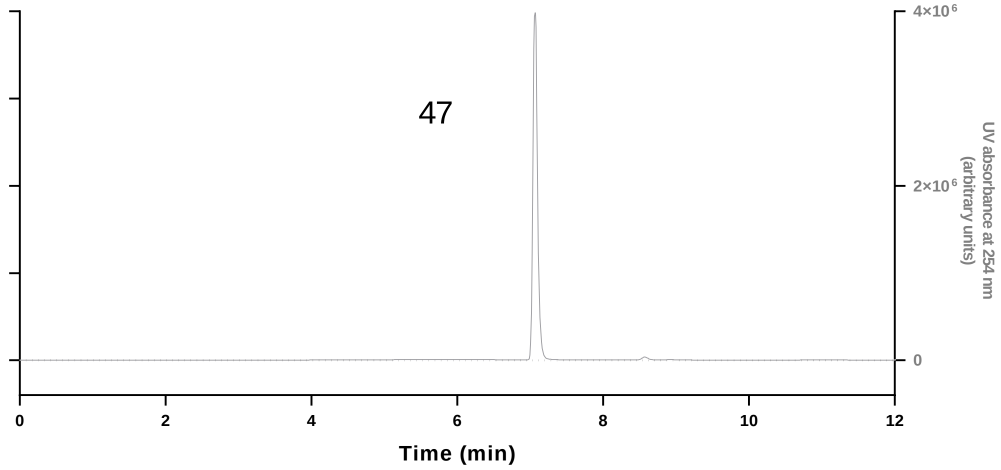

Supplementary Figure 163. Analytical HPLC chromatogram for **47.**

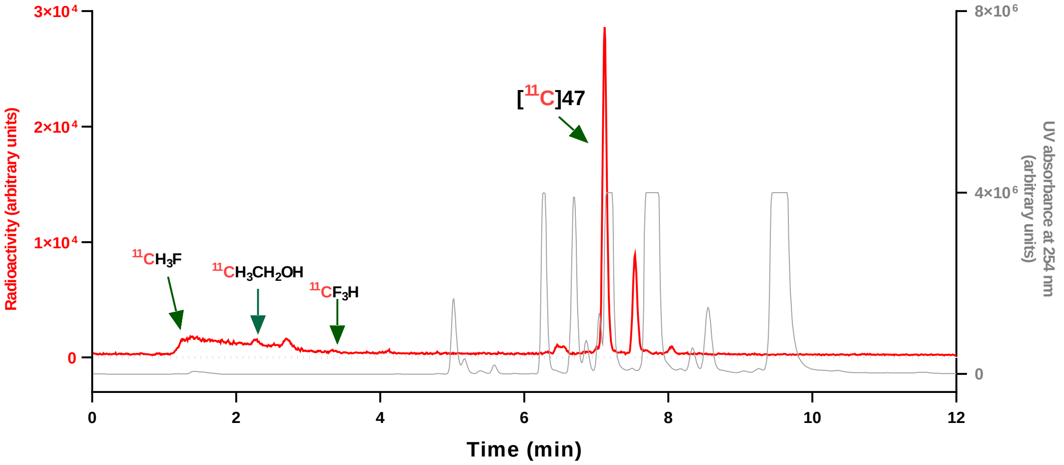

Supplementary Figure 164. Analytical HPLC chromatogram for reaction mixture.

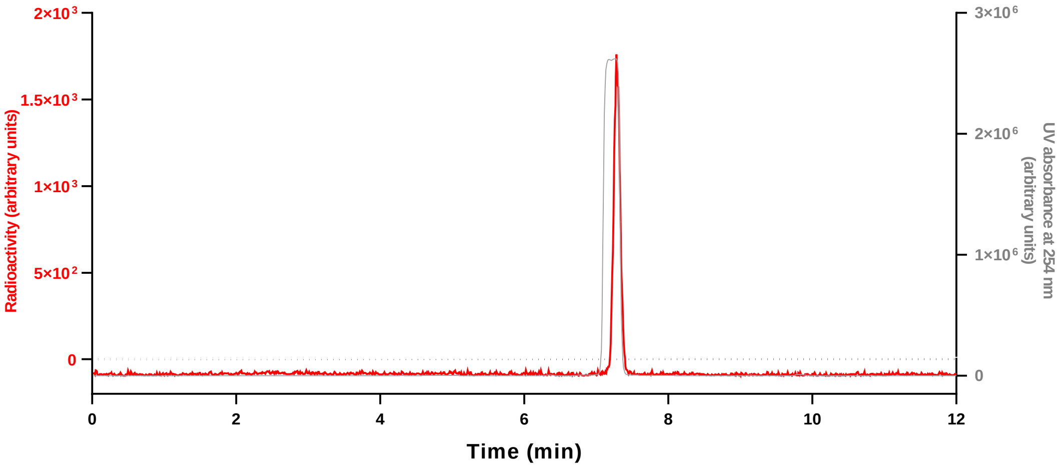

Supplementary Figure 165. Analytical HPLC chromatogram for [^{11}C]**47** collected from the above HPLC analysis when co–injected with **47.**

Run	Retention time (min)	Yield (decay-corrected) (%)	Mean yield ± SD (%)	Isolated yield (%)
1	7.117	71	71 ± 1	64
2	7.117	72		
3	7.110	70		

HPLC analysis of [^{11}C]48

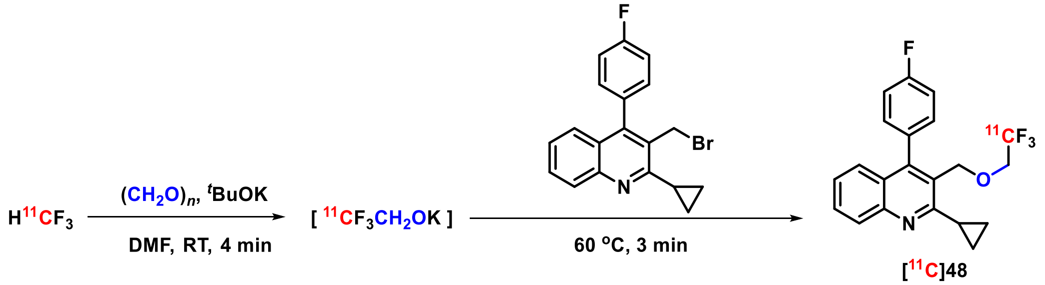

Prepared following general procedure B and analyzed using HPLC condition A.

HPLC condition A

Column: Luna C18(2) (10 μm, 250 × 4.6 mm; i.d. Phenomenex)

Solvent A: H_2O, Solvent B: MeCN

Flow rate: 2 mL/min

0–1 min: 40% B

1–3 min: 40% to 60% B

3–6 min: 60% to 95% B

6–12 min: 95% B

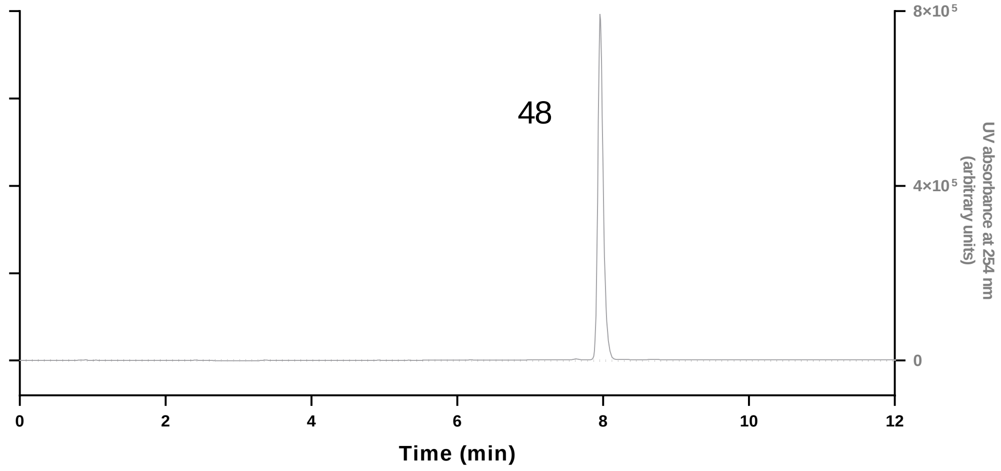

Supplementary Figure 166. Analytical HPLC chromatogram for **48.**

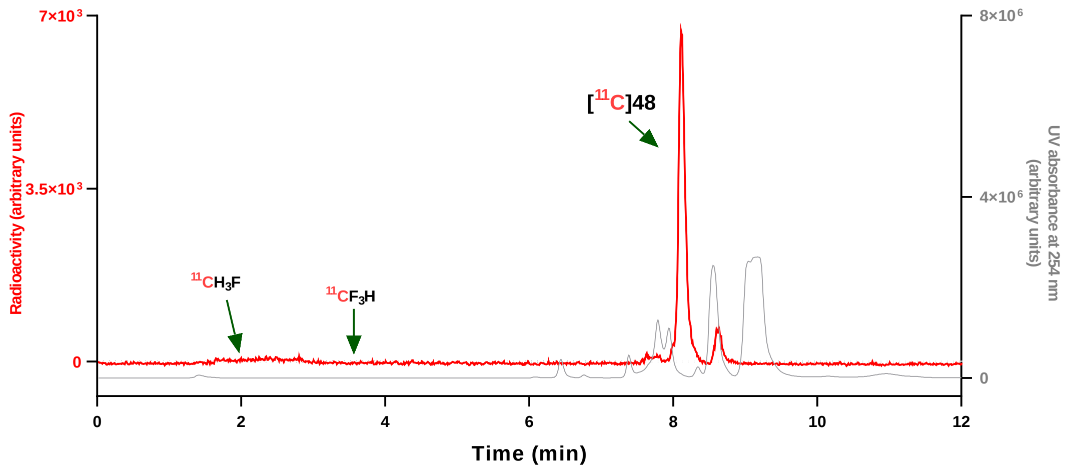

Supplementary Figure 167. Analytical HPLC chromatogram for reaction mixture.

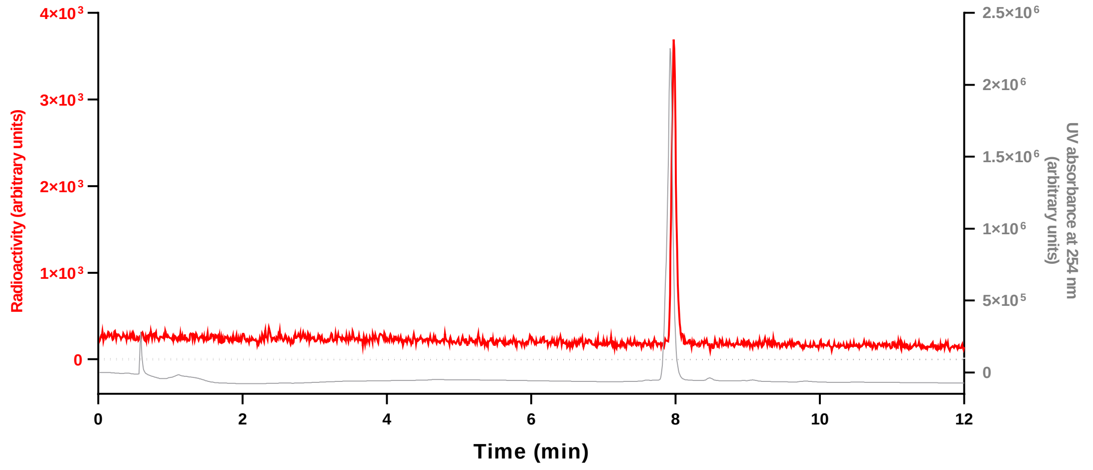

Supplementary Figure 168. Analytical HPLC chromatogram for [^{11}C]**48** collected from the above HPLC analysis when co–injected with **48.**

Run	Retention time (min)	Yield (decay-corrected) (%)	Mean yield ± SD (%)	Isolated yield (%)
1	7.988	84	85 ± 2	81
2	8.118	85		
3	8.111	87		

HPLC analysis of [^{11}C]49

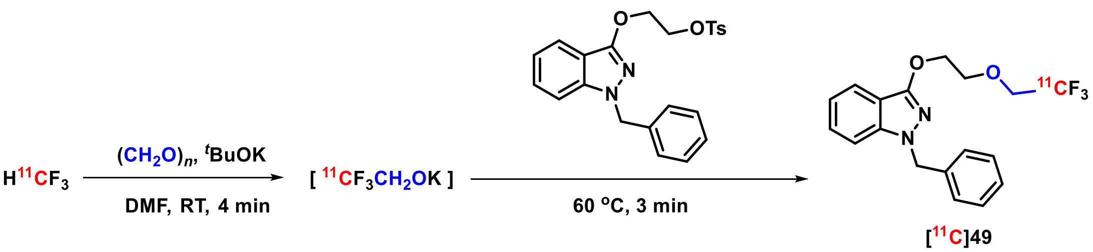

Prepared following general procedure B and analyzed using HPLC condition A.

HPLC condition A

Column: Luna C18(2) (10 μm, 250 × 4.6 mm; i.d. Phenomenex)

Solvent A: H_2O, Solvent B: MeCN

Flow rate: 2 mL/min

0–1 min: 40% B

1–3 min: 40% to 60% B

3–6 min: 60% to 95% B

6–12 min: 95% B

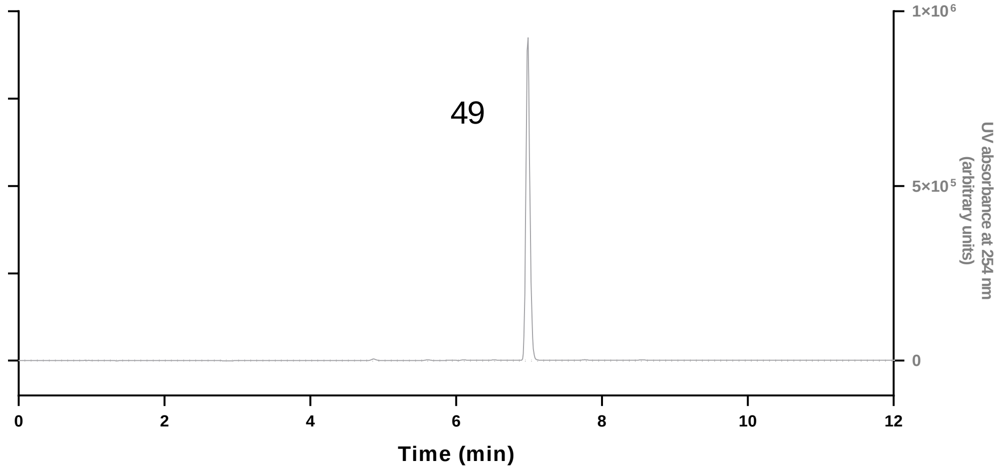

Supplementary Figure 169. Analytical HPLC chromatogram for **49.**

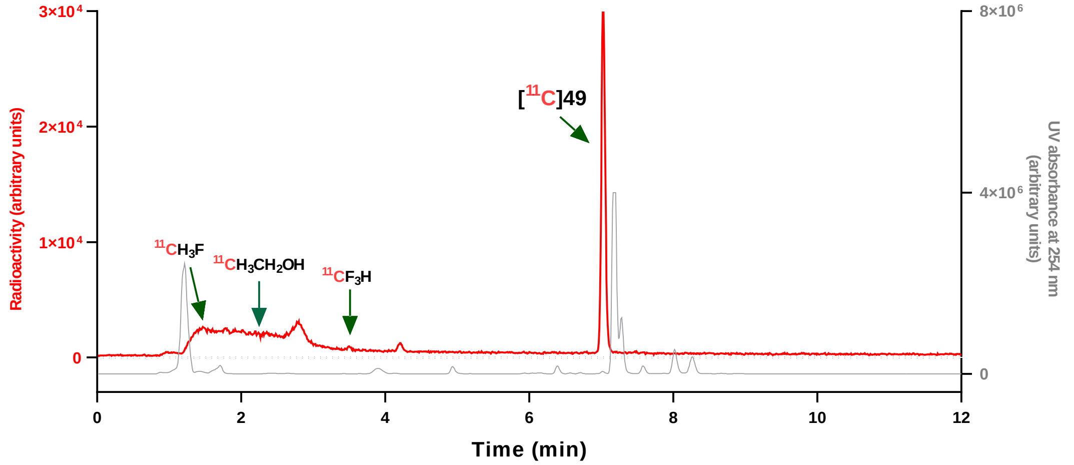

Supplementary Figure 170. Analytical HPLC chromatogram for reaction mixture.

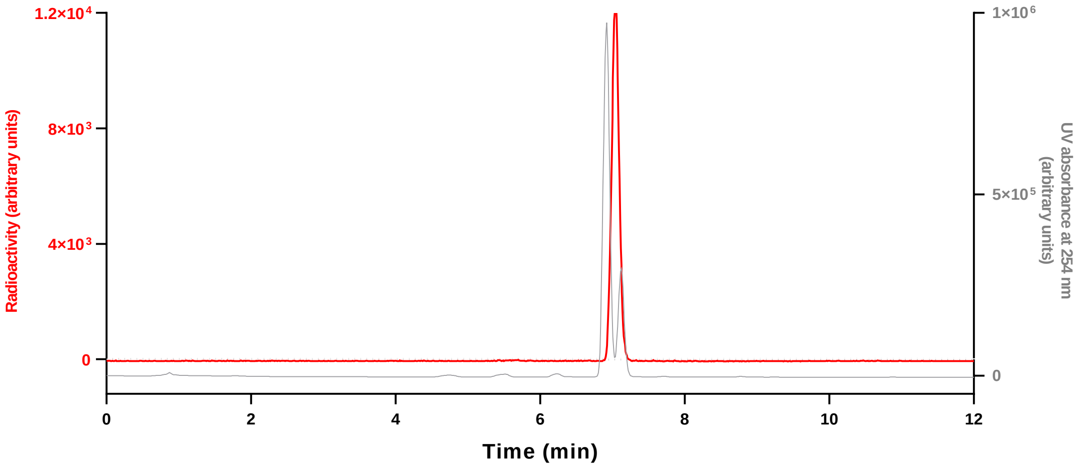

Supplementary Figure 171. Analytical HPLC chromatogram for [^{11}C]**49** collected from the HPLC analysis when co–injected with **49.**

Run	Retention time (min)	Yield (decay-corrected) (%)	Mean yield ± SD (%)	Isolated yield (%)
1	7.025	87	86 ± 1	84
2	7.028	87		
3	7.028	85		

HPLC analysis of [^{11}C]50

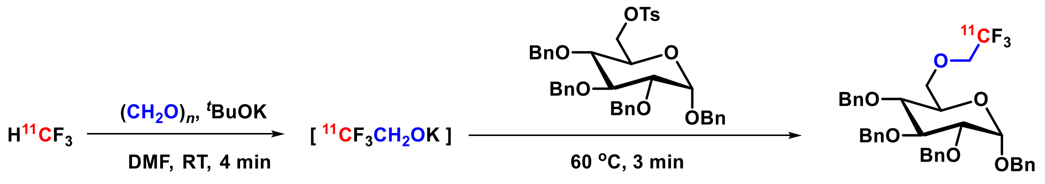

[^{11}C]50

Prepared following general procedure B and analyzed using HPLC condition A.

HPLC condition A

Column: Luna C18(2) (10 μm, 250 × 4.6 mm; i.d. Phenomenex)

Solvent A: H_2O, Solvent B: MeCN

Flow rate: 2 mL/min

0–1 min: 40% B

1–3 min: 40% to 60% B

3–6 min: 60% to 95% B

6–12 min: 95% B

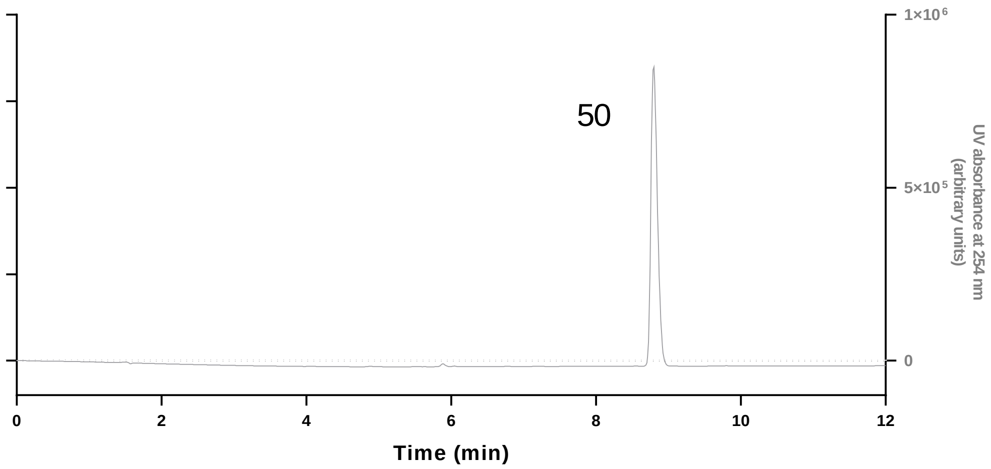

Supplementary Figure 172. Analytical HPLC chromatogram for **50.**

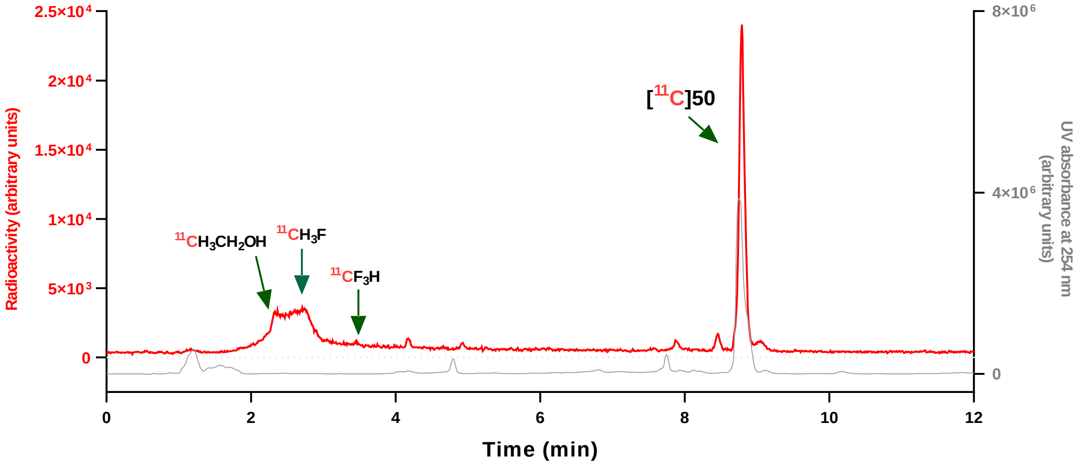

Supplementary Figure 173. Analytical HPLC chromatogram for reaction mixture.

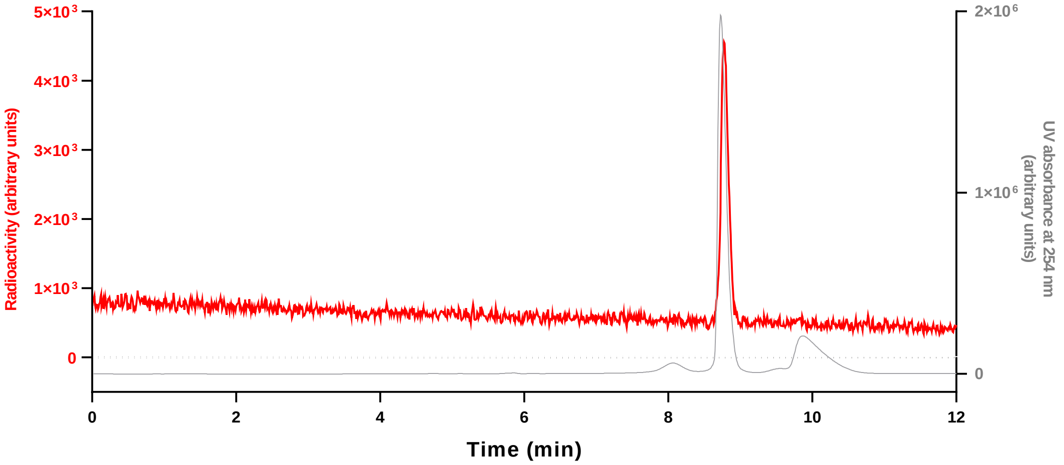

Supplementary Figure 174. Analytical HPLC chromatogram for [^{11}C]**50** collected from the above HPLC analysis when co–injected with **50.**

Run	Retention time (min)	Yield (decay-corrected) (%)	Mean yield ± SD (%)	Isolated yield (%)
1	8.800	82	82 ± 1	77
2	8.787	81		
3	8.790	82		

HPLC analysis of [^{11}C]51

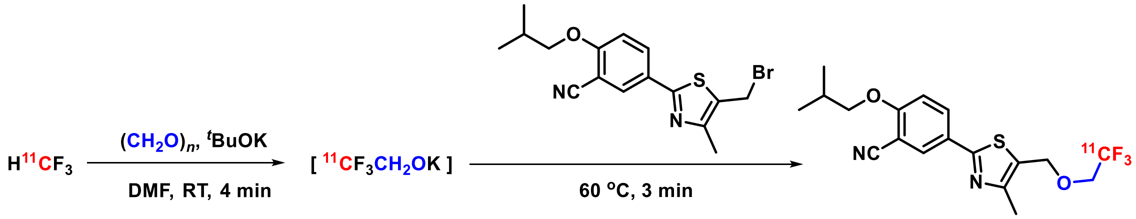

[^{11}C]51

Prepared following general procedure B and analyzed using HPLC condition A.

HPLC condition A

Column: Luna C18(2) (10 μm, 250 × 4.6 mm; i.d. Phenomenex)

Solvent A: H_2O, Solvent B: MeCN

Flow rate: 2 mL/min

0–1 min: 40% B

1–3 min: 40% to 60% B

3–6 min: 60% to 95% B

6–12 min: 95% B

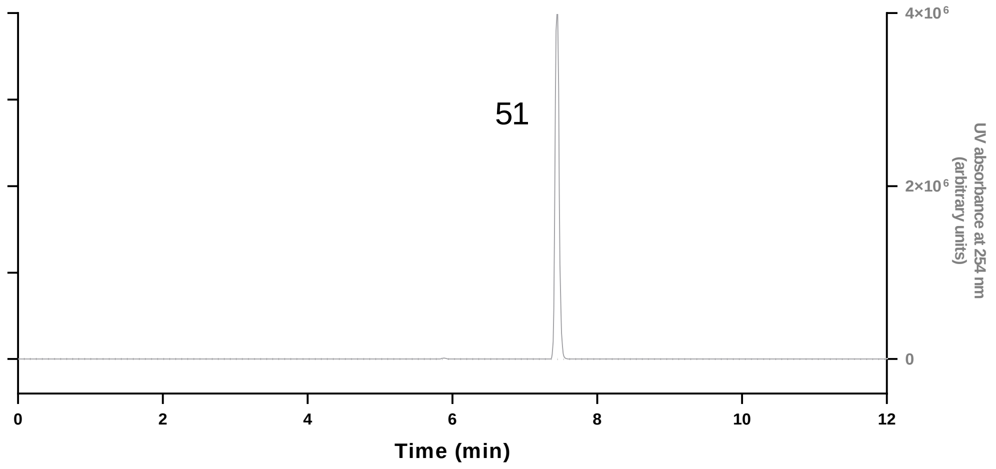

Supplementary Figure 175. Analytical HPLC chromatogram for **51.**

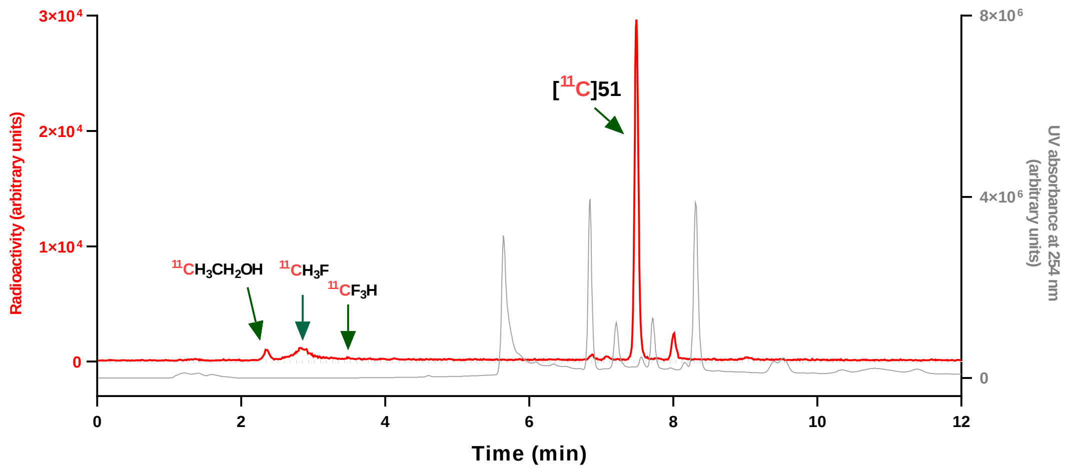

Supplementary Figure 176. Analytical HPLC chromatogram for reaction mixture.

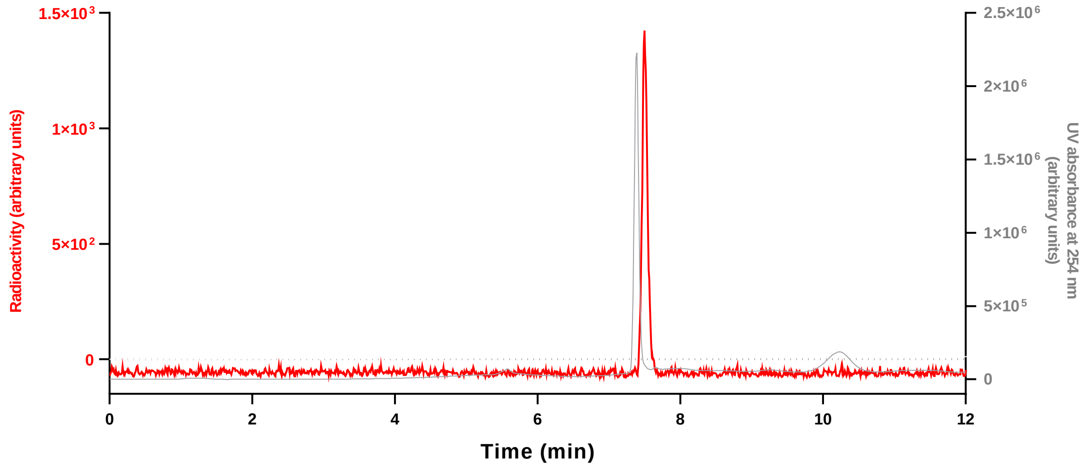

Supplementary Figure 177. Analytical HPLC chromatogram for [^{11}C]**51** collected from the above HPLC analysis when co–injected with **51.**

Run	Retention time (min)	Yield (decay-corrected) (%)	Mean yield ± SD (%)	Isolated yield (%)
1	7.370	86	85 ± 2	88
2	7.482	83		
3	7.489	85		

HPLC analysis of [^{11}C]52

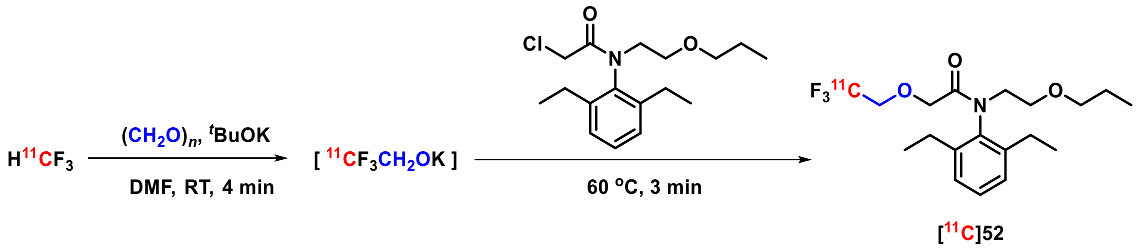

Prepared following general procedure B and analyzed using HPLC condition A.

HPLC condition A

Column: Luna C18(2) (10 μm, 250 × 4.6 mm; i.d. Phenomenex)

Solvent A: H_2O, Solvent B: MeCN

Flow rate: 2 mL/min

0–1 min: 40% B

1–3 min: 40% to 60% B

3–6 min: 60% to 95% B

6–12 min: 95% B

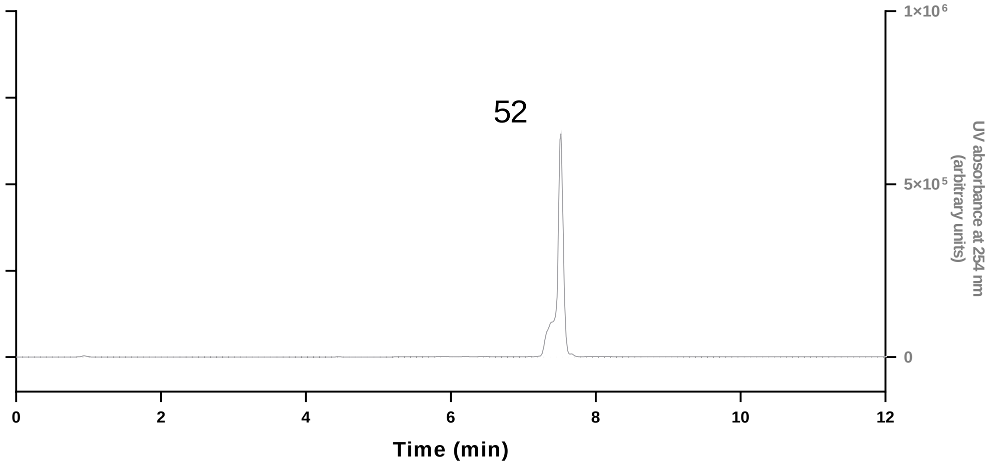

Supplementary Figure 178. Analytical HPLC chromatogram for **52.**

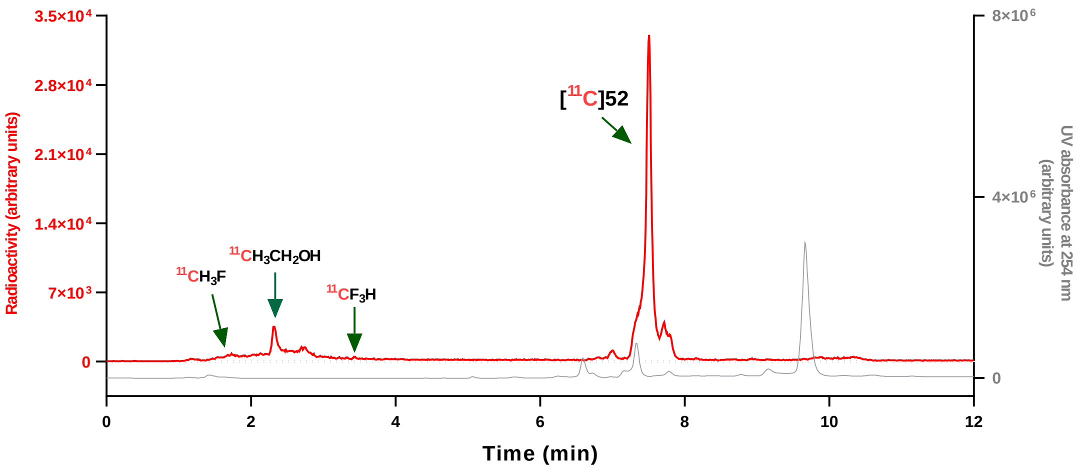

Supplementary Figure 179. Analytical HPLC chromatogram for reaction mixture.

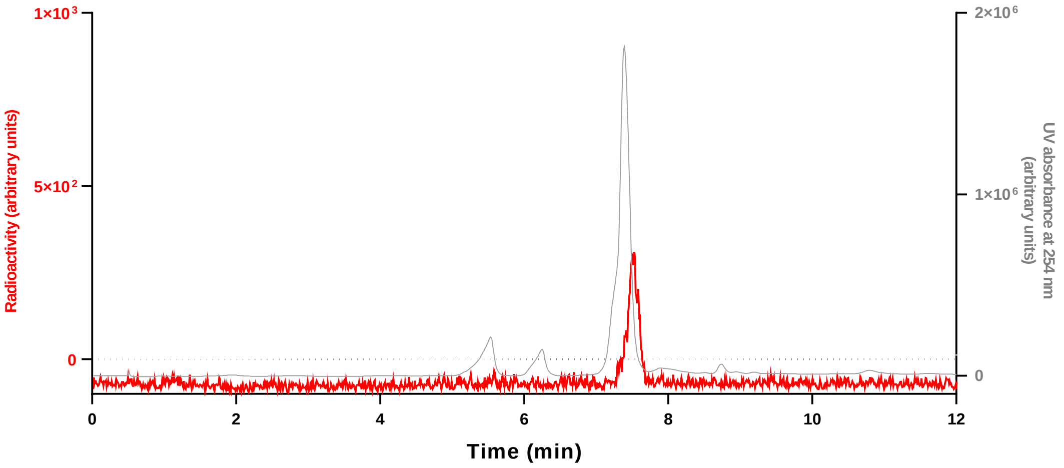

Supplementary Figure 180. Analytical HPLC chromatogram for [^{11}C]**52** collected from the above HPLC analysis when co–injected with **52.**

Run	Retention time (min)	Yield (decay-corrected) (%)	Mean yield ± SD (%)	Isolated yield (%)
1	7.506	70	73 ± 3	58
2	7.608	76		
3	7.585	73		

HPLC analysis of [^{11}C]53

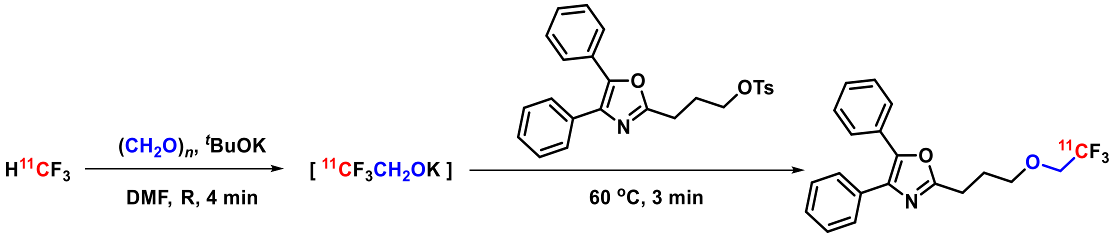

Prepared following general procedure B and analyzed using HPLC condition A.

HPLC condition A

Column: Luna C18(2) (10 μm, 250 × 4.6 mm; i.d. Phenomenex)

Solvent A: H_2O, Solvent B: MeCN

Flow rate: 2 mL/min

0–1 min: 40% B

1–3 min: 40% to 60% B

3–6 min: 60% to 95% B

6–12 min: 95% B

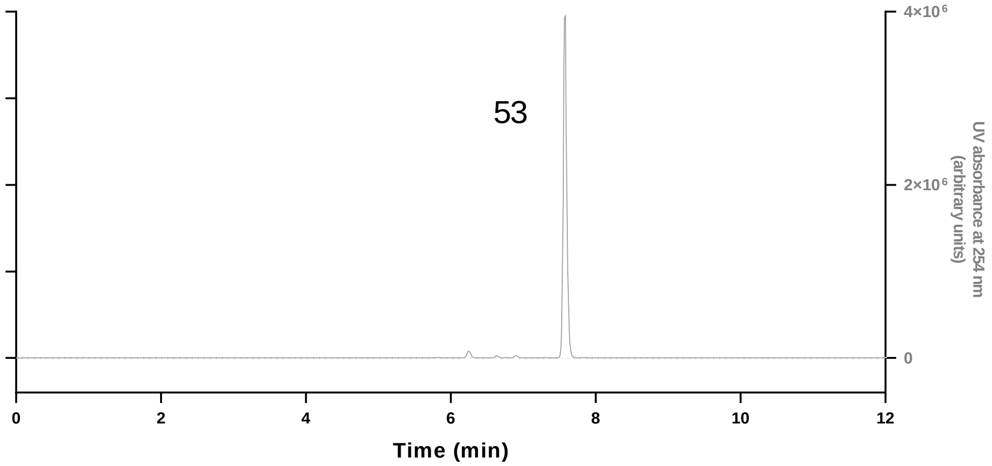

Supplementary Figure 181. Analytical HPLC chromatogram for **53.**

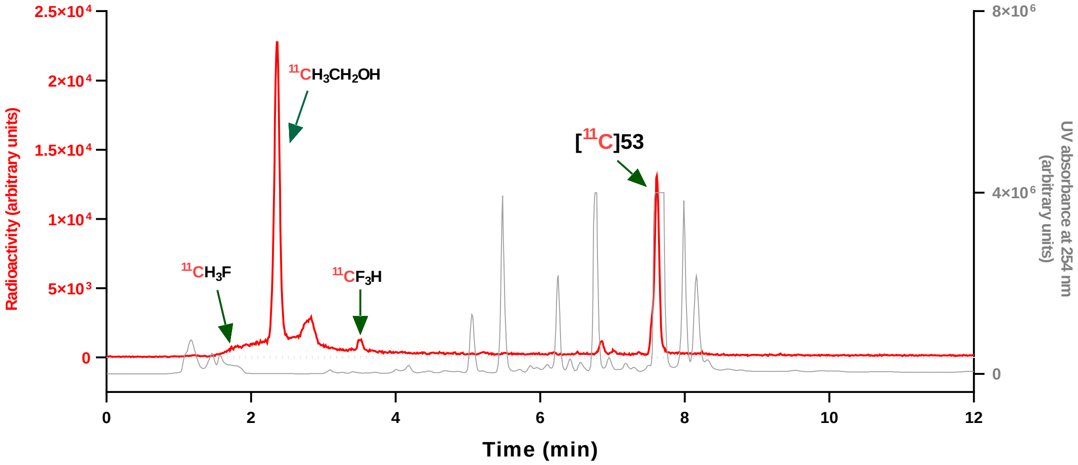

Supplementary Figure 182. Analytical HPLC chromatogram for reaction mixture.

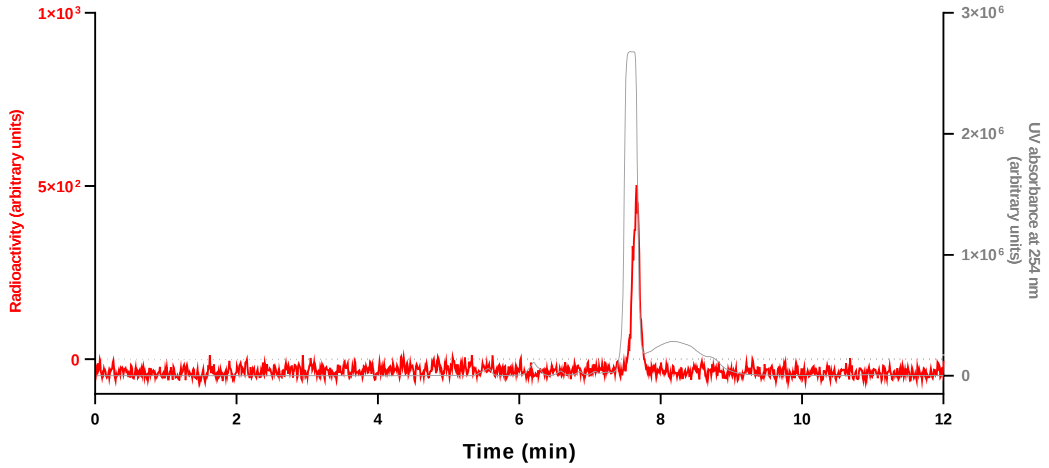

Supplementary Figure 183. Analytical HPLC chromatogram for [^{11}C]**53** collected from the above HPLC analysis when co–injected with **53.**

Run	Retention time (min)	Yield (decay-corrected) (%)	Mean yield ± SD (%)	Isolated yield (%)
1	7.615	35	34 ± 2	23
2	7.621	35		
3	7.615	32		

HPLC analysis of [^{11}C]54

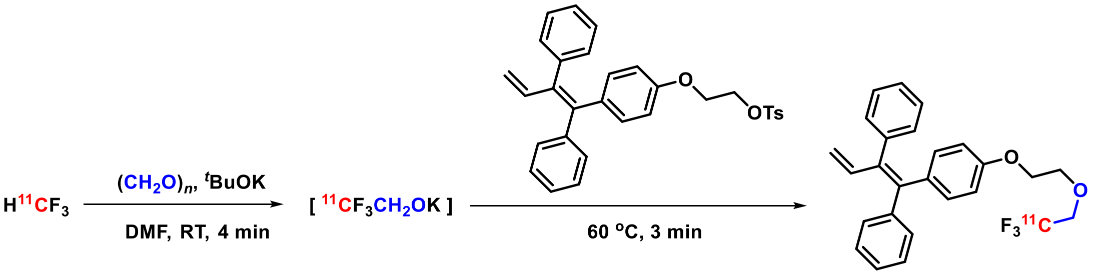

Prepared following general procedure B and analyzed using HPLC condition A.

HPLC condition A

Column: Luna C18(2) (10 μm, 250 × 4.6 mm; i.d. Phenomenex)

Solvent A: H_2O, Solvent B: MeCN

Flow rate: 2 mL/min

0–1 min: 40% B

1–3 min: 40% to 60% B

3–6 min: 60% to 95% B

6–12 min: 95% B

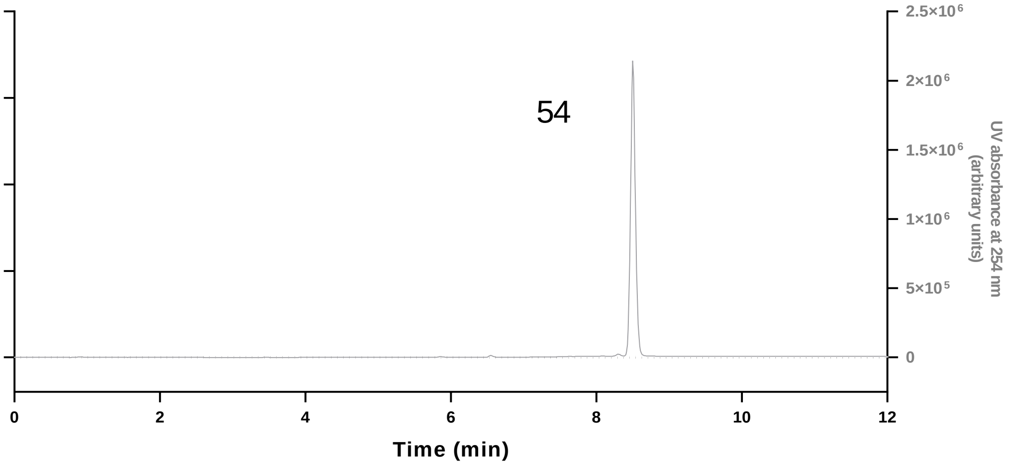

Supplementary Figure 184. Analytical HPLC chromatogram for **54.**

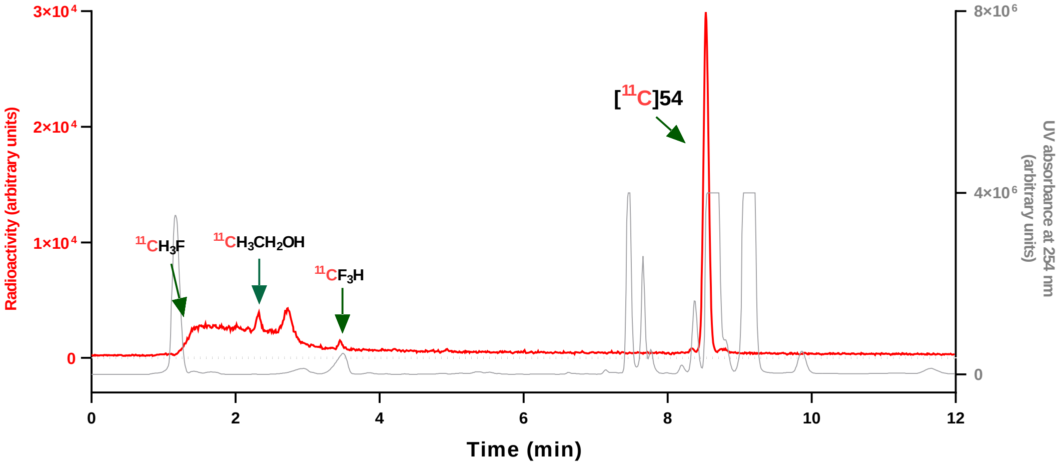

Supplementary Figure 185. Analytical HPLC chromatogram for reaction mixture.

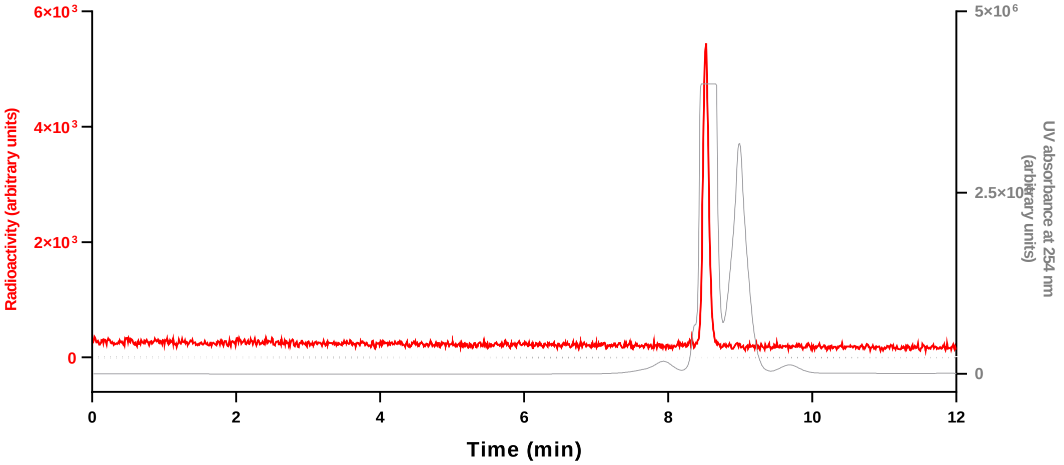

Supplementary Figure 186. Analytical HPLC chromatogram for [^{11}C]**54** collected from the above HPLC analysis when co–injected with **54.**

Run	Retention time (min)	Yield (decay-corrected) (%)	Mean yield ± SD (%)	Isolated yield (%)
1	8.533	91	93 ± 2	81
2	8.537	93		
3	8.544	95		

HPLC analysis of [^{11}C]55

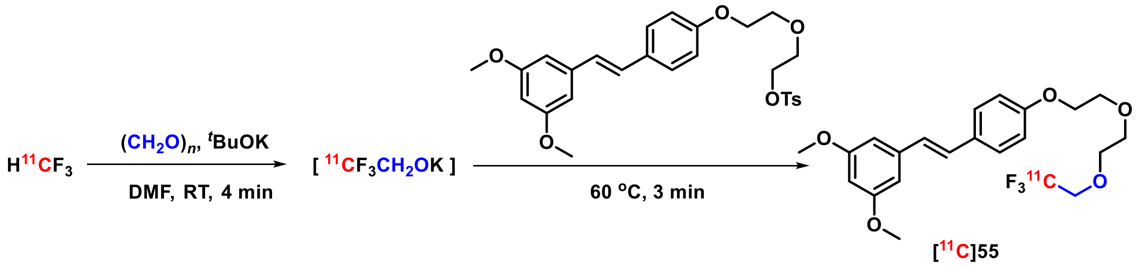

Prepared following general procedure B and analyzed using HPLC condition A.

HPLC condition A

Column: Luna C18(2) (10 μm, 250 × 4.6 mm; i.d. Phenomenex)

Solvent A: H_2O, Solvent B: MeCN

Flow rate: 2 mL/min

0–1 min: 40% B

1–3 min: 40% to 60% B

3–6 min: 60% to 95% B

6–12 min: 95% B

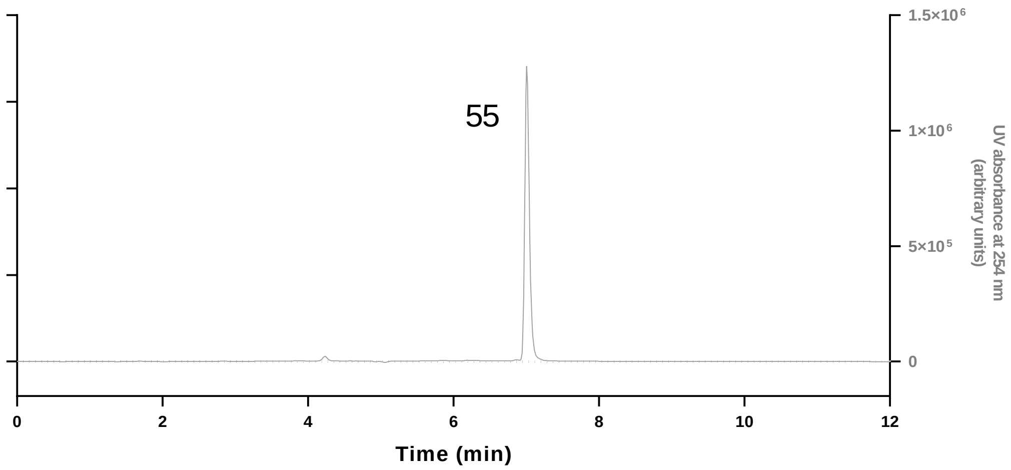

Supplementary Figure 187. Analytical HPLC chromatogram for **55.**

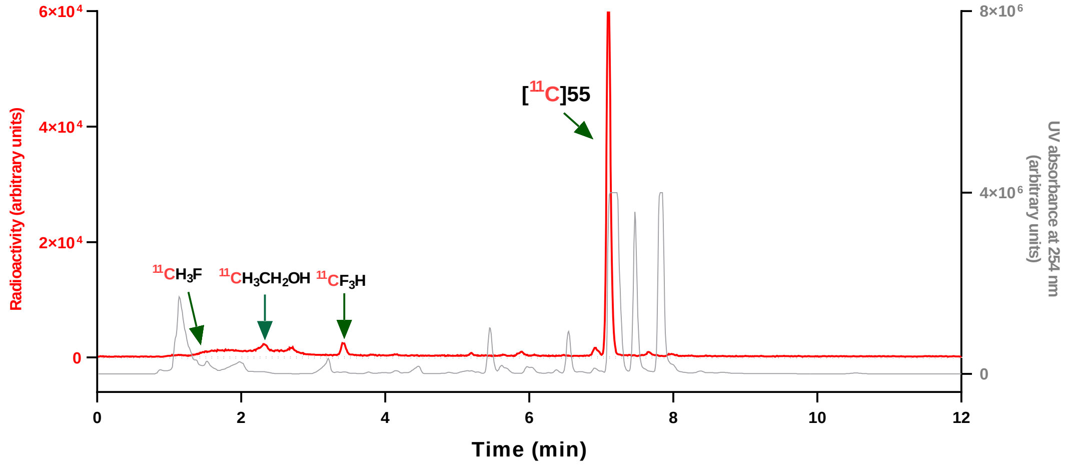

Supplementary Figure 188. Analytical HPLC chromatogram for reaction mixture.

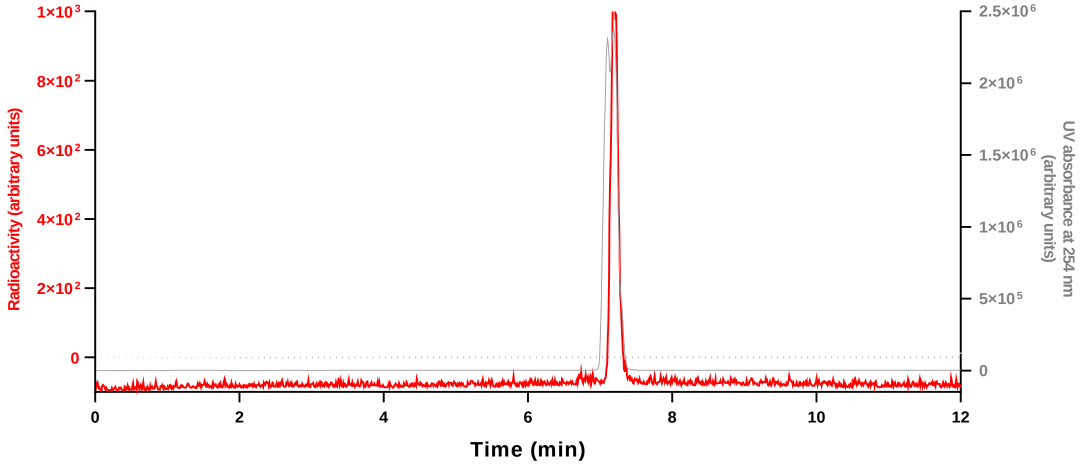

Supplementary Figure 189. Analytical HPLC chromatogram for [^{11}C]**55** collected from the above HPLC analysis when co–injected with **55.**

Run	Retention time (min)	Yield (decay-corrected) (%)	Mean yield ± SD (%)	Isolated yield (%)
1	7.102	84	87 ± 3	83
2	7.041	89		
3	7.147	89		

HPLC analysis of [^{11}C]56

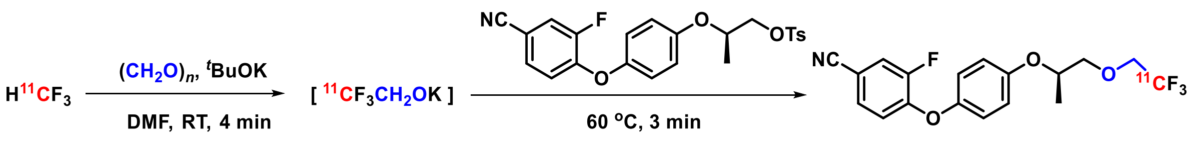

[^{11}C]56

Prepared following general procedure B and analyzed using HPLC condition A.

HPLC condition A

Column: Luna C18(2) (10 μm, 250 × 4.6 mm; i.d. Phenomenex)

Solvent A: H_2O, Solvent B: MeCN

Flow rate: 2 mL/min

0–1 min: 40% B

1–3 min: 40% to 60% B

3–6 min: 60% to 95% B

6–12 min: 95% B

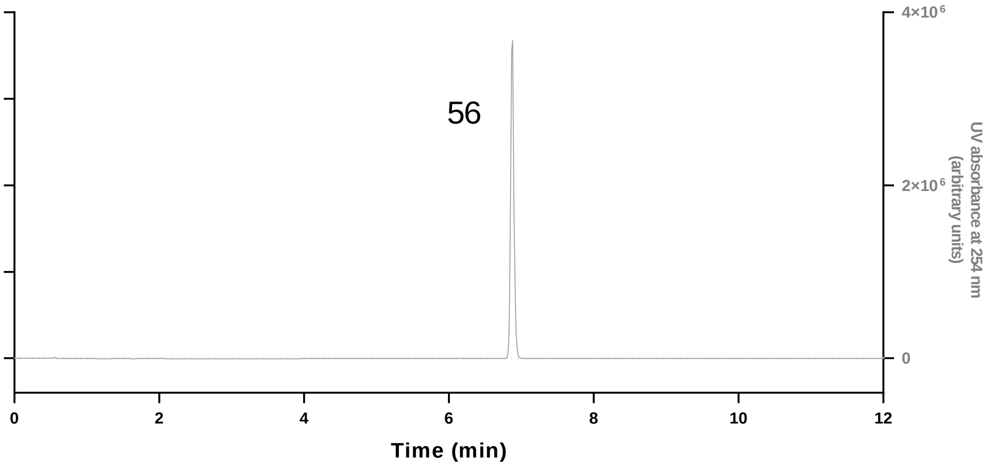

Supplementary Figure 190. Analytical HPLC chromatogram for **56.**

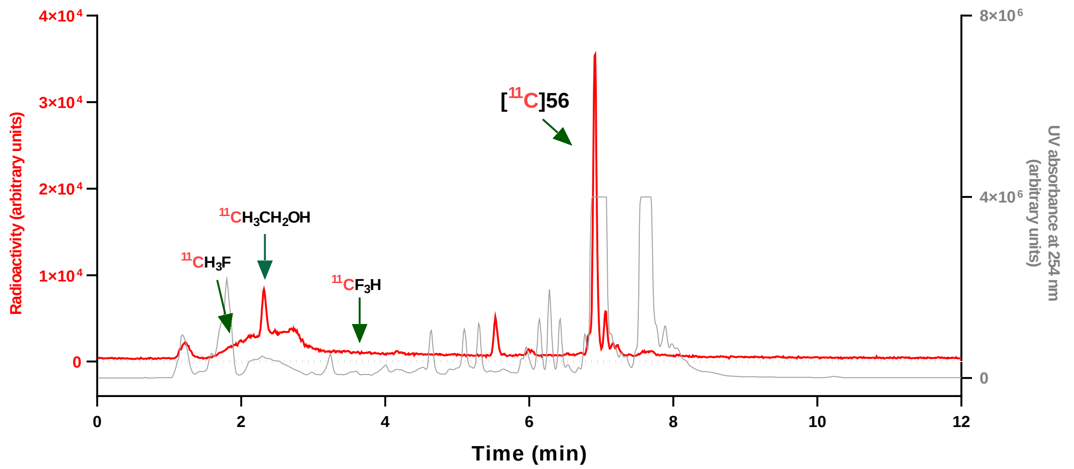

Supplementary Figure 191. Analytical HPLC chromatogram for reaction mixture.

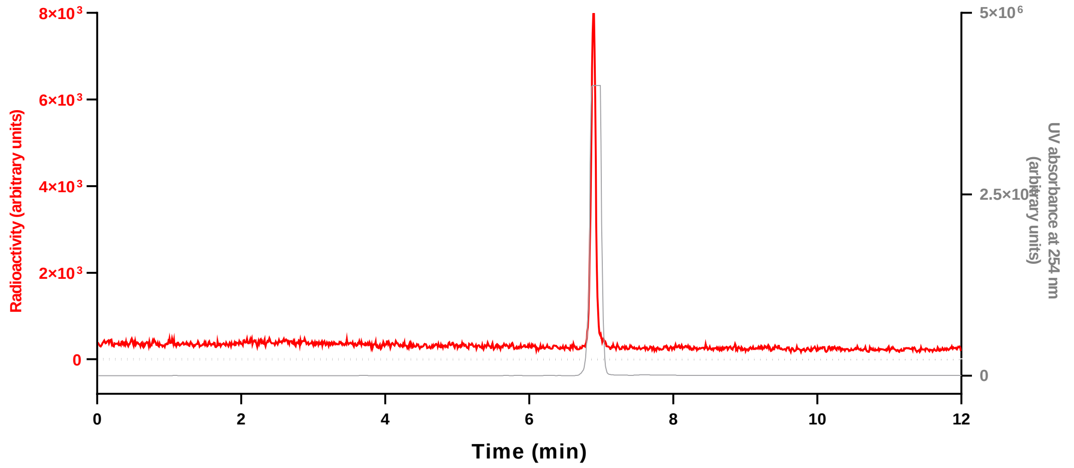

Supplementary Figure 192. Analytical HPLC chromatogram for [^{11}C]**56** collected from the above HPLC analysis when co–injected with **56.**

Run	Retention time (min)	Yield (decay-corrected) (%)	Mean yield ± SD (%)	Isolated yield (%)
1	6.913	50	51 ± 1	52
2	6.913	51		
3	6.914	52		

7.2. HPLC analysis of ^{18}F-labeled compounds.

HPLC analysis of [^{18}F]1

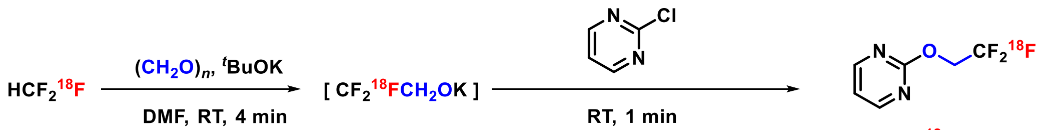

[^{18}F]1

Prepared following general procedure B and analyzed using HPLC condition C.

HPLC condition C

Column: Luna C18(2) (10 μm, 250 × 4.6 mm; i.d. Phenomenex)

Solvent A: H_2O, Solvent B: MeCN

Flow rate = 2 mL/min

0–1 min: 30% B

1–15 min: 30% to 80% B

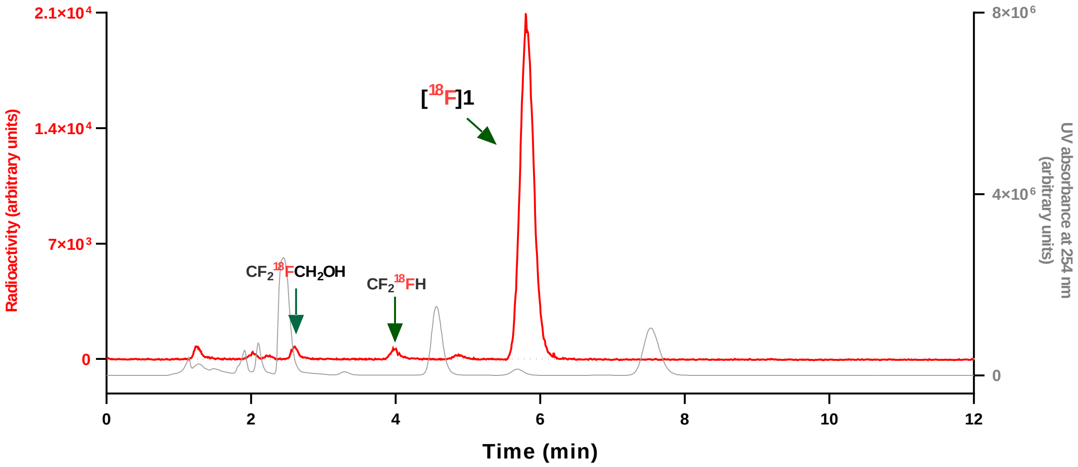

Supplementary Figure 193. Analytical HPLC chromatogram for reaction mixture.

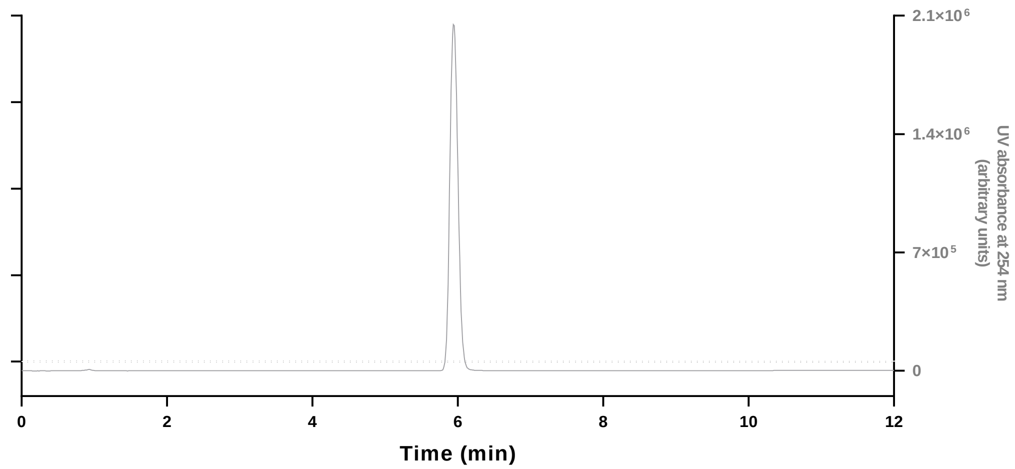

Supplementary Figure 194. Analytical HPLC chromatogram for **1.**

Run	Retention time (min)	Yield (decay-corrected) (%)	Mean yield ± SD (%)	Isolated yield (%)
1	5.818	82	88 ± 5	79
2	5.824	91		
3	5.804	91		

HPLC analysis of [^{18}F]4

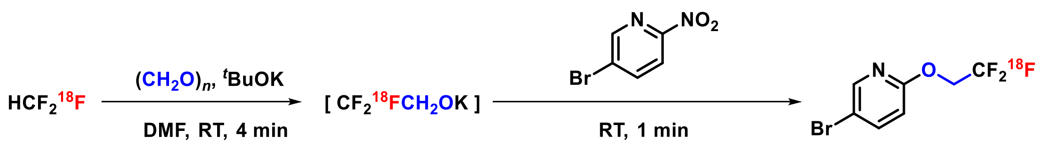

[^{18}F]4

Prepared following general procedure B and analyzed using HPLC condition A.

HPLC condition A

Column: Luna C18(2) (10 μm, 250 × 4.6 mm; i.d. Phenomenex)

Solvent A: H_2O, Solvent B: MeCN

Flow rate: 2 mL/min

0–1 min: 40% B

1–3 min: 40% to 60% B

3–6 min: 60% to 95% B

6–12 min: 95% B

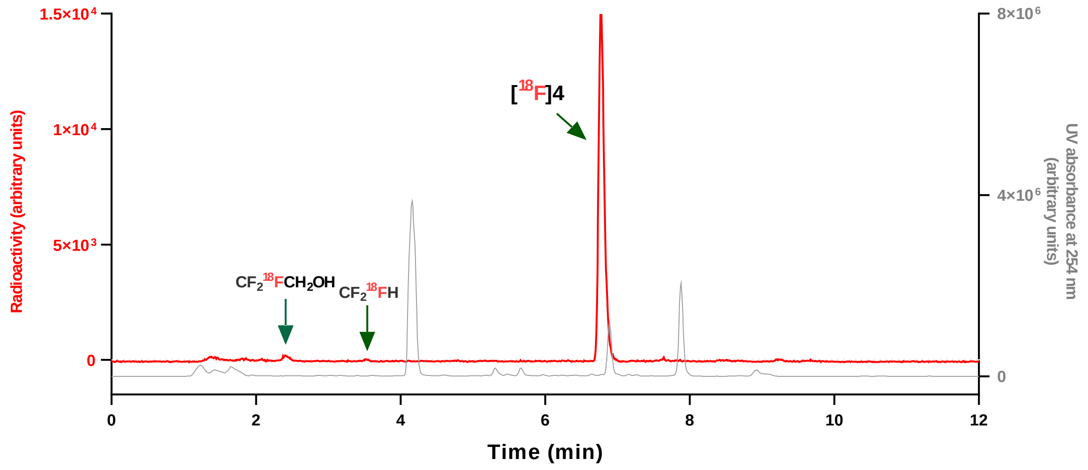

Supplementary Figure 195. Analytical HPLC chromatogram for reaction mixture.

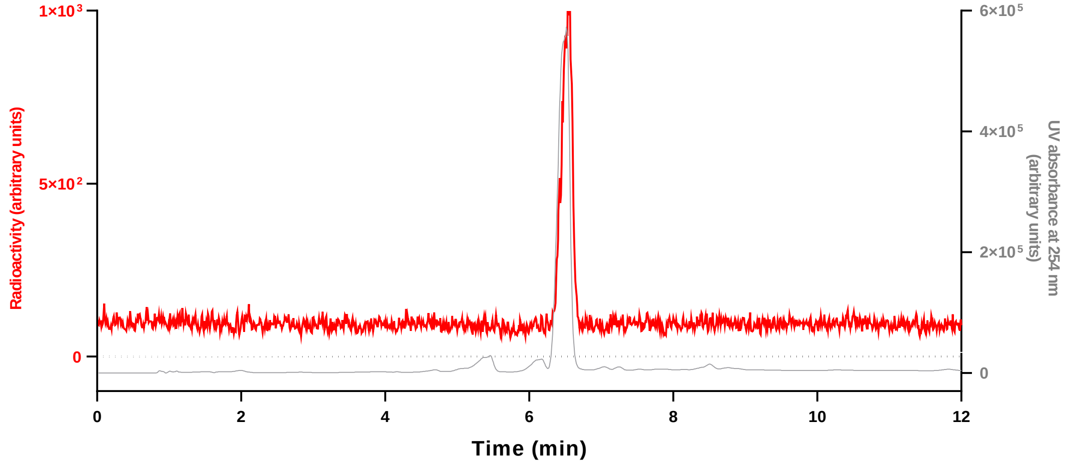

Supplementary Figure 196. Analytical HPLC chromatogram for [^{18}F]**4** collected from the above HPLC analysis when co–injected with **4.**

Run	Retention time (min)	Yield (decay-corrected) (%)	Mean yield ± SD (%)	Isolated yield (%)
1	6.767	94	96 ± 2	83
2	6.775	98		
3	6.773	95		

HPLC analysis of [^{18}F]6

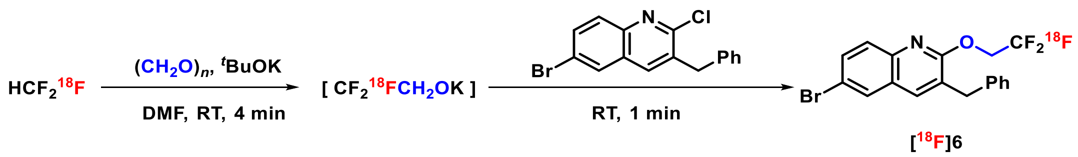

Prepared following general procedure B and analyzed using HPLC condition A.

HPLC condition A

Column: Luna C18(2) (10 μm, 250 × 4.6 mm; i.d. Phenomenex)

Solvent A: H_2O, Solvent B: MeCN

Flow rate: 2 mL/min

0–1 min: 40% B

1–3 min: 40% to 60% B

3–6 min: 60% to 95% B

6–12 min: 95% B

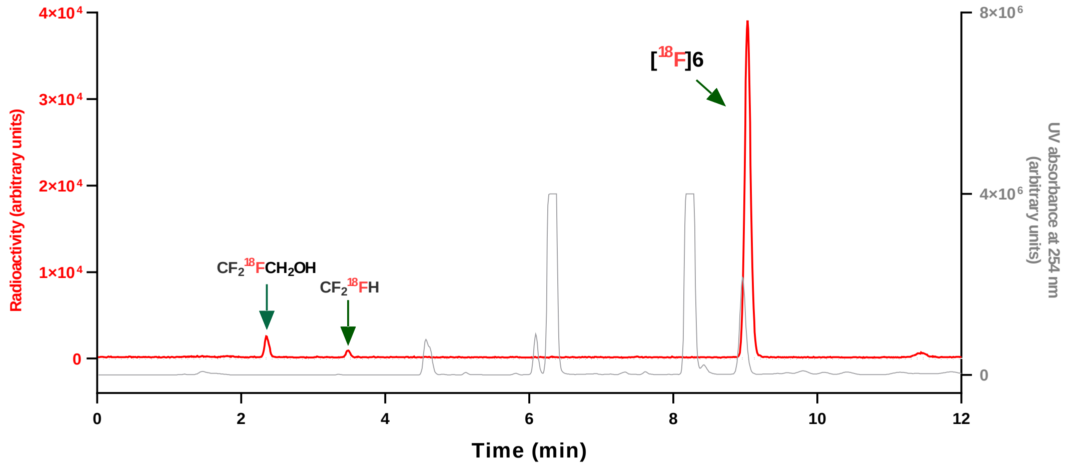

Supplementary Figure 197. Analytical HPLC chromatogram for reaction mixture.

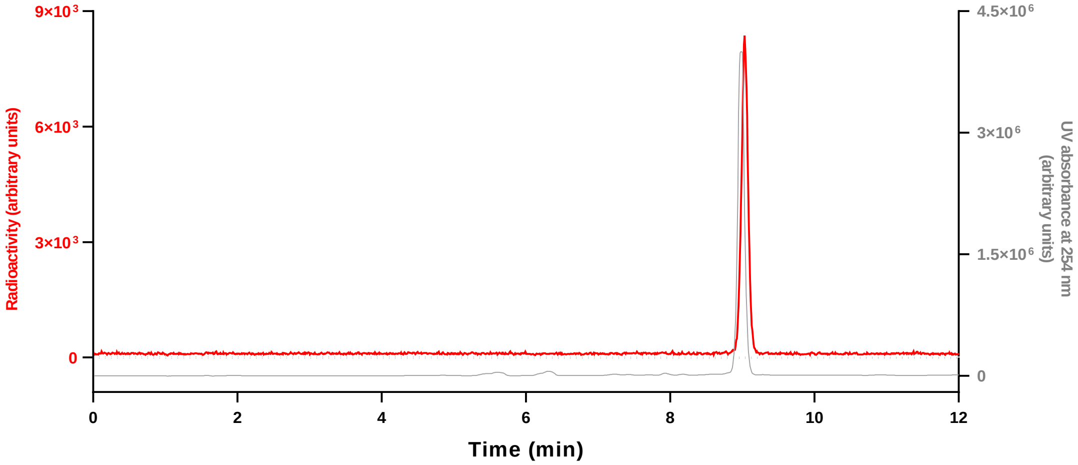

Supplementary Figure 198. Analytical HPLC chromatogram for [^{18}F]**6** collected from the above HPLC analysis when co–injected with **6.**

Run	Retention time (min)	Yield (decay-corrected) (%)	Mean yield ± SD (%)	Isolated yield (%)
1	8.912	88	91 ± 2	83
2	9.033	92		
3	9.047	92		

HPLC analysis of [^{18}F]8

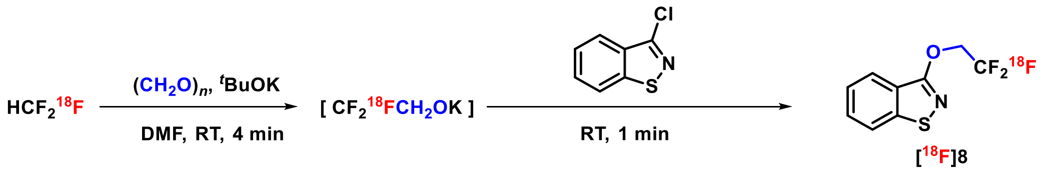

Prepared following general procedure B and analyzed using HPLC condition A.

HPLC condition A

Column: Luna C18(2) (10 μm, 250 × 4.6 mm; i.d. Phenomenex)

Solvent A: H_2O, Solvent B: MeCN

Flow rate: 2 mL/min

0–1 min: 40% B

1–3 min: 40% to 60% B

3–6 min: 60% to 95% B

6–12 min: 95% B

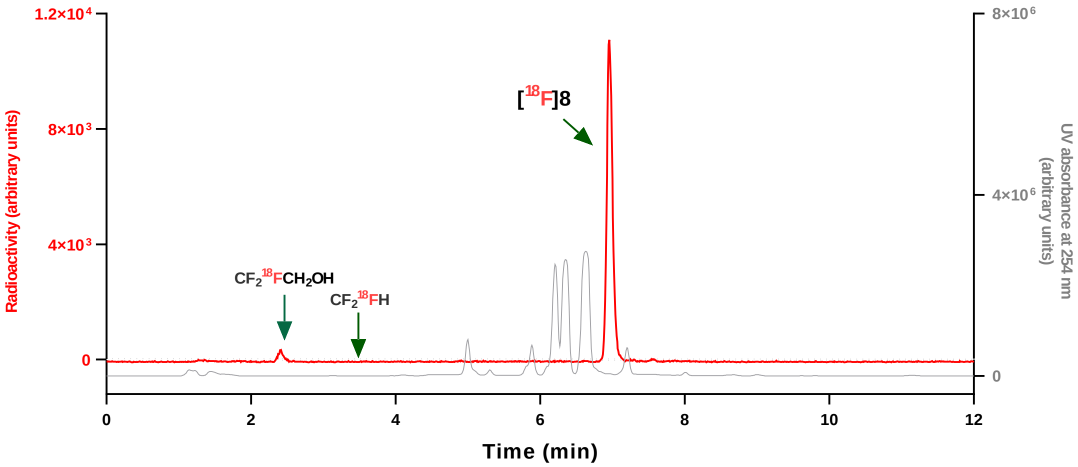

Supplementary Figure 199. Analytical HPLC chromatogram for reaction mixture.

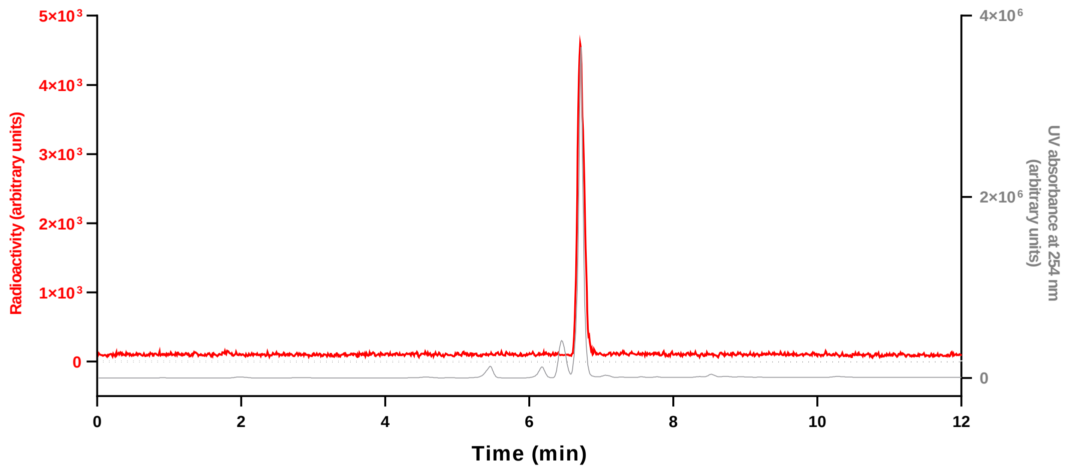

Supplementary Figure 200. Analytical HPLC chromatogram for [^{18}F]**8** collected from the above HPLC analysis when co–injected with **8.**

Run	Retention time (min)	Yield (decay-corrected) (%)	Mean yield ± SD (%)	Isolated yield (%)
1	6.964	92	94 ± 2	85
2	6.956	96		
3	6.953	94		

HPLC analysis of [^{18}F]13

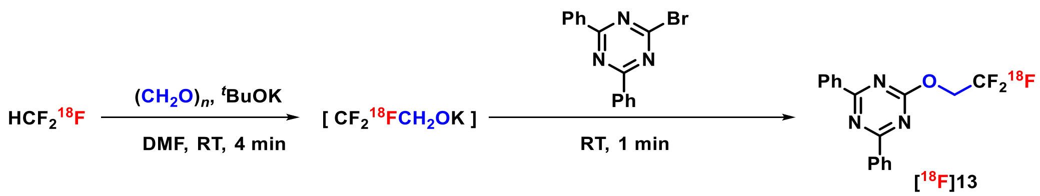

Prepared following general procedure B and analyzed using HPLC condition A.

HPLC condition A

Column: Luna C18(2) (10 μm, 250 × 4.6 mm; i.d. Phenomenex)

Solvent A: H_2O, Solvent B: MeCN

Flow rate: 2 mL/min

0–1 min: 40% B

1–3 min: 40% to 60% B

3–6 min: 60% to 95% B

6–12 min: 95% B

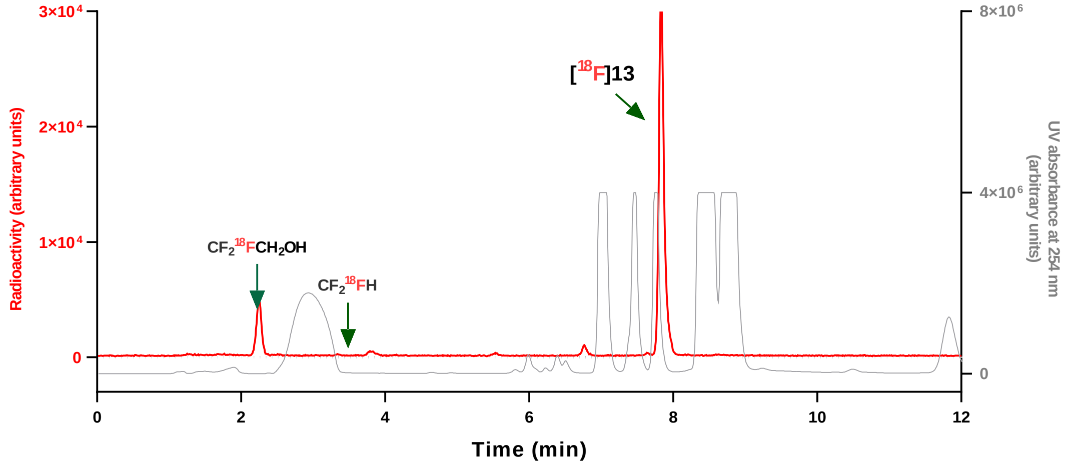

Supplementary Figure 201. Analytical HPLC chromatogram for reaction mixture.

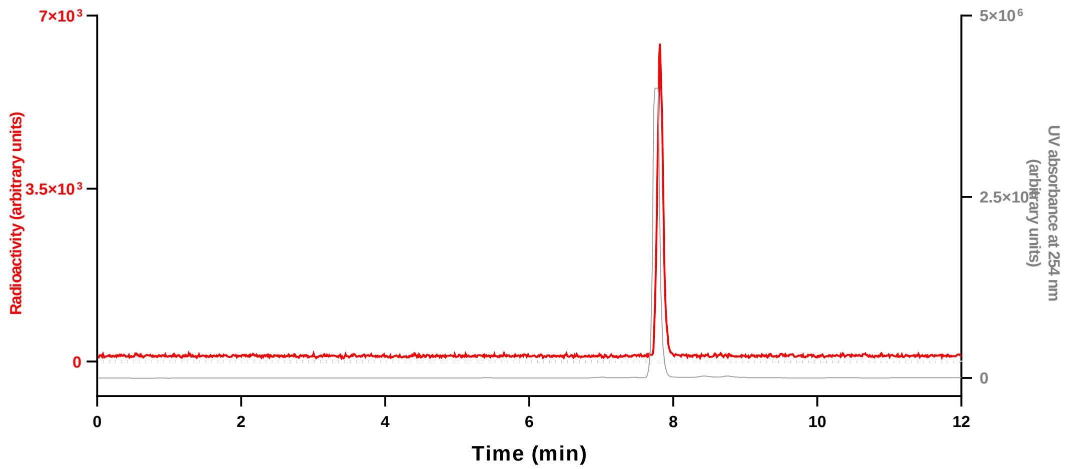

Supplementary Figure 202. Analytical HPLC chromatogram for [^{18}F]**13** collected from the above HPLC analysis when co–injected with **13.**

Run	Retention time (min)	Yield (decay-corrected) (%)	Mean yield ± SD (%)	Isolated yield (%)
1	7.826	83	82 ± 2	77
2	7.833	83		
3	7.836	80		

HPLC analysis of [^{18}F]15

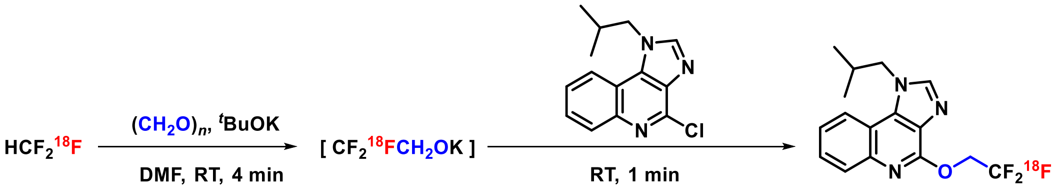

[^{18}F]15

Prepared following general procedure B and analyzed using HPLC condition A.

HPLC condition A

Column: Luna C18(2) (10 μm, 250 × 4.6 mm; i.d. Phenomenex)

Solvent A: H_2O, Solvent B: MeCN

Flow rate: 2 mL/min

0–1 min: 40% B

1–3 min: 40% to 60% B

3–6 min: 60% to 95% B

6–12 min: 95% B

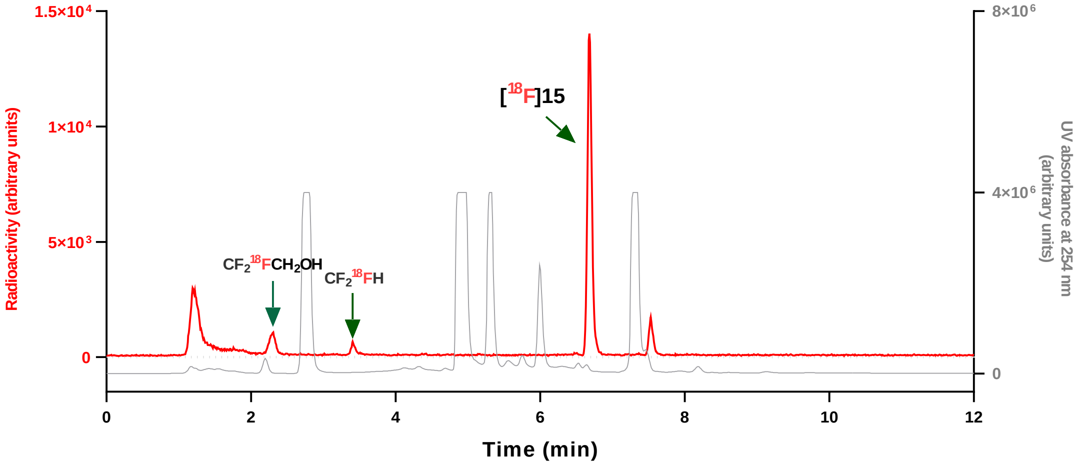

Supplementary Figure 203. Analytical HPLC chromatogram for reaction mixture.

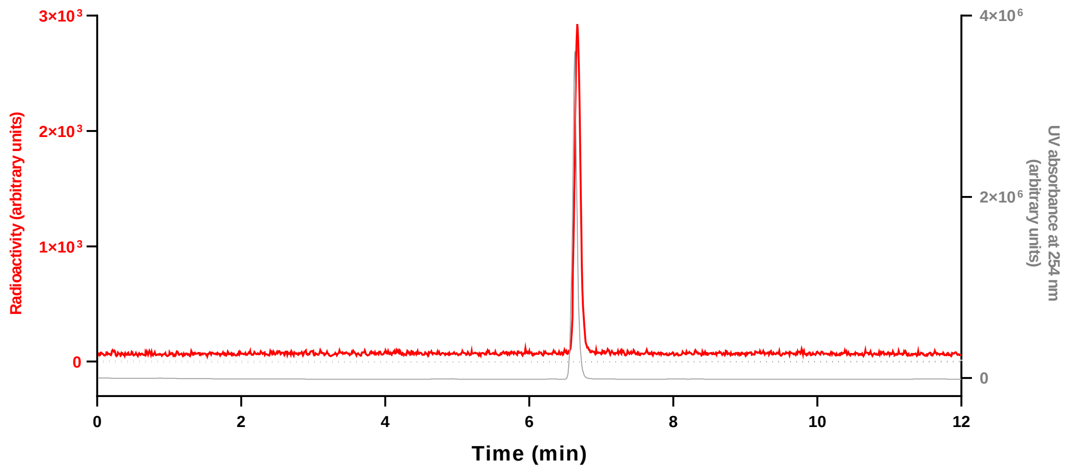

Supplementary Figure 204. Analytical HPLC chromatogram for [^{18}F]**15** collected from the above HPLC analysis when co–injected with **15.**

Run	Retention time (min)	Yield (decay-corrected) (%)	Mean yield ± SD (%)	Isolated yield (%)
1	6.683	54	56 ± 4	57
2	6.662	54		
3	6.652	60		

HPLC analysis of [^{18}F]17

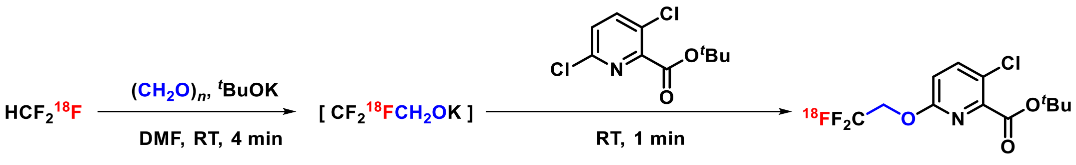

Prepared following general procedure B and analyzed using HPLC condition A.

HPLC condition A

Column: Luna C18(2) (10 μm, 250 × 4.6 mm; i.d. Phenomenex)

Solvent A: H_2O, Solvent B: MeCN

Flow rate: 2 mL/min

0–1 min: 40% B

1–3 min: 40% to 60% B

3–6 min: 60% to 95% B

6–12 min: 95% B

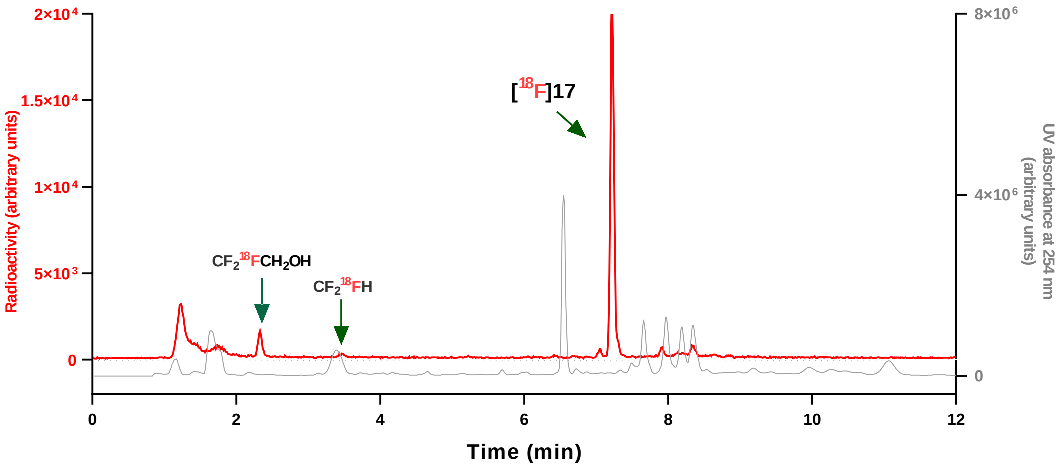

Supplementary Figure 205. Analytical HPLC chromatogram for reaction mixture.

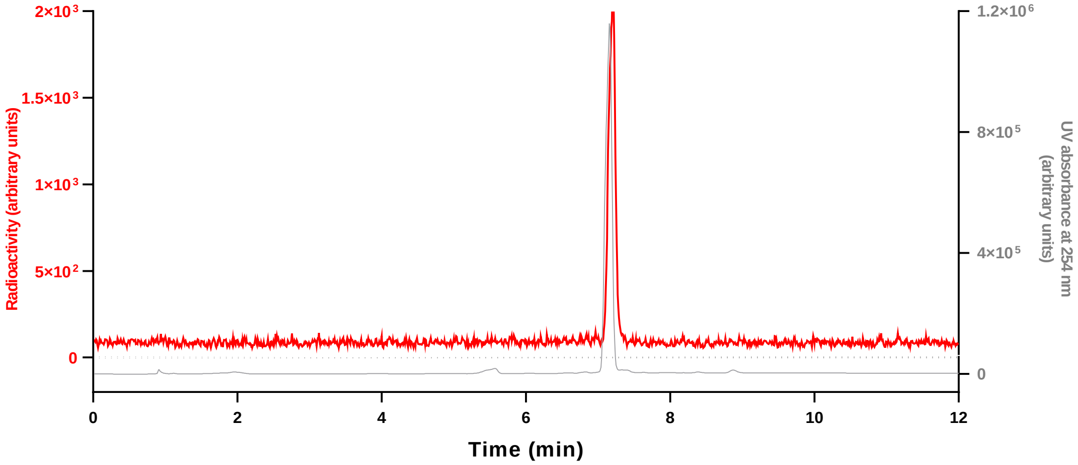

Supplementary Figure 206. Analytical HPLC chromatogram for [^{18}F]**17** collected from the above HPLC analysis when co–injected with **17.**

Run	Retention time (min)	Yield (decay-corrected) (%)	Mean yield ± SD (%)	Isolated yield (%)
1	7.214	58	56 ± 2	44
2	7.229	54		
3	7.220	57		

HPLC analysis of [^{18}F]18

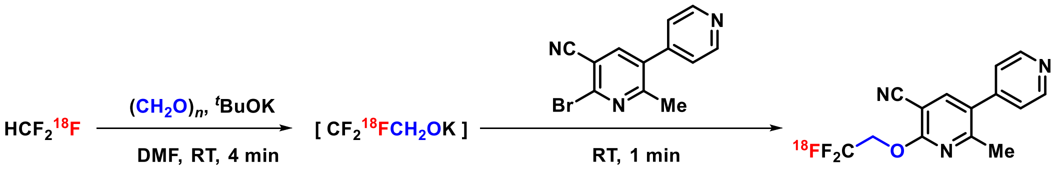

[^{18}F]18

Prepared following general procedure B and analyzed using HPLC condition A.

HPLC condition A

Column: Luna C18(2) (10 μm, 250 × 4.6 mm; i.d. Phenomenex)

Solvent A: H_2O, Solvent B: MeCN

Flow rate: 2 mL/min

0–1 min: 40% B

1–3 min: 40% to 60% B

3–6 min: 60% to 95% B

6–12 min: 95% B

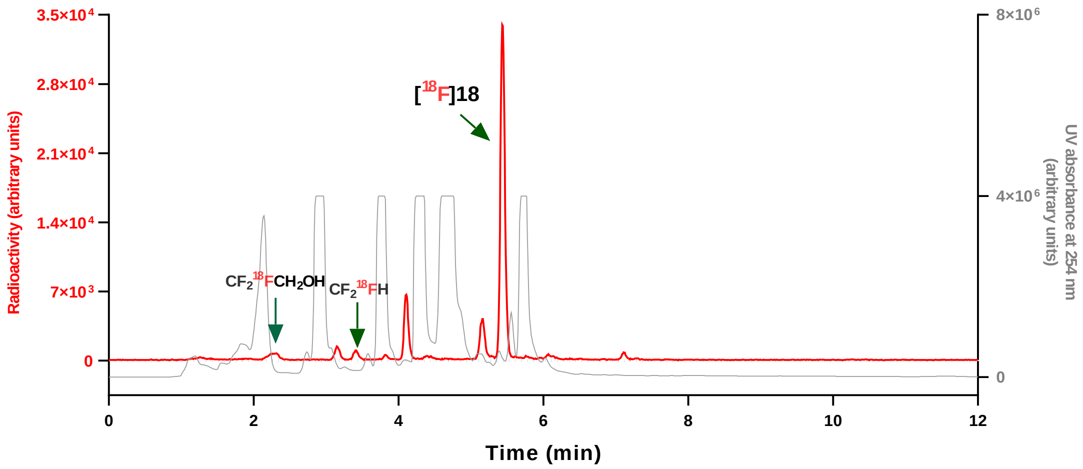

Supplementary Figure 207. Analytical HPLC chromatogram for reaction mixture.

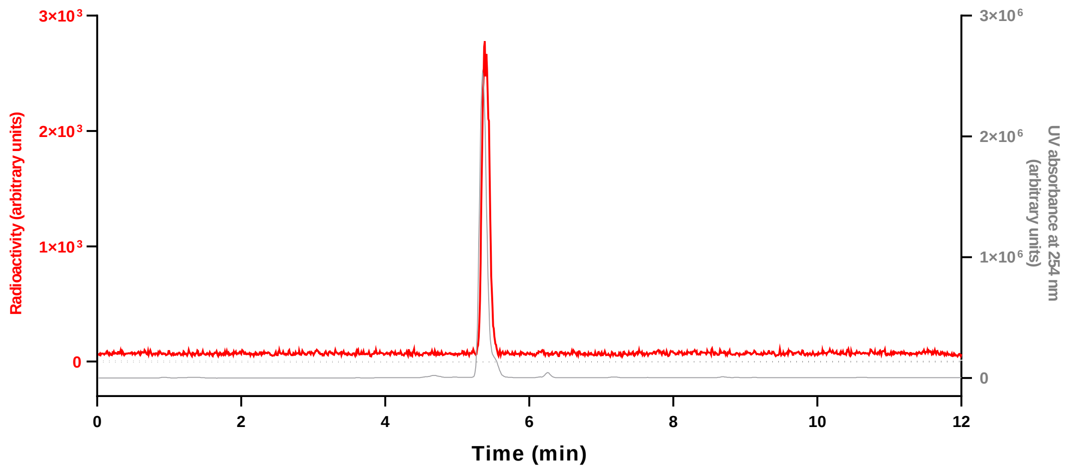

Supplementary Figure 208. Analytical HPLC chromatogram for [^{18}F]**18** collected from the above HPLC analysis when co–injected with **18.**

Run	Retention time (min)	Yield (decay-corrected) (%)	Mean yield ± SD (%)	Isolated yield (%)
1	5.437	63	64 ± 1	56
2	5.436	64		
3	5.413	64		

HPLC analysis of [^{18}F]19

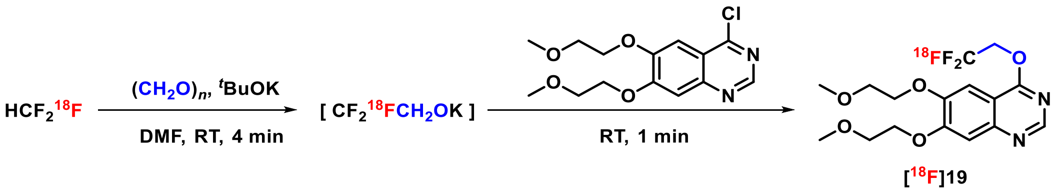

Prepared following general procedure B and analyzed using HPLC condition A.

HPLC condition A

Column: Luna C18(2) (10 μm, 250 × 4.6 mm; i.d. Phenomenex)

Solvent A: H_2O, Solvent B: MeCN

Flow rate: 2 mL/min

0–1 min: 40% B

1–3 min: 40% to 60% B

3–6 min: 60% to 95% B

6–12 min: 95% B

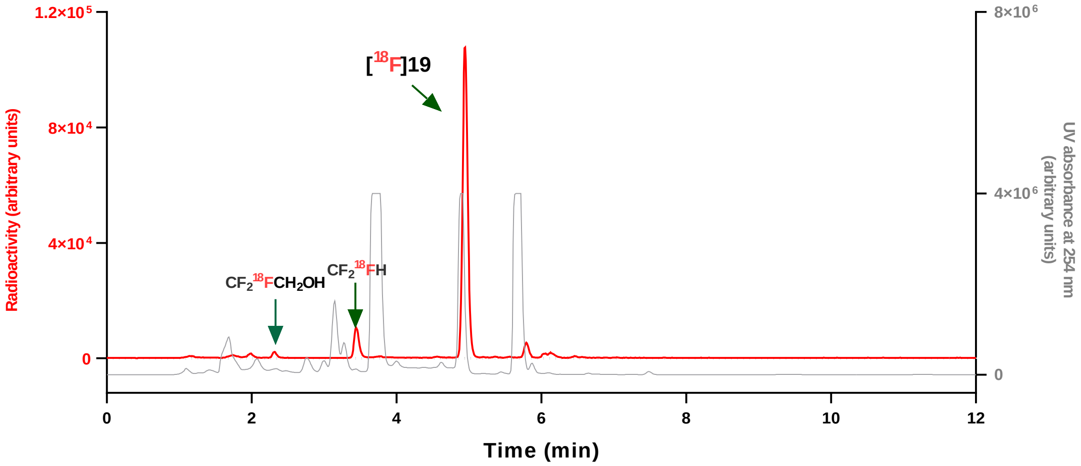

Supplementary Figure 209. Analytical HPLC chromatogram for reaction mixture.

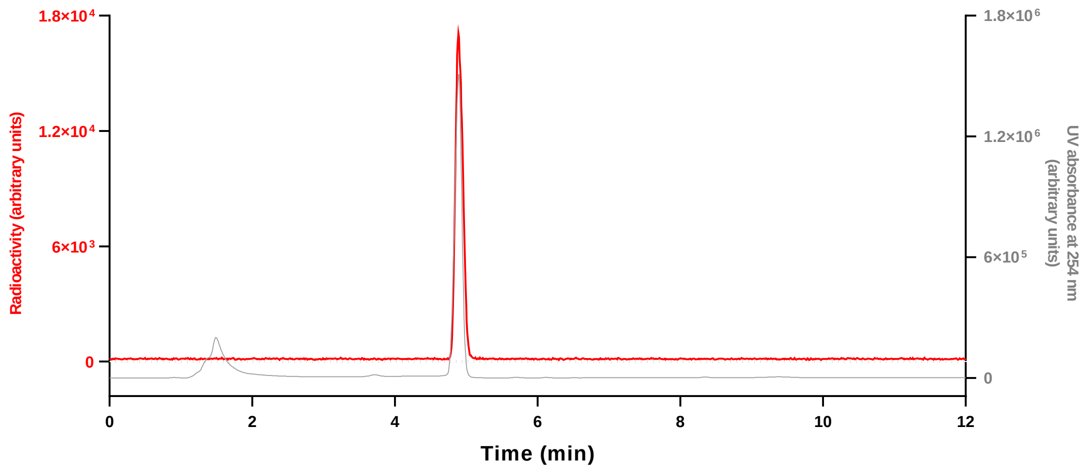

Supplementary Figure 210. Analytical HPLC chromatogram for [^{18}F]**19** collected from the above HPLC analysis when co–injected with **19.**

Run	Retention time (min)	Yield (decay-corrected) (%)	Mean yield ± SD (%)	Isolated yield (%)
1	4.946	72	77 ± 5	80
2	4.948	79		
3	4.946	81		

HPLC analysis of [^{18}F]20

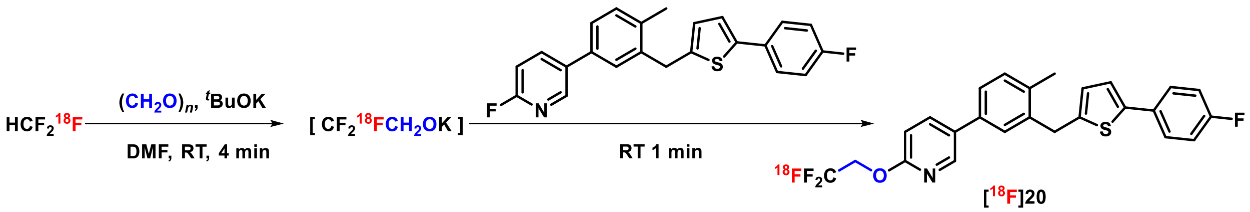

Prepared following general procedure B and analyzed using HPLC condition A.

HPLC condition A

Column: Luna C18(2) (10 μm, 250 × 4.6 mm; i.d. Phenomenex)

Solvent A: H_2O, Solvent B: MeCN

Flow rate: 2 mL/min

0–1 min: 40% B

1–3 min: 40% to 60% B

3–6 min: 60% to 95% B

6–12 min: 95% B

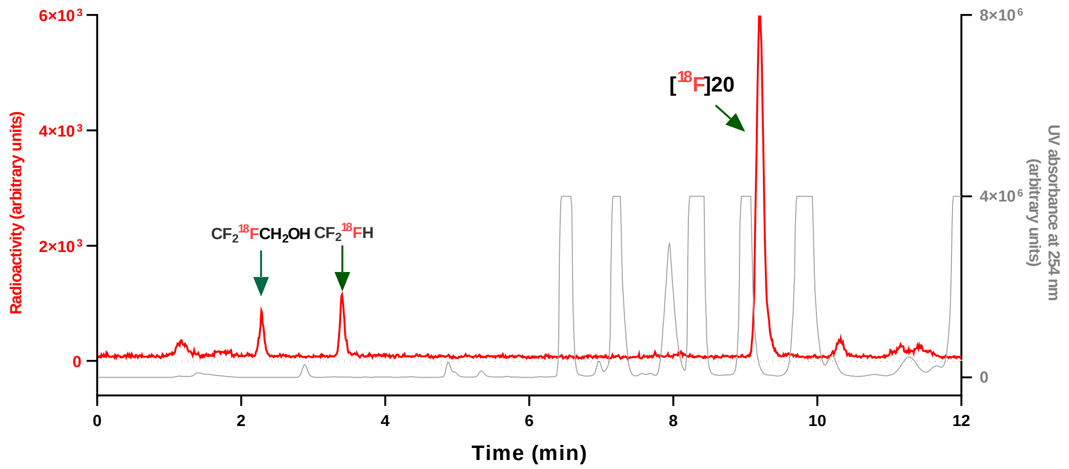

Supplementary Figure 211. Analytical HPLC chromatogram for reaction mixture

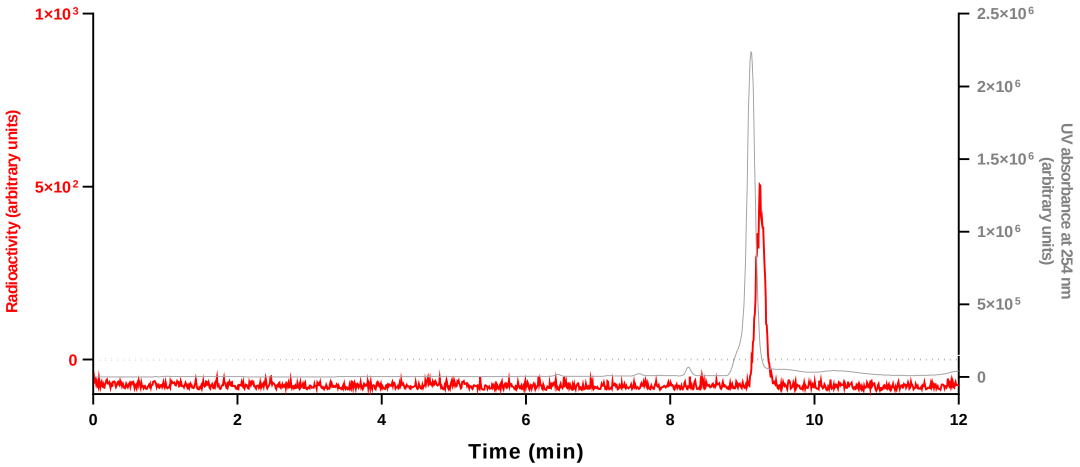

Supplementary Figure 212. Analytical HPLC chromatogram for [^{18}F]**20** collected from the above HPLC analysis when co–injected with **20.**

Run	Retention time (min)	Yield (decay-corrected) (%)	Mean yield ± SD (%)	Isolated yield (%)
1	9.248	61	66 ± 4	45
2	9.201	67		
3	9.252	69		

HPLC analysis of [^{18}F]21

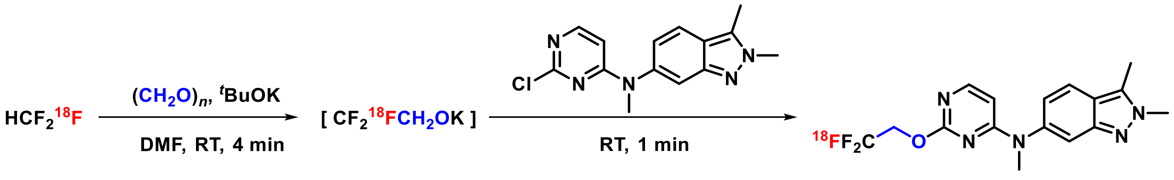

[^{18}F]21

Prepared following general procedure B and analyzed using HPLC condition A.

HPLC condition A

Column: Luna C18(2) (10 μm, 250 × 4.6 mm; i.d. Phenomenex)

Solvent A: H_2O, Solvent B: MeCN

Flow rate: 2 mL/min

0–1 min: 40% B

1–3 min: 40% to 60% B

3–6 min: 60% to 95% B

6–12 min: 95% B

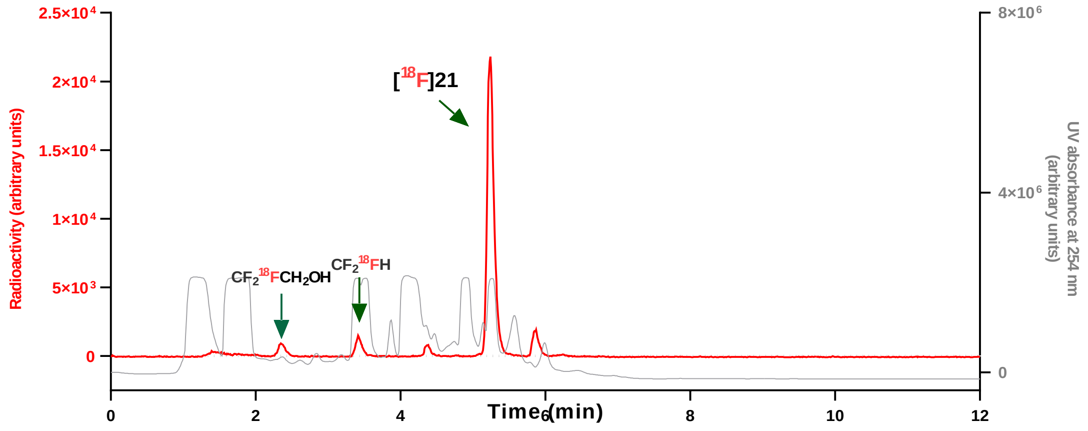

Supplementary Figure 213. Analytical HPLC chromatogram for reaction mixture.

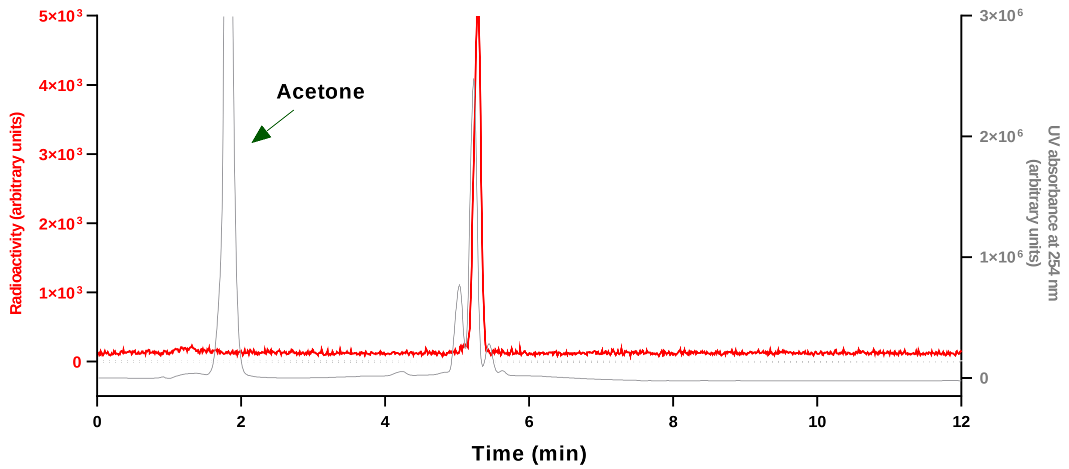

Supplementary Figure 214. Analytical HPLC chromatogram for [^{18}F]**21** collected from the above HPLC analysis when co–injected with **21.**

Run	Retention time (min)	Yield (decay-corrected) (%)	Mean yield ± SD (%)	Isolated yield (%)
1	5.240	75	69 ± 6	67
2	5.228	63		
3	5.233	69		

HPLC analysis of [^{18}F]22

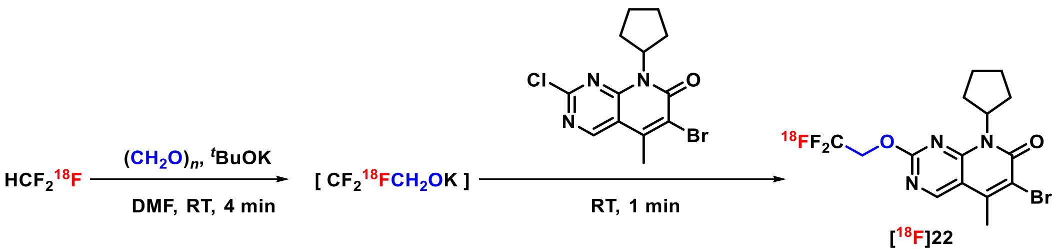

Prepared following general procedure B and analyzed using HPLC condition A.

HPLC condition A

Column: Luna C18(2) (10 μm, 250 × 4.6 mm; i.d. Phenomenex)

Solvent A: H_2O, Solvent B: MeCN

Flow rate: 2 mL/min

0–1 min: 40% B

1–3 min: 40% to 60% B

3–6 min: 60% to 95% B

6–12 min: 95% B

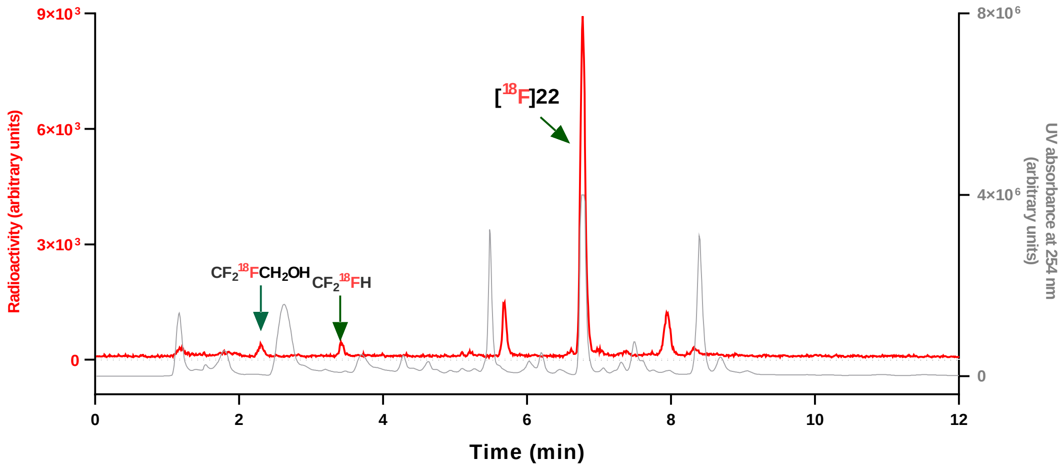

Supplementary Figure 215. Analytical HPLC chromatogram for reaction mixture.

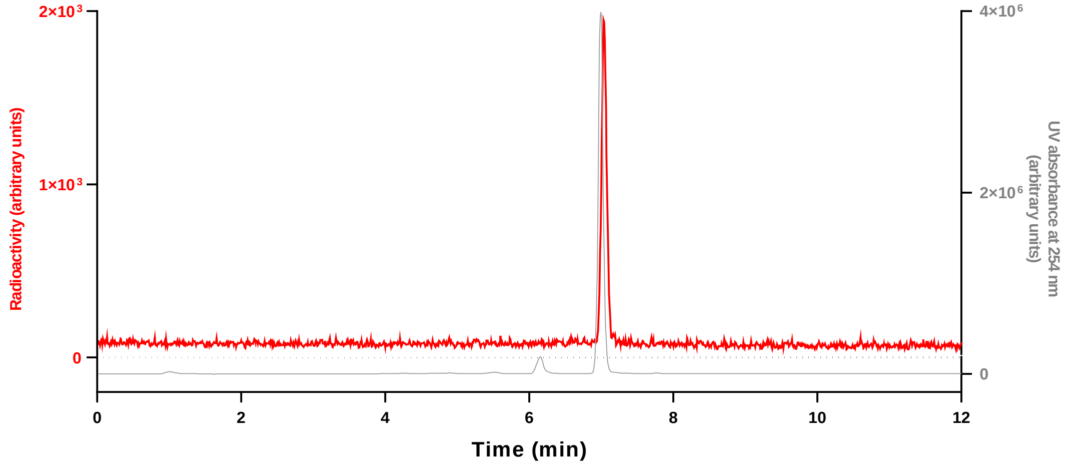

Supplementary Figure 216. Analytical HPLC chromatogram for [^{18}F]**22** collected from the above HPLC analysis when co–injected with **22.**

Run	Retention time (min)	Yield (decay-corrected) (%)	Mean yield ± SD (%)	Isolated yield (%)
1	6.986	54	61 ± 6	65
2	6.774	63		
3	7.056	66		

HPLC analysis of [^{18}F]27

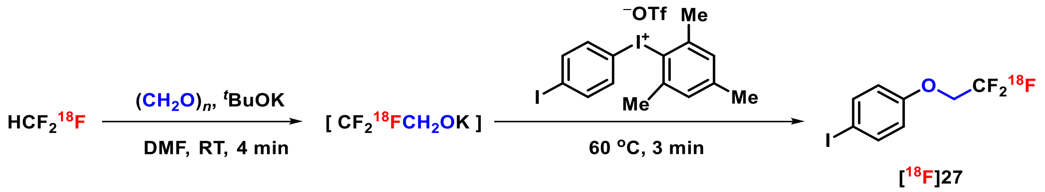

Prepared following general procedure B and analyzed using HPLC condition A.

HPLC condition A

Column: Luna C18(2) (10 μm, 250 × 4.6 mm; i.d. Phenomenex)

Solvent A: H_2O, Solvent B: MeCN

Flow rate: 2 mL/min

0–1 min: 40% B

1–3 min: 40% to 60% B

3–6 min: 60% to 95% B

6–12 min: 95% B

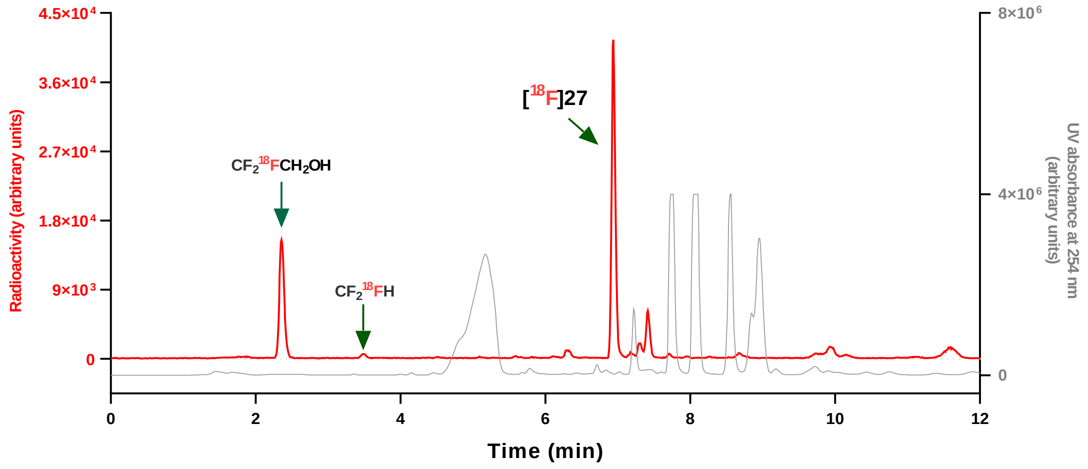

Supplementary Figure 217. Analytical HPLC chromatogram for reaction mixture.

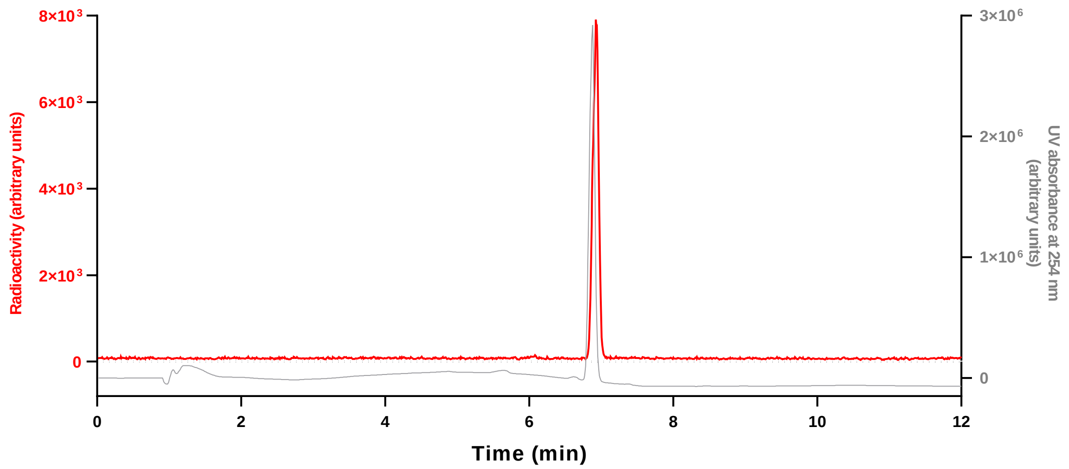

Supplementary Figure 218. Analytical HPLC chromatogram for [^{18}F]**27** collected from the above HPLC analysis when co–injected with **27.**

Run	Retention time (min)	Yield (decay-corrected) (%)	Mean yield ± SD (%)	Isolated yield (%)
1	6.908	52	49 ± 3	43
2	6.939	48		
3	6.908	47		

HPLC analysis of [^{18}F]31

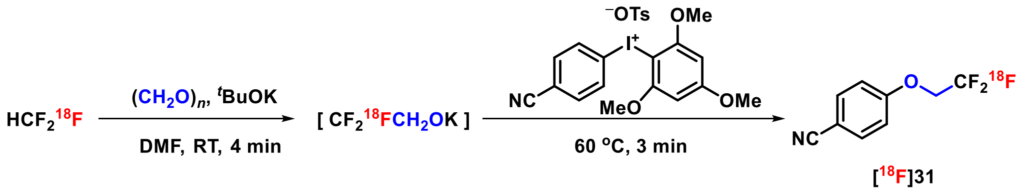

Prepared following general procedure B and analyzed using HPLC condition A.

HPLC condition A

Column: Luna C18(2) (10 μm, 250 × 4.6 mm; i.d. Phenomenex)

Solvent A: H_2O, Solvent B: MeCN

Flow rate: 2 mL/min

0–1 min: 40% B

1–3 min: 40% to 60% B

3–6 min: 60% to 95% B

6–12 min: 95% B

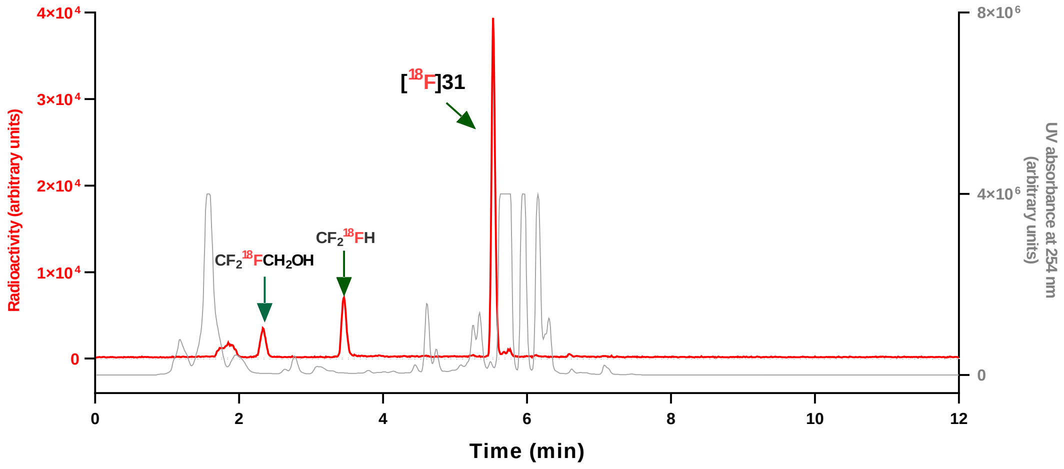

Supplementary Figure 219. Analytical HPLC chromatogram for reaction mixture.

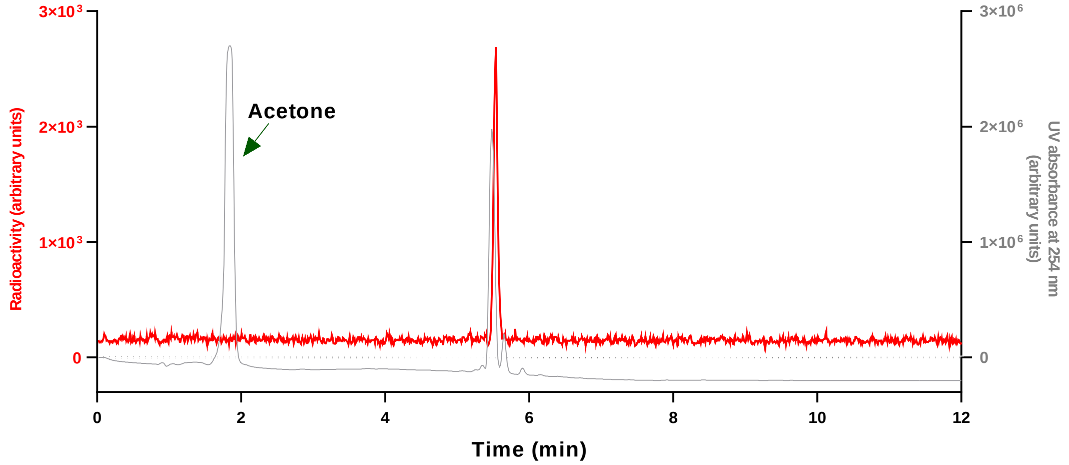

Supplementary Figure 220. Analytical HPLC chromatogram for [^{18}F]**33** collected from the above HPLC analysis when co–injected with **33.**

Run	Retention time (min)	Yield (decay-corrected) (%)	Mean yield ± SD (%)	Isolated yield (%)
1	5.480	76	69 ± 6	58
2	5.532	65		
3	5.475	67		

HPLC analysis of [^{18}F]35

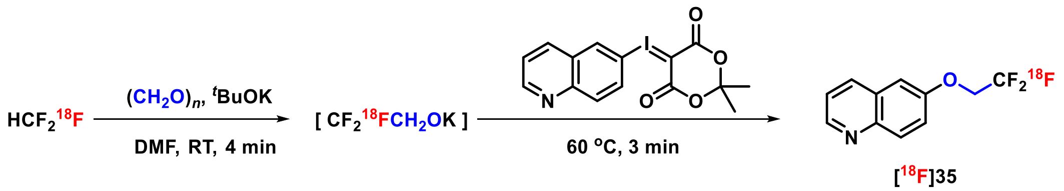

Prepared following general procedure B and analyzed using HPLC condition A.

HPLC condition A

Column: Luna C18(2) (10 μm, 250 × 4.6 mm; i.d. Phenomenex)

Solvent A: H_2O, Solvent B: MeCN

Flow rate: 2 mL/min

0–1 min: 40% B

1–3 min: 40% to 60% B

3–6 min: 60% to 95% B

6–12 min: 95% B

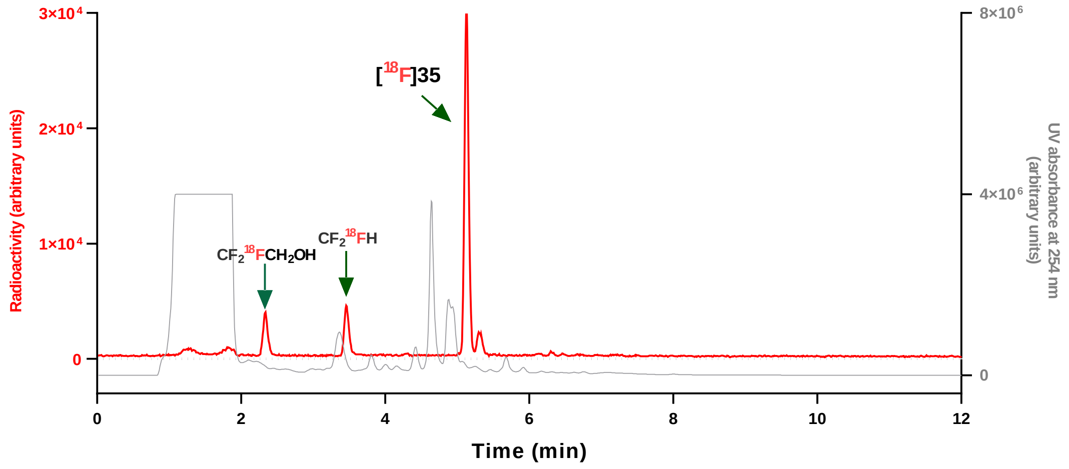

Supplementary Figure 221. Analytical HPLC chromatogram for reaction mixture.

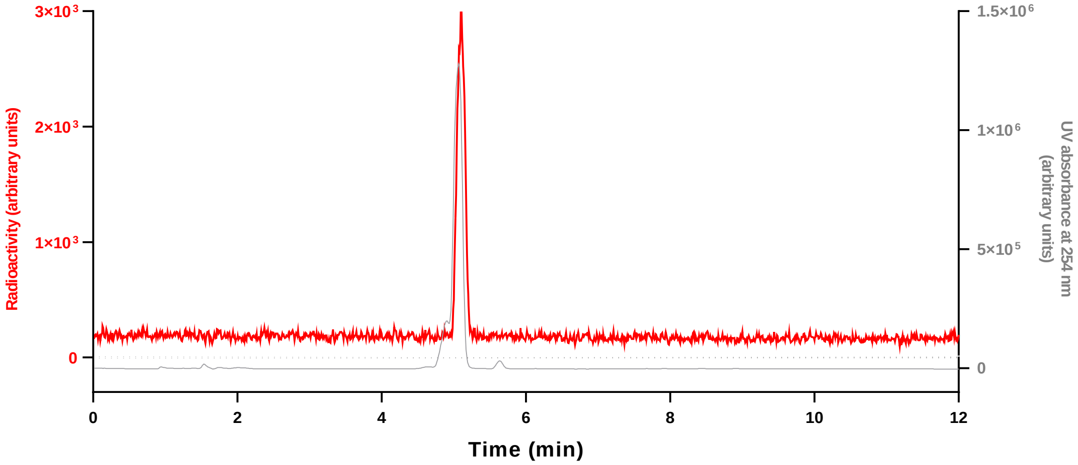

Supplementary Figure 222. Analytical HPLC chromatogram for [^{18}F]**35** collected from the above HPLC analysis when co–injected with **35.**

Run	Retention time (min)	Yield (decay-corrected) (%)	Mean yield ± SD (%)	Isolated yield (%)
1	5.075	73	68 ± 4	55
2	5.131	66		
3	5.078	66		

HPLC analysis of [^{18}F]36

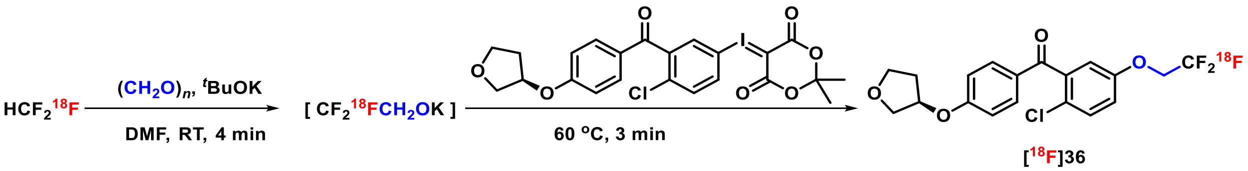

[^{18}F]36

Prepared following general procedure B and analyzed using HPLC condition A.

HPLC condition A

Column: Luna C18(2) (10 μm, 250 × 4.6 mm; i.d. Phenomenex)

Solvent A: H_2O, Solvent B: MeCN

Flow rate: 2 mL/min

0–1 min: 40% B

1–3 min: 40% to 60% B

3–6 min: 60% to 95% B

6–12 min: 95% B

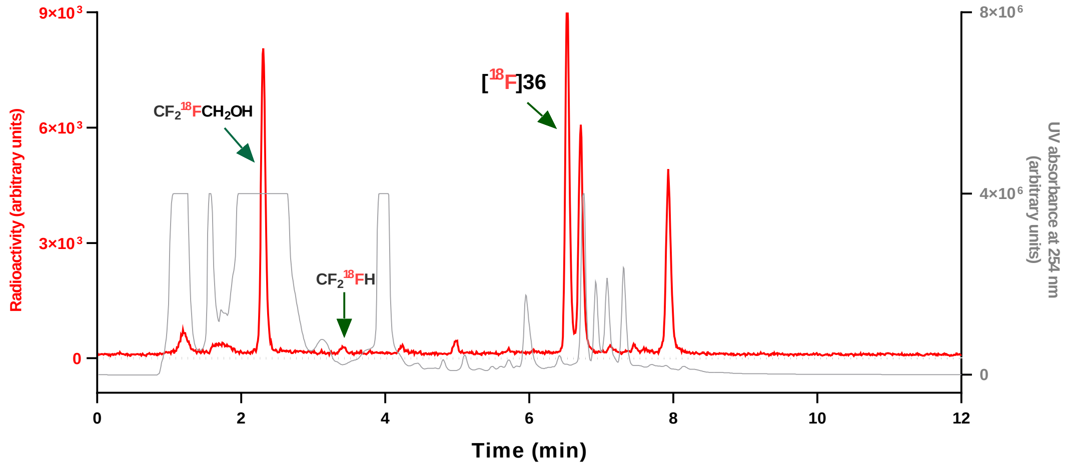

Supplementary Figure 223. Analytical HPLC chromatogram for reaction mixture.

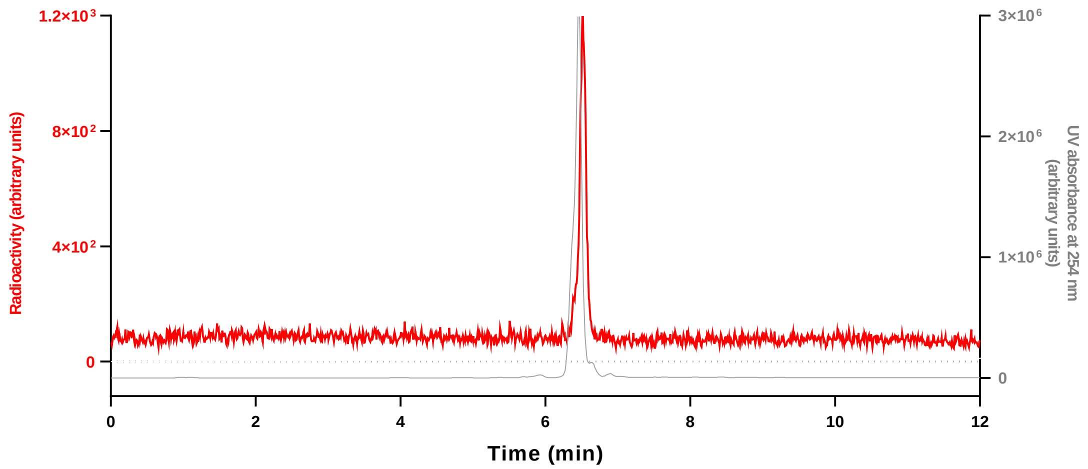

Supplementary Figure 224. Analytical HPLC chromatogram for [^{18}F]**36** collected from the above HPLC analysis when co–injected with **36.**

Run	Retention time (min)	Yield (decay-corrected) (%)	Mean yield ± SD (%)	Isolated yield (%)
1	6.586	25	27 ± 2	21
2	6.554	27		
3	6.527	29		

HPLC analysis of [^{18}F]37

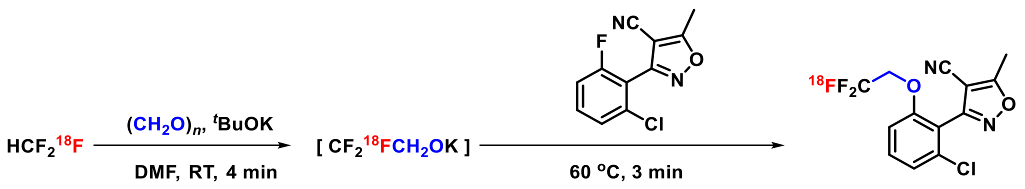

Prepared following general procedure B and analyzed using HPLC condition A.

HPLC condition A

Column: Luna C18(2) (10 μm, 250 × 4.6 mm; i.d. Phenomenex)

Solvent A: H_2O, Solvent B: MeCN

Flow rate: 2 mL/min

0–1 min: 40% B

1–3 min: 40% to 60% B

3–6 min: 60% to 95% B

6–12 min: 95% B

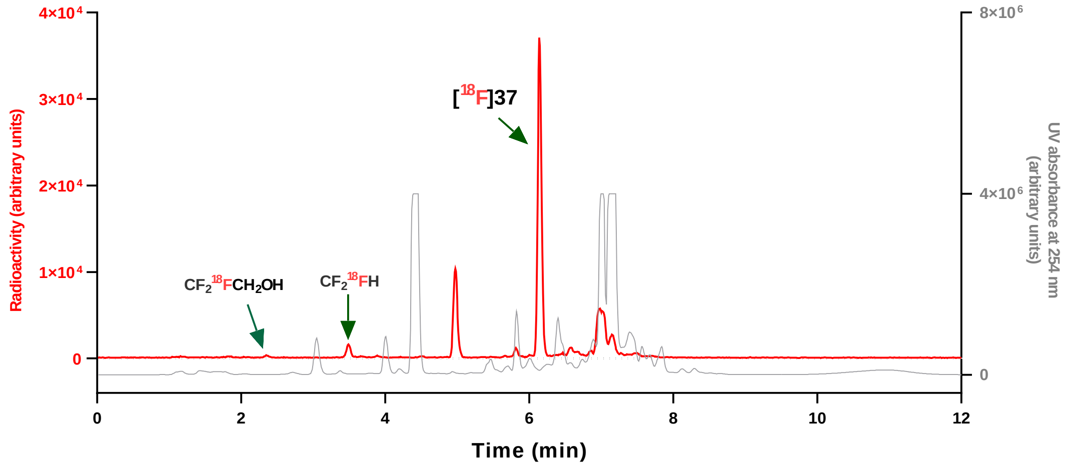

Supplementary Figure 225. Analytical HPLC chromatogram for reaction mixture.

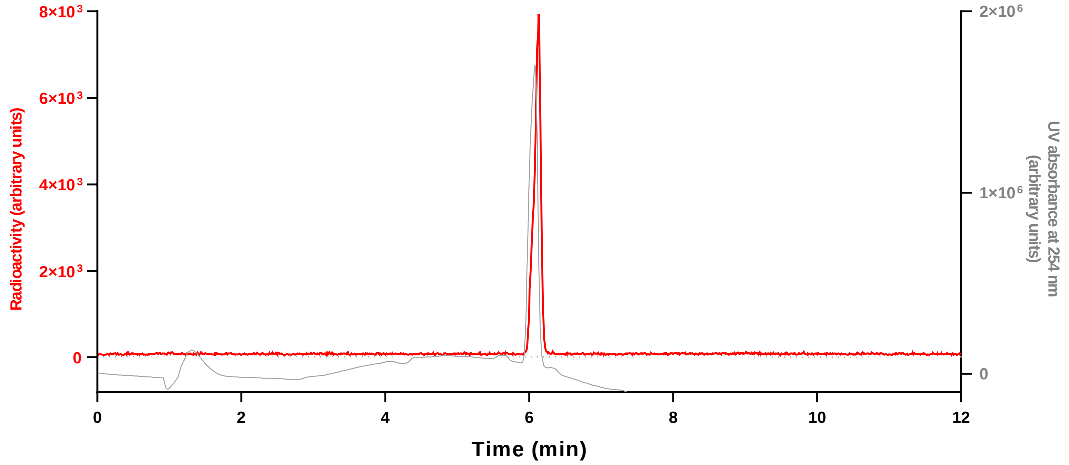

Supplementary Figure 226. Analytical HPLC chromatogram for [^{18}F]**37** collected from the above HPLC analysis when co–injected with **37.**

Run	Retention time (min)	Yield (decay-corrected) (%)	Mean yield ± SD (%)	Isolated yield (%)
1	6.075	51	52 ± 2	51
2	6.144	51		
3	6.064	54		

HPLC analysis of [^{18}F]38

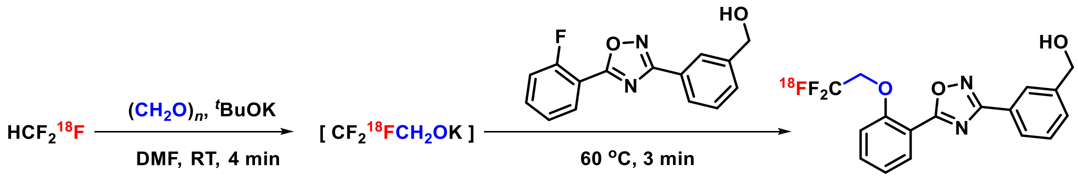

[^{18}F]38

Prepared following general procedure B and analyzed using HPLC condition A.

HPLC condition A

Column: Luna C18(2) (10 μm, 250 × 4.6 mm; i.d. Phenomenex)

Solvent A: H_2O, Solvent B: MeCN

Flow rate: 2 mL/min

0–1 min: 40% B

1–3 min: 40% to 60% B

3–6 min: 60% to 95% B

6–12 min: 95% B

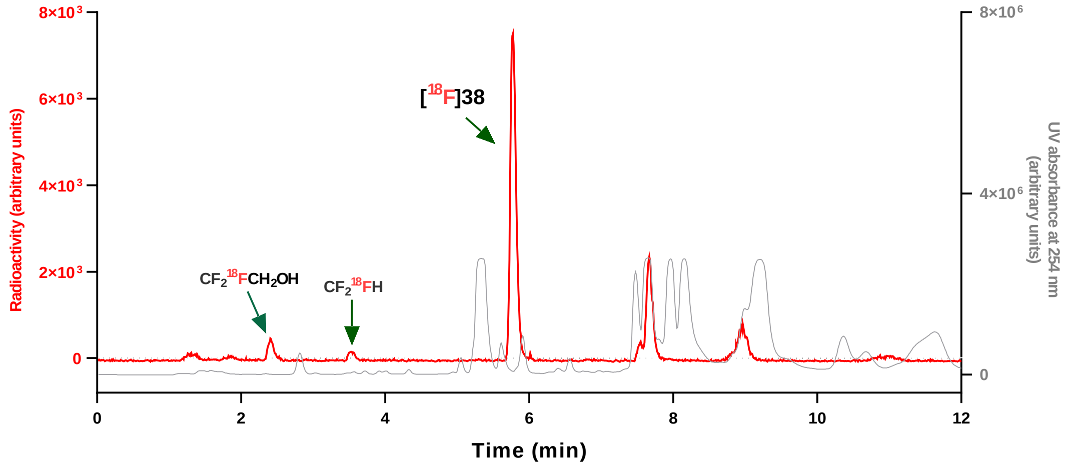

Supplementary Figure 227. Analytical HPLC chromatogram for reaction mixture.

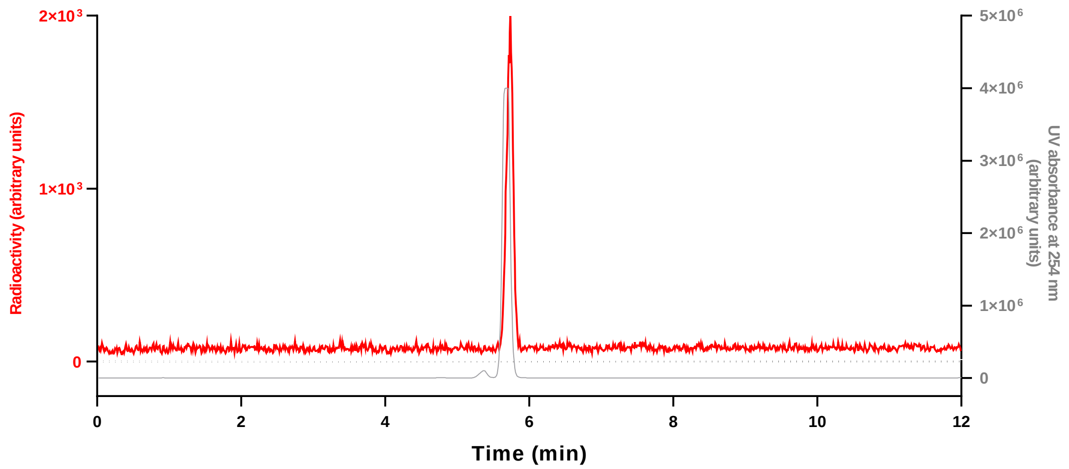

Supplementary Figure 228. Analytical HPLC chromatogram for [^{18}F]**38** collected from the above HPLC analysis when co-injected with **38.**

Run	Retention time (min)	Yield (decay-corrected) (%)	Mean yield ± SD (%)	Isolated yield (%)
1	5.773	55	53 ± 2	48
2	6.865	52		
3	5.772	53		

HPLC analysis of [^{18}F]44

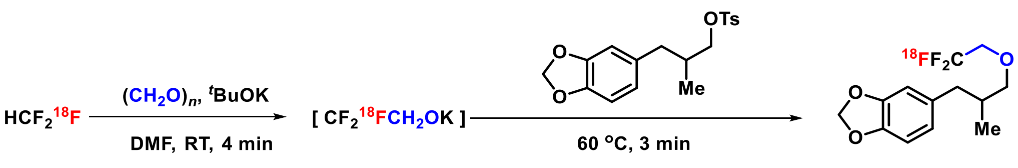

[^{18}F]44

Prepared following general procedure B and analyzed using HPLC condition A.

HPLC condition A

Column: Luna C18(2) (10 μm, 250 × 4.6 mm; i.d. Phenomenex)

Solvent A: H_2O, Solvent B: MeCN

Flow rate: 2 mL/min

0–1 min: 40% B

1–3 min: 40% to 60% B

3–6 min: 60% to 95% B

6–12 min: 95% B

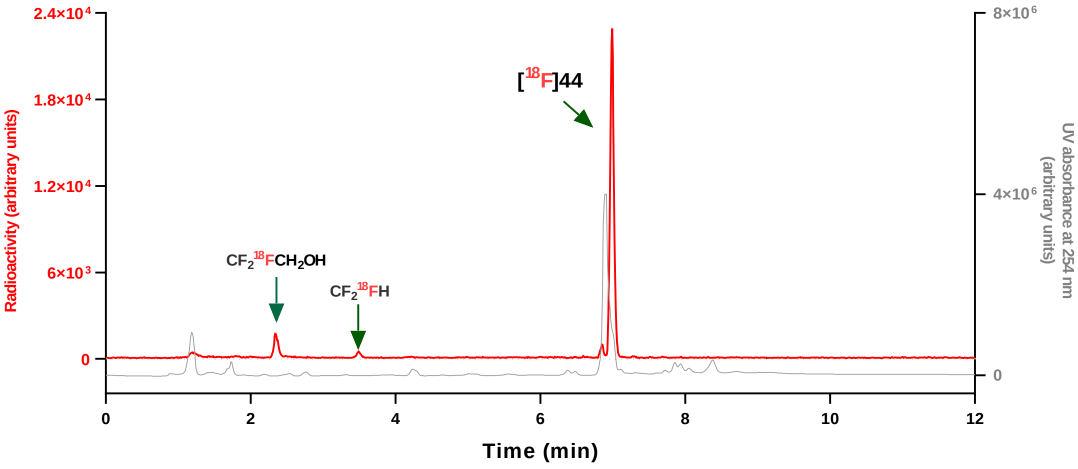

Supplementary Figure 229. Analytical HPLC chromatogram for reaction mixture.

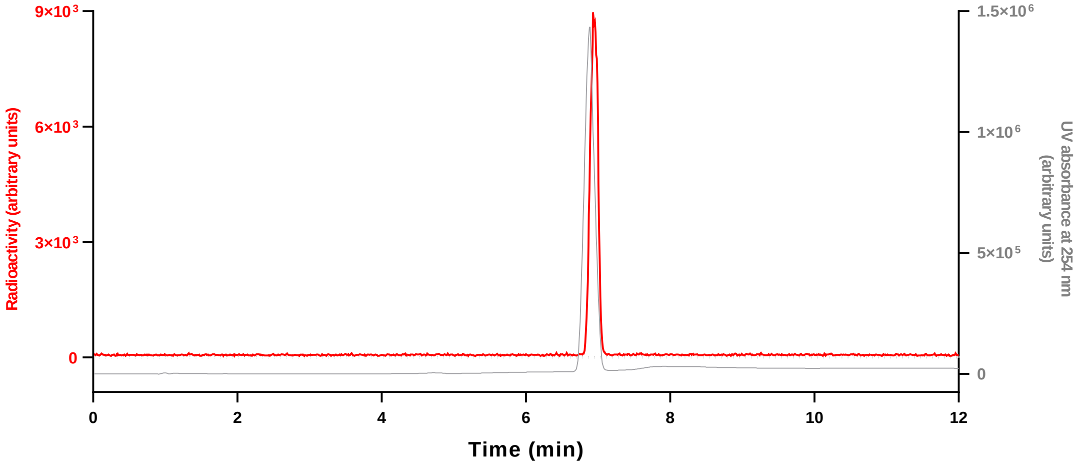

Supplementary Figure 230. Analytical HPLC chromatogram for [^{18}F]**44** collected from the above HPLC analysis when co–injected with **44.**

Run	Retention time (min)	Yield (decay-corrected) (%)	Mean yield ± SD (%)	Isolated yield (%)
1	6.990	84	83 ± 1	79
2	6.984	82		
3	6.904	84		

HPLC analysis of [^{18}F]45

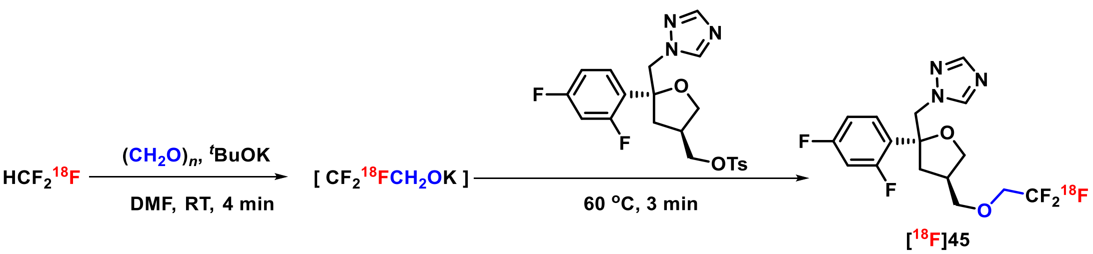

Prepared following general procedure B and analyzed using HPLC condition A.

HPLC condition A

Column: Luna C18(2) (10 μm, 250 × 4.6 mm; i.d. Phenomenex)

Solvent A: H_2O, Solvent B: MeCN

Flow rate: 2 mL/min

0–1 min: 40% B

1–3 min: 40% to 60% B

3–6 min: 60% to 95% B

6–12 min: 95% B

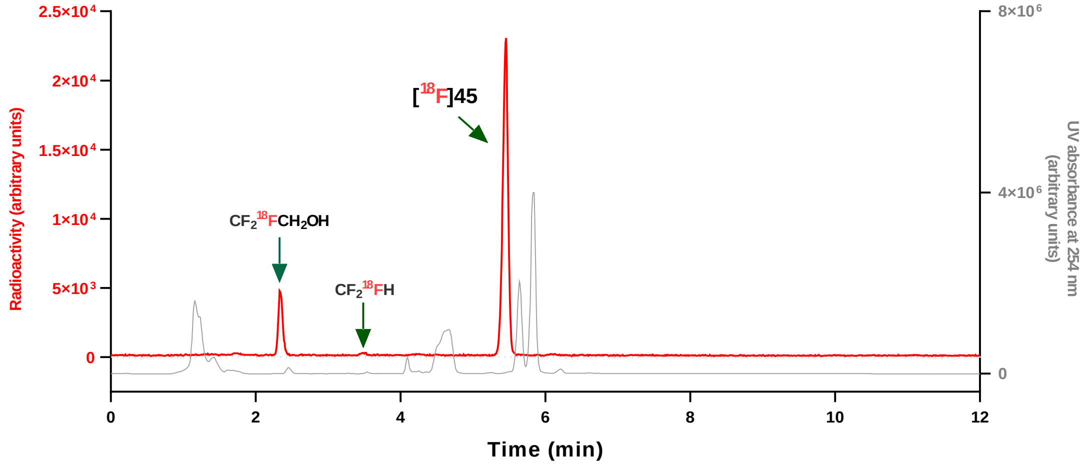

Supplementary Figure 231. Analytical HPLC chromatogram for reaction mixture.

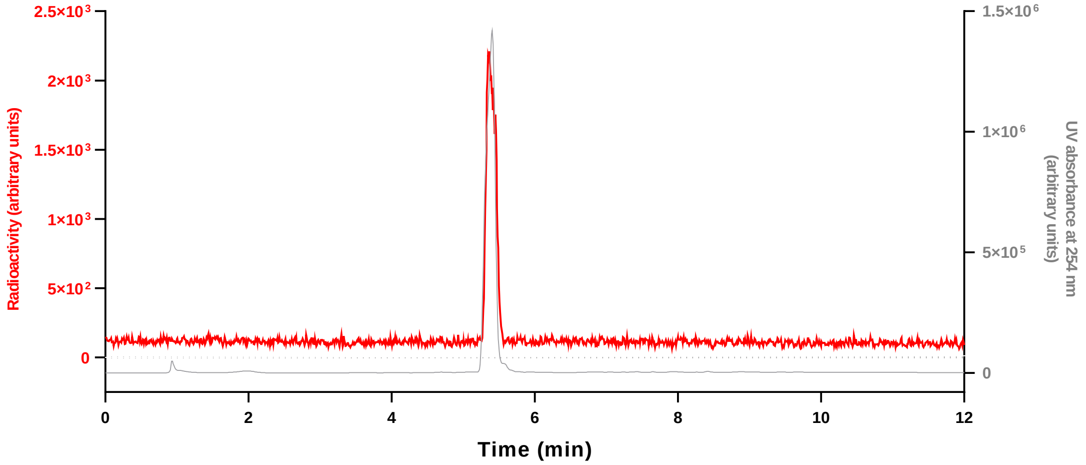

Supplementary Figure 232. Analytical HPLC chromatogram for [^{18}F]**45** collected from the above HPLC analysis when co–injected with **45.**

Run	Retention time (min)	Yield (decay-corrected) (%)	Mean yield ± SD (%)	Isolated yield (%)
1	5.498	87	87 ± 1	79
2	5.503	88		
3	5.455	86		

HPLC analysis of [^{18}F]46

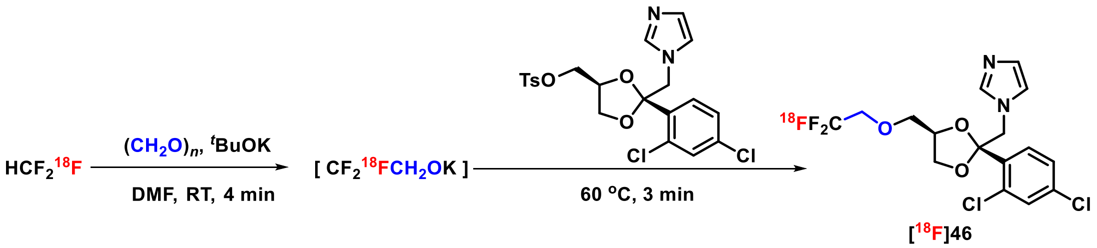

Prepared following general procedure B and analyzed using HPLC condition A.

HPLC condition A

Column: Luna C18(2) (10 μm, 250 × 4.6 mm; i.d. Phenomenex)

Solvent A: H_2O, Solvent B: MeCN

Flow rate: 2 mL/min

0–1 min: 40% B

1–3 min: 40% to 60% B

3–6 min: 60% to 95% B

6–12 min: 95% B

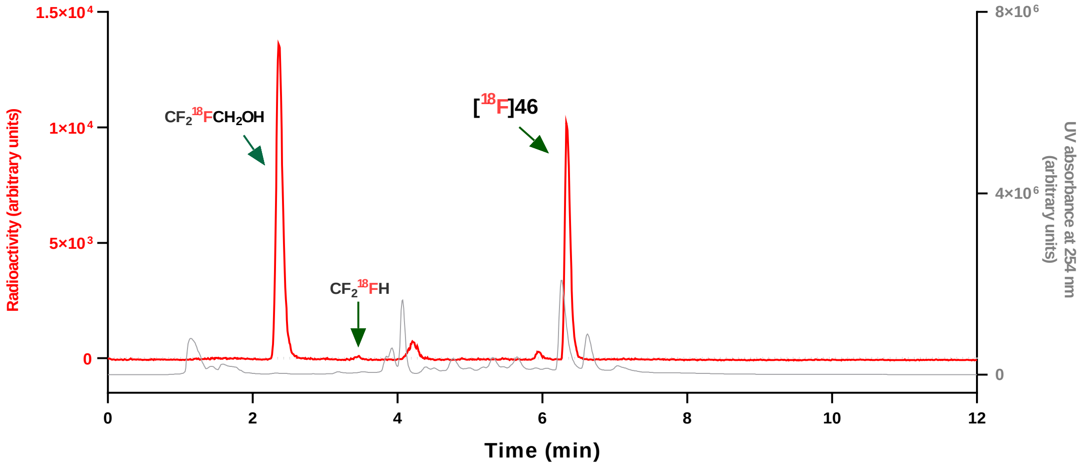

Supplementary Figure 233. Analytical HPLC chromatogram for reaction mixture.

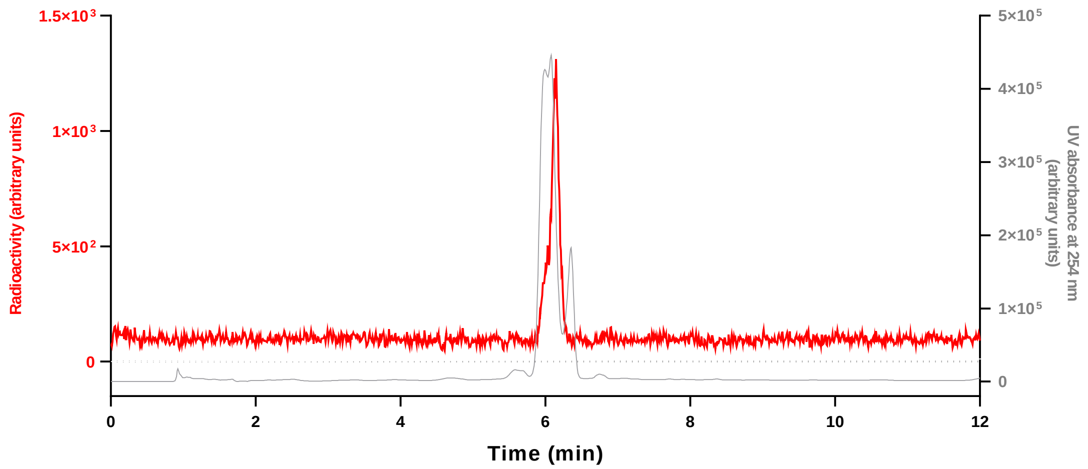

Supplementary Figure 234. Analytical HPLC chromatogram for [^{18}F]**46** collected from the above HPLC analysis when co–injected with **46.**

Run	Retention time (min)	Yield (decay-corrected) (%)	Mean yield ± SD (%)	Isolated yield (%)
1	6.345	35	36 ± 1	31
2	6.337	36		
3	6.236	37		

HPLC analysis of [^{18}F]49

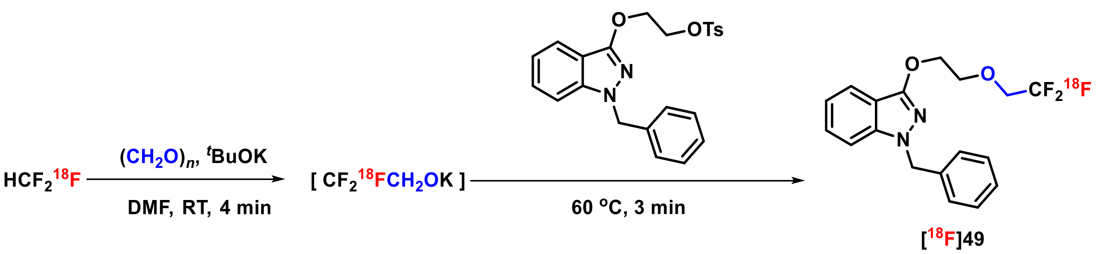

Prepared following general procedure B and analyzed using HPLC condition A.

HPLC condition A

Column: Luna C18(2) (10 μm, 250 × 4.6 mm; i.d. Phenomenex)

Solvent A: H_2O, Solvent B: MeCN

Flow rate: 2 mL/min

0–1 min: 40% B

1–3 min: 40% to 60% B

3–6 min: 60% to 95% B

6–12 min: 95% B

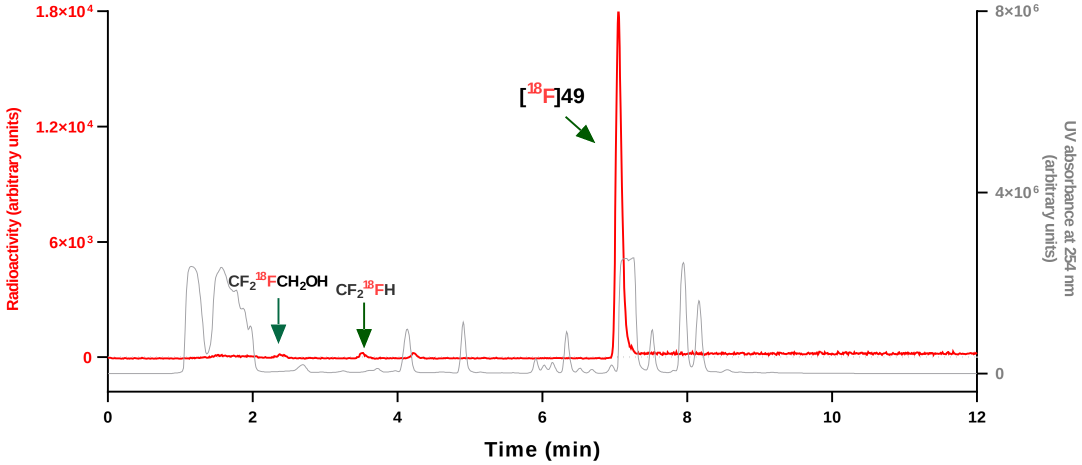

Supplementary Figure 235. Analytical HPLC chromatogram for reaction mixture.

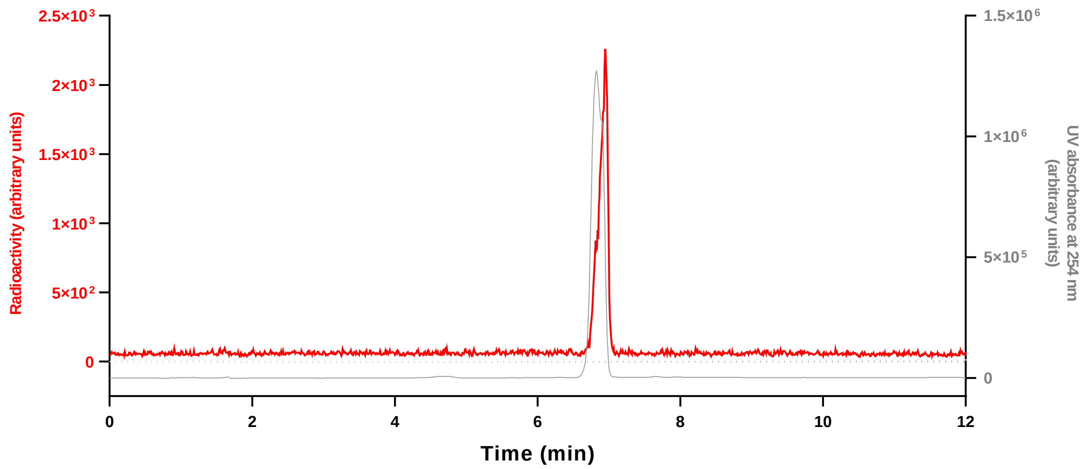

Supplementary Figure 236. Analytical HPLC chromatogram for [18F]**49** collected from the above HPLC analysis when co–injected with **49.**

Run	Retention time (min)	Yield (decay-corrected) (%)	Mean yield ± SD (%)	Isolated yield (%)
1	6.099	86	90 ± 4	75
2	7.051	94		
3	6.979	91		

HPLC analysis of [^{18}F]50

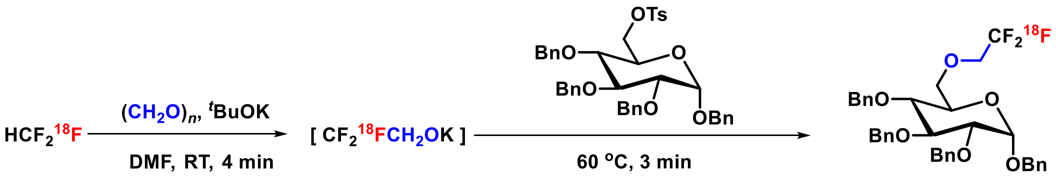

[^{18}F]50

Prepared following general procedure B and analyzed using HPLC condition A.

HPLC condition A

Column: Luna C18(2) (10 μm, 250 × 4.6 mm; i.d. Phenomenex)

Solvent A: H_2O, Solvent B: MeCN

Flow rate: 2 mL/min

0–1 min: 40% B

1–3 min: 40% to 60% B

3–6 min: 60% to 95% B

6–12 min: 95% B

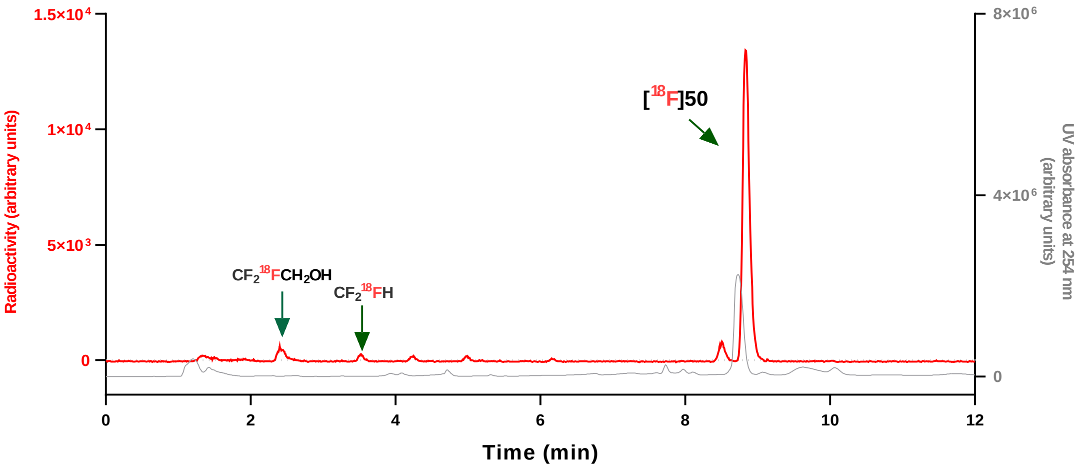

Supplementary Figure 237. Analytical HPLC chromatogram for reaction mixture.

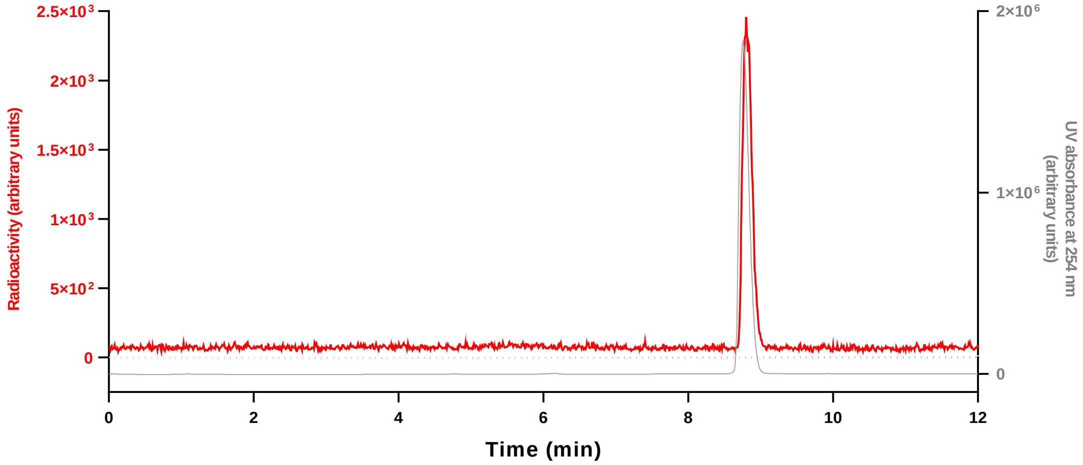

Supplementary Figure 238. Analytical HPLC chromatogram for [^{18}F]**50** collected from the above HPLC analysis when co–injected with **50**

Run	Retention time (min)	Yield (decay-corrected) (%)	Mean yield ± SD (%)	Isolated yield (%)
1	8.857	82	84 ± 3	76
2	8.837	82		
3	8.844	87		

HPLC analysis of [^{18}F]51

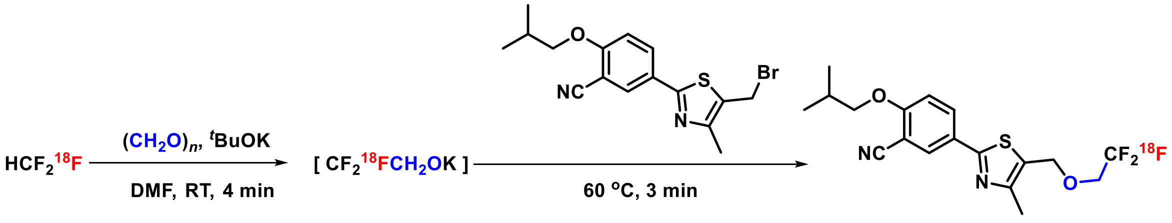

Prepared following general procedure B and analyzed using HPLC condition A.

HPLC condition A

Column: Luna C18(2) (10 μm, 250 × 4.6 mm; i.d. Phenomenex)

Solvent A: H_2O, Solvent B: MeCN

Flow rate: 2 mL/min

0–1 min: 40% B

1–3 min: 40% to 60% B

3–6 min: 60% to 95% B

6–12 min: 95% B

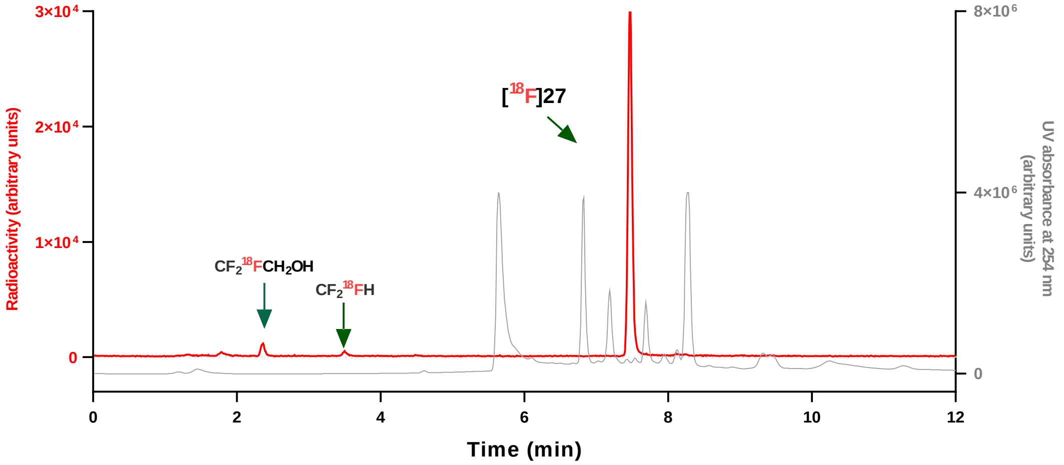

Supplementary Figure 239. Analytical HPLC chromatogram for reaction mixture.

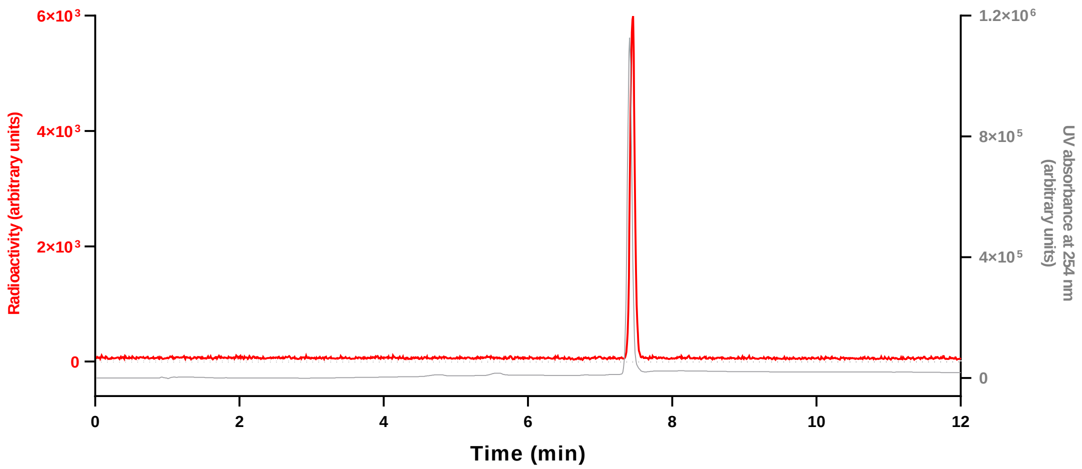

Supplementary Figure 240. Analytical HPLC chromatogram for [^{18}F]**51** collected from the above HPLC analysis when co–injected with **51.**

Run	Retention time (min)	Yield (decay-corrected) (%)	Mean yield ± SD (%)	Isolated yield (%)
1	7.472	64	83 ± 16	80
2	7.472	91		
3	7.467	93		

HPLC analysis of [^{18}F]52

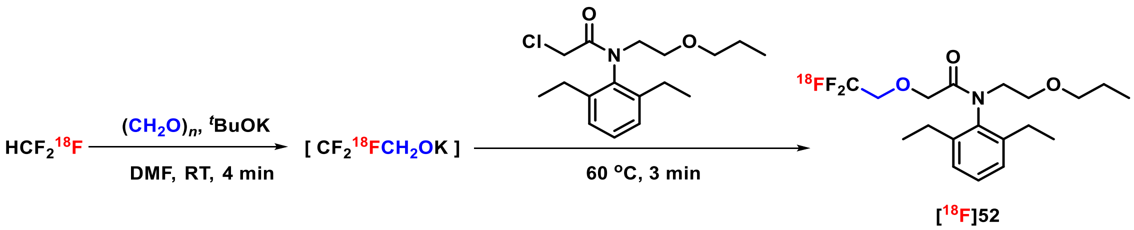

Prepared following general procedure B and analyzed using HPLC condition A.

HPLC condition A

Column: Luna C18(2) (10 μm, 250 × 4.6 mm; i.d. Phenomenex)

Solvent A: H_2O, Solvent B: MeCN

Flow rate: 2 mL/min

0–1 min: 40% B

1–3 min: 40% to 60% B

3–6 min: 60% to 95% B

6–12 min: 95% B

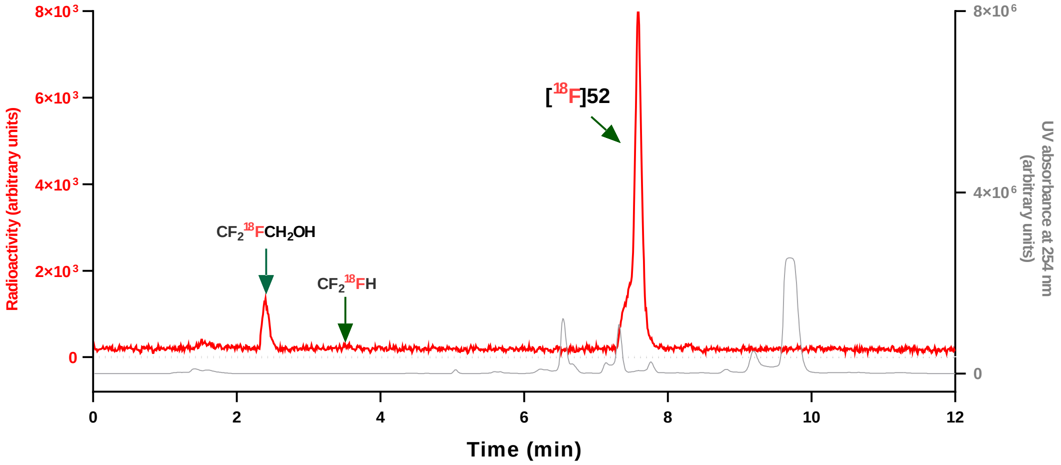

Supplementary Figure 241. Analytical HPLC chromatogram for reaction mixture.

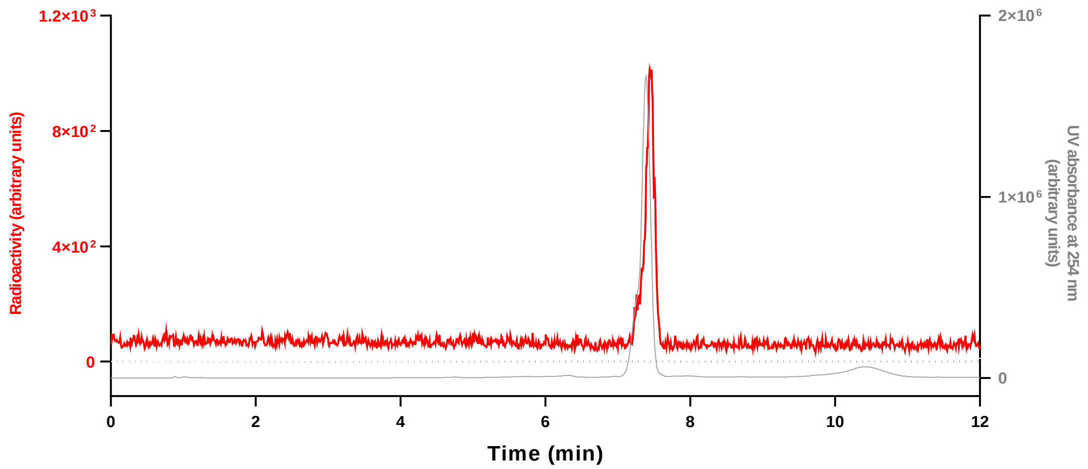

Supplementary Figure 242. Analytical HPLC chromatogram for [^{18}F]**52** collected from the above HPLC analysis when co–injected with **52.**

Run	Retention time (min)	Yield (decay-corrected) (%)	Mean yield ± SD (%)	Isolated yield (%)
1	7.558	80	75 ± 5	70
2	7.530	73		
3	7.516	71		

HPLC analysis of [^{18}F]53

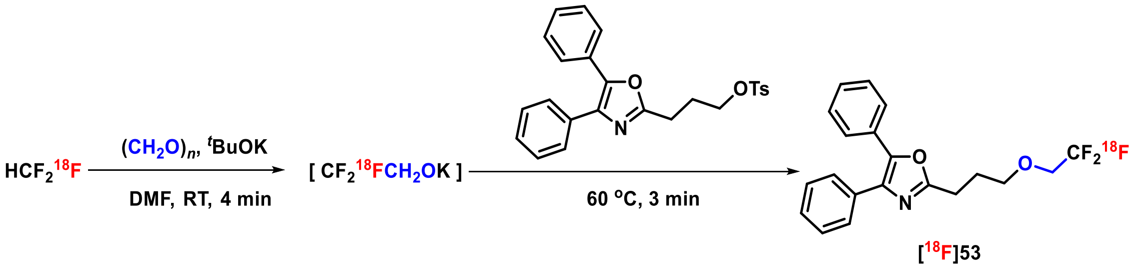

Prepared following general procedure B and analyzed using HPLC condition A.

HPLC condition A

Column: Luna C18(2) (10 μm, 250 × 4.6 mm; i.d. Phenomenex)

Solvent A: H_2O, Solvent B: MeCN

Flow rate: 2 mL/min

0–1 min: 40% B

1–3 min: 40% to 60% B

3–6 min: 60% to 95% B

6–12 min: 95% B

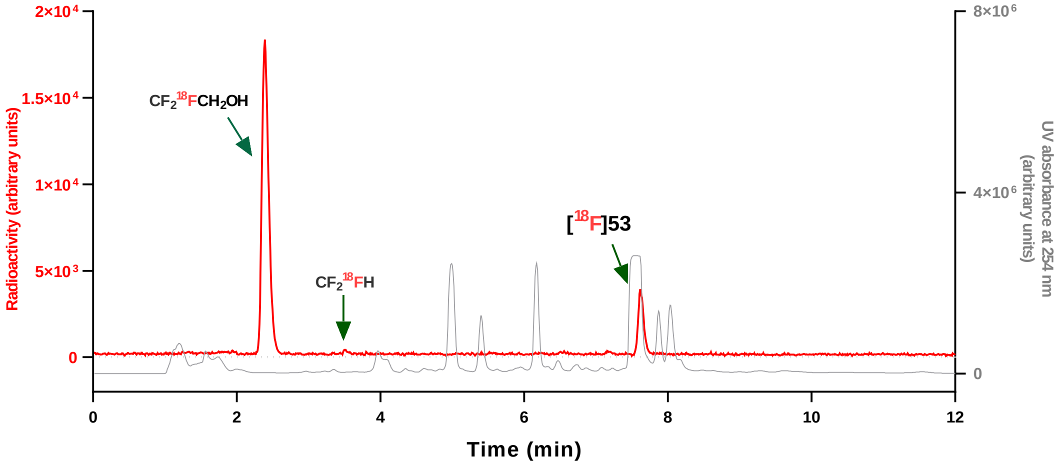

Supplementary Figure 243. Analytical HPLC chromatogram for reaction mixture.

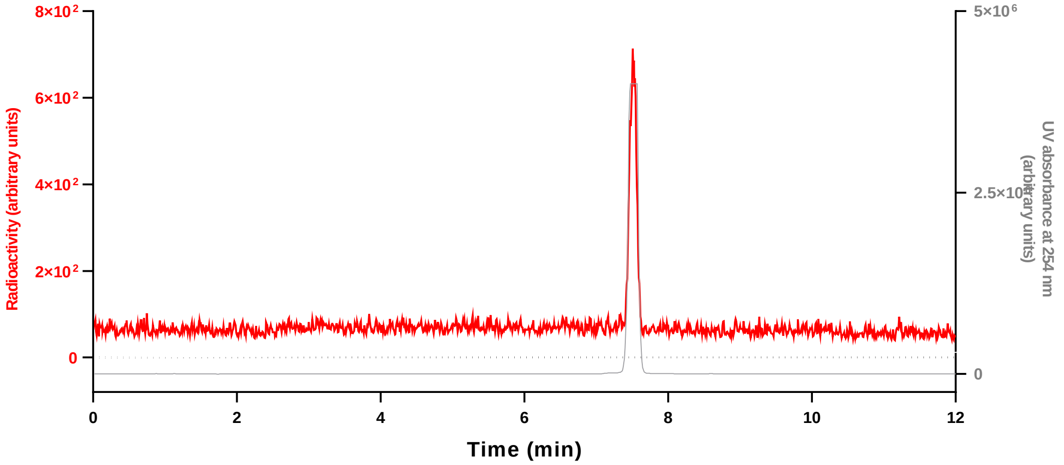

Supplementary Figure 244. Analytical HPLC chromatogram for [^{18}F]**53** collected from the above HPLC analysis when co–injected with **53.**

Run	Retention time (min)	Yield (decay-corrected) (%)	Mean yield ± SD (%)	Isolated yield (%)
1	7.621	15	15 ± 1	13
2	7.620	15		
3	7.622	14		

HPLC analysis of [^{18}F]54

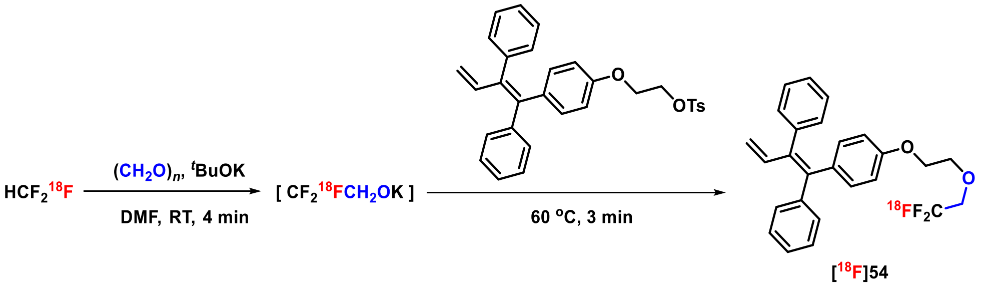

Prepared following general procedure B and analyzed using HPLC condition A.
HPLC condition A

Column: Luna C18(2) (10 μm, 250 × 4.6 mm; i.d. Phenomenex)

Solvent A: H_2O, Solvent B: MeCN

Flow rate: 2 mL/min

0–1 min: 40% B

1–3 min: 40% to 60% B

3–6 min: 60% to 95% B

6–12 min: 95% B

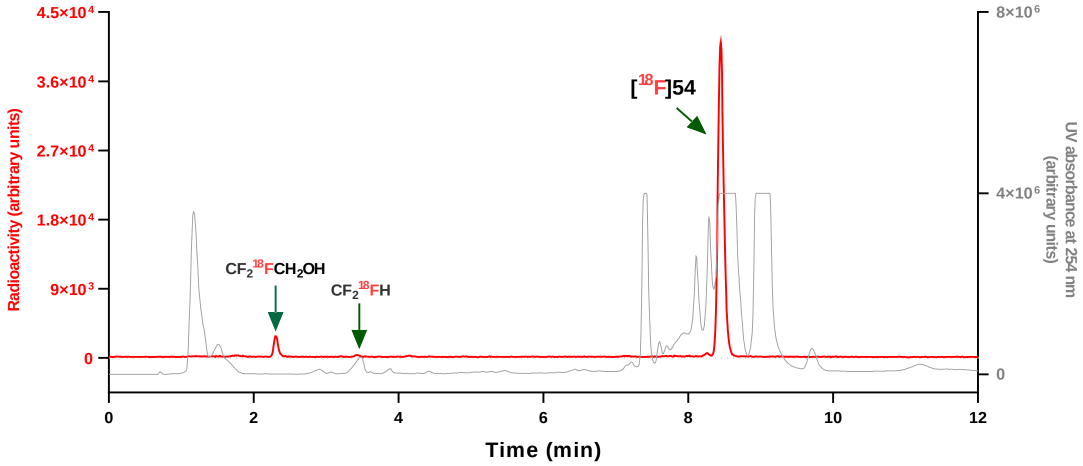

Supplementary Figure 245. Analytical HPLC chromatogram for reaction mixture.

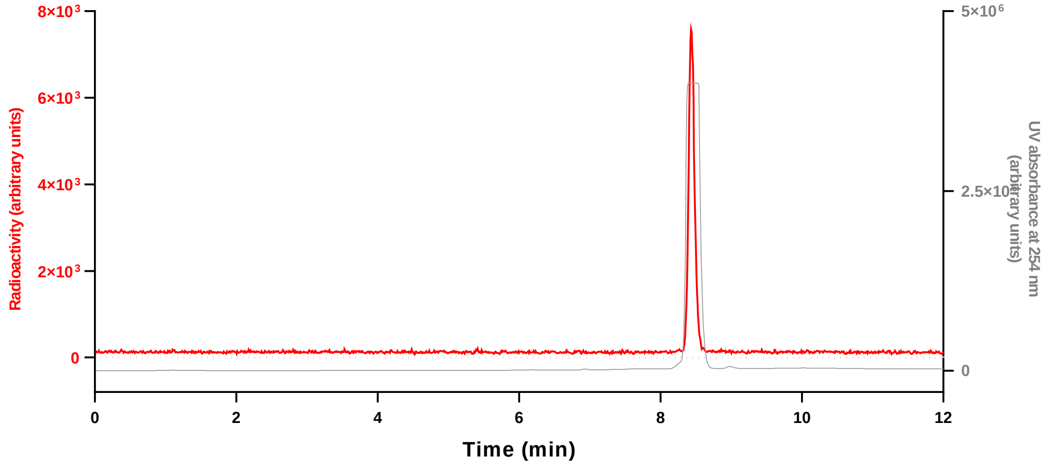

Supplementary Figure 246. Analytical HPLC chromatogram for [^{18}F]**54** collected from the above HPLC analysis when co–injected with **54.**

Run	Retention time (min)	Yield (decay-corrected) (%)	Mean yield ± SD (%)	Isolated yield (%)
1	8.452	95	95 ± 1	86
2	8.456	95		
3	8.449	94		

HPLC analysis of [^{18}F]55

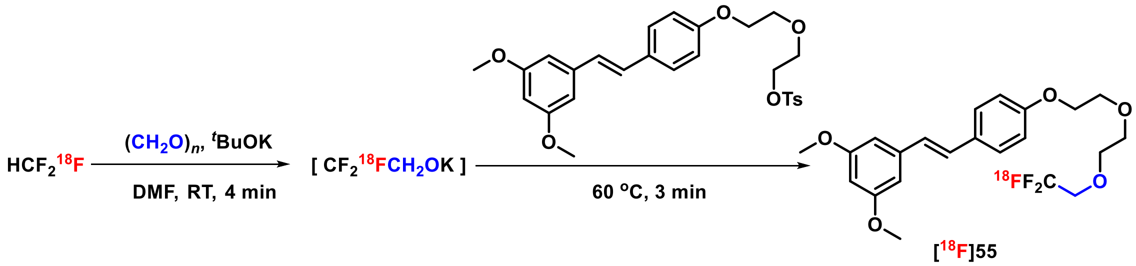

Prepared following general procedure B and analyzed using HPLC condition A.

HPLC condition A

Column: Luna C18(2) (10 μm, 250 × 4.6 mm; i.d. Phenomenex)

Solvent A: H_2O, Solvent B: MeCN

Flow rate: 2 mL/min

0–1 min: 40% B

1–3 min: 40% to 60% B

3–6 min: 60% to 95% B

6–12 min: 95% B

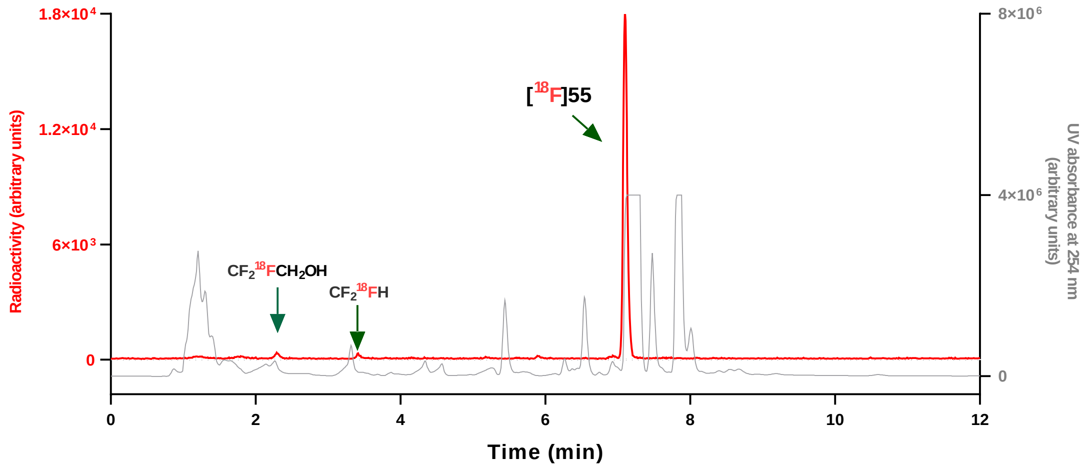

Supplementary Figure 247. Analytical HPLC chromatogram for reaction mixture.

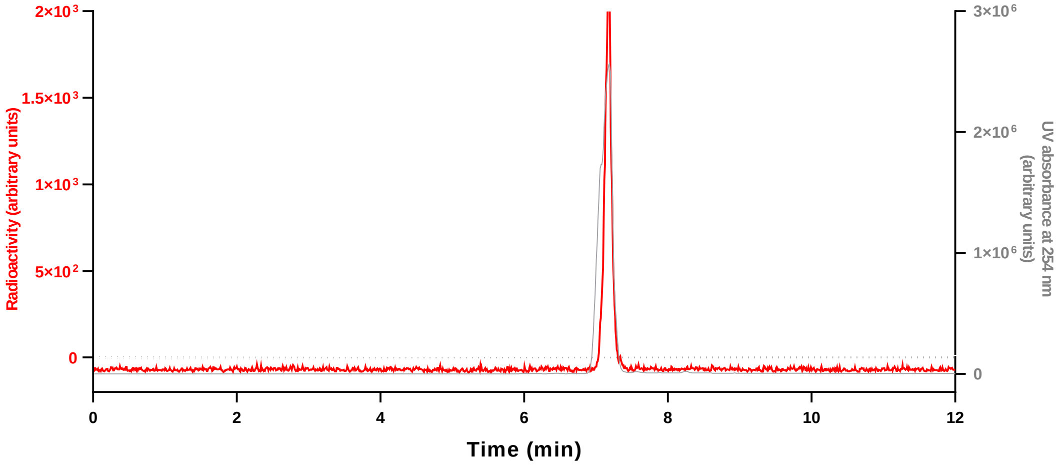

Supplementary Figure 248. Analytical HPLC chromatogram for [^{18}F]**55** collected from the above HPLC analysis when co–injected with **55.**

Run	Retention time (min)	Yield (decay-corrected) (%)	Mean yield ± SD (%)	Isolated yield (%)
1	7.102	91	94 ± 3	90
2	7.096	96		
3	7.100	96		

HPLC analysis of [¹⁸F]56

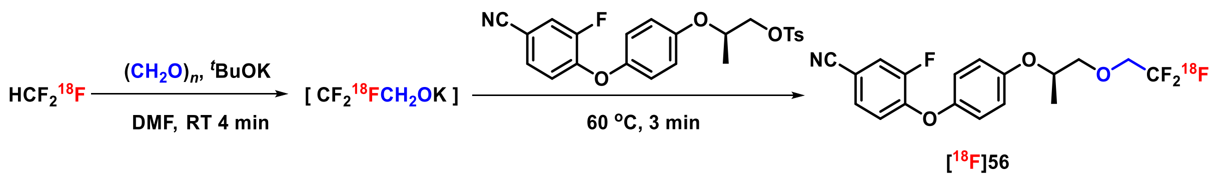

Prepared following general procedure B and analyzed using HPLC condition A.

HPLC condition A

Column: Luna C18(2) (10 μm, 250 × 4.6 mm; i.d. Phenomenex)

Solvent A: H_2O, Solvent B: MeCN

Flow rate: 2 mL/min

0–1 min: 40% B

1–3 min: 40% to 60% B

3–6 min: 60% to 95% B

6–12 min: 95% B

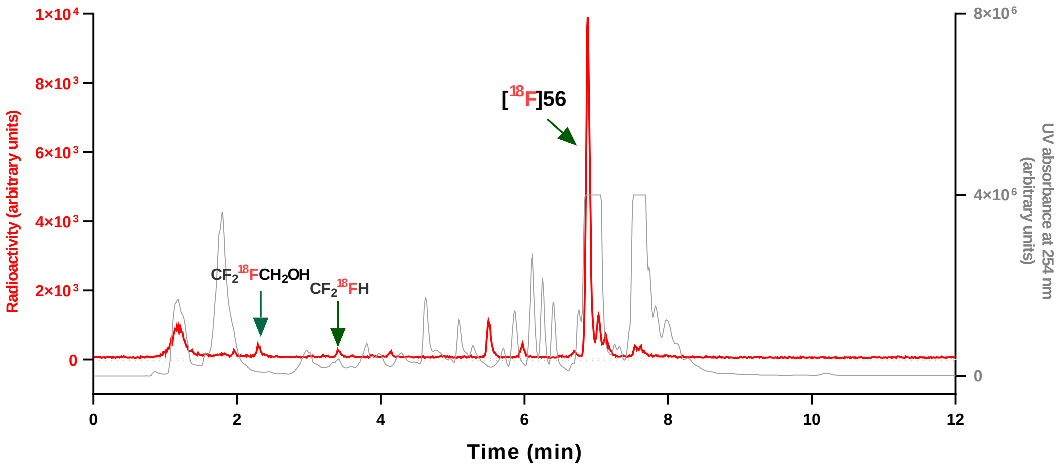

Supplementary Figure 249. Analytical HPLC chromatogram for reaction mixture.

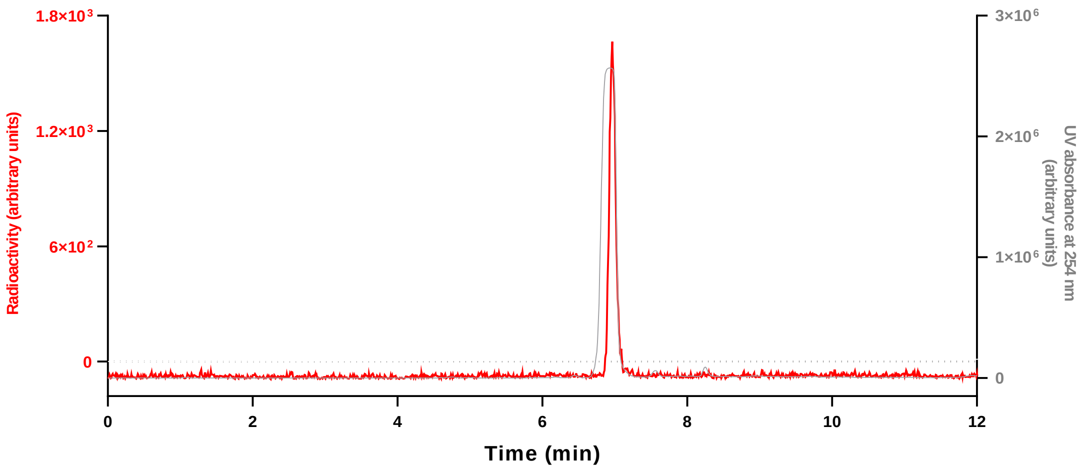

Supplementary Figure 250. Analytical HPLC chromatogram for [^{18}F]**56** collected from the above HPLC analysis when co–injected with **56.**

Run	Retention time (min)	Yield (decay-corrected) (%)	Mean yield ± SD (%)	Isolated yield (%)
1	6.883	56	56 ± 1	49
2	6.882	57		
3	6.886	56		

7.3. HPLC and LCMS analysis of PS13 analogues.

HPLC analysis of [^{11}C]57

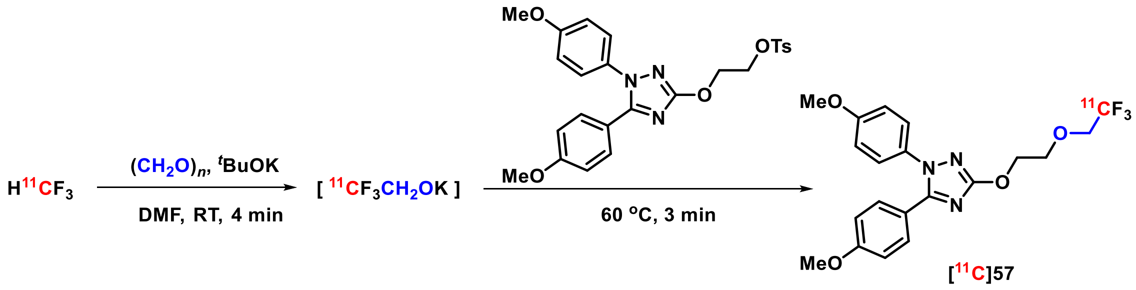

Prepared following general procedure B and analyzed using HPLC condition D.

HPLC condition D

Column: Luna C18(2) (10 μm, 250 × 4.6 mm; i.d. Phenomenex)

Solvent A: H_2O, Solvent B: MeCN

Flow rate = 2 mL/min

0–1 min: 30% B

1–5 min: 30% to 50% B

5–10 min: 90% B

10–15 min: 90% B

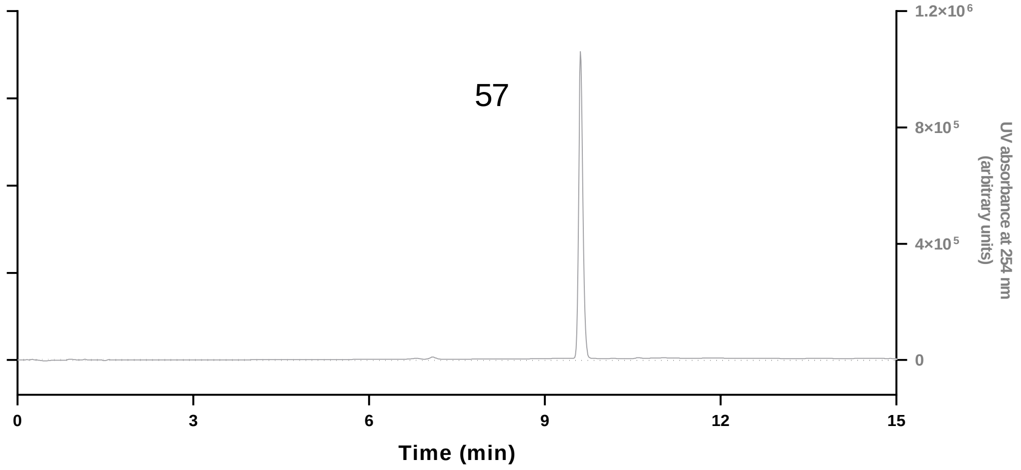

Supplementary Figure 251. Analytical HPLC chromatogram for **57.**

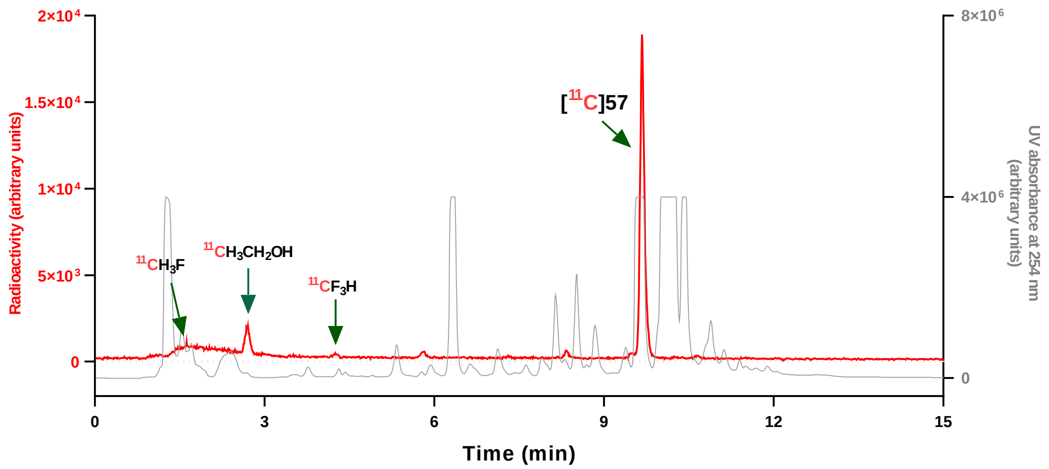

Supplementary Figure 252. Analytical HPLC chromatogram for reaction mixture.

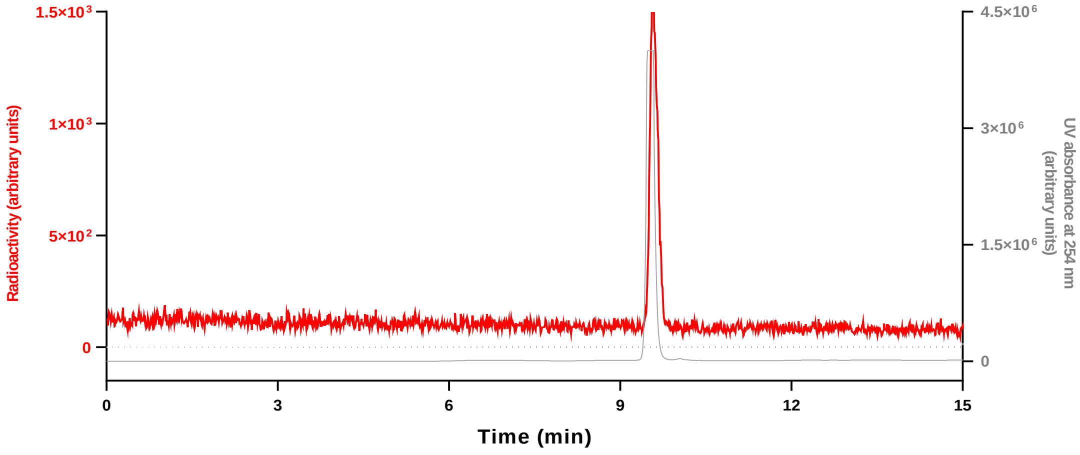

Supplementary Figure 253. Analytical HPLC chromatogram for [^{11}C]**57** collected from above HPLC analysis when co–injected with **57.**

Run	Retention time (min)	Yield (decay-corrected) (%)	Mean yield ± SD (%)	Isolated yield (%)
1	9.676	87	88 ± 2	74
2	9.628	87		
3	9.742	90		

HPLC analysis of [^{18}F]57

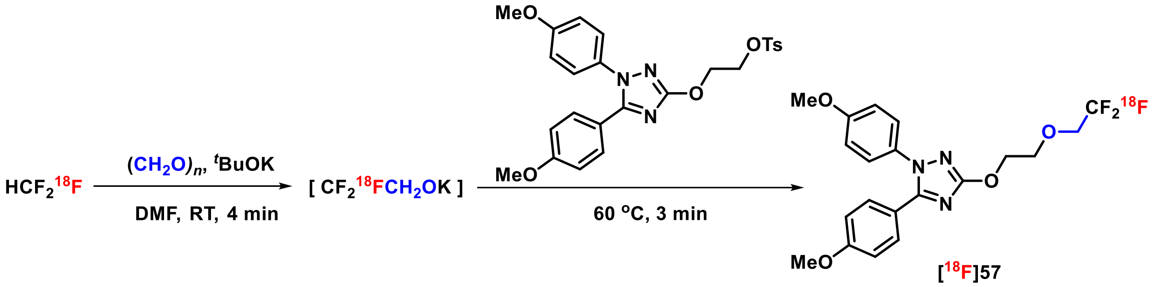

Prepared following general procedure B and analyzed using HPLC condition D.

HPLC condition D

Column: Luna C18(2) (10 μm, 250 × 4.6 mm; i.d. Phenomenex)

Solvent A: H_2O, Solvent B: MeCN

Flow rate = 2 mL/min

0–1 min: 30% B

1–5 min: 30% to 50% B

5–10 min: 90% B

10–15 min: 90% B

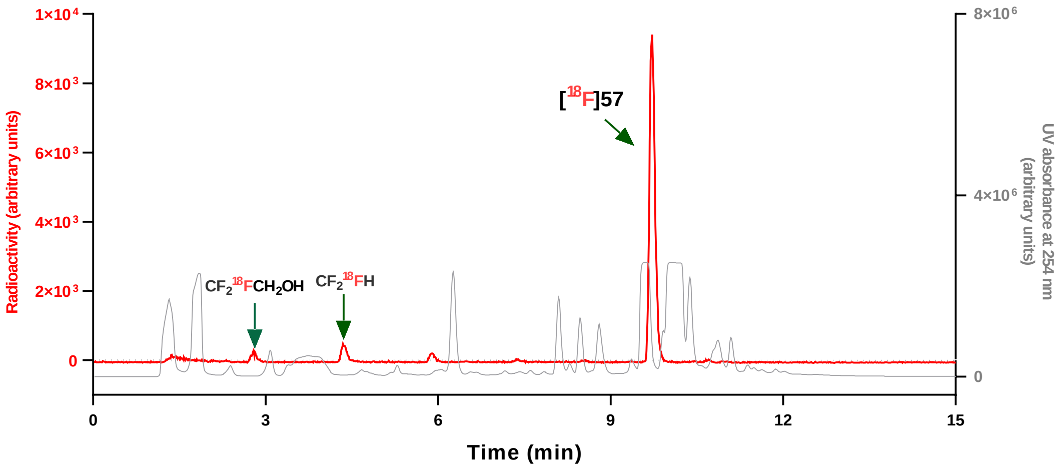

Supplementary Figure 254. Analytical HPLC chromatogram for reaction mixture.

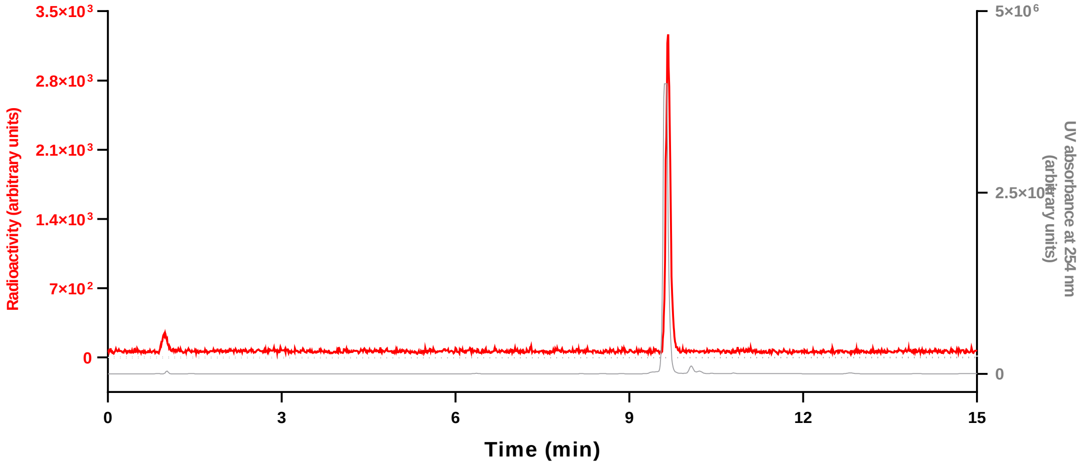

Supplementary Figure 255. Analytical HPLC chromatogram for [^{18}F]**57** collected from HPLC analysis when co–injected with **57.**

Run	Retention time (min)	Yield (decay-corrected) (%)	Mean yield ± SD (%)	Isolated yield (%)
1	9.722	86	84 ± 2	70
2	9.709	85		
3	9.696	82		

HPLC analysis of [$^2H/^{11}C$]57

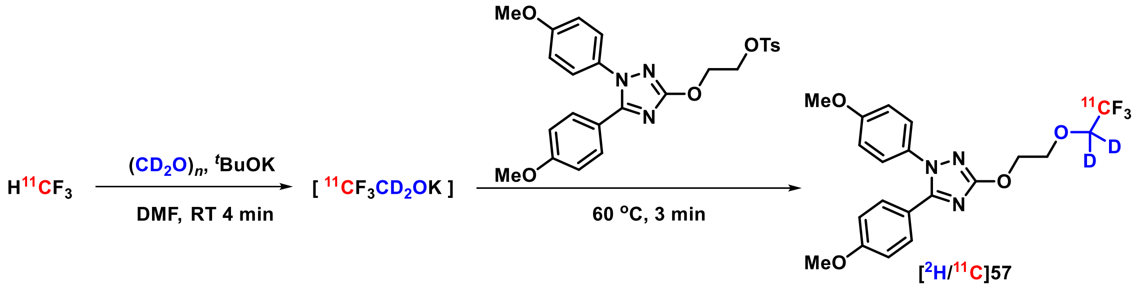

Prepared following general procedure B and analyzed using HPLC condition D.

HPLC condition D

Column: Luna C18(2) (10 μm, 250 × 4.6 mm; i.d. Phenomenex)

Solvent A: H_2O, Solvent B: MeCN

Flow rate = 2 mL/min

0–1 min: 30% B

1–5 min: 30% to 50% B

5–10 min: 90% B

10–15 min: 90% B

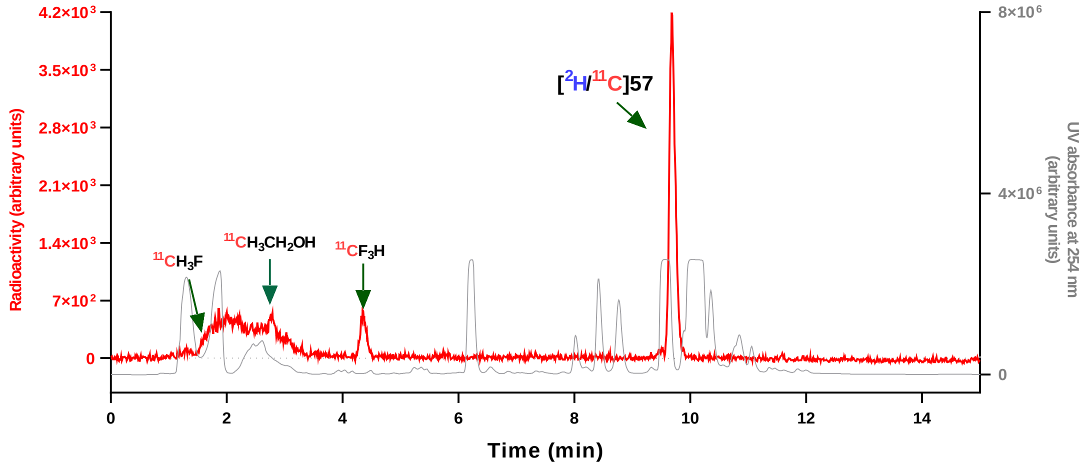

Supplementary Figure 256. Analytical HPLC chromatogram for reaction mixture.

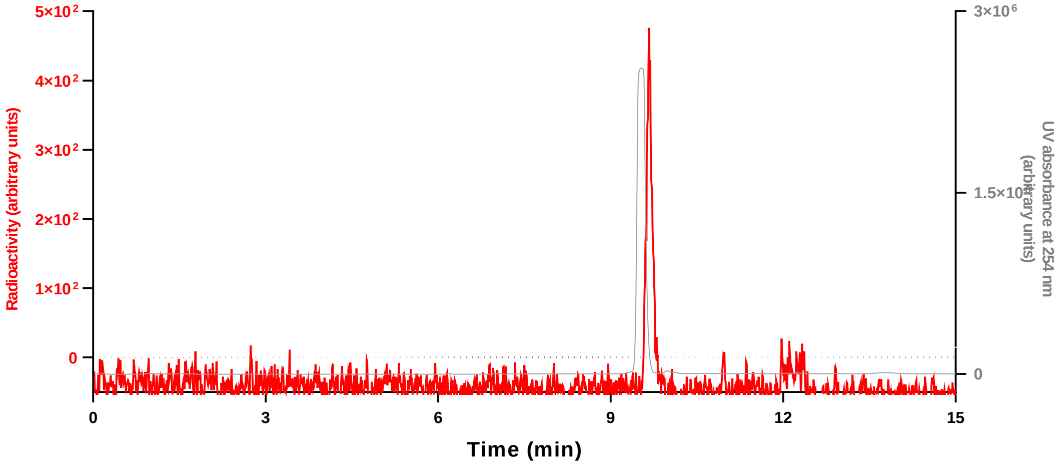

Supplementary Figure 257. Analytical HPLC chromatogram for [^{2}H/^{11}C]**57** collected from the above HPLC analysis when co-injected with **57.**

Run	Retention time (min)	Yield (decay-corrected) (%)	Mean yield ± SD (%)	Isolated yield (%)
1	9.680	83	82 ± 2	79
2	9.687	82		
3	9.698	80		

HPLC analysis of [$^2H/^{18}F$]57

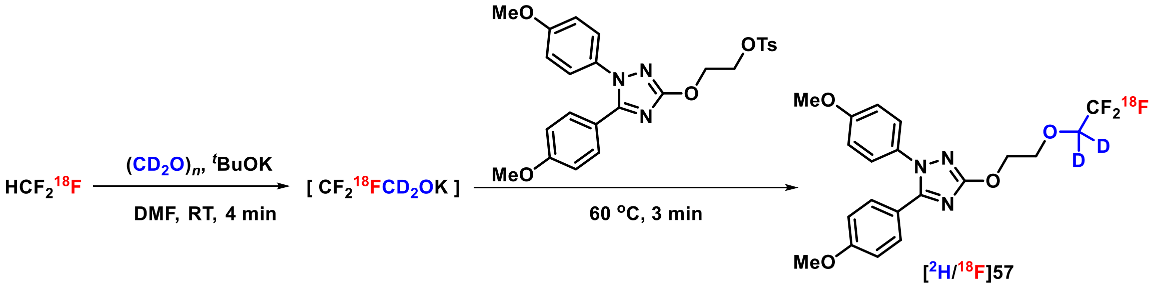

Prepared following general procedure B and analyzed using HPLC condition D.

HPLC condition D

Column: Luna C18(2) (10 μm, 250 × 4.6 mm; i.d. Phenomenex)

Solvent A: H_2O, Solvent B: MeCN

Flow rate = 2 mL/min

0–1 min: 30% B

1–5 min: 30% to 50% B

5–10 min: 90% B

10–15 min: 90% B

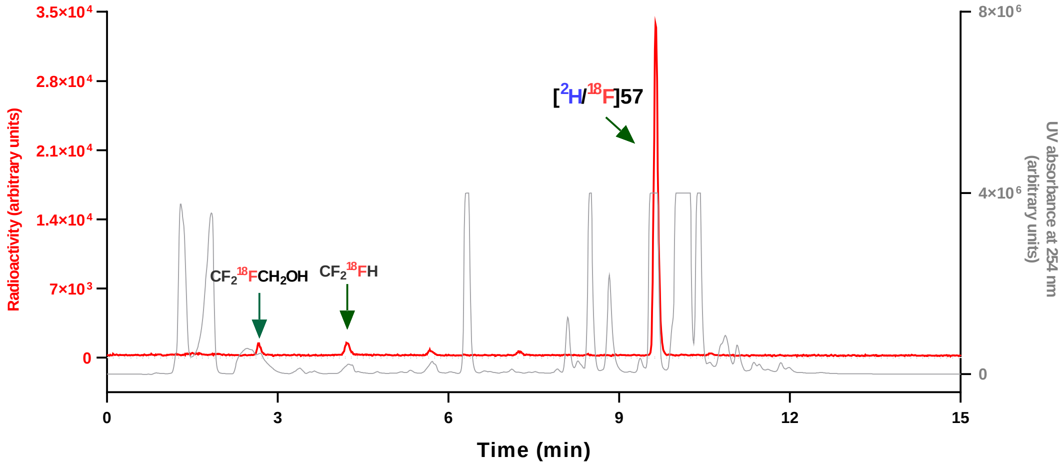

Supplementary Figure 258. Analytical HPLC chromatogram for reaction mixture.

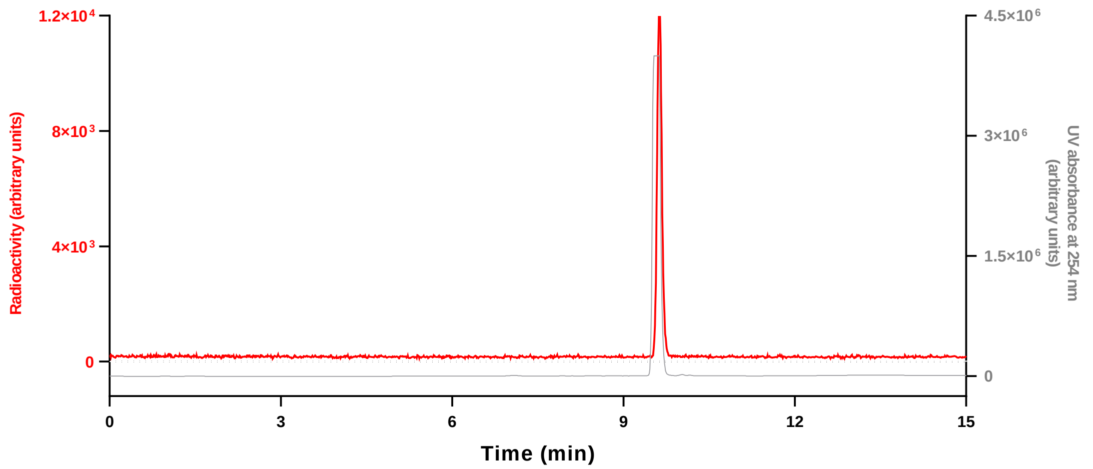

Supplementary Figure 259. Analytical HPLC chromatogram for [^{2}H/^{18}F]**57** collected from the above HPLC analysis when co-injected with **57.**

Run	Retention time (min)	Yield (decay-corrected) (%)	Mean yield ± SD (%)	Isolated yield (%)
1	9.642	80	86 ± 5	81
2	9.645	90		
3	9.649	87		

HPLC analysis of [$^{13}C/^{11}C$]57

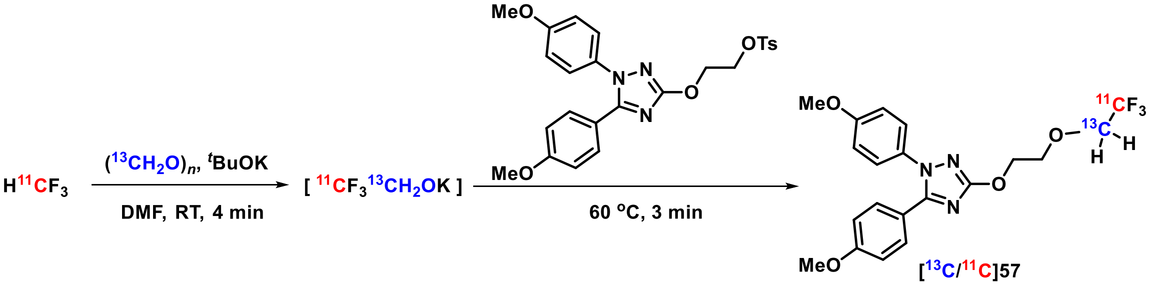

Prepared following general procedure B and analyzed using HPLC condition D.

HPLC condition D

Column: Luna C18(2) (10 μm, 250 × 4.6 mm; i.d. Phenomenex)

Solvent A: H_2O, Solvent B: MeCN

Flow rate = 2 mL/min

0–1 min: 30% B

1–5 min: 30% to 50% B

5–10 min: 90% B

10–15 min: 90% B

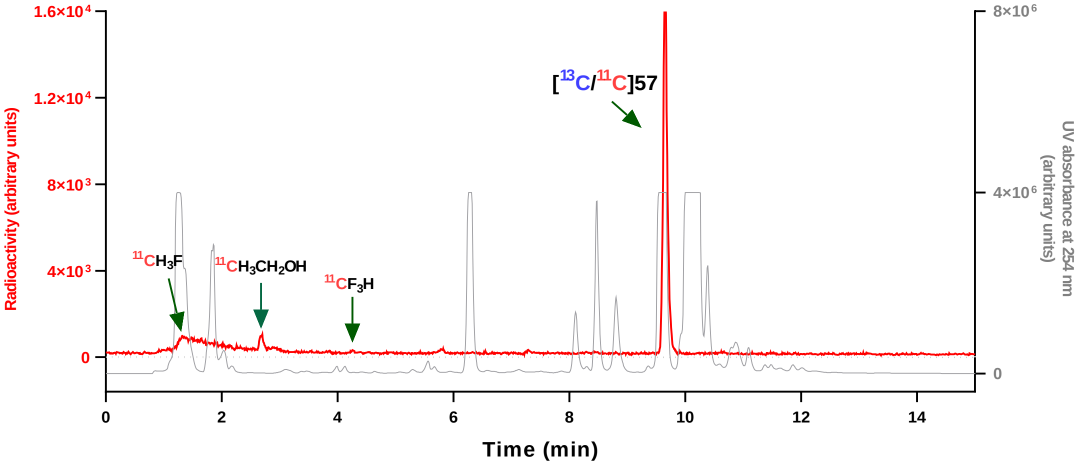

Supplementary Figure 260. Analytical HPLC chromatogram for reaction mixture.

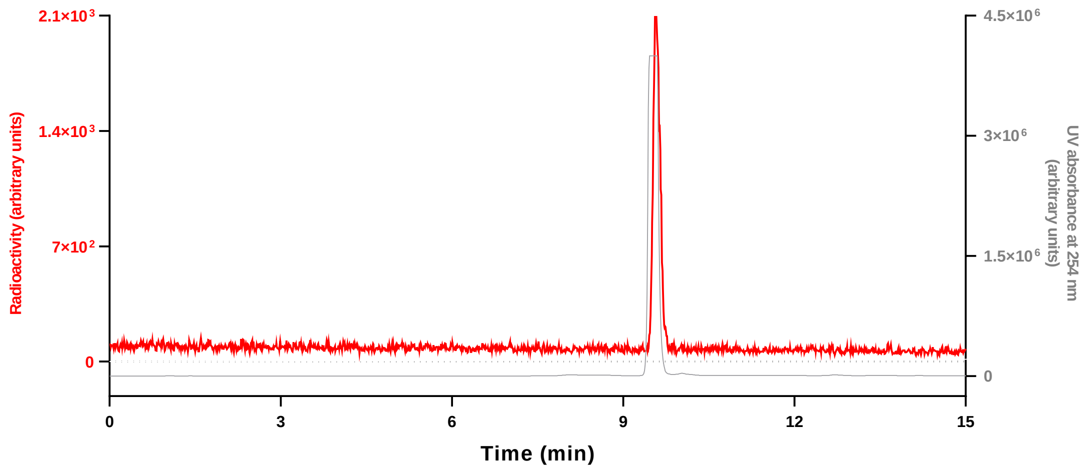

Supplementary Figure 261. Analytical HPLC chromatogram for [^{13}C/^{11}C]**57** collected from the HPLC analysis when co-injected with **57.**

Run	Retention time (min)	Yield (decay-corrected) (%)	Mean yield ± SD (%)	Isolated yield (%)
1	9.653	93	92 ± 1	81
2	9.652	92		
3	9.667	92		

HPLC analysis of [^{13}C/^{18}F]57

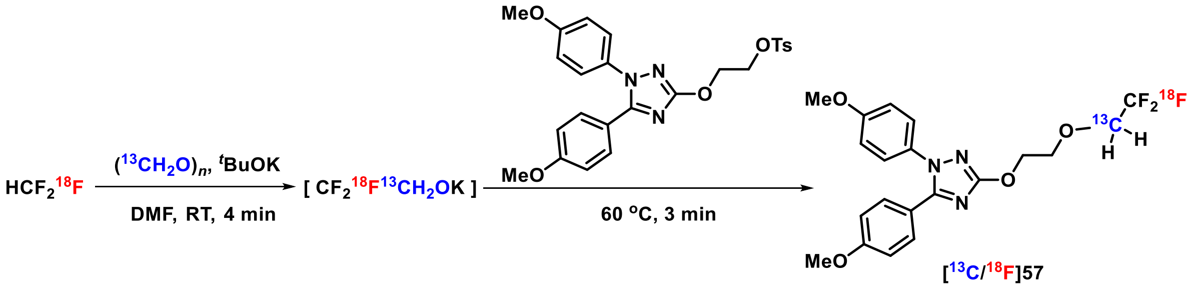

Prepared following general procedure B and analyzed using HPLC condition D.

HPLC condition D

Column: Luna C18(2) (10 μm, 250 × 4.6 mm; i.d. Phenomenex)

Solvent A: H_2O, Solvent B: MeCN

Flow rate = 2 mL/min

0–1 min: 30% B

1–5 min: 30% to 50% B

5–10 min: 90% B

10–15 min: 90% B

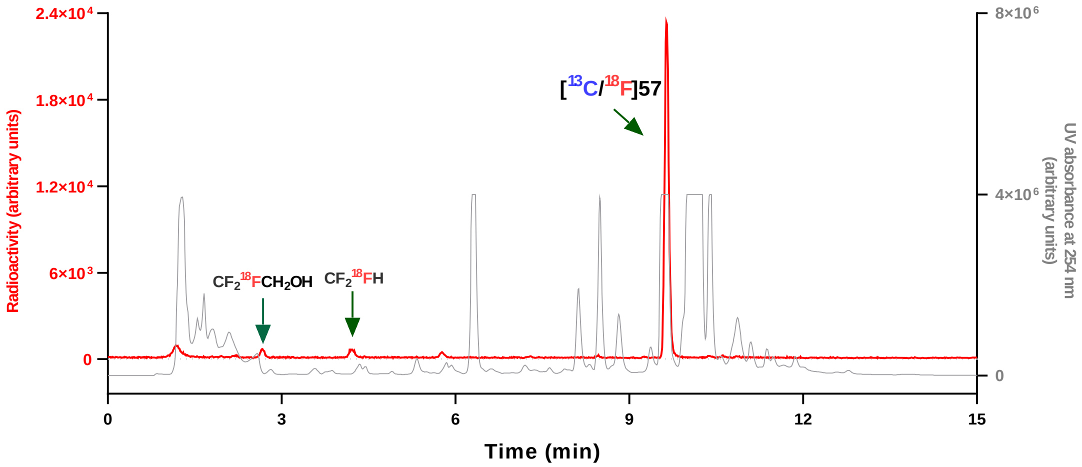

Supplementary Figure 262. Analytical HPLC chromatogram for reaction mixture.

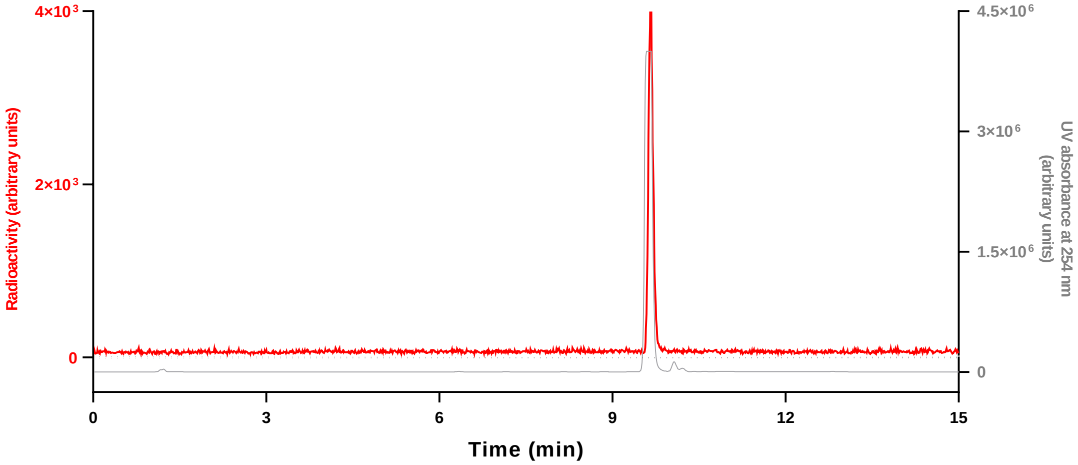

Supplementary Figure 263. Analytical HPLC chromatogram for [^{13}C/^{18}F]**57** collected from the above HPLC analysis when co–injected with **57.**

Run	Retention time (min)	Yield (decay-corrected) (%)	Mean yield ± SD (%)	Isolated yield (%)
1	9.645	85	84 ± 1	73
2	9.654	83		
3	9.655	83		

Supplementary Abbreviations

DCC, *N,N*'-dicyclohexylcarbodiimide

DCM, dichloromethane

DMAP, 4-dimethylaminopyridine

DMF, dimethylformamide

DMSO, dimethyl sulfoxide

ESI, electrospray ionization

HPLC, high performance liquid chromatography

K 2.2.2, 2.2.2. cryptand (4,7,13,16,21,24-hexaoxa-1,10-diazabicyclo[8.8.8]hexacosane)

*m*CPBA, *meta* chloroperbenzoic acid

Meldrum's acid, 2,2-dimethyl-1,3-dioxane-4,6-dione

Mesyl, mesityl (2,4,6-trimethylphenyl)

NIDDK, National Institute of Diabetes, Digestive and Kidney Diseases

NIMH, National Institute of Mental Health

RT, room temperature

THF, tetrahydrofuran

Supplementary NMR spectra

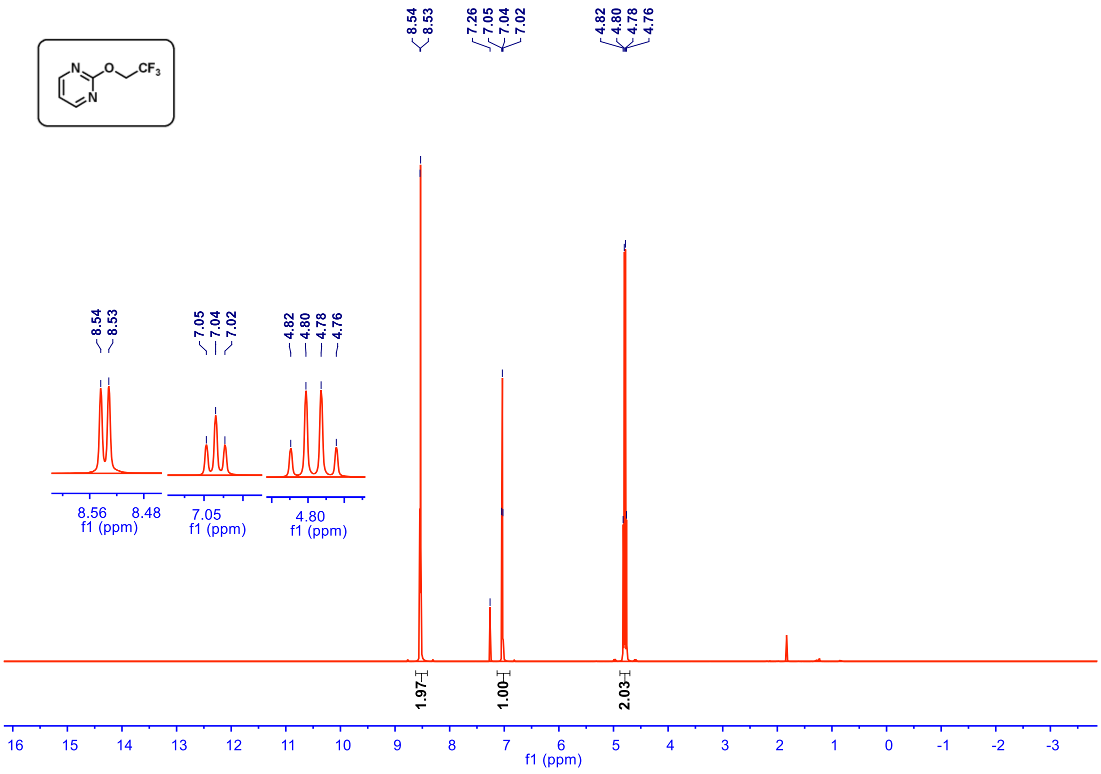

Supplementary Figure 264. ^{1}H NMR spectrum of compound **1** (400 MHz, $CDCl_3$)

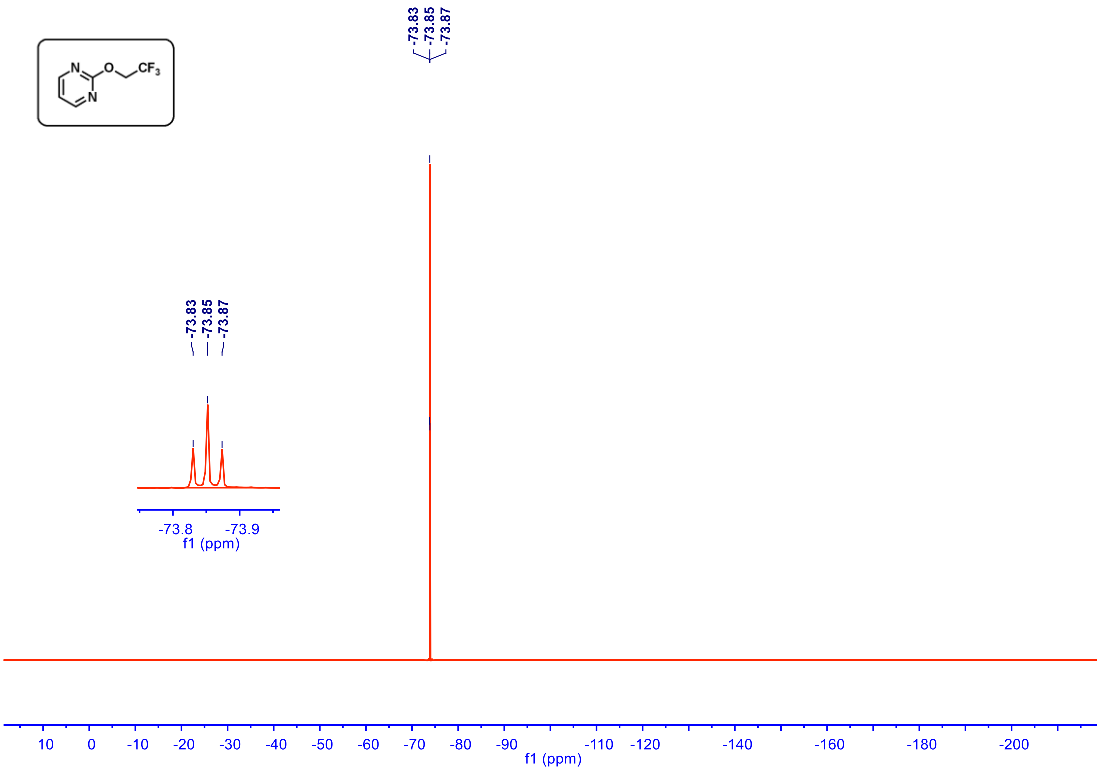

Supplementary Figure 265. ^{19}F NMR spectrum of compound **1** (376 MHz, $CDCl_3$)

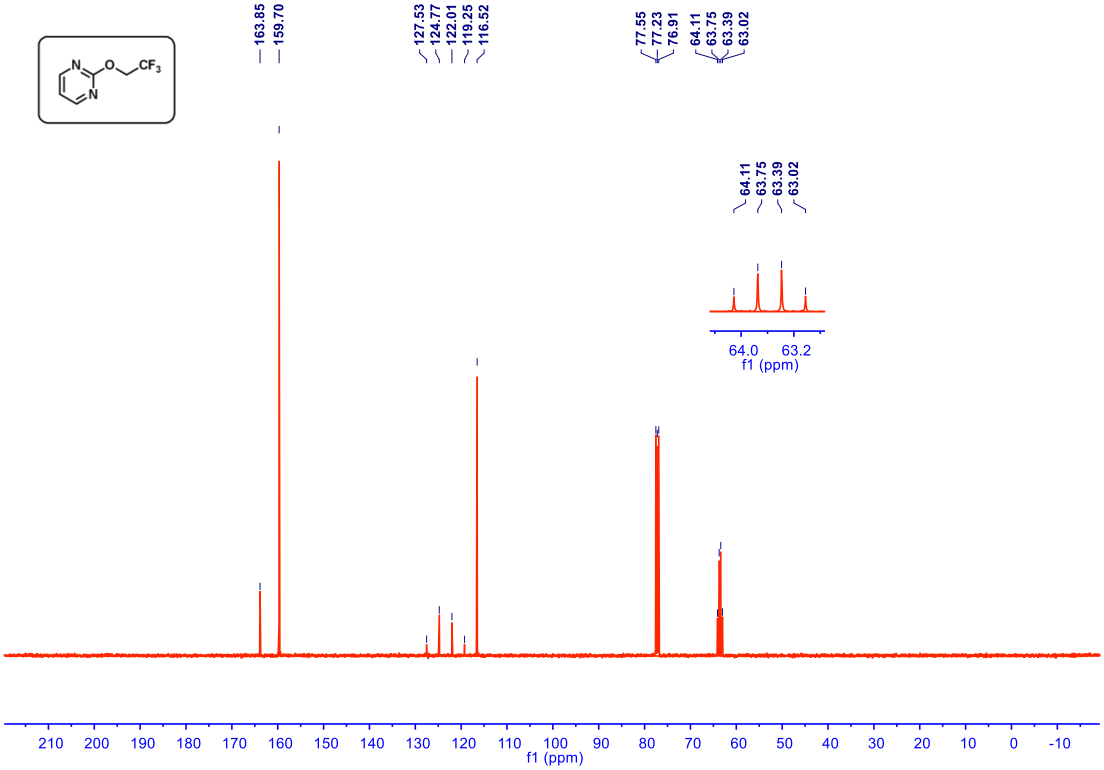

Supplementary Figure 266. ^{13}C NMR spectrum of compound **1** (101 MHz, $CDCl_3$)

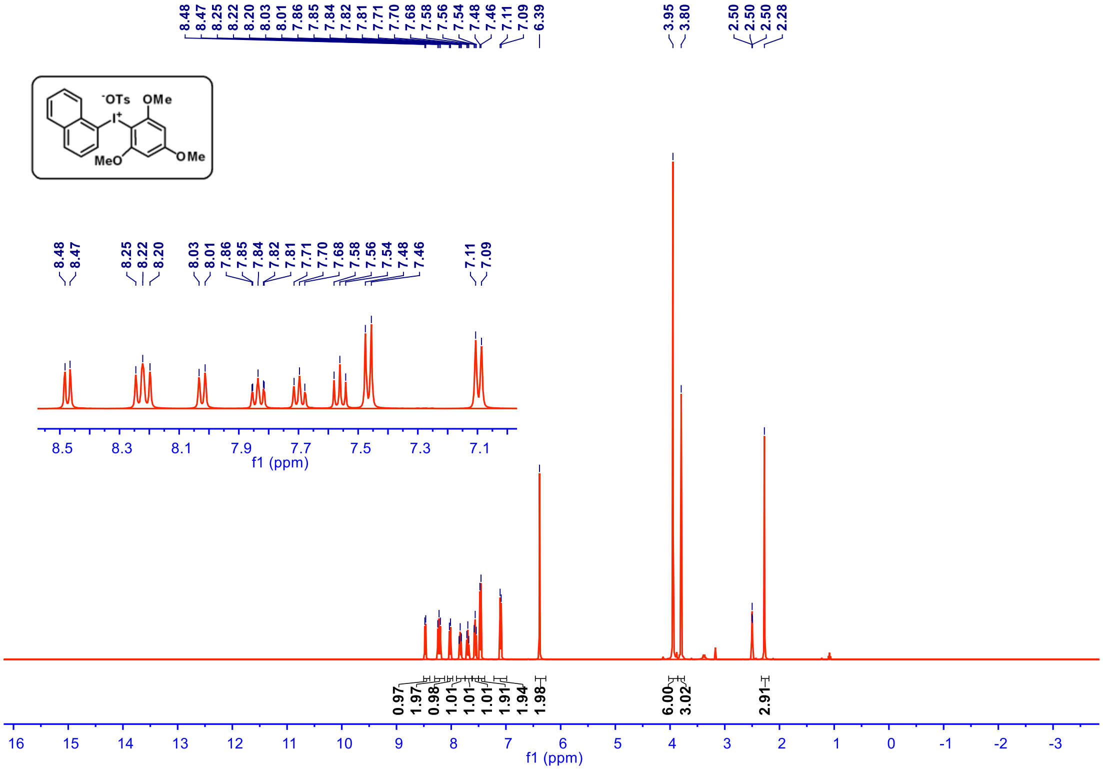

Supplementary Figure 267. ^{1}H NMR spectrum of **Naphthalen-1-yl(2,4,6-trimethoxyphenyl)iodonium tosylate** (400 MHz, $CDCl_3$)

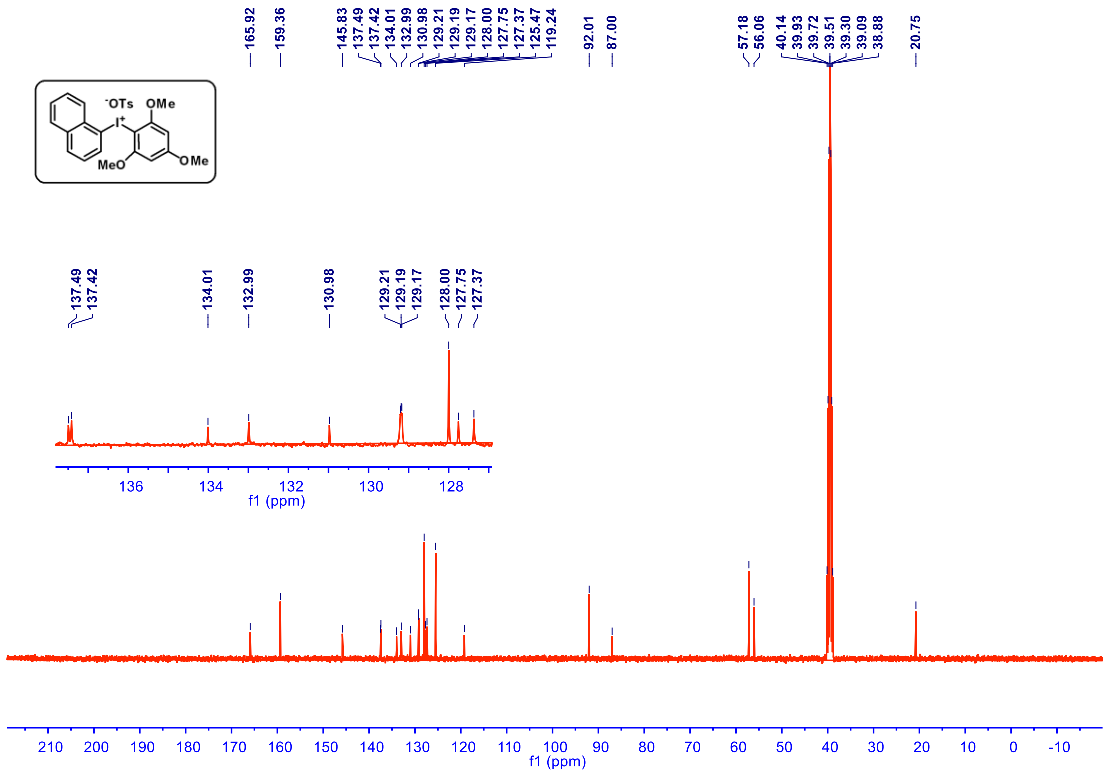

Supplementary Figure 268. ^{13}C NMR spectrum of **Naphthalen-1-yl(2,4,6-trimethoxyphenyl)iodonium tosylate** (101 MHz, $CDCl_3$)

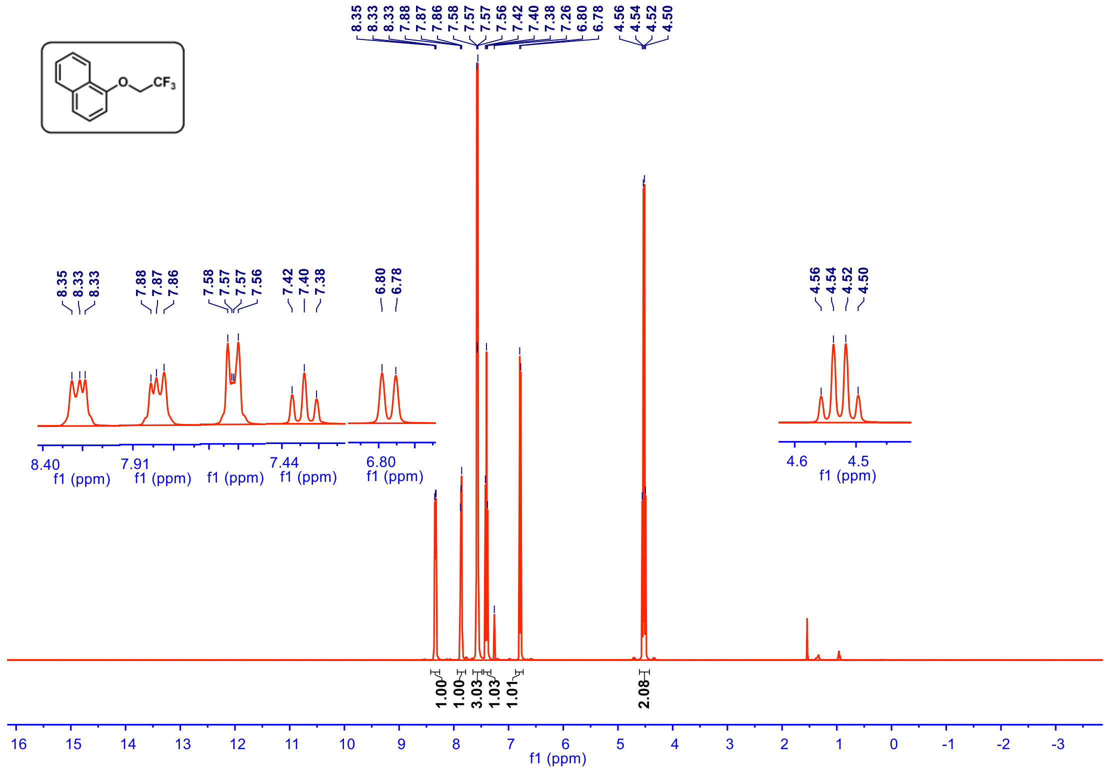

Supplementary Figure 269. ^{1}H NMR spectrum of compound **2** (400 MHz, $CDCl_3$)

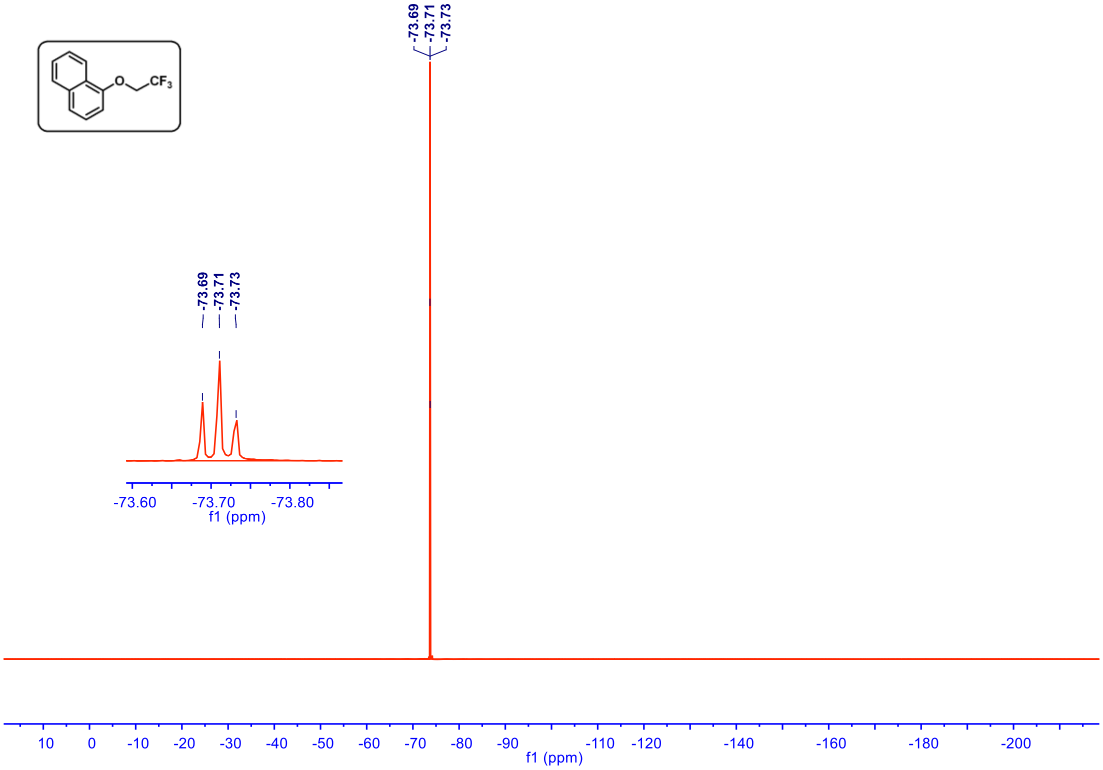

Supplementary Figure 270. ^{19}F NMR spectrum of compound **2** (376 MHz, $CDCl_3$)

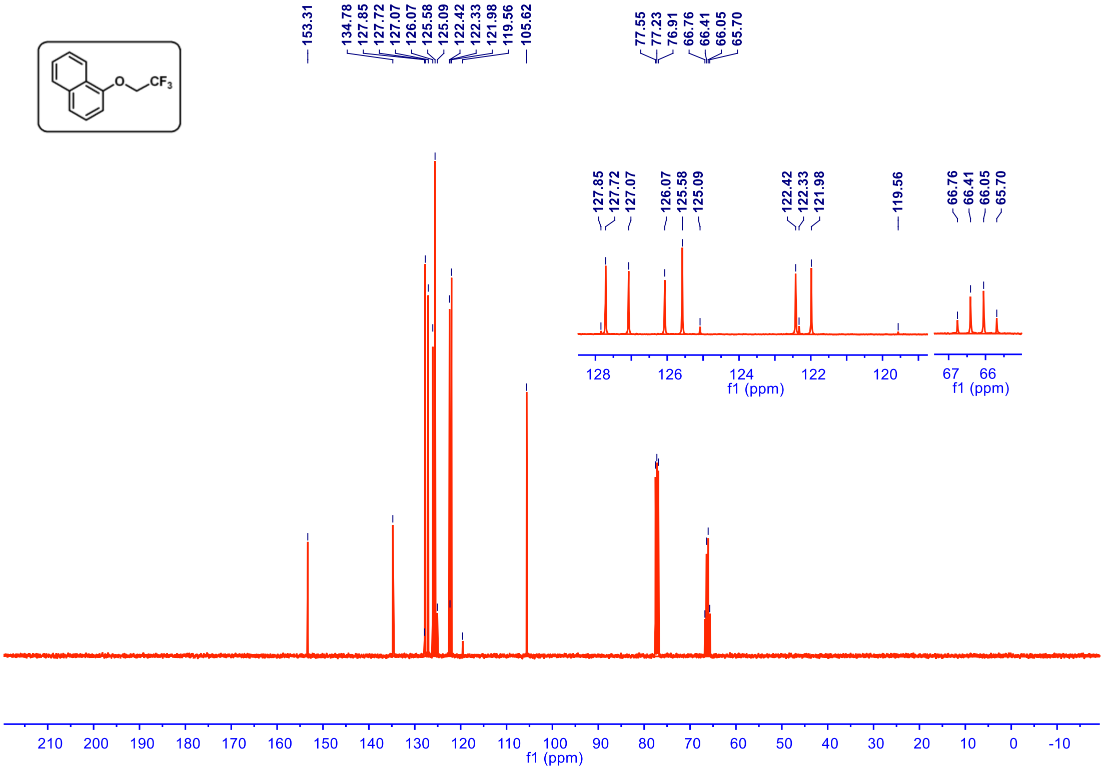

Supplementary Figure 271. ^{13}C NMR spectrum of compound **2** (101 MHz, $CDCl_3$)

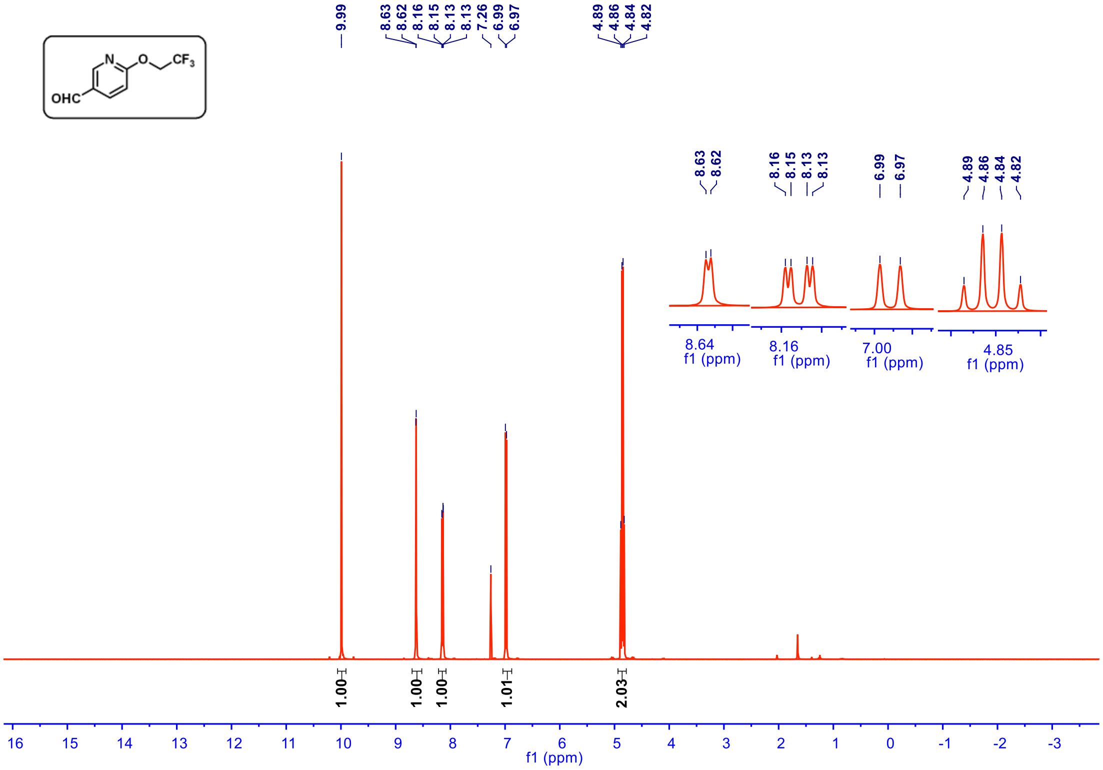

Supplementary Figure 272. ^{1}H NMR spectrum of compound **3** (400 MHz, $CDCl_3$)

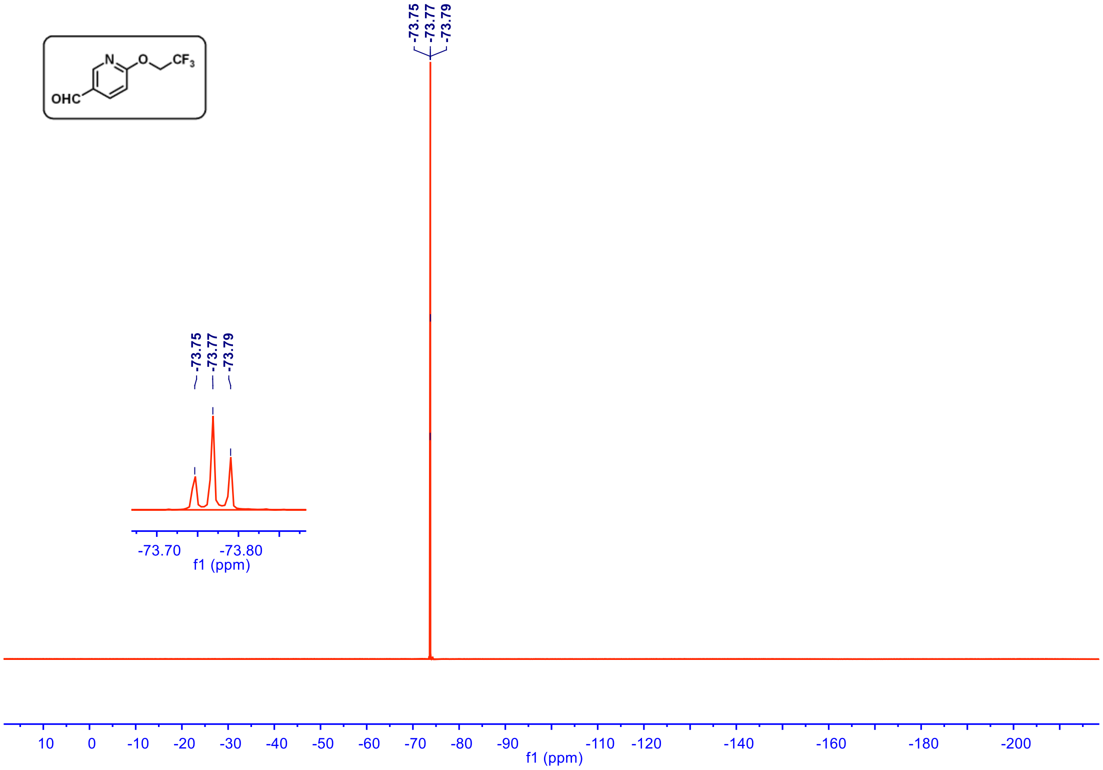

Supplementary Figure 273. ^{19}F NMR spectrum of compound **3** (376 MHz, $CDCl_3$)

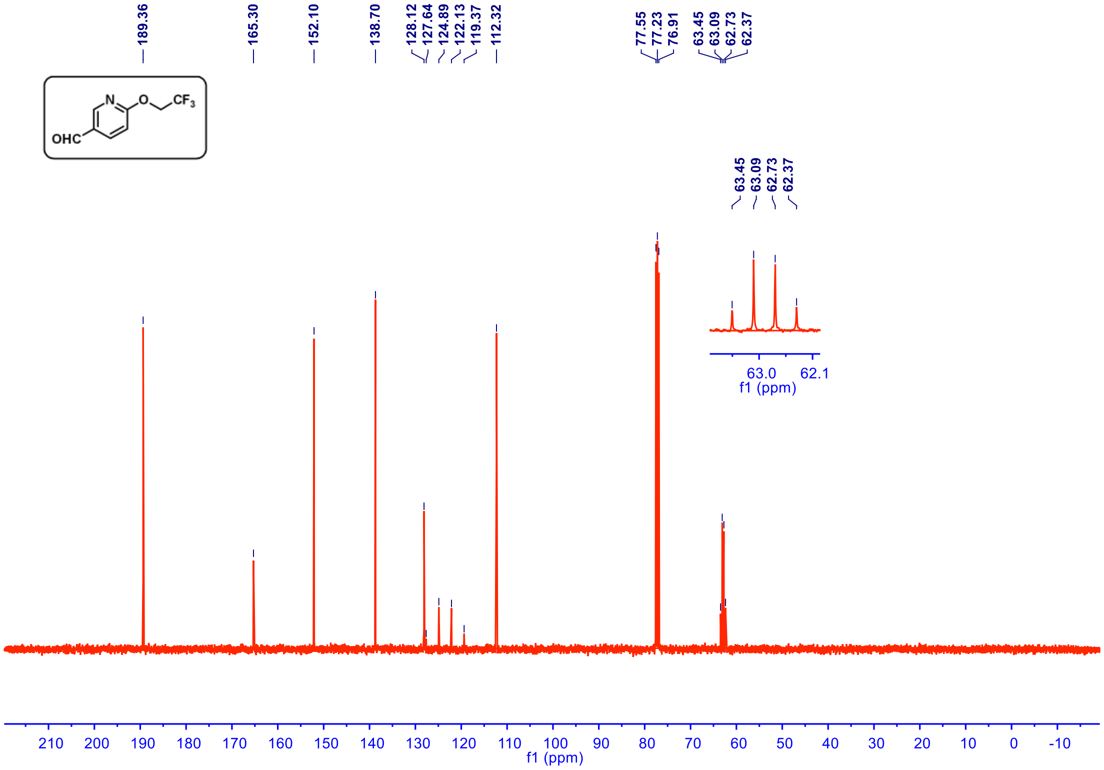

Supplementary Figure 274. ^{13}C NMR spectrum of compound **3** (101 MHz, $CDCl_3$)

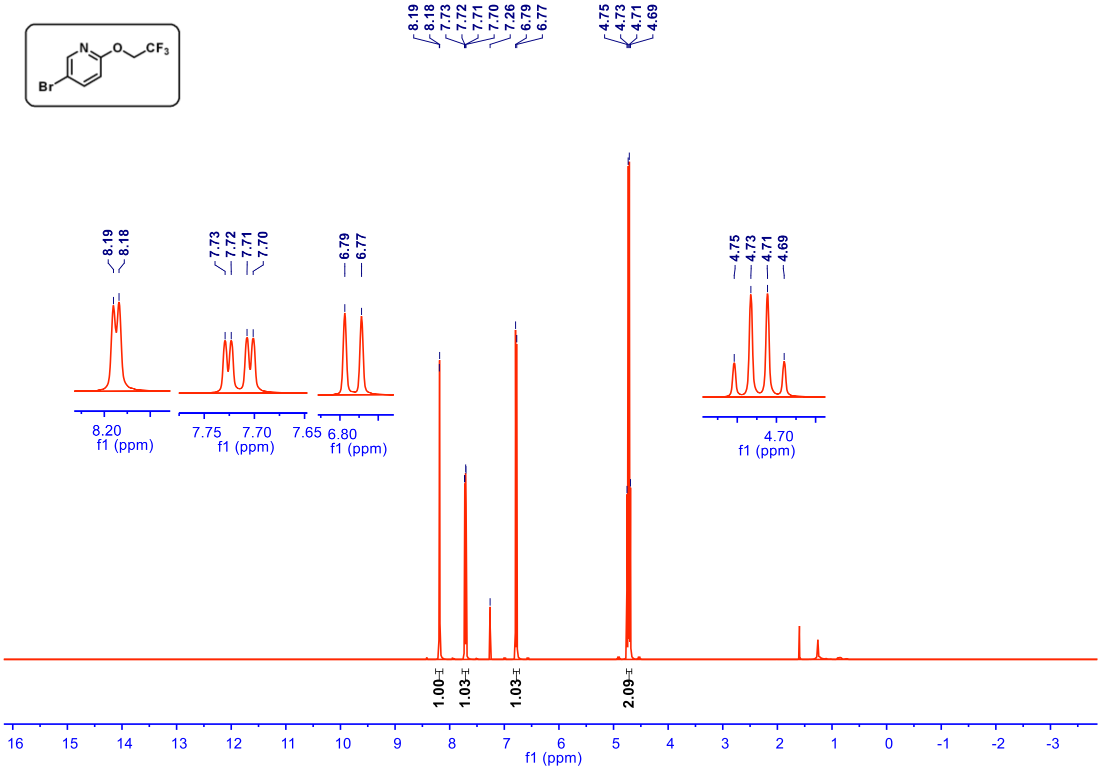

Supplementary Figure 275. ^{1}H NMR spectrum of compound **4** (400 MHz, $CDCl_3$)

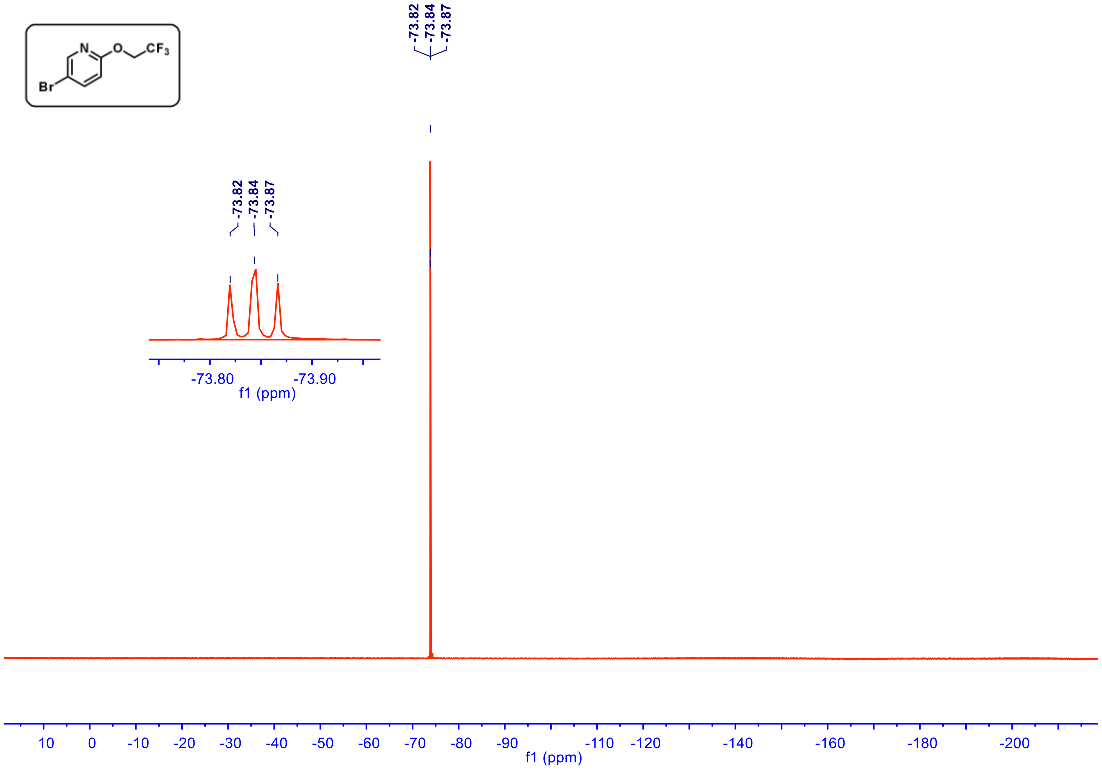

Supplementary Figure 276. ^{19}F NMR spectrum of compound **4** (376 MHz, $CDCl_3$)

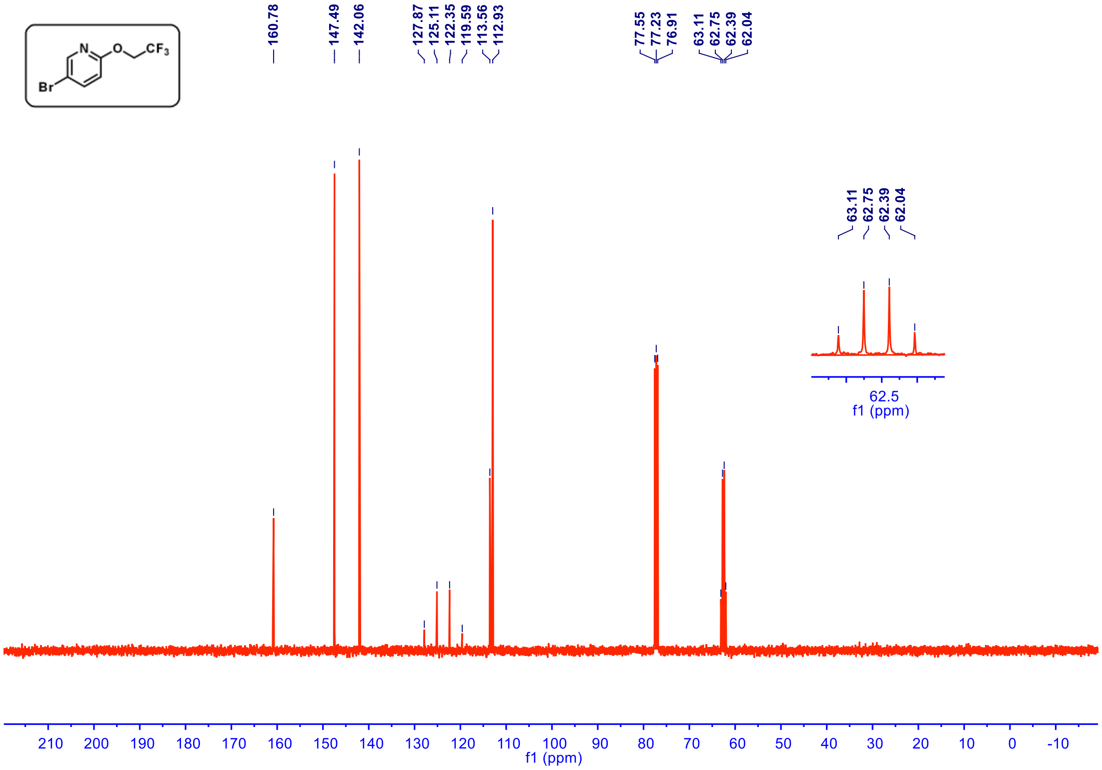

Supplementary Figure 277. ^{13}C NMR spectrum of compound **4** (101 MHz, $CDCl_3$)

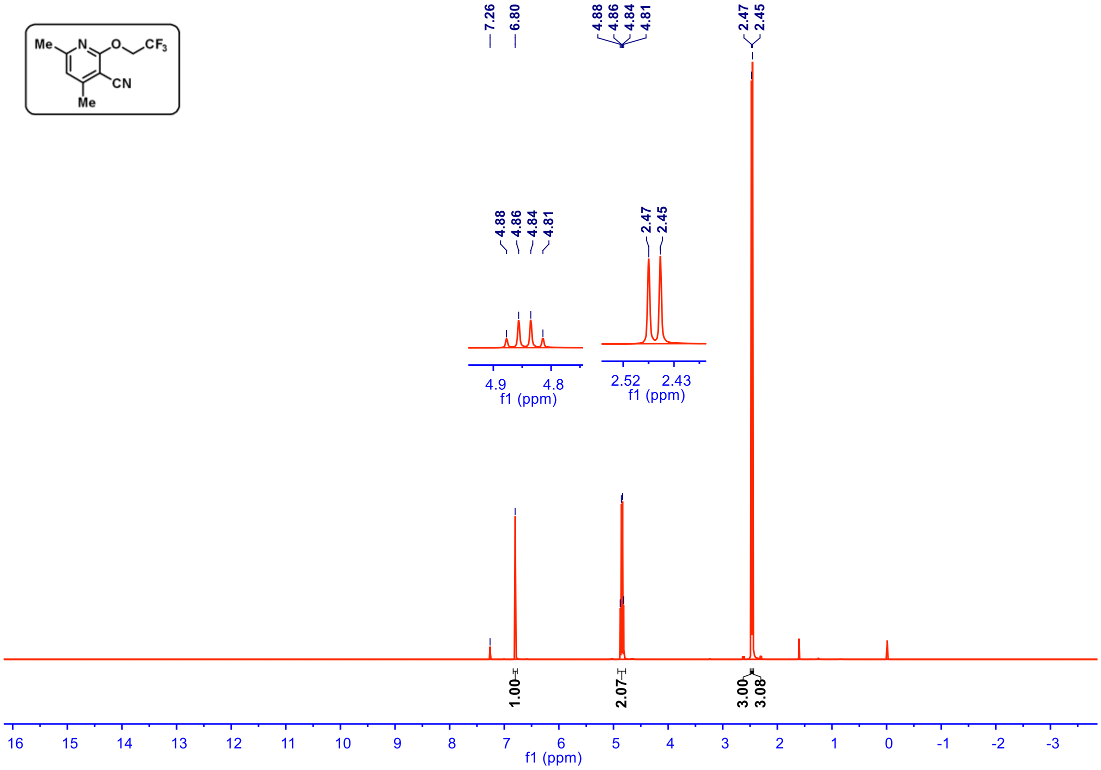

Supplementary Figure 278. ^{1}H NMR spectrum of compound **5** (400 MHz, $CDCl_3$)

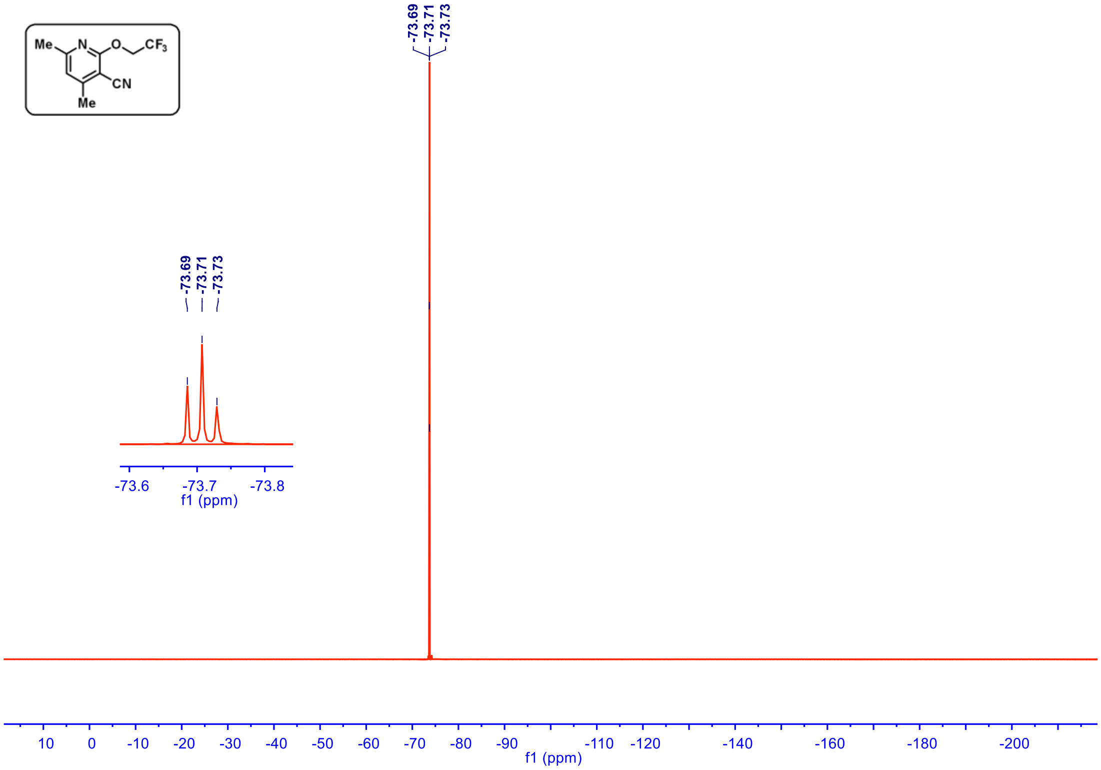

Supplementary Figure 279. ^{19}F NMR spectrum of compound **5** (376 MHz, $CDCl_3$)

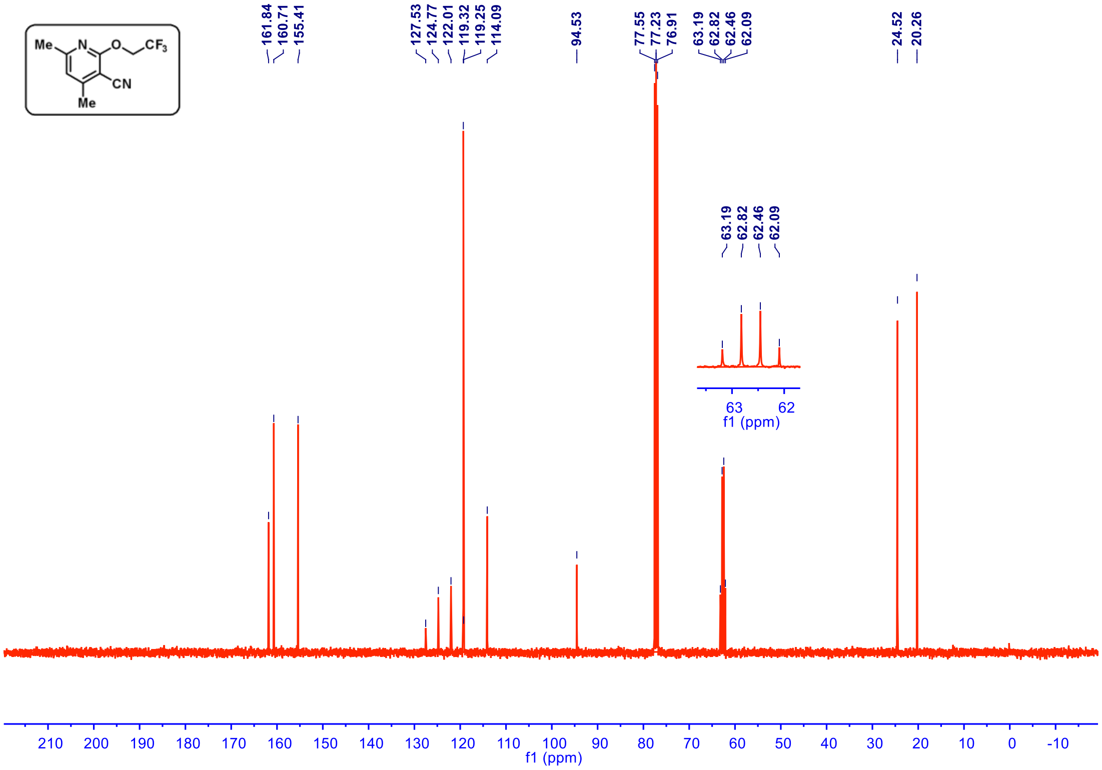

Supplementary Figure 280. ^{13}C NMR spectrum of compound **5** (101 MHz, $CDCl_3$)

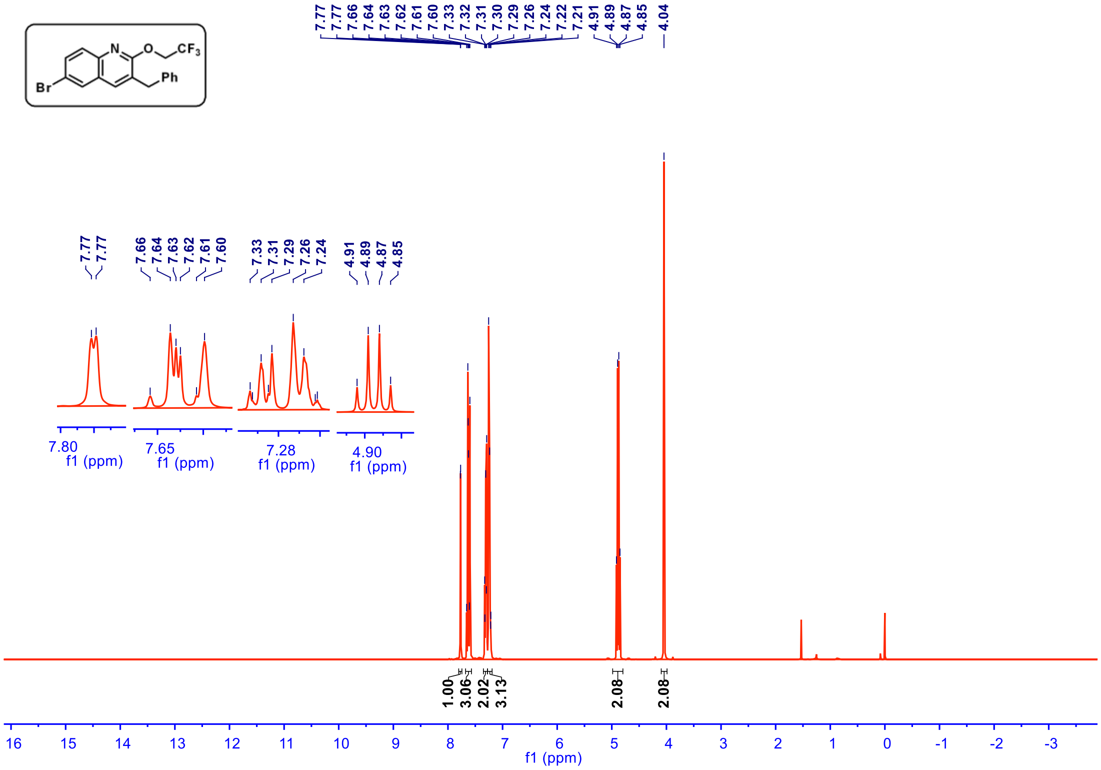

Supplementary Figure 281. ^{1}H NMR spectrum of compound **6** (400 MHz, $CDCl_3$)

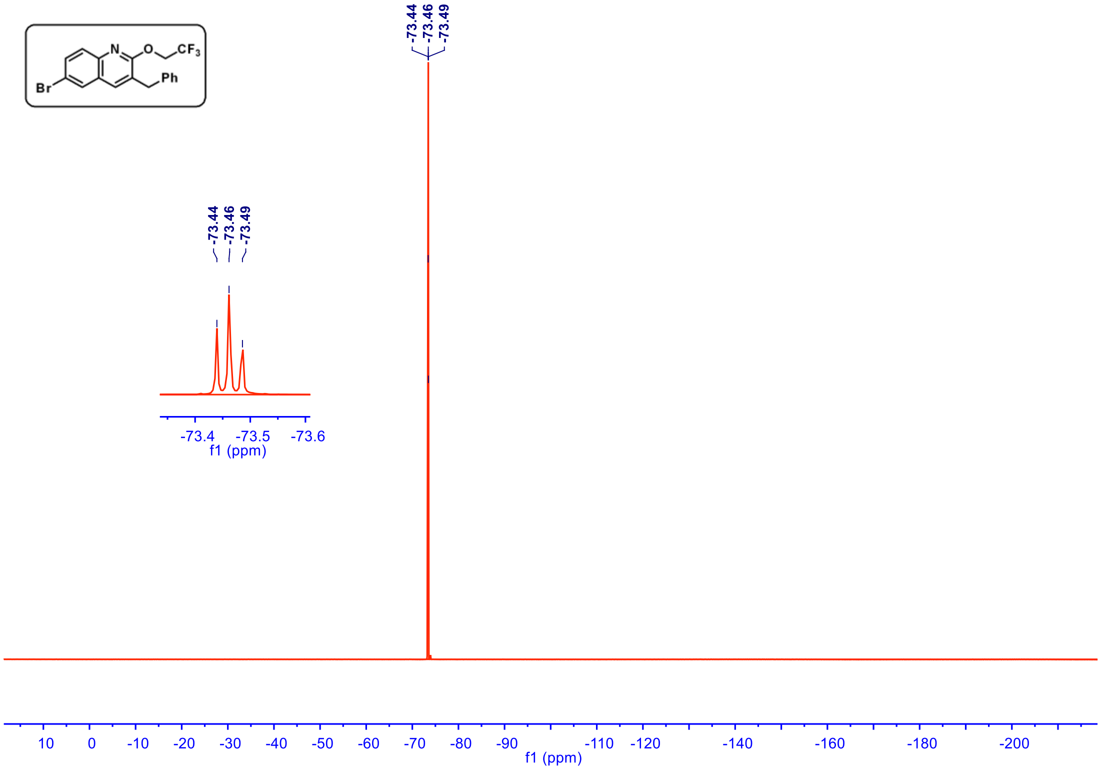

Supplementary Figure 282. ^{19}F NMR spectrum of compound **6** (376 MHz, $CDCl_3$)

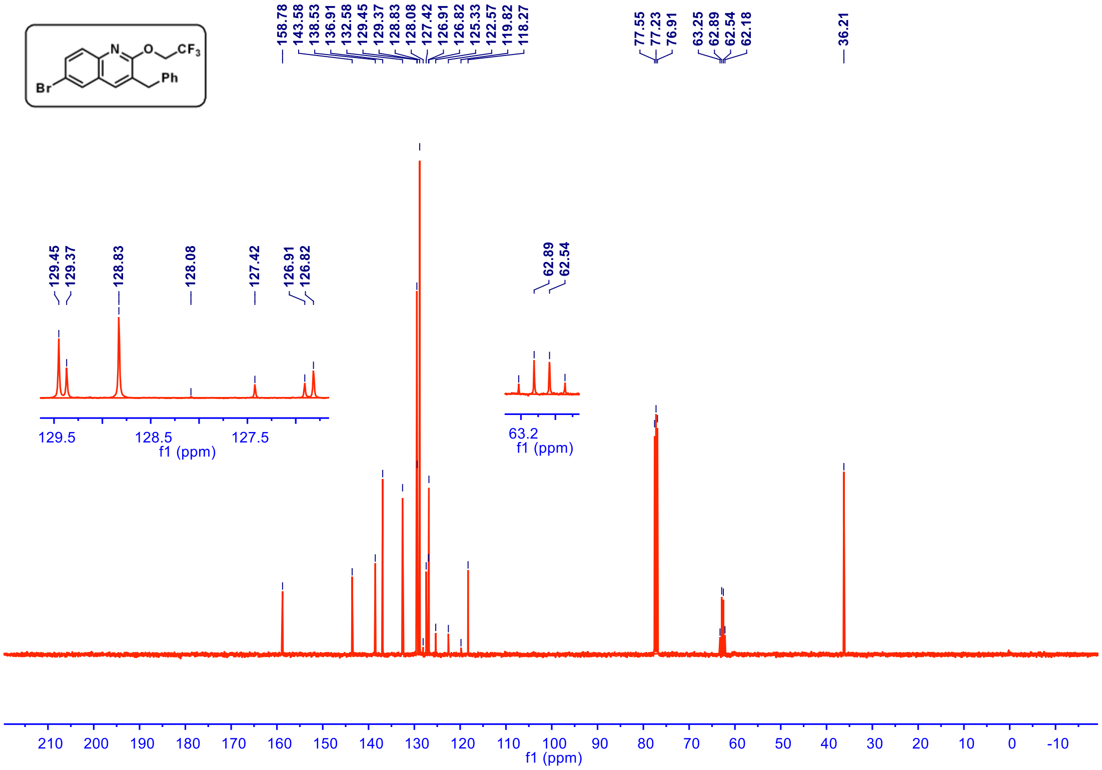

Supplementary Figure 283. ^{13}C NMR spectrum of compound **6** (101 MHz, $CDCl_3$)

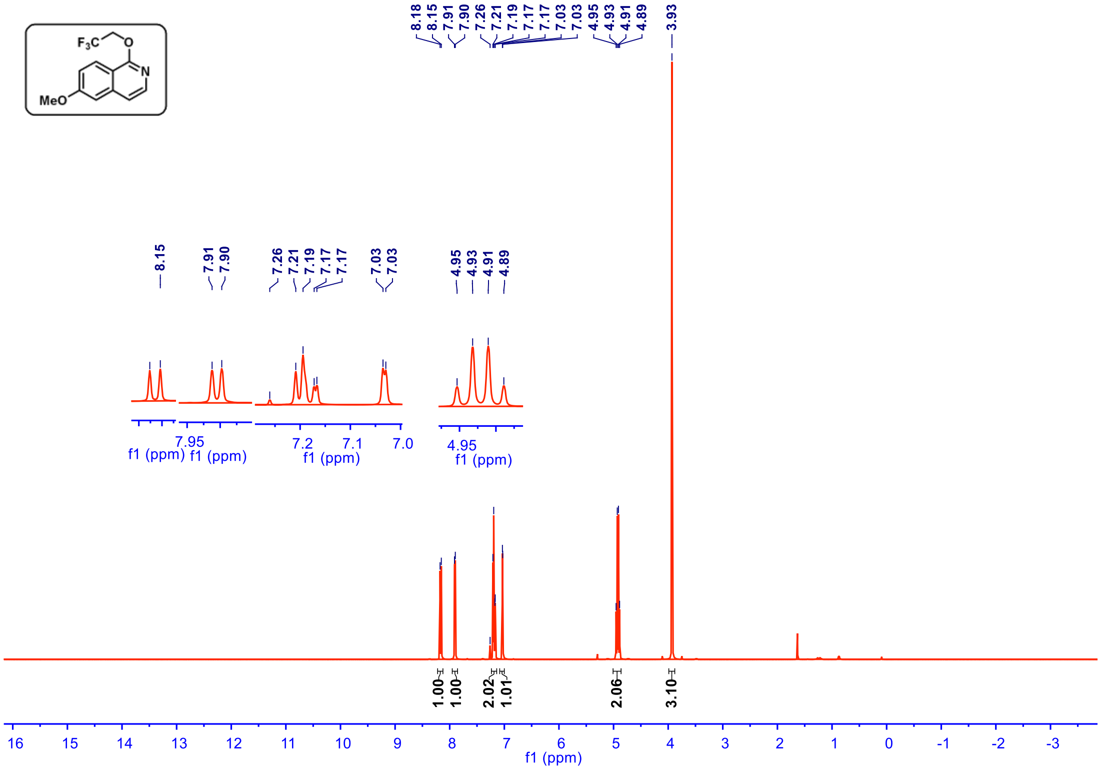

Supplementary Figure 284. ^{1}H NMR spectrum of compound **7** (400 MHz, $CDCl_3$)

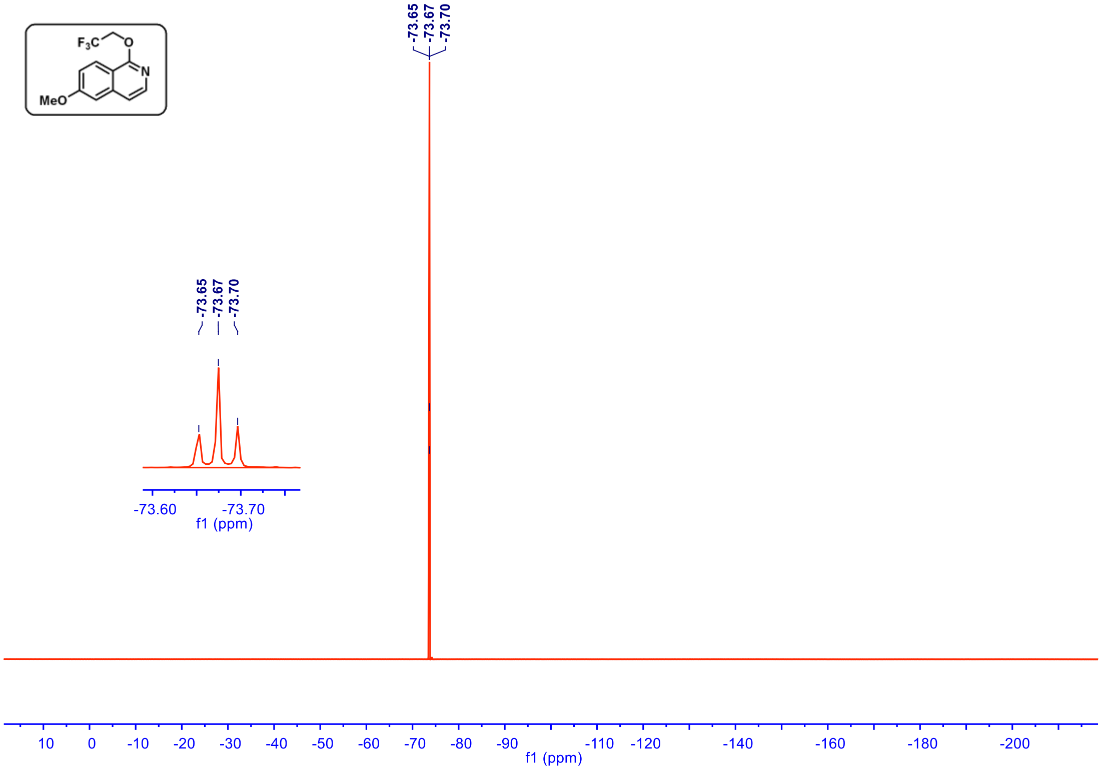

Supplementary Figure 285. ^{19}F NMR spectrum of compound **7** (376 MHz, $CDCl_3$)

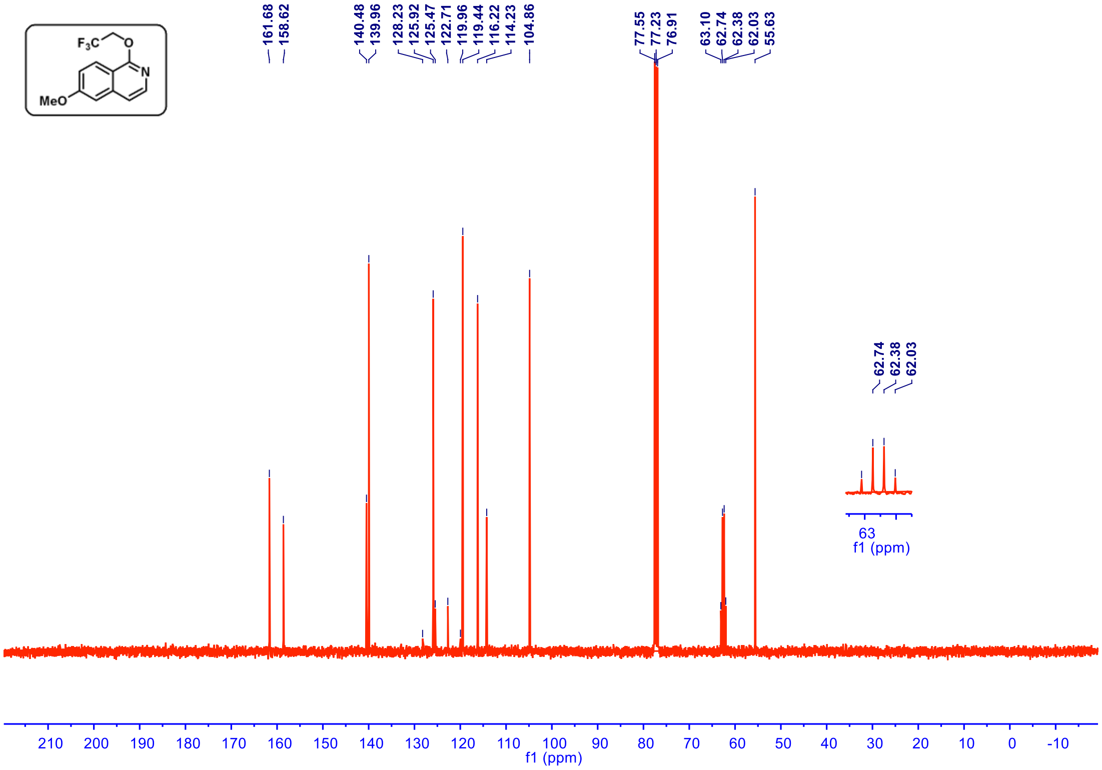

Supplementary Figure 286. ^{13}C NMR spectrum of compound **7** (101 MHz, $CDCl_3$)

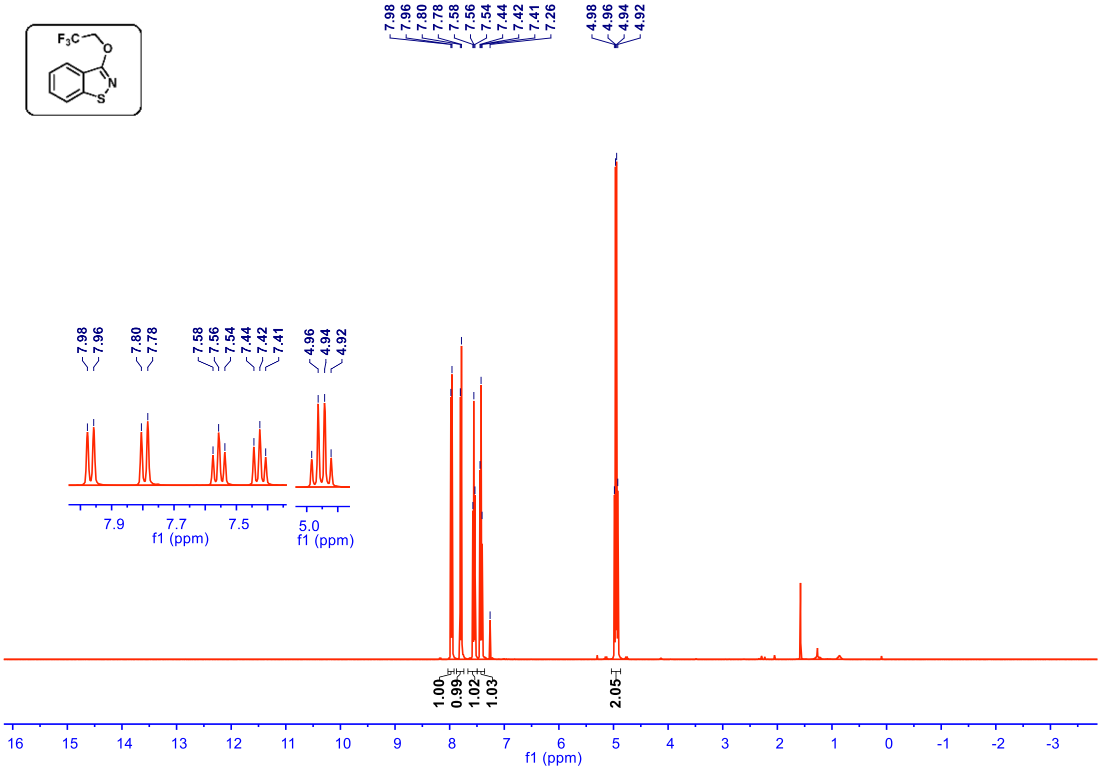

Supplementary Figure 287. ^{1}H NMR spectrum of compound **8** (400 MHz, $CDCl_3$)

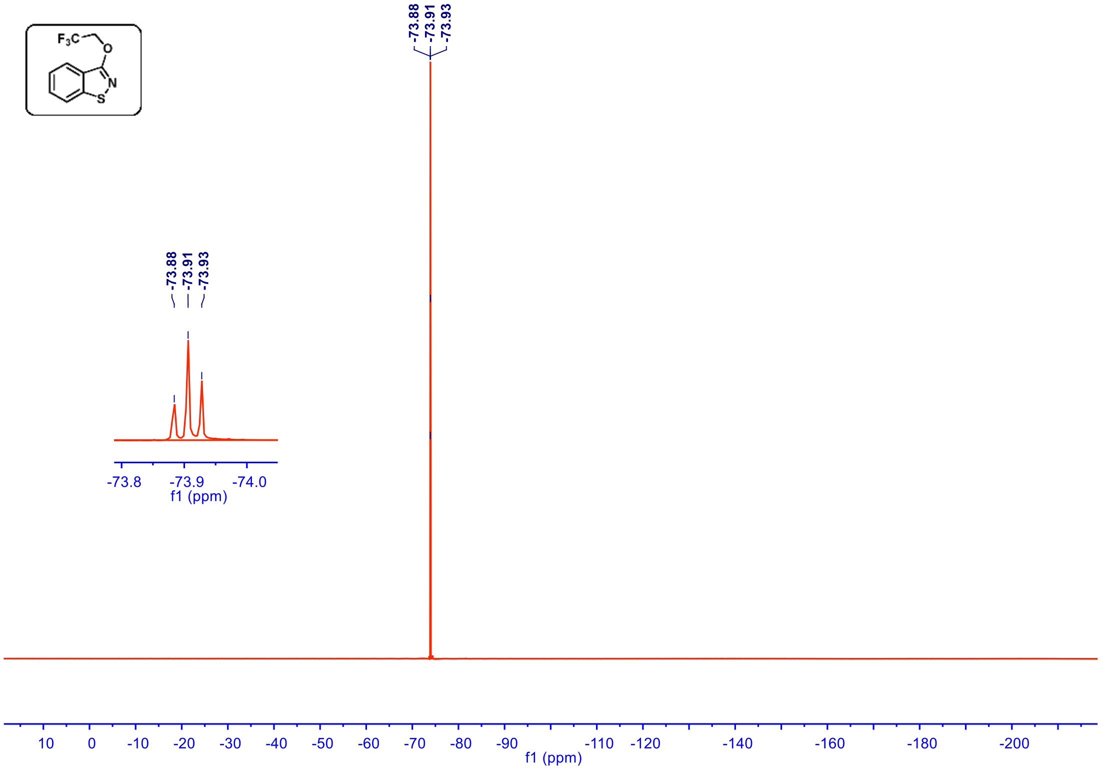

Supplementary Figure 288. ^{19}F NMR spectrum of compound **8** (376 MHz, $CDCl_3$)

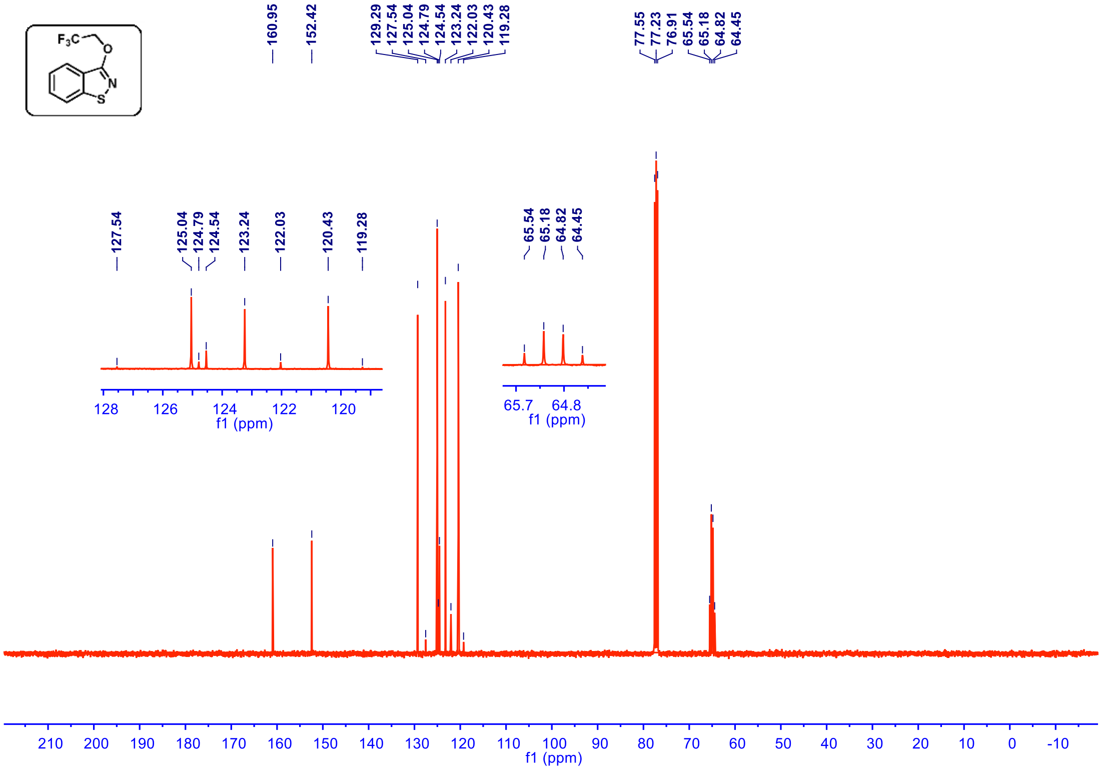

Supplementary Figure 289. ^{13}C NMR spectrum of compound **8** (101 MHz, $CDCl_3$)

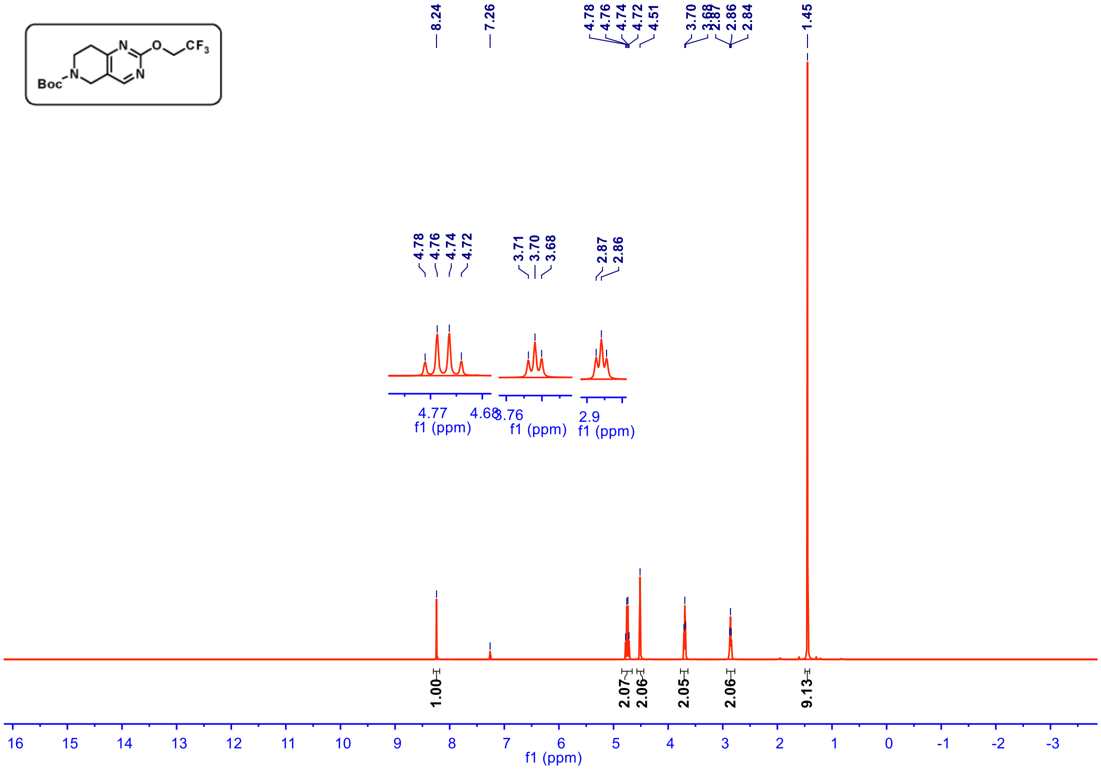

Supplementary Figure 290. ^{1}H NMR spectrum of compound **9** (400 MHz, $CDCl_3$)

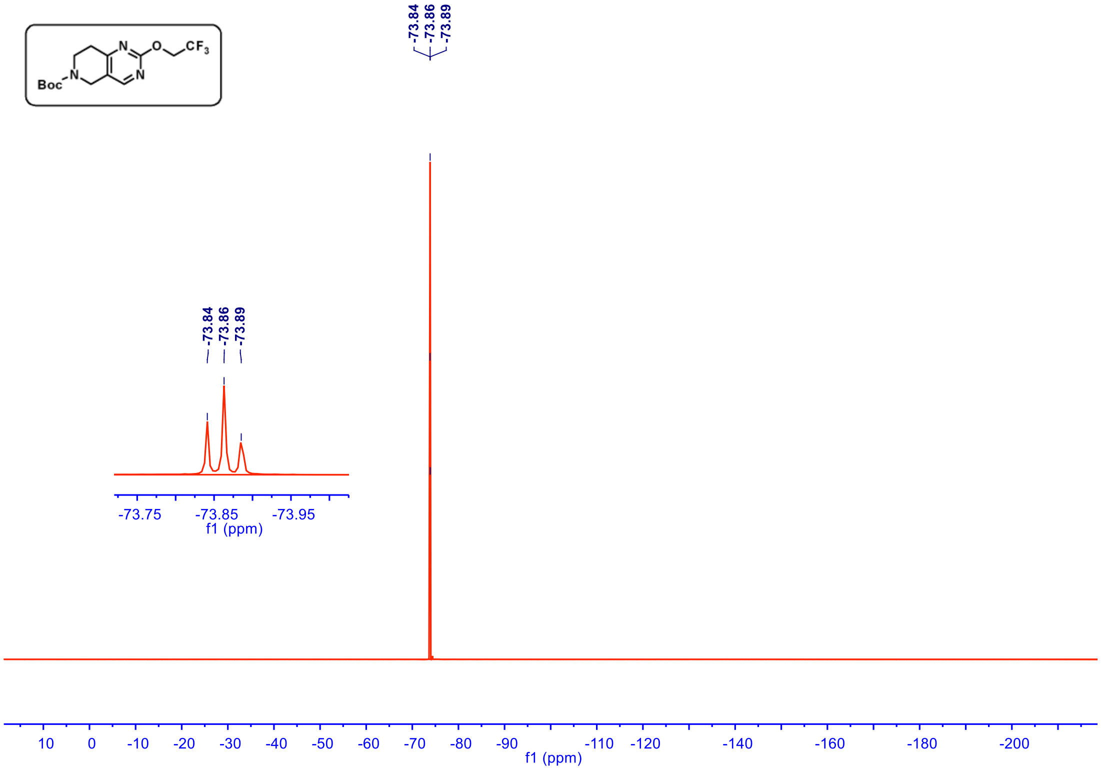

Supplementary Figure 291. ^{19}F NMR spectrum of compound **9** (376 MHz, $CDCl_3$)

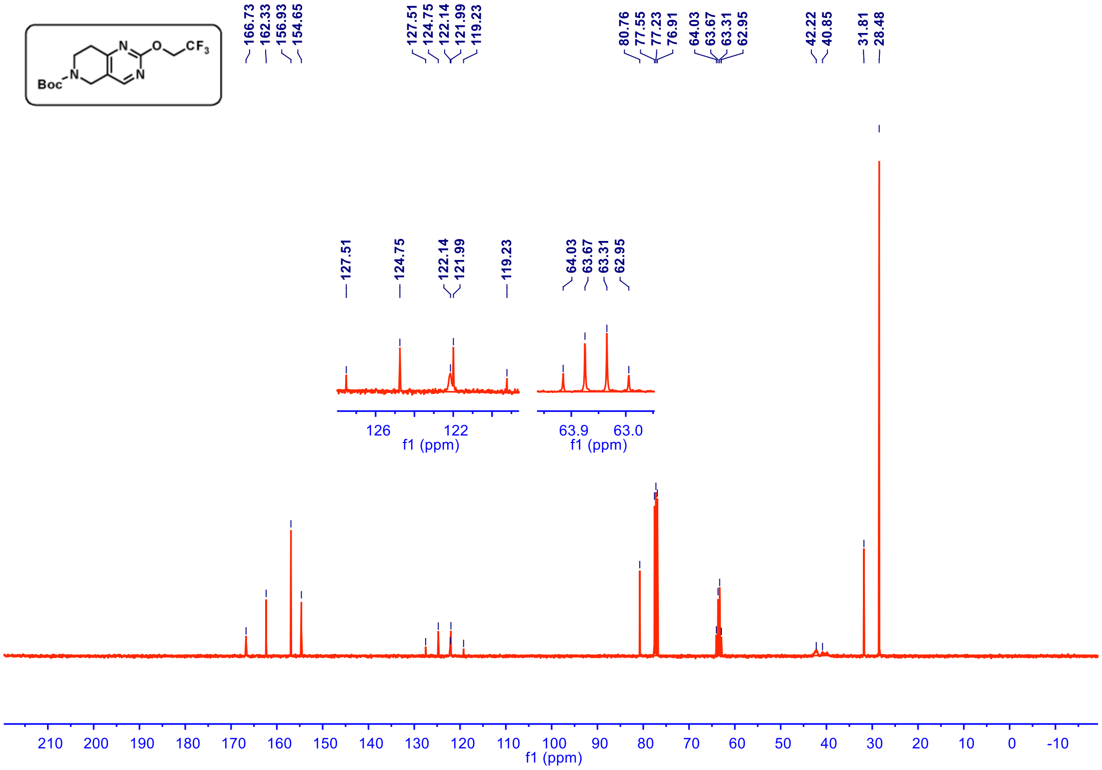

Supplementary Figure 292. ^{13}C NMR spectrum of compound **9** (101 MHz, $CDCl_3$)

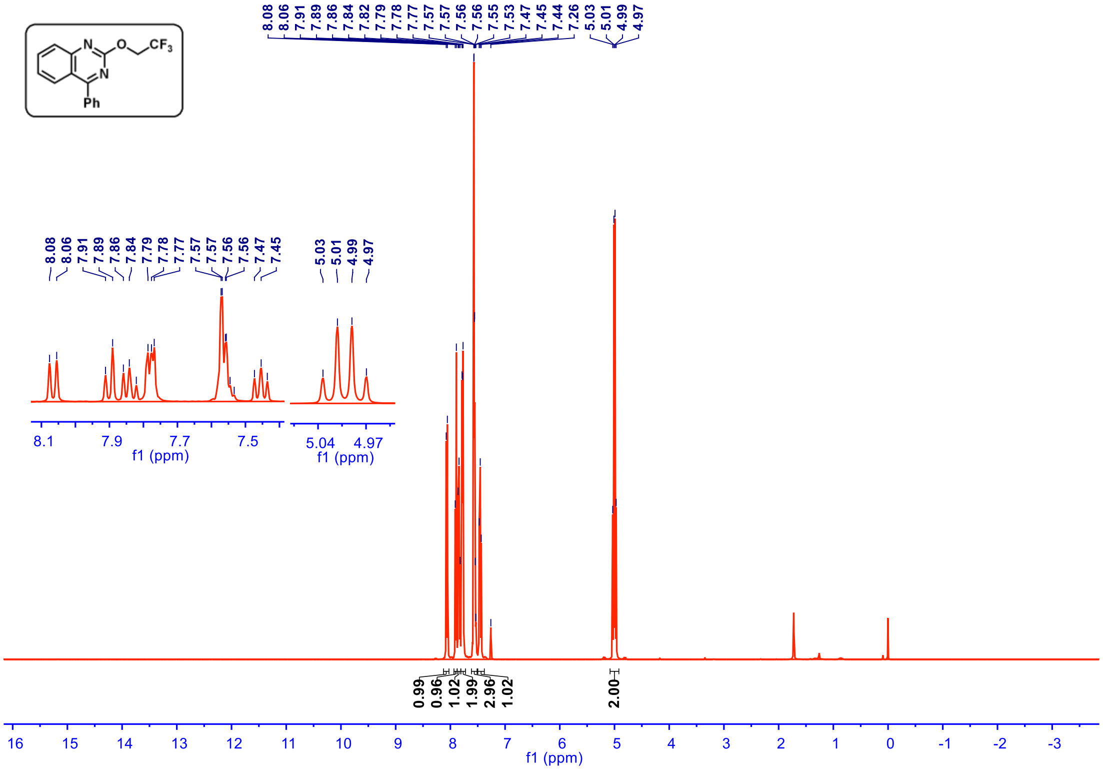

Supplementary Figure 293. ^{1}H NMR spectrum of compound **10** (400 MHz, $CDCl_3$)

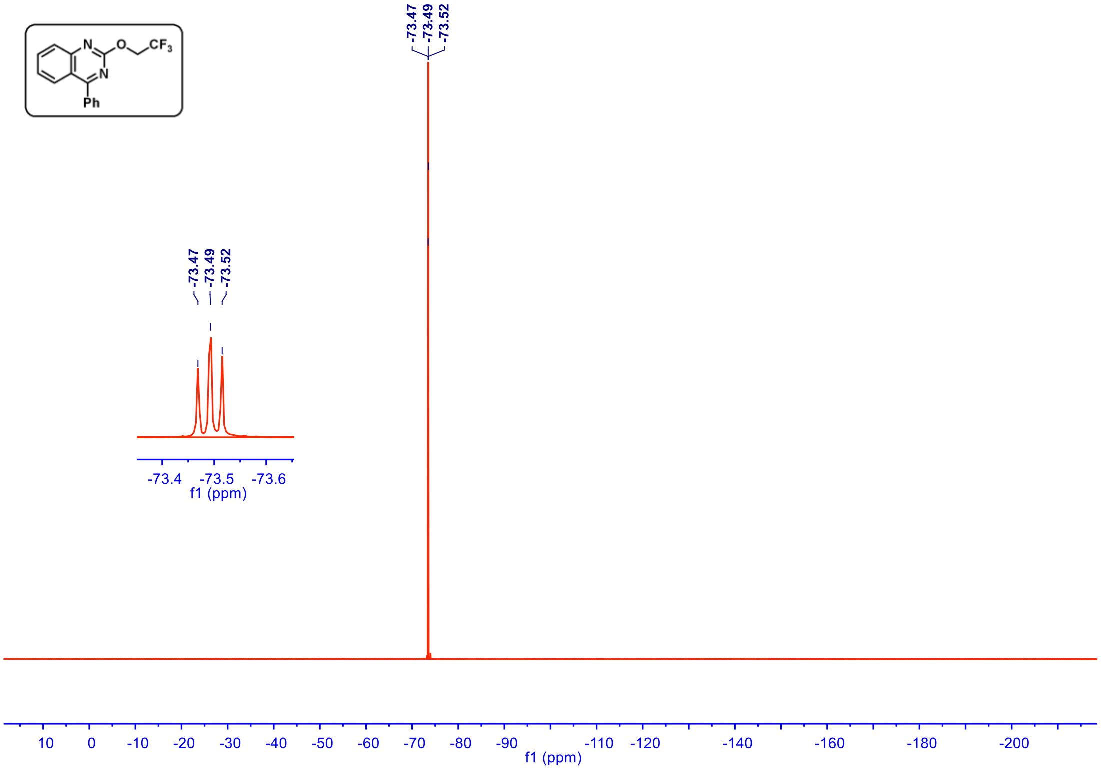

Supplementary Figure 294. ^{19}F NMR spectrum of compound **10** (376 MHz, $CDCl_3$)

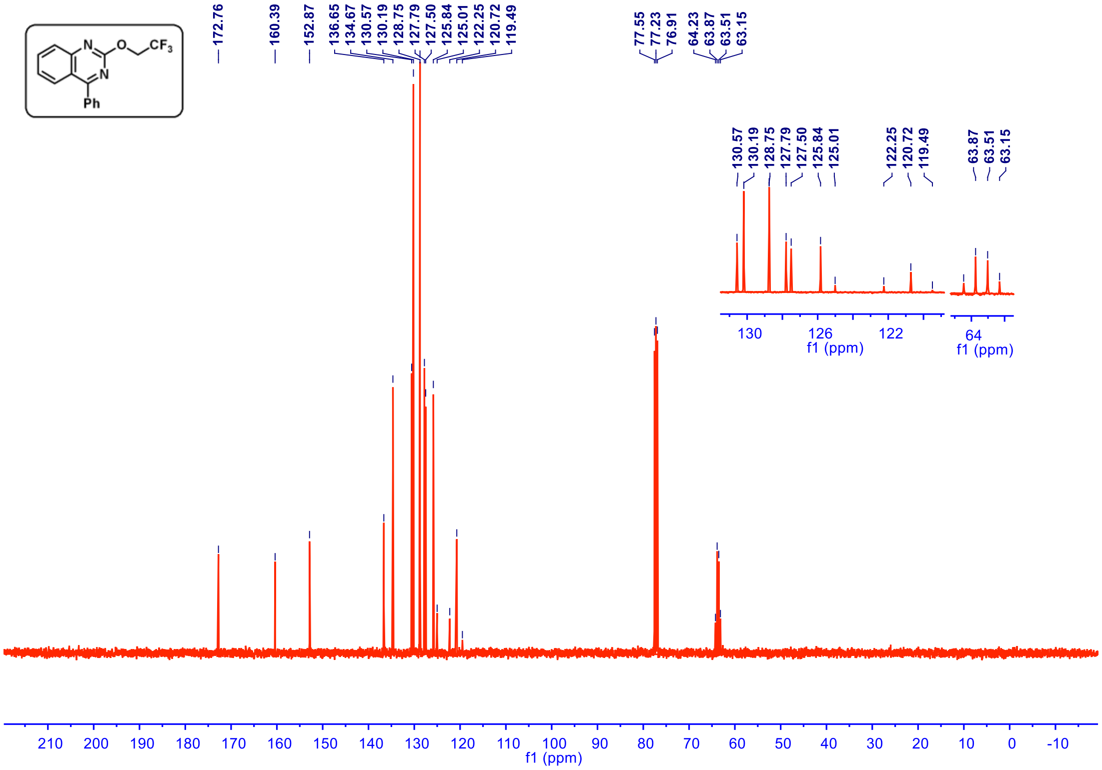

Supplementary Figure 295. ^{13}C NMR spectrum of compound **10** (101 MHz, $CDCl_3$)

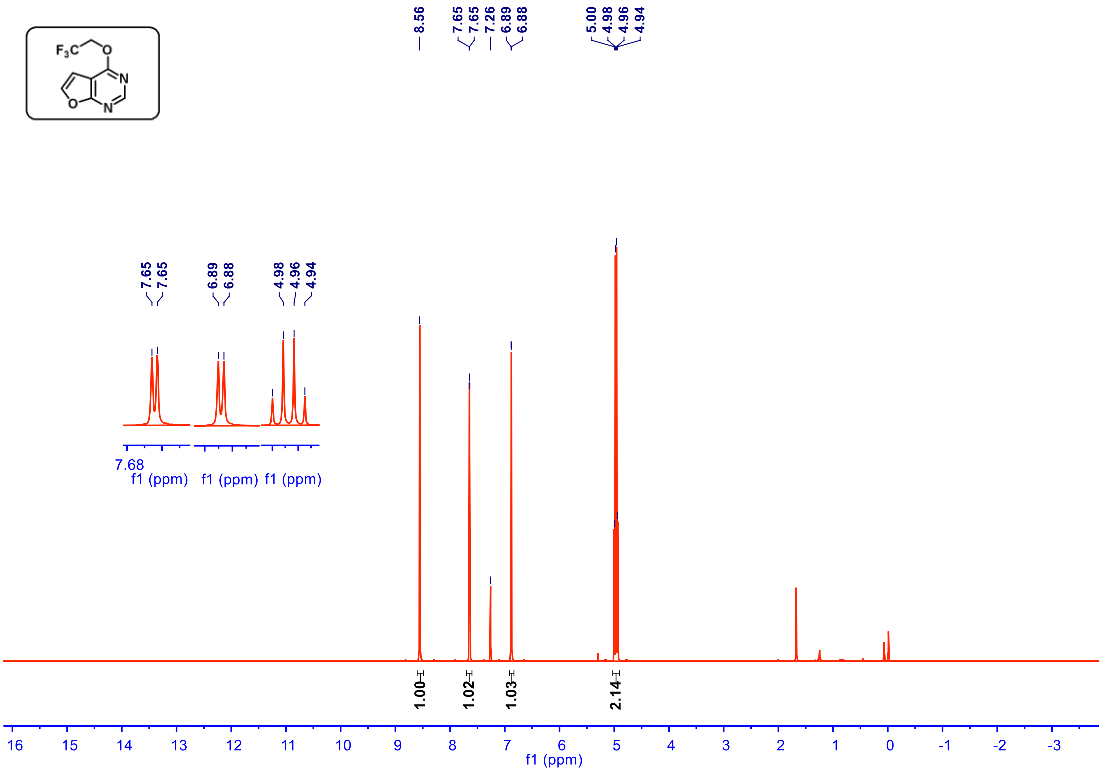

Supplementary Figure 296. ^{1}H NMR spectrum of compound **11** (400 MHz, $CDCl_3$)

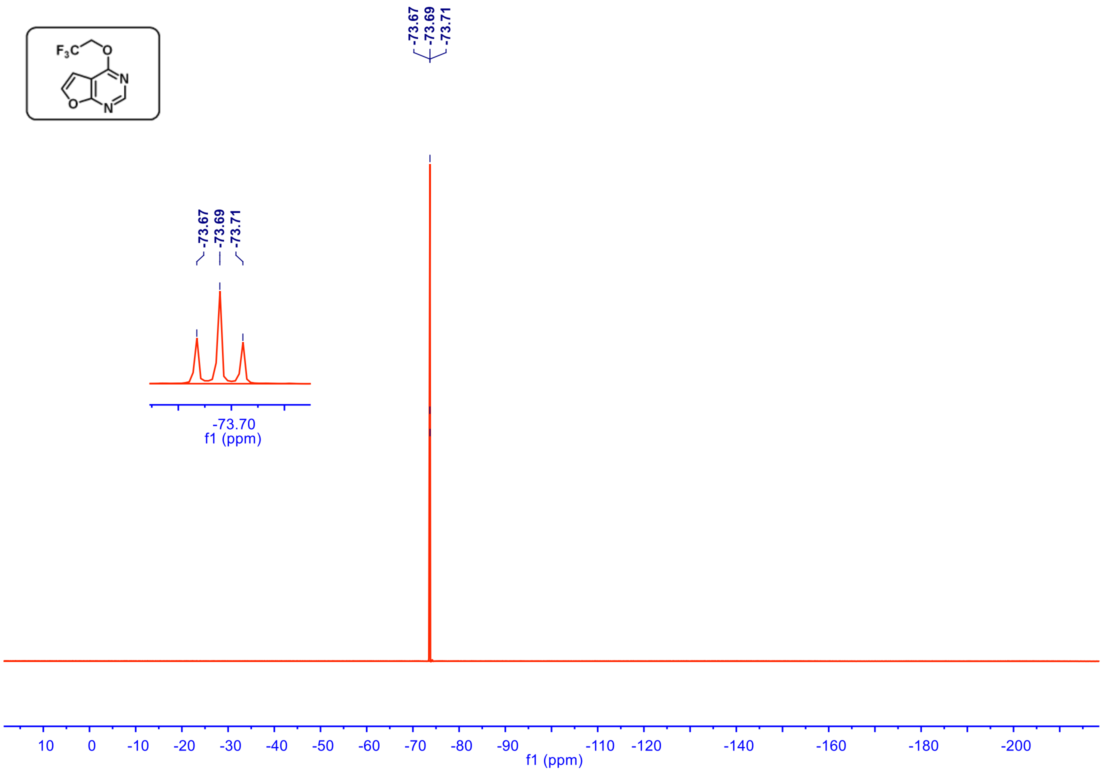

Supplementary Figure 297. ^{19}F NMR spectrum of compound **11** (376 MHz, $CDCl_3$)

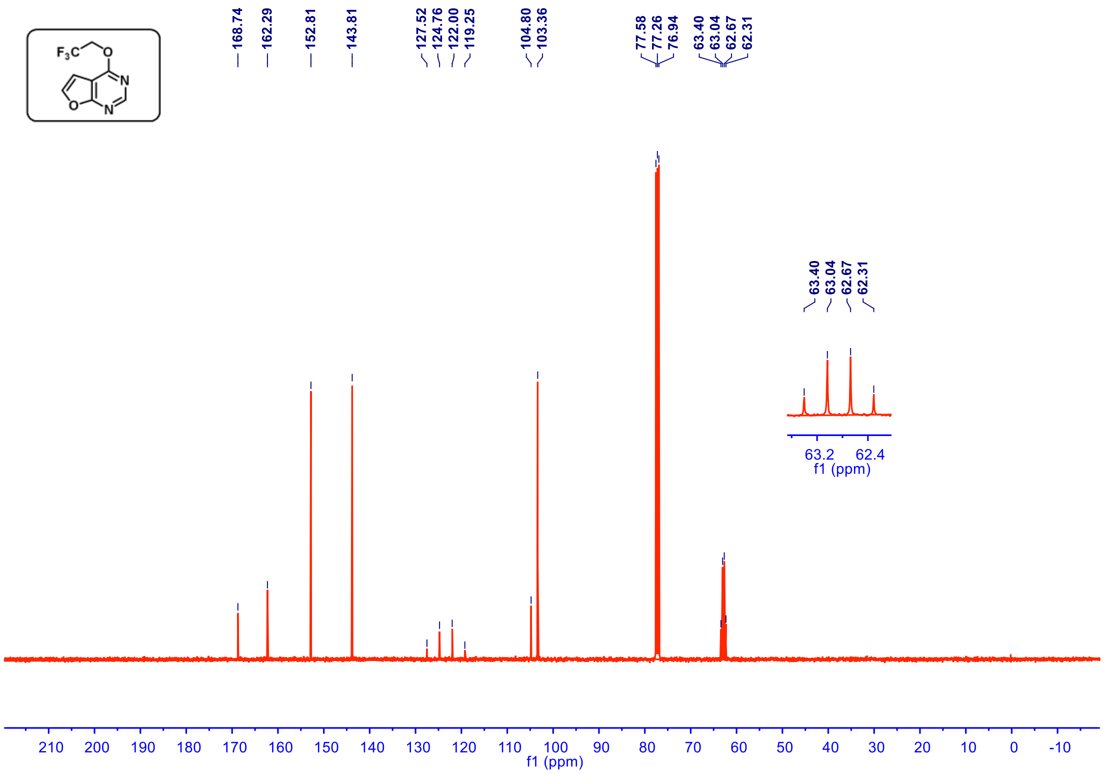

Supplementary Figure 298. ^{13}C NMR spectrum of compound **11** (101 MHz, $CDCl_3$)

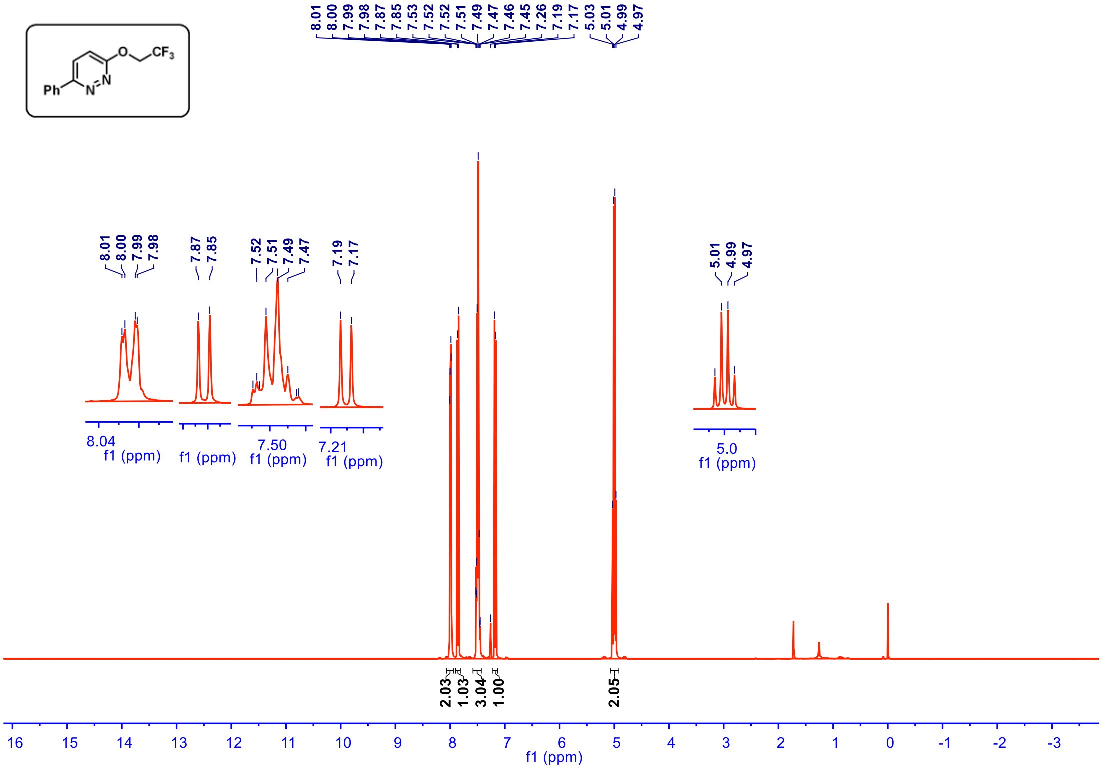

Supplementary Figure 299. ^{1}H NMR spectrum of compound **12** (400 MHz, $CDCl_3$)

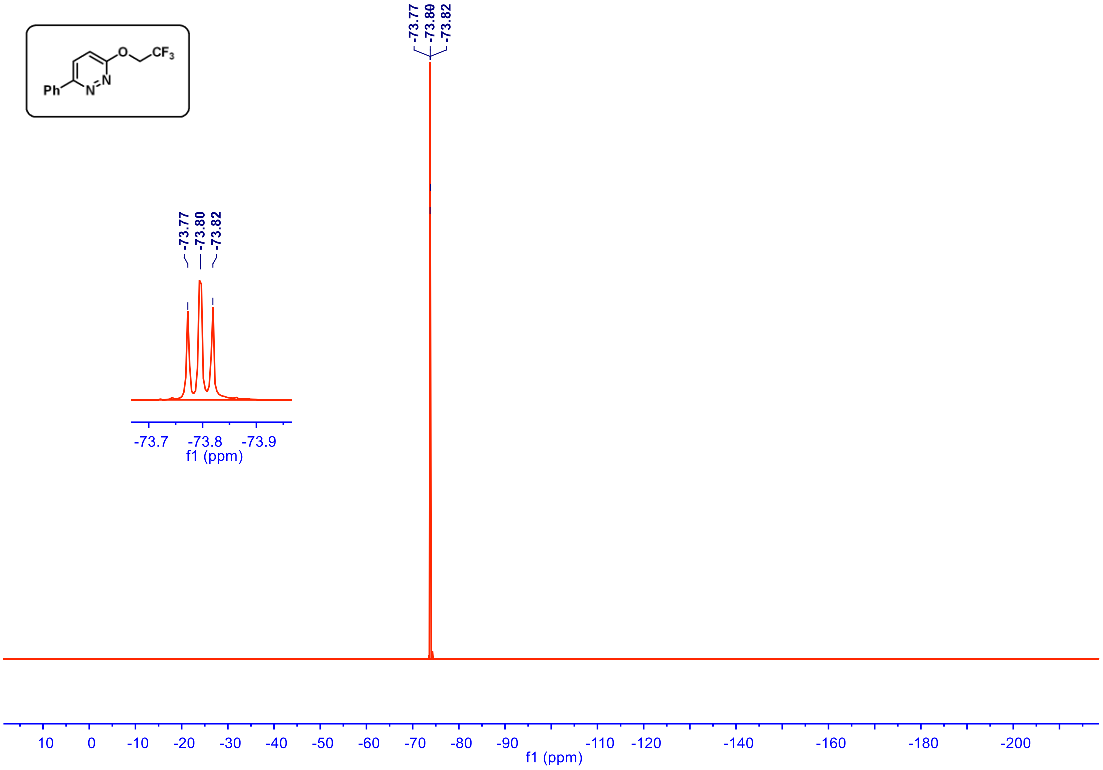

Supplementary Figure 300. ^{19}F NMR spectrum of compound **12** (376 MHz, $CDCl_3$)

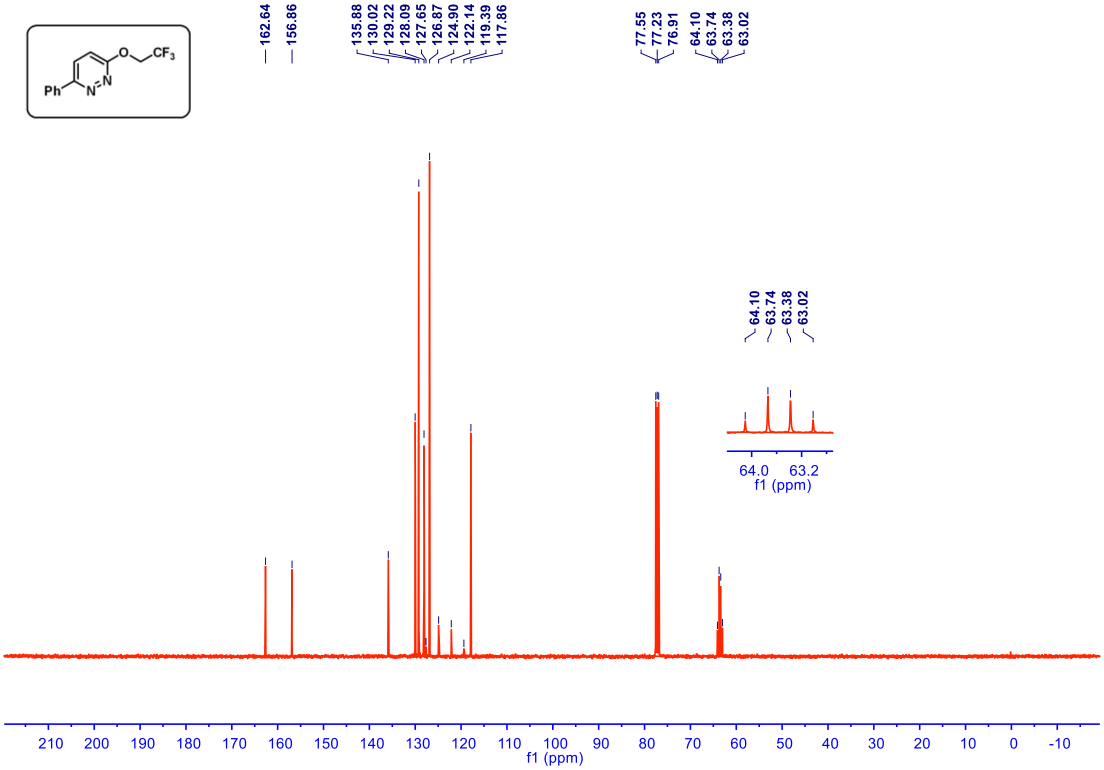

Supplementary Figure 301. ^{13}C NMR spectrum of compound **12** (101 MHz, $CDCl_3$)

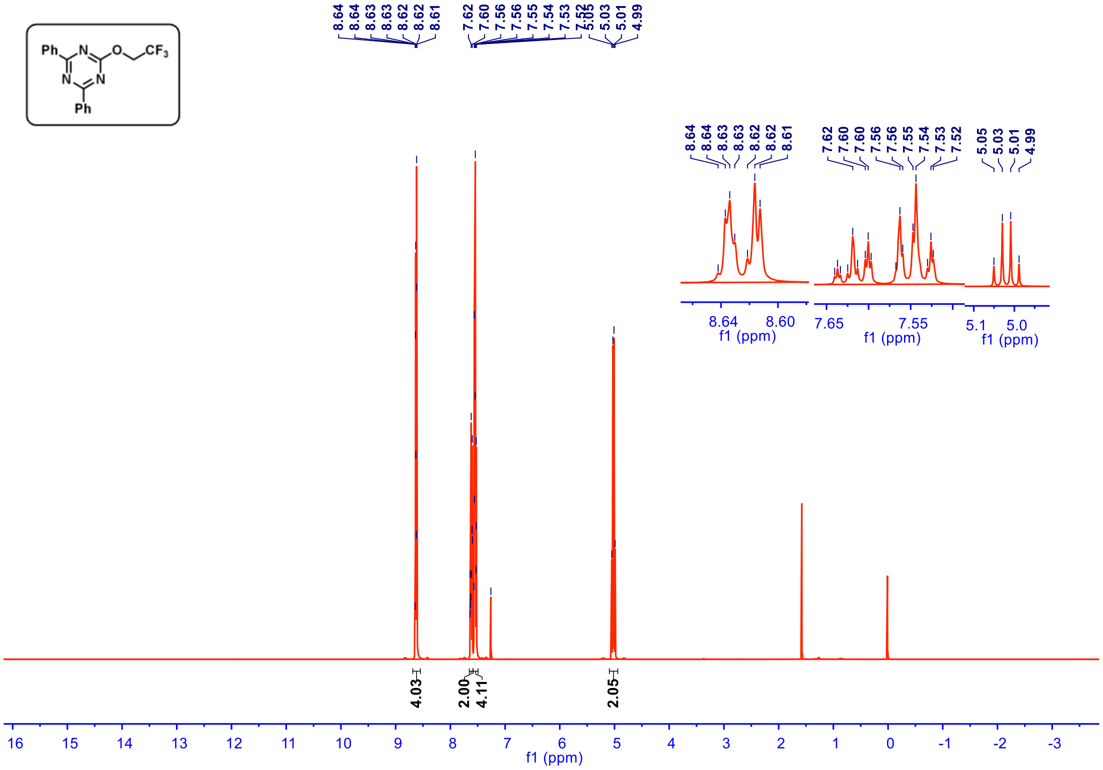

Supplementary Figure 302. ^{1}H NMR spectrum of compound **13** (400 MHz, $CDCl_3$)

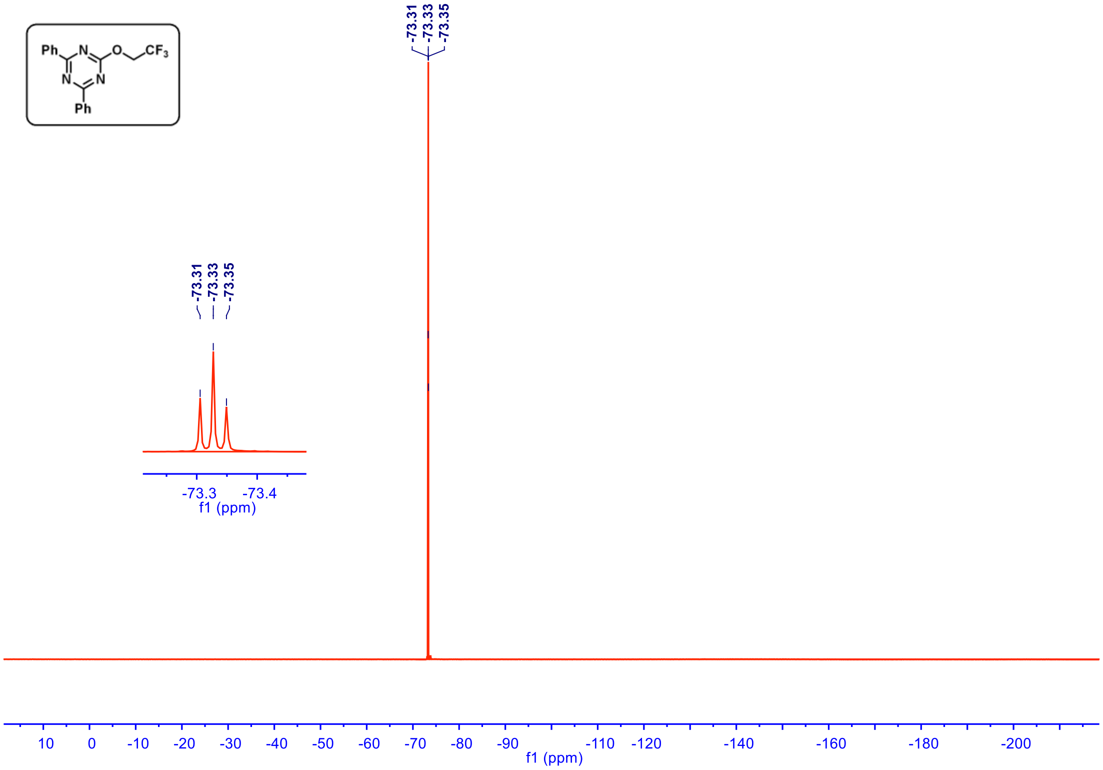

Supplementary Figure 303. ^{19}F NMR spectrum of compound **13** (376 MHz, $CDCl_3$)

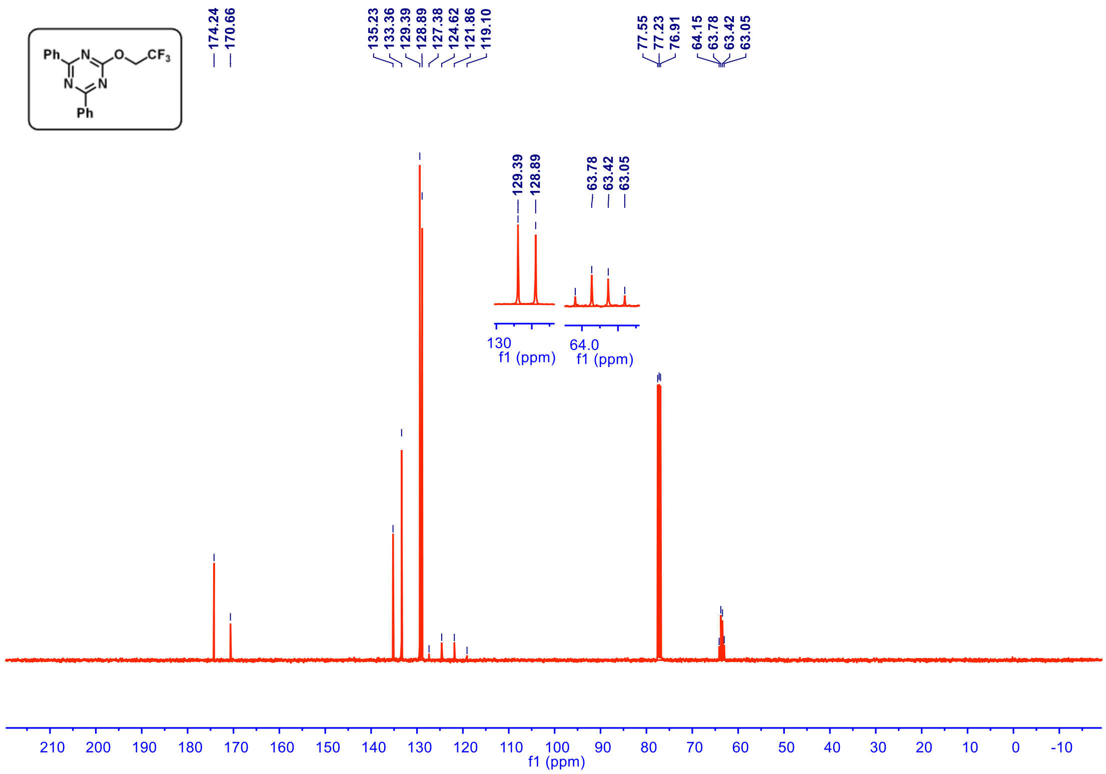

Supplementary Figure 304. ^{13}C NMR spectrum of compound **13** (101 MHz, $CDCl_3$)

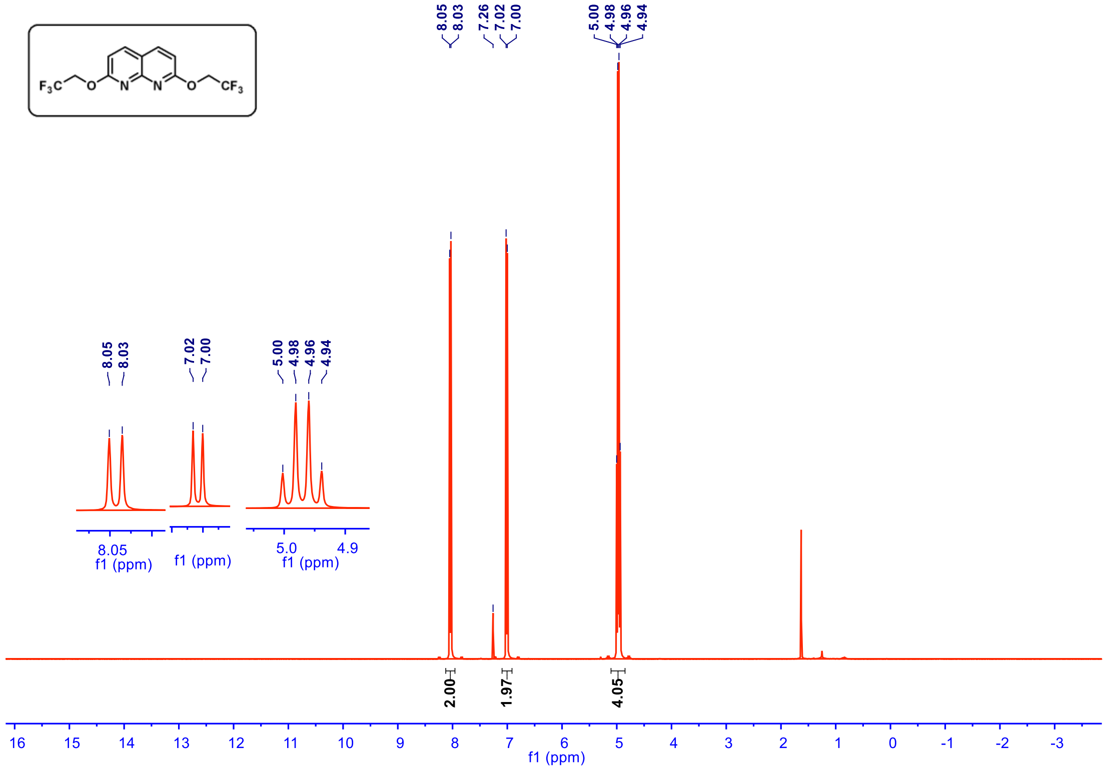

Supplementary Figure 305. ^{1}H NMR spectrum of compound **14** (400 MHz, $CDCl_3$)

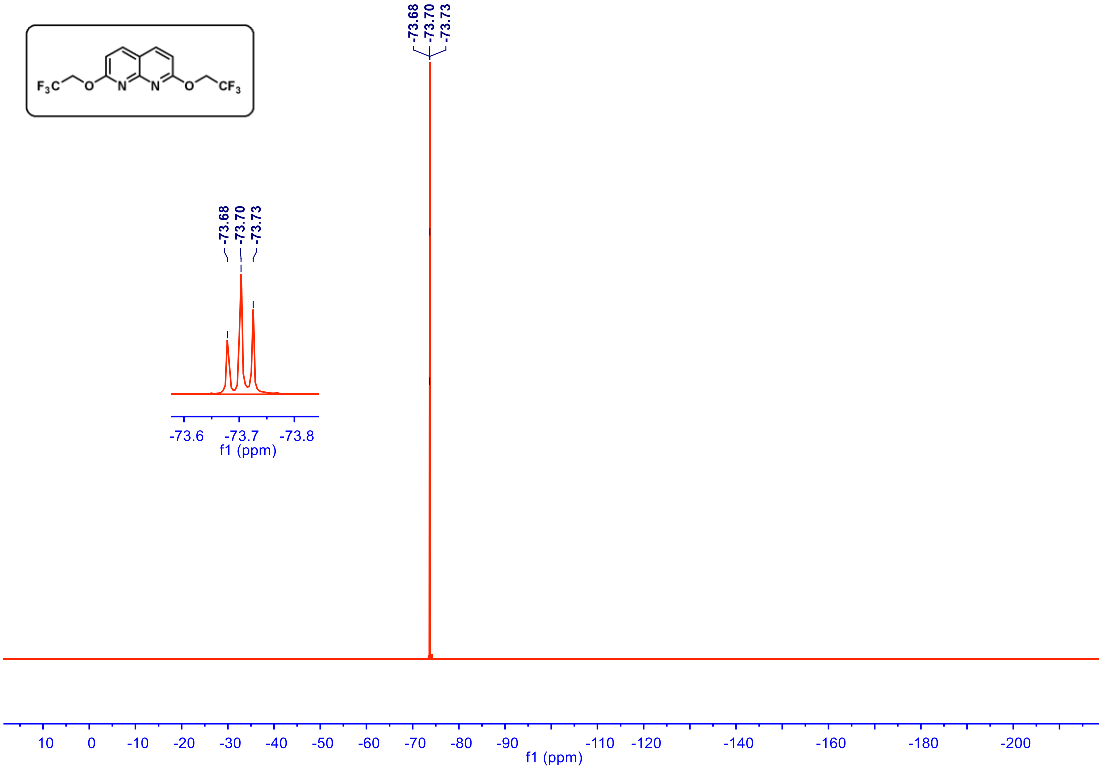

Supplementary Figure 306. ^{19}F NMR spectrum of compound **14** (376 MHz, $CDCl_3$)

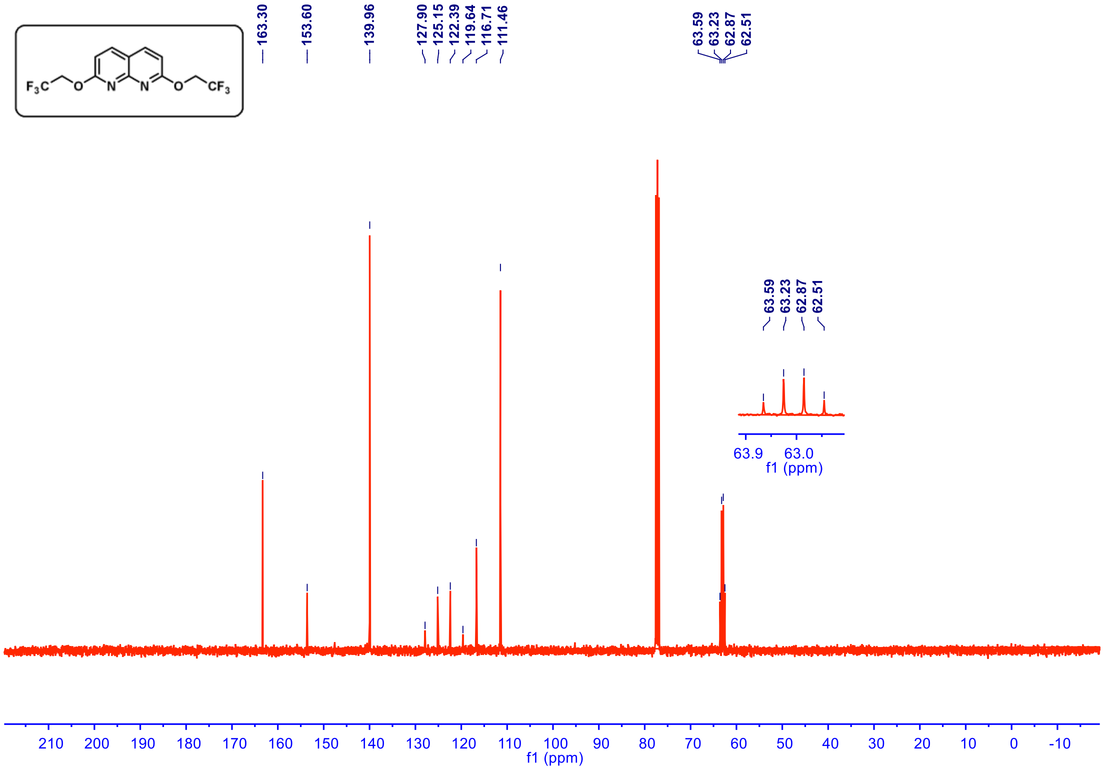

Supplementary Figure 307. ^{13}C NMR spectrum of compound **14** (101 MHz, $CDCl_3$)

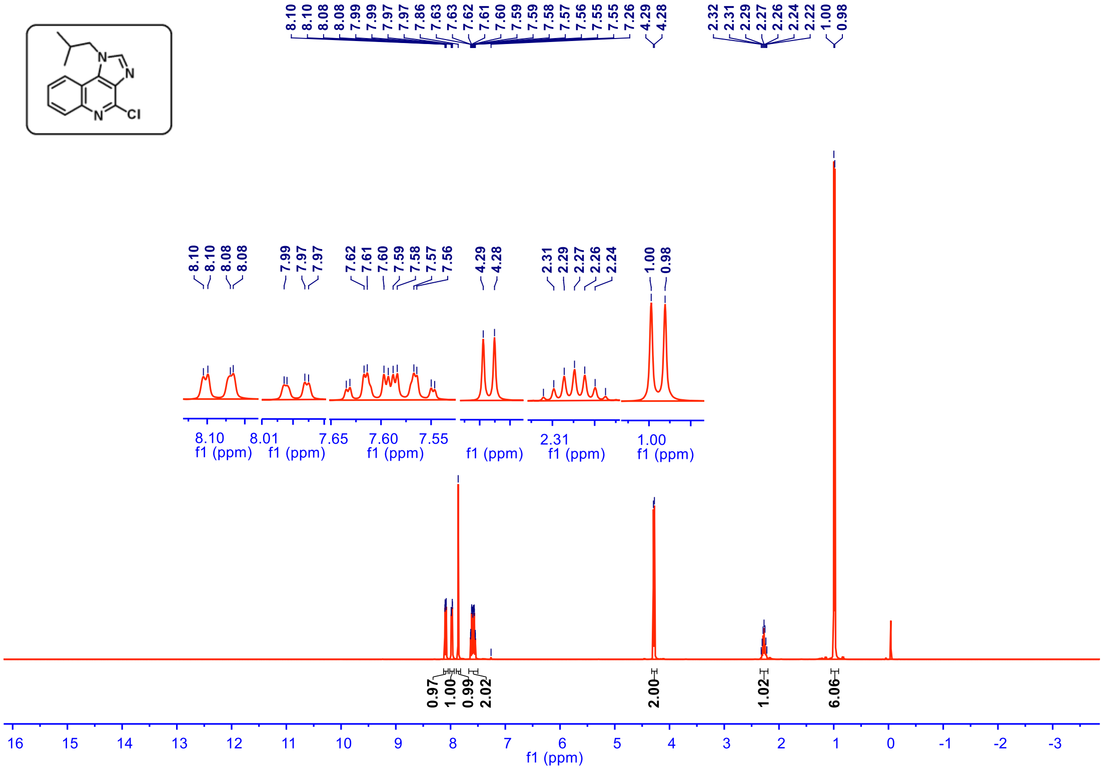

Supplementary Figure 308. ^{1}H NMR spectrum of **4-chloro-1-isobutyl-1*H*–imidazo[4,5-*c*]quinoline** (400 MHz, $CDCl_3$)

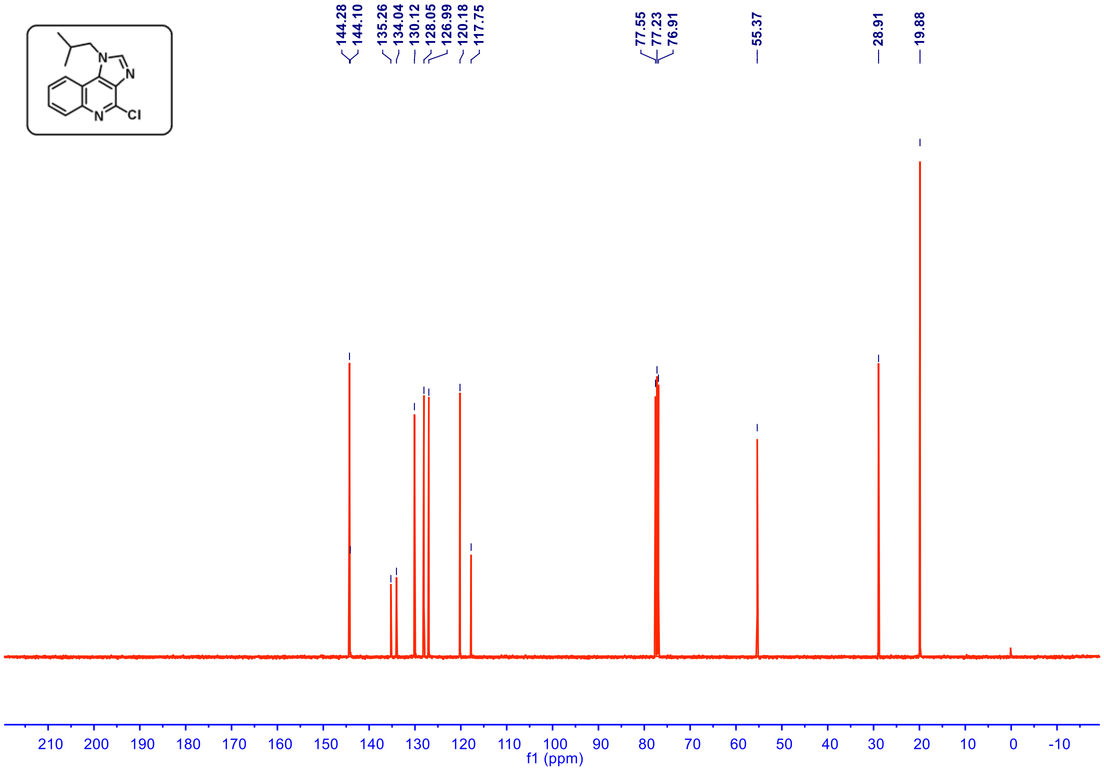

Supplementary Figure 309. ^{13}C NMR spectrum of **4-chloro-1-isobutyl-1*H*–imidazo[4,5-*c*]quinoline** (101 MHz, $CDCl_3$)

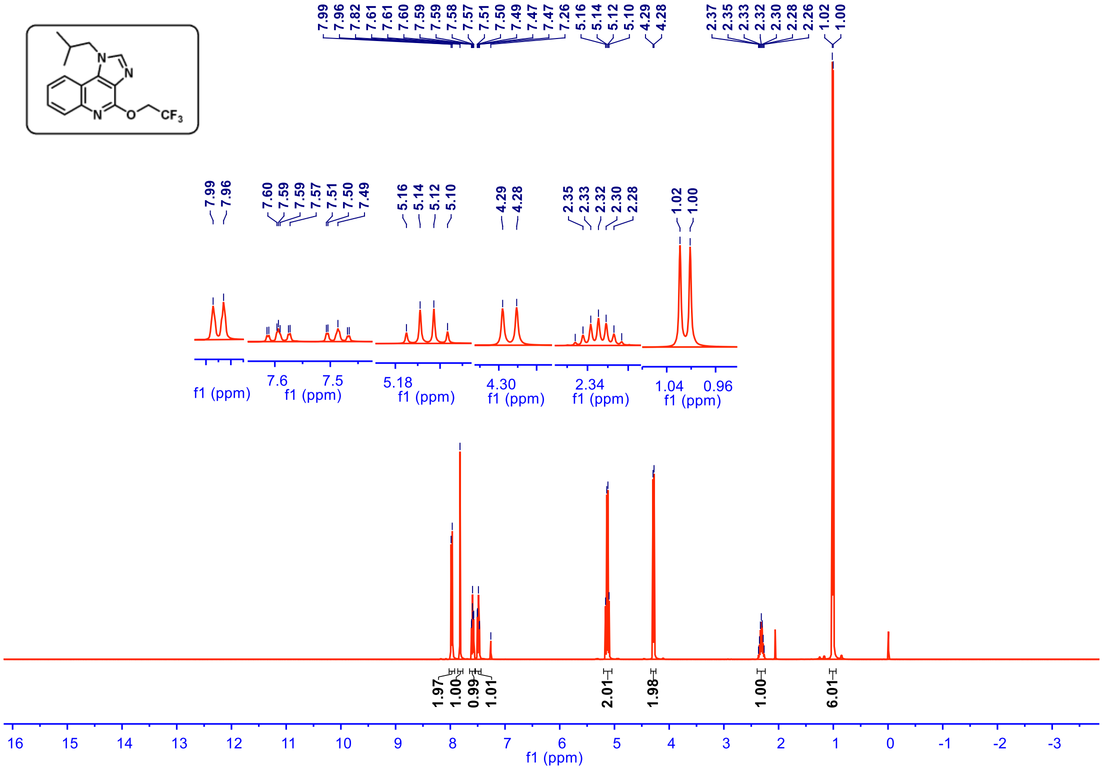

Supplementary Figure 310. ^{1}H NMR spectrum of compound **15** (400 MHz, $CDCl_3$)

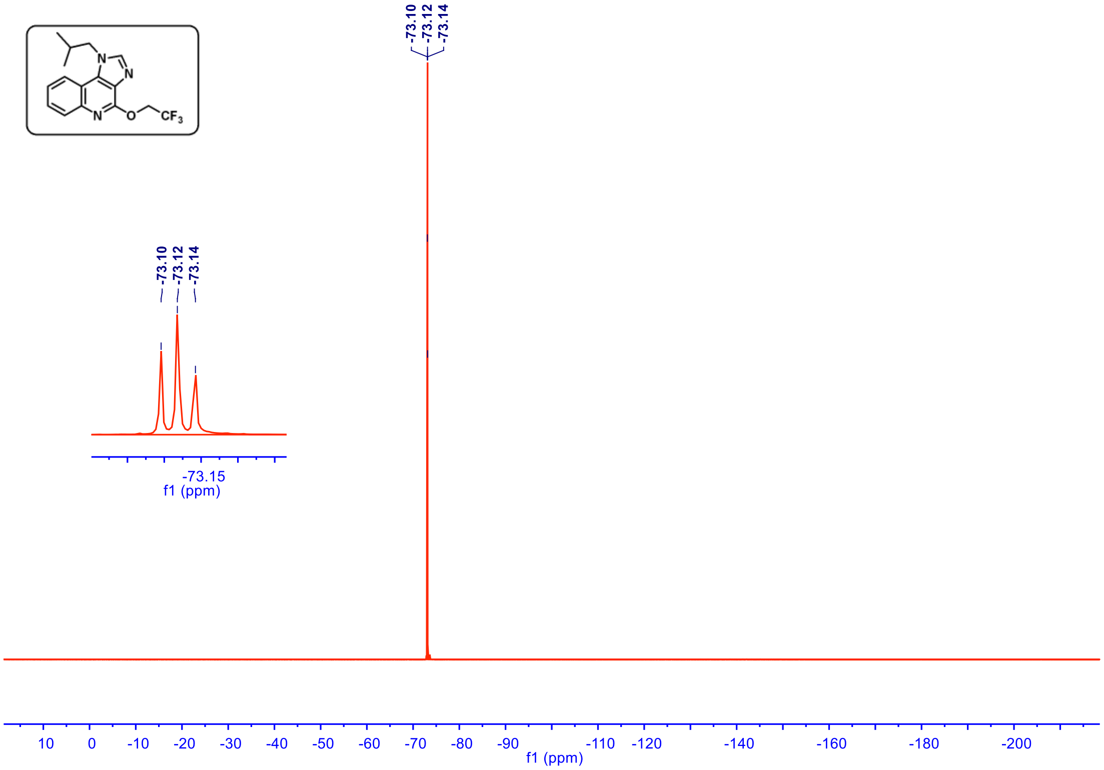

Supplementary Figure 311. ^{19}F NMR spectrum of compound **15** (376 MHz, $CDCl_3$)

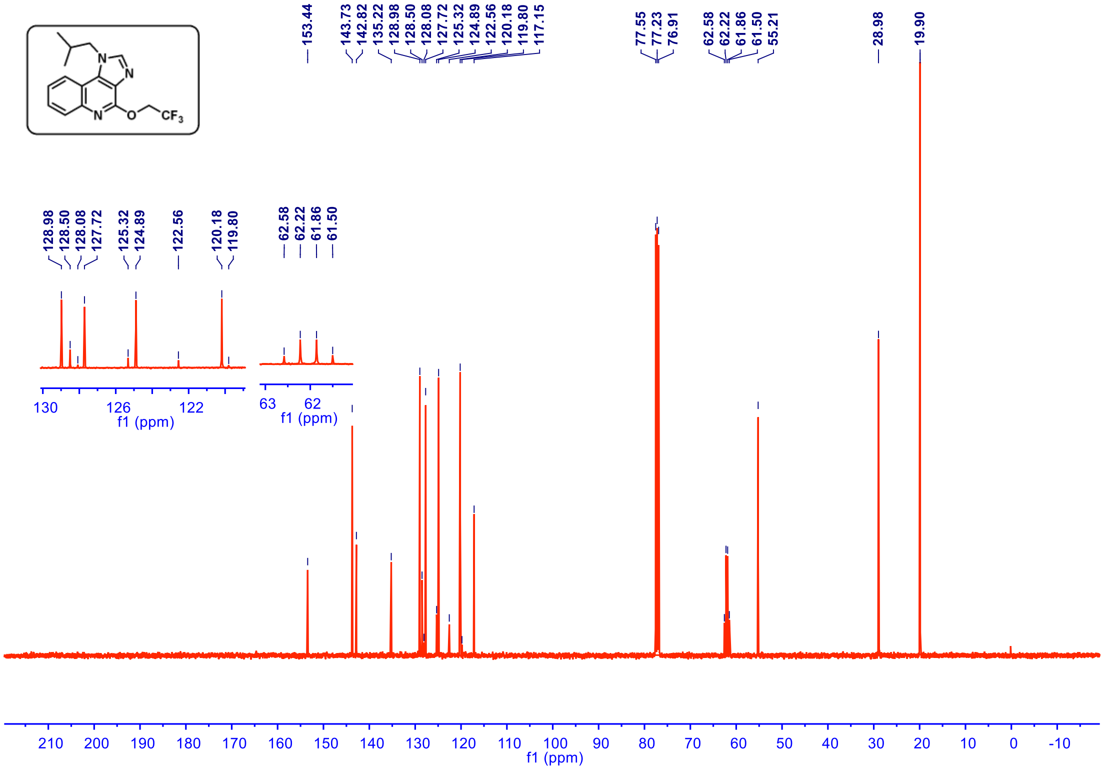

Supplementary Figure 312. ^{13}C NMR spectrum of compound **15** (101 MHz, $CDCl_3$)

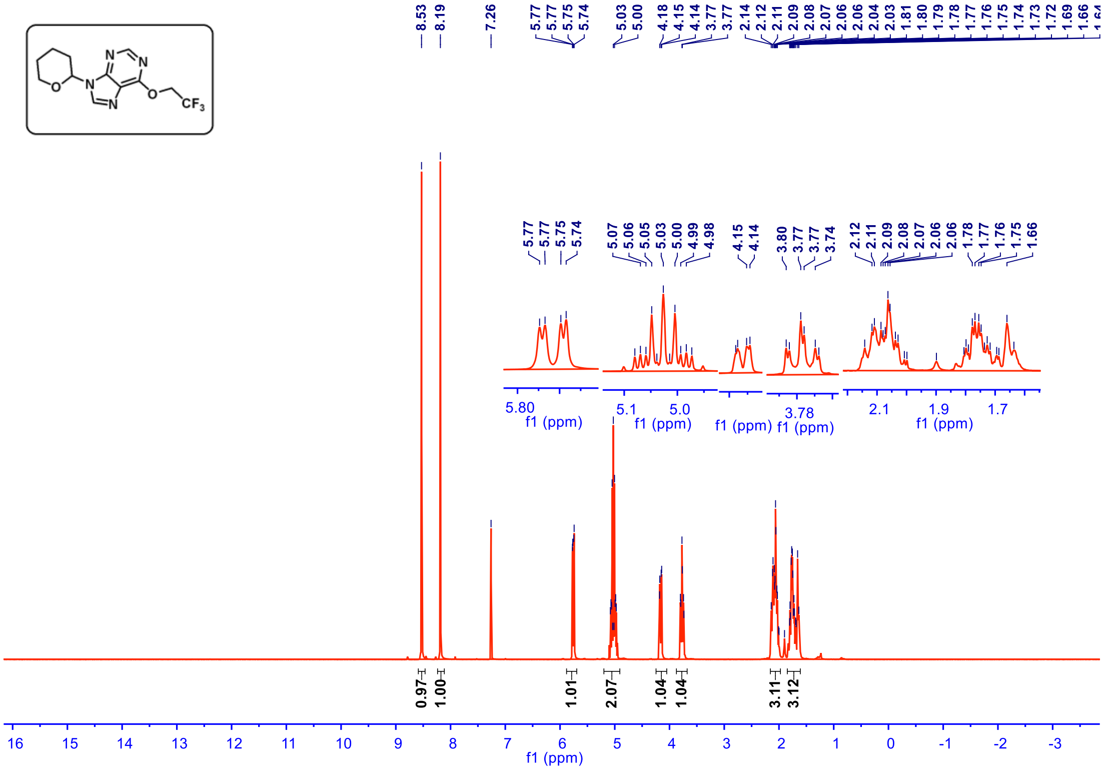

Supplementary Figure 313. ^{1}H NMR spectrum of compound **16** (400 MHz, $CDCl_3$)

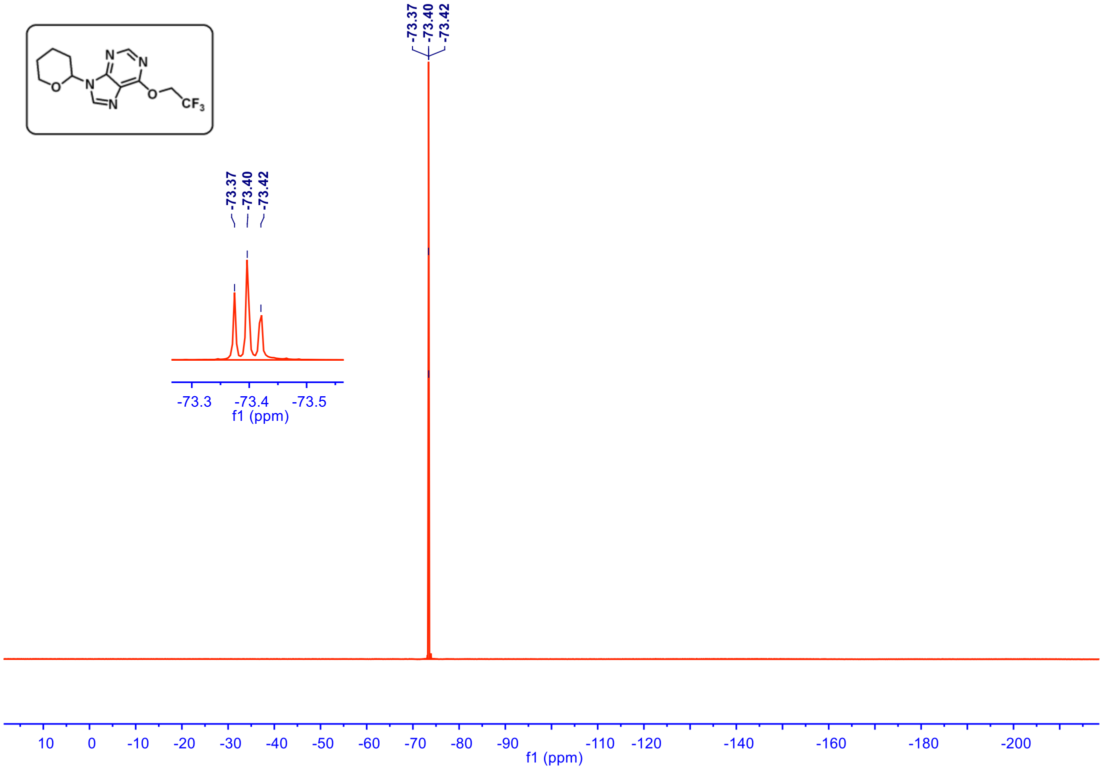

Supplementary Figure 314. ^{19}F NMR spectrum of compound **16** (376 MHz, $CDCl_3$)

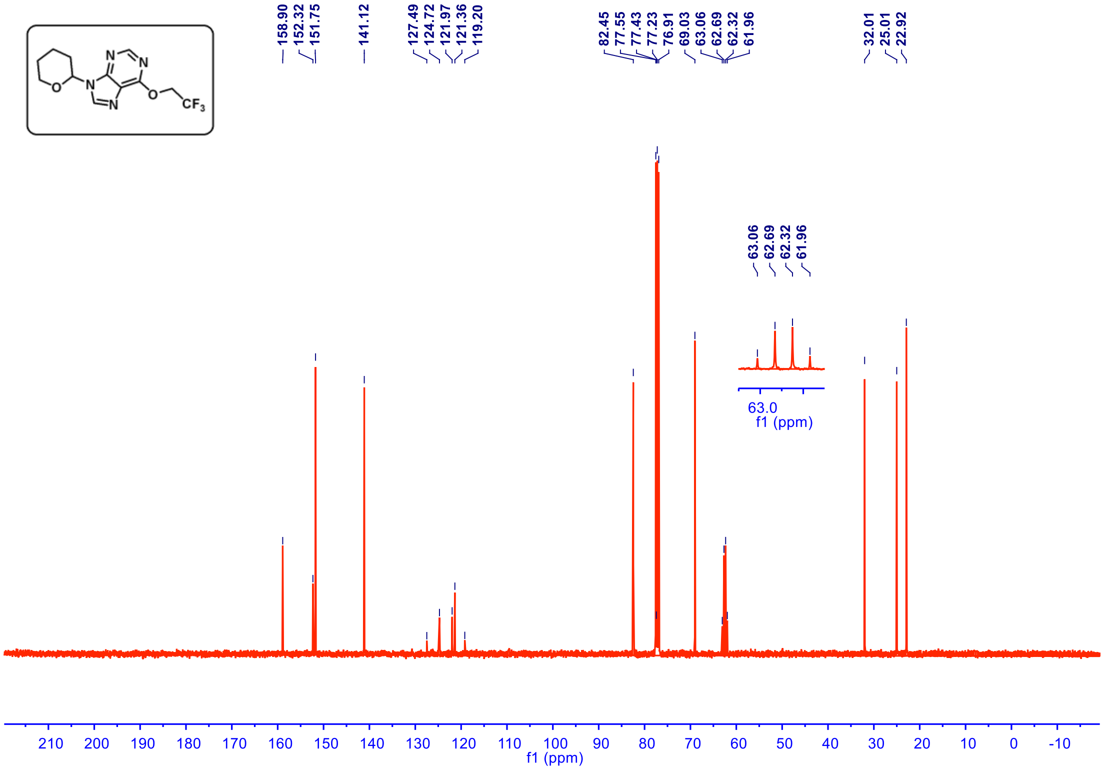

Supplementary Figure 315. ^{13}C NMR spectrum of compound **16** (101 MHz, $CDCl_3$)

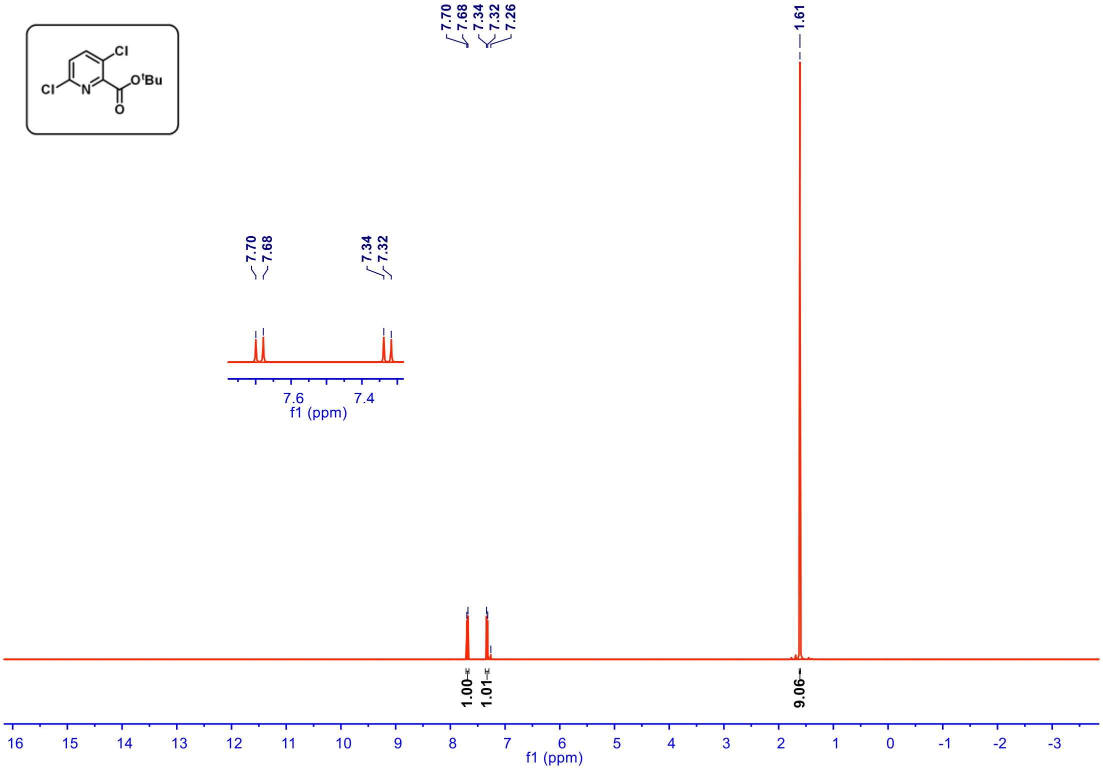

Supplementary Figure 316. ^{1}H NMR spectrum of ***tert*-butyl 3,6-dichloropicolinate** (400 MHz, $CDCl_3$)

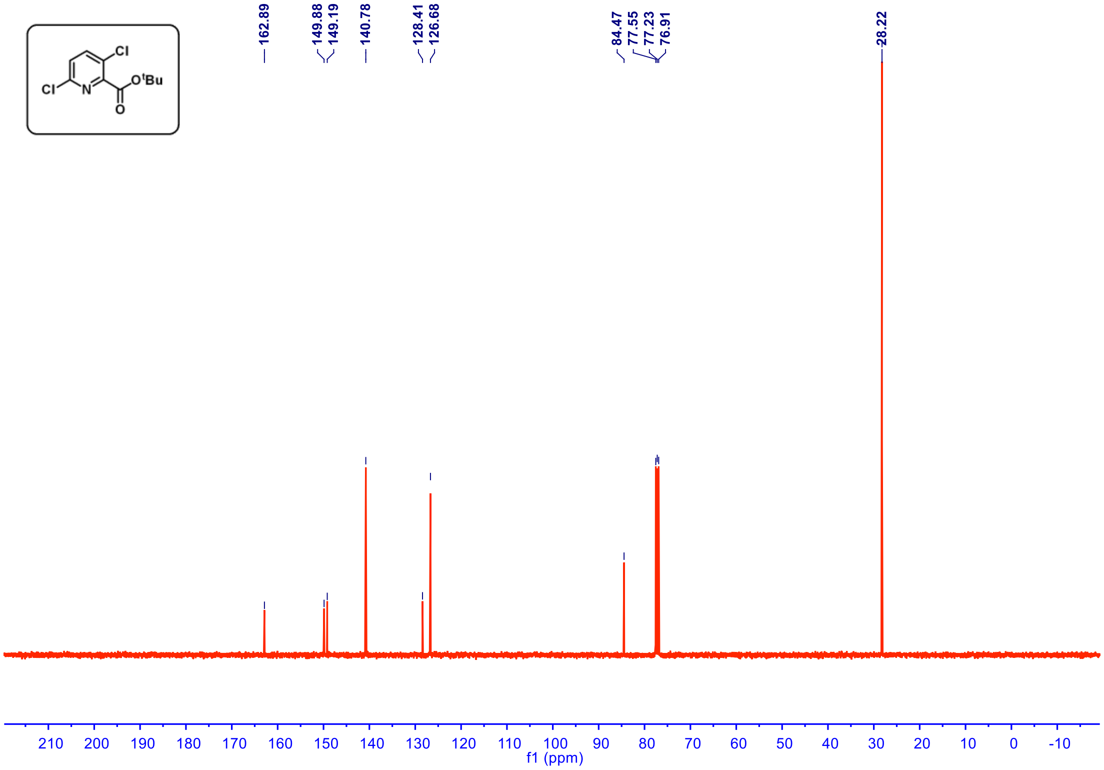

Supplementary Figure 317. ^{13}C NMR spectrum of ***tert*-butyl 3,6-dichloropicolinate** (101 MHz, $CDCl_3$)

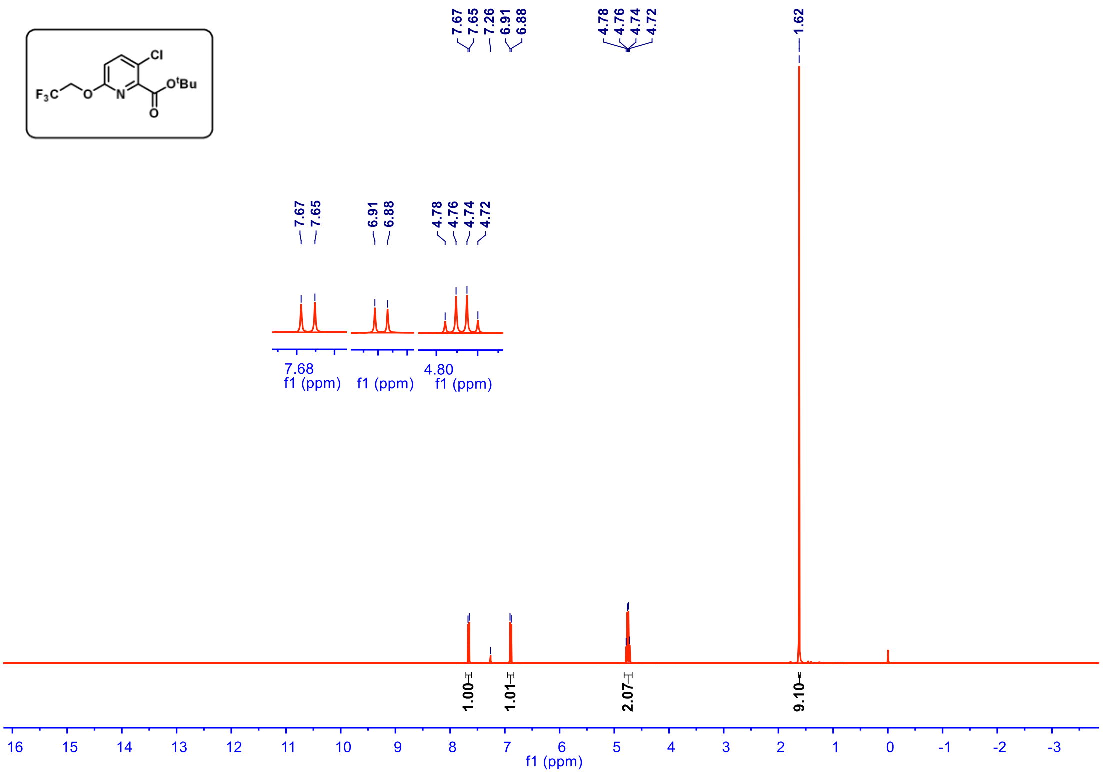

Supplementary Figure 318. ^{1}H NMR spectrum of compound **17** (400 MHz, $CDCl_3$)

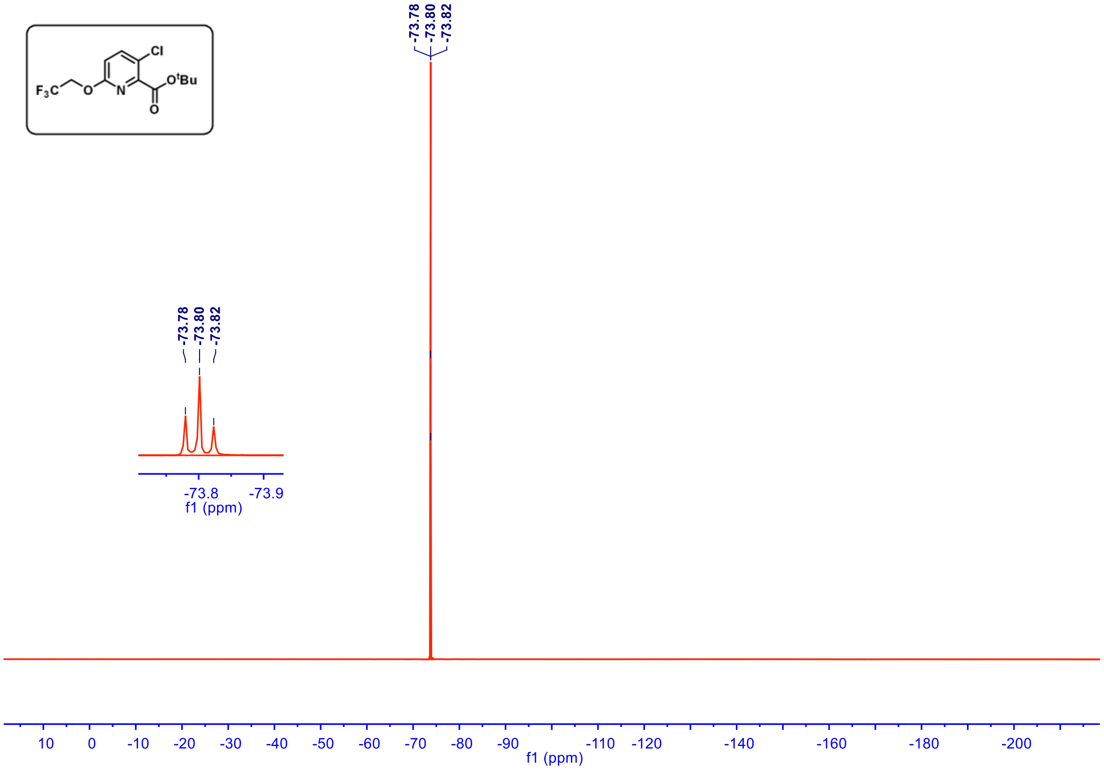

Supplementary Figure 319. ^{19}F NMR spectrum of compound **17** (376 MHz, $CDCl_3$)

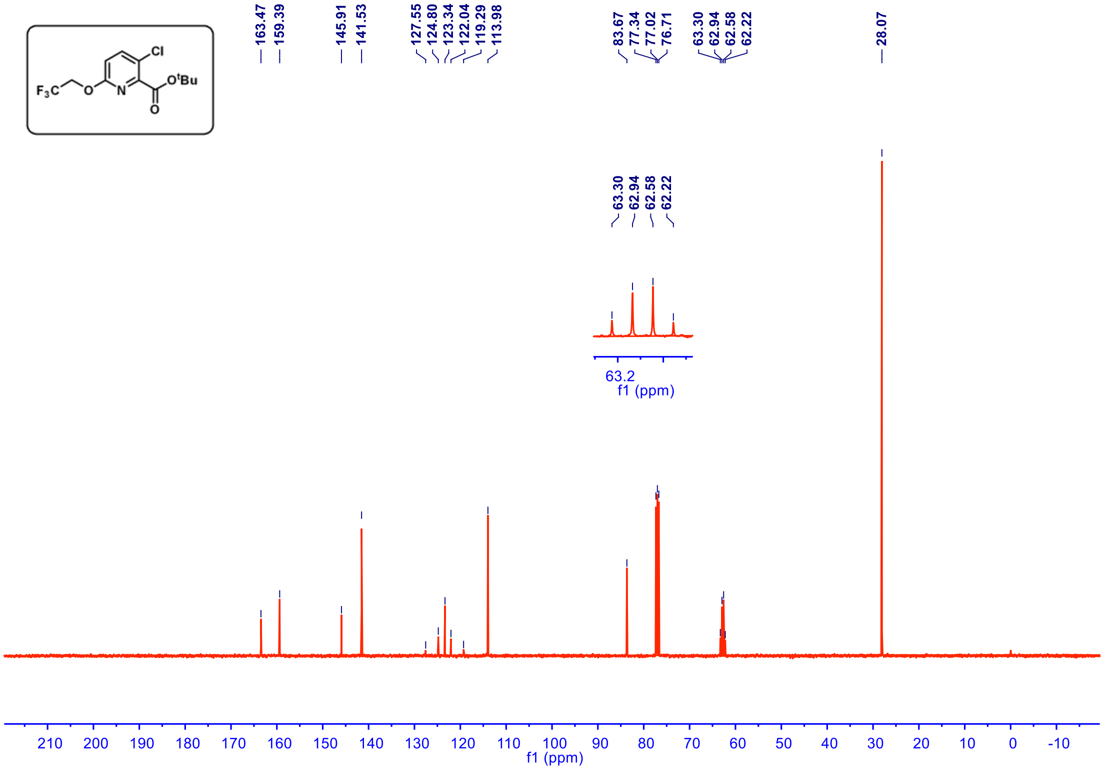

Supplementary Figure 320. ^{13}C NMR spectrum of compound **17** (101 MHz, $CDCl_3$)

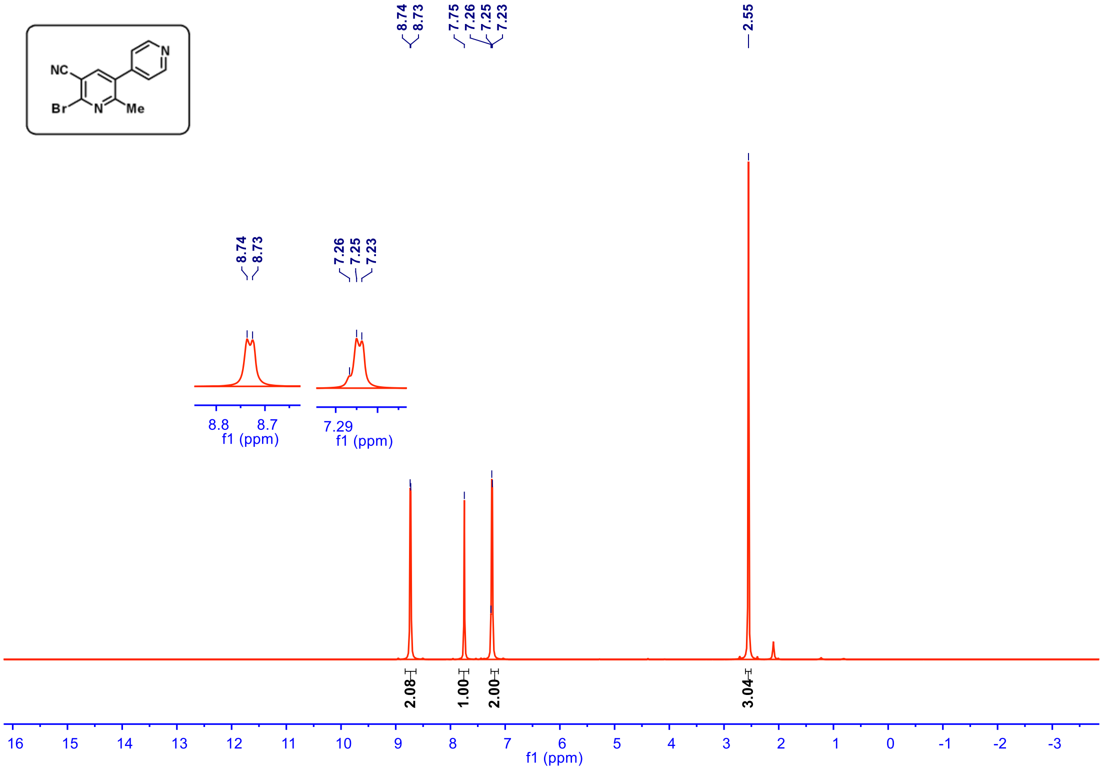

Supplementary Figure 321. ^{1}H NMR spectrum of **6-bromo-2-methyl-3,4'-bipyridine-5-carbonitrile** (400 MHz, $CDCl_3$)

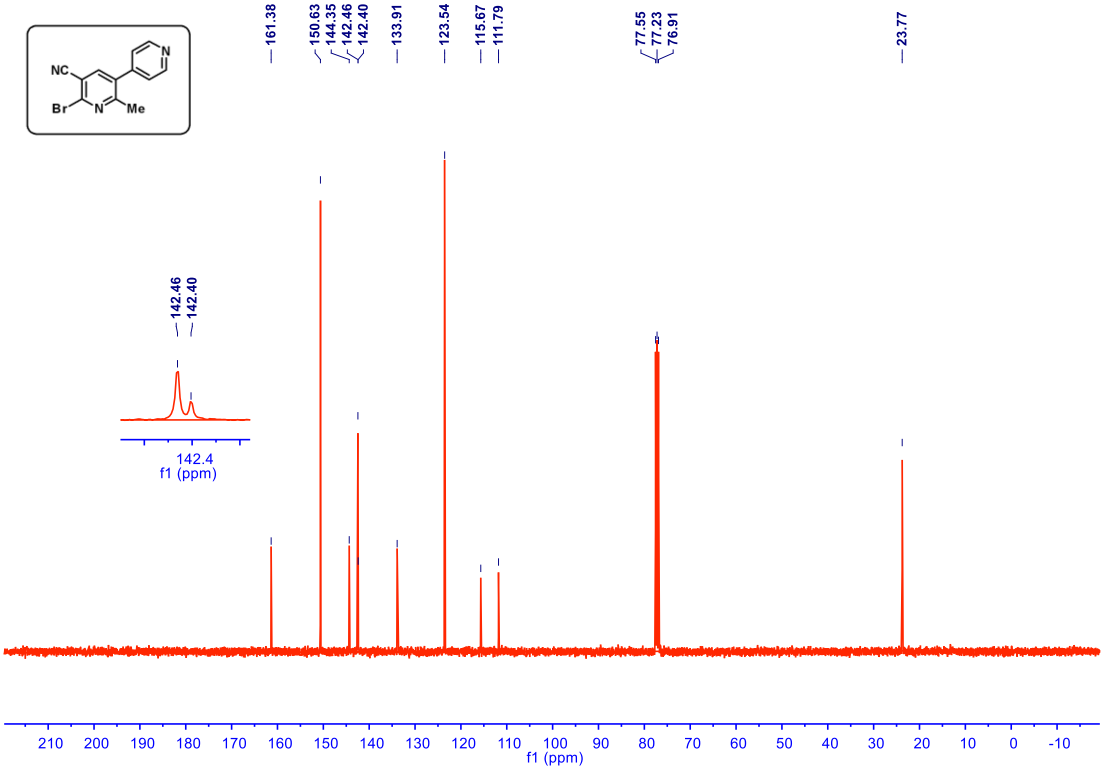

Supplementary Figure 322. ^{13}C NMR spectrum of **6-bromo-2-methyl-3,4'-bipyridine-5-carbonitrile** (101 MHz, $CDCl_3$)

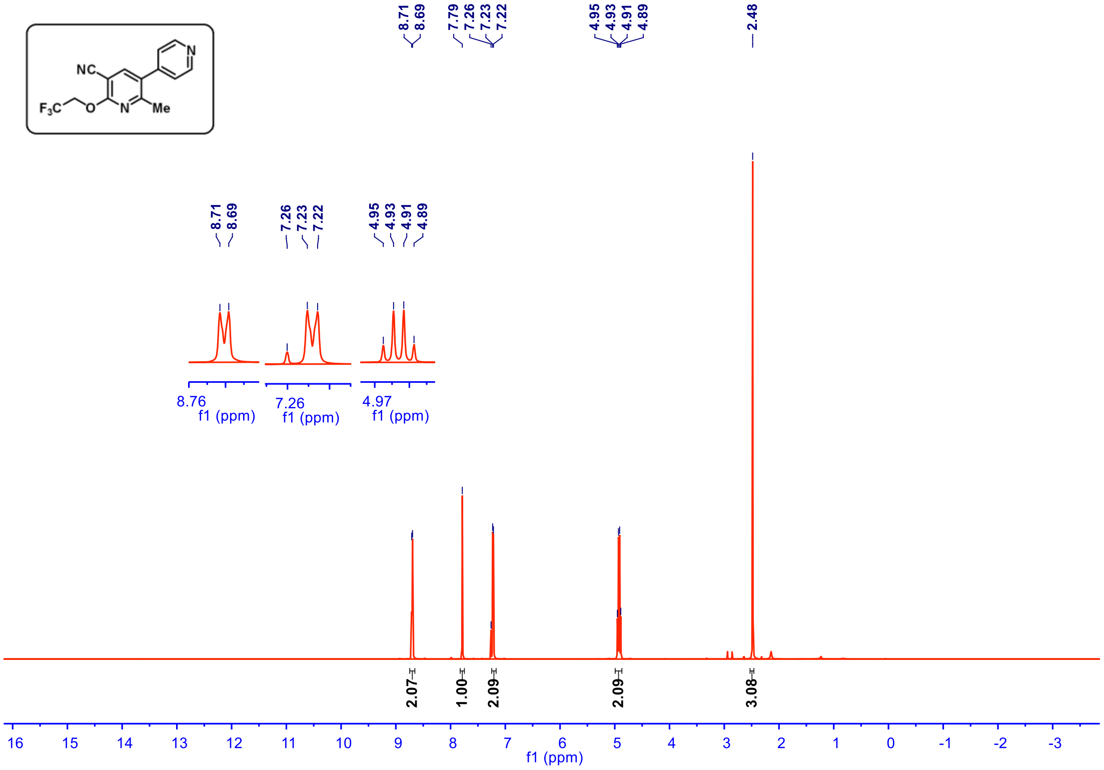

Supplementary Figure 323. ^{1}H NMR spectrum of compound **18** (400 MHz, $CDCl_3$)

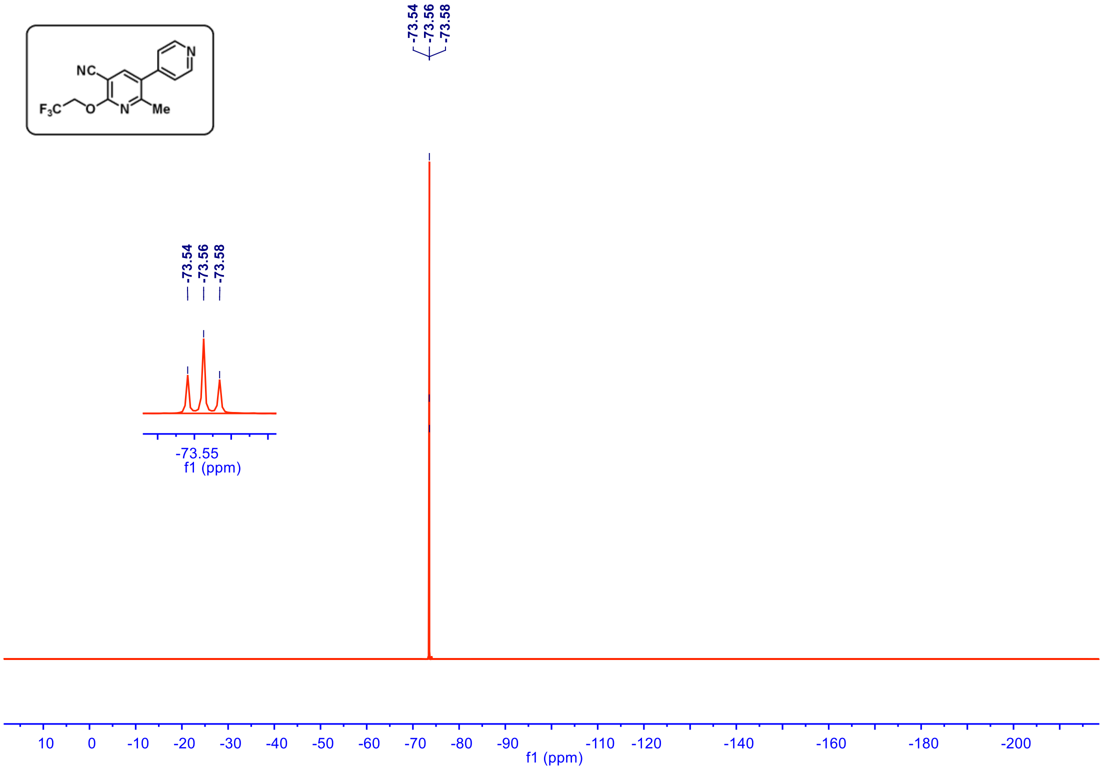

Supplementary Figure 324. ^{19}F NMR spectrum of compound **18** (376 MHz, $CDCl_3$)

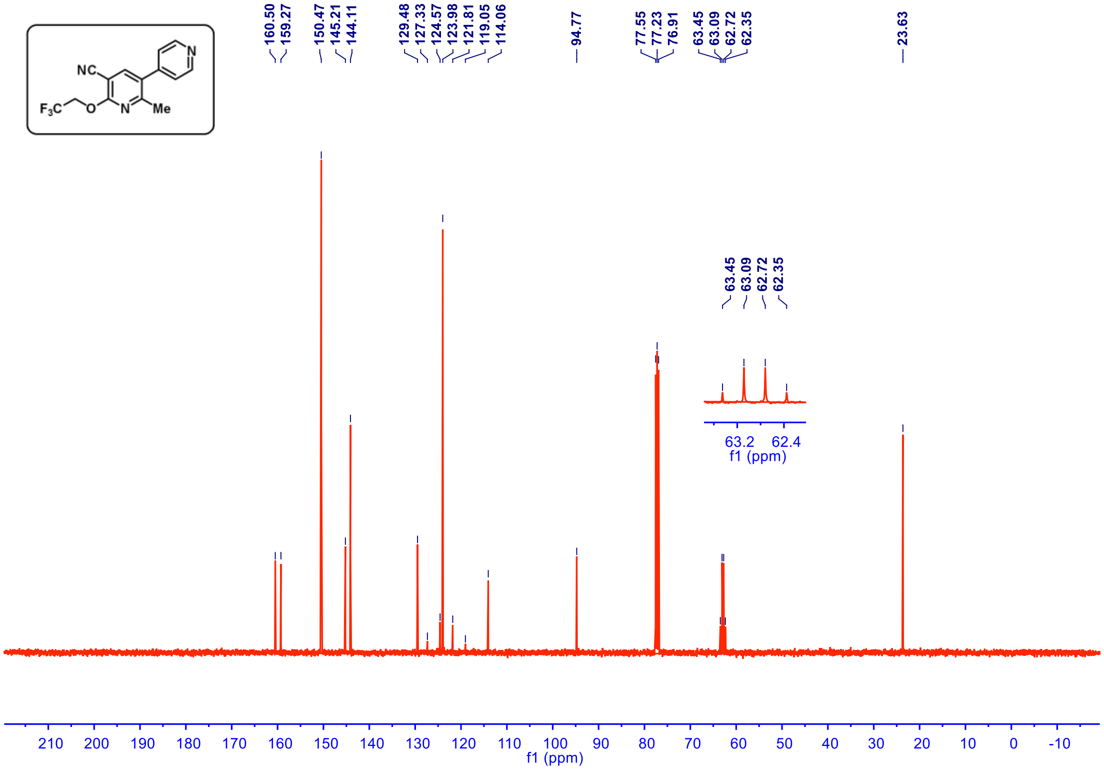

Supplementary Figure 325. ^{13}C NMR spectrum of compound **18** (101 MHz, $CDCl_3$)

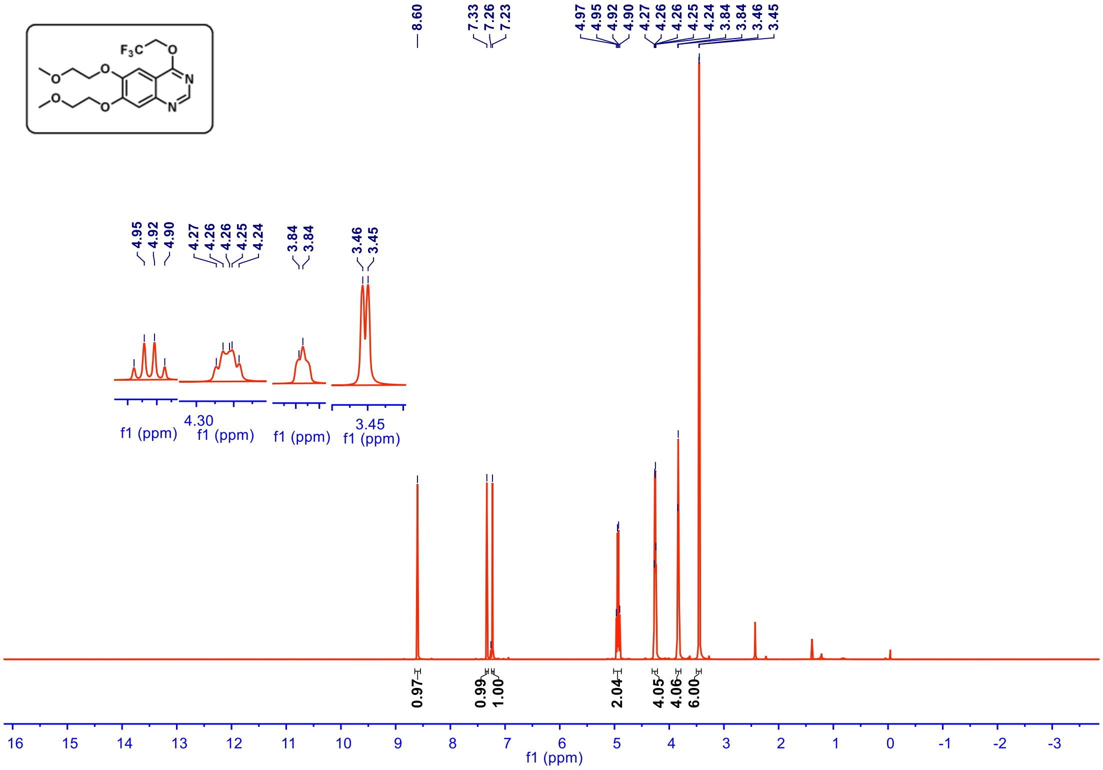

Supplementary Figure 326. ^{1}H NMR spectrum of compound **19** (400 MHz, $CDCl_3$)

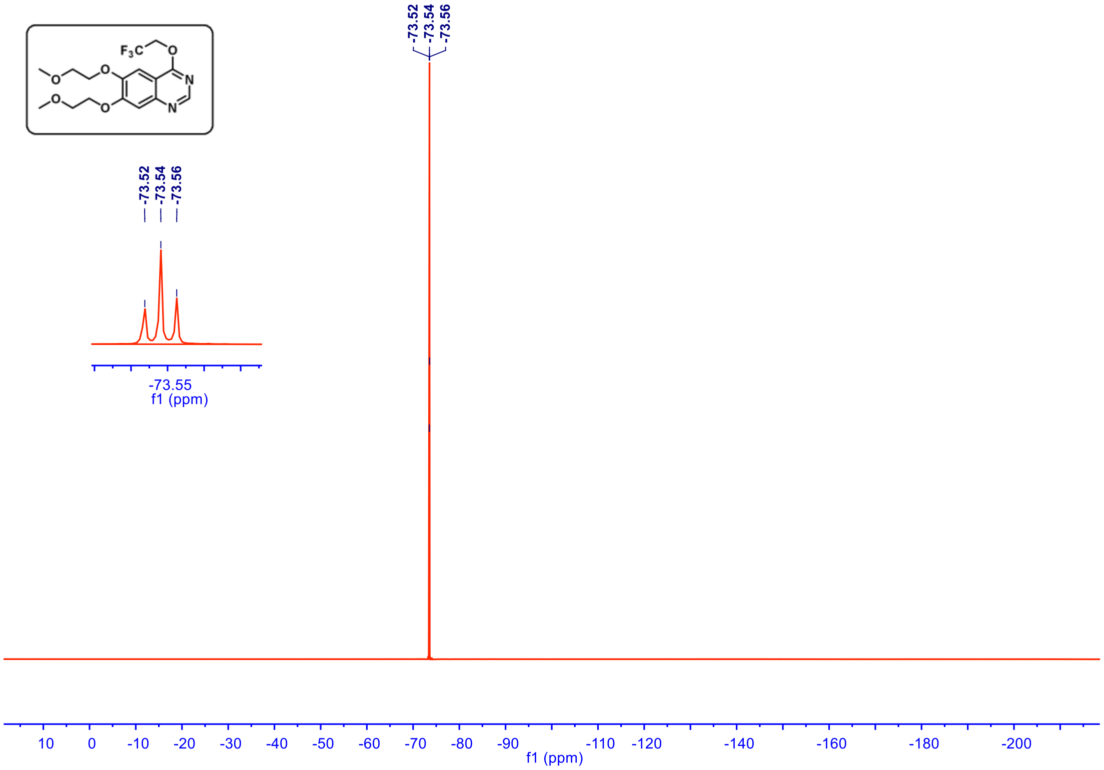

Supplementary Figure 327. ^{19}F NMR spectrum of compound **19** (376 MHz, $CDCl_3$)

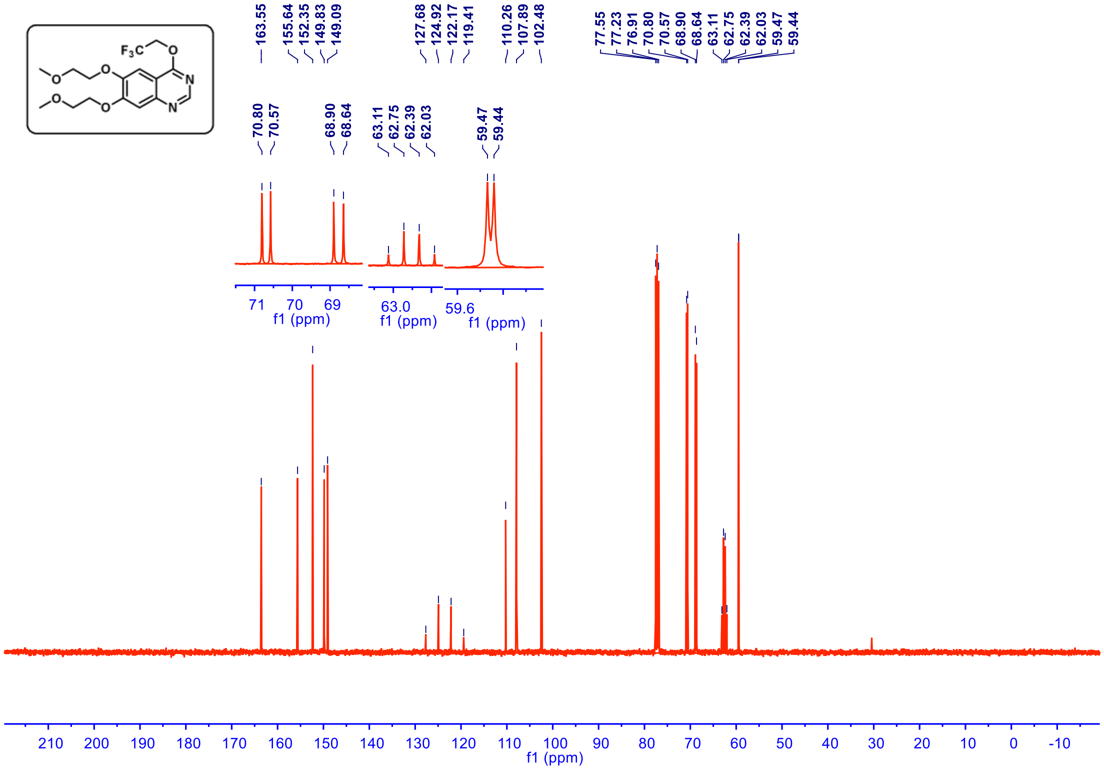

Supplementary Figure 328. ^{13}C NMR spectrum of compound **19** (101 MHz, $CDCl_3$)

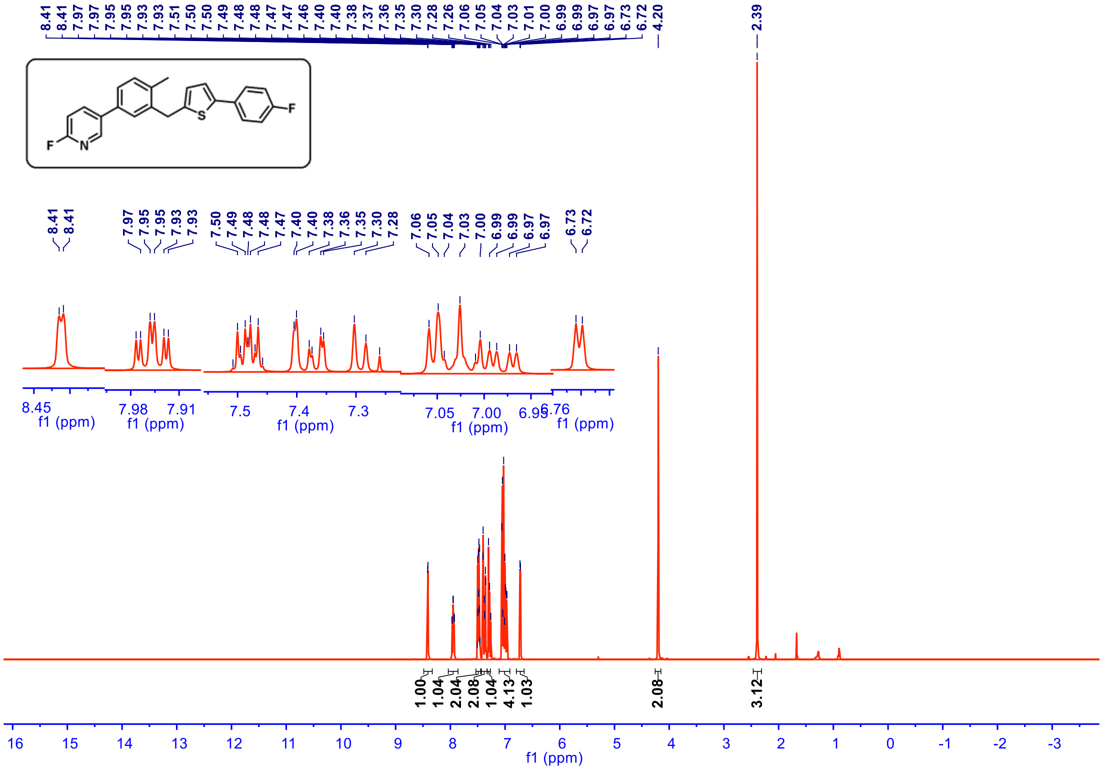

Supplementary Figure 329. ^{1}H NMR spectrum of **2-fluoro-5-(3-((5-(4-fluorophenyl)thiophen-2-yl)methyl)-4-methylphenyl)pyridine** (400 MHz, $CDCl_3$)

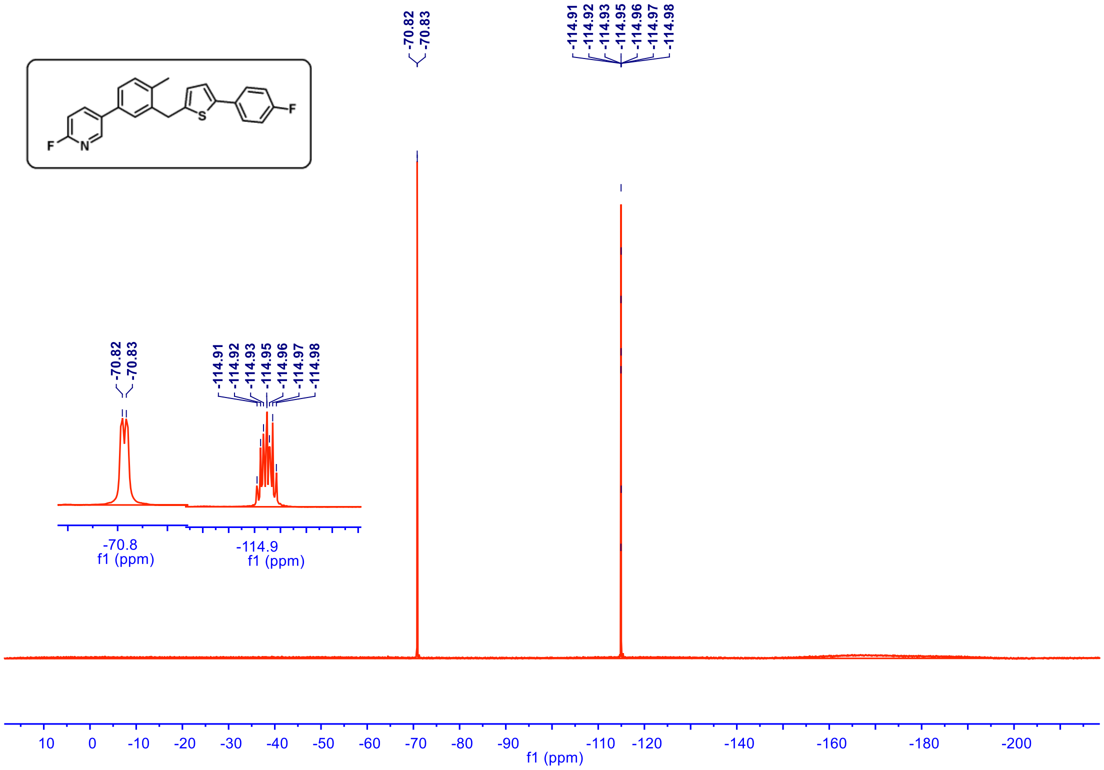

Supplementary Figure 330. ^{19}F NMR spectrum of **2-fluoro-5-(3-((5-(4-fluorophenyl)thiophen-2-yl)methyl)-4-methylphenyl)pyridine** (376 MHz, $CDCl_3$)

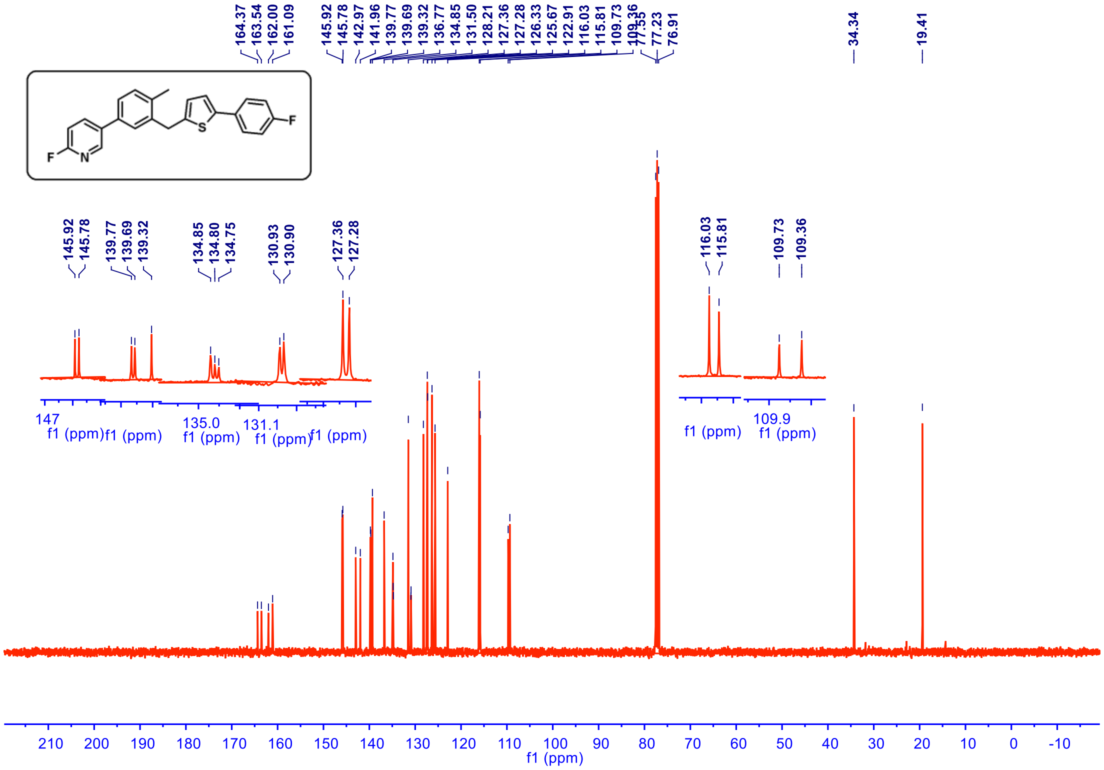

Supplementary Figure 331. ^{13}C NMR spectrum of **2-fluoro-5-(3-((5-(4-fluorophenyl)thiophen-2-yl)methyl)-4-methylphenyl)pyridine** (101 MHz, $CDCl_3$)

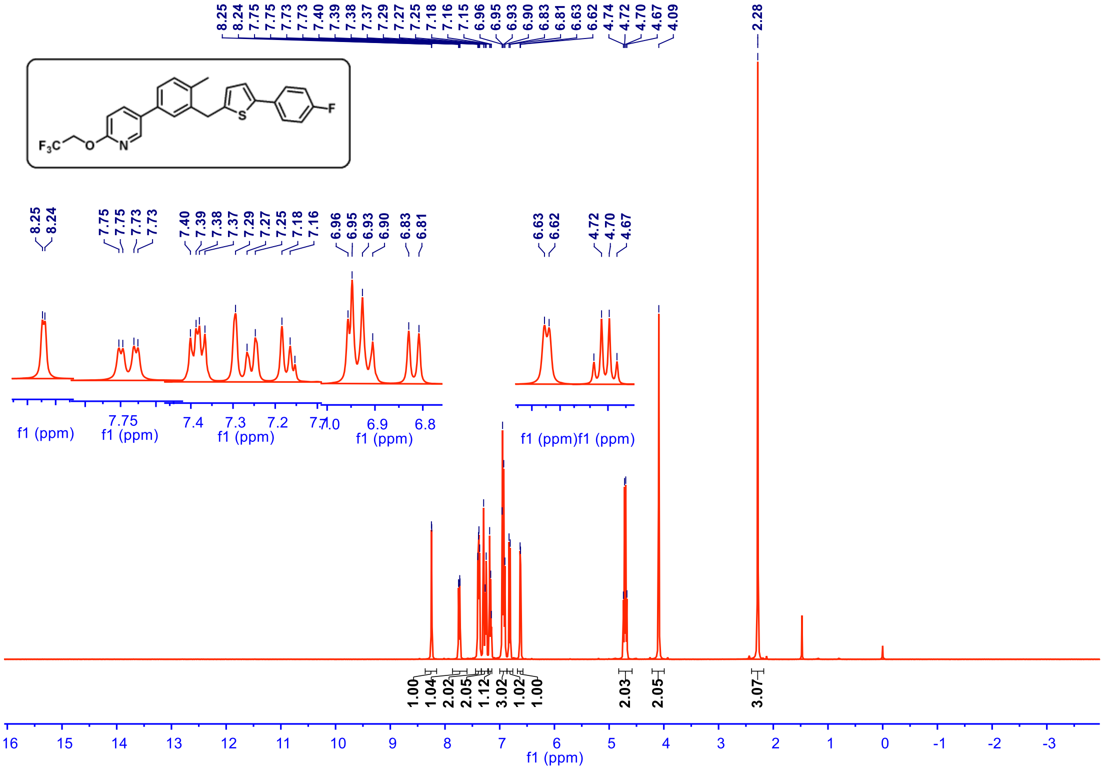

Supplementary Figure 332. ^{1}H NMR spectrum of compound **20** (400 MHz, $CDCl_3$)

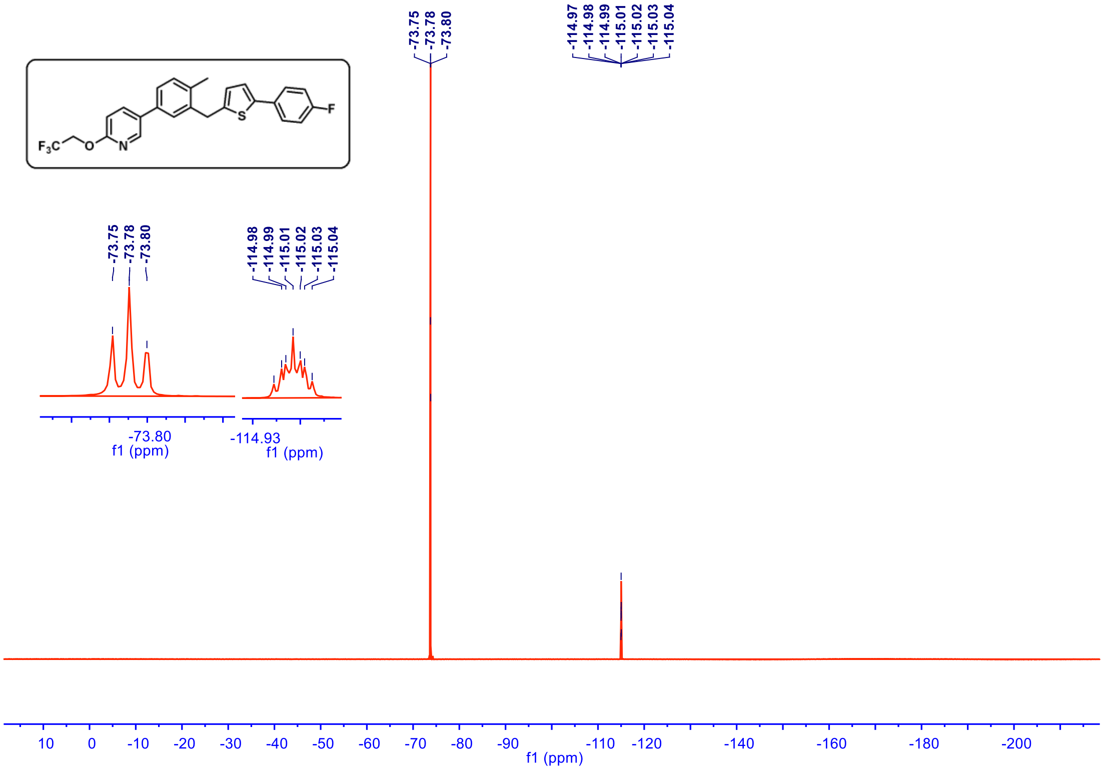

Supplementary Figure 333. ^{19}F NMR spectrum of compound **20** (376 MHz, $CDCl_3$)

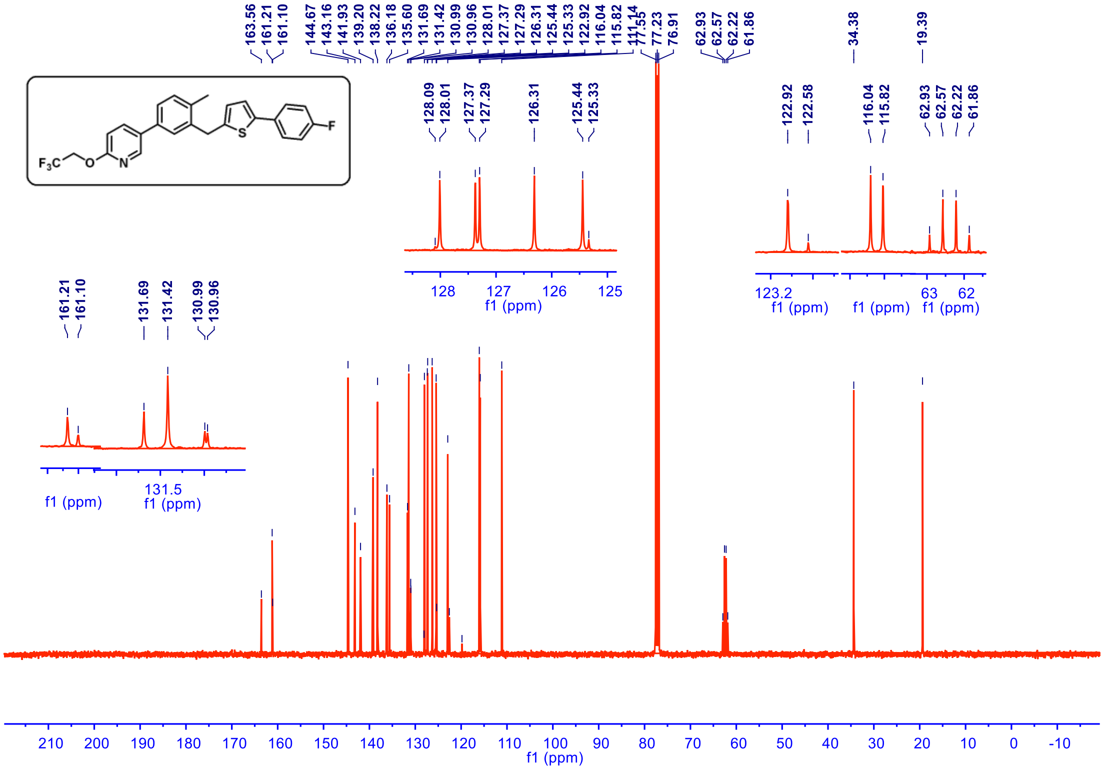

Supplementary Figure 334. ^{13}C NMR spectrum of compound **20** (101 MHz, $CDCl_3$)

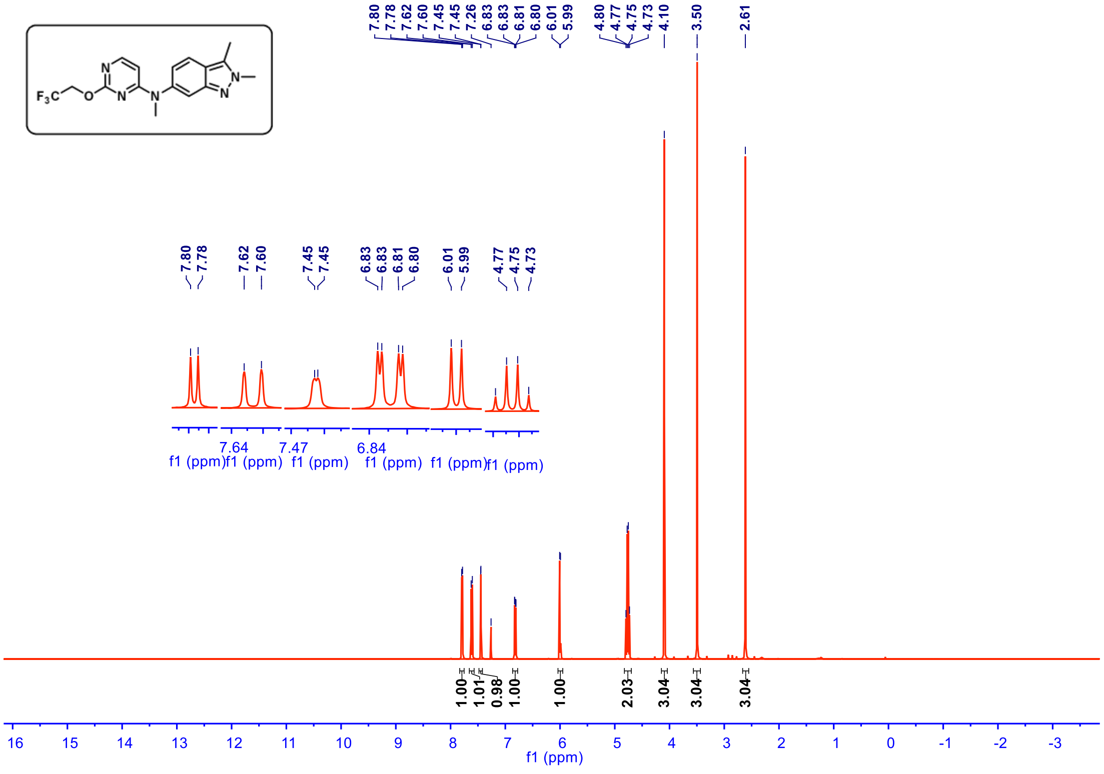

Supplementary Figure 335. ^{1}H NMR spectrum of compound **21** (400 MHz, $CDCl_3$)

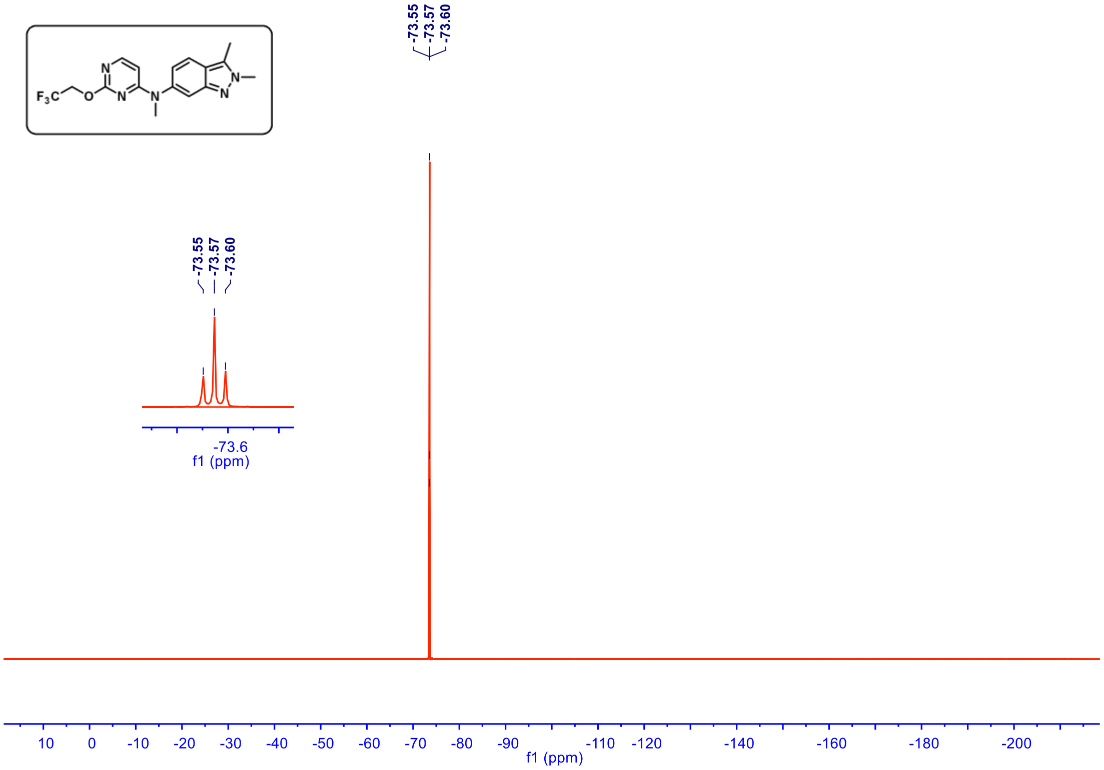

Supplementary Figure 336. ^{19}F NMR spectrum of compound **21** (376 MHz, $CDCl_3$)

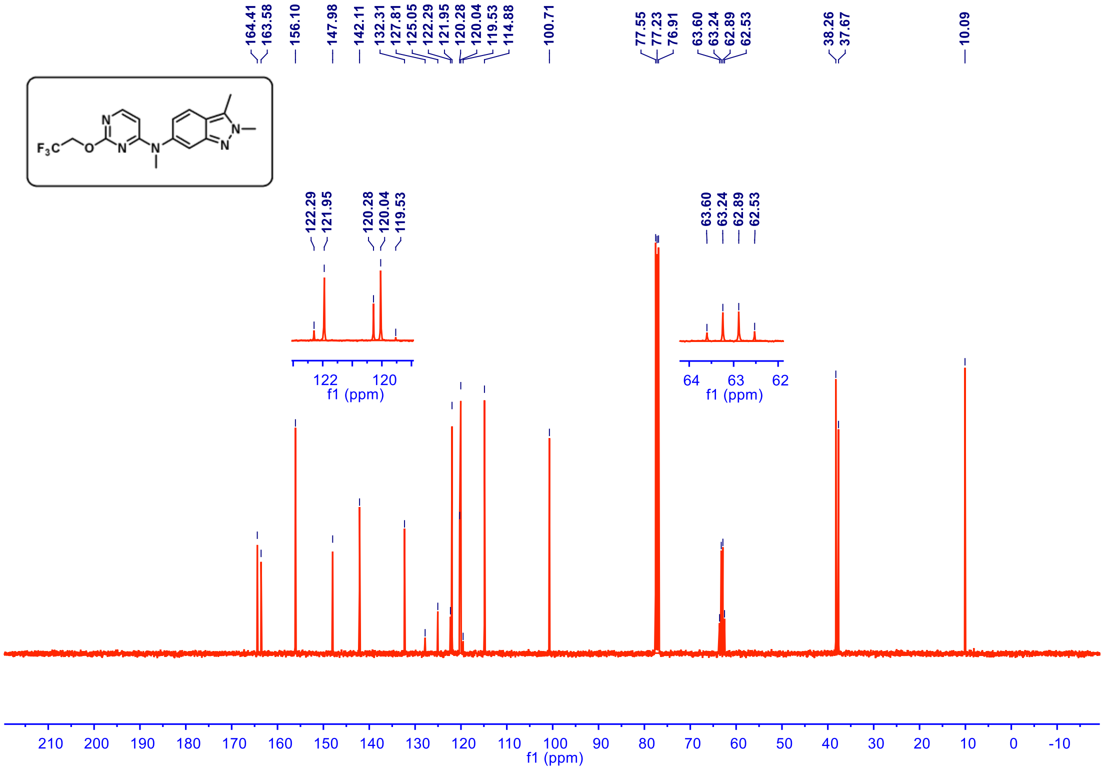

Supplementary Figure 337. ^{13}C NMR spectrum of compound **21** (101 MHz, $CDCl_3$)

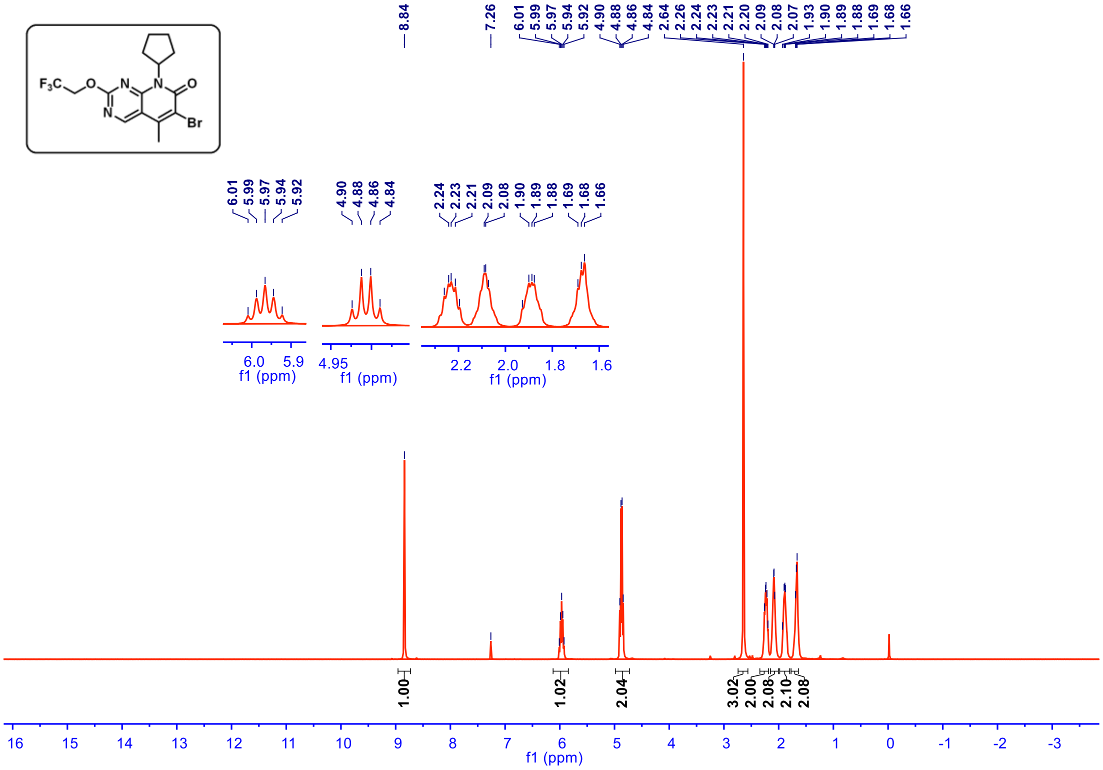

Supplementary Figure 338. ^{1}H NMR spectrum of compound **22** (400 MHz, $CDCl_3$)

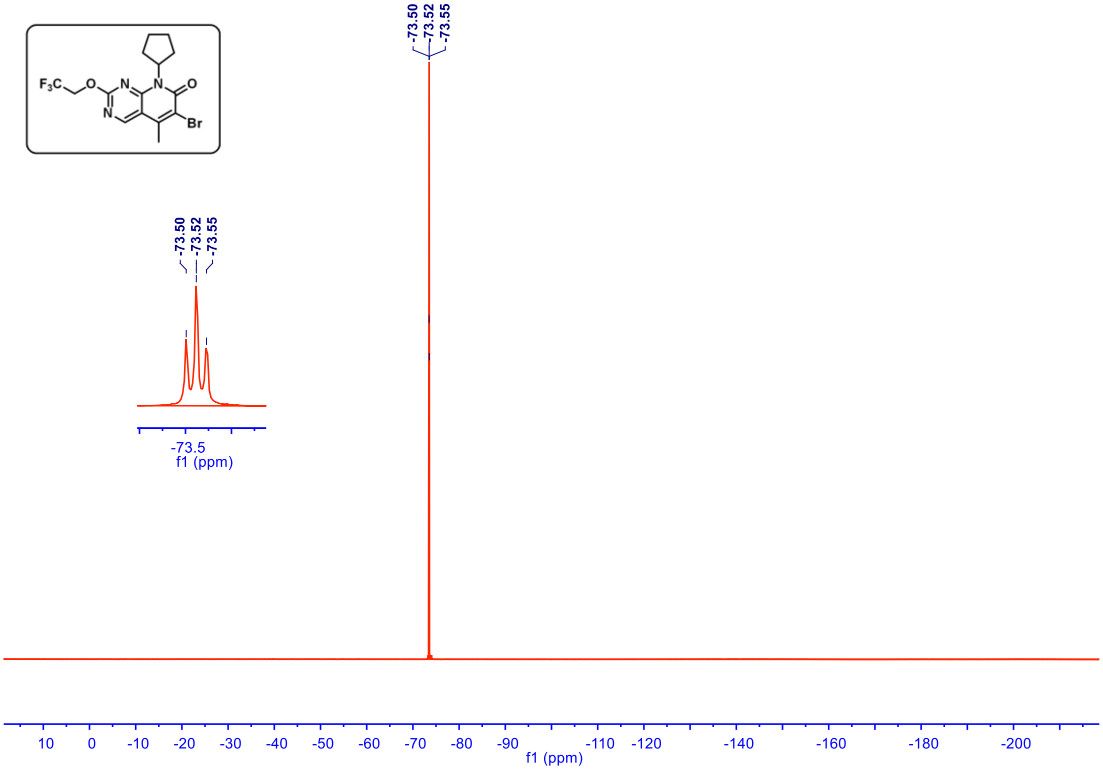

Supplementary Figure 339. ^{19}F NMR spectrum of compound **22** (376 MHz, $CDCl_3$)

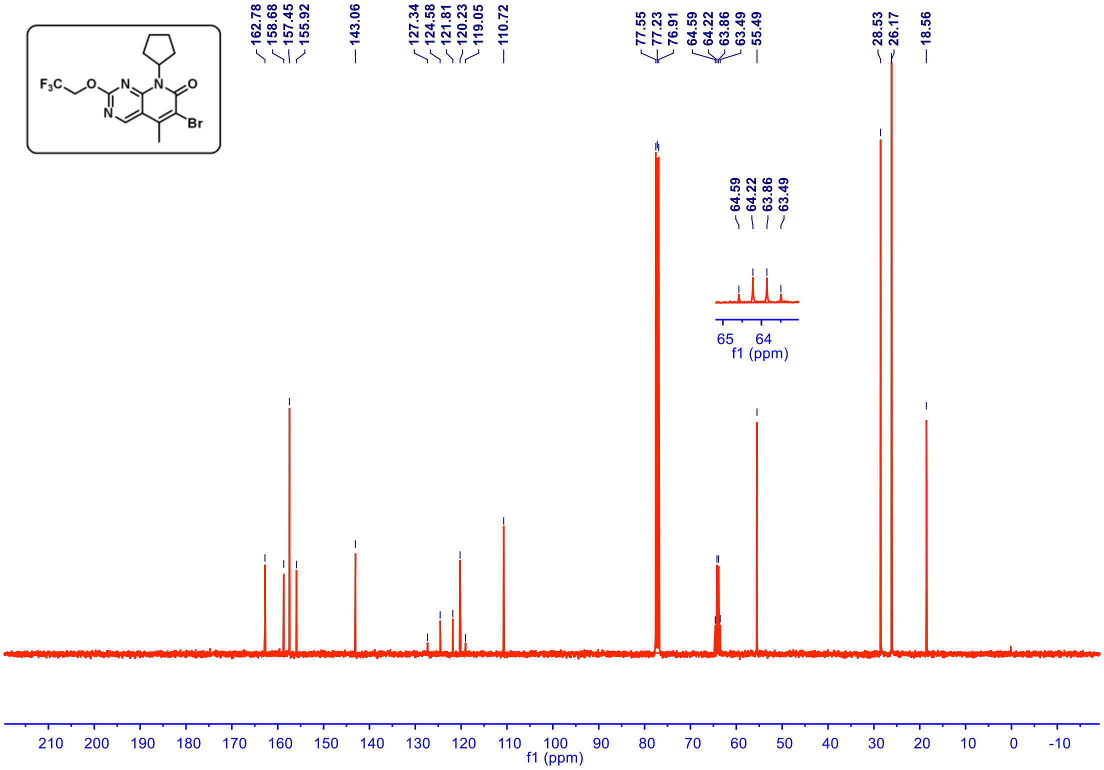

Supplementary Figure 340. ^{13}C NMR spectrum of compound **22** (101 MHz, $CDCl_3$)

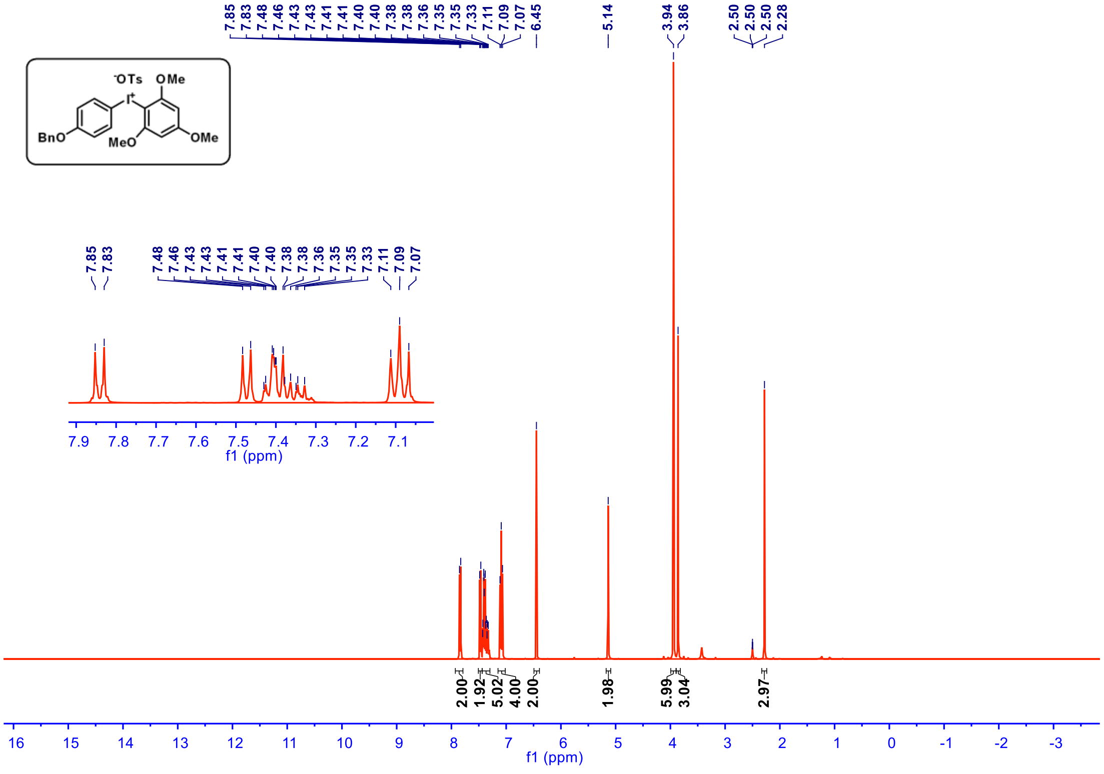

Supplementary Figure 341. ^{1}H NMR spectrum of **(4-benzyloxyphenyl)(2,4,6-trimethoxyphenyl)iodonium tosylate** (400 MHz, $CDCl_3$)

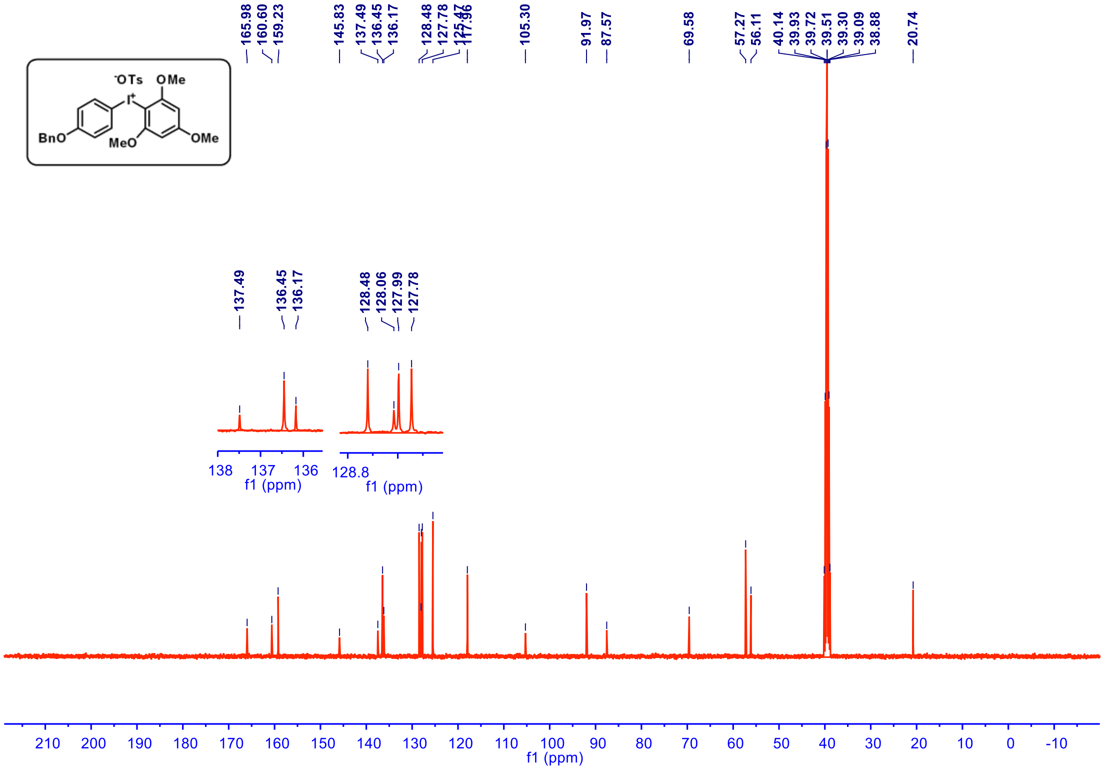

Supplementary Figure 342. ^{13}C NMR spectrum of **(4-benzyloxyphenyl)(2,4,6-trimethoxyphenyl)iodonium tosylate** (101 MHz, $CDCl_3$)

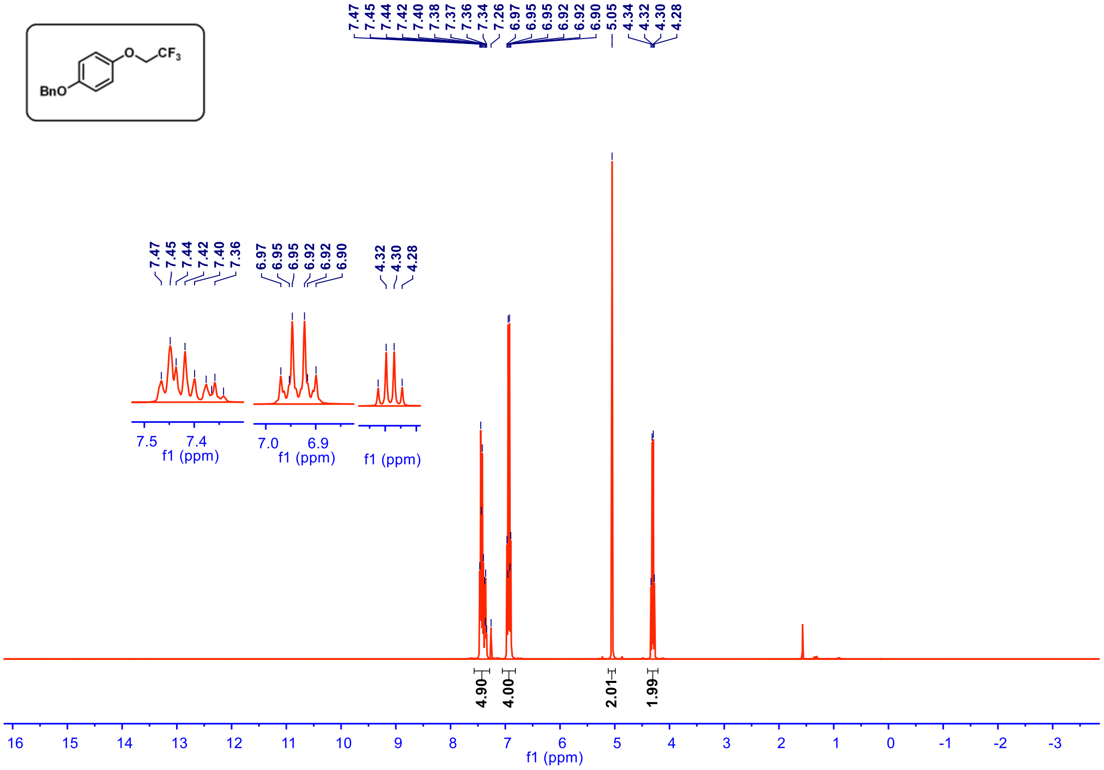

Supplementary Figure 343. ^{1}H NMR spectrum of compound **23** (400 MHz, $CDCl_3$)

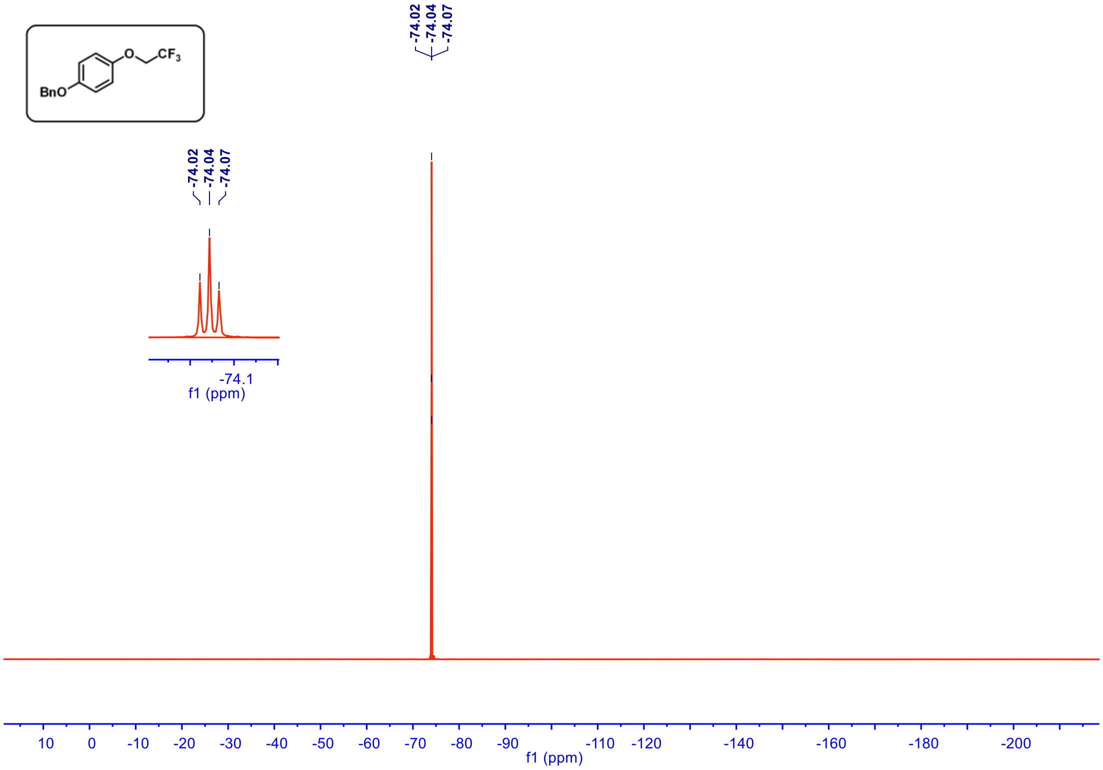

Supplementary Figure 344. ^{19}F NMR spectrum of compound **23** (376 MHz, $CDCl_3$)

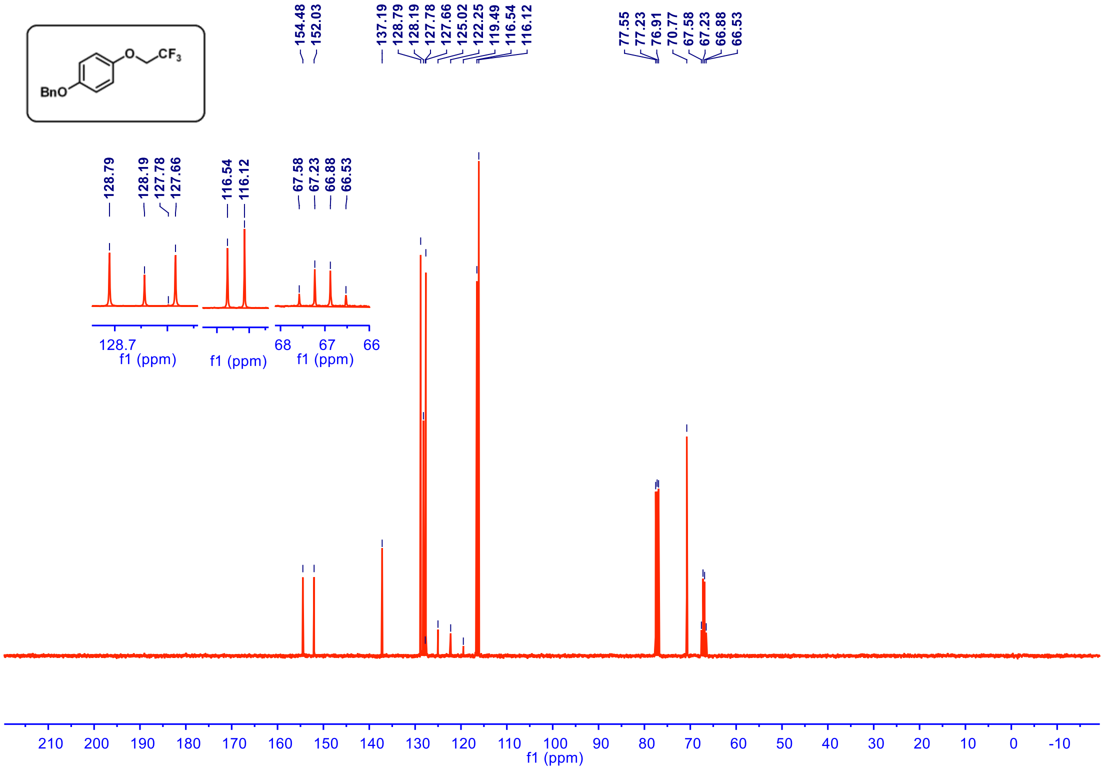

Supplementary Figure 345. ^{13}C NMR spectrum of compound **23** (101 MHz, $CDCl_3$)

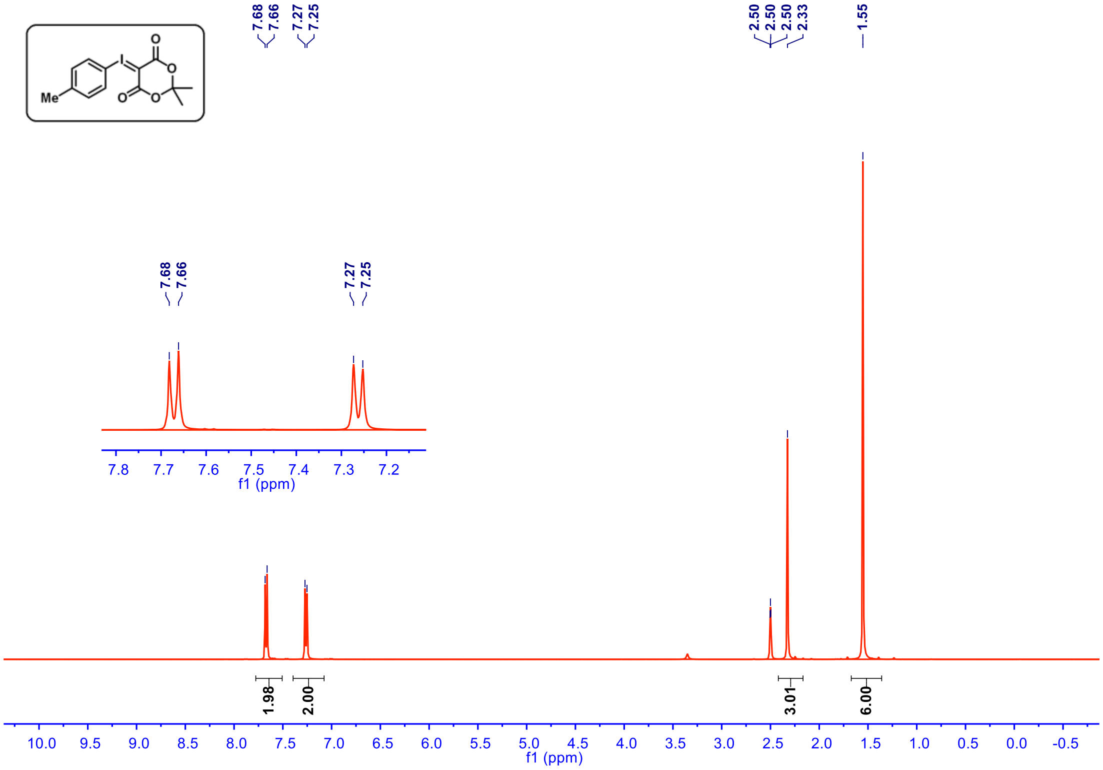

Supplementary Figure 346. ^{1}H NMR spectrum of **(*p*-tolyl)iodonium-(2,2-dimethyl-1,3-dioxane-4,6-dione)ylide** (400 MHz, $CDCl_3$)

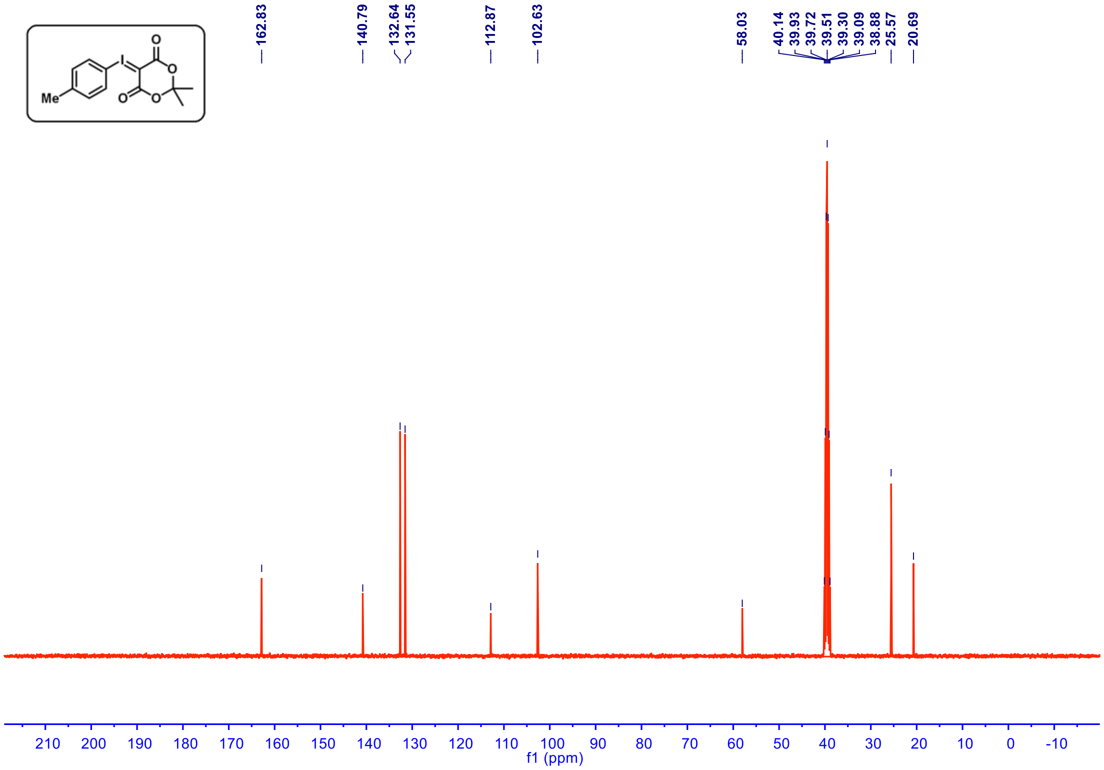

Supplementary Figure 347. ^{13}C NMR spectrum of **(*p*-tolyl)iodonium-(2,2-dimethyl-1,3-dioxane-4,6-dione)ylide** (101 MHz, $CDCl_3$)

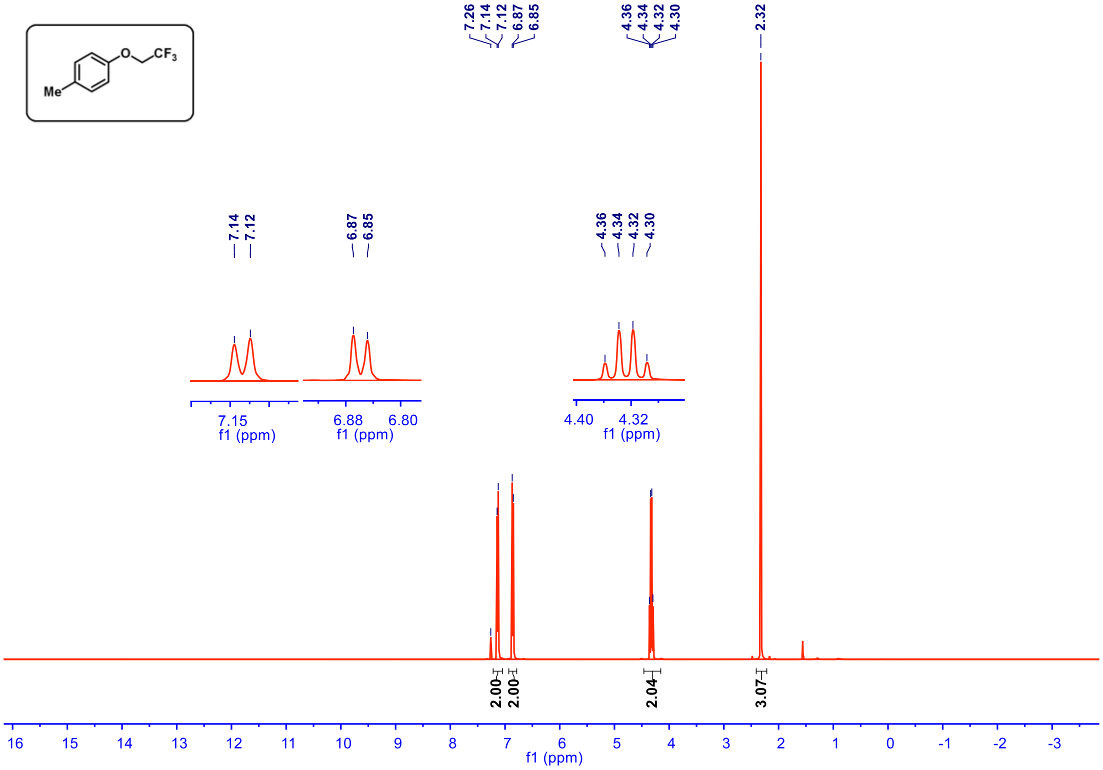

Supplementary Figure 348. ^{1}H NMR spectrum of compound **24** (400 MHz, $CDCl_3$)

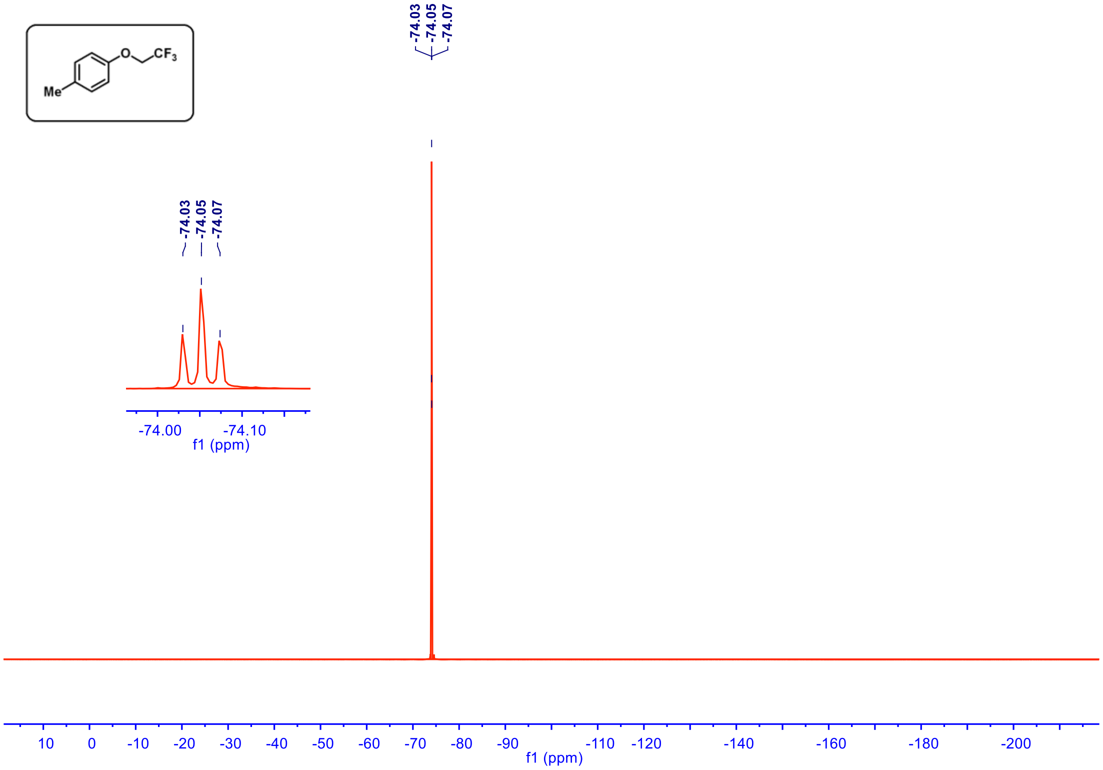

Supplementary Figure 349. ^{19}F NMR spectrum of compound **24** (376 MHz, $CDCl_3$)

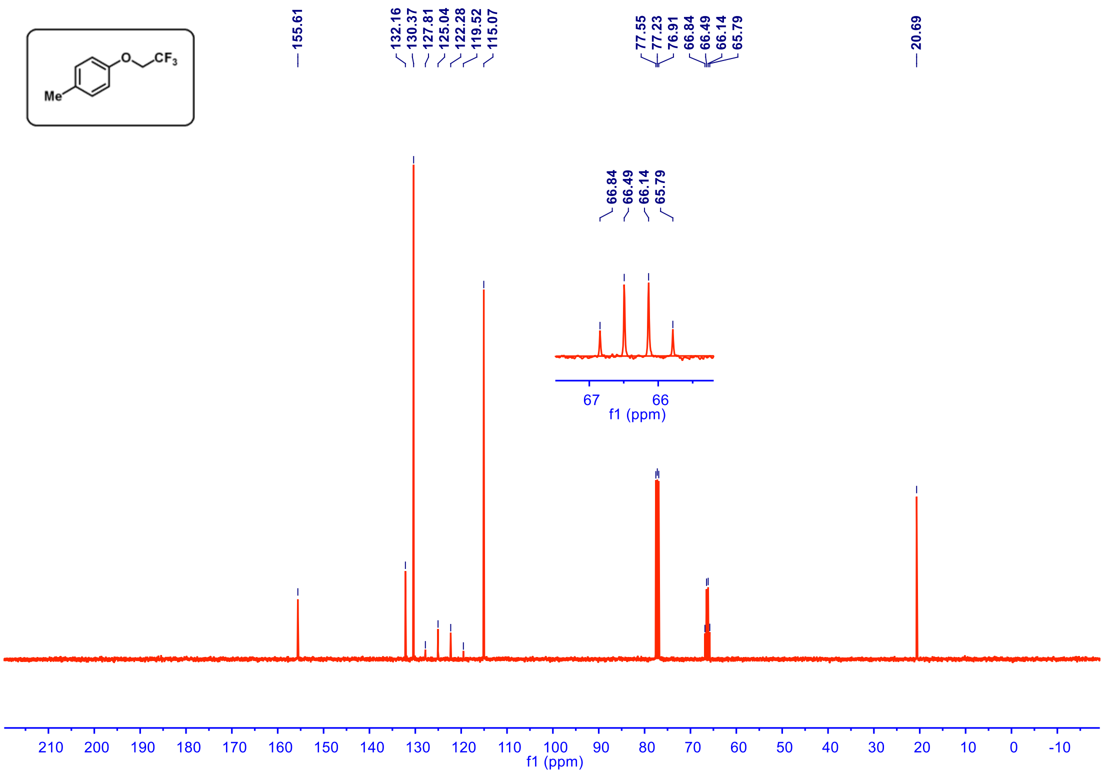

Supplementary Figure 350. ^{13}C NMR spectrum of compound **24** (101 MHz, $CDCl_3$)

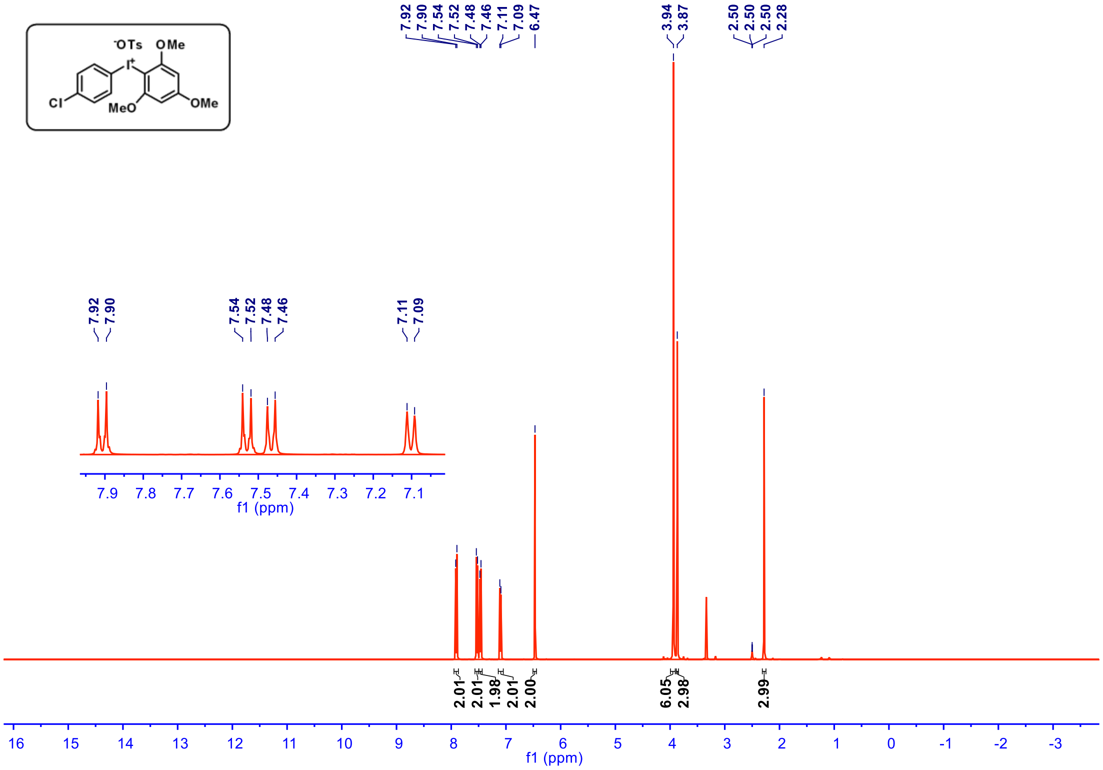

Supplementary Figure 351. ^{1}H NMR spectrum of **(4-chlorophenyl)(2,4,6-trimethoxyphenyl)iodonium tosylate** (400 MHz, $CDCl_3$)

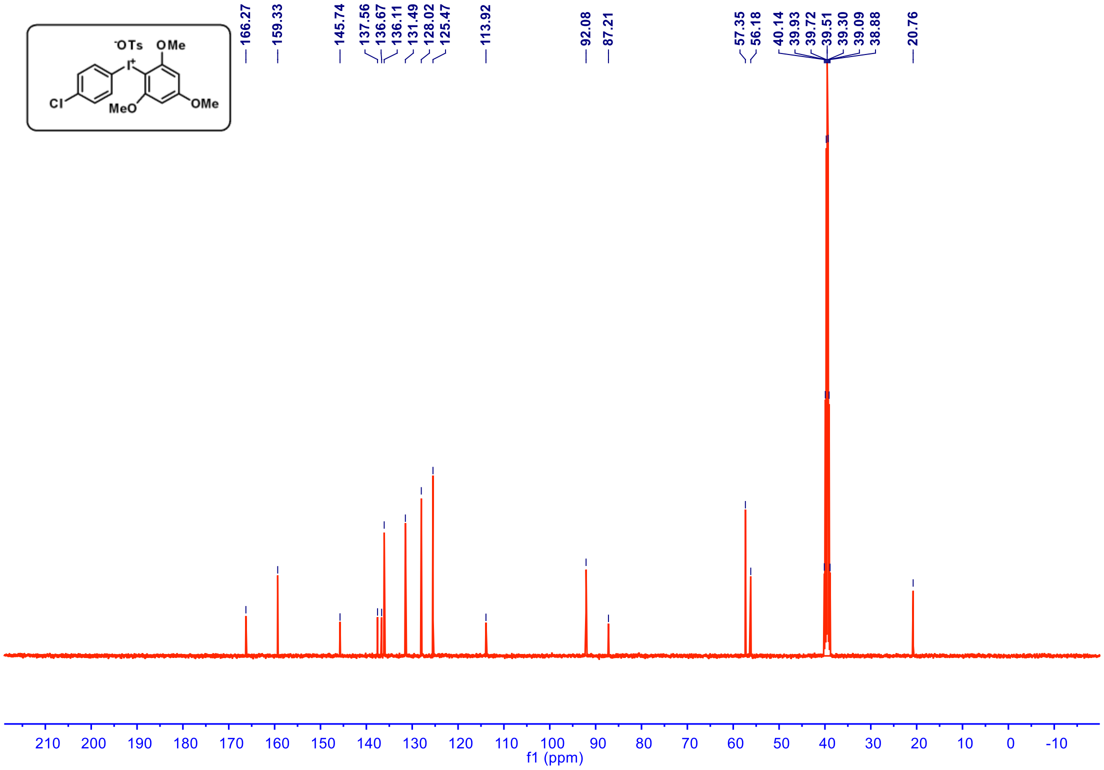

Supplementary Figure 352. ^{13}C NMR spectrum of **(4-chlorophenyl)(2,4,6-trimethoxyphenyl)iodonium tosylate** (101 MHz, $CDCl_3$)

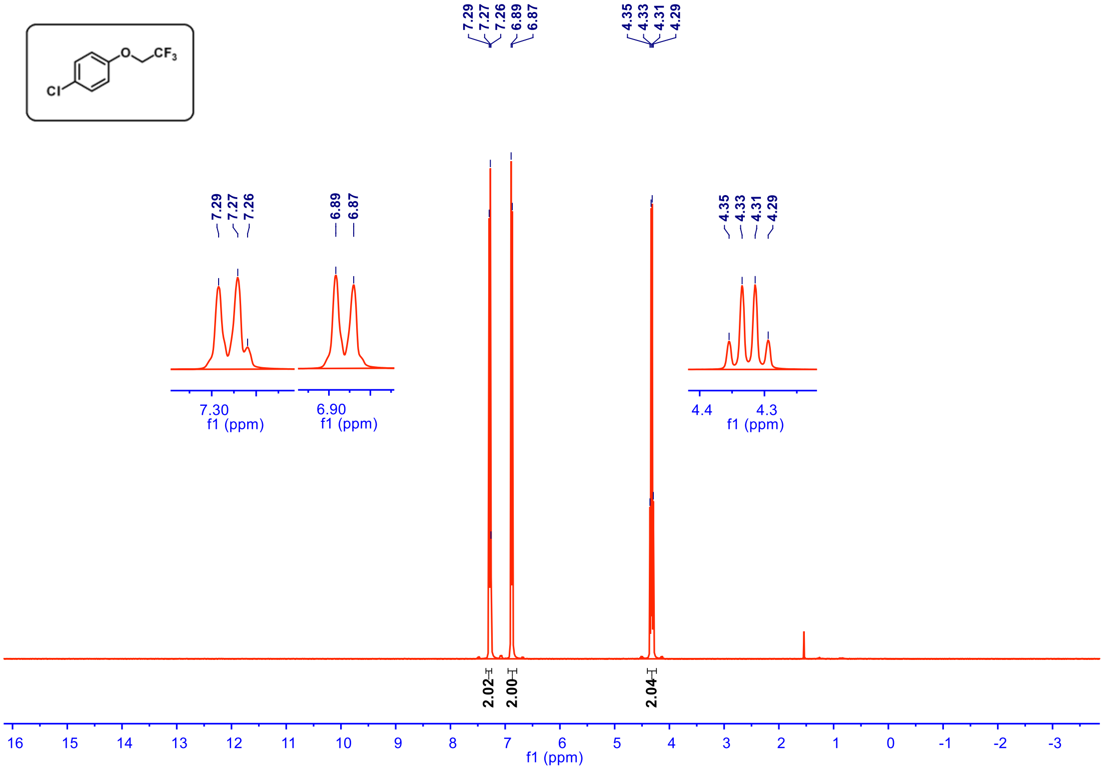

Supplementary Figure 353. ^{1}H NMR spectrum of compound **25** (400 MHz, $CDCl_3$)

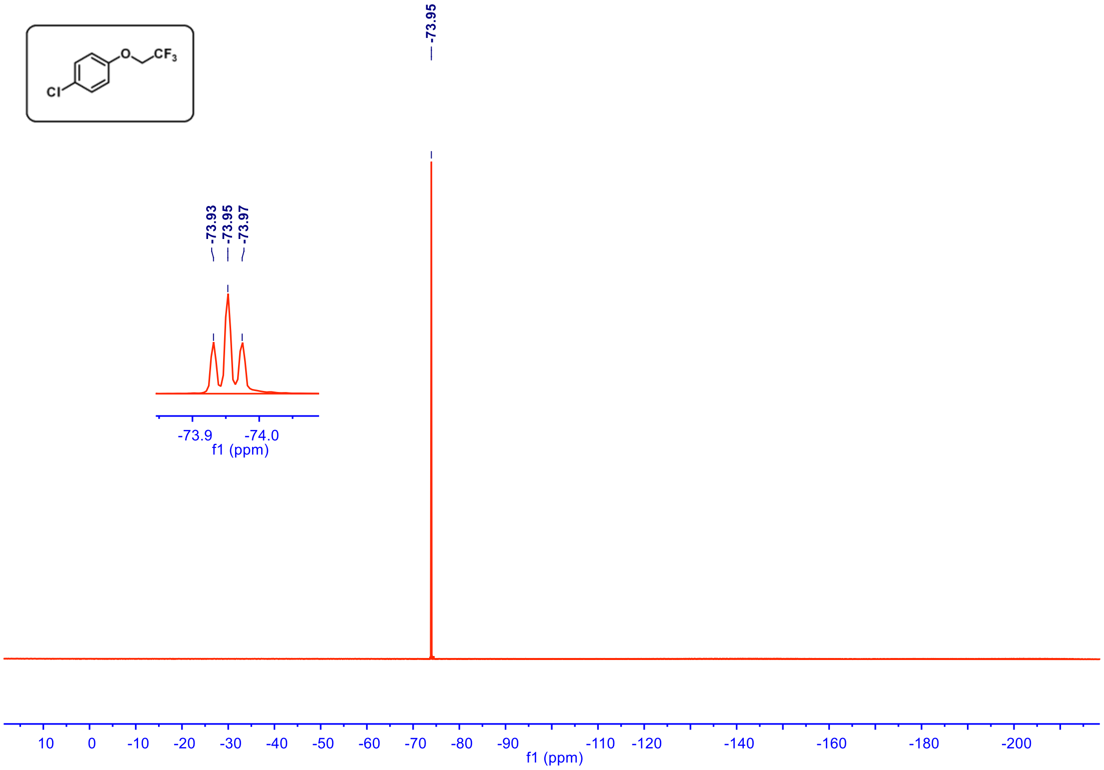

Supplementary Figure 354. ^{19}F NMR spectrum of compound **25** (376 MHz, $CDCl_3$)

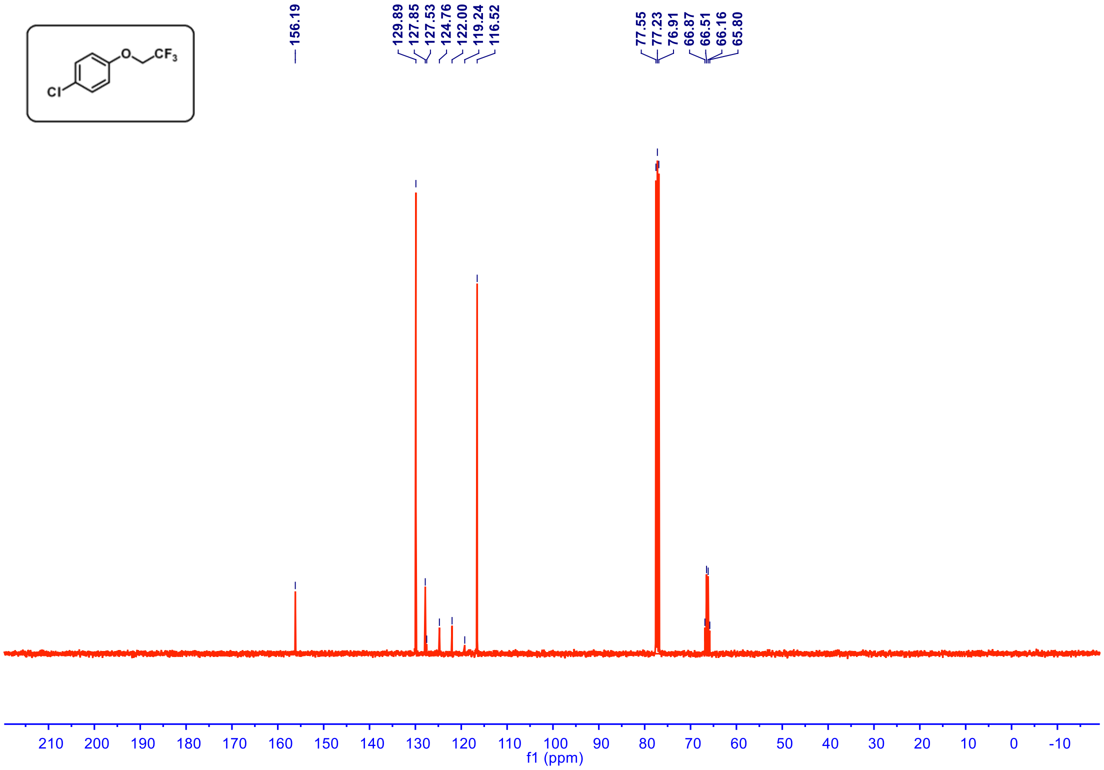

Supplementary Figure 355. ^{13}C NMR spectrum of compound **25** (101 MHz, $CDCl_3$)

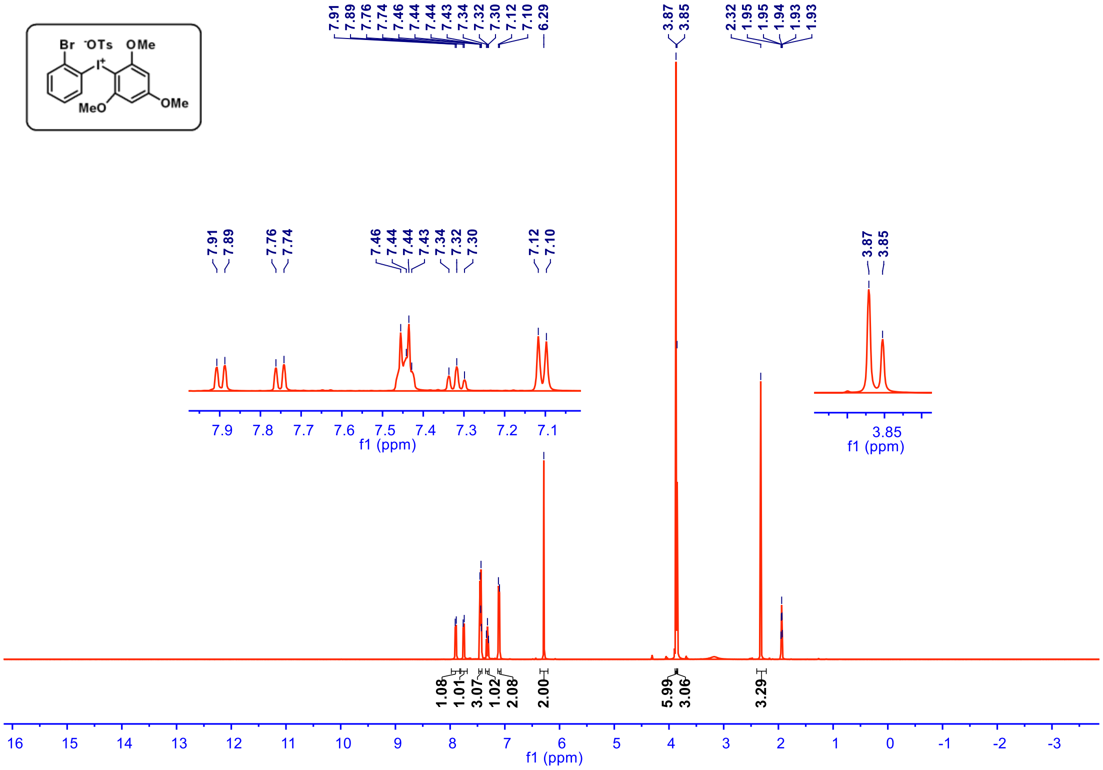

Supplementary Figure 356. ^{1}H NMR spectrum of **(2-bromophenyl)(2,4,6-trimethoxyphenyl)iodonium tosylate** (400 MHz, $CDCl_3$)

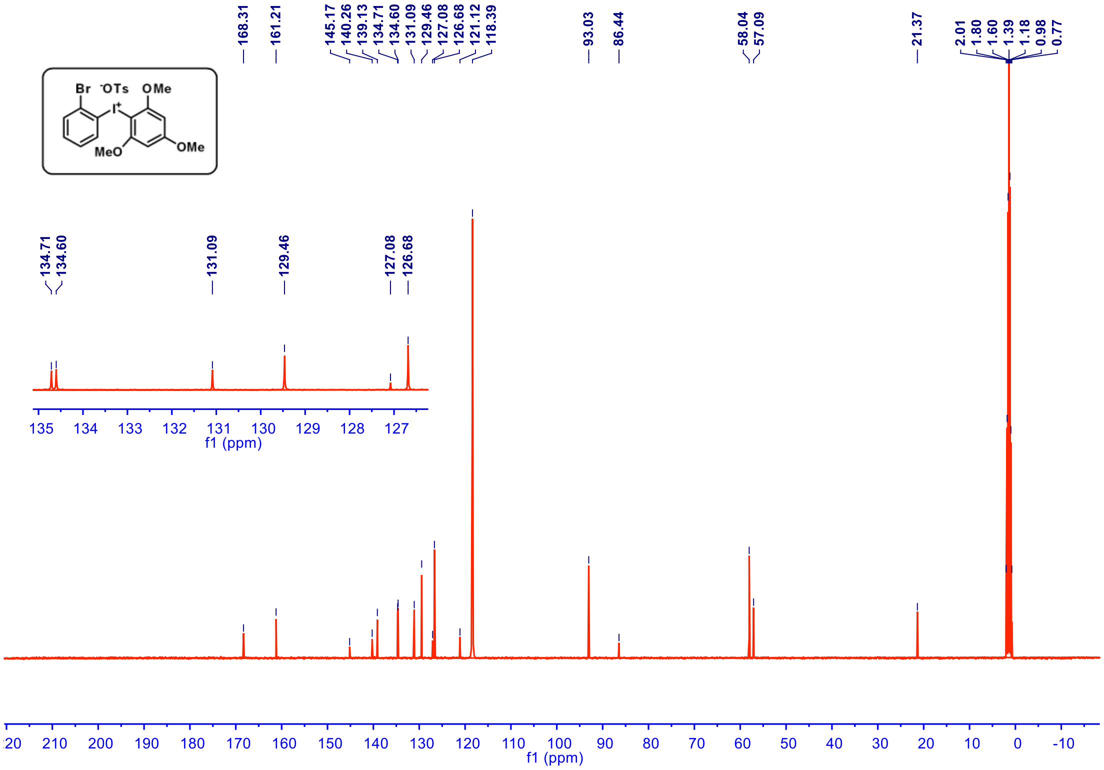

Supplementary Figure 357. ^{13}C NMR spectrum of **(2-bromophenyl)(2,4,6-trimethoxyphenyl)iodonium tosylate** (101 MHz, $CDCl_3$)

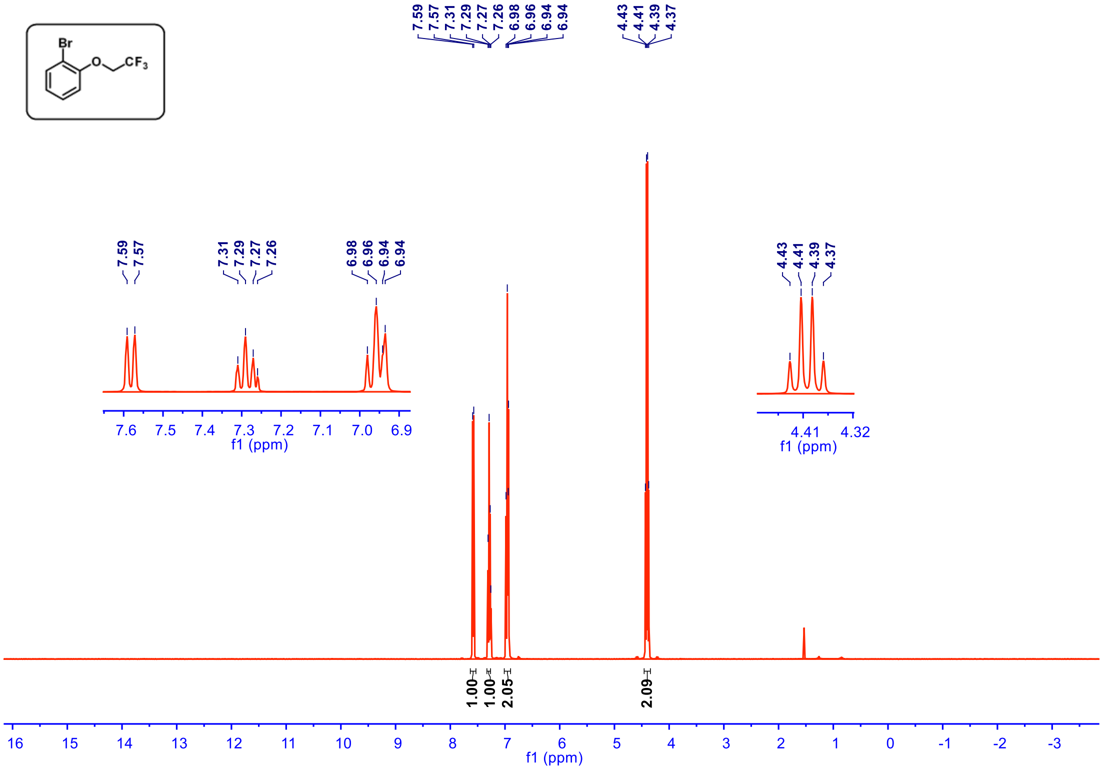

Supplementary Figure 358. ^{1}H NMR spectrum of compound **26** (400 MHz, $CDCl_3$)

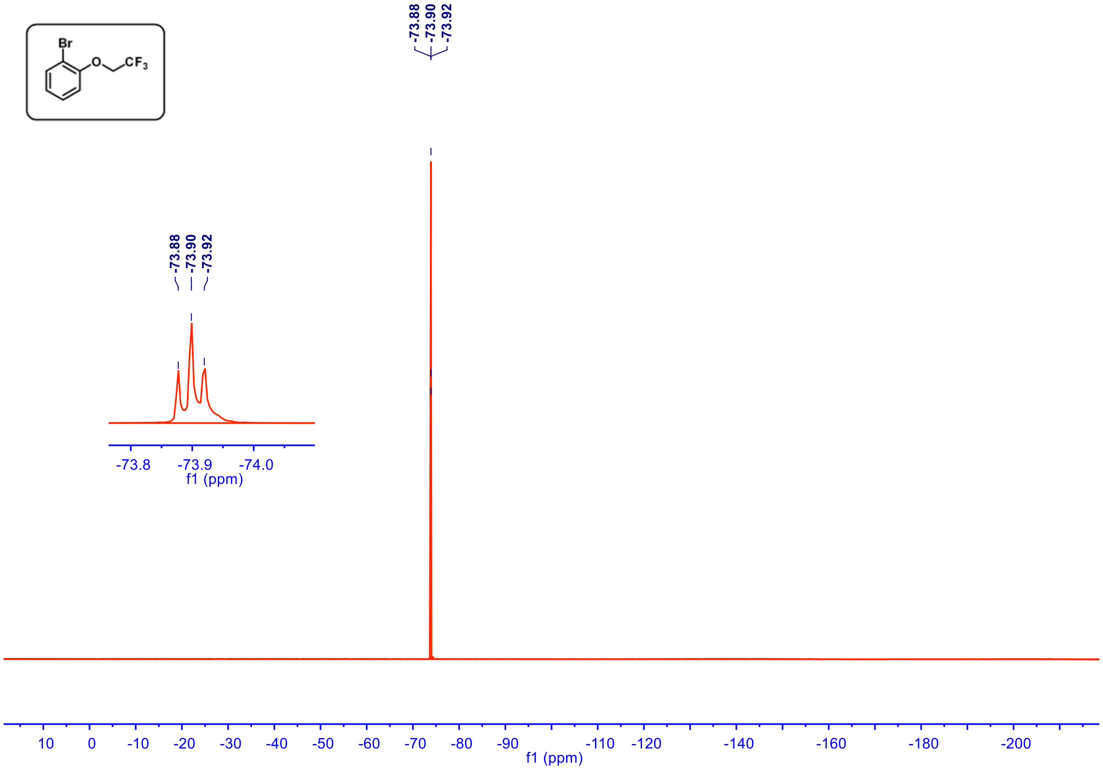

Supplementary Figure 359. ^{19}F NMR spectrum of compound **26** (376 MHz, $CDCl_3$)

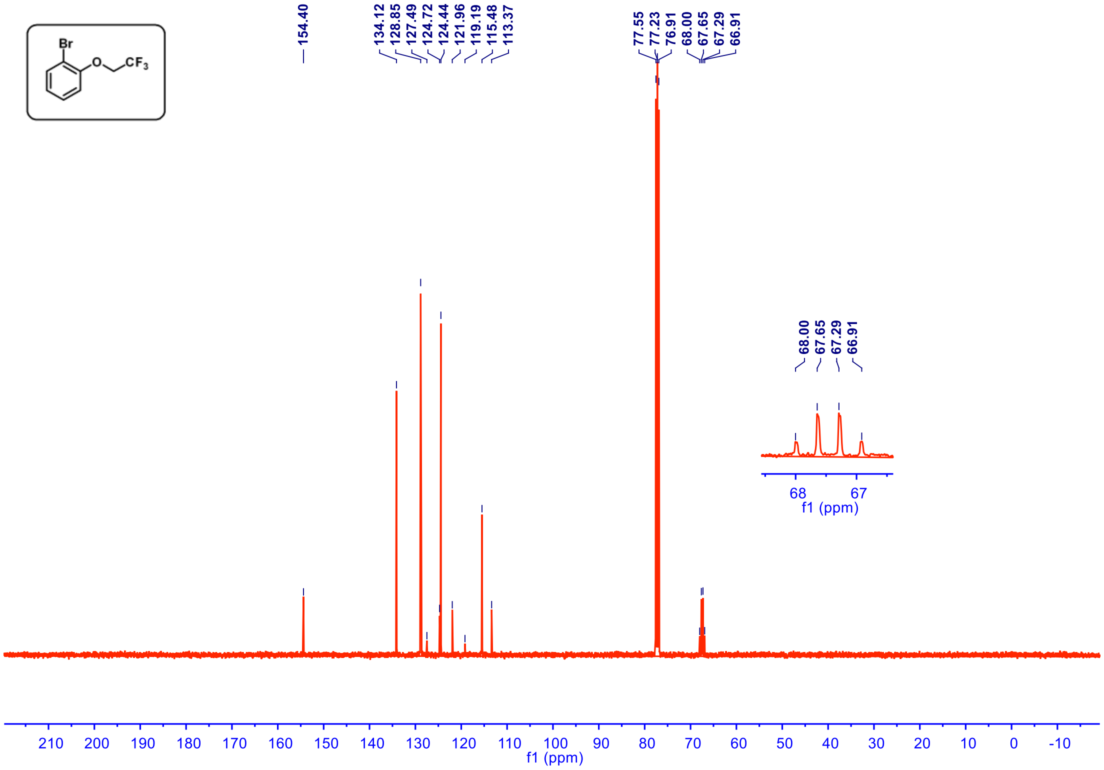

Supplementary Figure 360. ^{13}C NMR spectrum of compound **26** (101 MHz, $CDCl_3$)

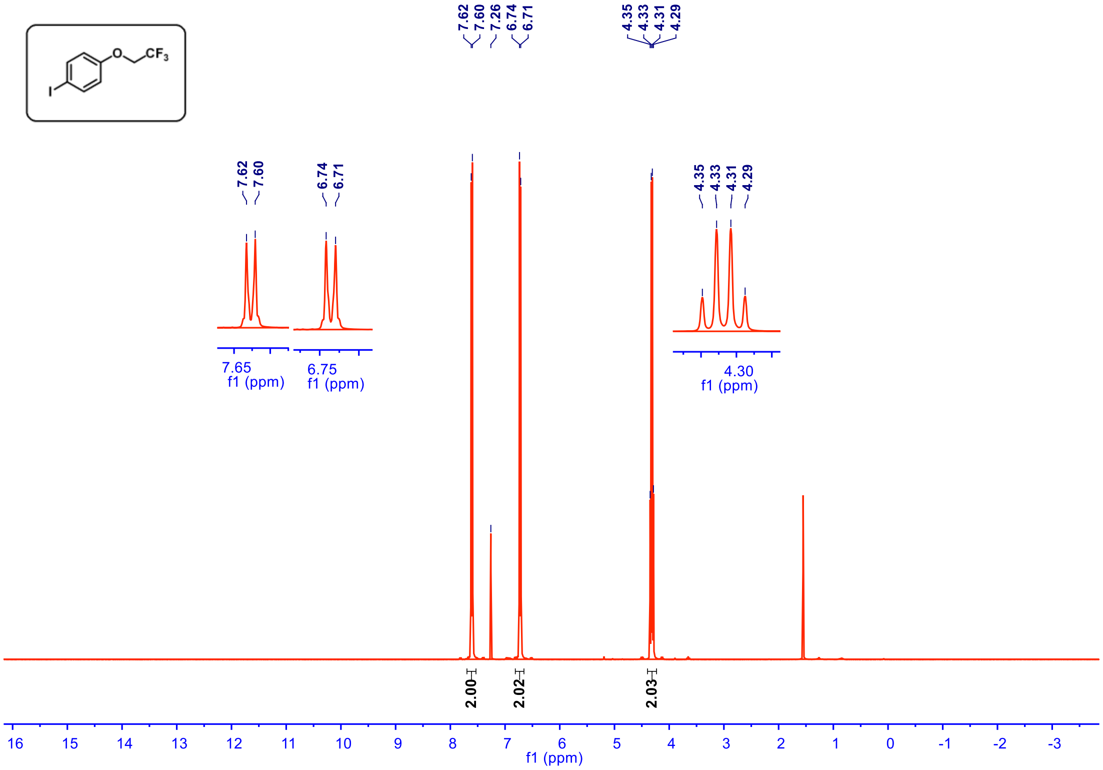

Supplementary Figure 361. ^{1}H NMR spectrum of compound **27** (400 MHz, $CDCl_3$)

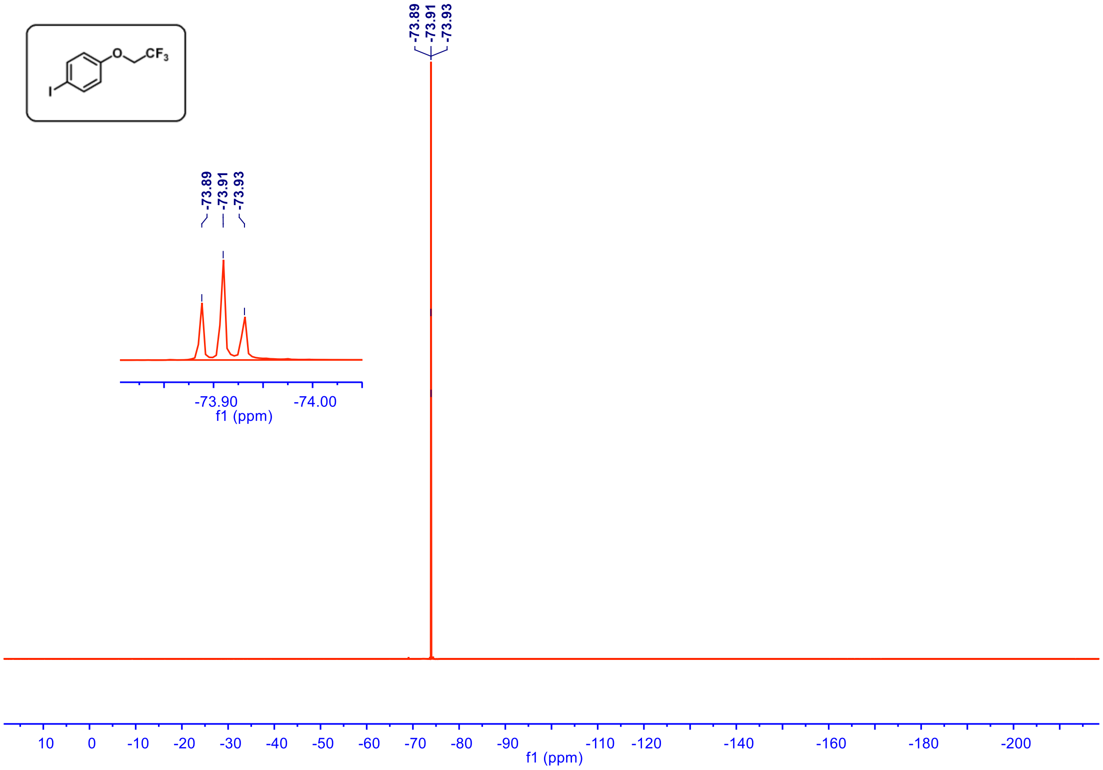

Supplementary Figure 362. ^{19}F NMR spectrum of compound **27** (376 MHz, $CDCl_3$)

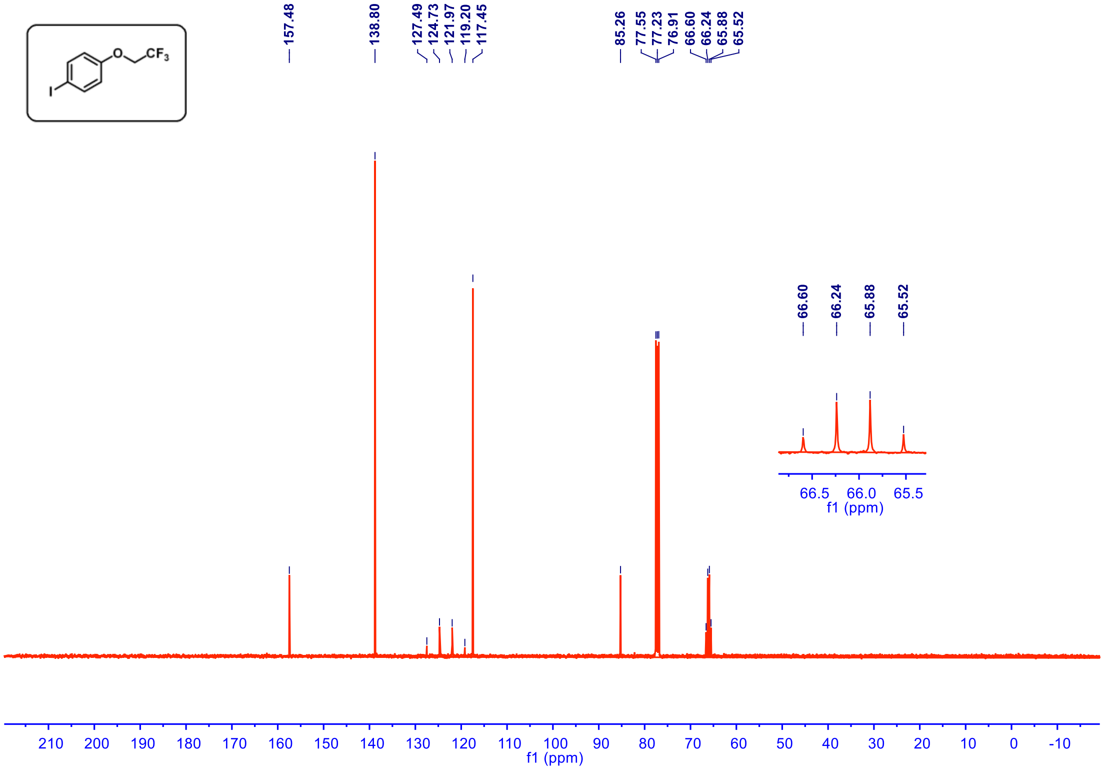

Supplementary Figure 363. ^{13}C NMR spectrum of compound **27** (101 MHz, $CDCl_3$)

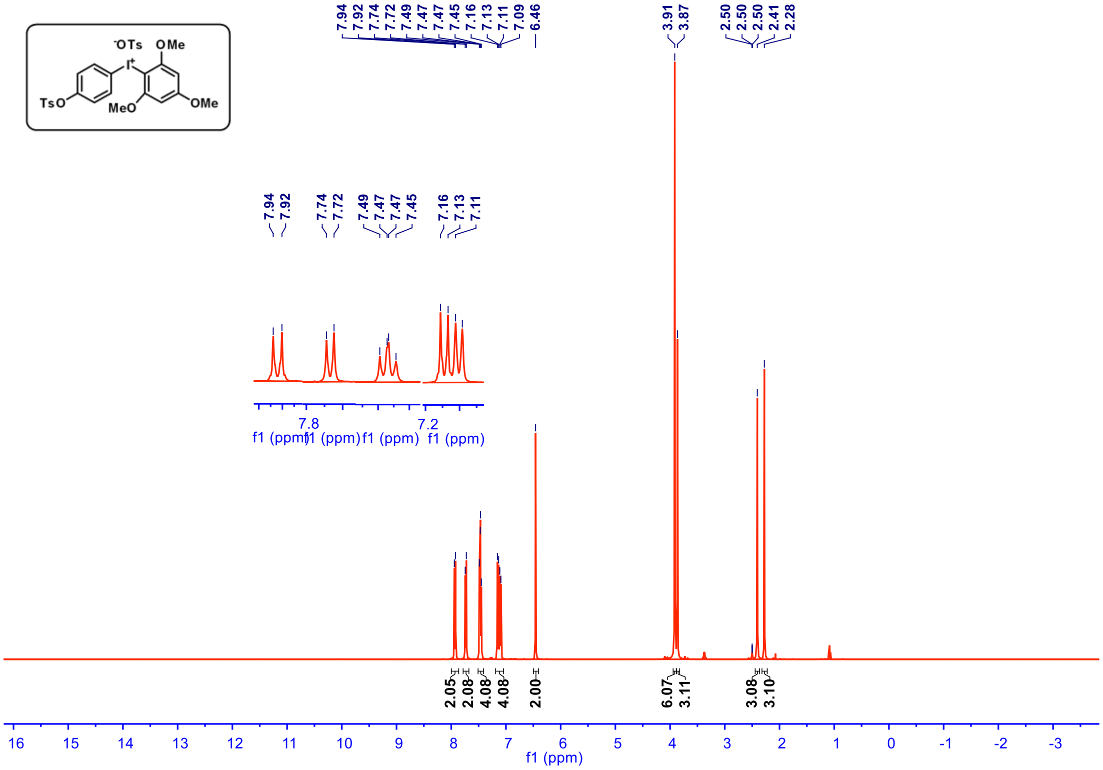

Supplementary Figure 364. ^{1}H NMR spectrum of **(4-methylbenzenesulfonate)(2,4,6-trimethoxyphenyl)iodonium tosylate** (400 MHz, $CDCl_3$)

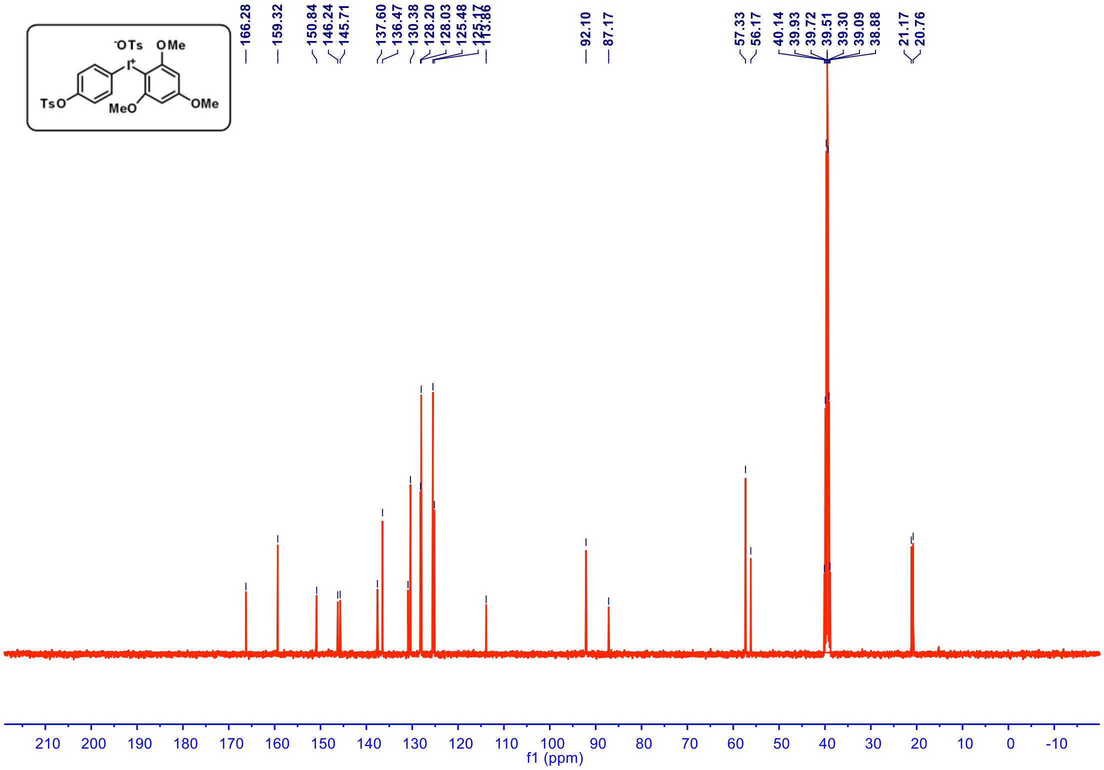

Supplementary Figure 365. ^{13}C NMR spectrum of **(4-methylbenzenesulfonate)(2,4,6-trimethoxyphenyl)iodonium tosylate** (101 MHz, $CDCl_3$)

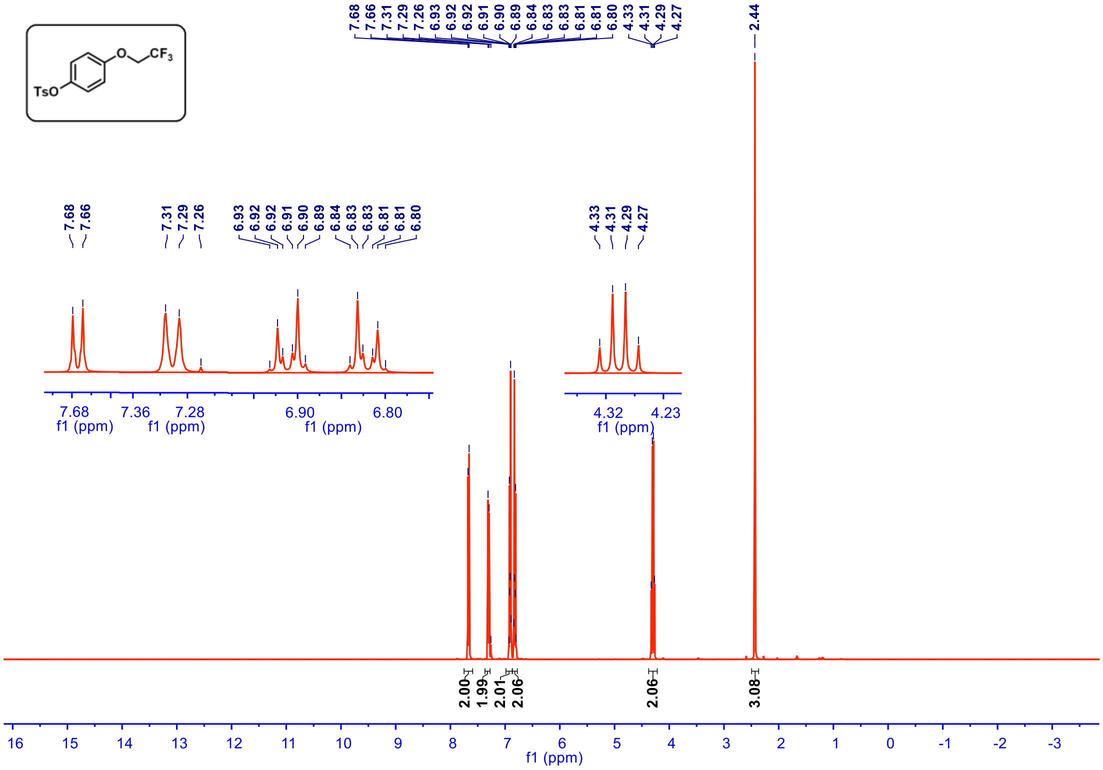

Supplementary Figure 366. ^{1}H NMR spectrum of compound **28** (400 MHz, $CDCl_3$)

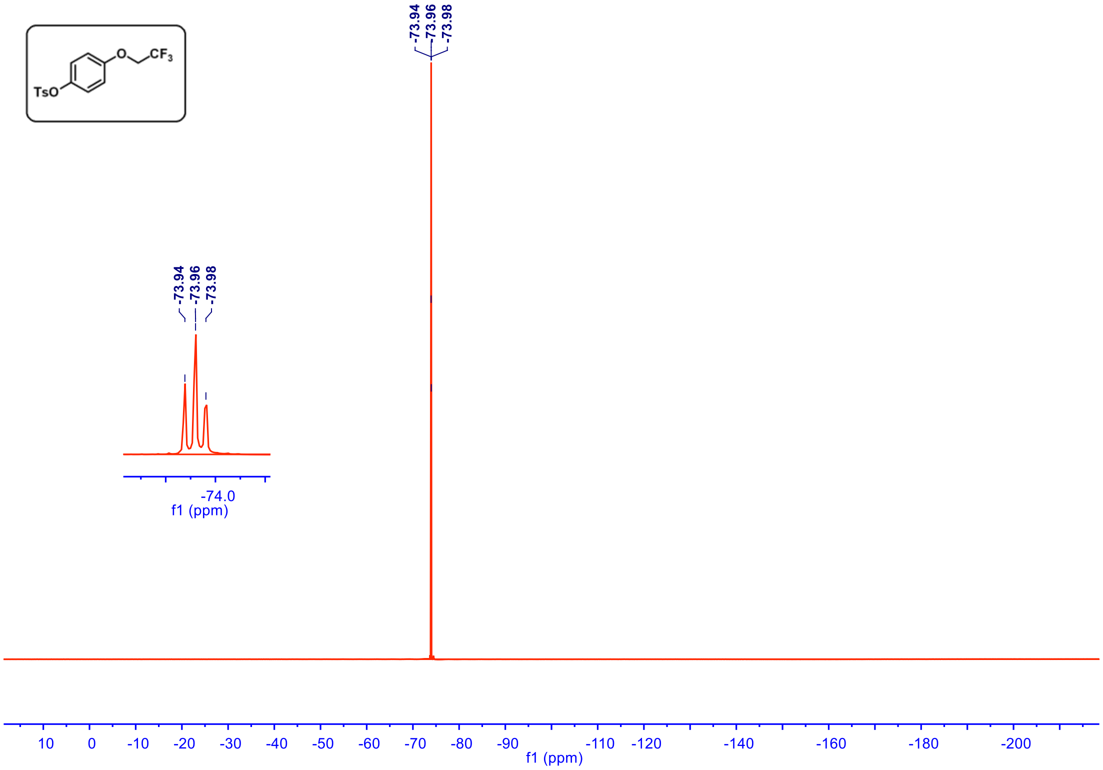

Supplementary Figure 367. ^{19}F NMR spectrum of compound **28** (376 MHz, $CDCl_3$)

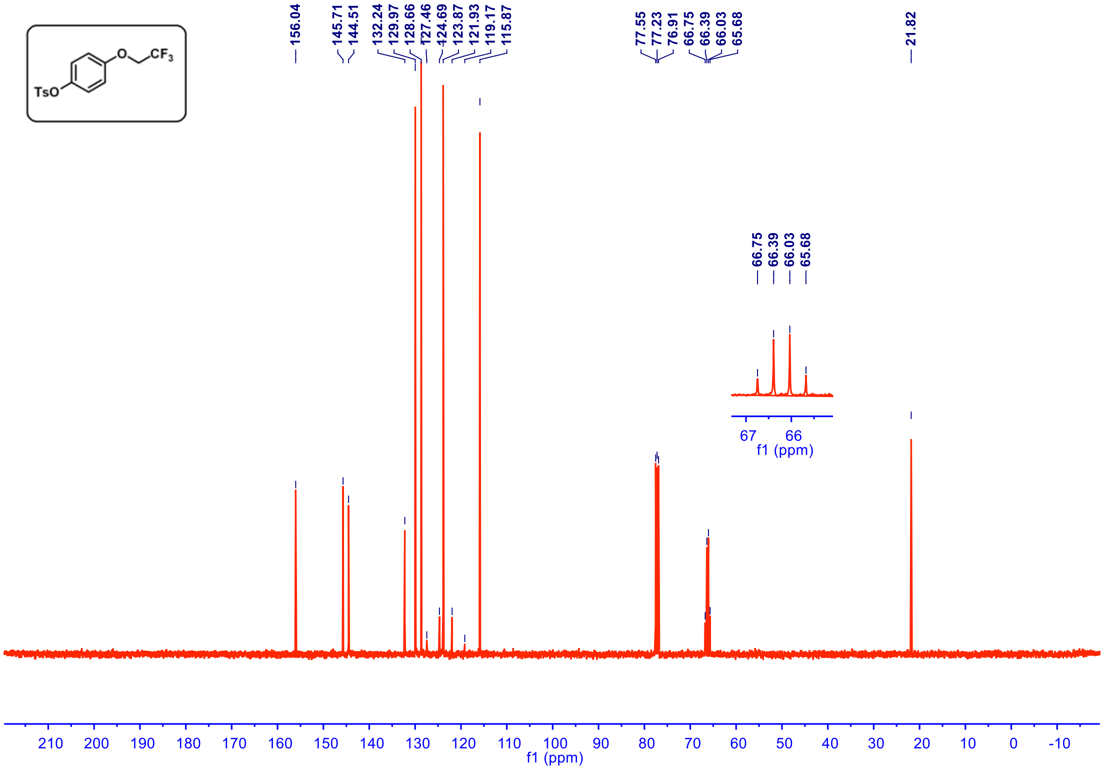

Supplementary Figure 368. ^{13}C NMR spectrum of compound **28** (101 MHz, $CDCl_3$)

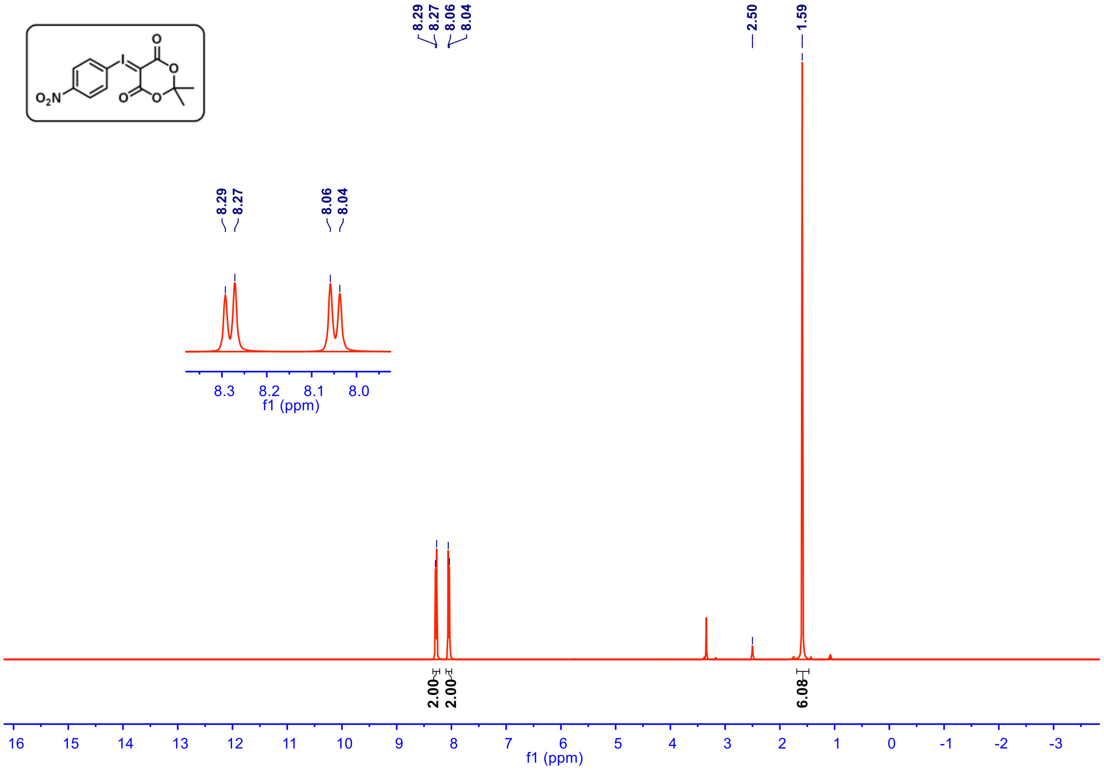

Supplementary Figure 369. ^{1}H NMR spectrum of **4-nitrophenyl)iodonium-(2,2-dimethyl-1,3-dioxane-4,6–dione)ylide** (400 MHz, $CDCl_3$)

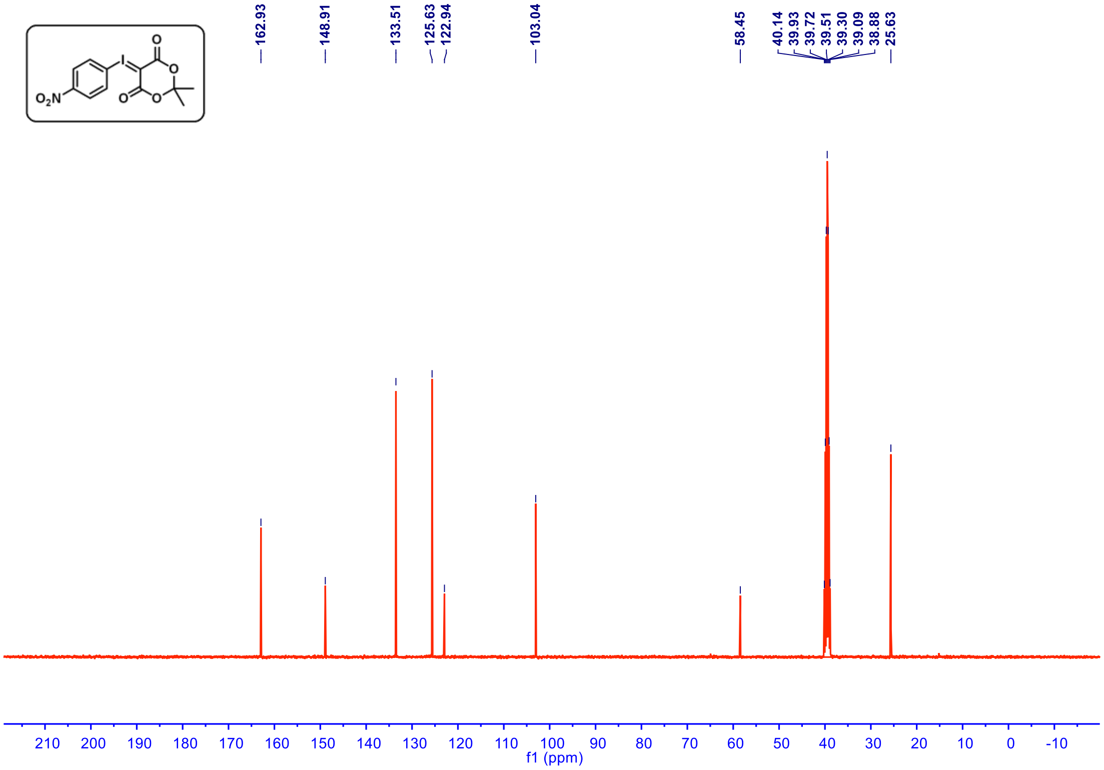

Supplementary Figure 370. ^{13}C NMR spectrum of **4-nitrophenyl)iodonium-(2,2-dimethyl-1,3-dioxane-4,6–dione)ylide** (101 MHz, $CDCl_3$)

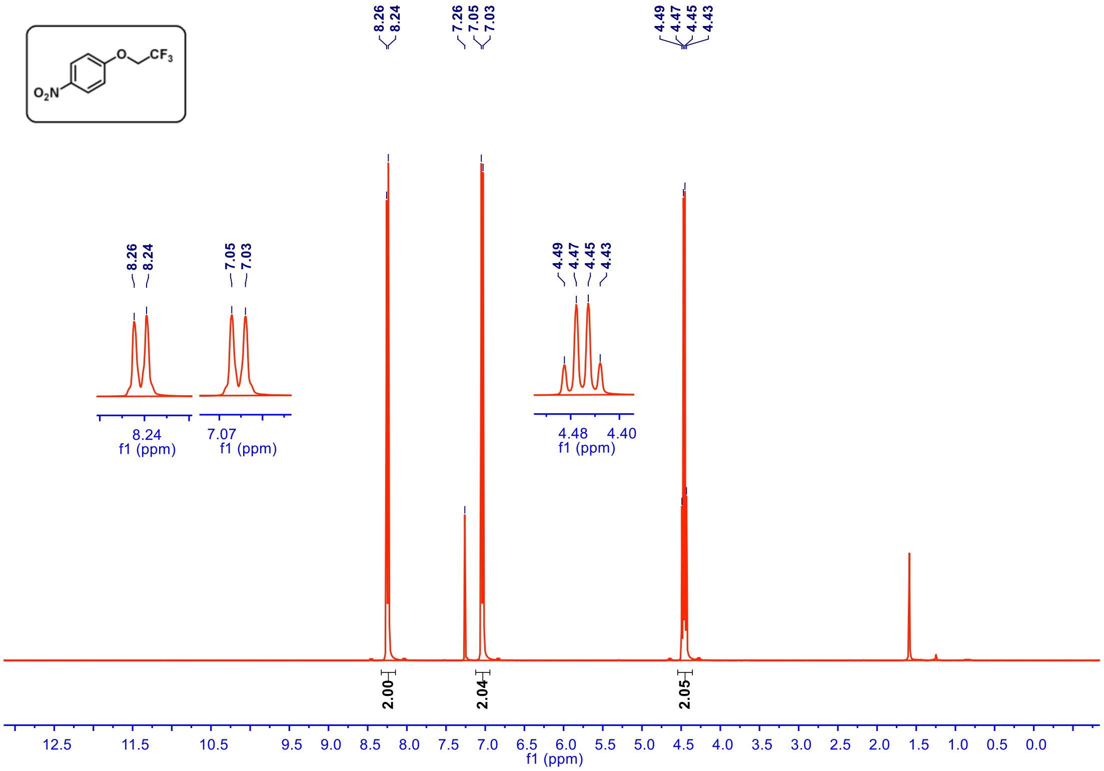

Supplementary Figure 371. ^{1}H NMR spectrum of compound **29** (400 MHz, $CDCl_3$)

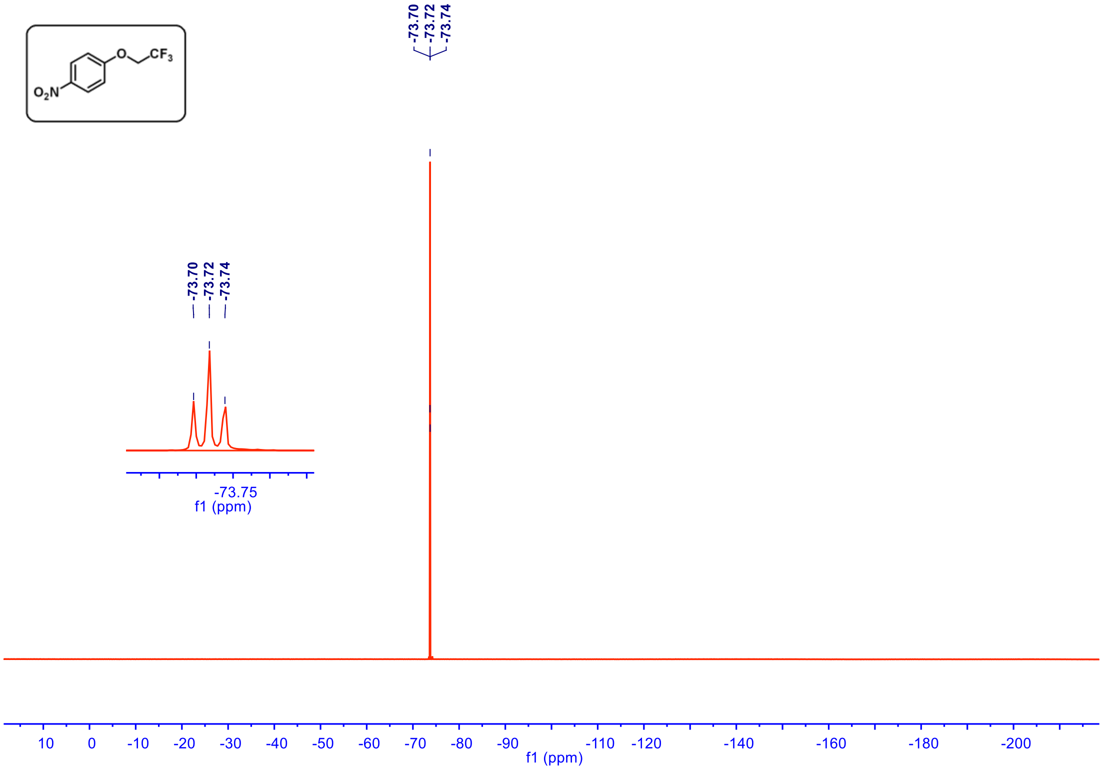

Supplementary Figure 372. ^{19}F NMR spectrum of compound **29** (376 MHz, $CDCl_3$)

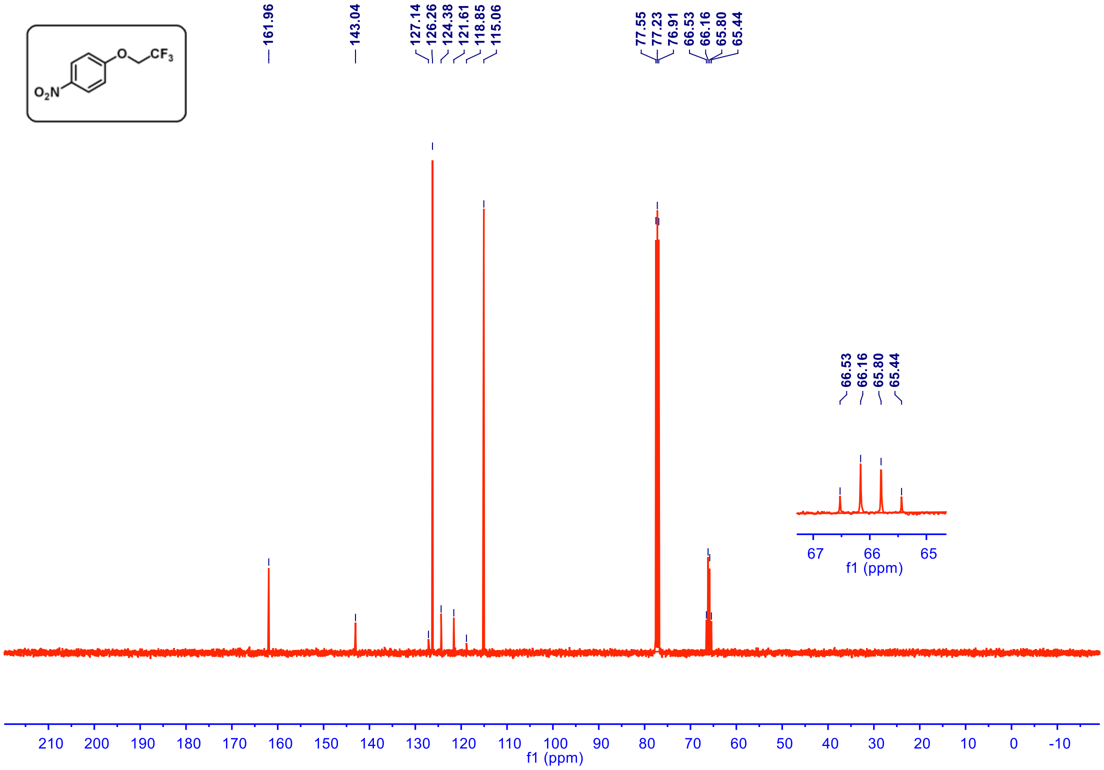

Supplementary Figure 373. ^{13}C NMR spectrum of compound **29** (101 MHz, $CDCl_3$)

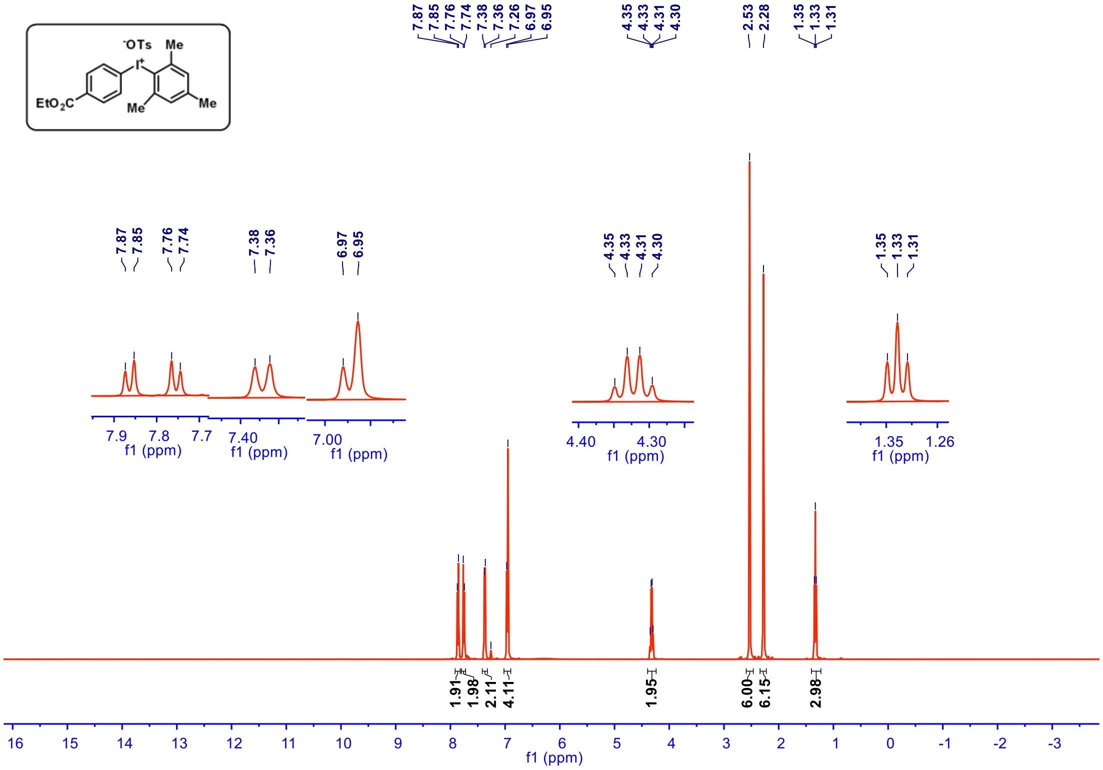

Supplementary Figure 374. ^{1}H NMR spectrum of **(4-ethoxycarbonylphenyl)(mesityl)iodonium tosylate** (400 MHz, $CDCl_3$)

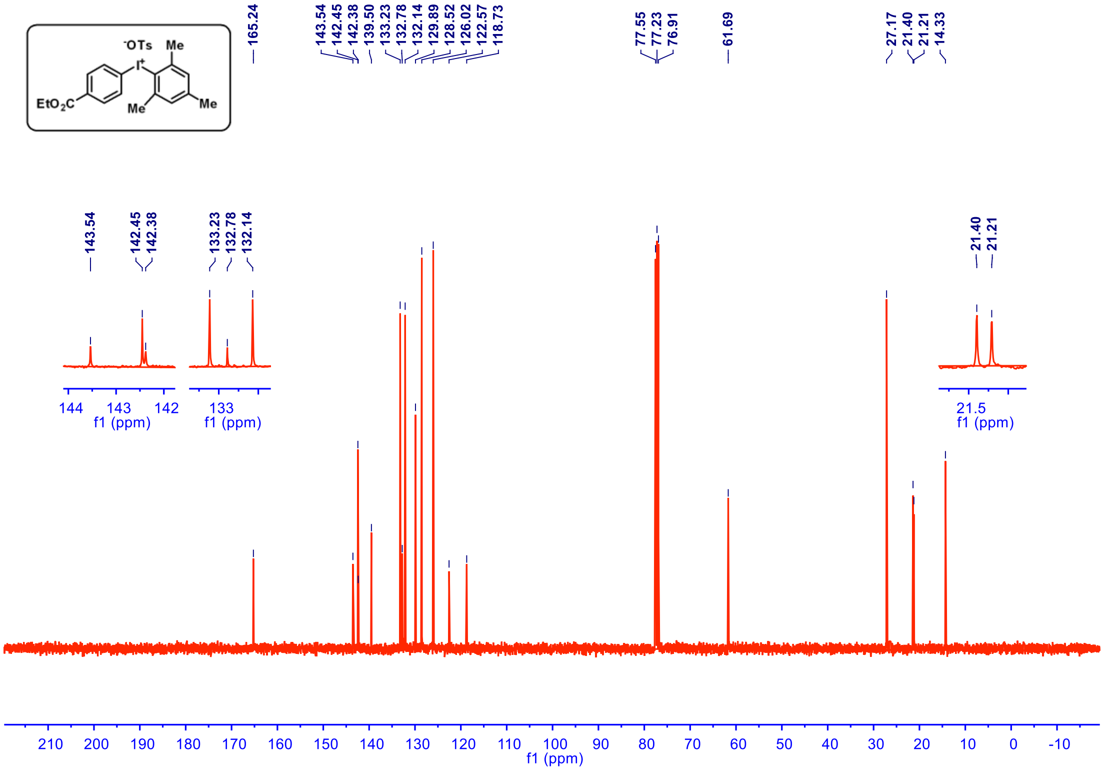

Supplementary Figure 375. ^{13}C NMR spectrum of **(4-ethoxycarbonylphenyl)(mesityl)iodonium tosylate** (101 MHz, $CDCl_3$)

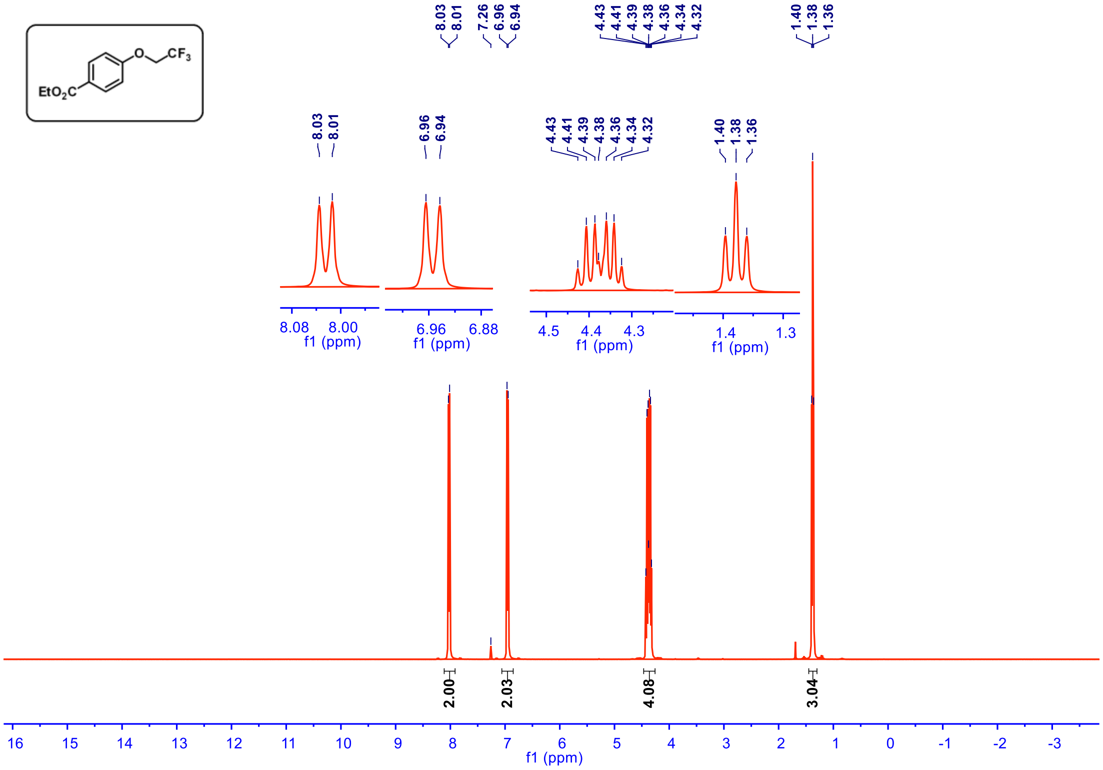

Supplementary Figure 376. ^{1}H NMR spectrum of compound **30** (400 MHz, $CDCl_3$)

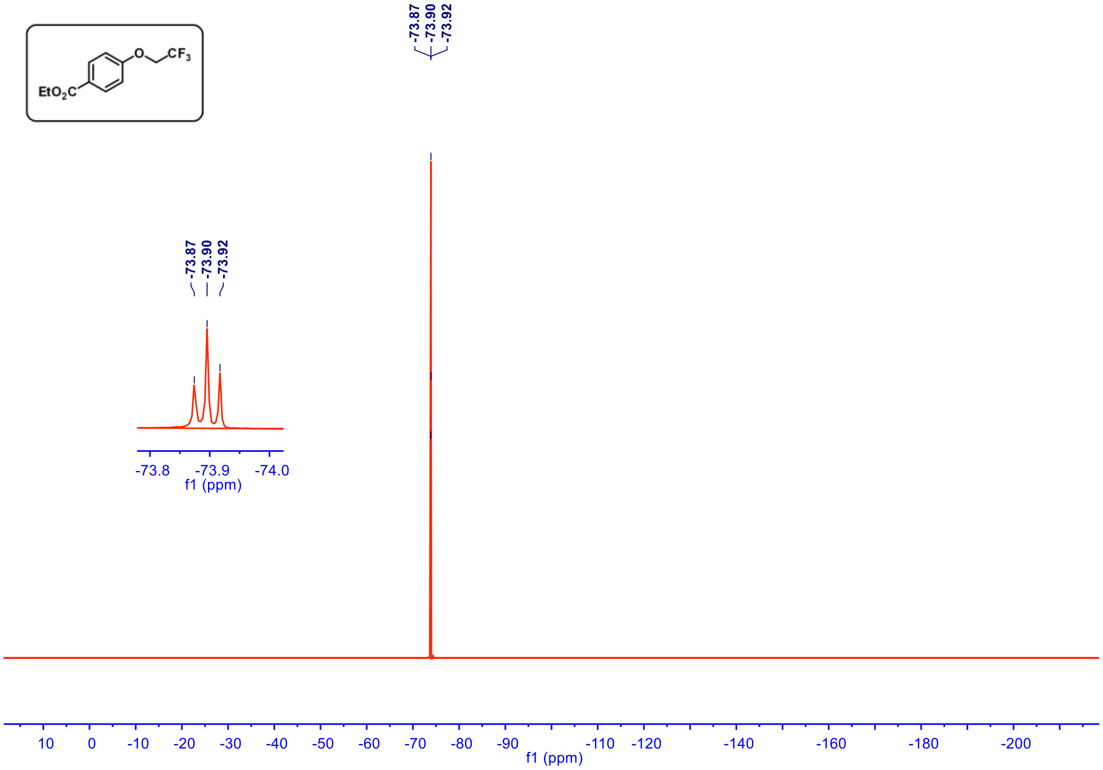

Supplementary Figure 377. ^{19}F NMR spectrum of compound **30** (376 MHz, $CDCl_3$)

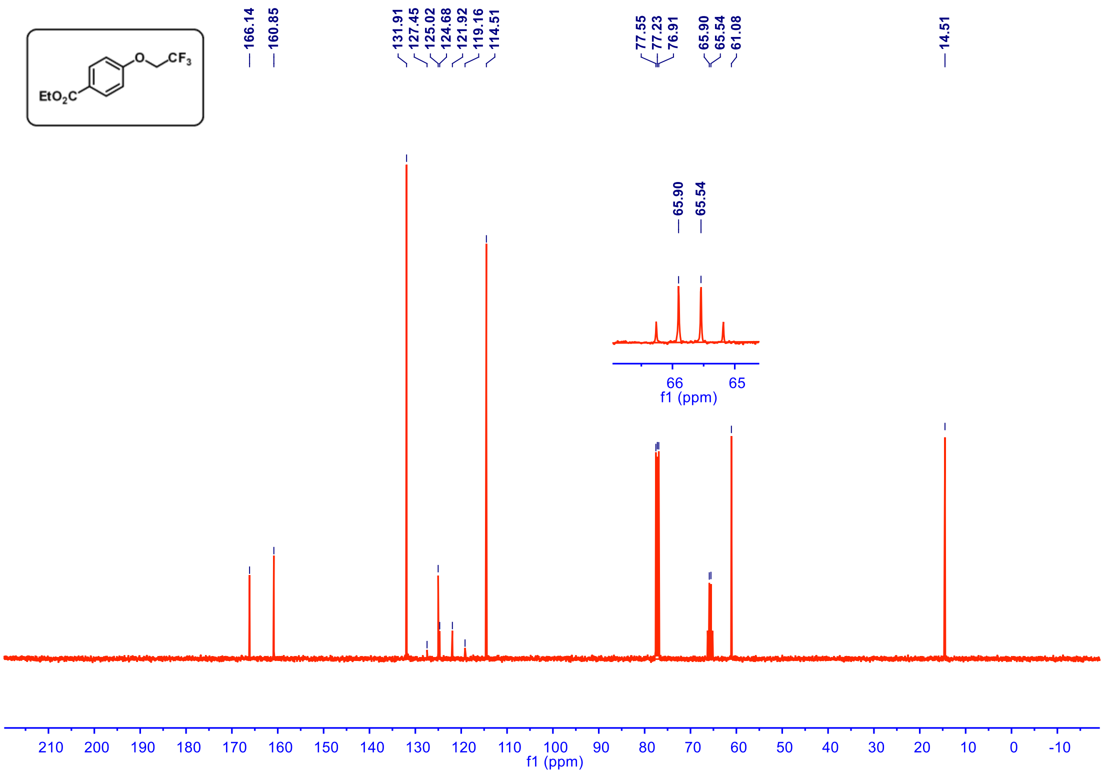

Supplementary Figure 378. ^{13}C NMR spectrum of compound **30** (101 MHz, $CDCl_3$)

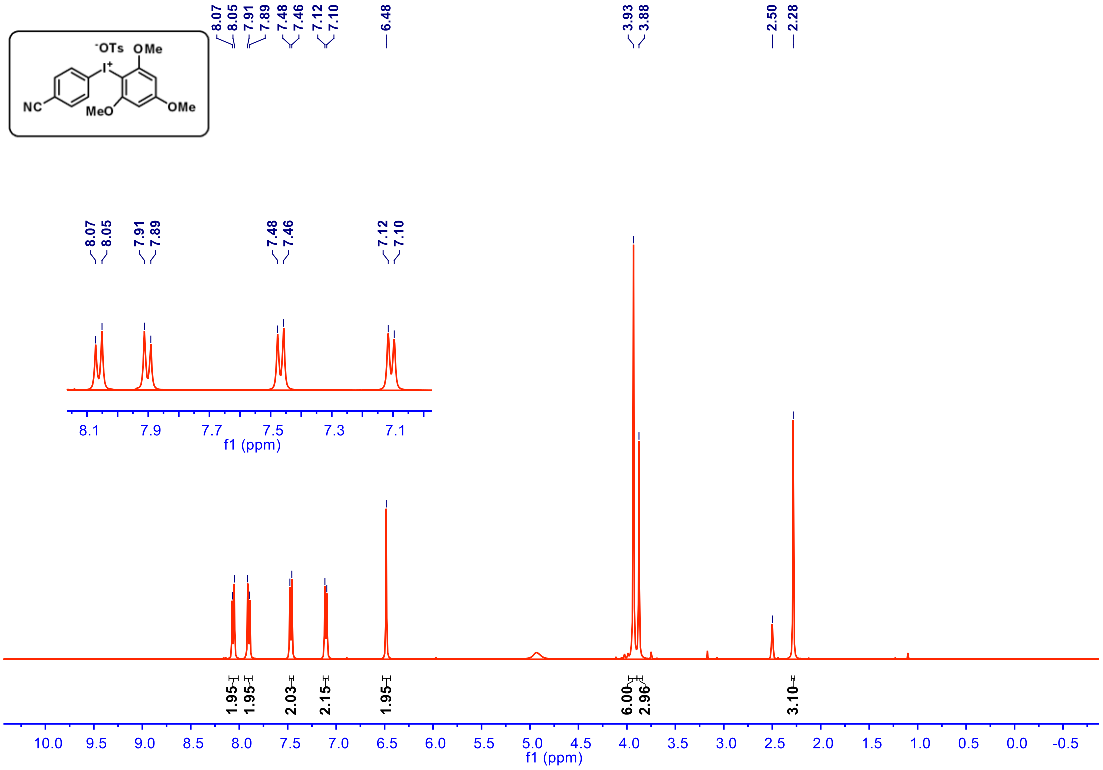

Supplementary Figure 379. ^{1}H NMR spectrum of **(4-Cyanophenyl)(2,4,6-trimethoxyphenyl)iodonium tosylate** (400 MHz, $CDCl_3$)

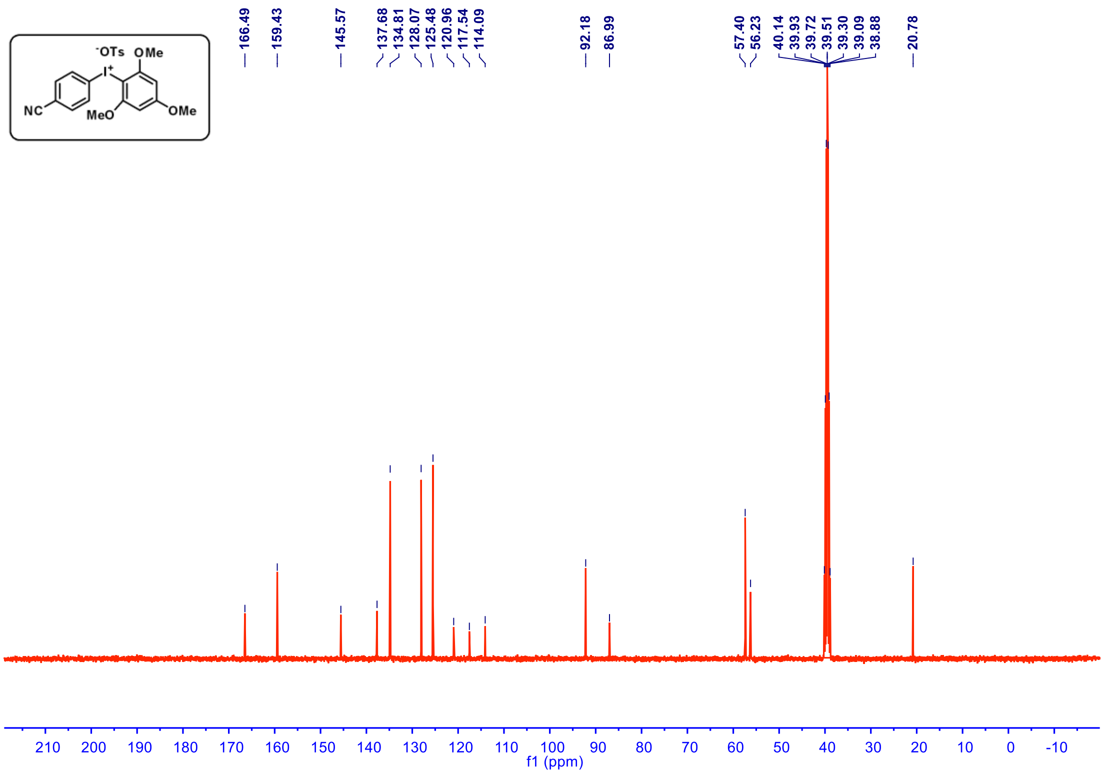

Supplementary Figure 380. ^{13}C NMR spectrum of **(4-Cyanophenyl)(2,4,6-trimethoxyphenyl)iodonium tosylate** (101 MHz, $CDCl_3$)

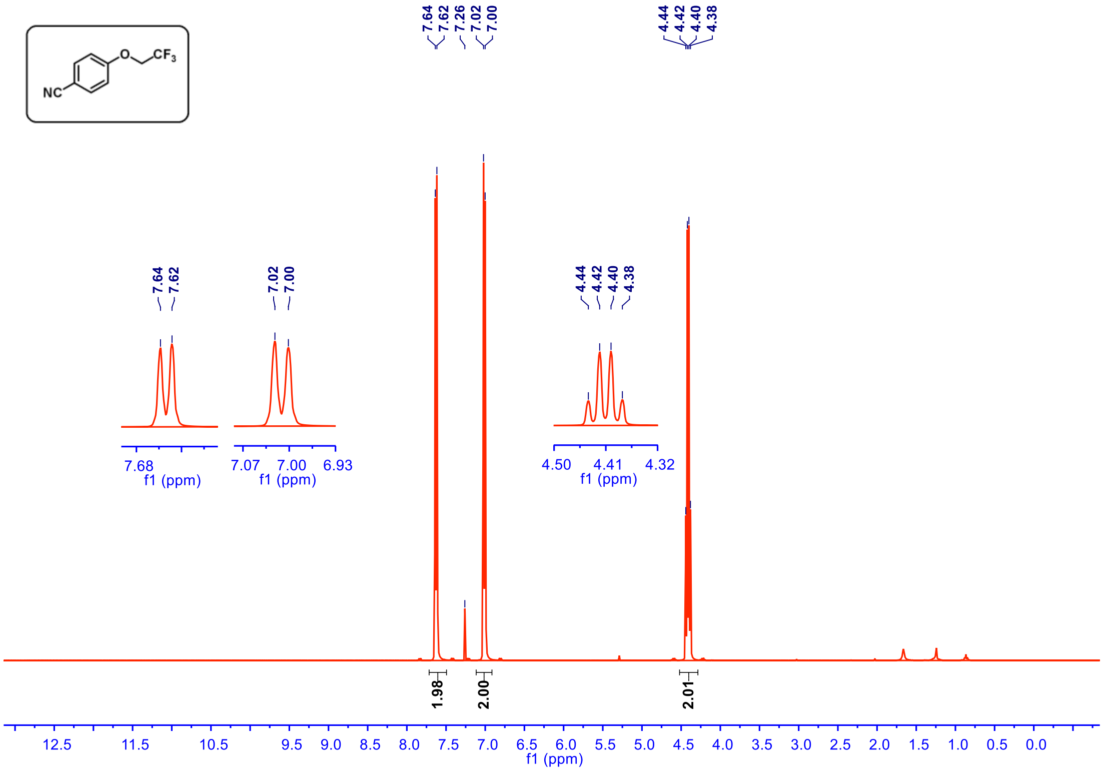

Supplementary Figure 381. ^{1}H NMR spectrum of compound **31** (400 MHz, $CDCl_3$)

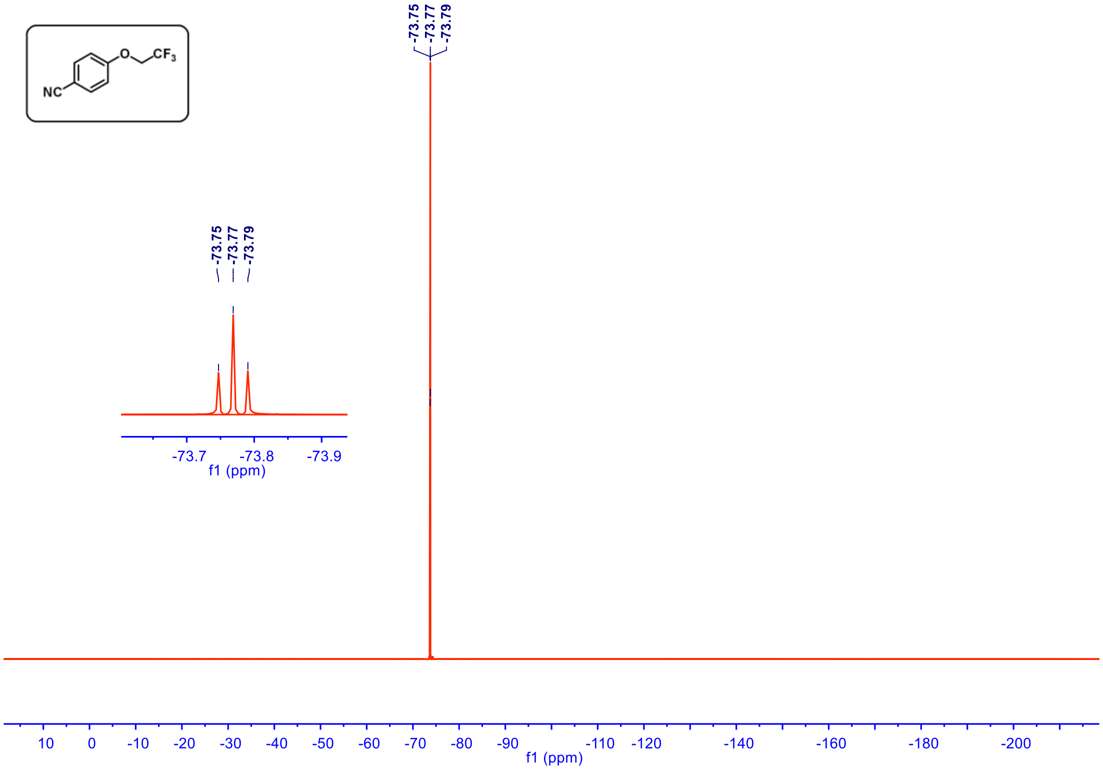

Supplementary Figure 382. ^{19}F NMR spectrum of compound **31** (376 MHz, $CDCl_3$)

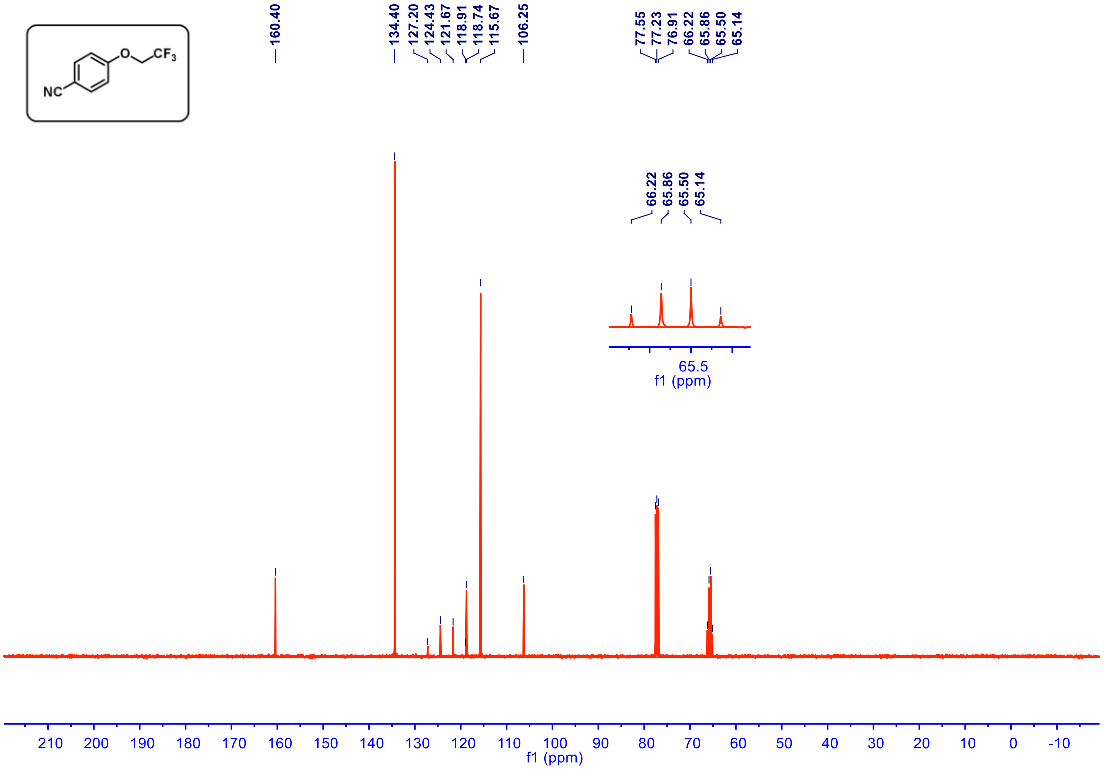

Supplementary Figure 383. ^{13}C NMR spectrum of compound **31** (101 MHz, $CDCl_3$)

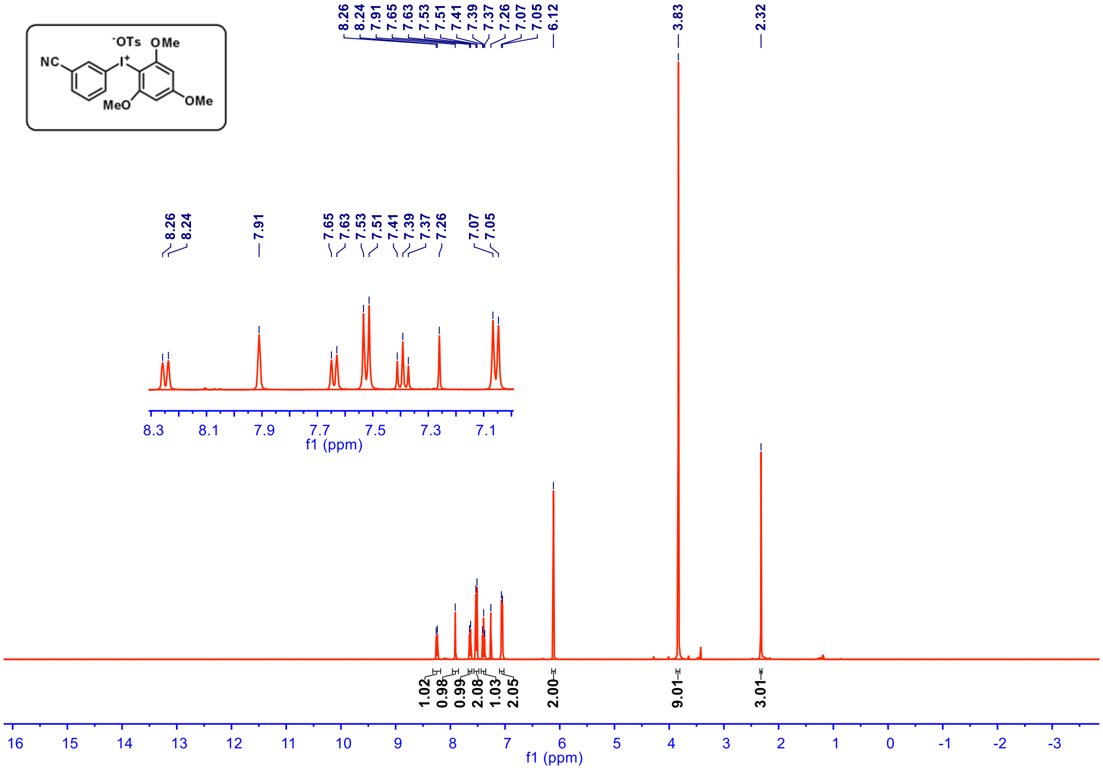

Supplementary Figure 384. ^{1}H NMR spectrum of **(3-cyanophenyl) (2,4,6-trimethoxyphenyl)iodonium tosylate** (400 MHz, $CDCl_3$)

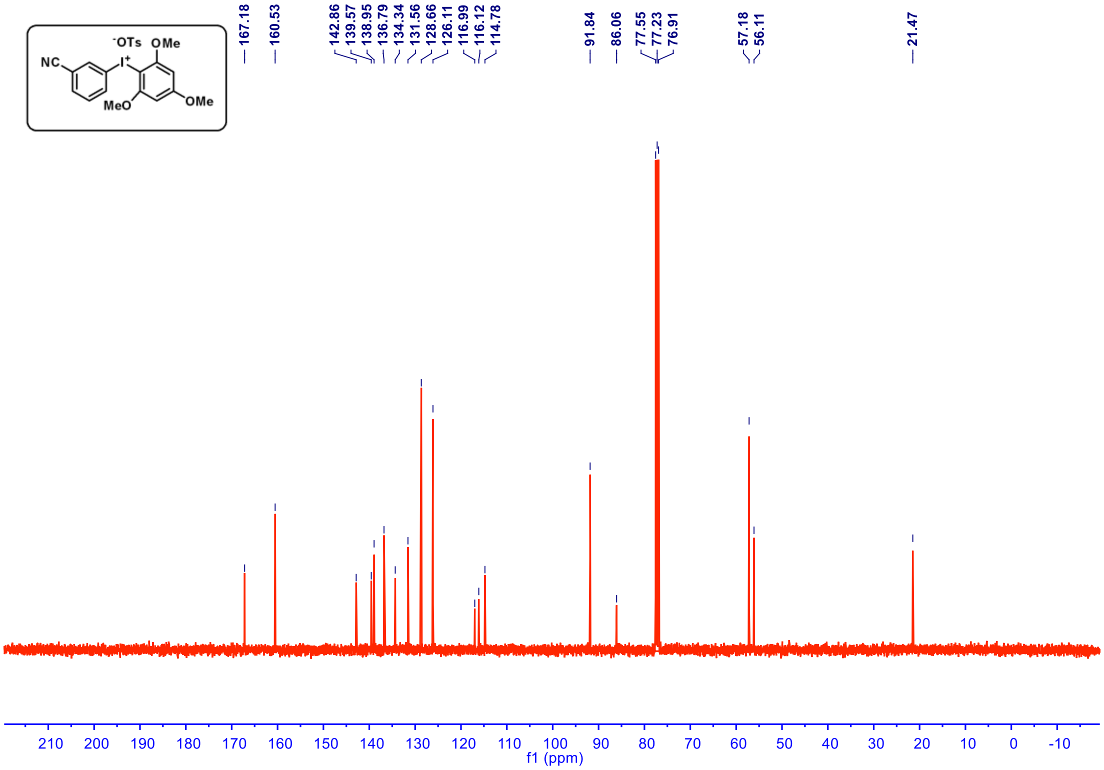

Supplementary Figure 385. ^{13}C NMR spectrum of **(3-cyanophenyl) (2,4,6-trimethoxyphenyl)iodonium tosylate** (101 MHz, $CDCl_3$)

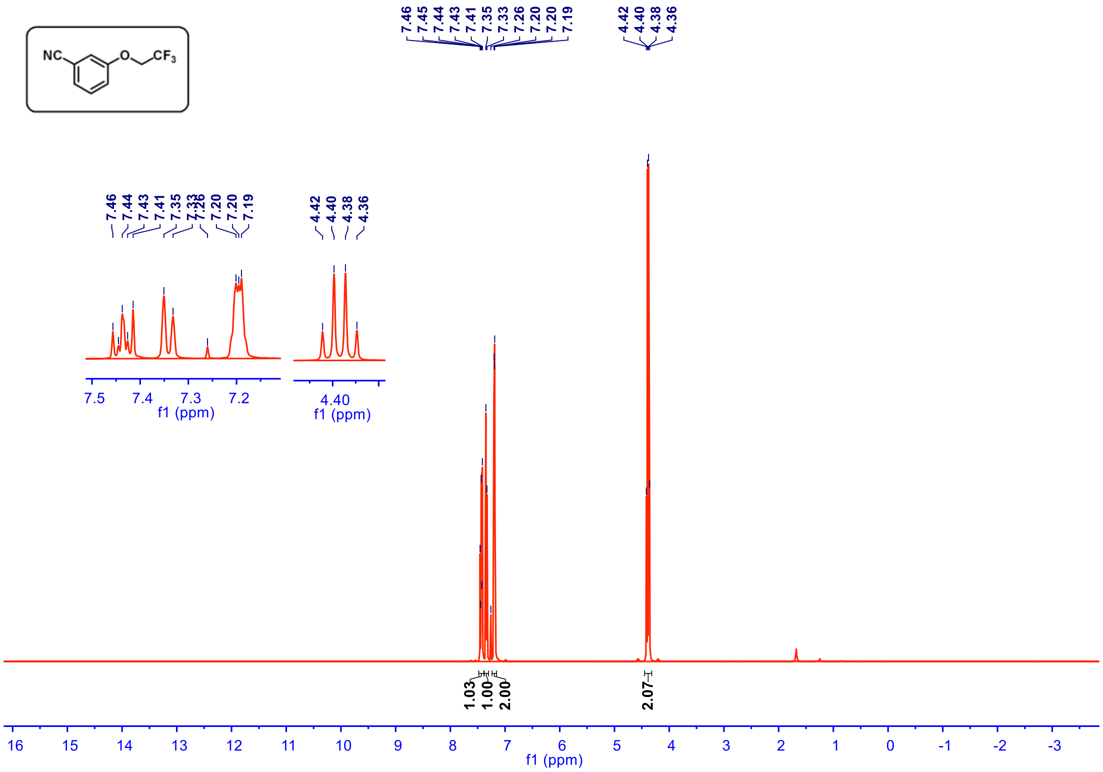

Supplementary Figure 386. ^{1}H NMR spectrum of compound **32** (400 MHz, $CDCl_3$)

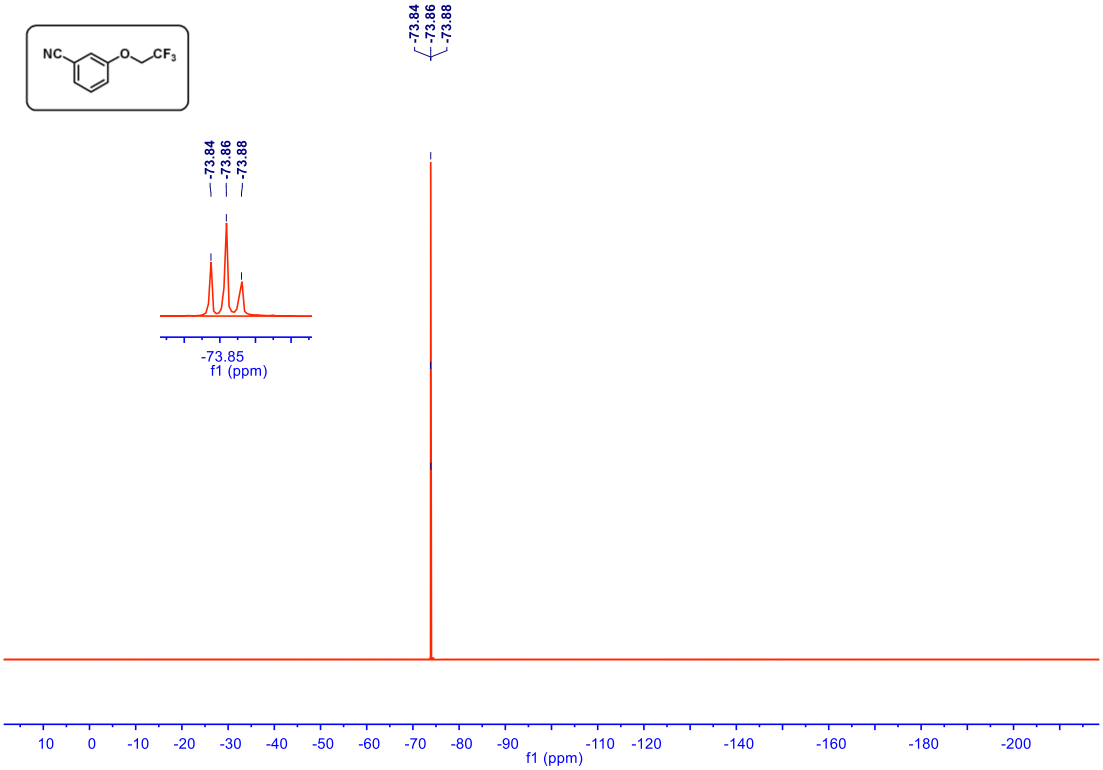

Supplementary Figure 387. ^{19}F NMR spectrum of compound **32** (376 MHz, $CDCl_3$)

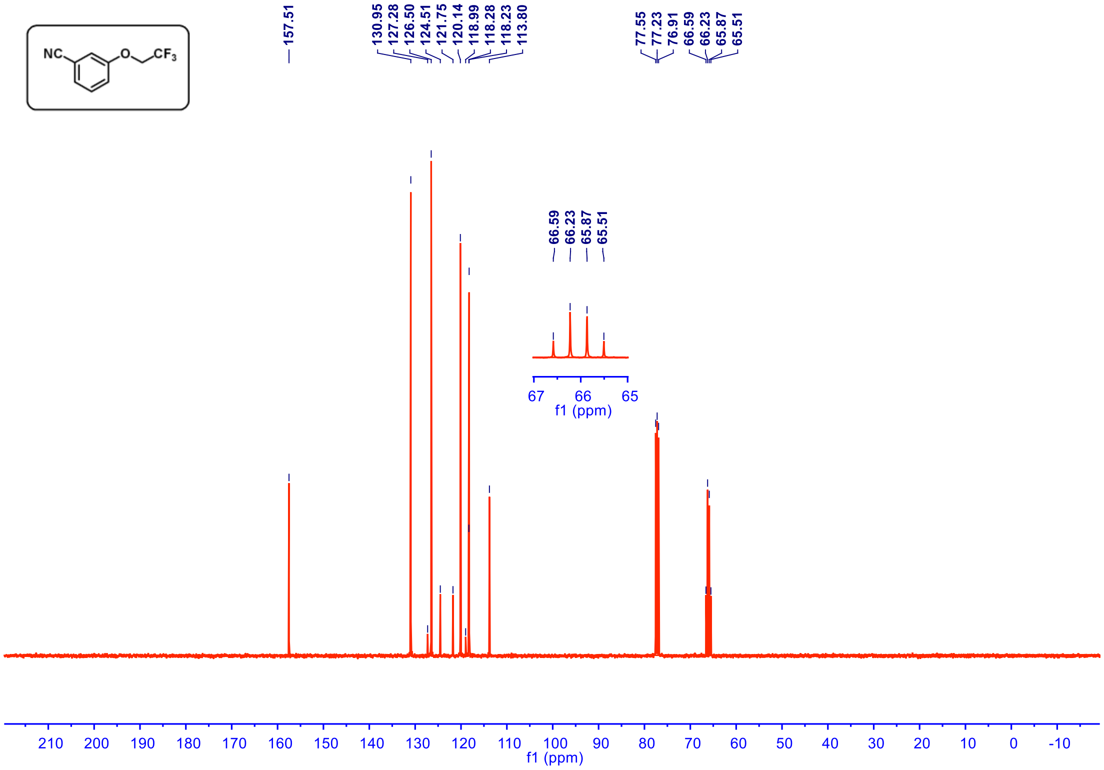

Supplementary Figure 388. ^{13}C NMR spectrum of compound **32** (101 MHz, $CDCl_3$)

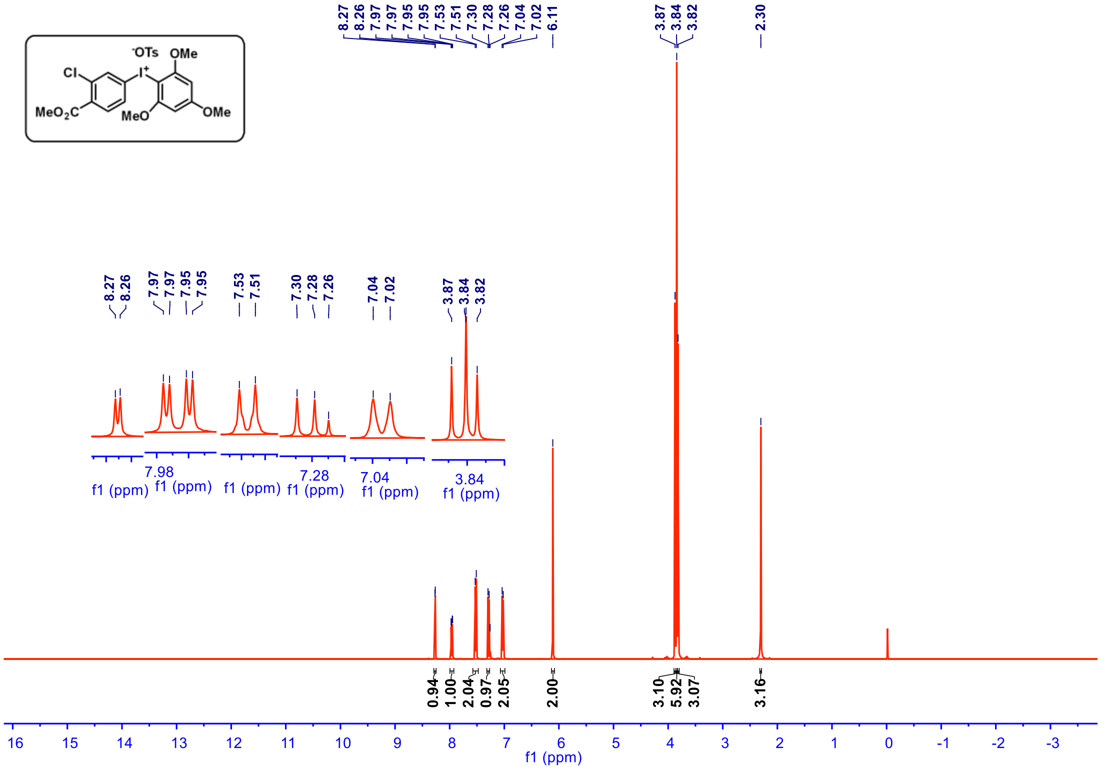

Supplementary Figure 389. ^{1}H NMR spectrum of **(3-chloro-4-(methoxycarbonyl)phenyl)(2,4,6-trimethoxyphenyl) tosylate** (400 MHz, $CDCl_3$)

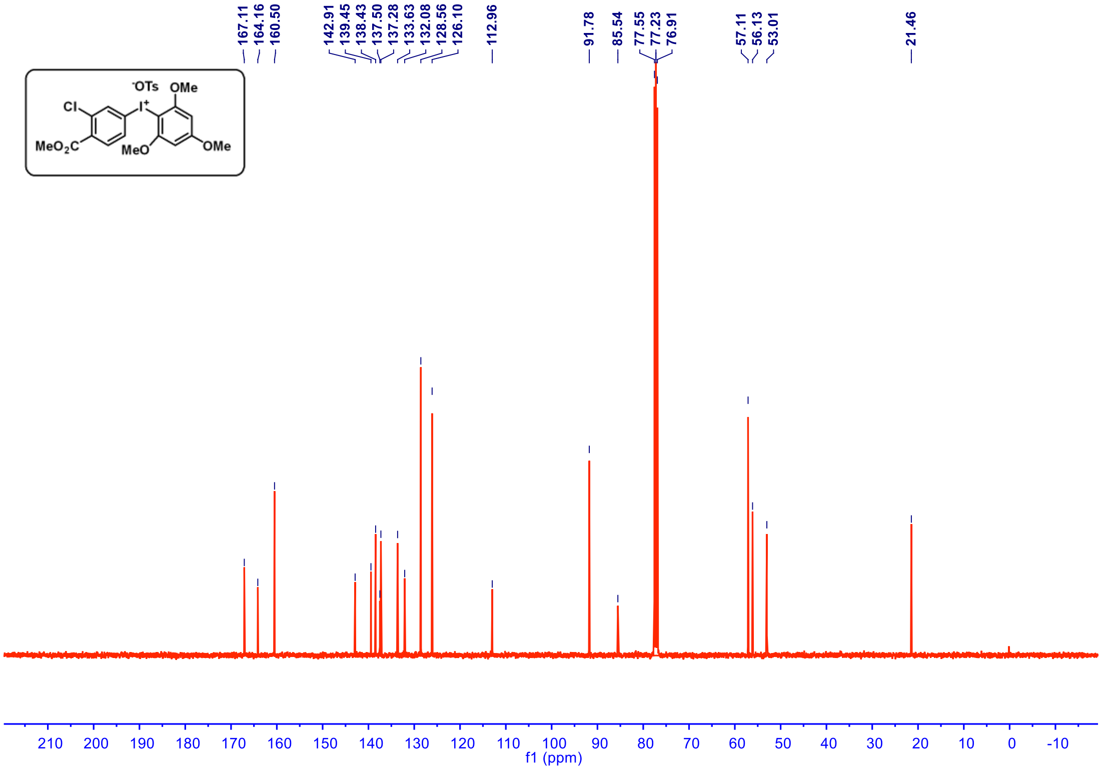

Supplementary Figure 390. ^{13}C NMR spectrum of **(3-chloro-4-(methoxycarbonyl)phenyl)(2,4,6-trimethoxyphenyl) tosylate** (101 MHz, $CDCl_3$)

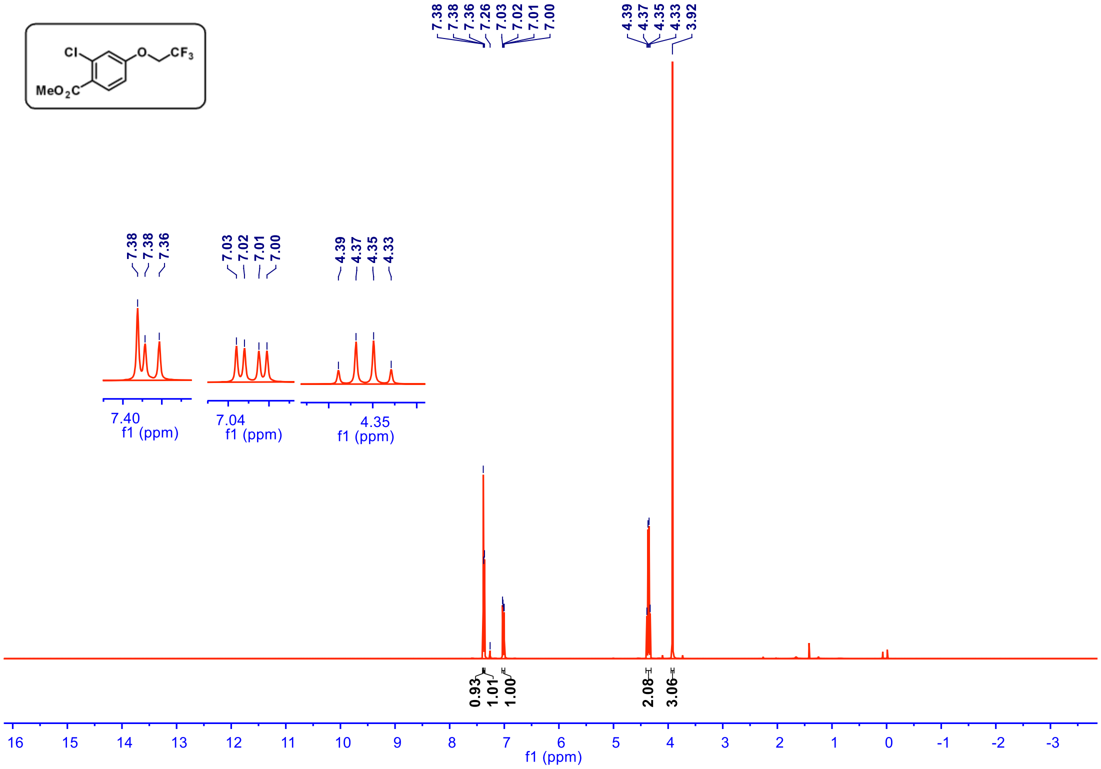

Supplementary Figure 391. ^{1}H NMR spectrum of compound **33** (400 MHz, $CDCl_3$)

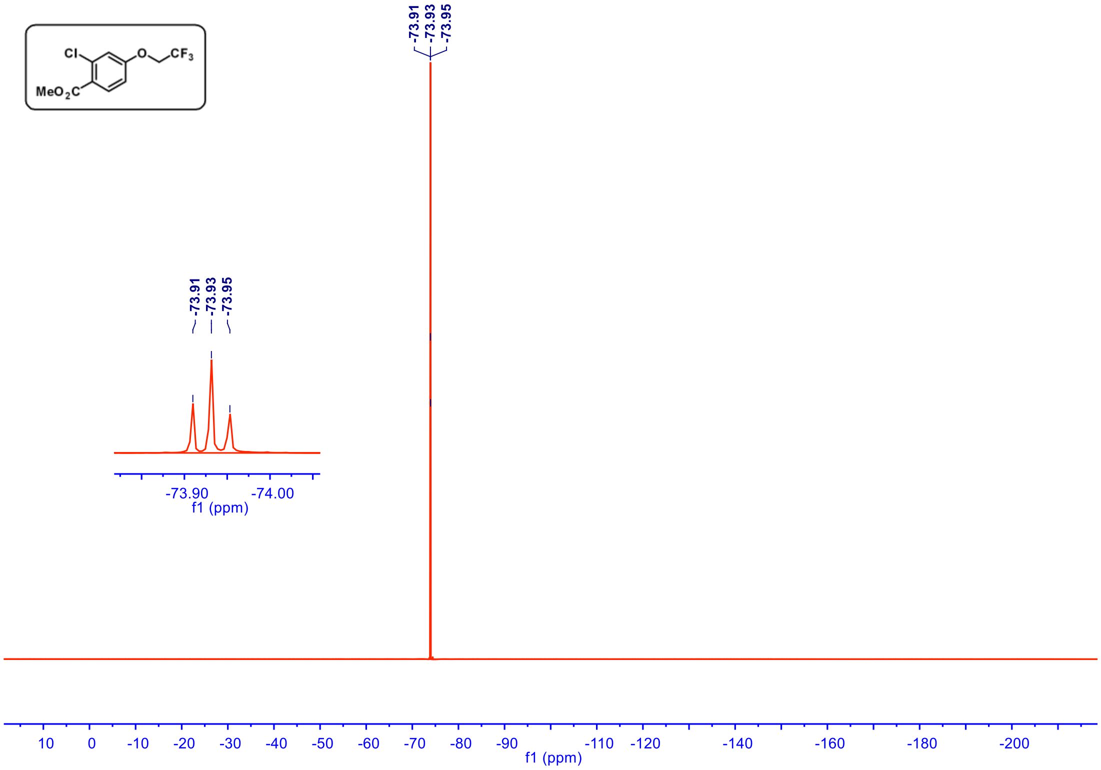

Supplementary Figure 392. ^{19}F NMR spectrum of compound **33** (376 MHz, $CDCl_3$)

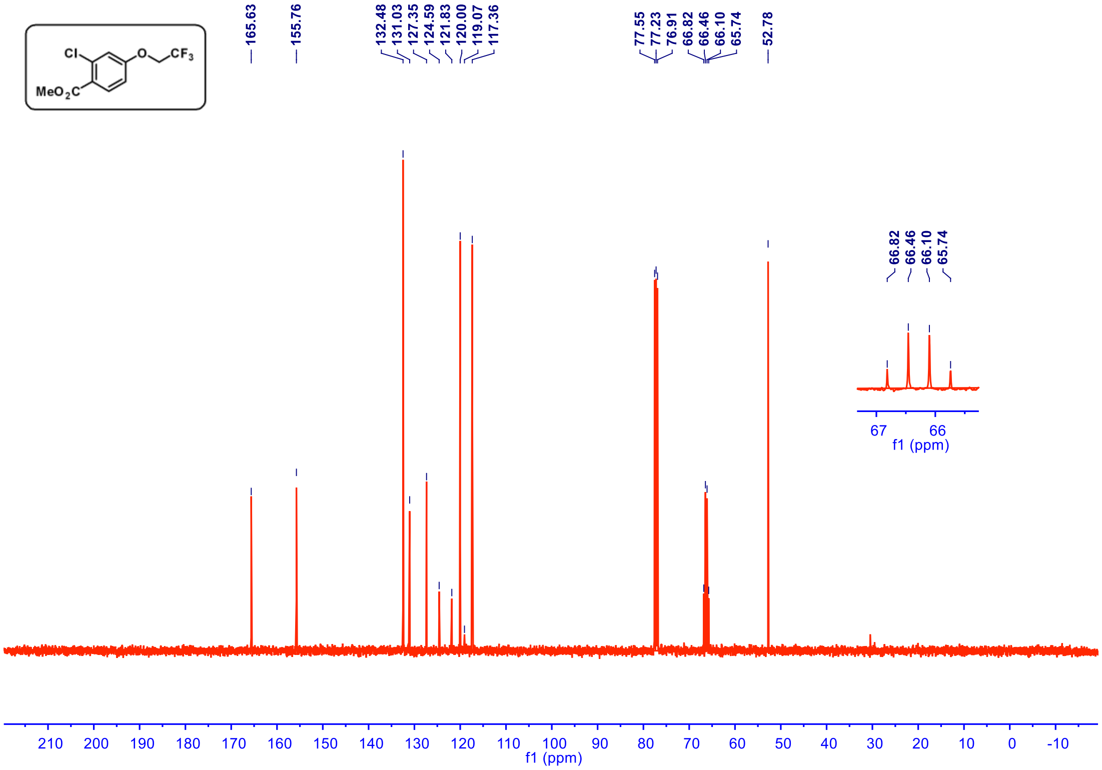

Supplementary Figure 393. ^{13}C NMR spectrum of compound **33** (101 MHz, $CDCl_3$)

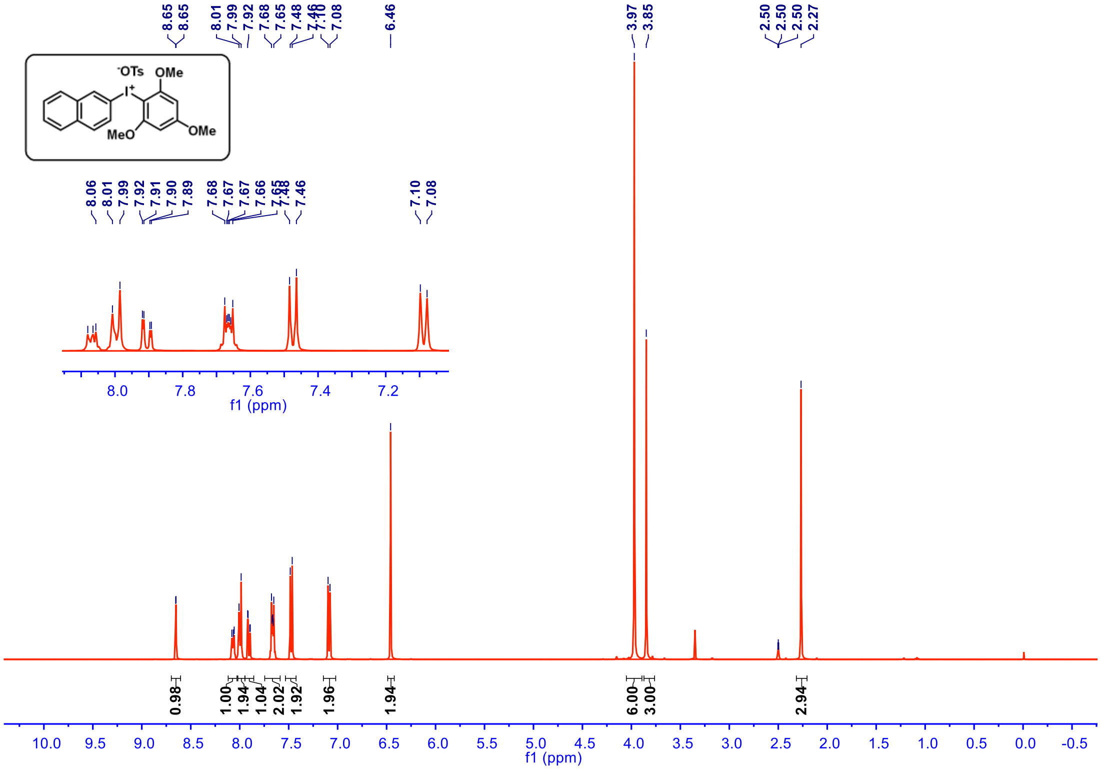

Supplementary Figure 394. ^{1}H NMR spectrum of **naphthalen-2-yl(2,4,6-trimethoxyphenyl)iodonium tosylate** (400 MHz, $CDCl_3$)

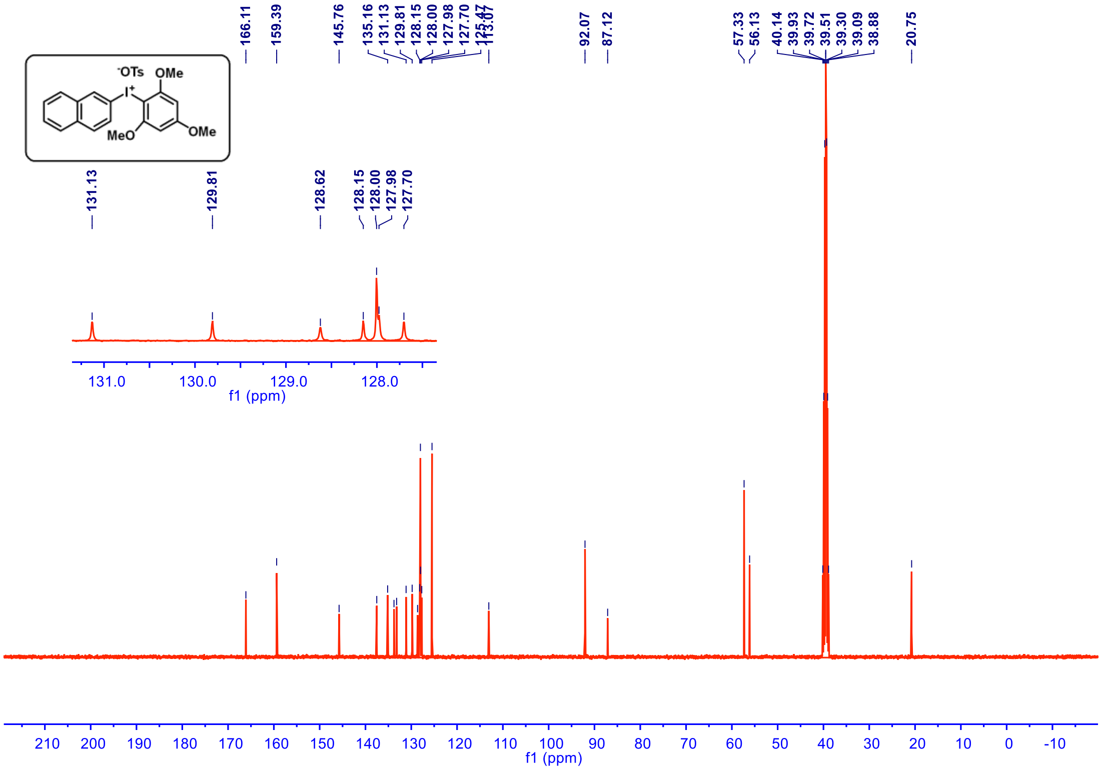

Supplementary Figure 395. ^{13}C NMR spectrum of **naphthalen-2-yl(2,4,6-trimethoxyphenyl)iodonium tosylate** (101 MHz, $CDCl_3$)

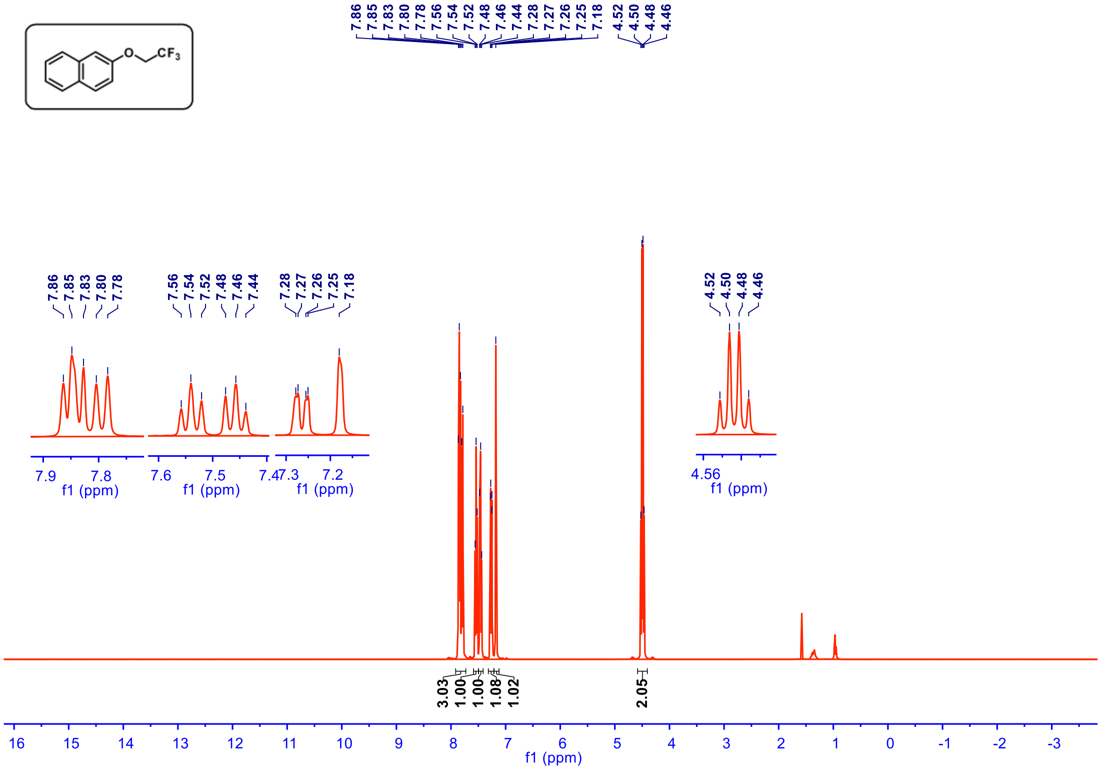

Supplementary Figure 396. ^{1}H NMR spectrum of compound **34** (400 MHz, $CDCl_3$)

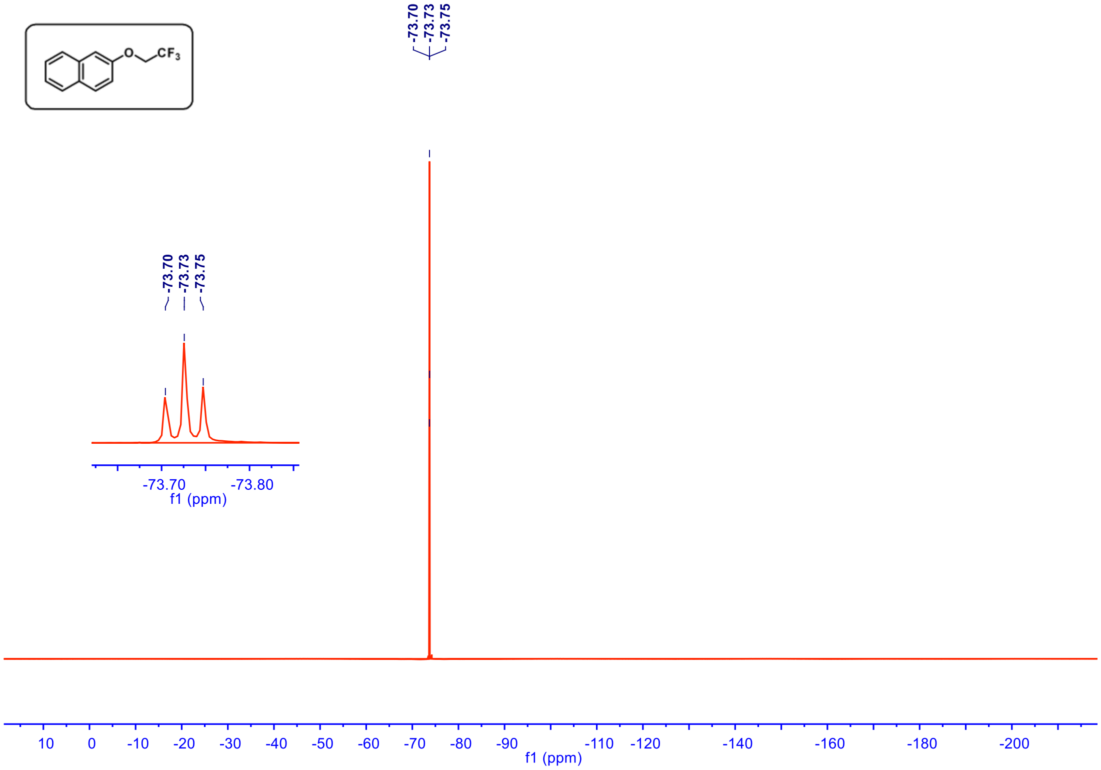

Supplementary Figure 397. ^{19}F NMR spectrum of compound **34** (376 MHz, $CDCl_3$)

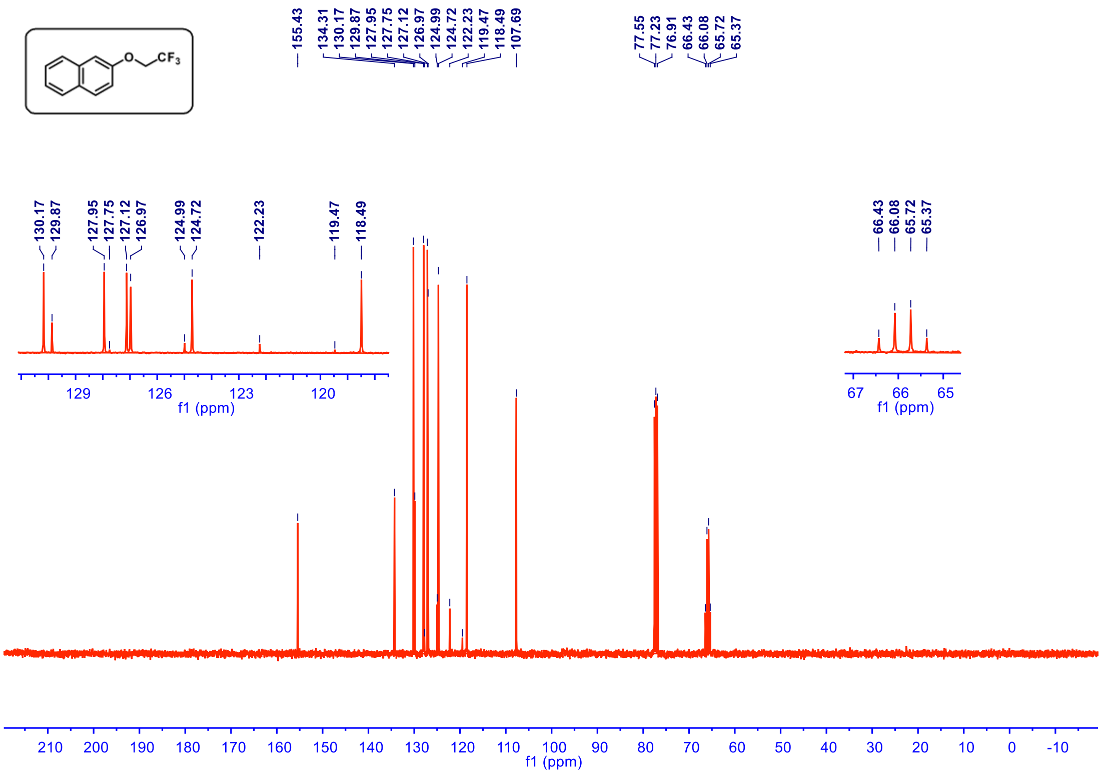

Supplementary Figure 398. ^{13}C NMR spectrum of compound **34** (101 MHz, $CDCl_3$)

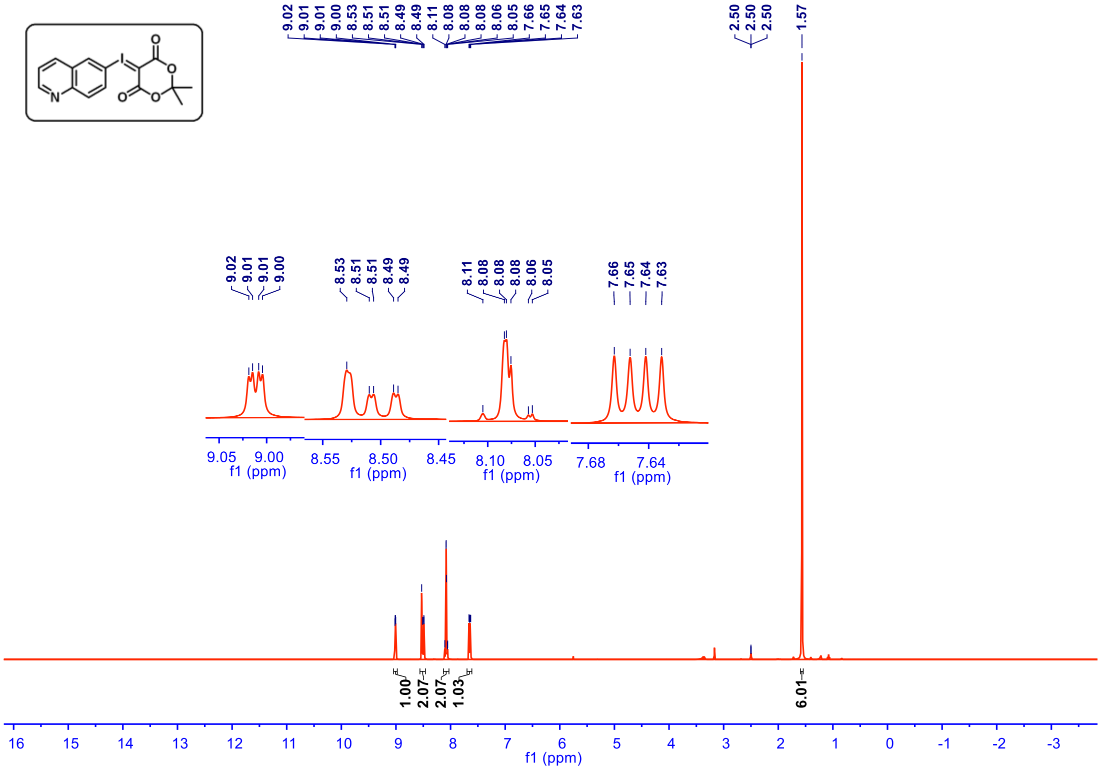

Supplementary Figure 399. ^{1}H NMR spectrum of **(quinolin-6-yl)iodonium-(2,2-dimethyl-1,3-dioxane-4,6-dione)ylide** (400 MHz, $CDCl_3$)

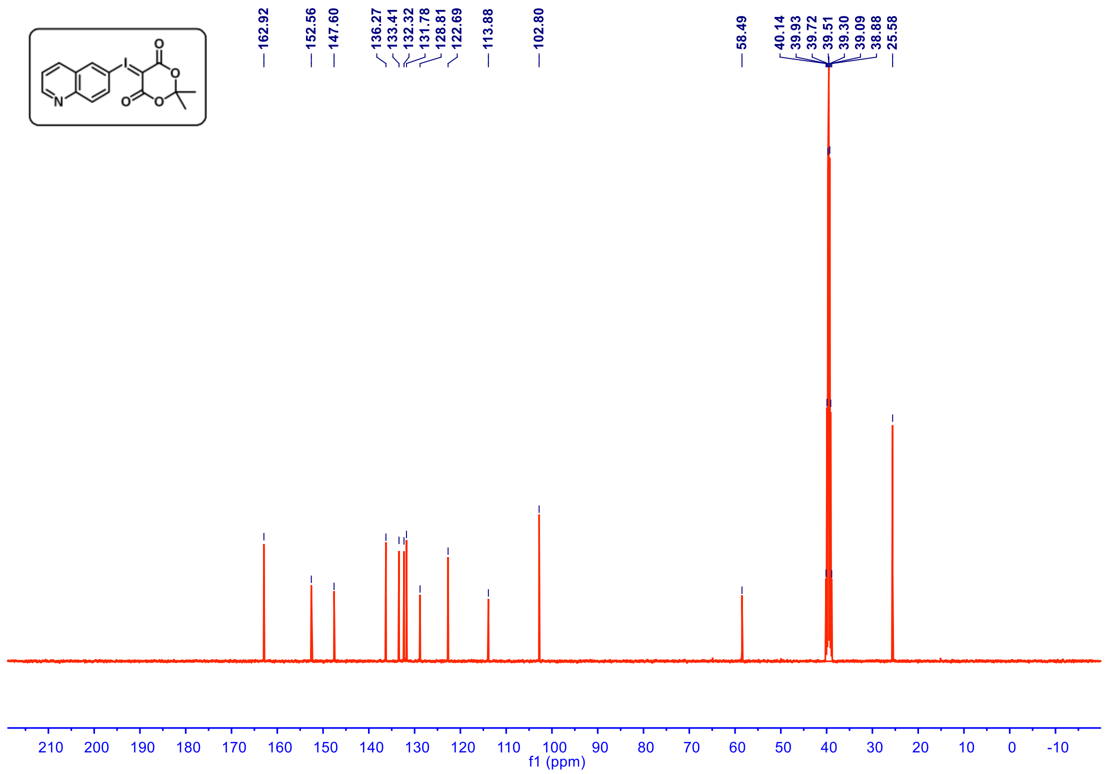

Supplementary Figure 400. ^{13}C NMR spectrum of **(quinolin-6-yl)iodonium-(2,2-dimethyl-1,3-dioxane-4,6-dione)ylide** (101 MHz, $CDCl_3$)

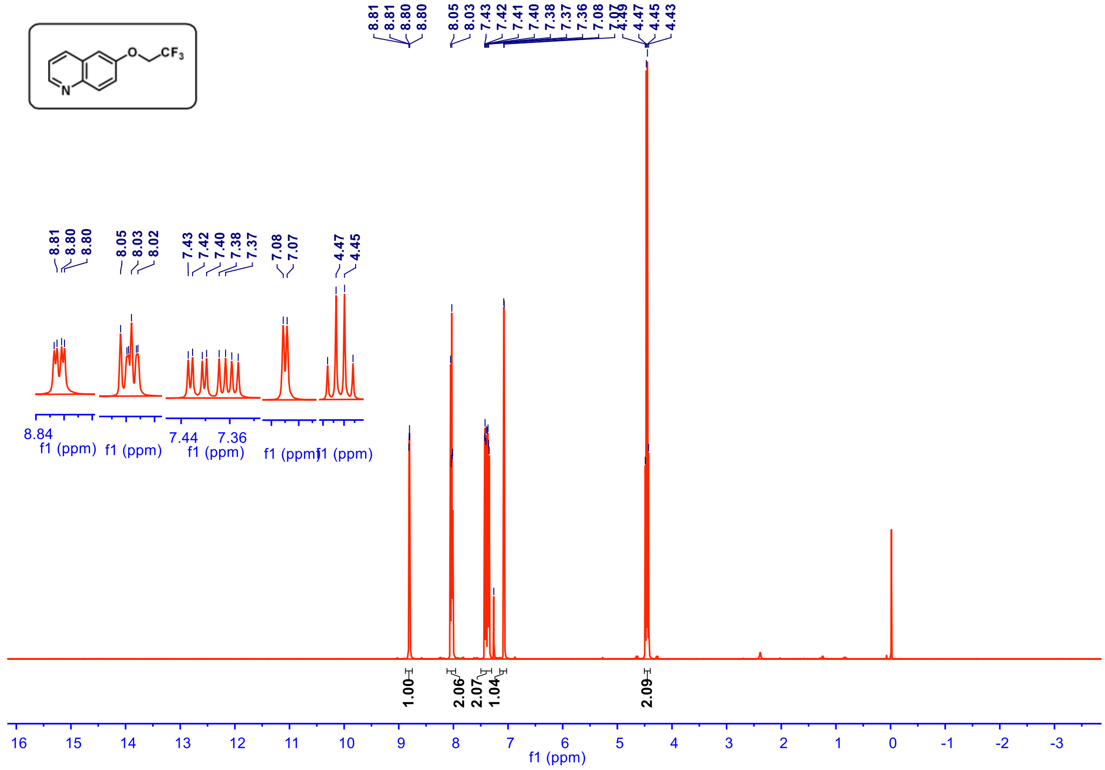

Supplementary Figure 401. ^{1}H NMR spectrum of compound **35** (400 MHz, $CDCl_3$)

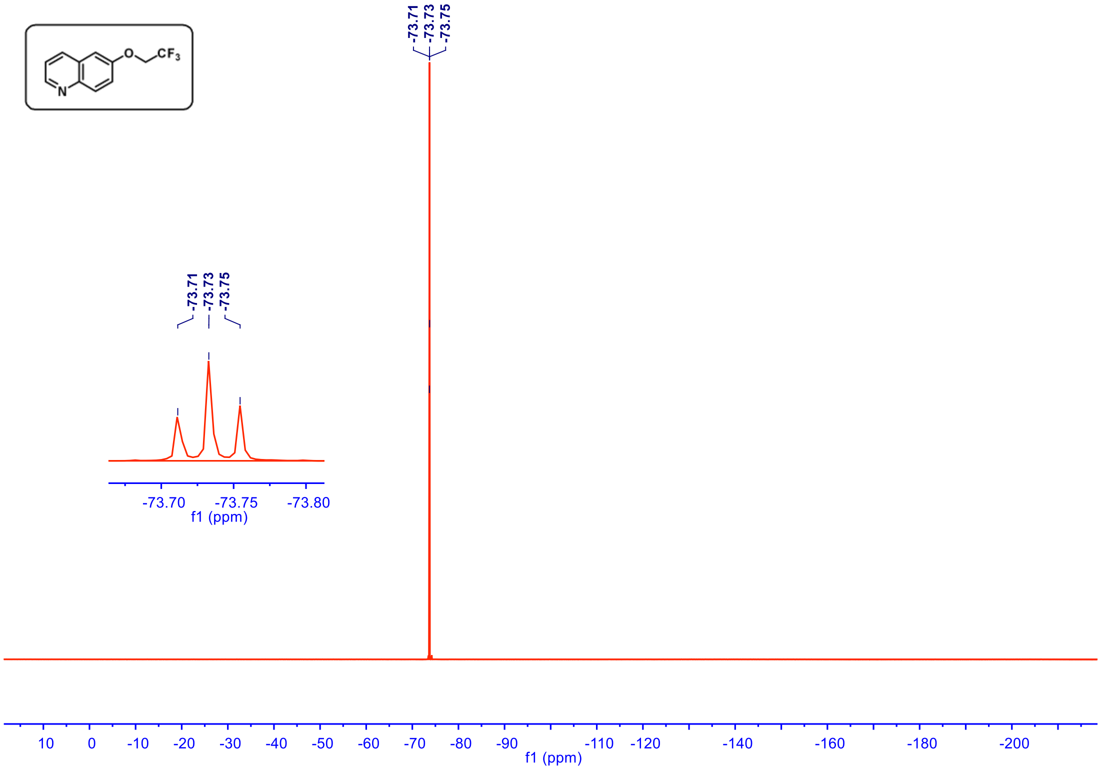

Supplementary Figure 402. ^{19}F NMR spectrum of compound **35** (376 MHz, $CDCl_3$)

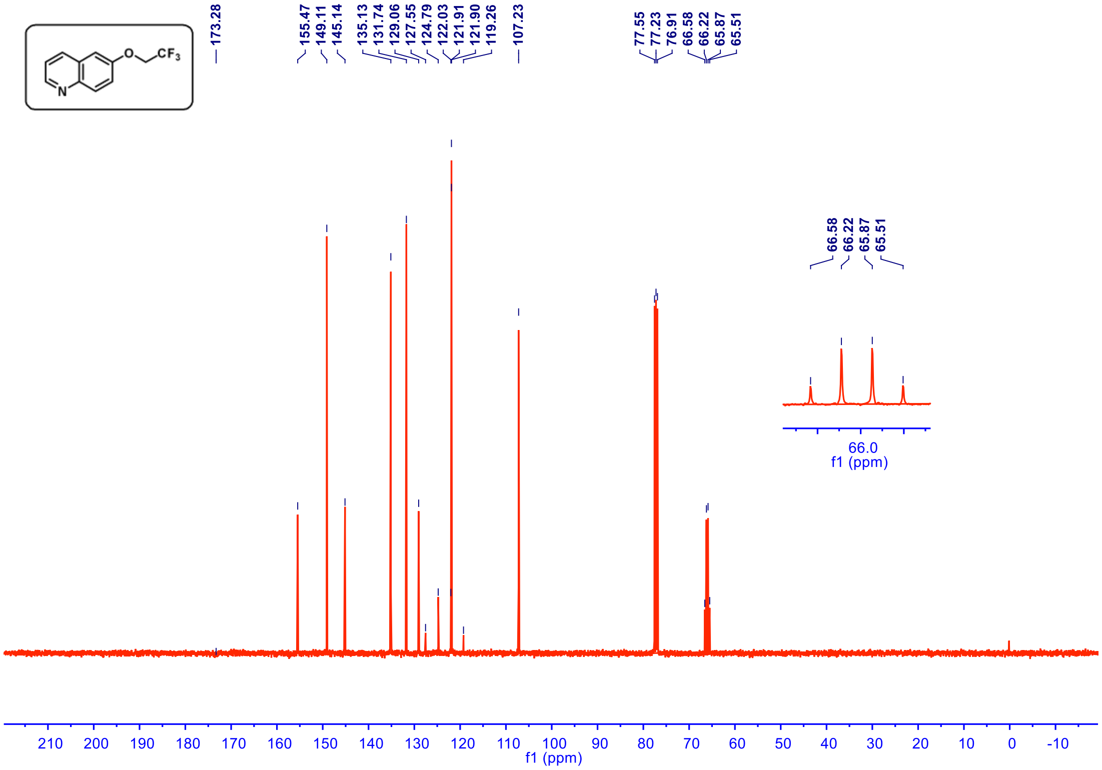

Supplementary Figure 403. ^{13}C NMR spectrum of compound **35** (101 MHz, $CDCl_3$)

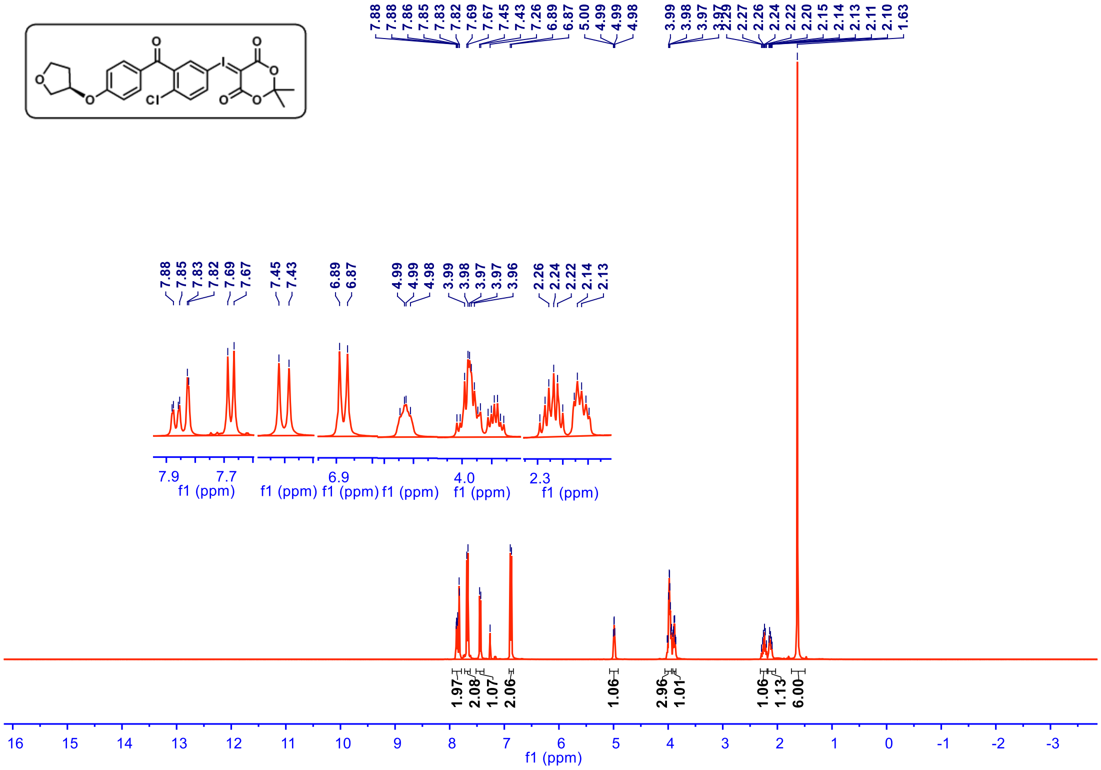

Supplementary Figure 404. ^{1}H NMR spectrum of **(*R*)-5-((4-Chloro-3-(4-((tetrahydrofuran-3-yl)oxy)benzoyl)phenyl) iodonium-(2,2-dimethyl-1,3-dioxane-4,6-dione)ylide** (400 MHz, $CDCl_3$)

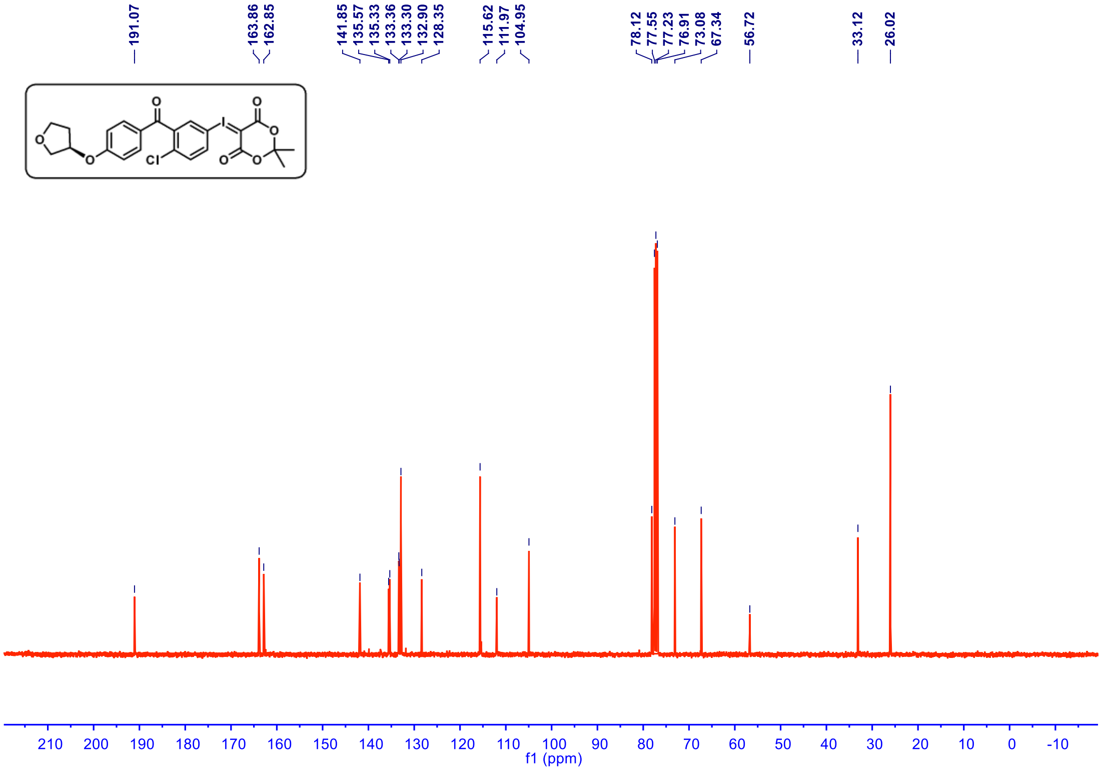

Supplementary Figure 405. ^{13}C NMR spectrum of **(*R*)-5-((4-Chloro-3-(4-((tetrahydrofuran-3-yl)oxy)benzoyl)phenyl) iodonium-(2,2-dimethyl-1,3-dioxane-4,6-dione)ylide** (101 MHz, $CDCl_3$)

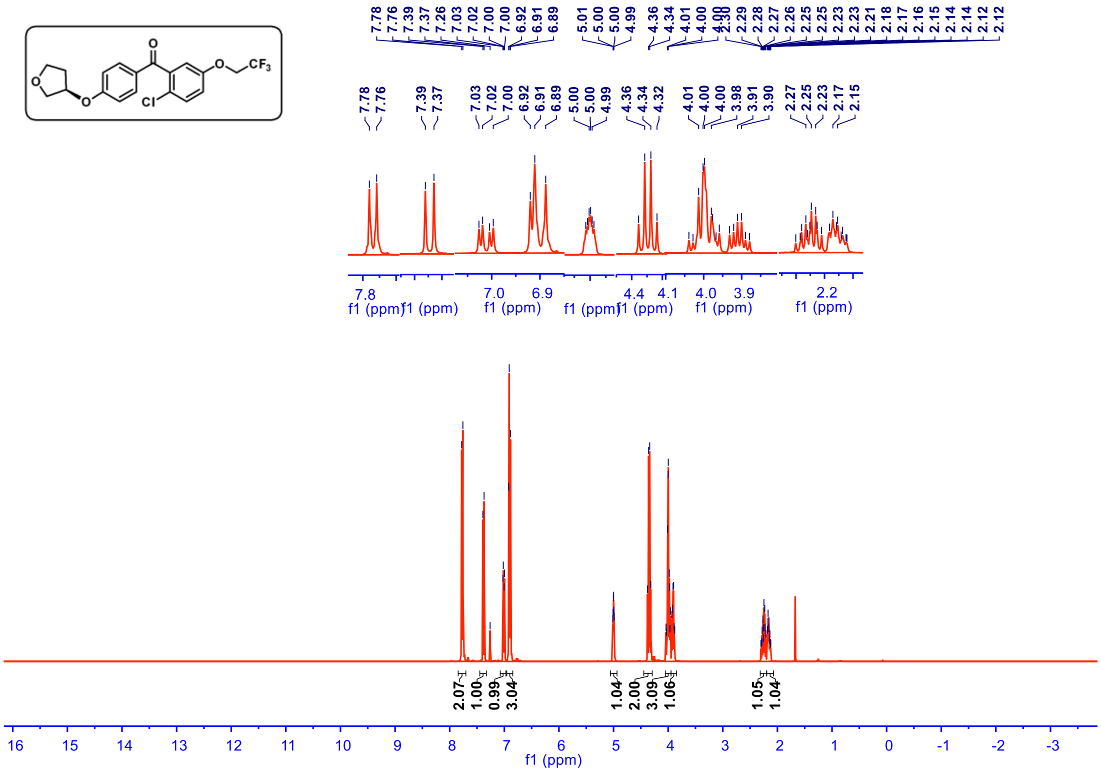

Supplementary Figure 406. ^{1}H NMR spectrum of compound **36** (400 MHz, $CDCl_3$)

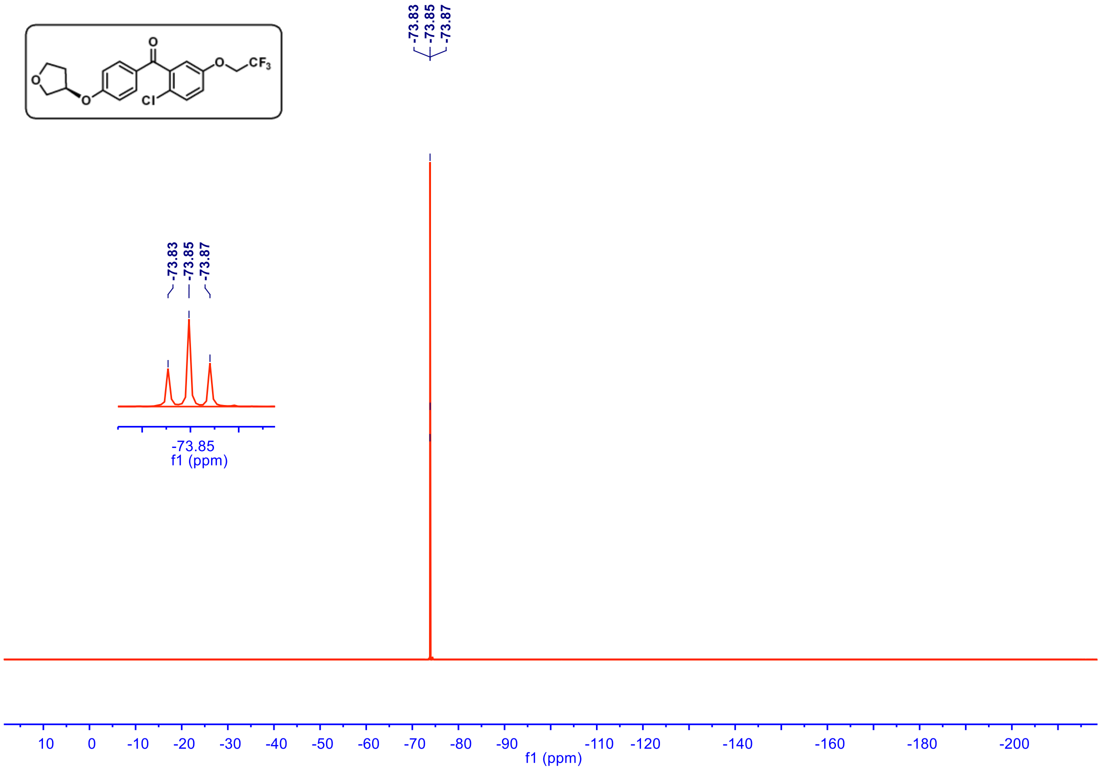

Supplementary Figure 407. ^{19}F NMR spectrum of compound **36** (376 MHz, $CDCl_3$)

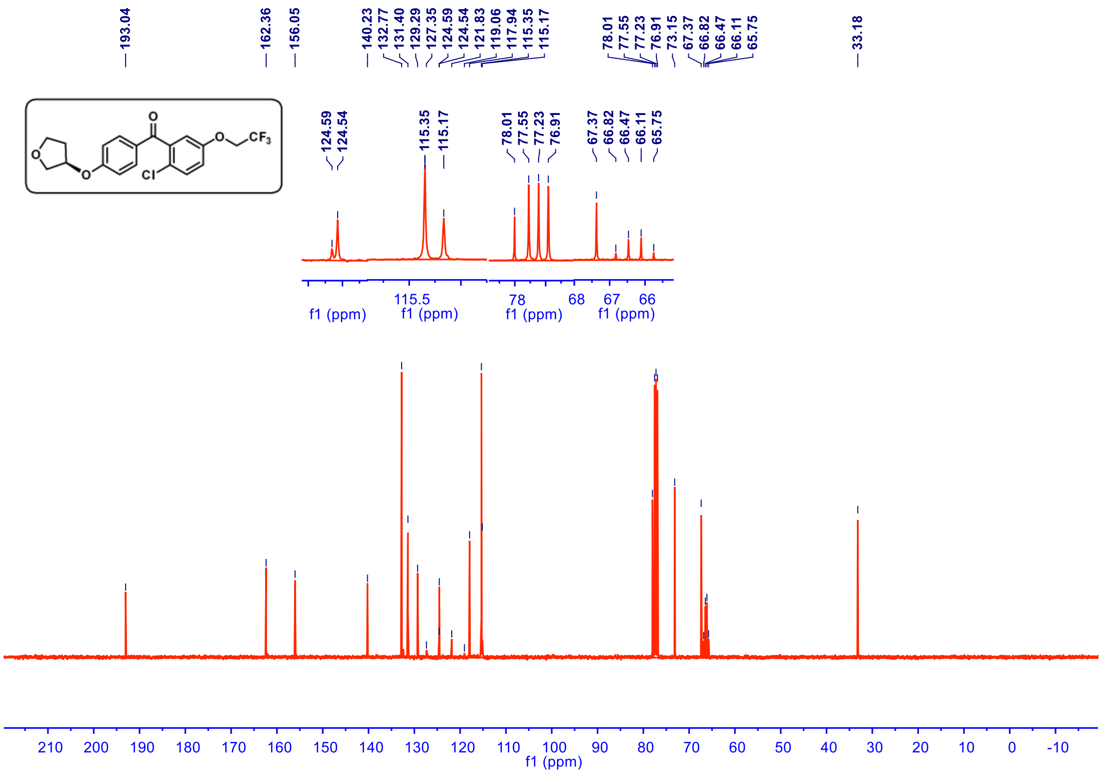

Supplementary Figure 408. ^{13}C NMR spectrum of compound **36** (101 MHz, $CDCl_3$)

^{1}H NMR, ^{19}F NMR and ^{13}C NMR spectra of compound **37**

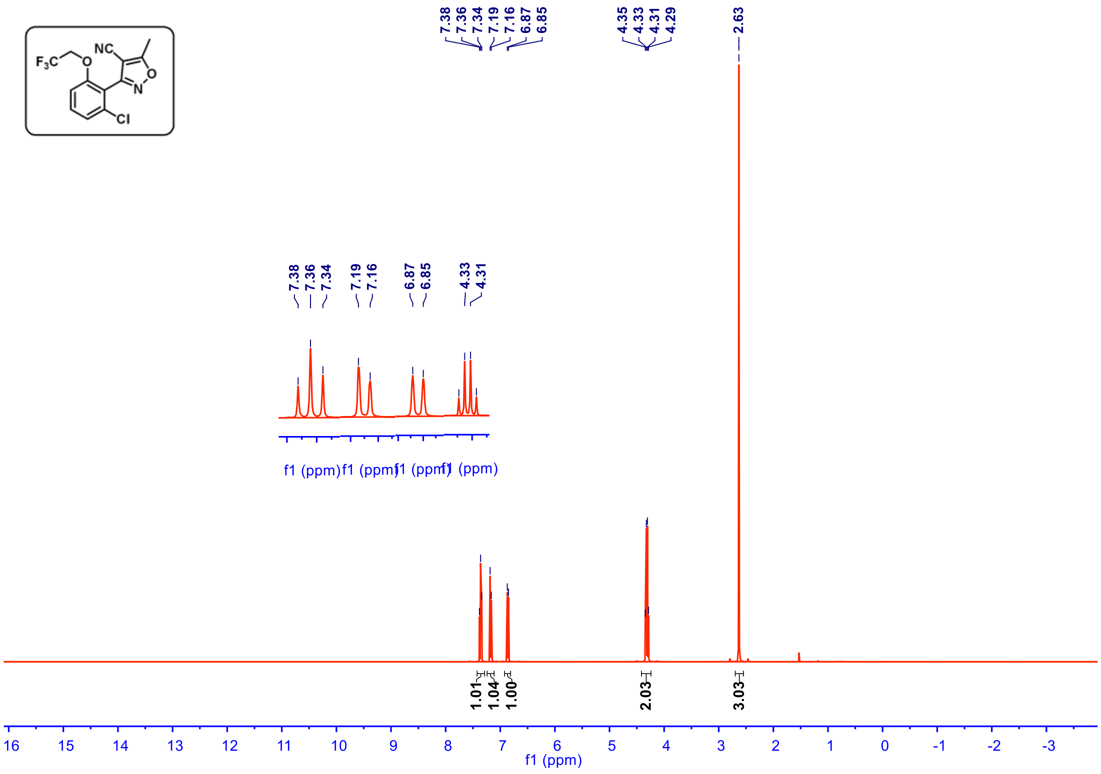

Supplementary Figure 409. ^{1}H NMR spectrum of compound **37** (400 MHz, $CDCl_3$)

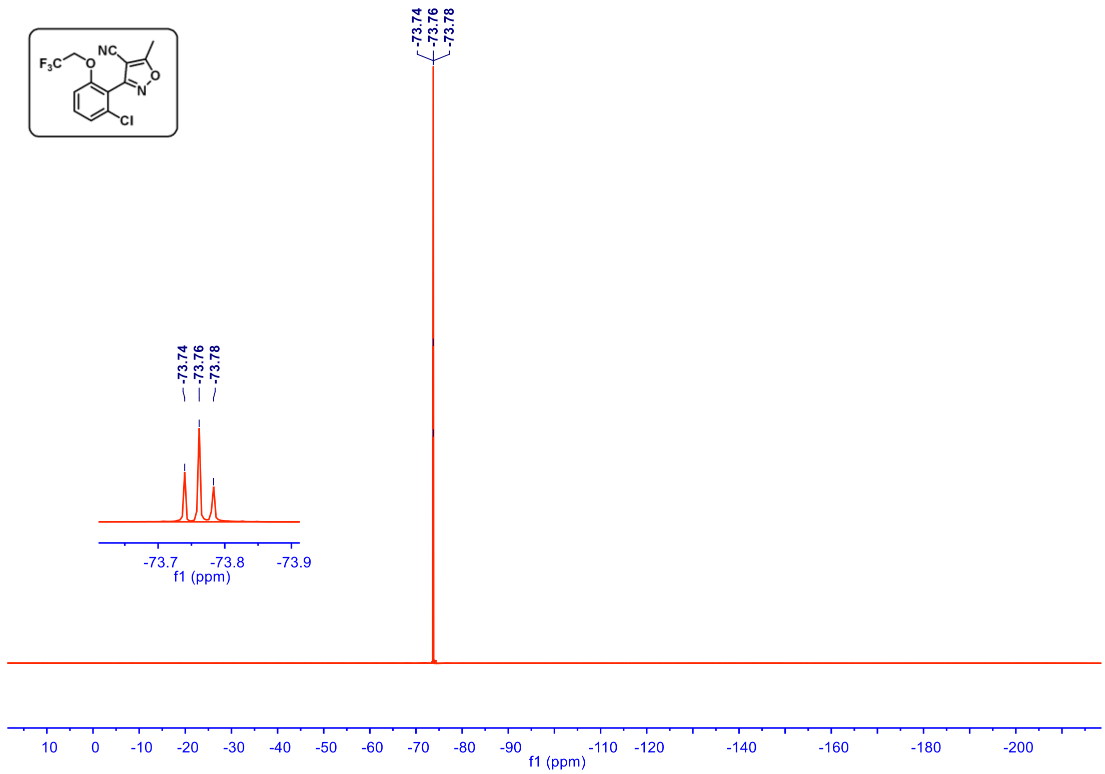

Supplementary Figure 410. ^{19}F NMR spectrum of compound **37** (376 MHz, $CDCl_3$)

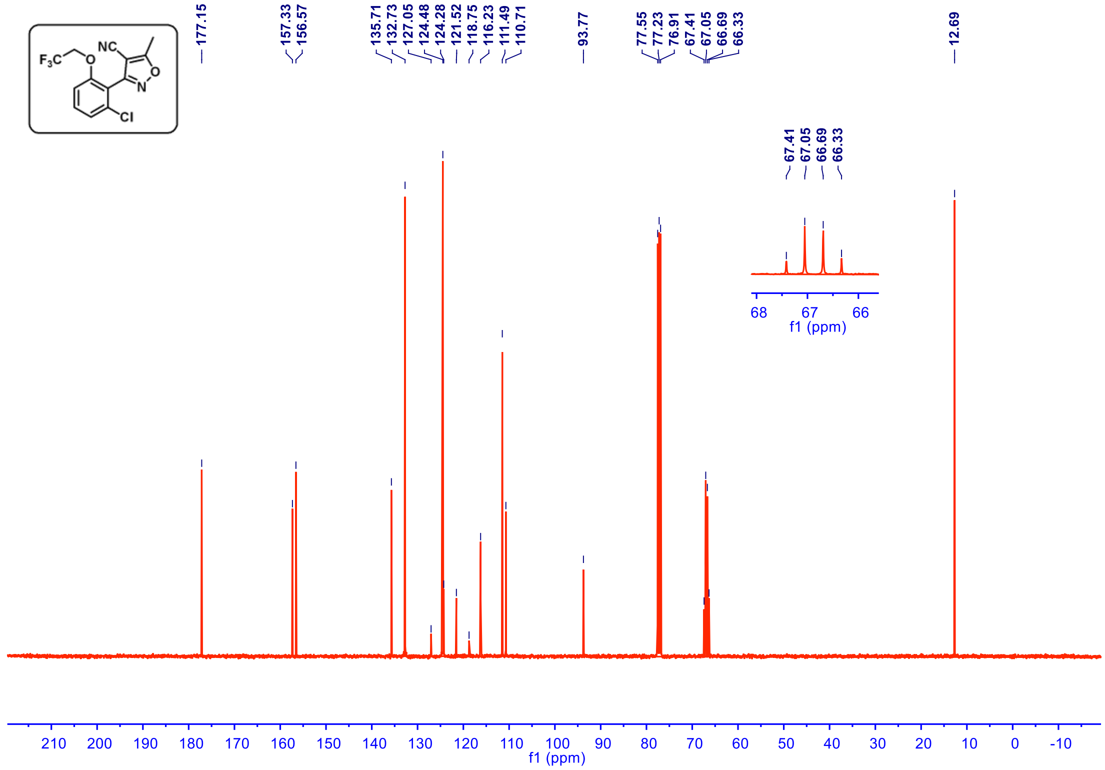

Supplementary Figure 411. ^{13}C NMR spectrum of compound **37** (101 MHz, $CDCl_3$)

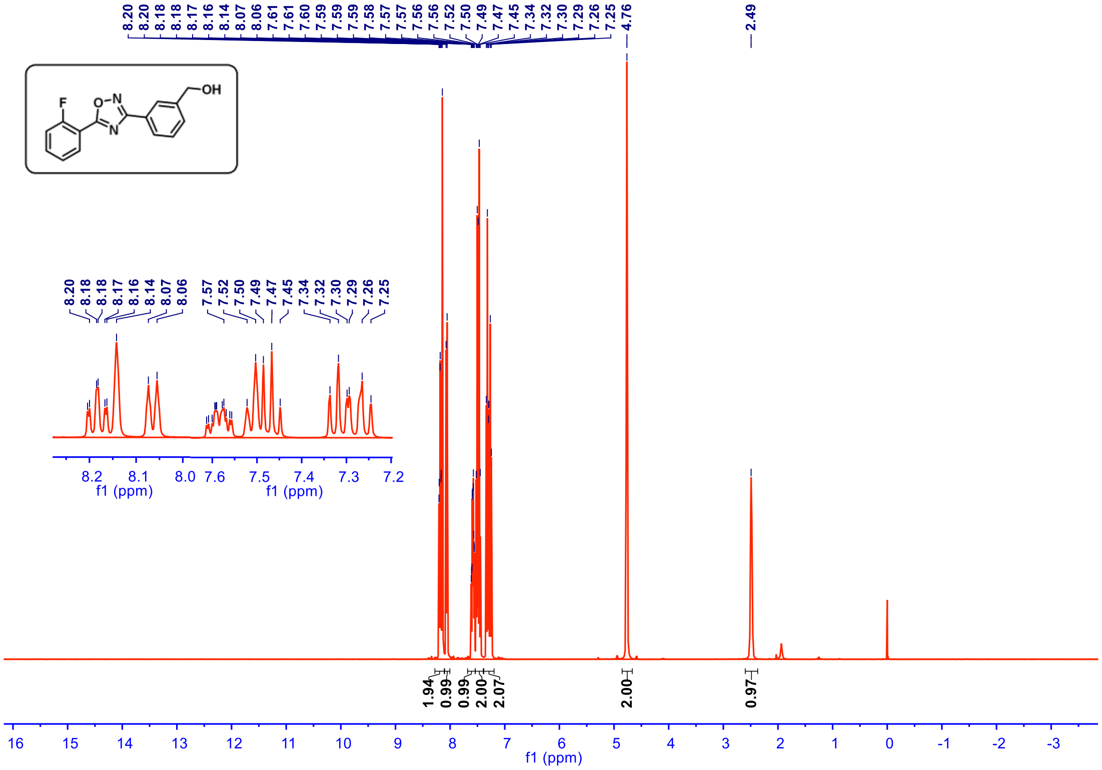

Supplementary Figure 412. ^{1}H NMR spectrum of **(3-(5-(2-fluorophenyl)-1,2,4-oxadiazol-3-yl)phenyl)methanol** (400 MHz, $CDCl_3$)

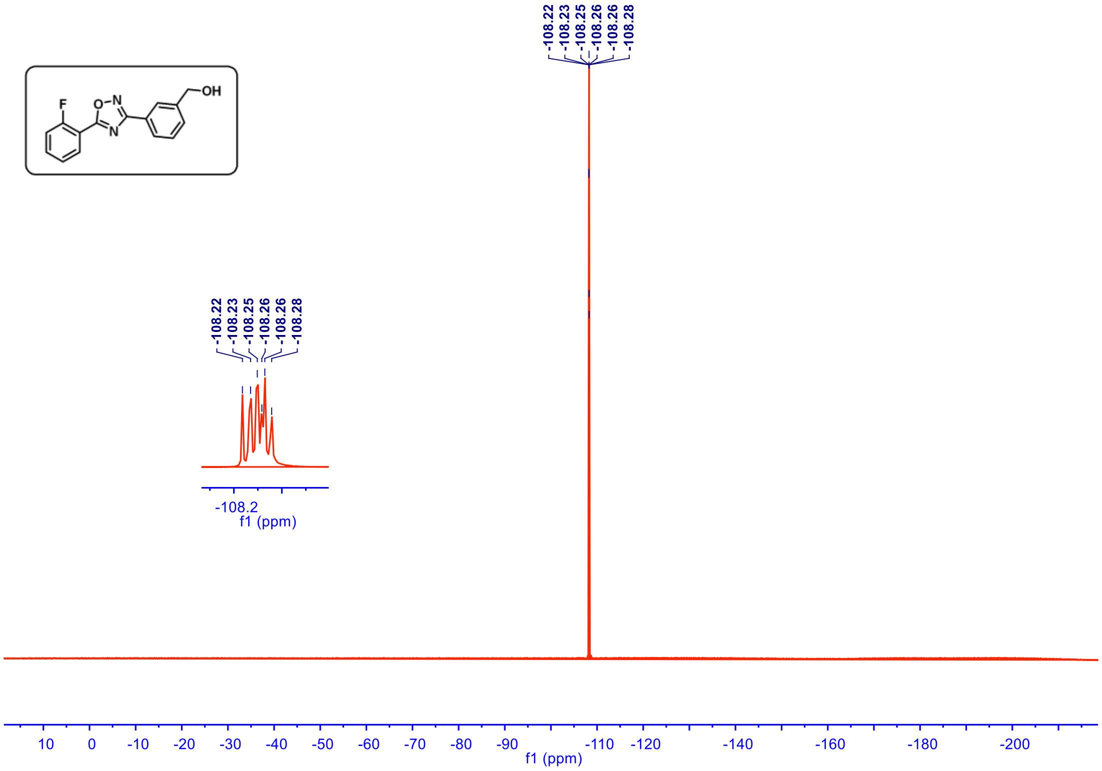

Supplementary Figure 413. ^{19}F NMR spectrum of **(3-(5-(2-fluorophenyl)-1,2,4-oxadiazol-3-yl)phenyl)methanol** (376 MHz, $CDCl_3$)

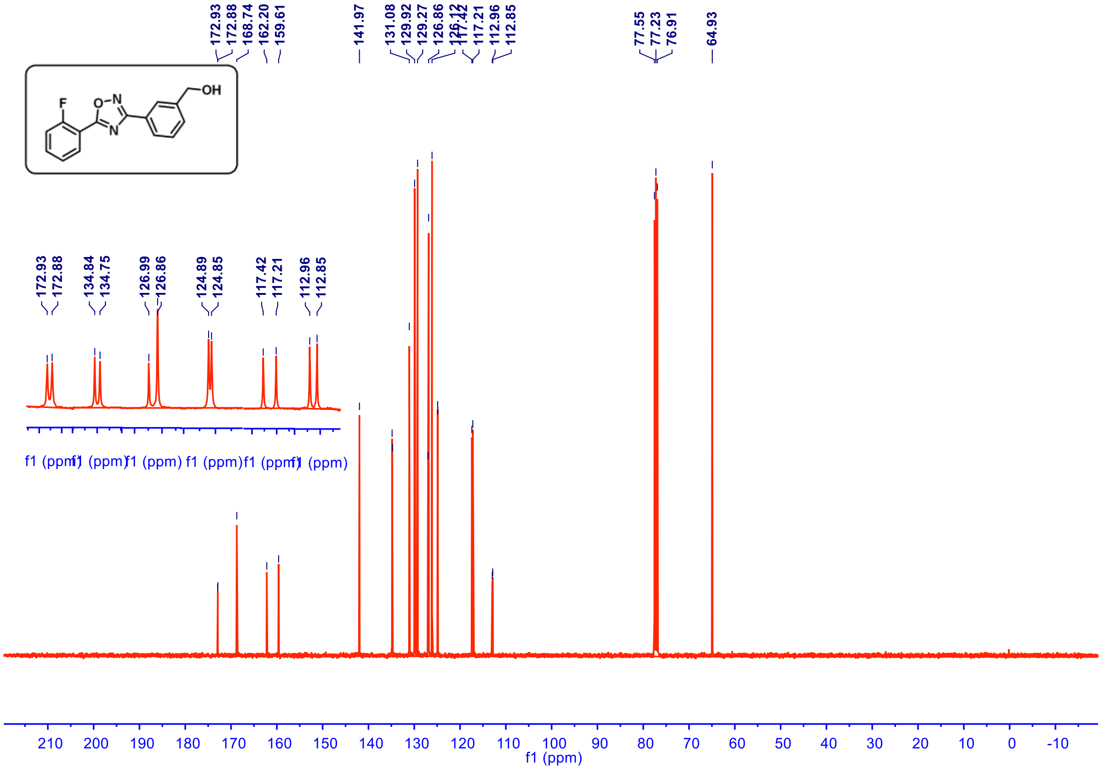

Supplementary Figure 414. ^{13}C NMR spectrum of **(3-(5-(2-fluorophenyl)-1,2,4-oxadiazol-3-yl)phenyl)methanol** (101 MHz, $CDCl_3$)

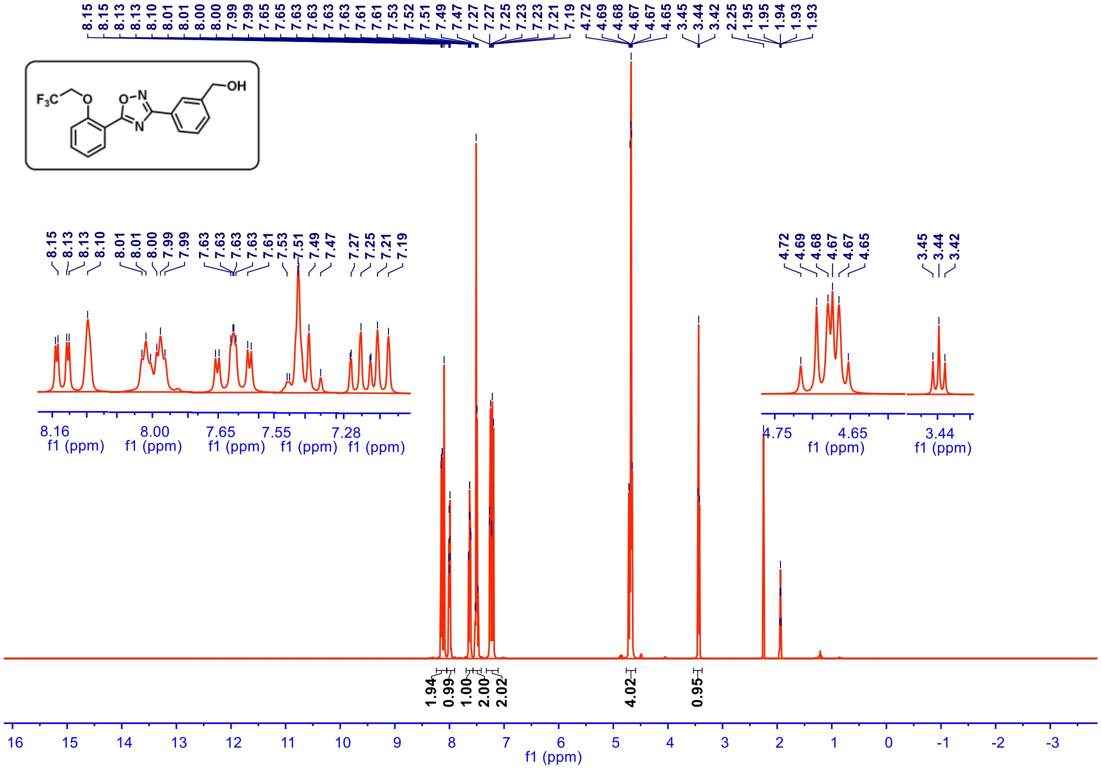

Supplementary Figure 415. ^{1}H NMR spectrum of compound **38** (400 MHz, $CDCl_3$)

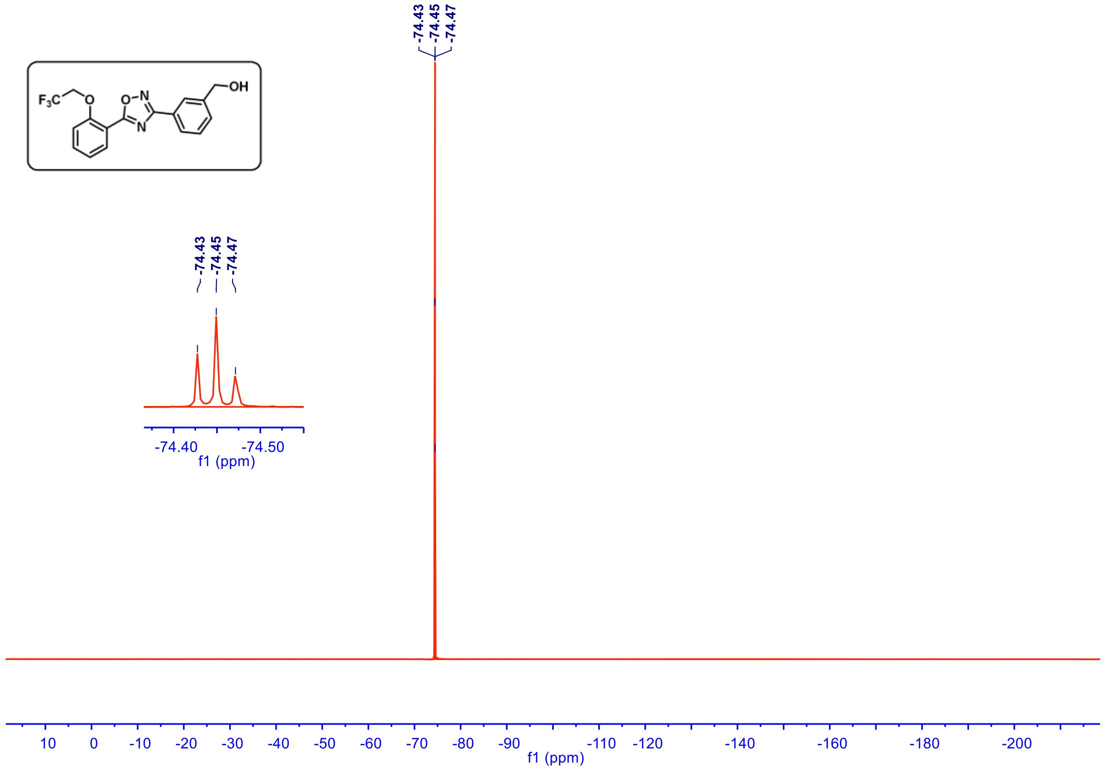

Supplementary Figure 416. ^{19}F NMR spectrum of compound **38** (376 MHz, $CDCl_3$)

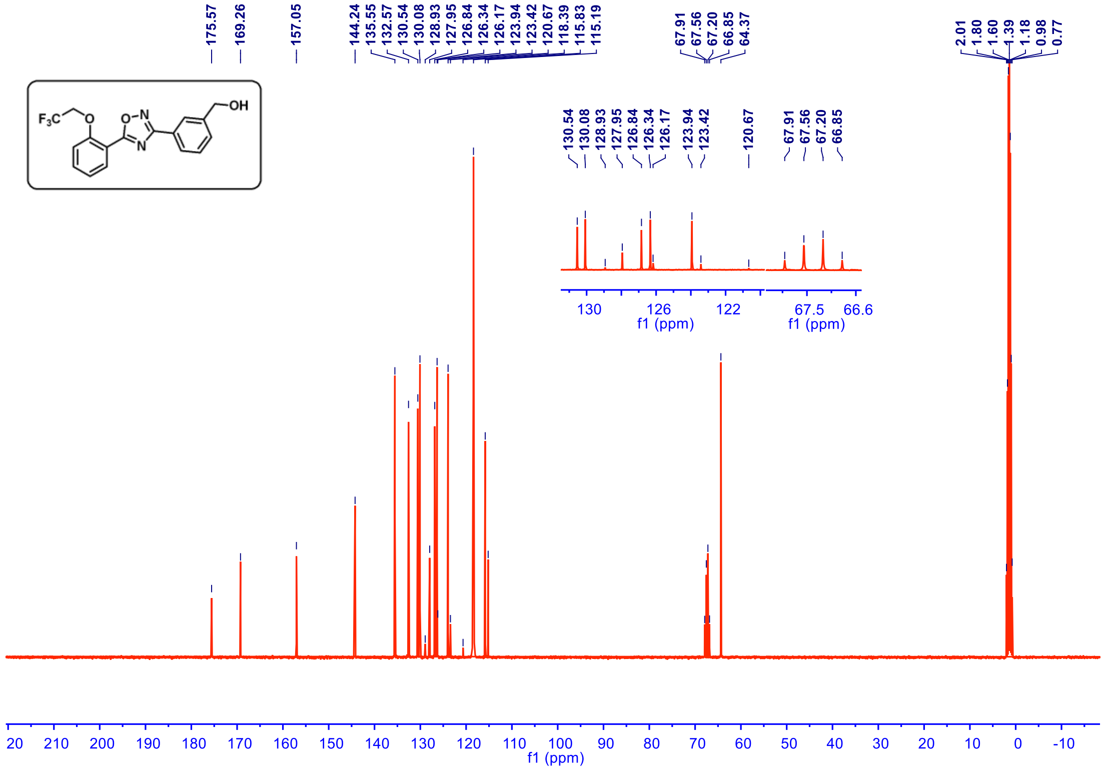

Supplementary Figure 417. ^{13}C NMR spectrum of compound **38** (101 MHz, $CDCl_3$)

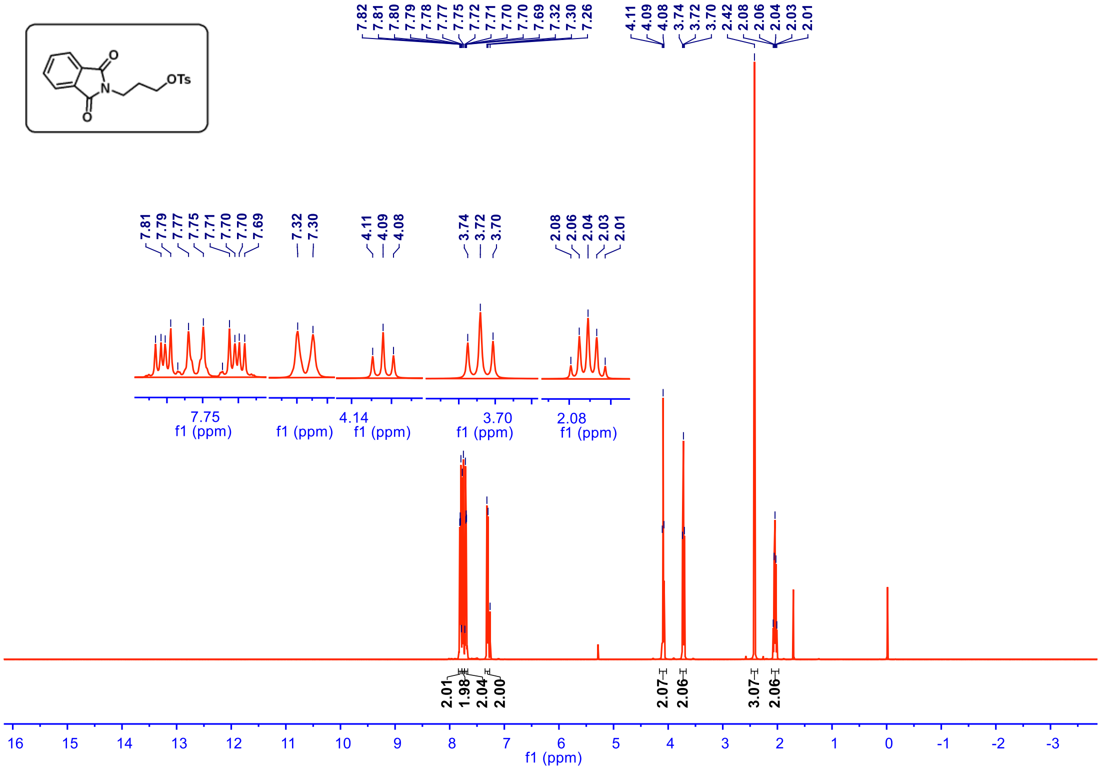

Supplementary Figure 418. ^{1}H NMR spectrum of **3-(1,3-dioxoisoindolin-2-yl)propyl 4-methylbenzenesulfonate** (400 MHz, $CDCl_3$)

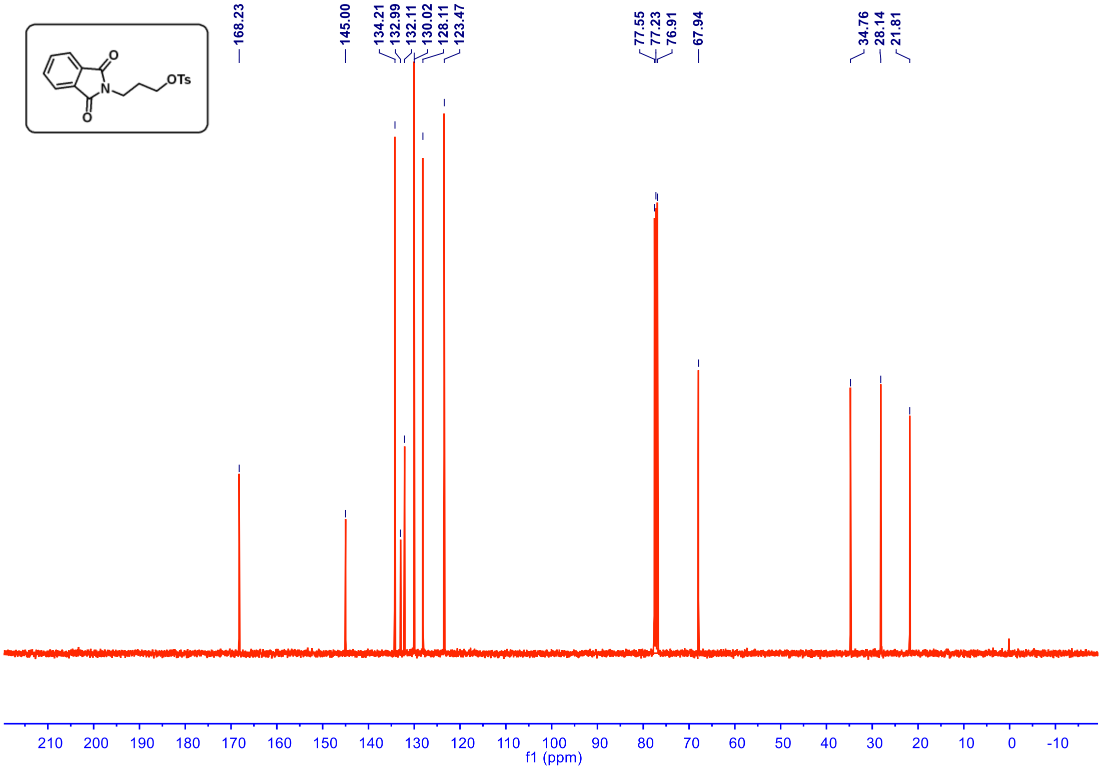

Supplementary Figure 419. ^{13}C NMR spectrum of **3-(1,3-dioxoisoindolin-2-yl)propyl 4-methylbenzenesulfonate** (101 MHz, $CDCl_3$)

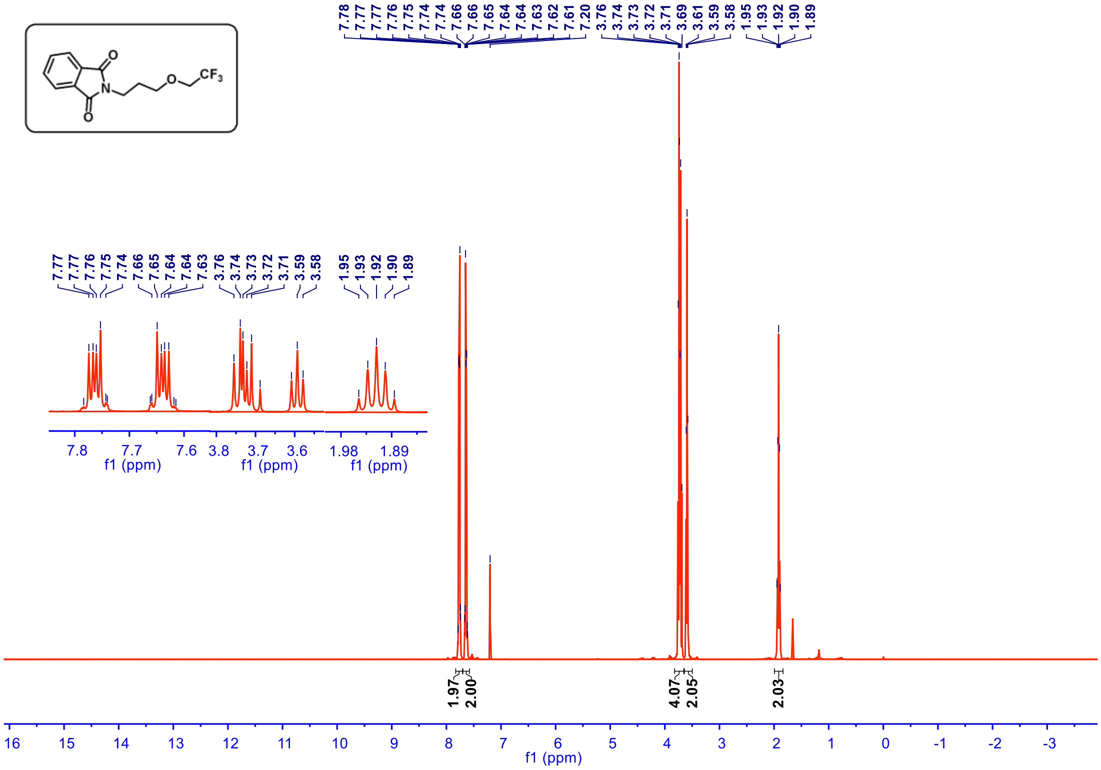

Supplementary Figure 420. ^{1}H NMR spectrum of compound **39** (400 MHz, $CDCl_3$)

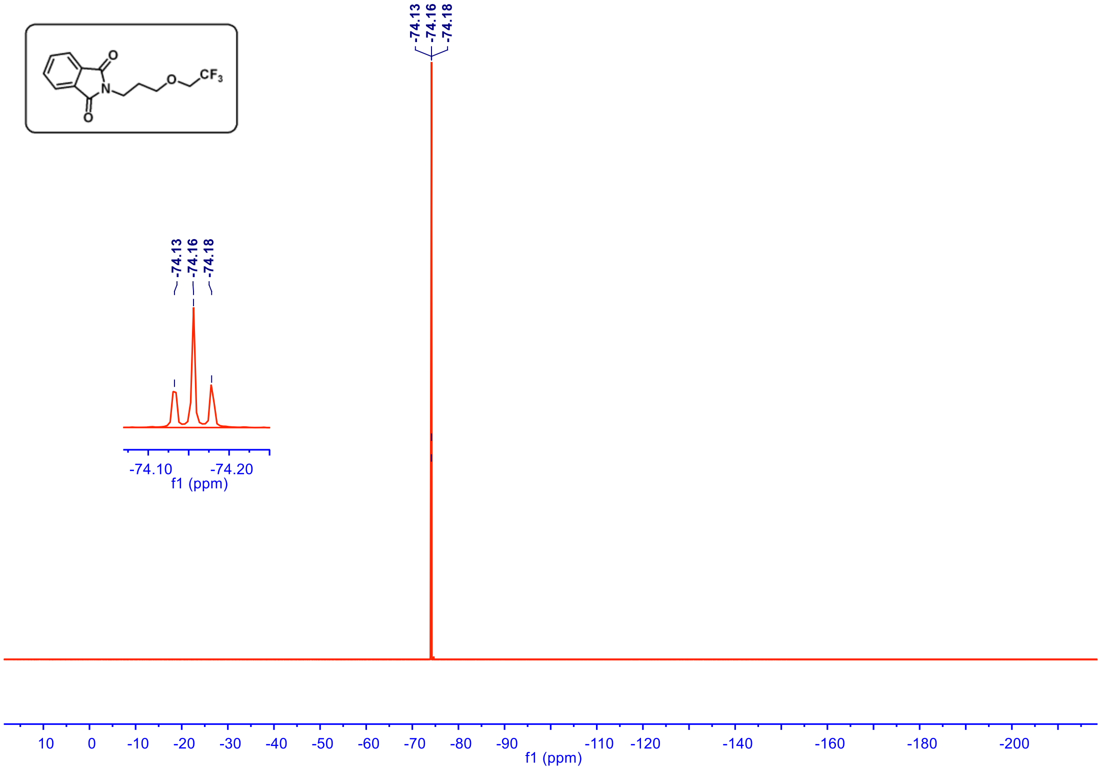

Supplementary Figure 421. ^{19}F NMR spectrum of compound **39** (376 MHz, $CDCl_3$)

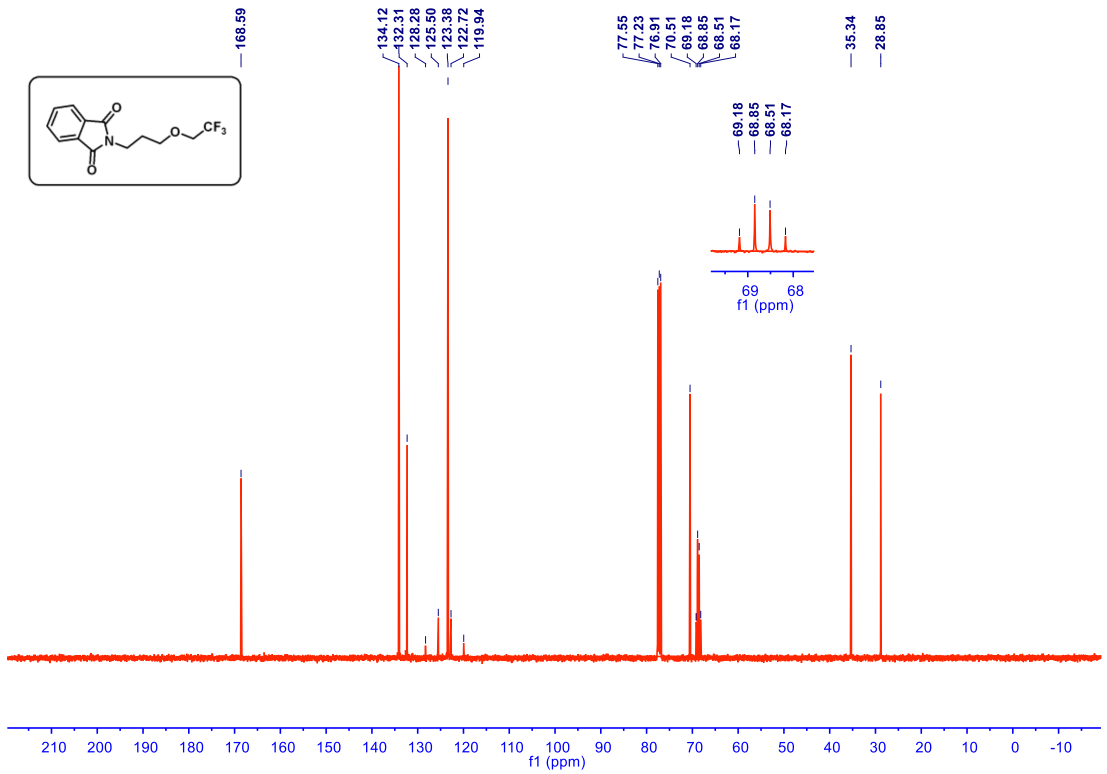

Supplementary Figure 422. ^{13}C NMR spectrum of compound **39** (101 MHz, $CDCl_3$)

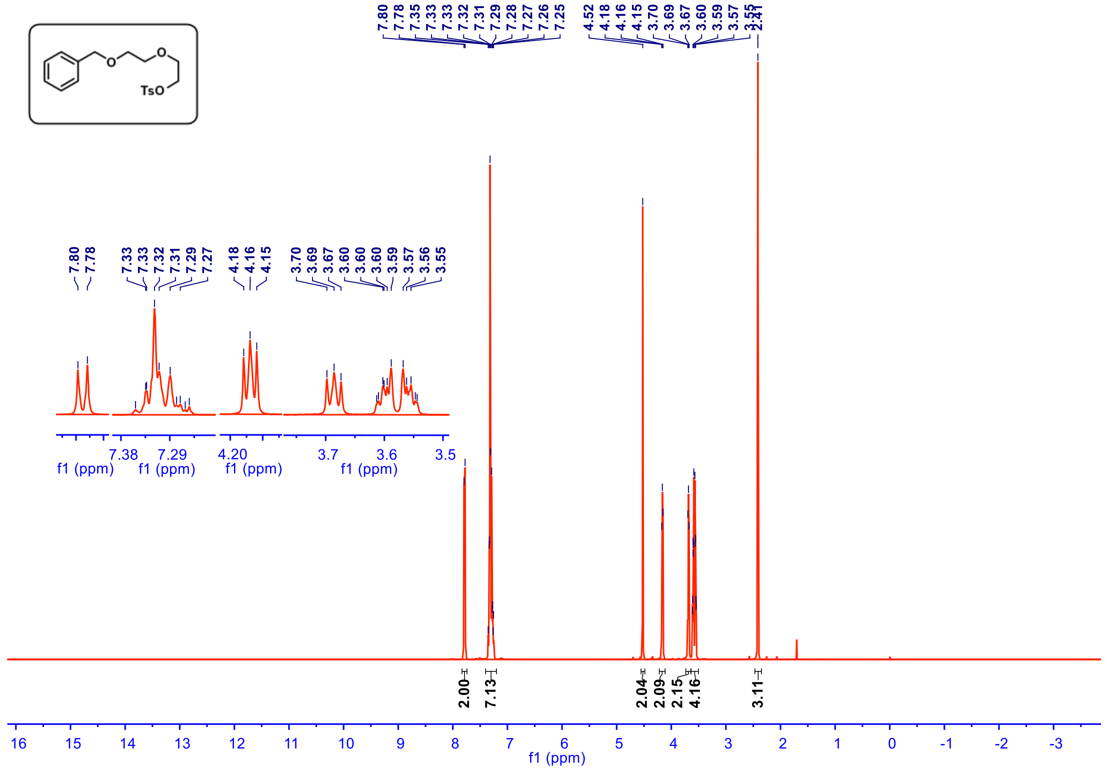

Supplementary Figure 423. ^{1}H NMR spectrum of **2-(2-(benzyloxy)ethoxy)ethyl 4-methylbenzenesulfonate** (400 MHz, $CDCl_3$)

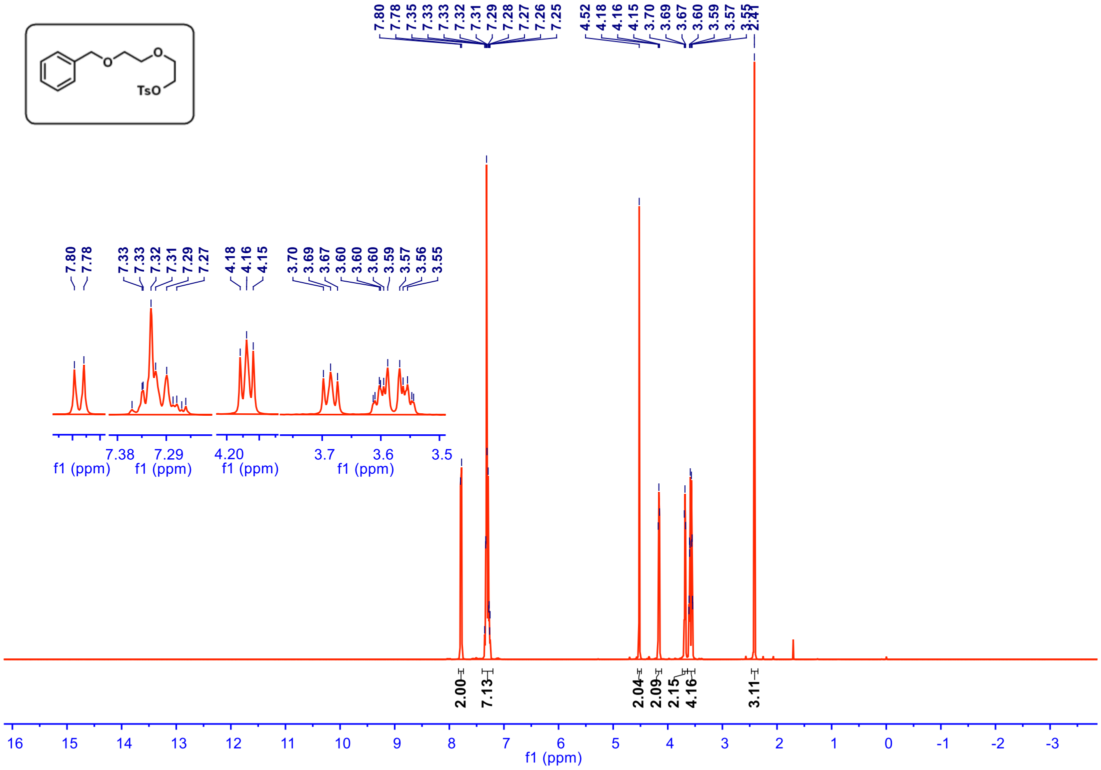

Supplementary Figure 424. ^{13}C NMR spectrum of **2-(2-(benzyloxy)ethoxy)ethyl 4-methylbenzenesulfonate** (101 MHz, $CDCl_3$)

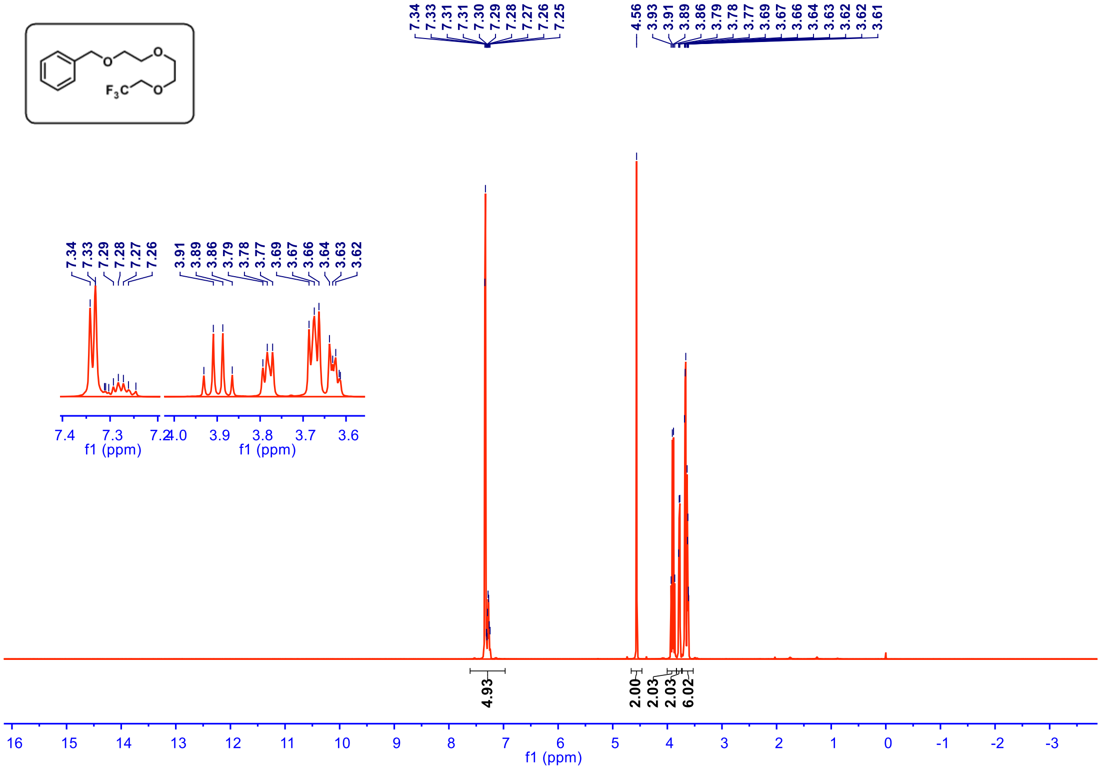

Supplementary Figure 425. ^{1}H NMR spectrum of compound **40** (400 MHz, $CDCl_3$)

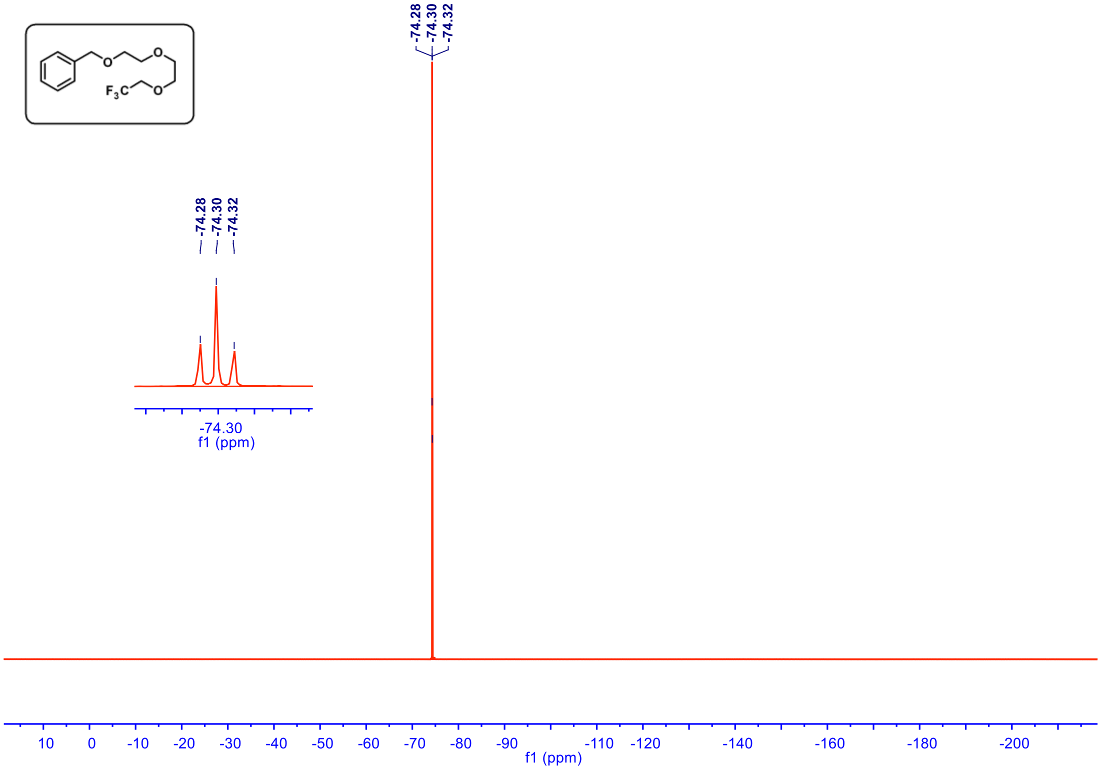

Supplementary Figure 426. ^{19}F NMR spectrum of compound **40** (376 MHz, $CDCl_3$)

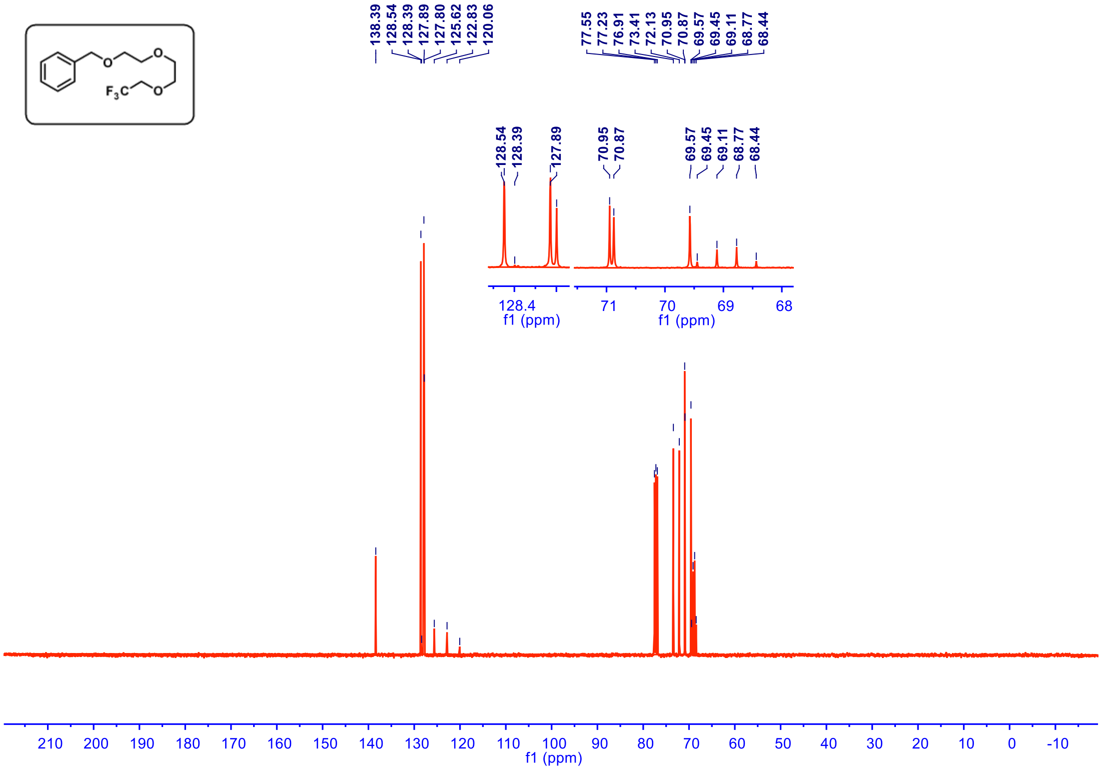

Supplementary Figure 427. ^{13}C NMR spectrum of compound **40** (101 MHz, $CDCl_3$)

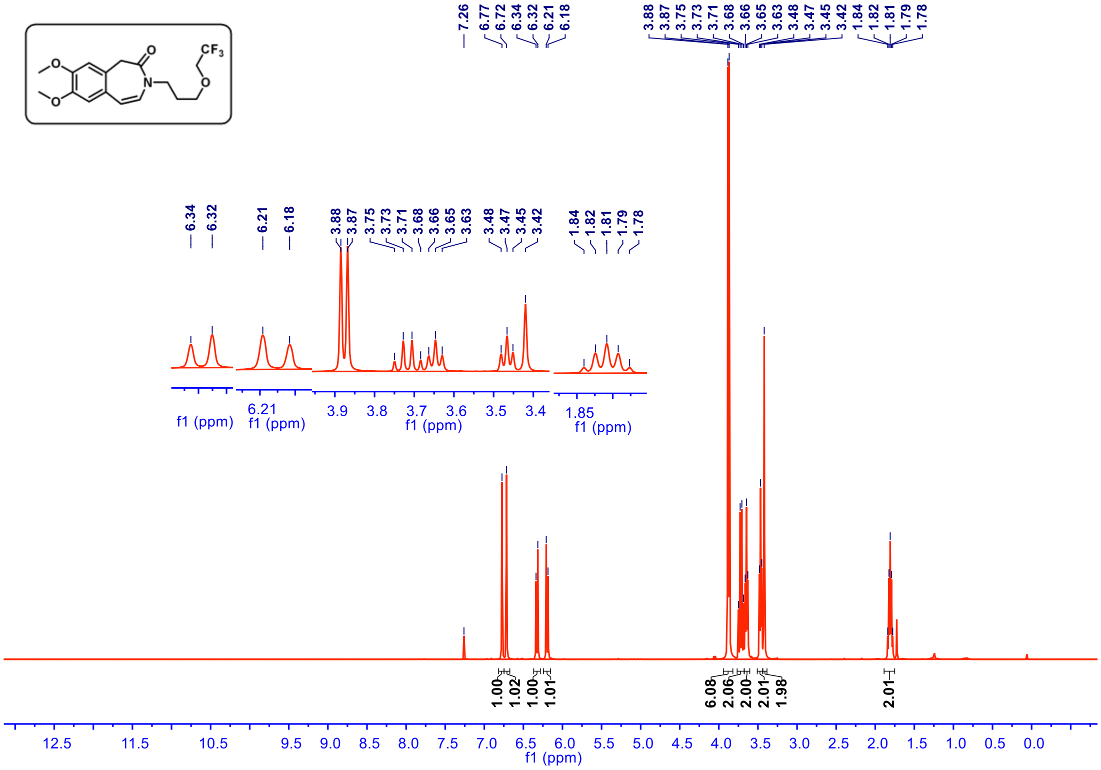

Supplementary Figure 428. ^{1}H NMR spectrum of compound **41** (400 MHz, $CDCl_3$)

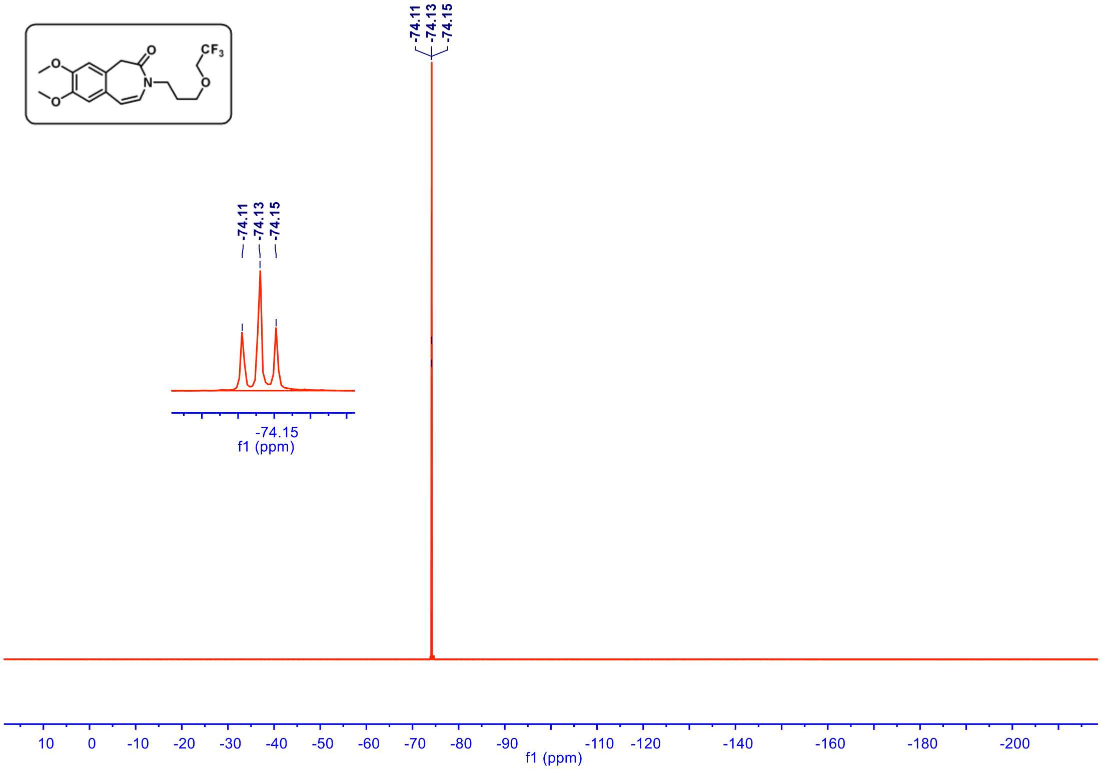

Supplementary Figure 429. ^{19}F NMR spectrum of compound **41** (376 MHz, $CDCl_3$)

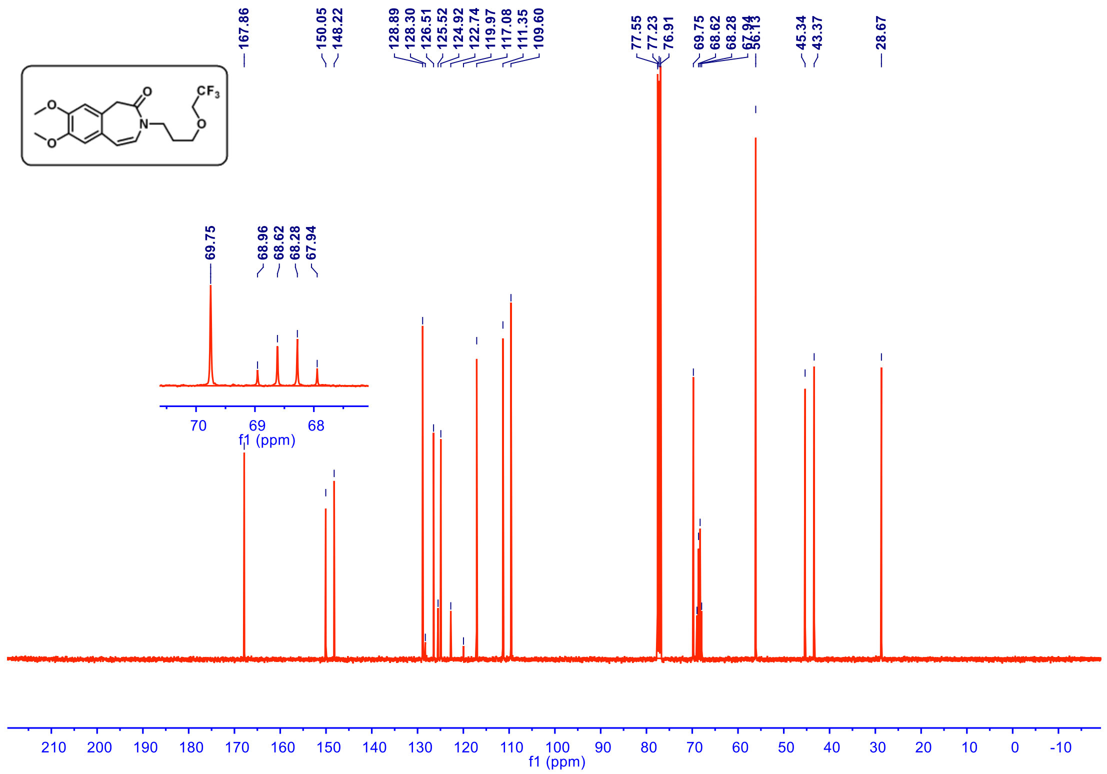

Supplementary Figure 430. ^{13}C NMR spectrum of compound **41** (101 MHz, $CDCl_3$)

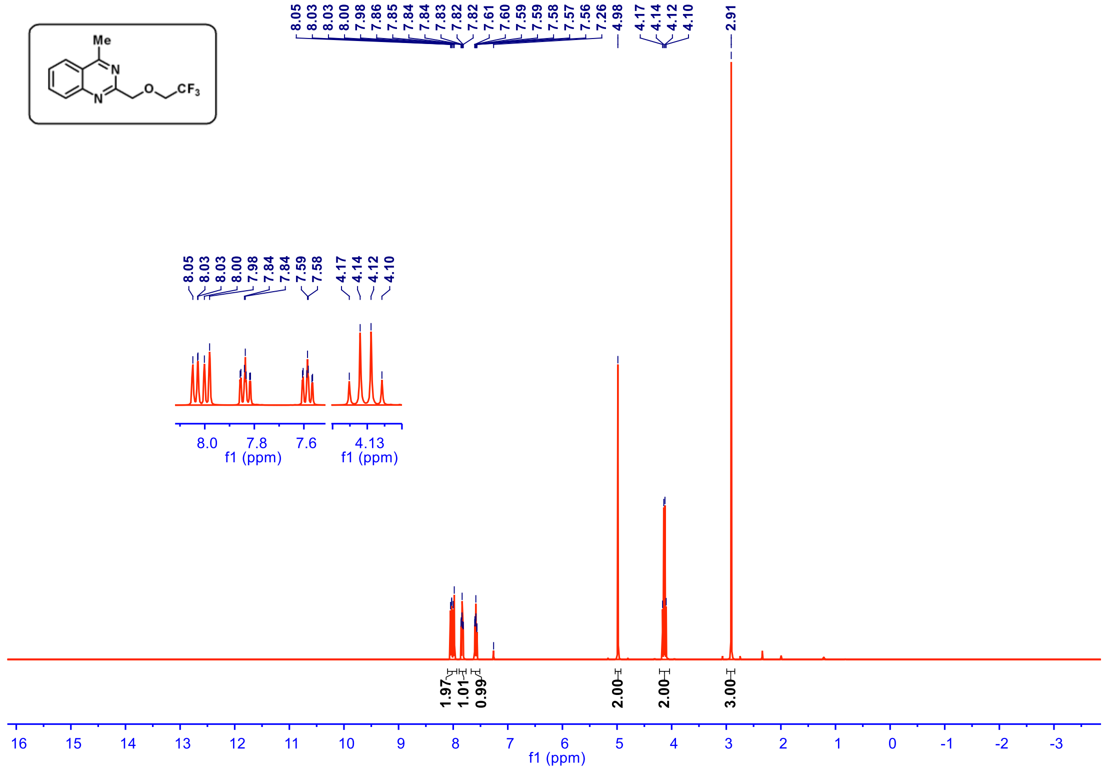

Supplementary Figure 431. ^{1}H NMR spectrum of compound **42** (400 MHz, $CDCl_3$)

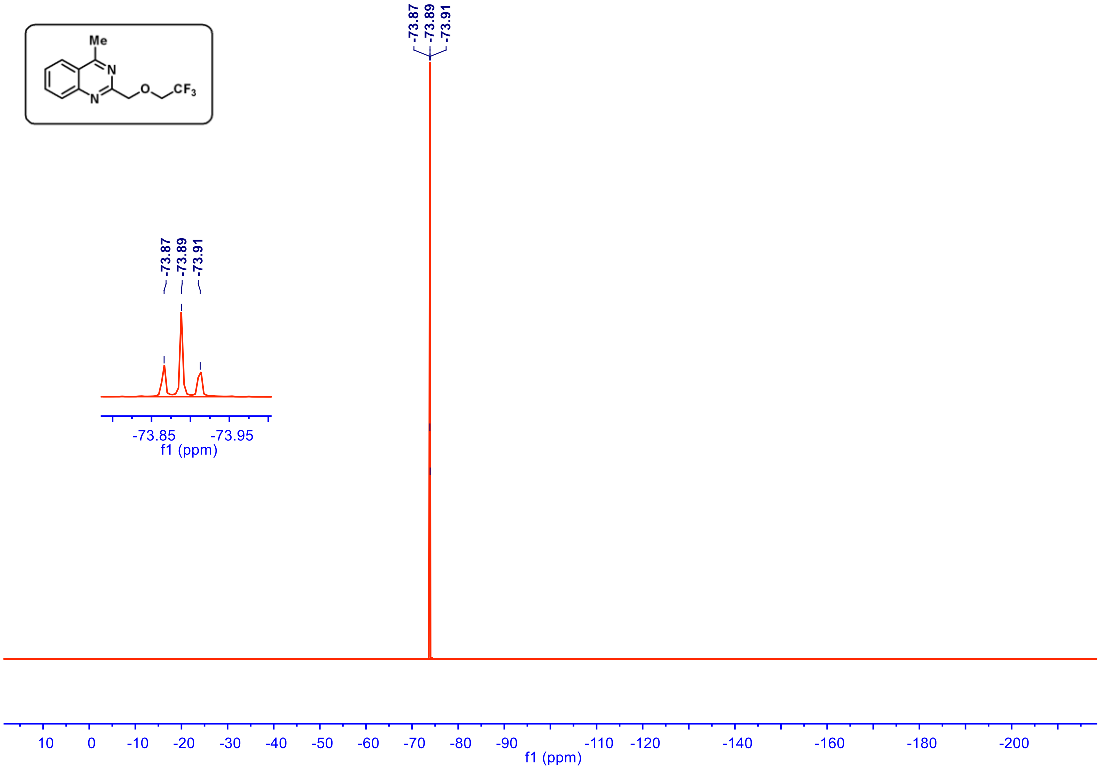

Supplementary Figure 432. ^{19}F NMR spectrum of compound **42** (376 MHz, $CDCl_3$)

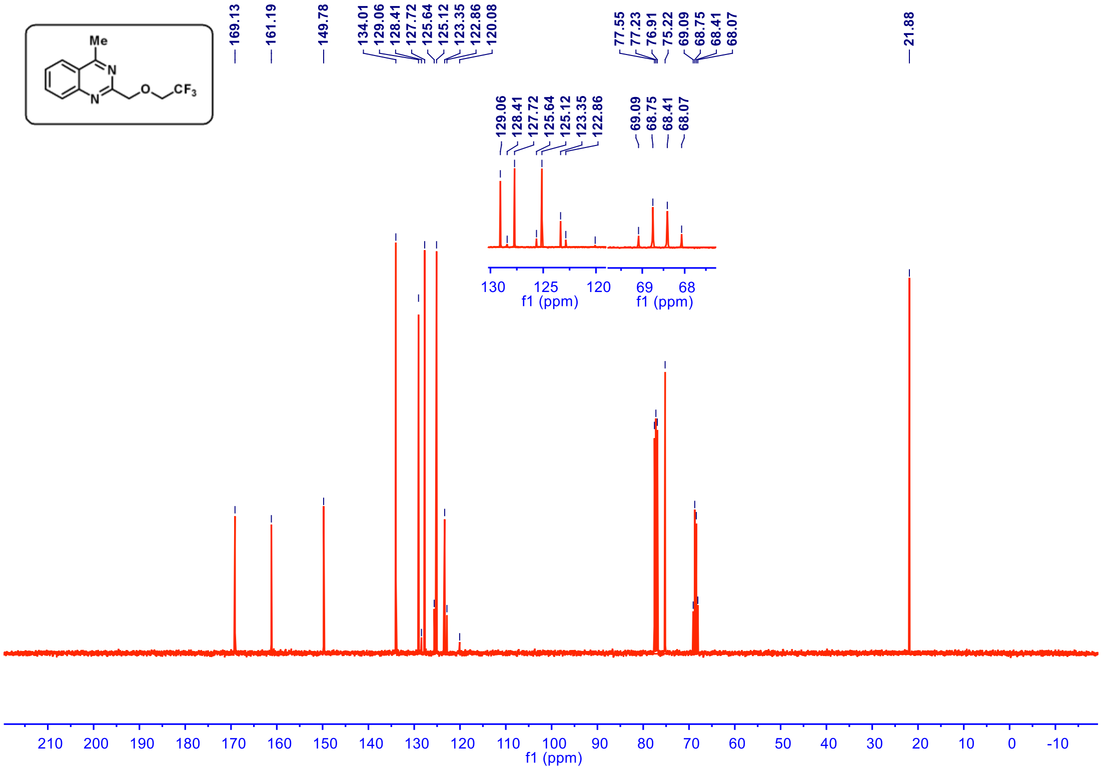

Supplementary Figure 433. ^{13}C NMR spectrum of compound **42** (101 MHz, $CDCl_3$)

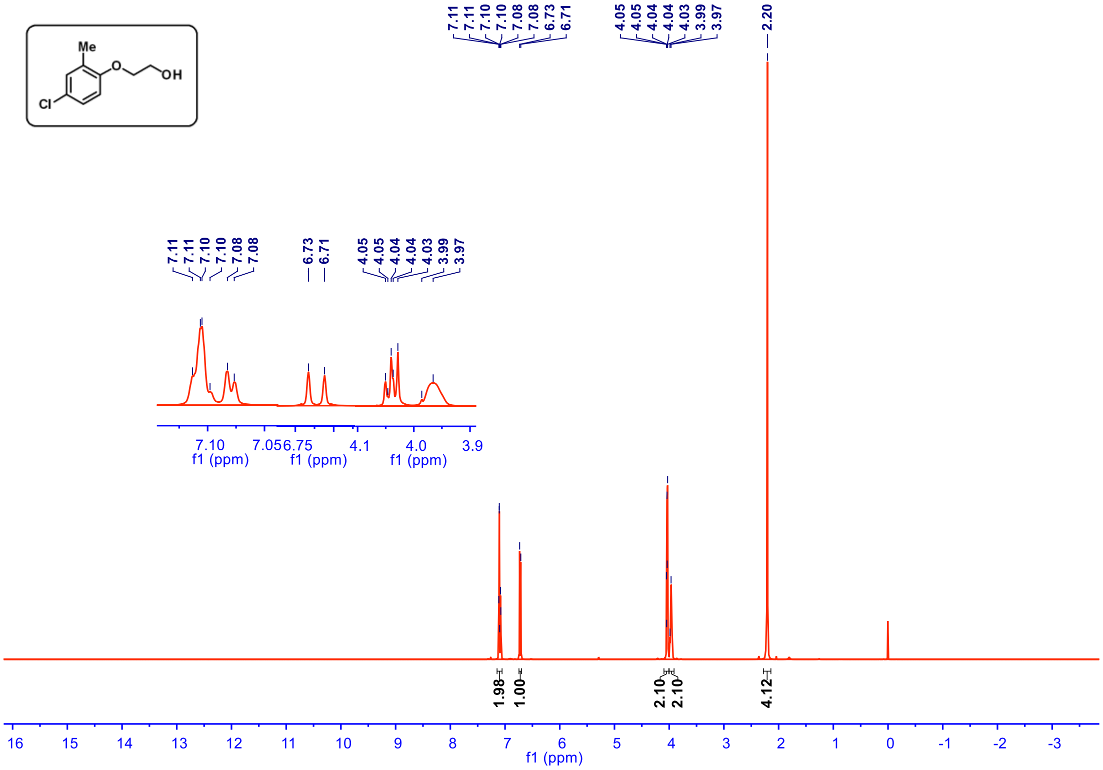

Supplementary Figure 434. ^{1}H NMR spectrum of **2-(4-chloro-2-methylphenoxy)ethan-1-ol** (400 MHz, $CDCl_3$)

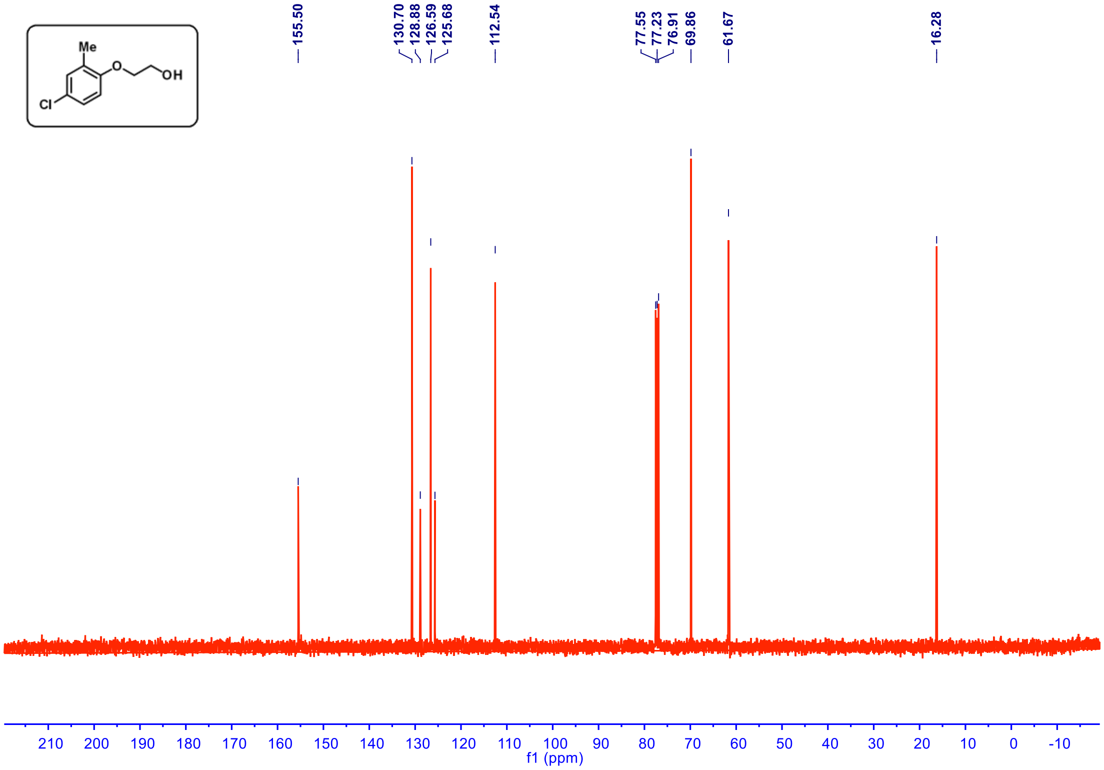

Supplementary Figure 435. ^{13}C NMR spectrum of **2-(4-chloro-2-methylphenoxy)ethan-1-ol** (101 MHz, $CDCl_3$)

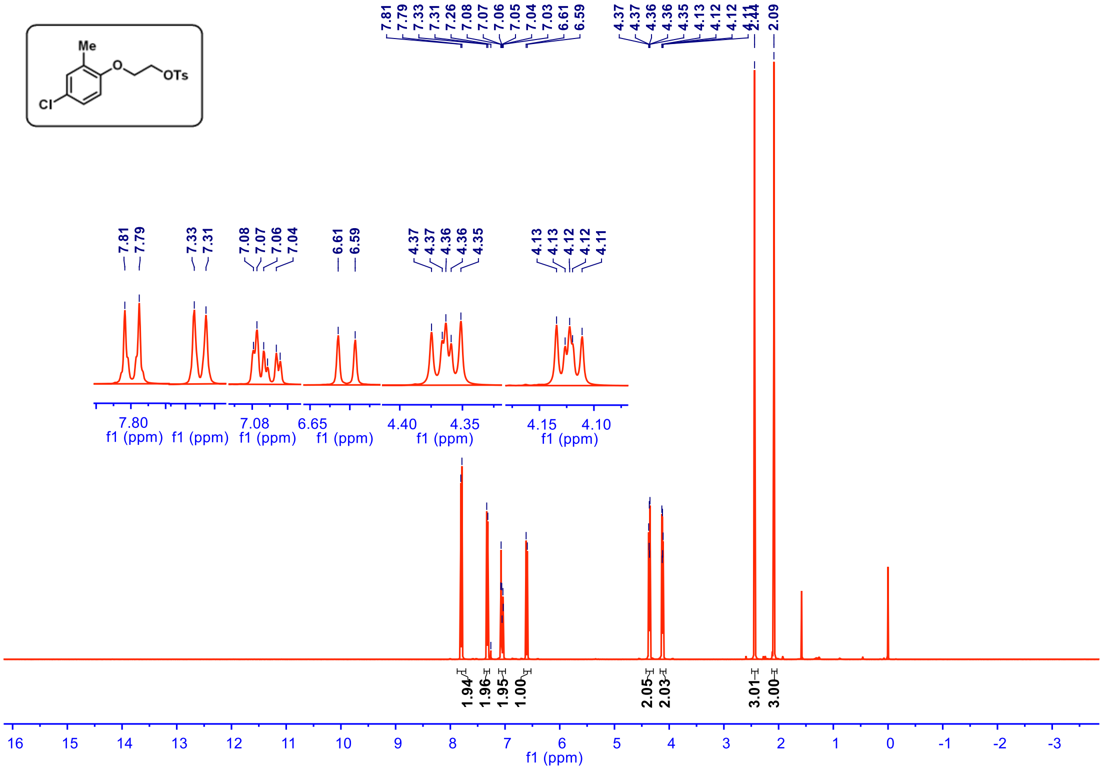

Supplementary Figure 436. ^{1}H NMR spectrum of **2-(4-chloro-2-methylphenoxy)ethyl 4-methylbenzenesulfonate** (400 MHz, $CDCl_3$)

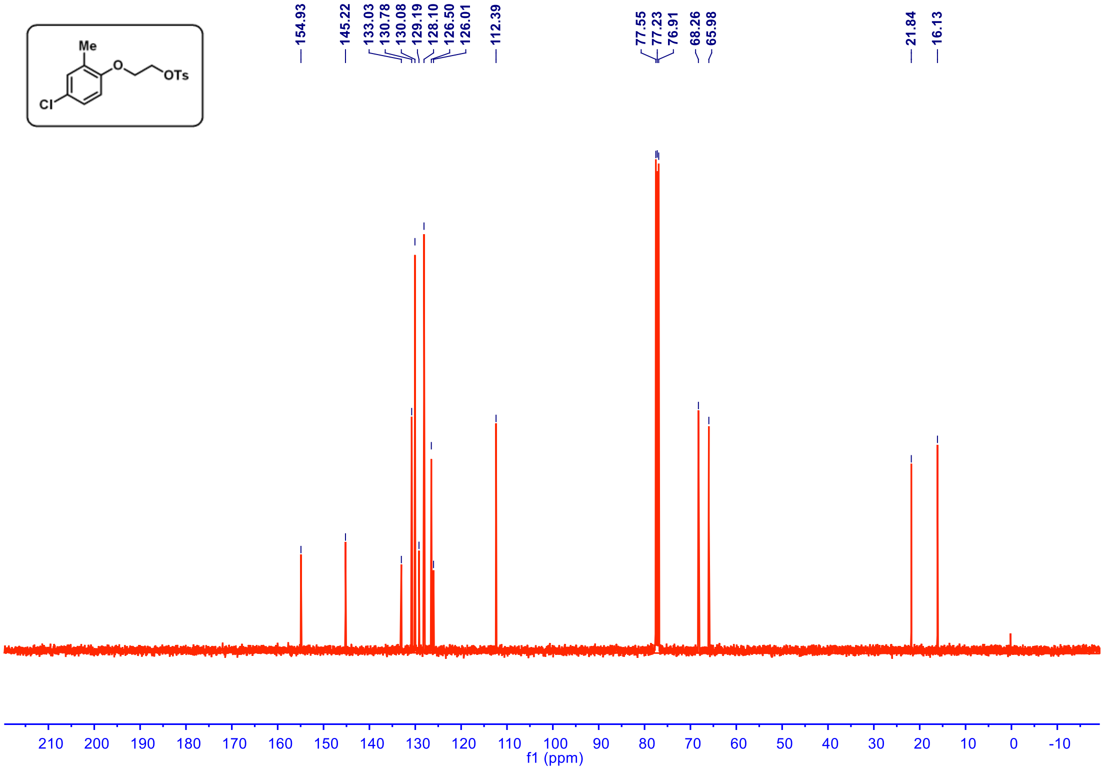

Supplementary Figure 437. ^{13}C NMR spectrum of **2-(4-chloro-2-methylphenoxy)ethyl 4-methylbenzenesulfonate** (101 MHz, $CDCl_3$)

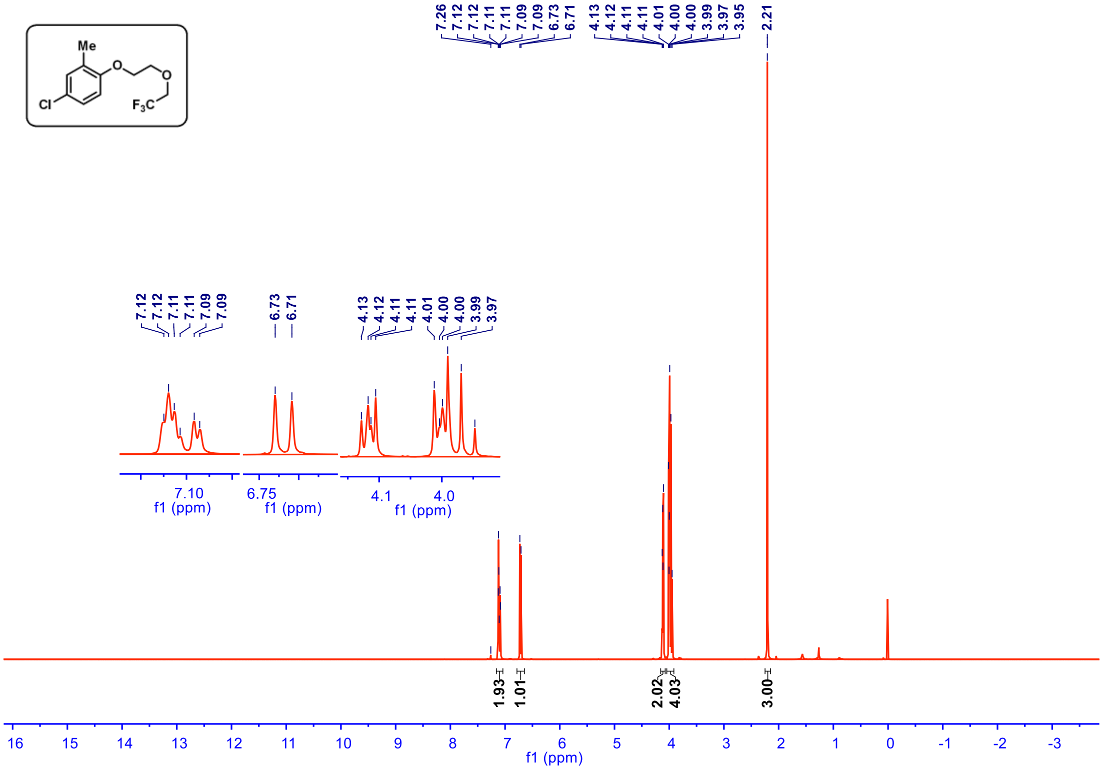

Supplementary Figure 438. ^{1}H NMR spectrum of compound **43** (400 MHz, $CDCl_3$)

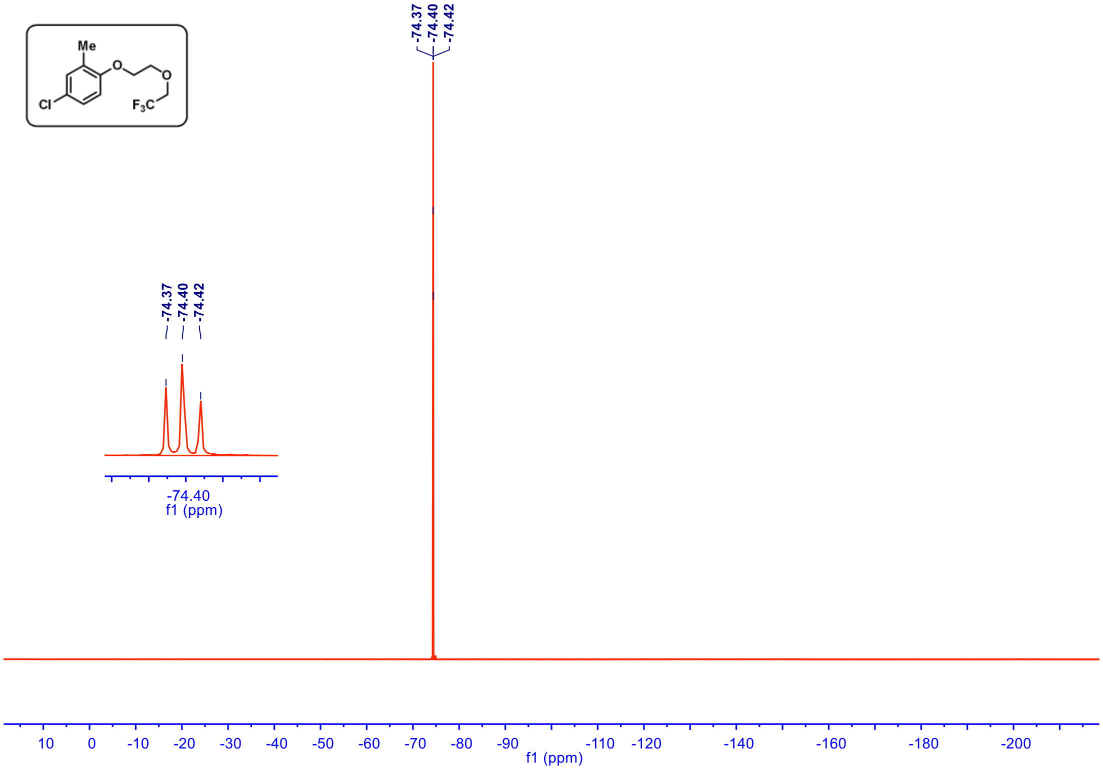

Supplementary Figure 439. ^{19}F NMR spectrum of compound **43** (376 MHz, $CDCl_3$)

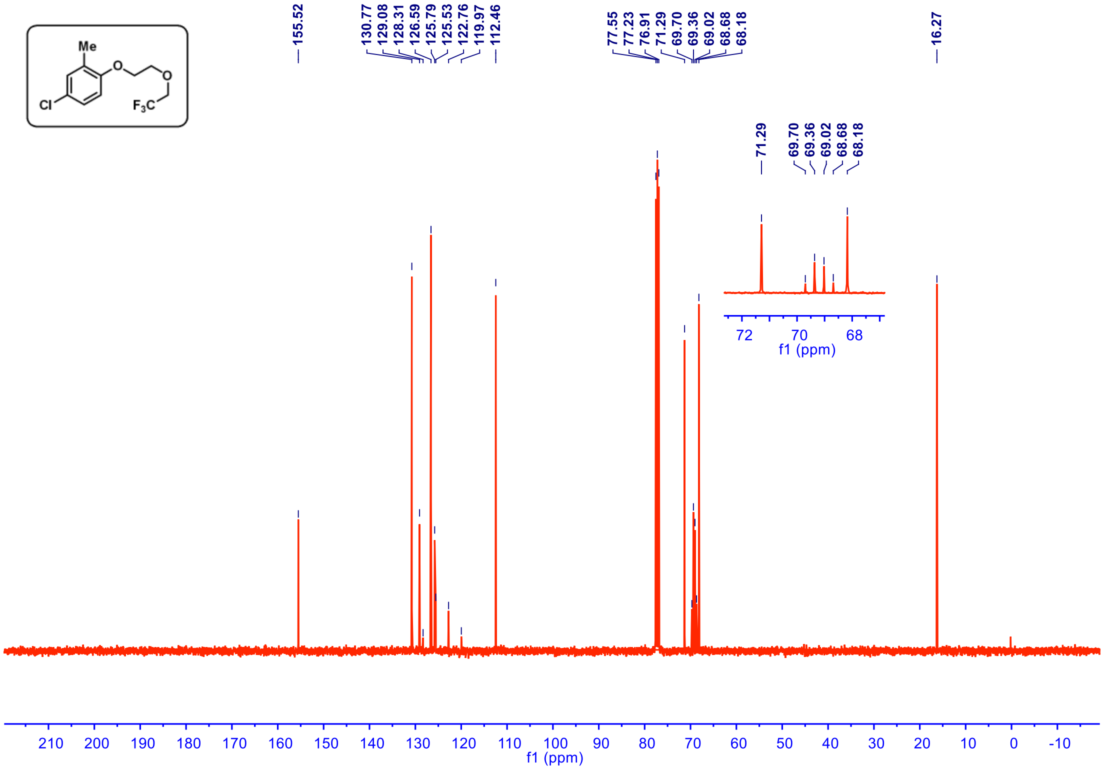

Supplementary Figure 440. ^{13}C NMR spectrum of compound **43** (101 MHz, $CDCl_3$)

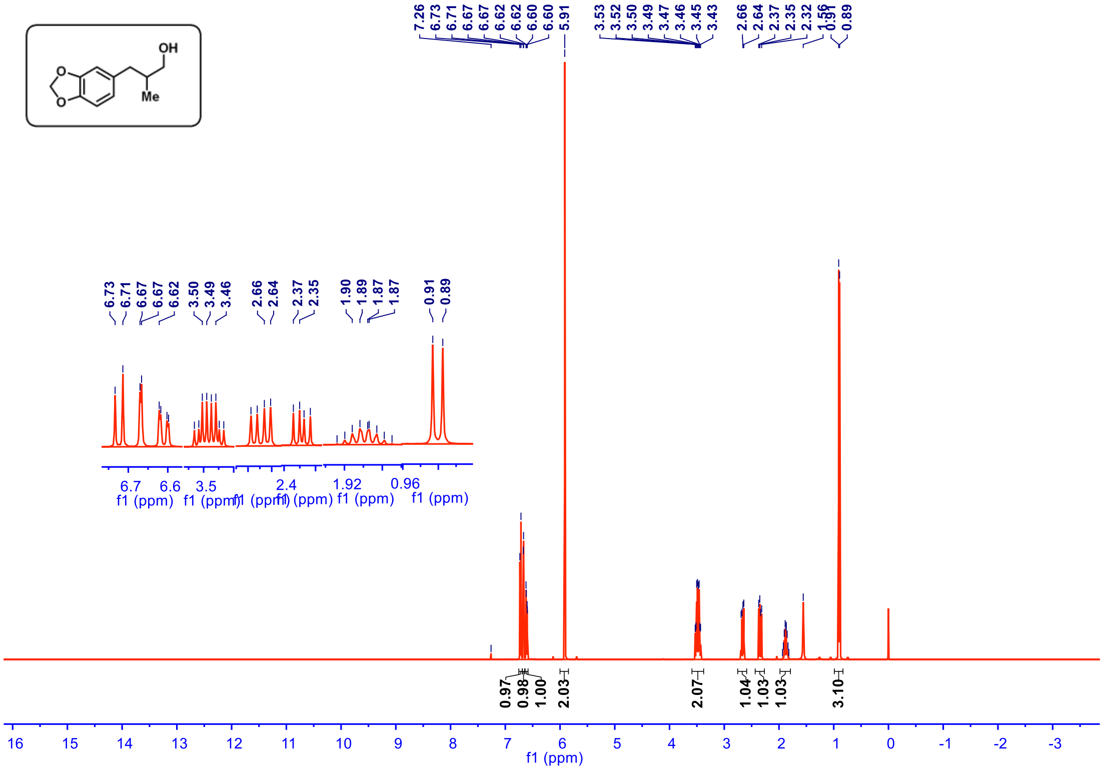

Supplementary Figure 441. ^{1}H NMR spectrum of **3-(benzo[*d*][1,3]dioxol-5-yl)-2-methylpropan-1-ol** (400 MHz, $CDCl_3$)

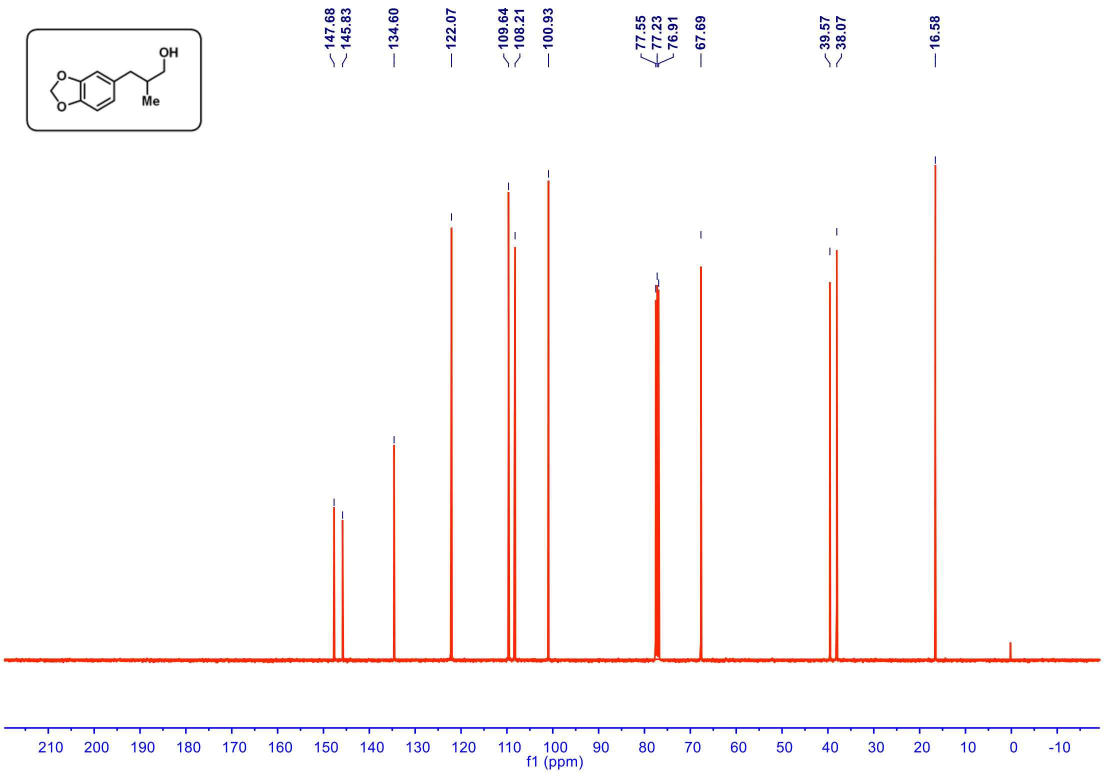

Supplementary Figure 442. ^{13}C NMR spectrum of **3-(benzo[*d*][1,3]dioxol-5-yl)-2-methylpropan-1-ol** (101 MHz, $CDCl_3$)

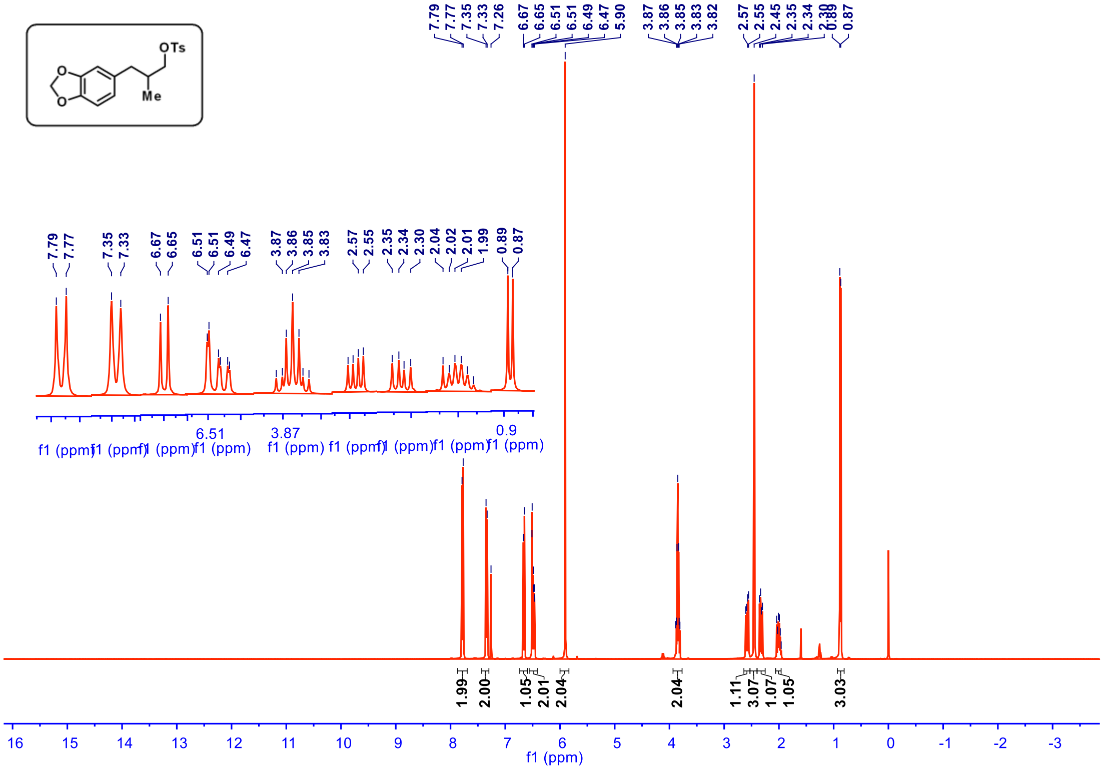

Supplementary Figure 443. ^{1}H NMR spectrum of **3-(benzo[*d*][1,3]dioxol-5-yl)-2-methylpropyl 4-methylbenzenesulfonate** (400 MHz, $CDCl_3$)

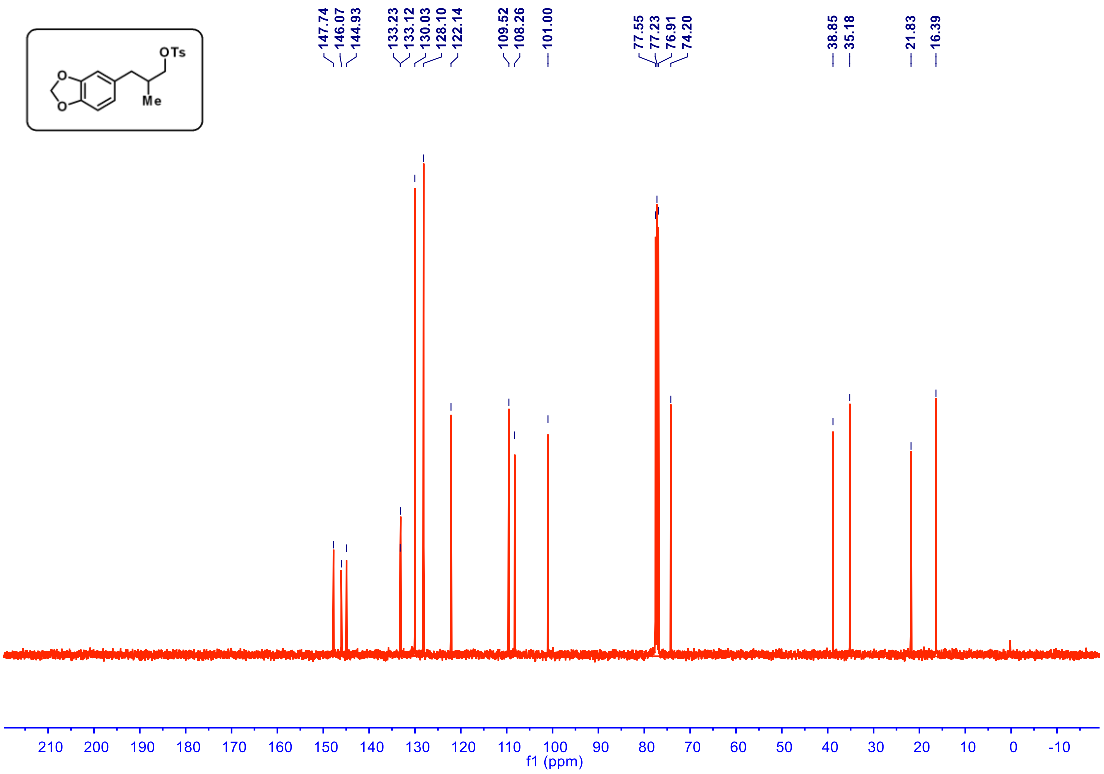

Supplementary Figure 444. ^{13}C NMR spectrum of **3-(benzo[*d*][1,3]dioxol-5-yl)-2-methylpropyl 4-methylbenzenesulfonate** (101 MHz, $CDCl_3$)

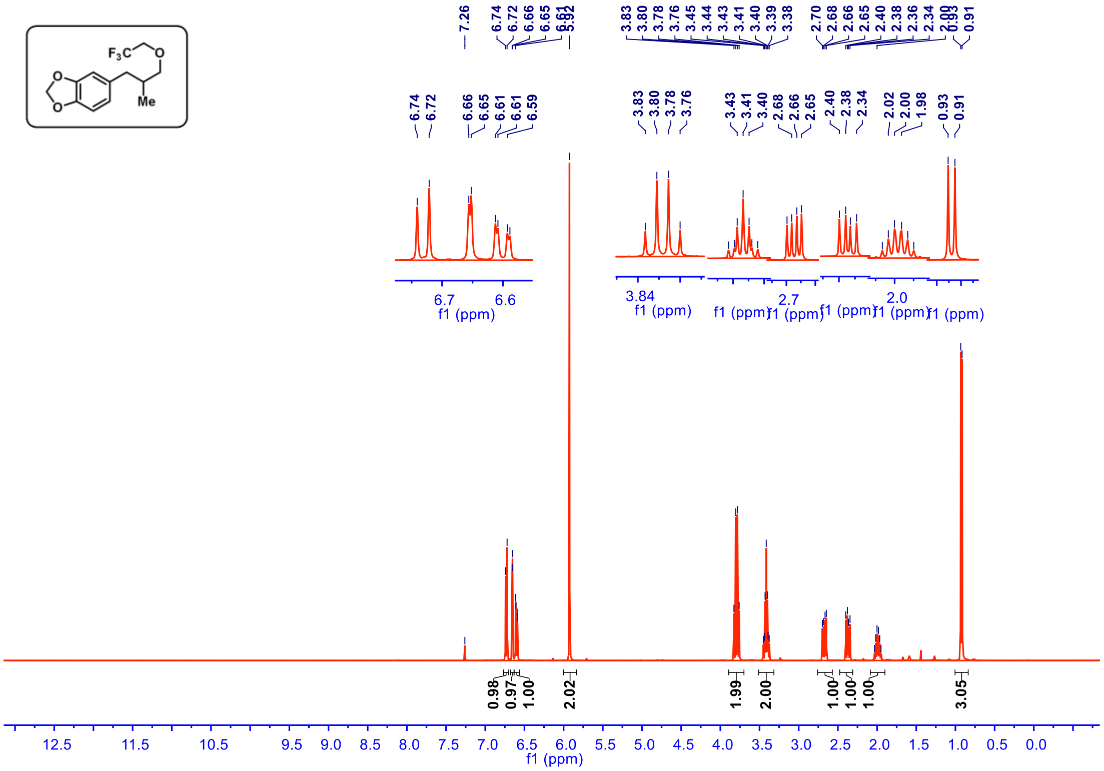

Supplementary Figure 445. ^{1}H NMR spectrum of compound **44** (400 MHz, $CDCl_3$)

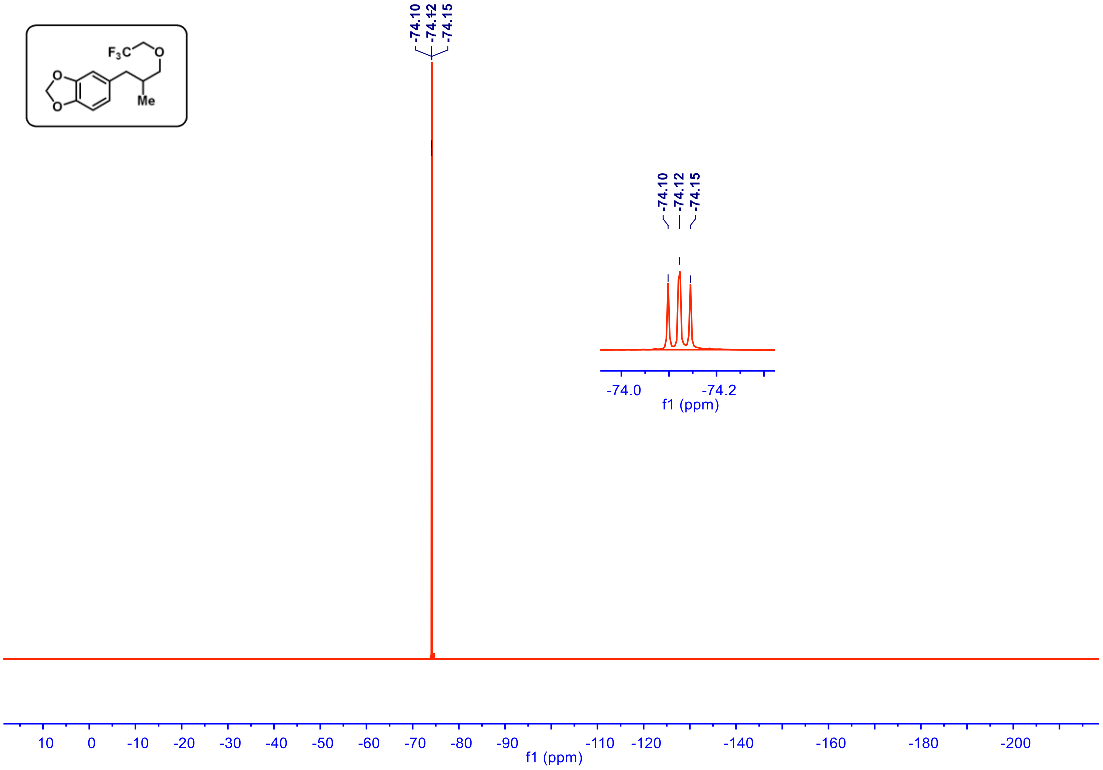

Supplementary Figure 446. ^{19}F NMR spectrum of compound **44** (376 MHz, $CDCl_3$)

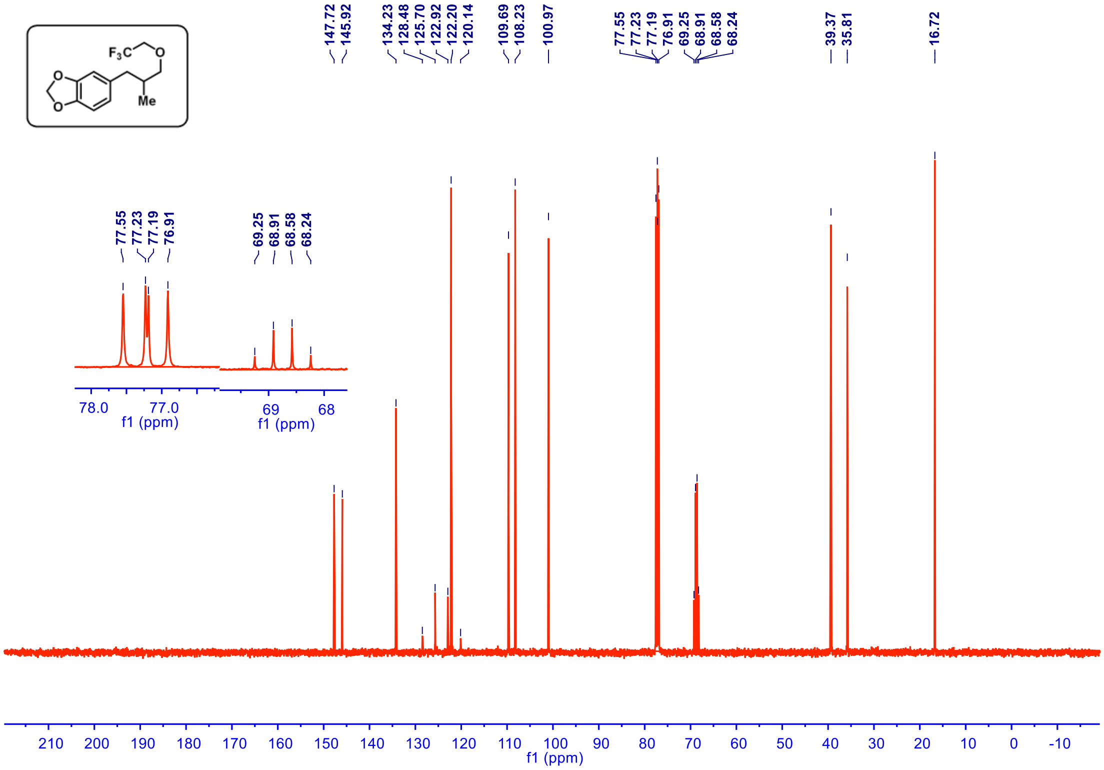

Supplementary Figure 447. ^{13}C NMR spectrum of compound **44** (101 MHz, $CDCl_3$)

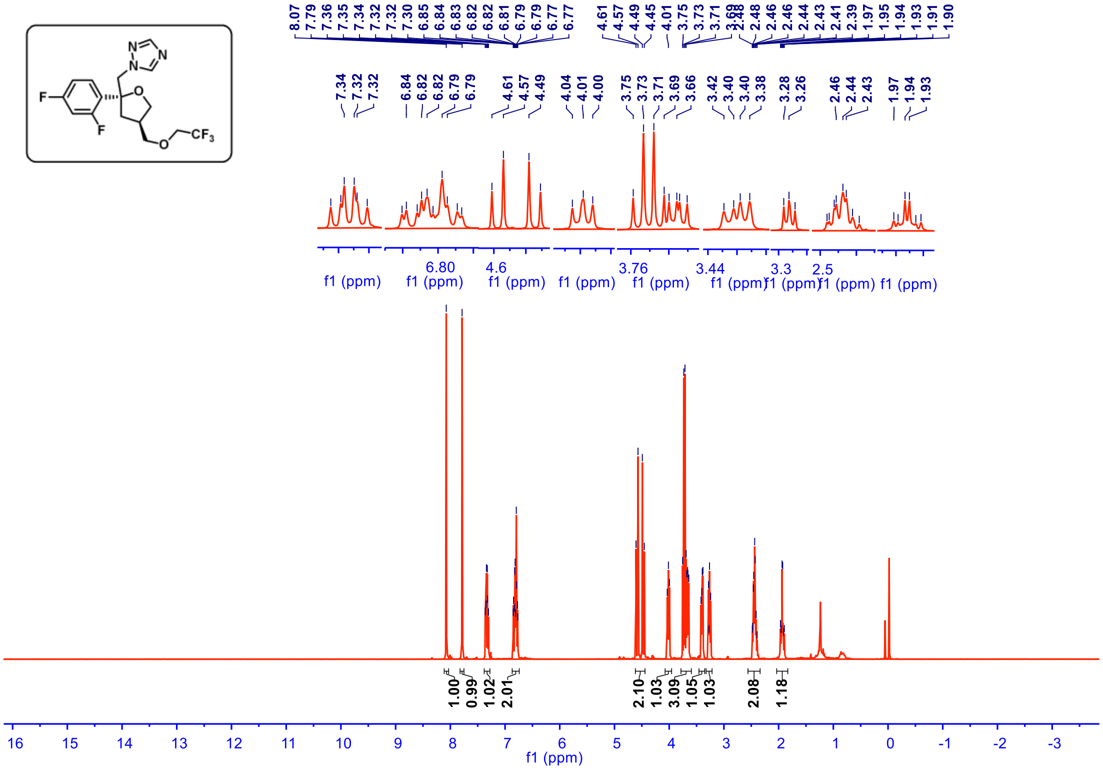

Supplementary Figure 448. ^{1}H NMR spectrum of compound **45** (400 MHz, $CDCl_3$)

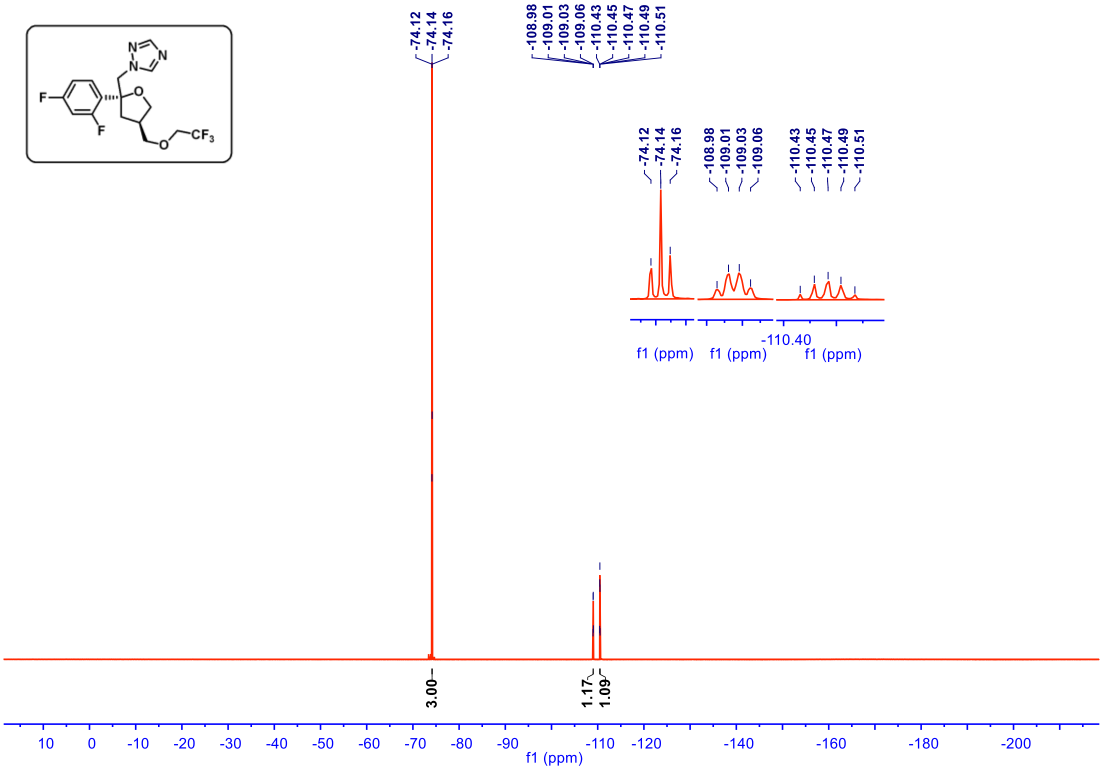

Supplementary Figure 449. ^{19}F NMR spectrum of compound **45** (376 MHz, $CDCl_3$)

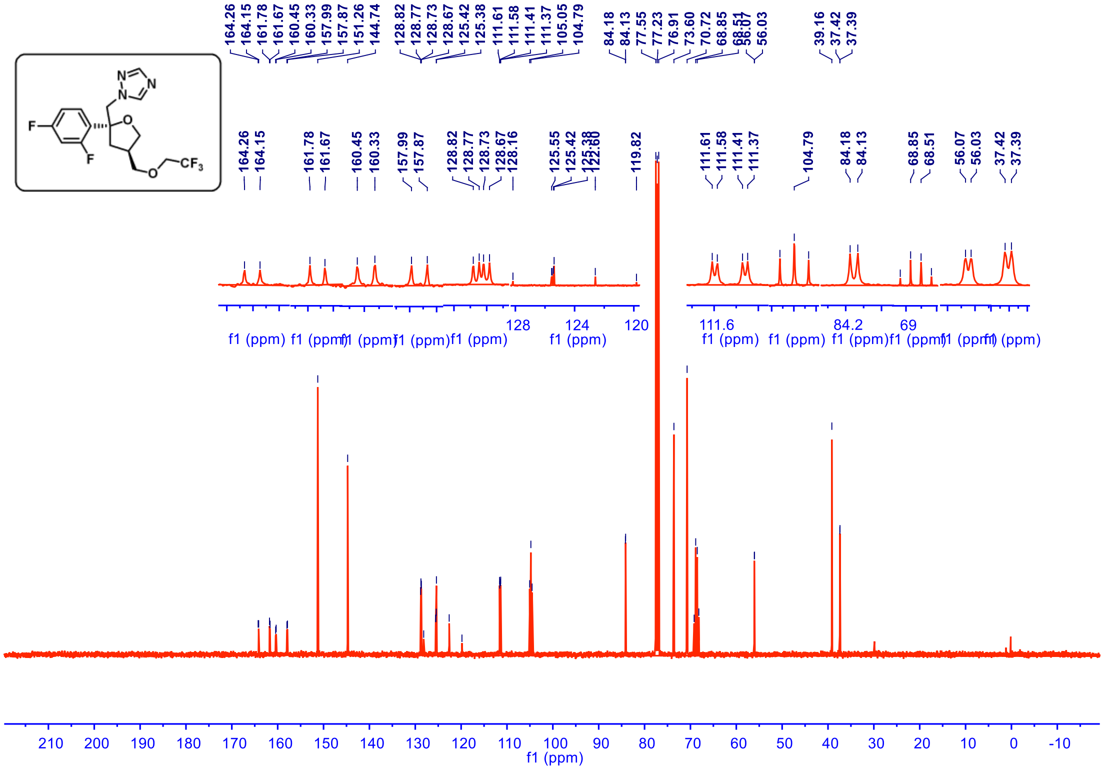

Supplementary Figure 450. ^{13}C NMR spectrum of compound **45** (101 MHz, $CDCl_3$)

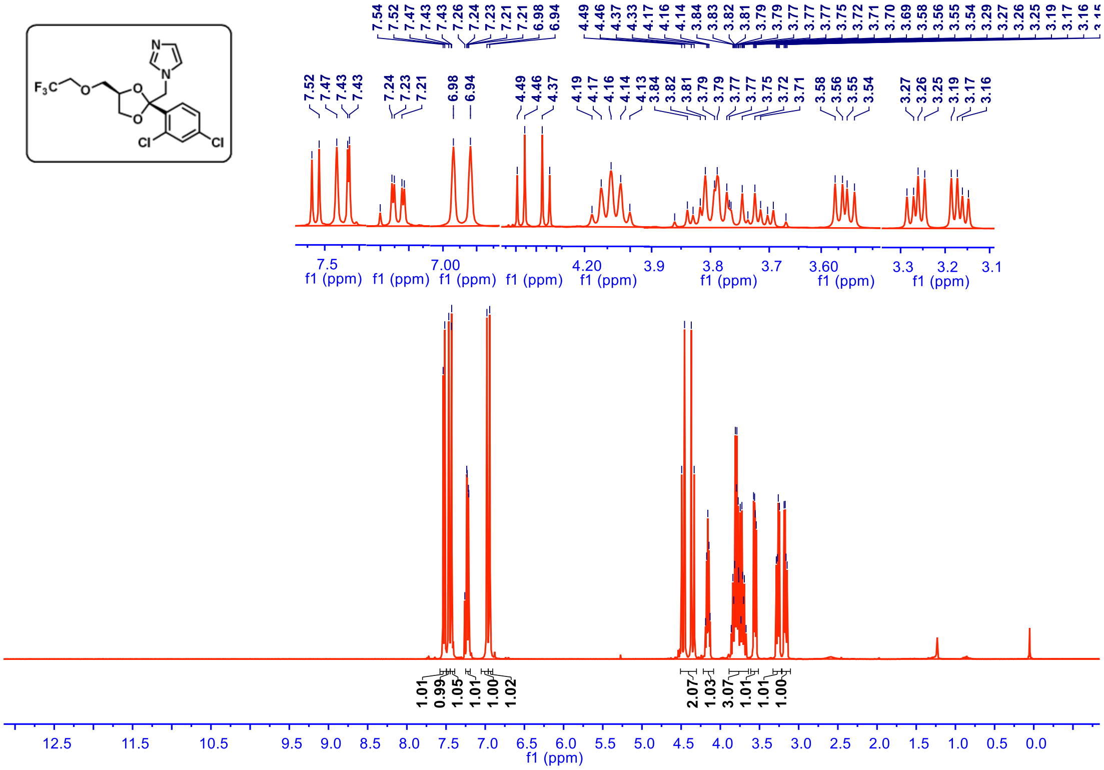

Supplementary Figure 451. ^{1}H NMR spectrum of compound **46** (400 MHz, $CDCl_3$)

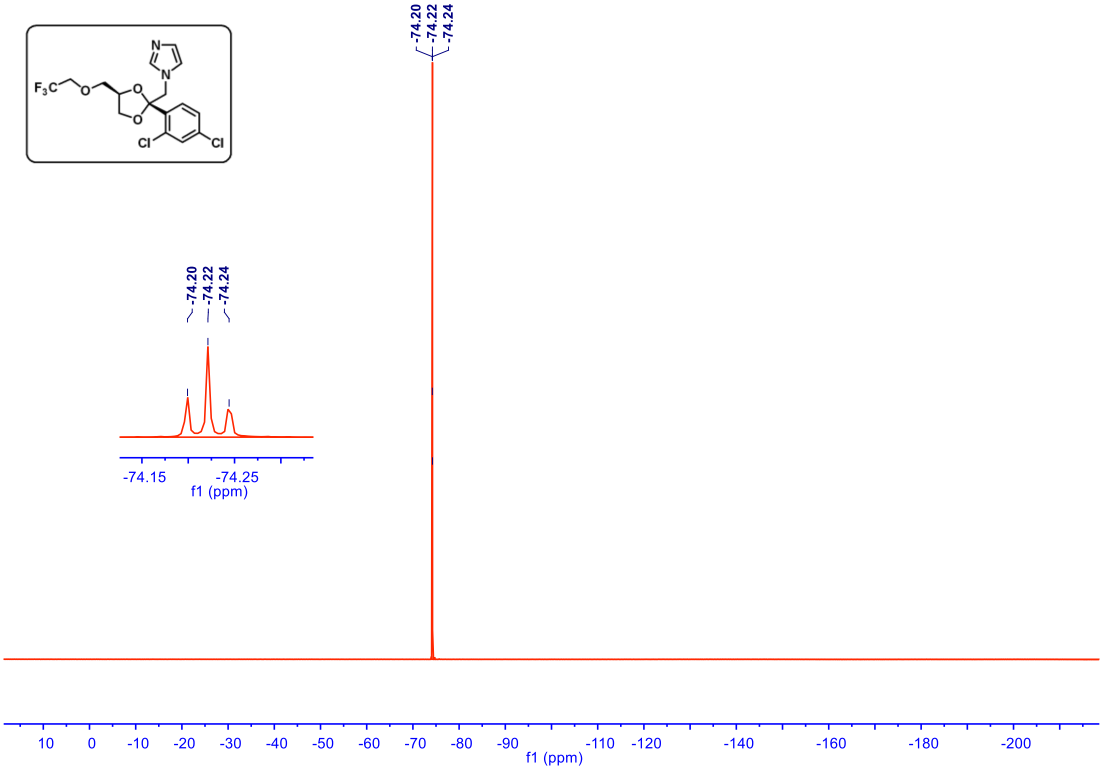

Supplementary Figure 452. ^{19}F NMR spectrum of compound **46** (376 MHz, $CDCl_3$)

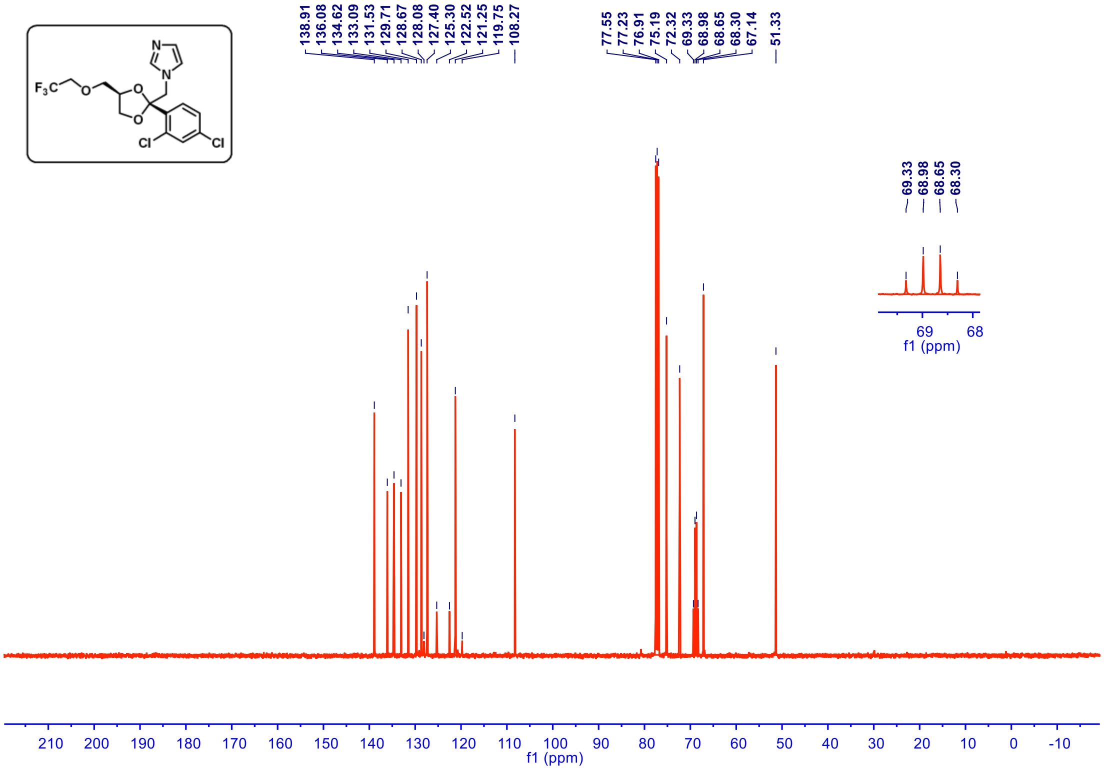

Supplementary Figure 453. ^{13}C NMR spectrum of compound **46** (101 MHz, $CDCl_3$)

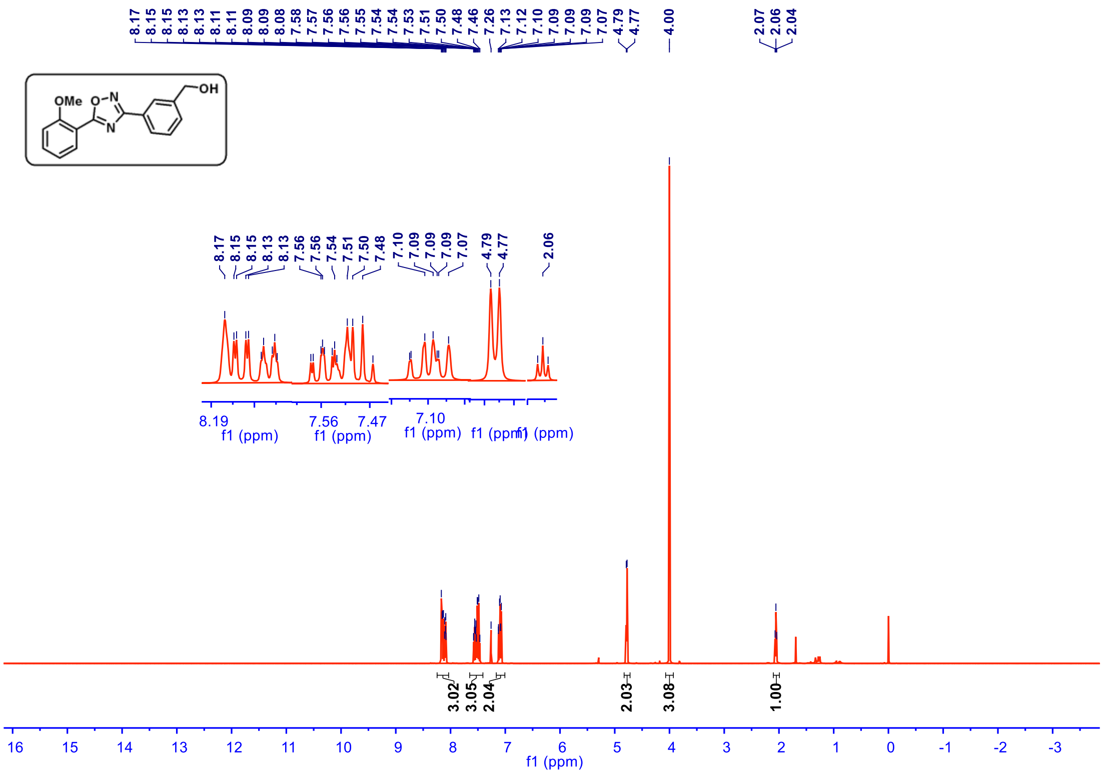

Supplementary Figure 454. ^{1}H NMR spectrum of **(3-(5-(2-methoxyphenyl)-1,2,4-oxadiazol-3-yl)phenyl)methanol** (400 MHz, $CDCl_3$)

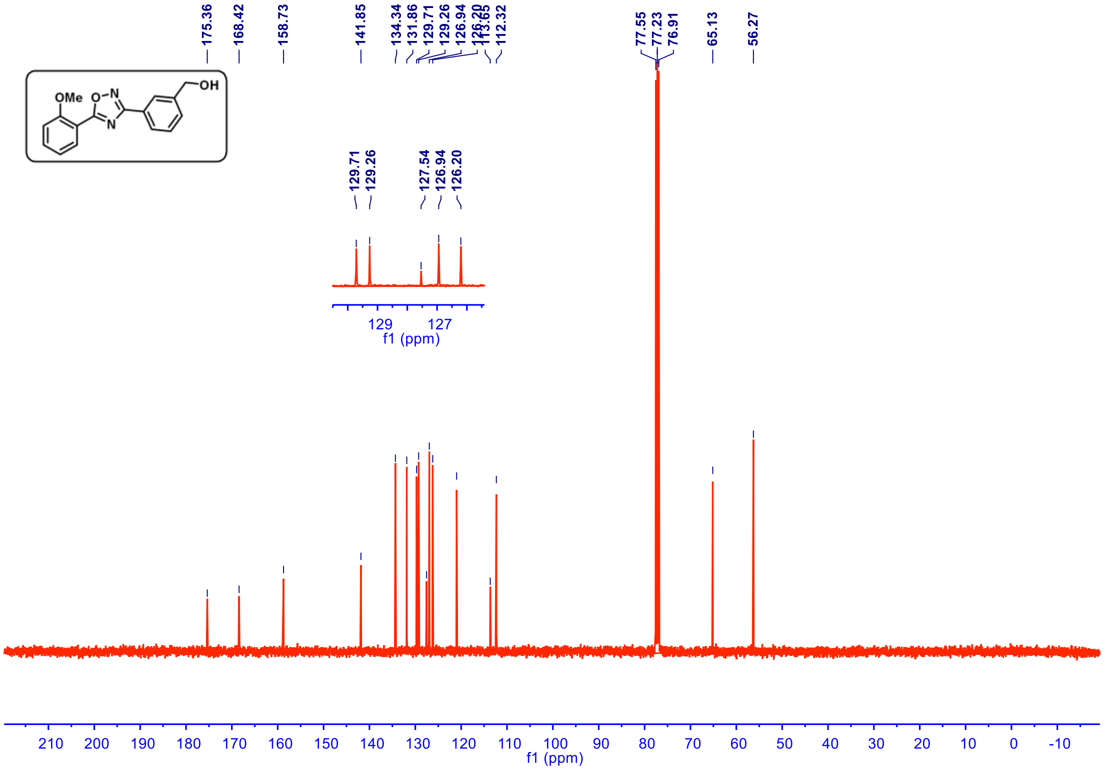

Supplementary Figure 455. ^{13}C NMR spectrum of **(3-(5-(2-methoxyphenyl)-1,2,4-oxadiazol-3-yl)phenyl)methanol** (101 MHz, $CDCl_3$)

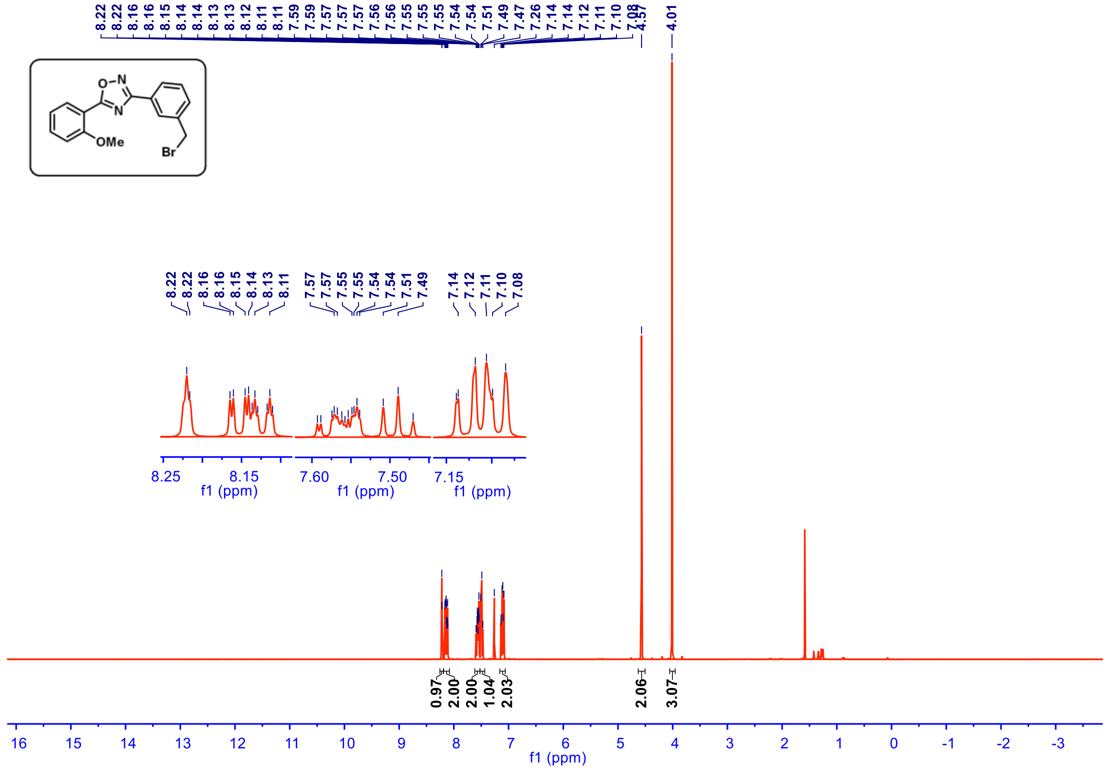

Supplementary Figure 456. ^{1}H NMR spectrum of **3-(3-(bromomethyl)phenyl)-5-(2-methoxyphenyl)-1,2,4-oxadiazole** (400 MHz, $CDCl_3$)

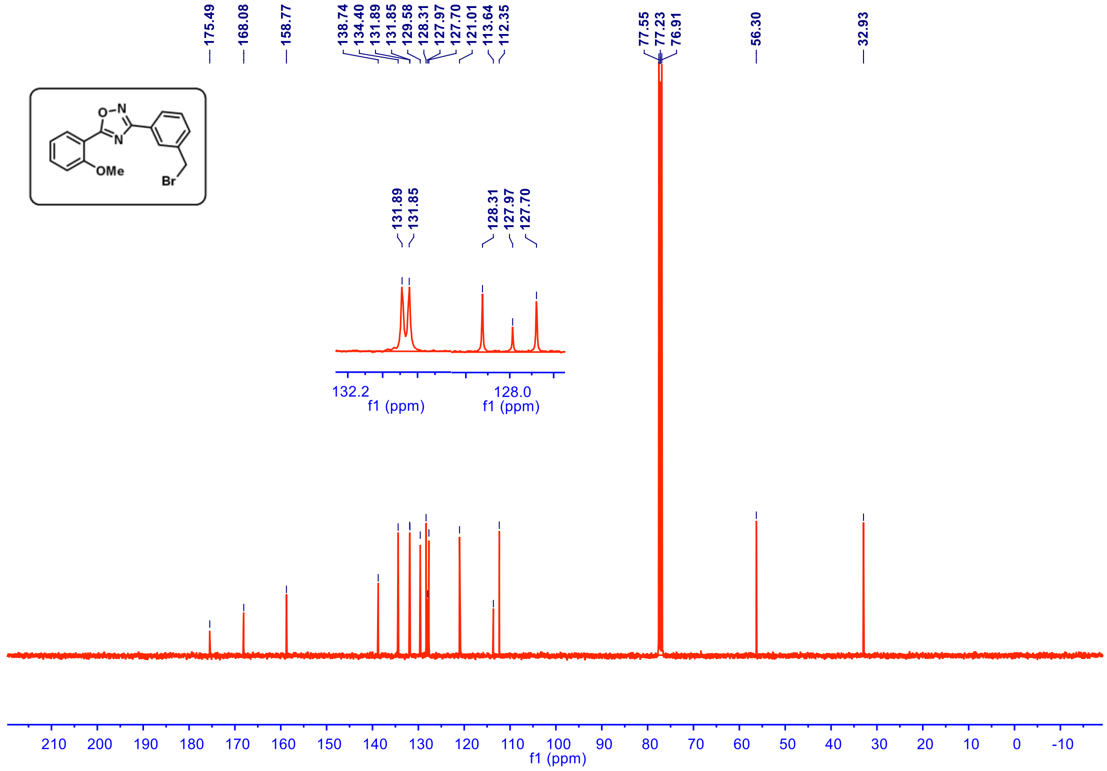

Supplementary Figure 457. ^{13}C NMR spectrum of **3-(3-(bromomethyl)phenyl)-5-(2-methoxyphenyl)-1,2,4-oxadiazole** (101 MHz, $CDCl_3$)

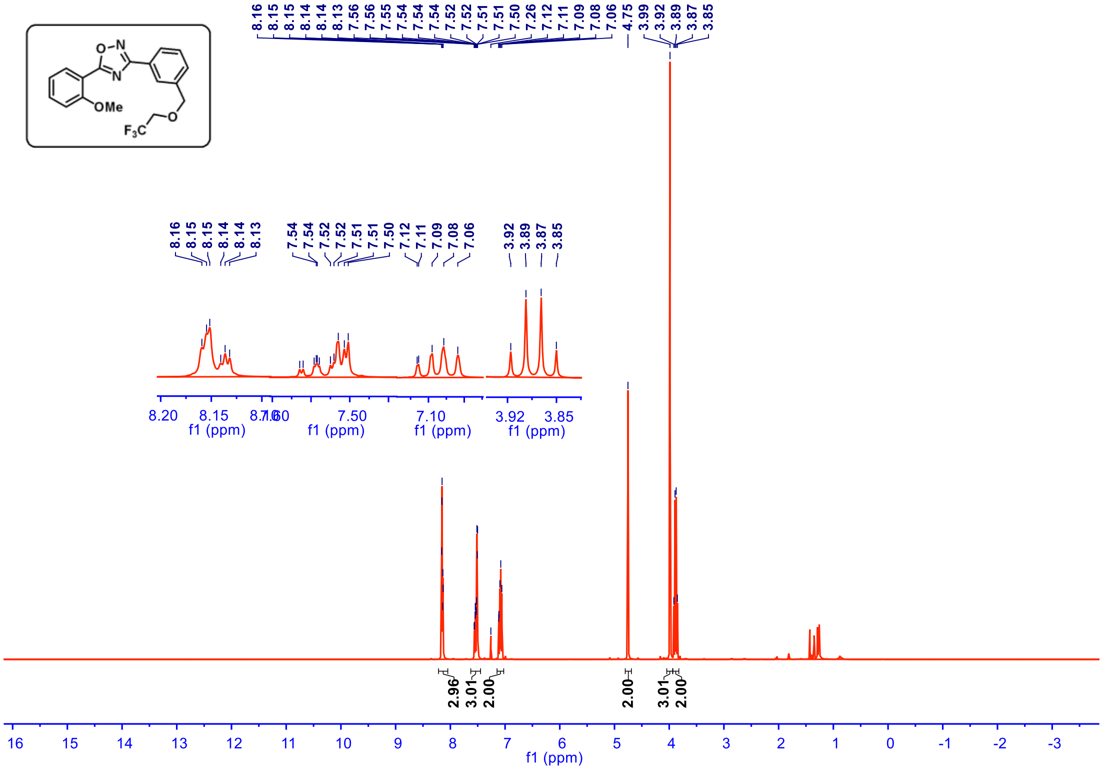

Supplementary Figure 458. ^{1}H NMR spectrum of compound **47** (400 MHz, $CDCl_3$)

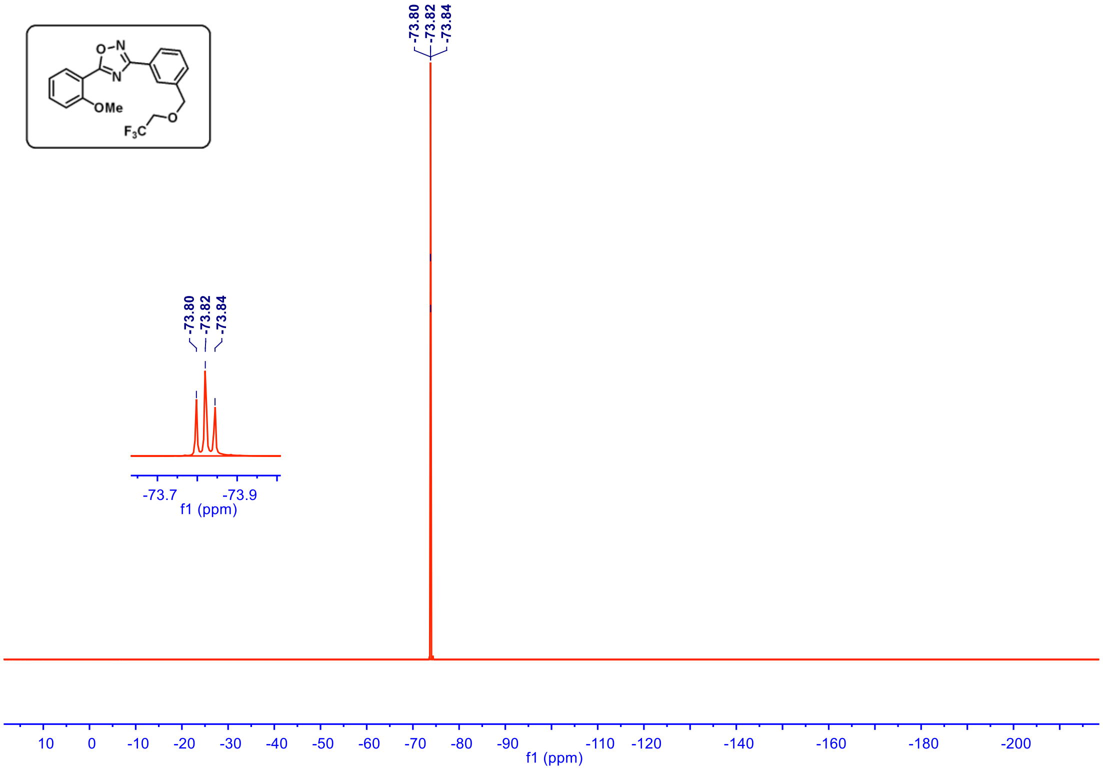

Supplementary Figure 459. ^{19}F NMR spectrum of compound **47** (376 MHz, $CDCl_3$)

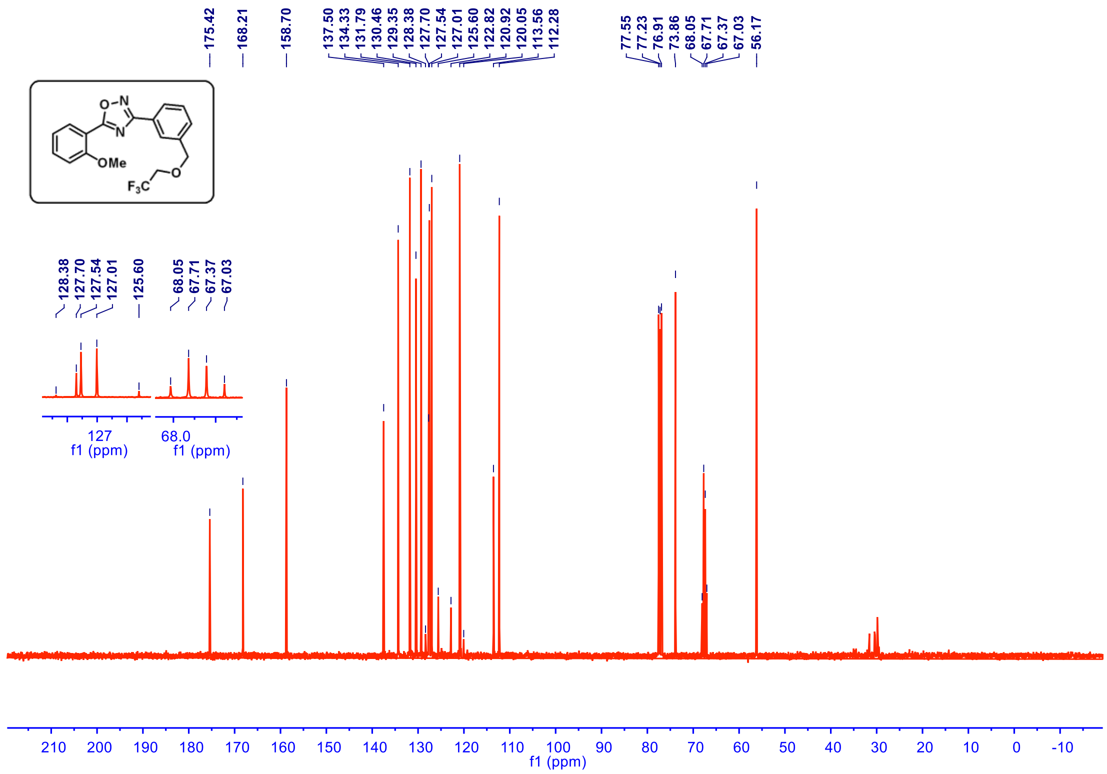

Supplementary Figure 460. ^{13}C NMR spectrum of compound **47** (101 MHz, $CDCl_3$)

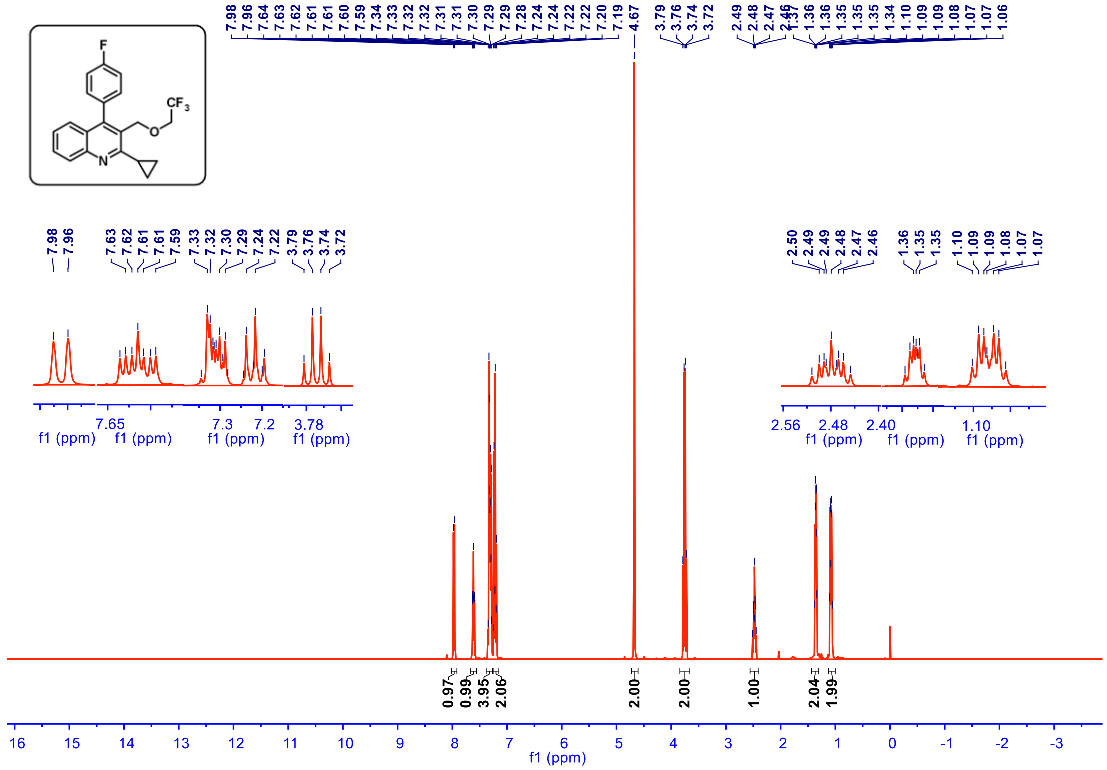

Supplementary Figure 461. ^{1}H NMR spectrum of compound **48** (400 MHz, $CDCl_3$)

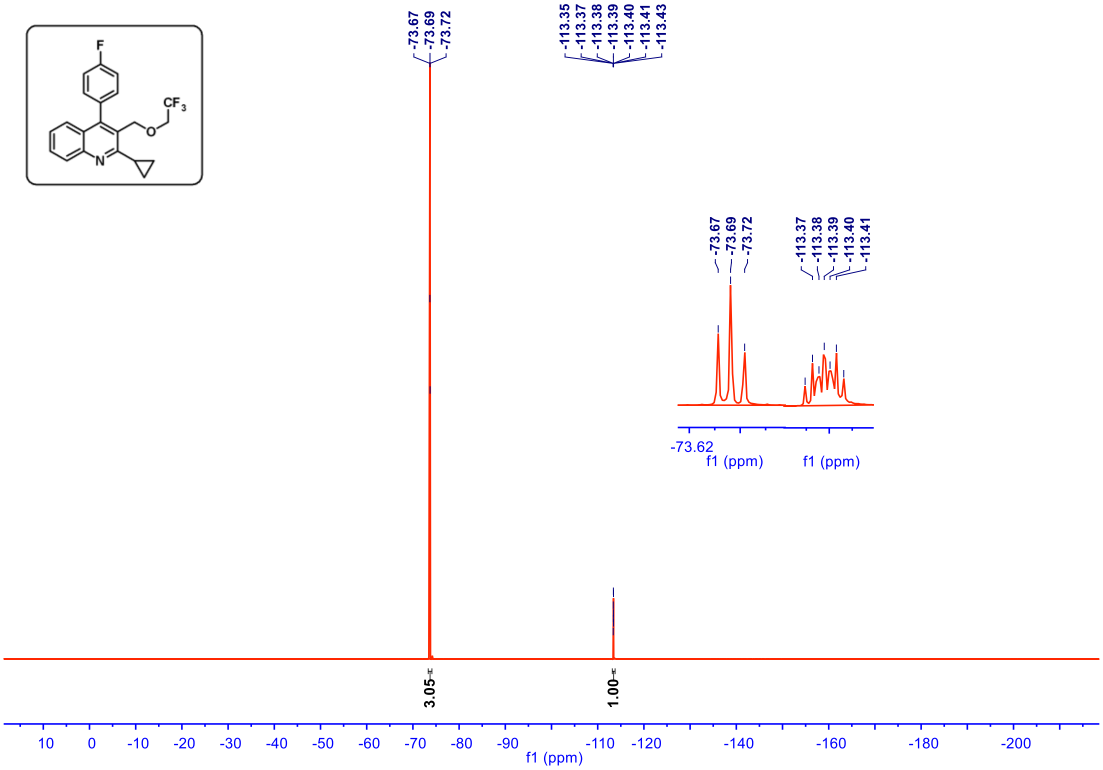

Supplementary Figure 462. ^{19}F NMR spectrum of compound **48** (376 MHz, $CDCl_3$)

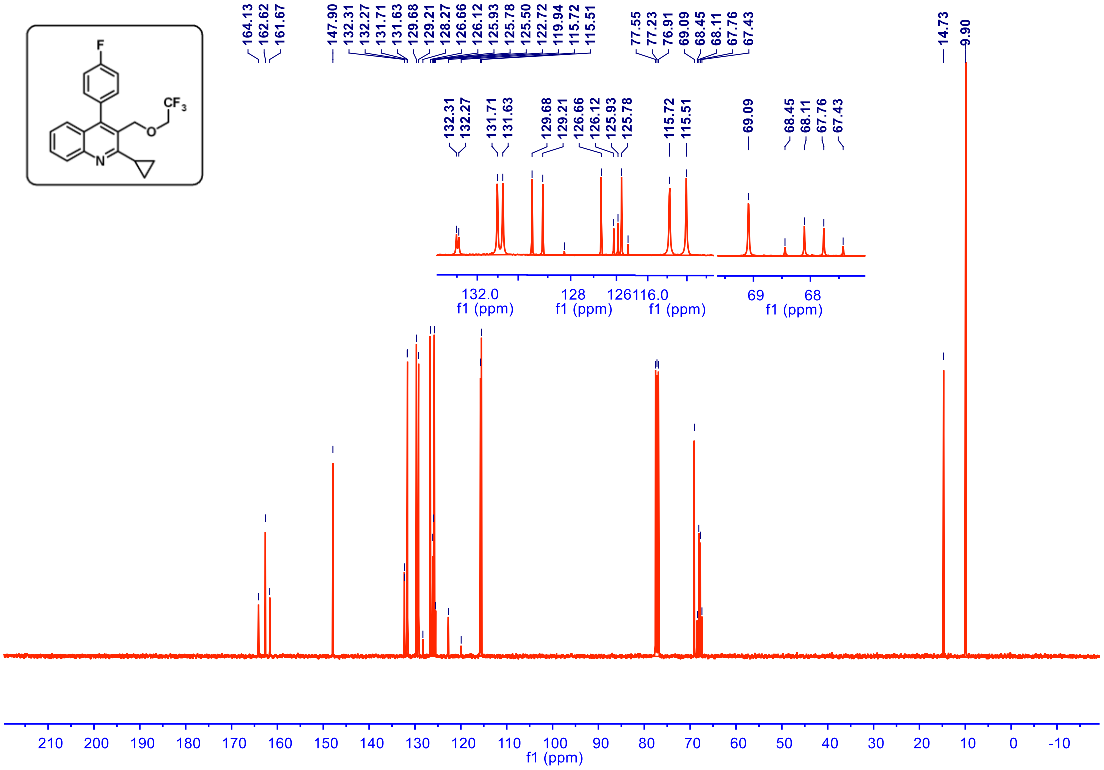

Supplementary Figure 463. ^{13}C NMR spectrum of compound **48** (101 MHz, $CDCl_3$)

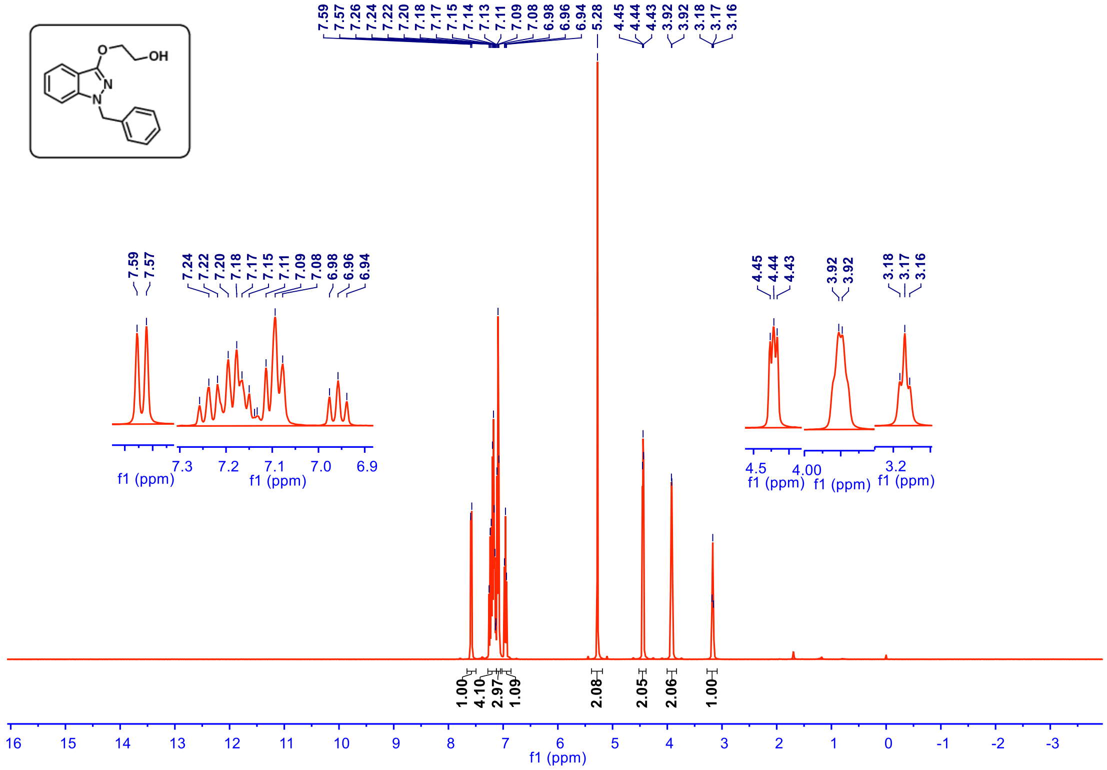

Supplementary Figure 464. ^{1}H NMR spectrum of **2-(1-benzyl-1*H*-indazol-3-yloxy)ethanol** (400 MHz, $CDCl_3$)

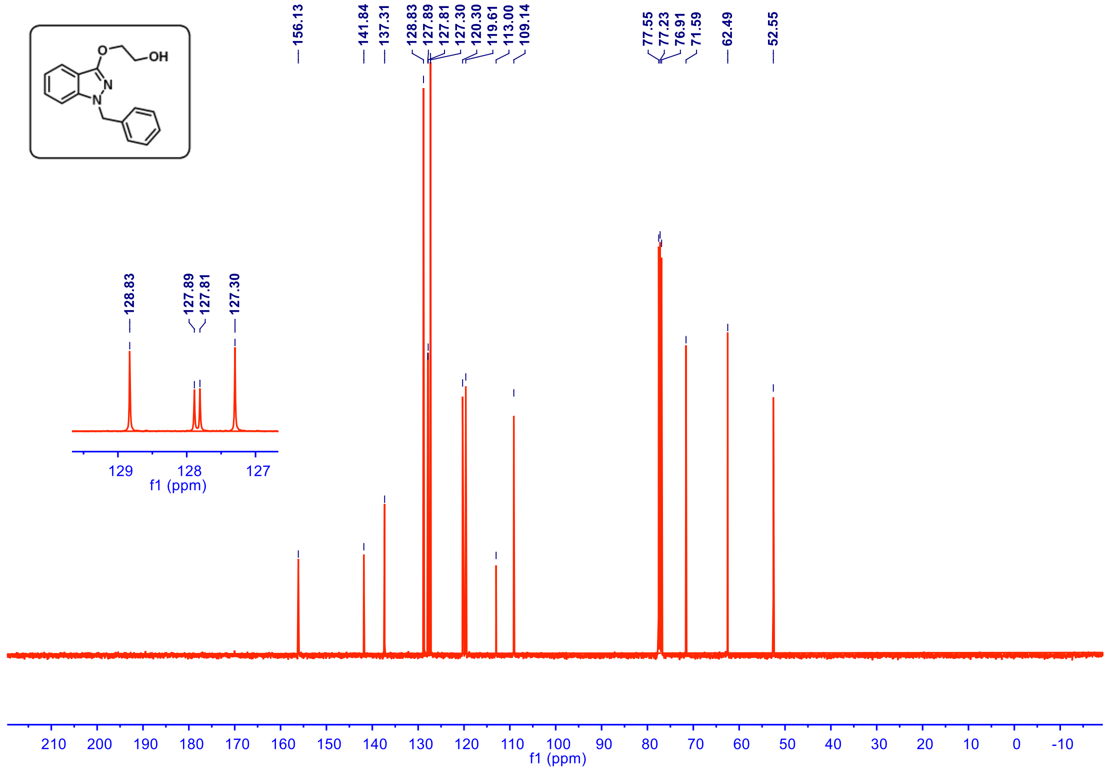

Supplementary Figure 465. ^{13}C NMR spectrum of **2-(1-benzyl-1*H*-indazol-3-yloxy)ethanol** (101 MHz, $CDCl_3$)

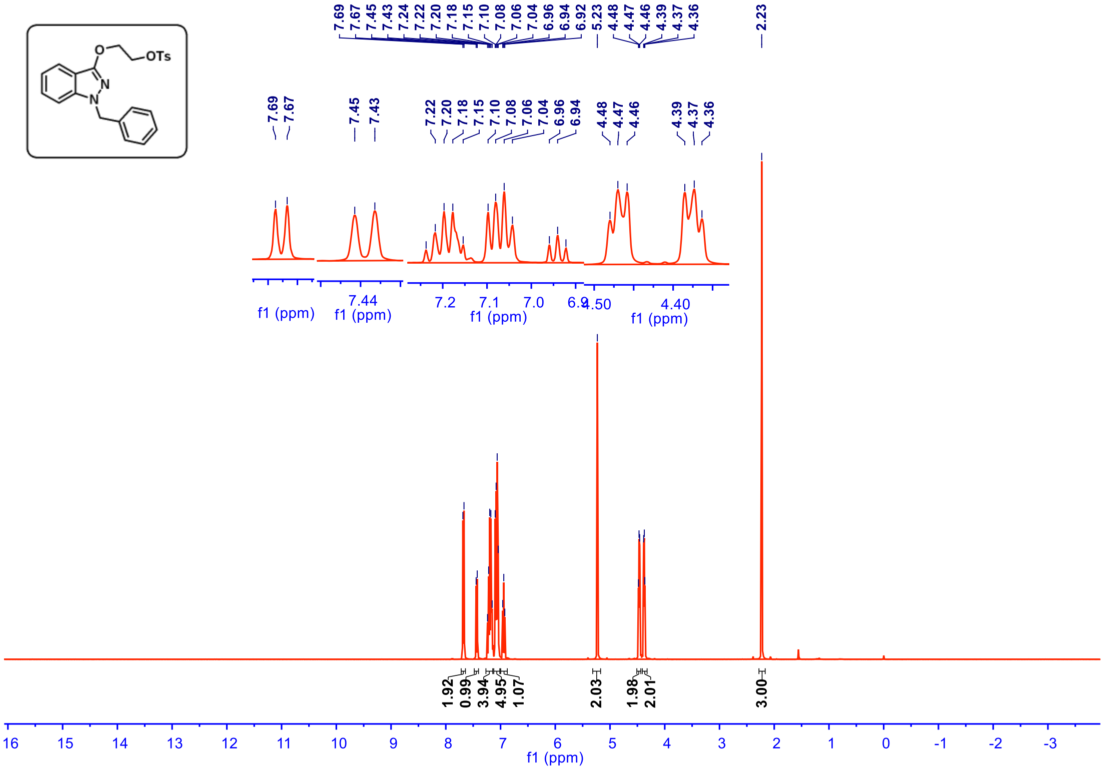

Supplementary Figure 466. ^{1}H NMR spectrum of **2-(1-benzyl-1*H*-indazol-3-yloxy)ethyl 4-methylbenzenesulfonate** (400 MHz, $CDCl_3$)

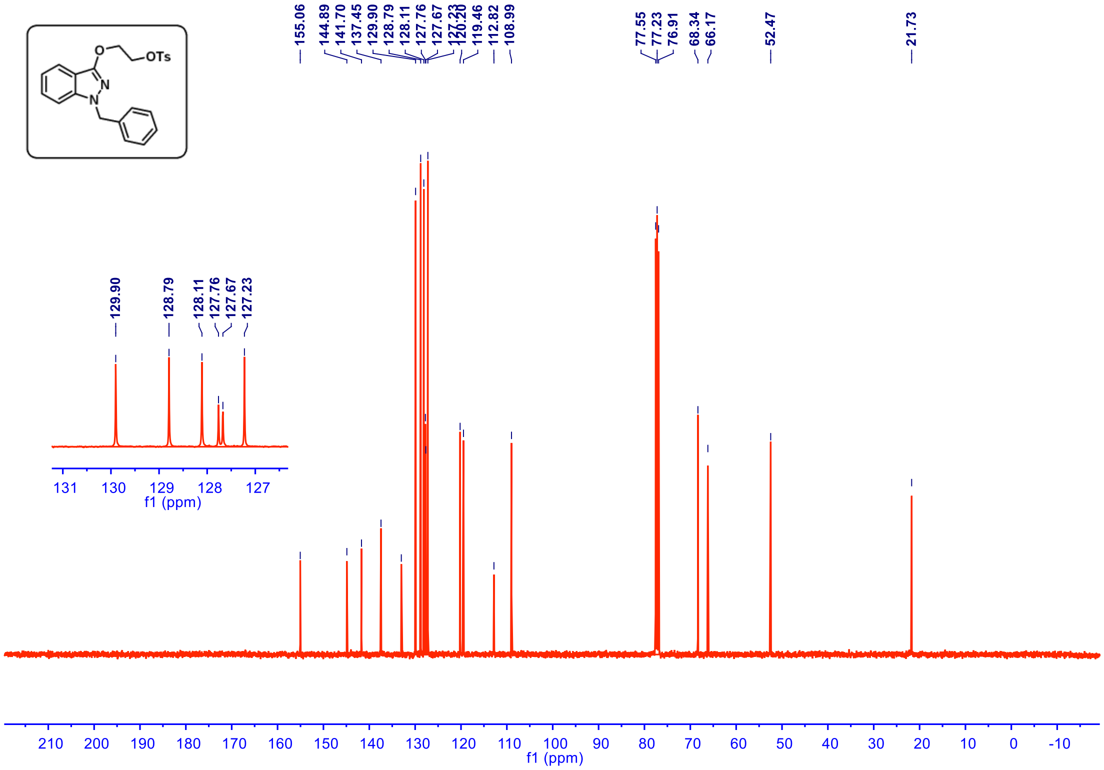

Supplementary Figure 467. ^{13}C NMR spectrum of **2-(1-benzyl-1*H*-indazol-3-yloxy)ethyl 4-methylbenzenesulfonate** (101 MHz, $CDCl_3$)

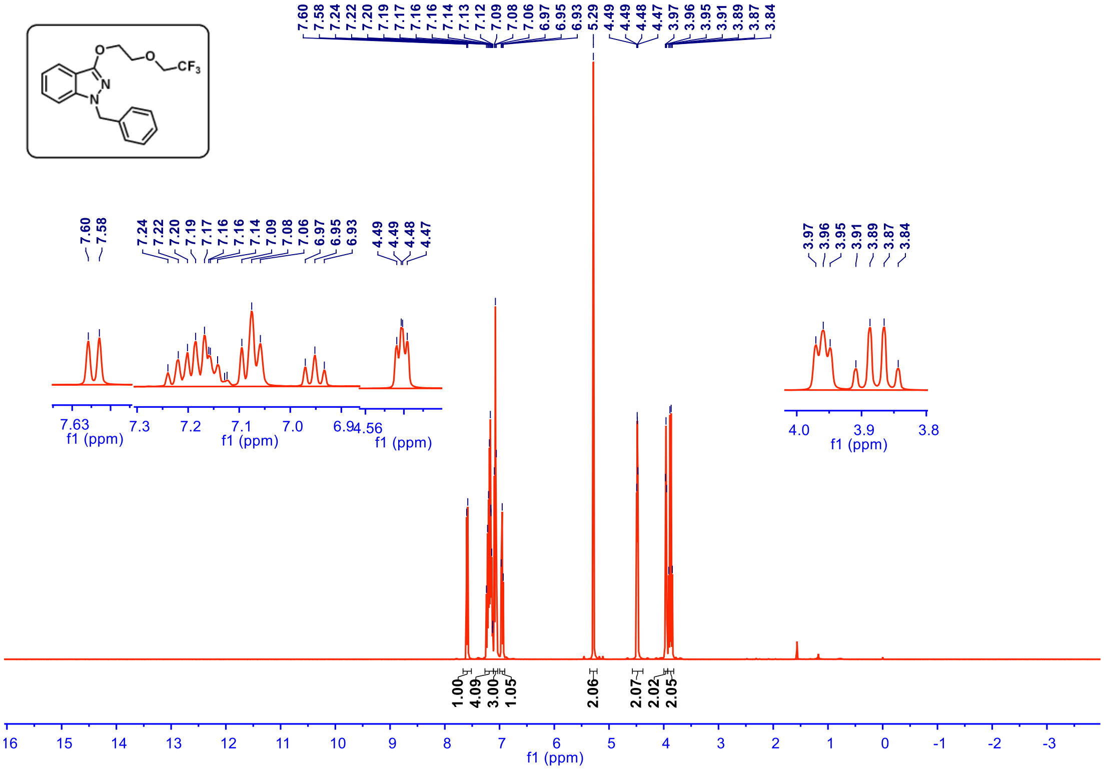

Supplementary Figure 468. ^{1}H NMR spectrum of compound **49** (400 MHz, $CDCl_3$)

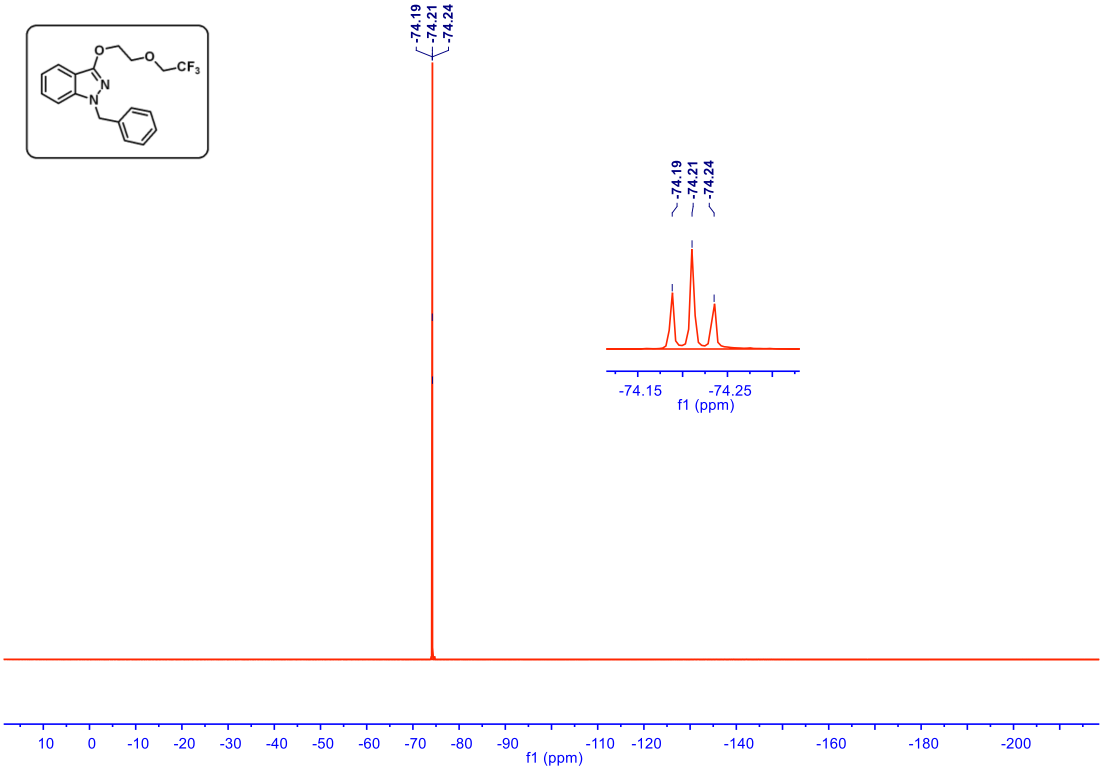

Supplementary Figure 469. ^{19}F NMR spectrum of compound **49** (376 MHz, $CDCl_3$)

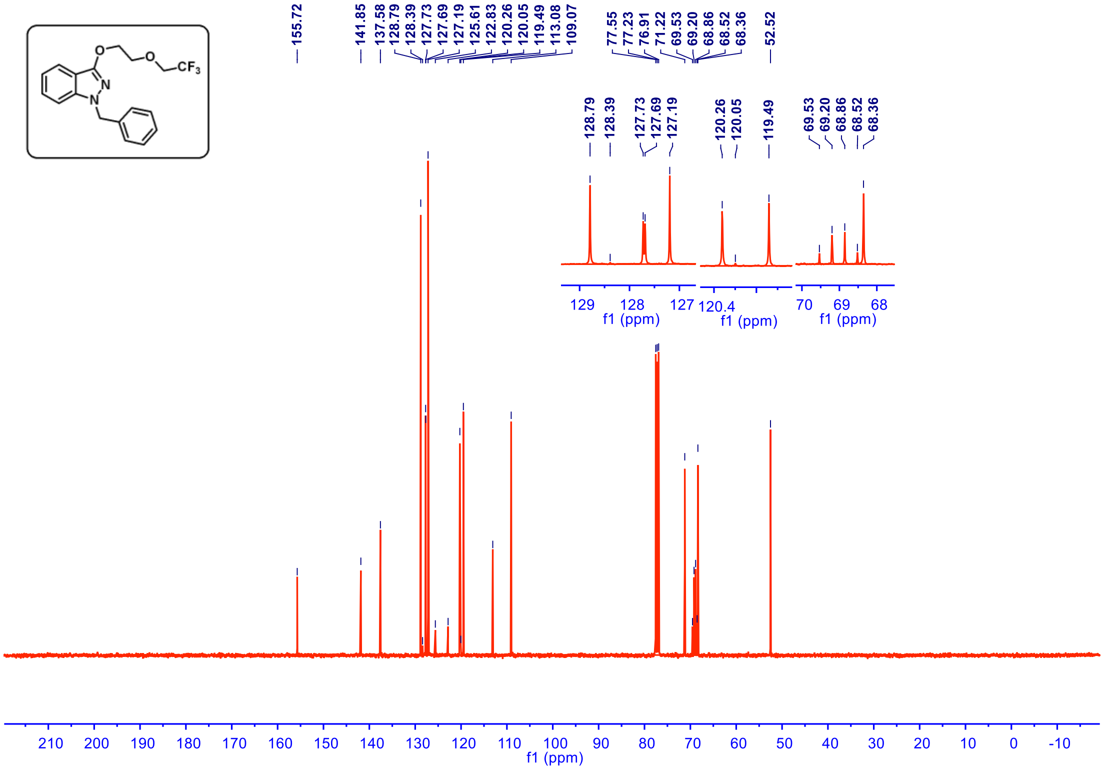

Supplementary Figure 470. ^{13}C NMR spectrum of compound **49** (101 MHz, $CDCl_3$)

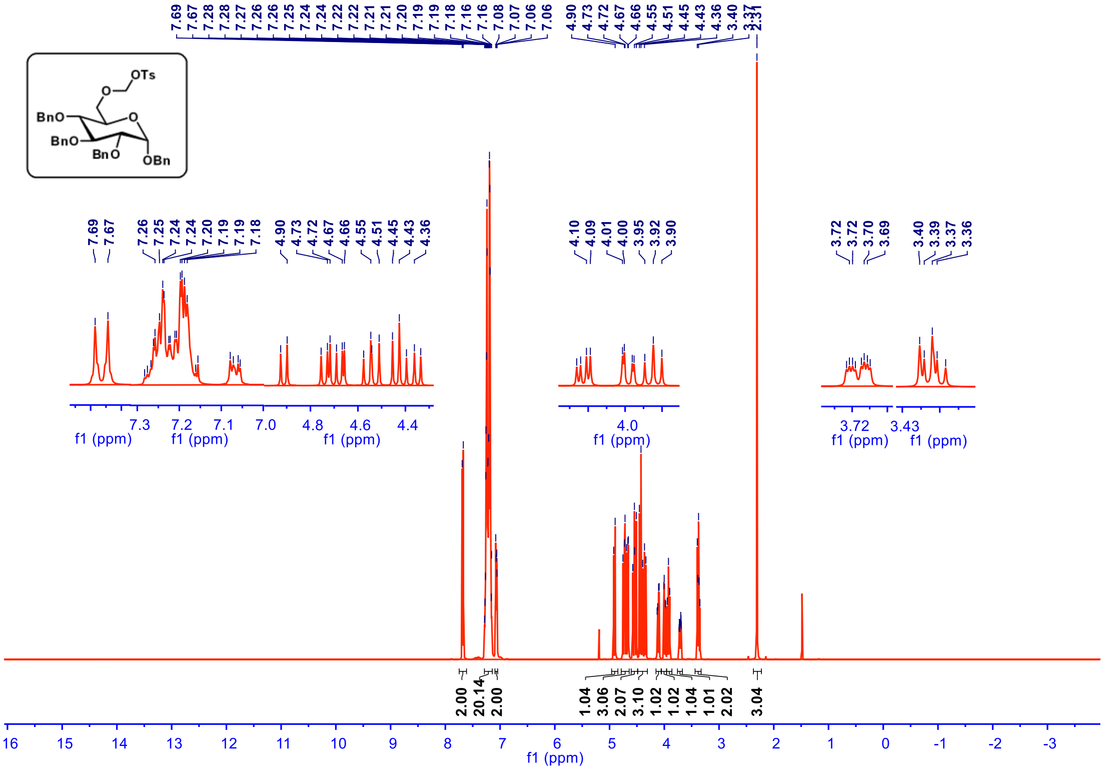

Supplementary Figure 471. ^{1}H NMR spectrum of **((2*R*,3*R*,4*S*,5*R*,6*S*)-3,4,5,6-tetrakis(benzyloxy)tetrahydro-2*H*-pyran-2-yl)methyl 4-methylbenzenesulfonate** (400 MHz, $CDCl_3$)

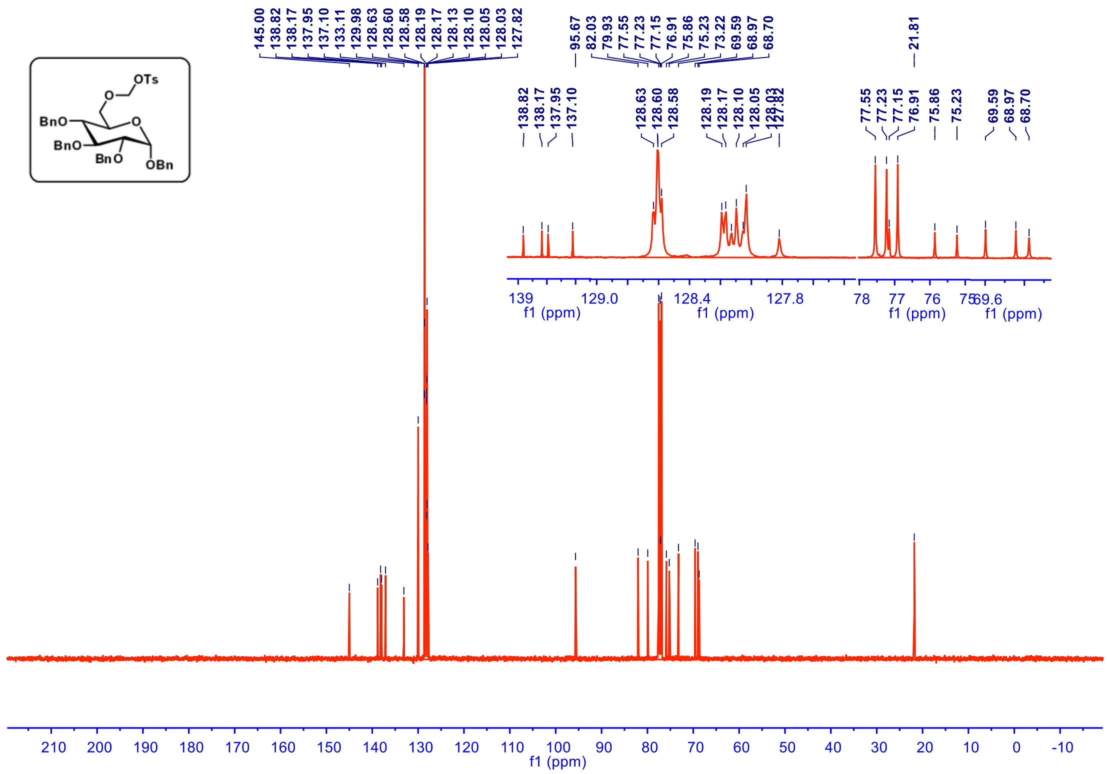

Supplementary Figure 472. ^{13}C NMR spectrum of **((2*R*,3*R*,4*S*,5*R*,6*S*)-3,4,5,6-tetrakis(benzyloxy)tetrahydro-2*H*-pyran-2-yl)methyl 4-methylbenzenesulfonate** (101 MHz, $CDCl_3$)

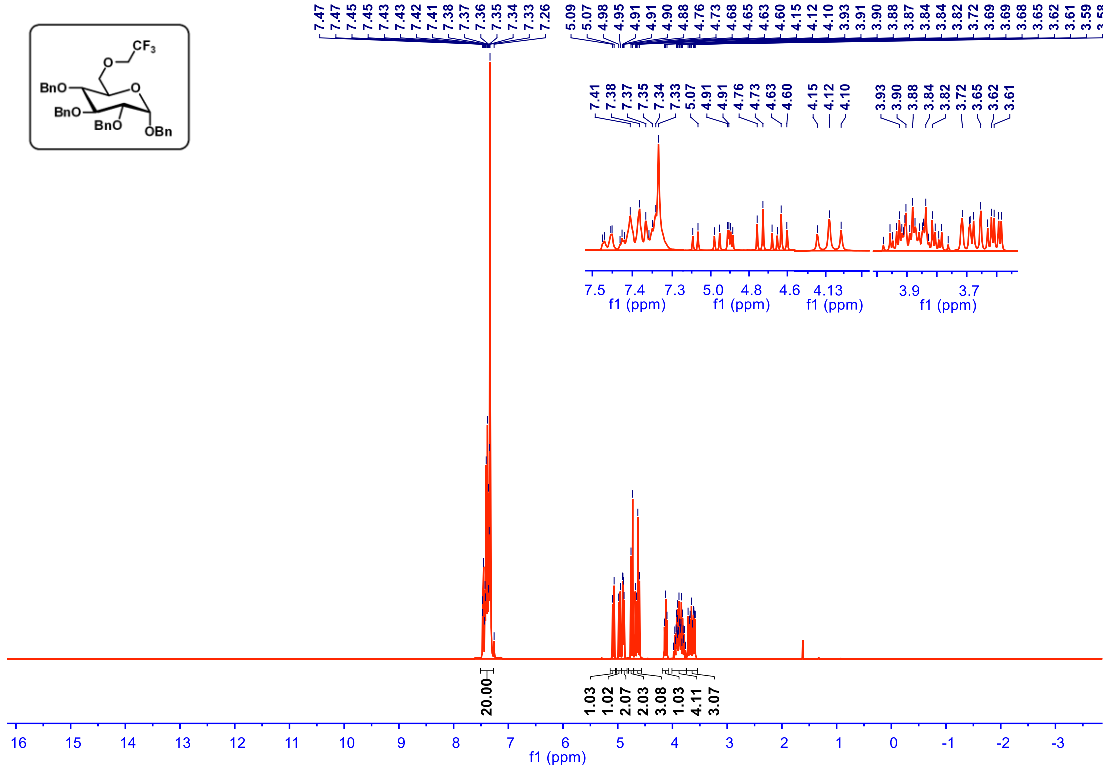

Supplementary Figure 473. ^{1}H NMR spectrum of compound **50** (400 MHz, $CDCl_3$)

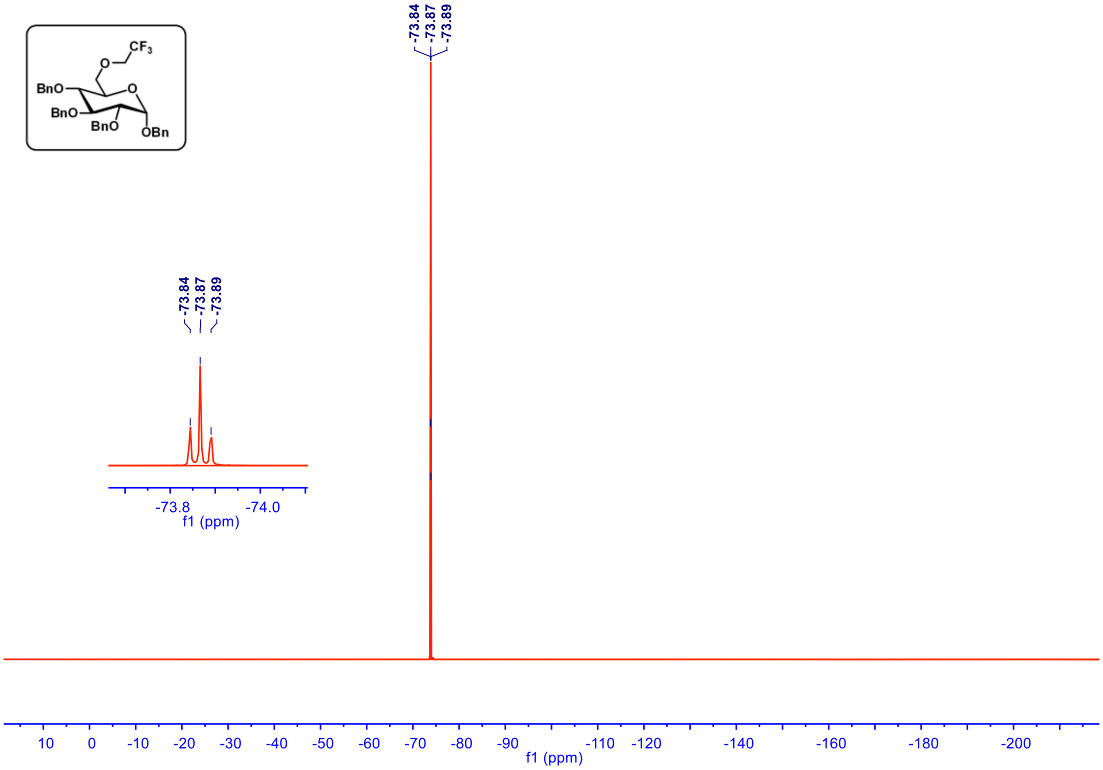

Supplementary Figure 474. ^{19}F NMR spectrum of compound **50** (376 MHz, $CDCl_3$)

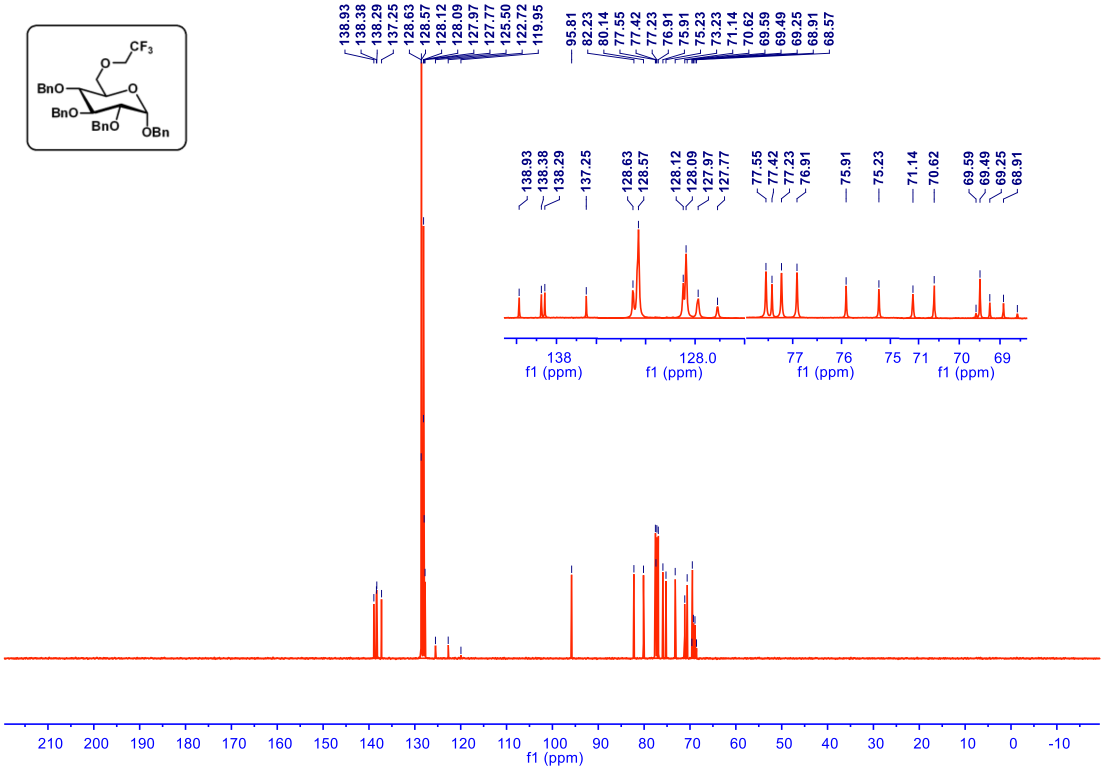

Supplementary Figure 475. ^{13}C NMR spectrum of compound **50** (101 MHz, $CDCl_3$)

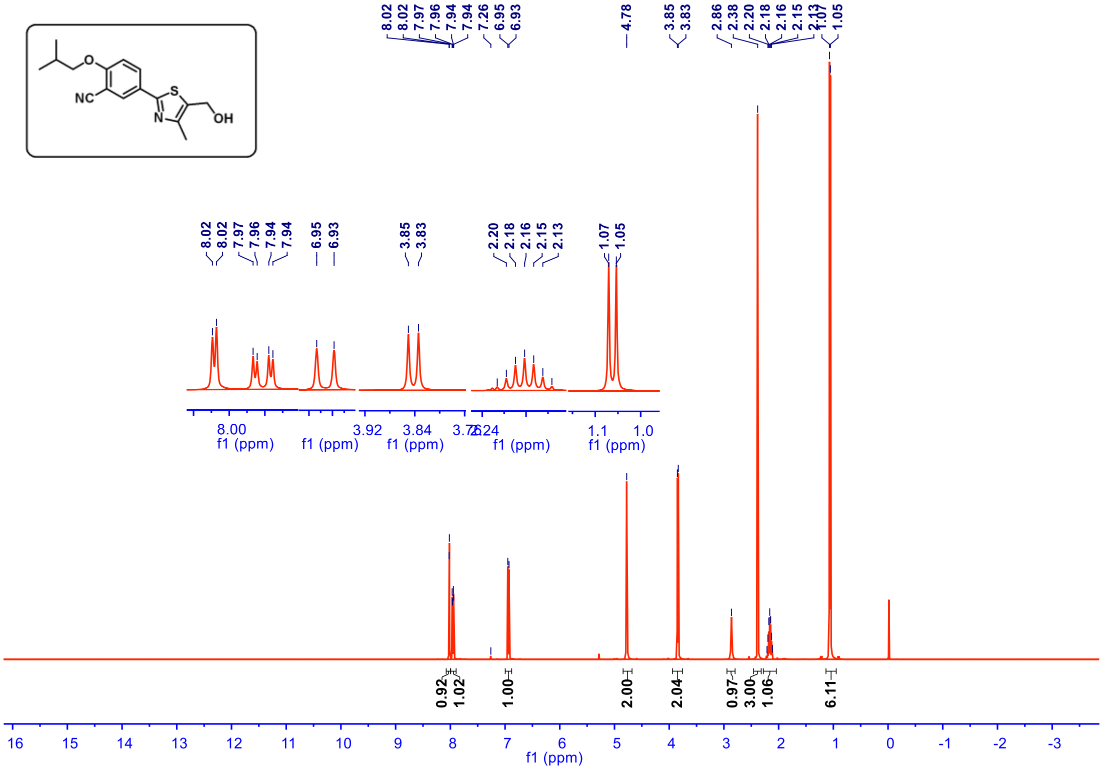

Supplementary Figure 476. ^{1}H NMR spectrum of **5-(5-(hydroxymethyl)-4-methylthiazol-2-yl)-2-isobutoxybenzonitrile** (400 MHz, $CDCl_3$)

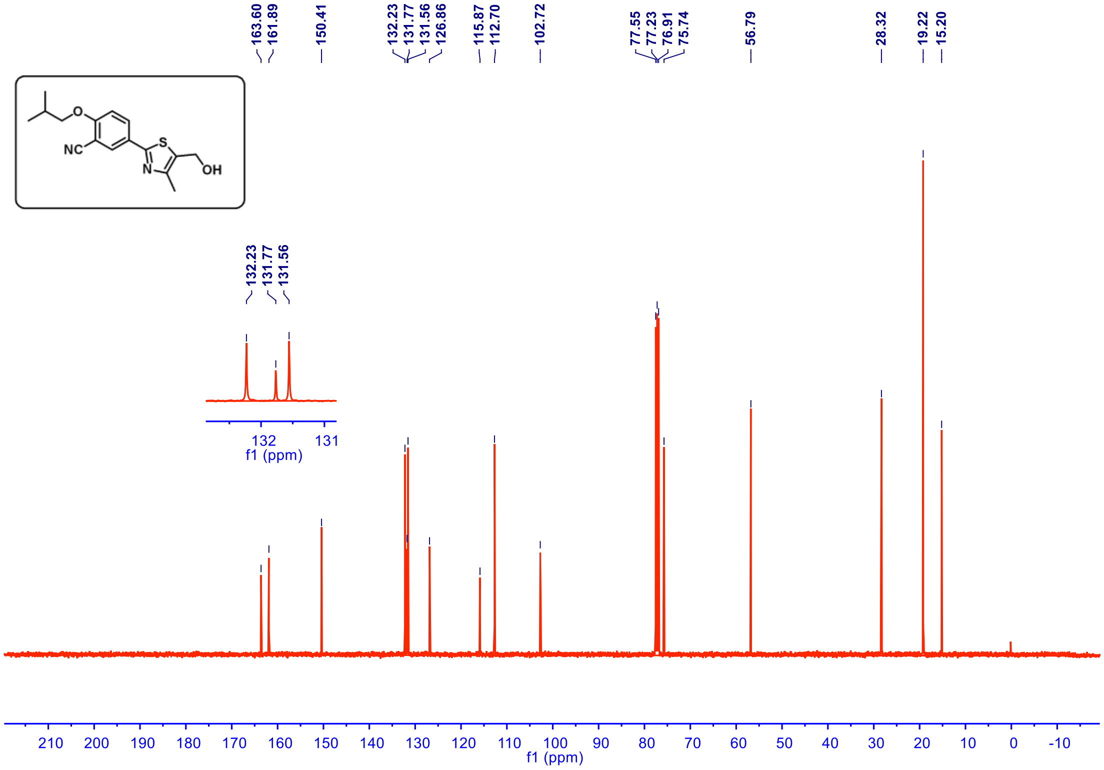

Supplementary Figure 477. ^{13}C NMR spectrum of **5-(5-(hydroxymethyl)-4-methylthiazol-2-yl)-2-isobutoxybenzonitrile** (101 MHz, $CDCl_3$)

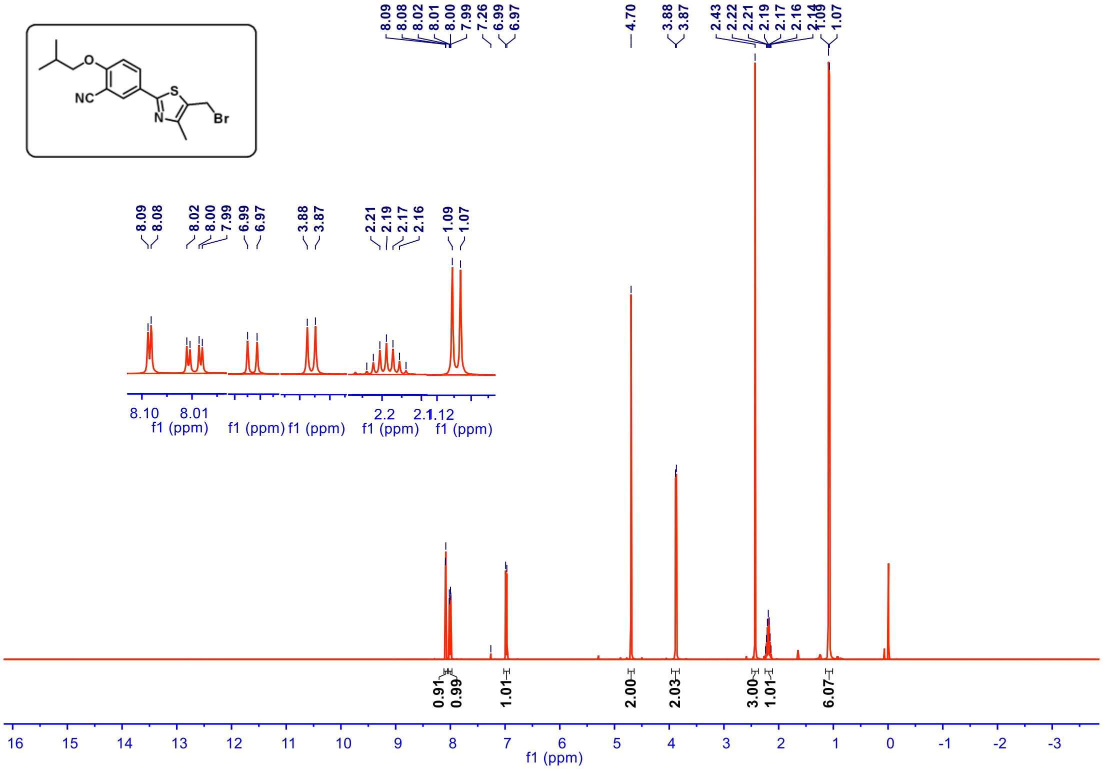

Supplementary Figure 478. ¹H NMR spectrum of **5-(5-(bromomethyl)-4-methylthiazol-2-yl)-2-isobutoxybenzonitrile** (400 MHz, $CDCl_3$)

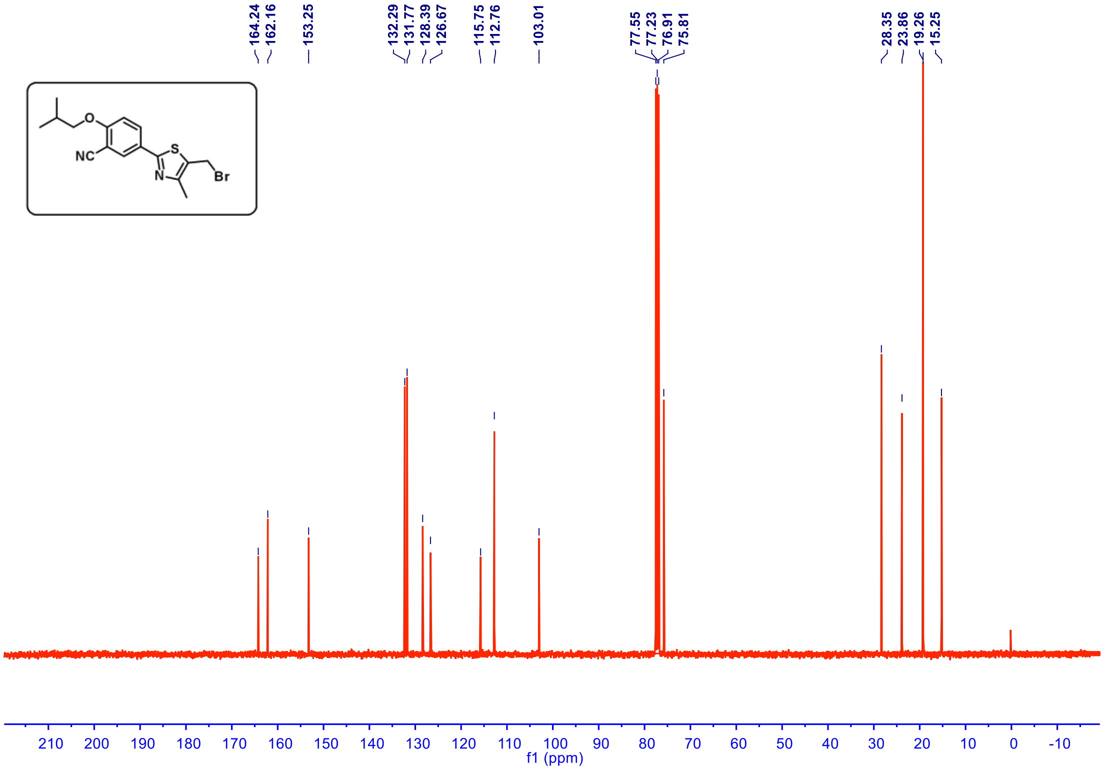

Supplementary Figure 479. ¹³C NMR spectrum of **5-(5-(bromomethyl)-4-methylthiazol-2-yl)-2-isobutoxybenzonitrile** (101 MHz, $CDCl_3$)

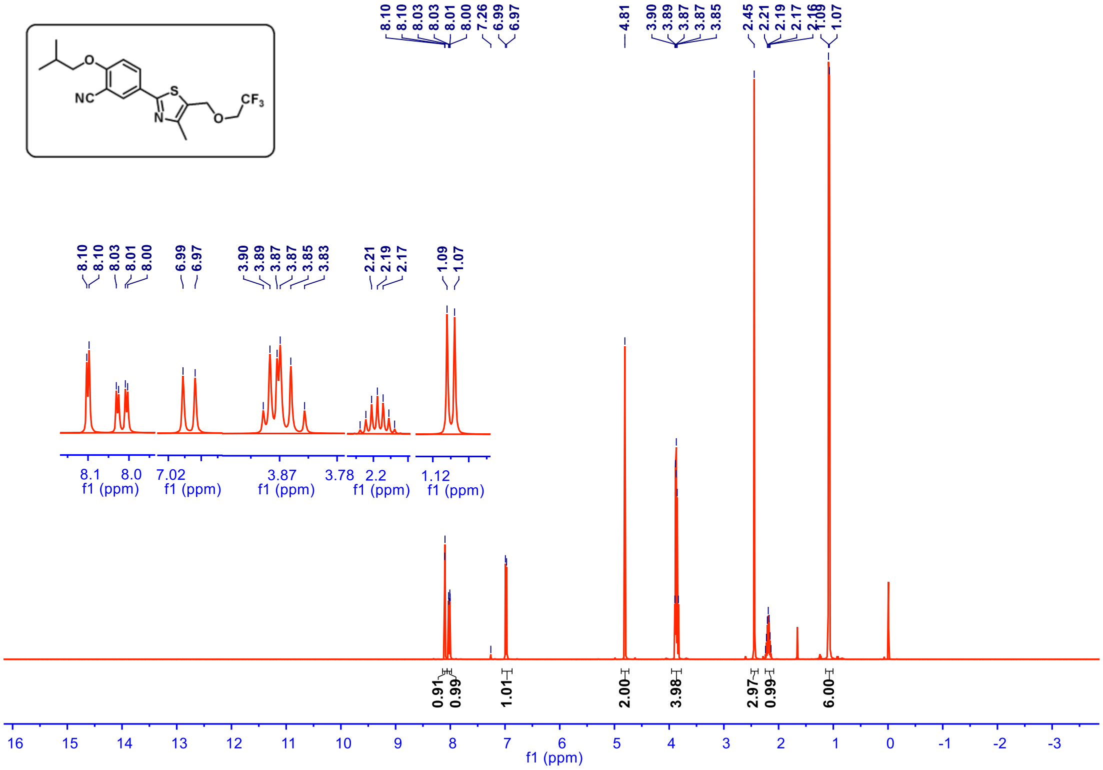

Supplementary Figure 480. ^{1}H NMR spectrum of compound **51** (400 MHz, $CDCl_3$)

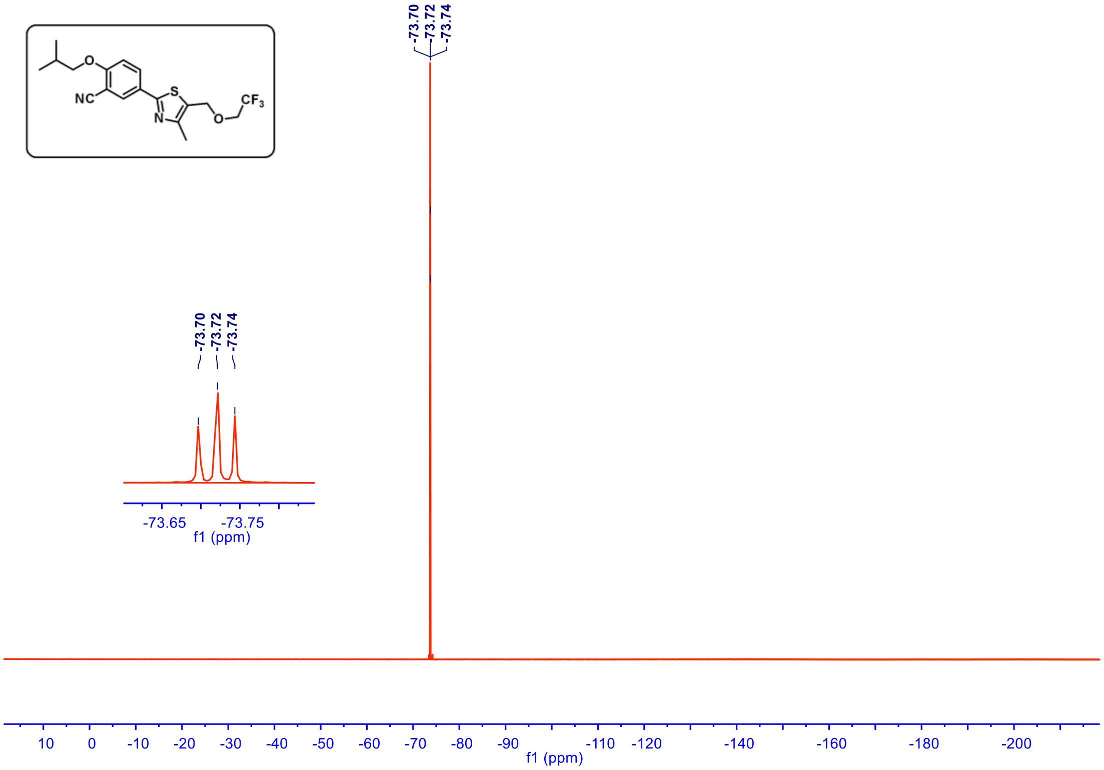

Supplementary Figure 481. ^{19}F NMR spectrum of compound **51** (376 MHz, $CDCl_3$)

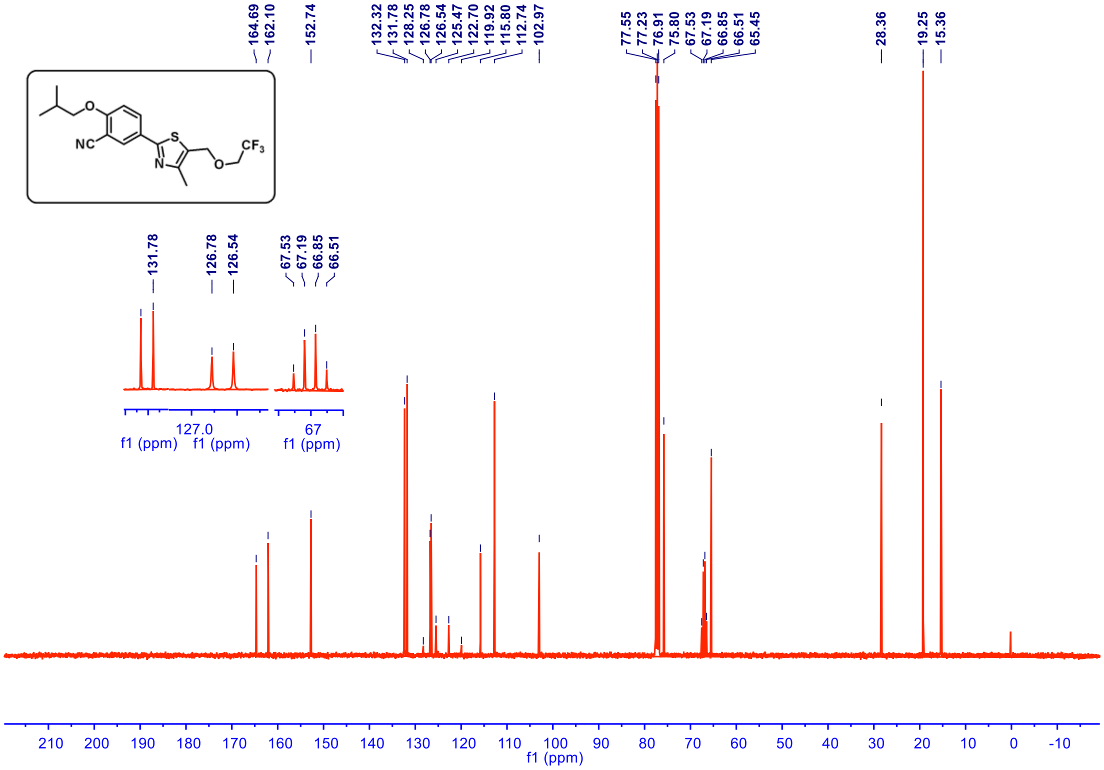

Supplementary Figure 482. ^{13}C NMR spectrum of compound **51** (101 MHz, $CDCl_3$)

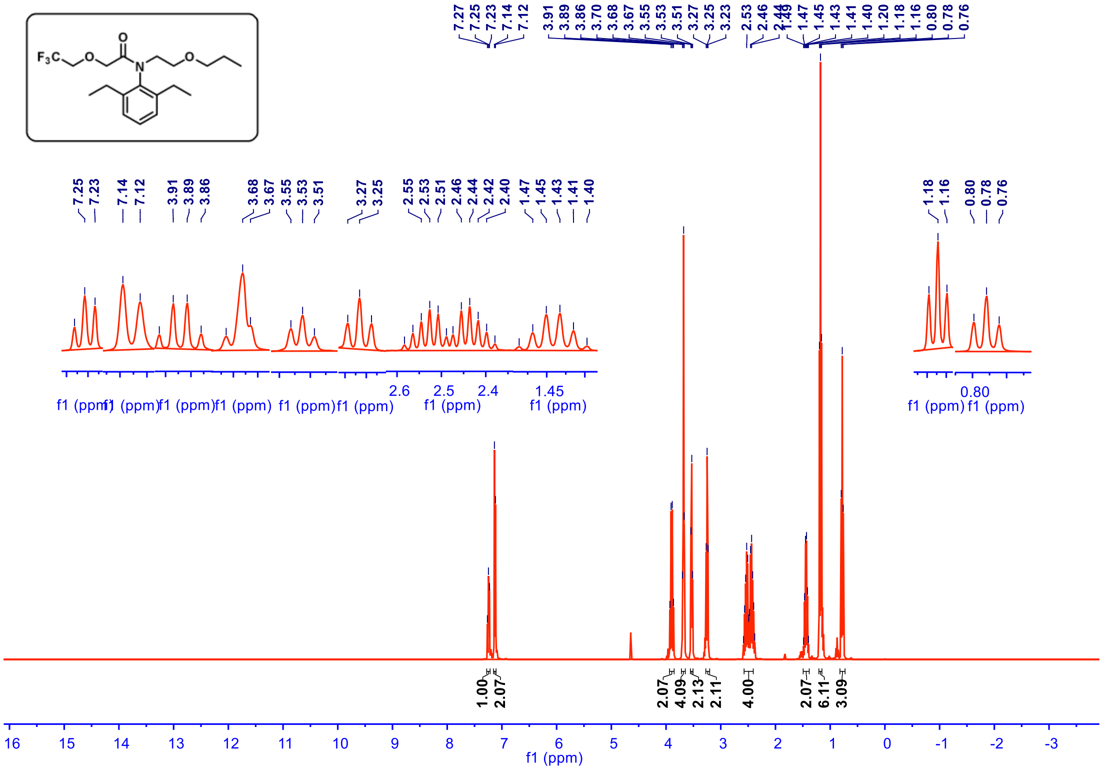

Supplementary Figure 483. ^{1}H NMR spectrum of compound **52** (400 MHz, $CDCl_3$)

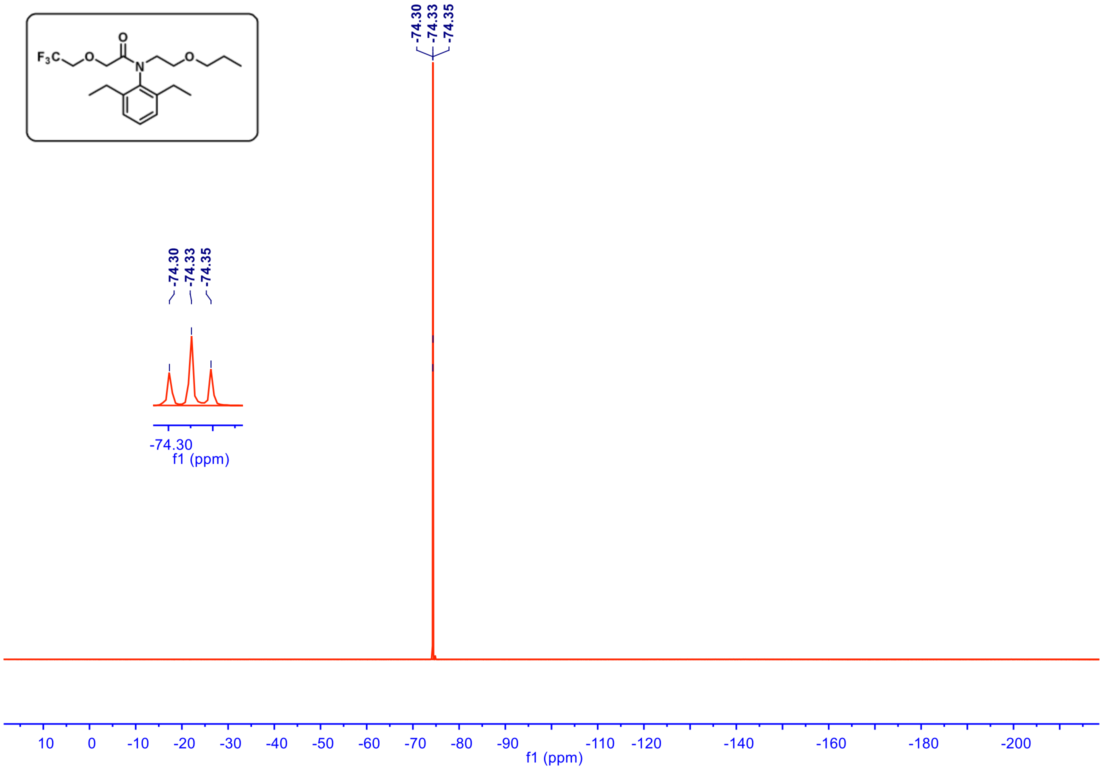

Supplementary Figure 484. ^{19}F NMR spectrum of compound **52** (376 MHz, $CDCl_3$)

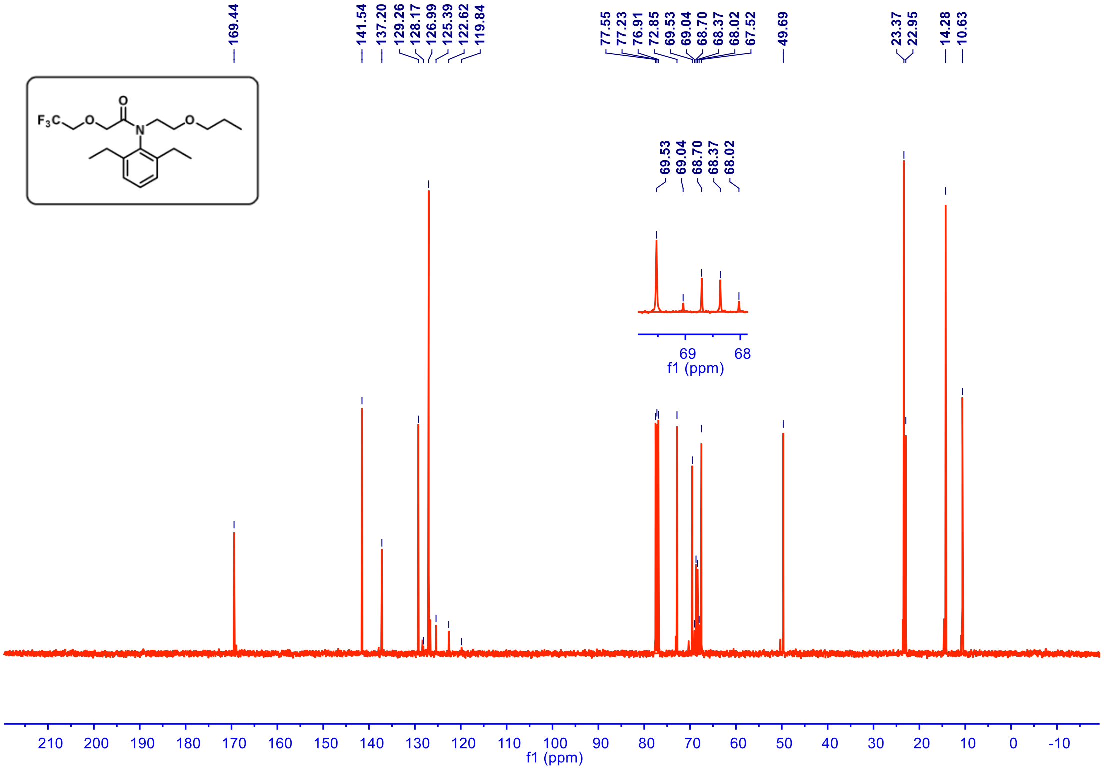

Supplementary Figure 485. ^{13}C NMR spectrum of compound **52** (101 MHz, $CDCl_3$)

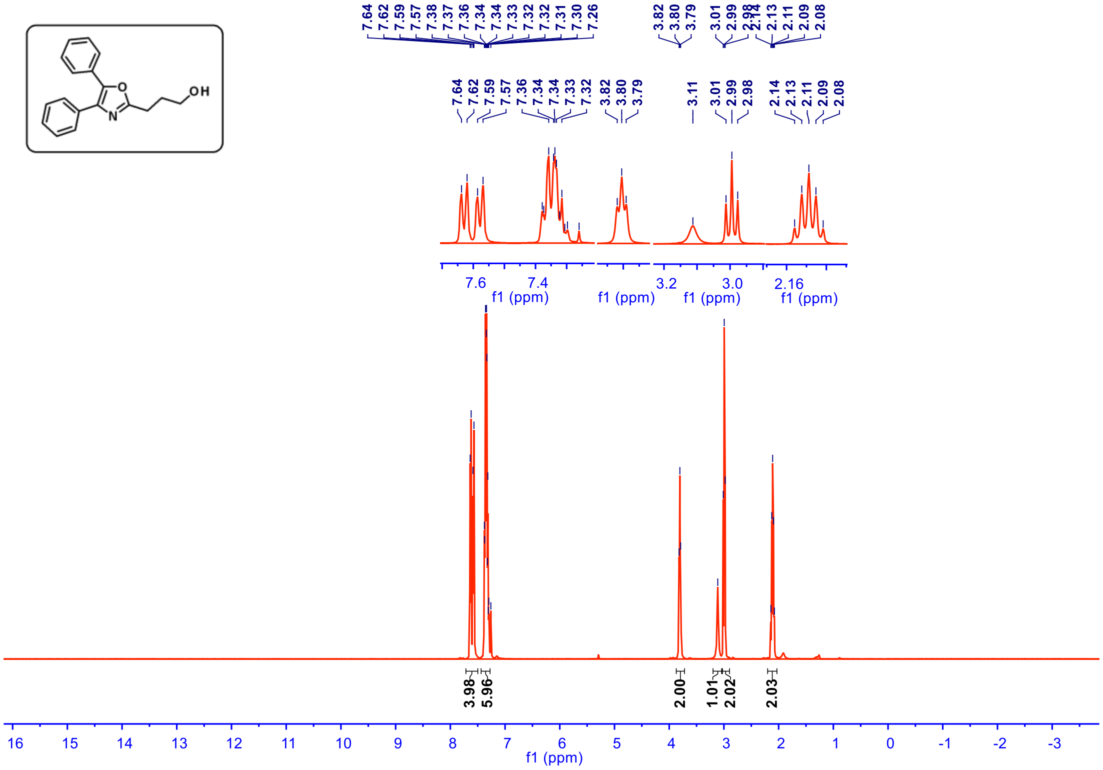

Supplementary Figure 486. ^{1}H NMR spectrum of **3-(4,5-diphenyloxazol-2-yl)propan-1-ol** (400 MHz, $CDCl_3$)

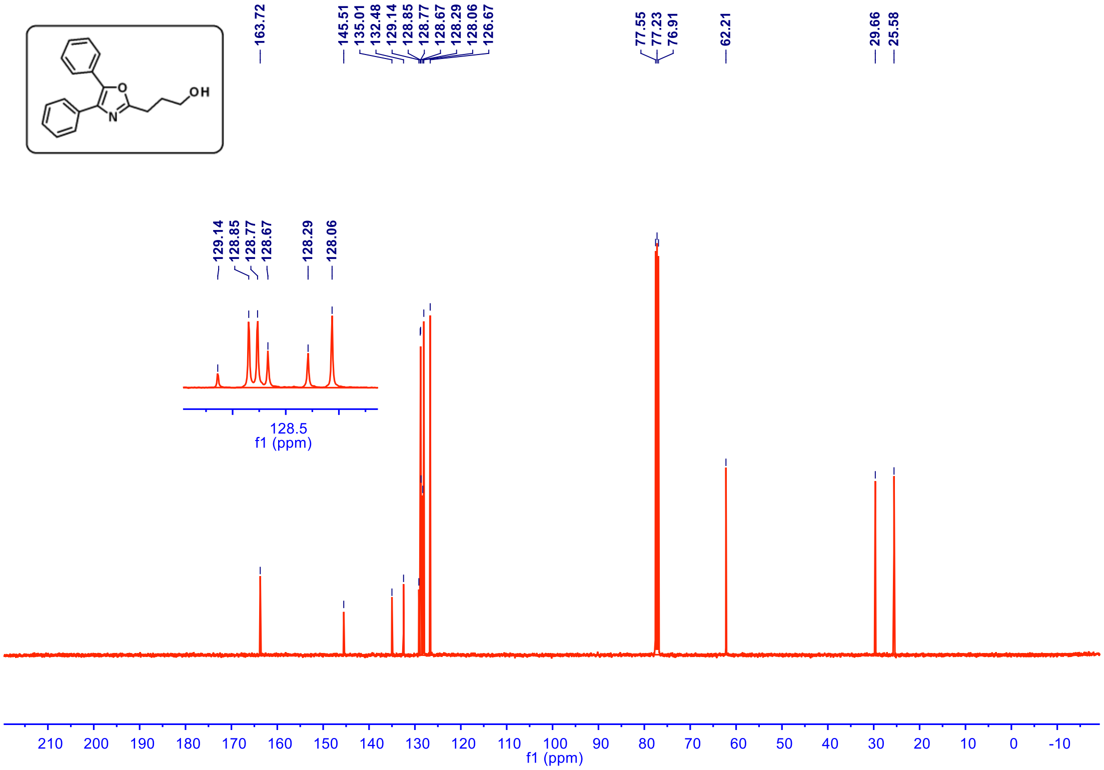

Supplementary Figure 487. ^{13}C NMR spectrum of **3-(4,5-diphenyloxazol-2-yl)propan-1-ol** (101 MHz, $CDCl_3$)

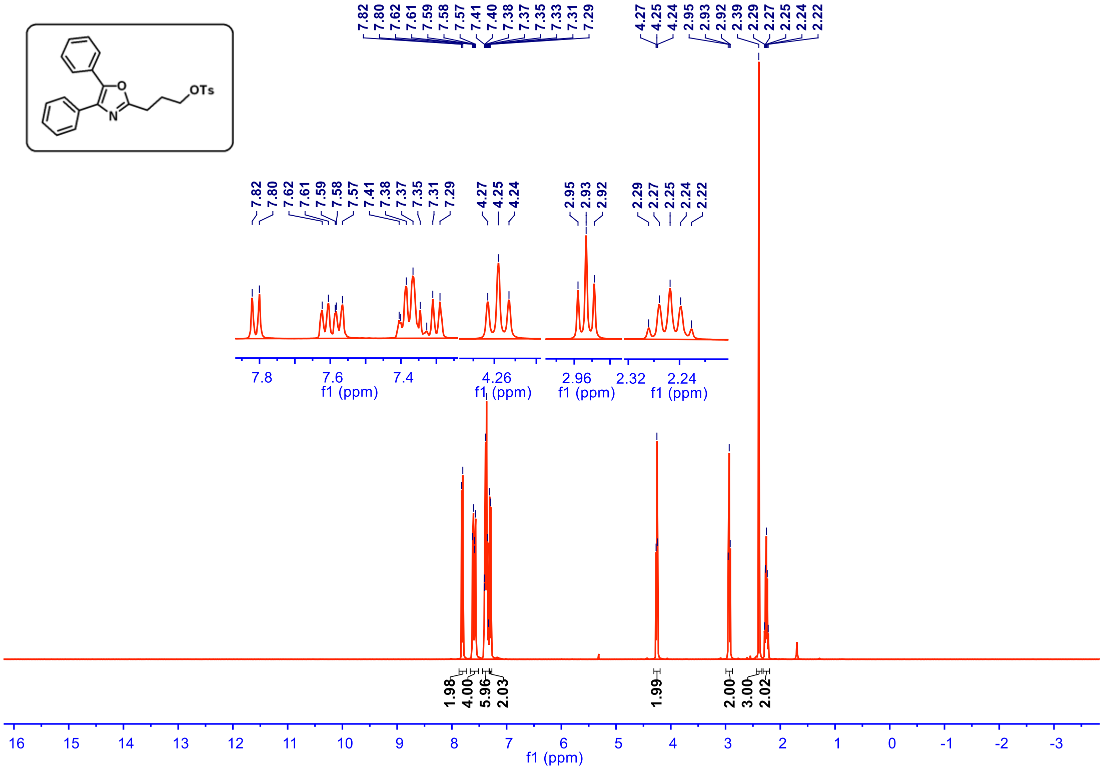

Supplementary Figure 488. ^{1}H NMR spectrum of **3-(4,5-diphenyloxazol-2-yl)propyl 4-methylbenzenesulfonate** (400 MHz, $CDCl_3$)

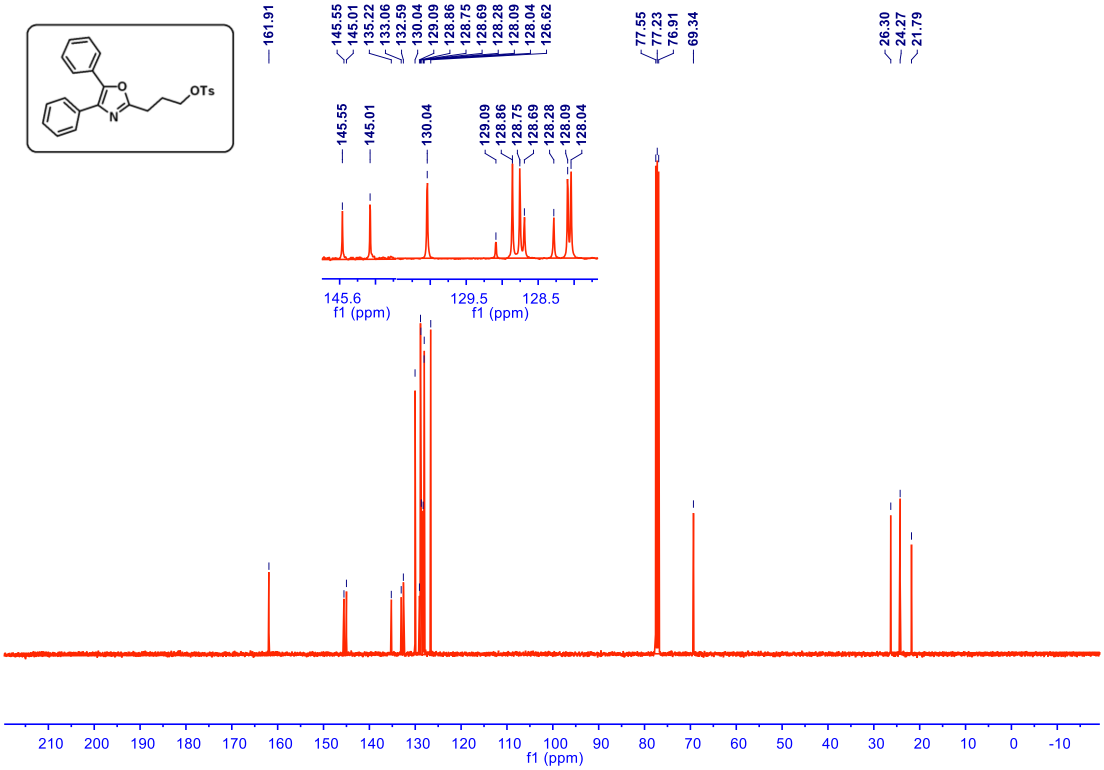

Supplementary Figure 489. ^{13}C NMR spectrum of **3-(4,5-diphenyloxazol-2-yl)propyl 4-methylbenzenesulfonate** (101 MHz, $CDCl_3$)

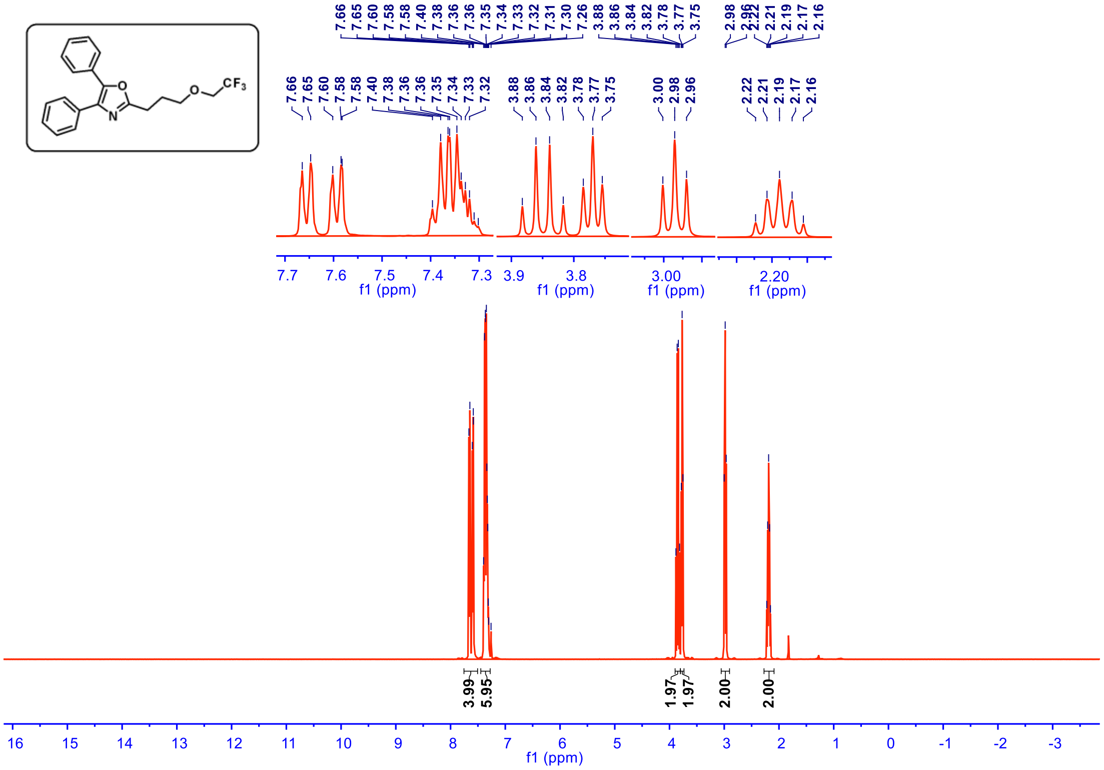

Supplementary Figure 490. ^{1}H NMR spectrum of compound **53** (400 MHz, $CDCl_3$)

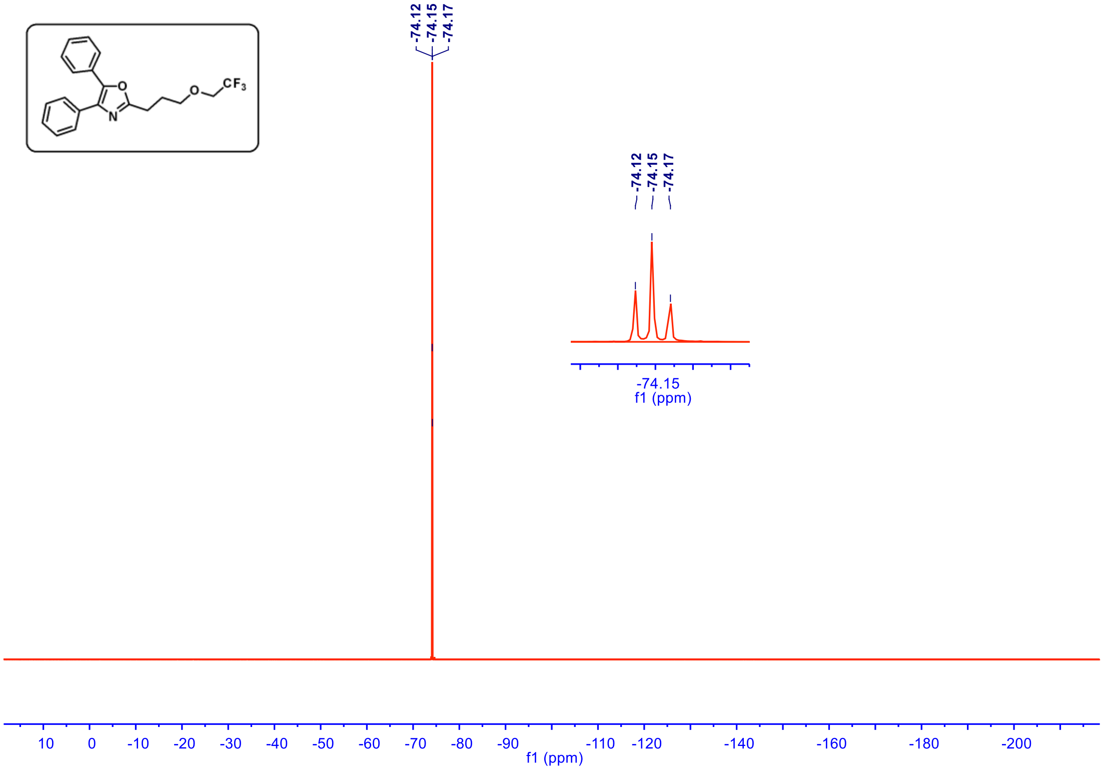

Supplementary Figure 491. ^{19}F NMR spectrum of compound **53** (376 MHz, $CDCl_3$)

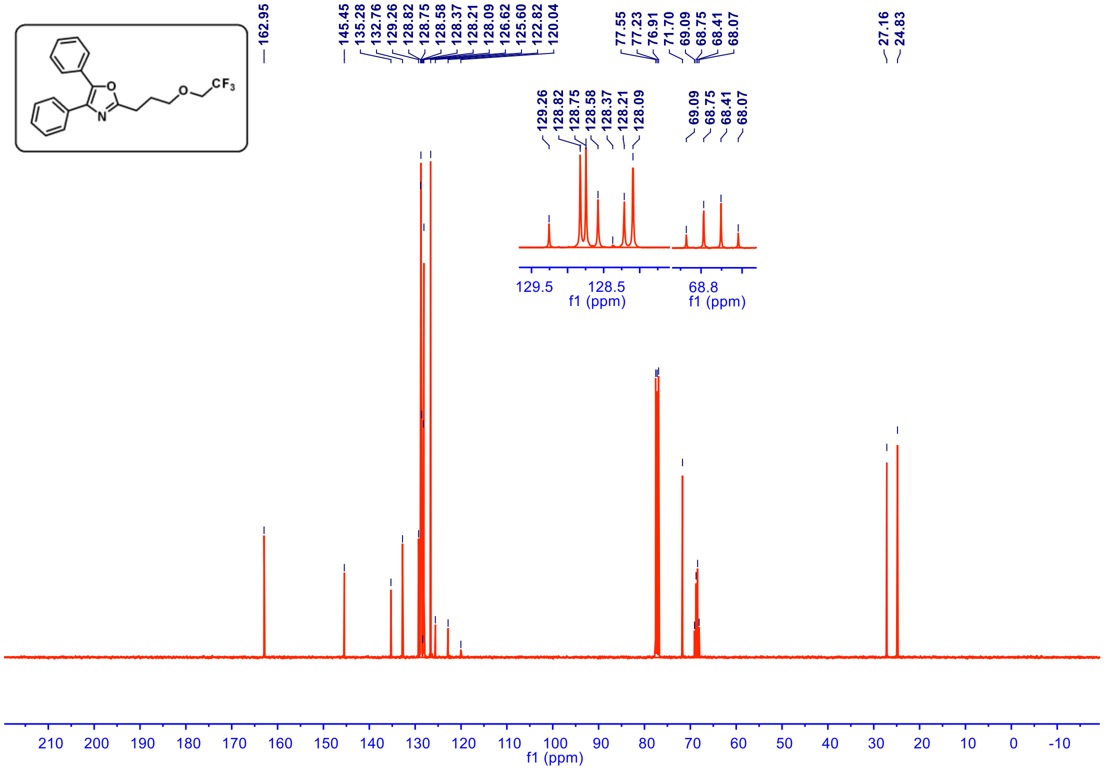

Supplementary Figure 492. ^{13}C NMR spectrum of compound **53** (101 MHz, $CDCl_3$)

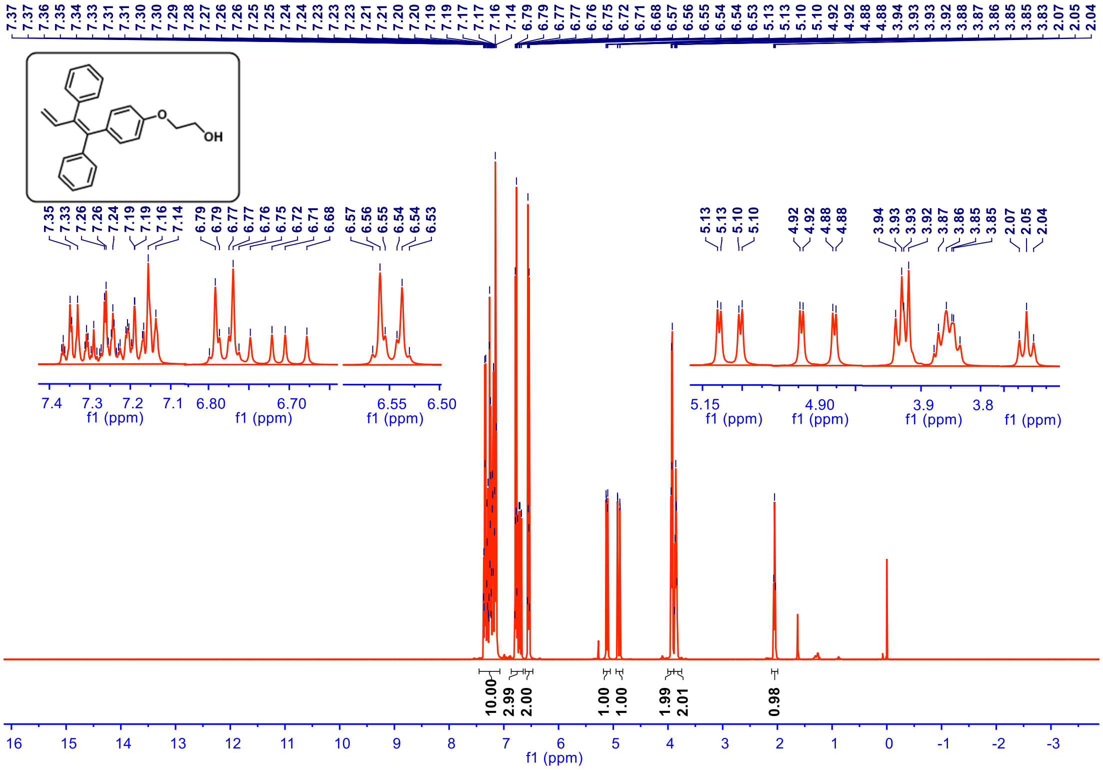

Supplementary Figure 493. ^{1}H NMR spectrum of **(*Z*)-2-(4-(1,2-diphenylbuta-1,3-dien-1-yl)phenoxy)ethan-1-ol** (400 MHz, $CDCl_3$)

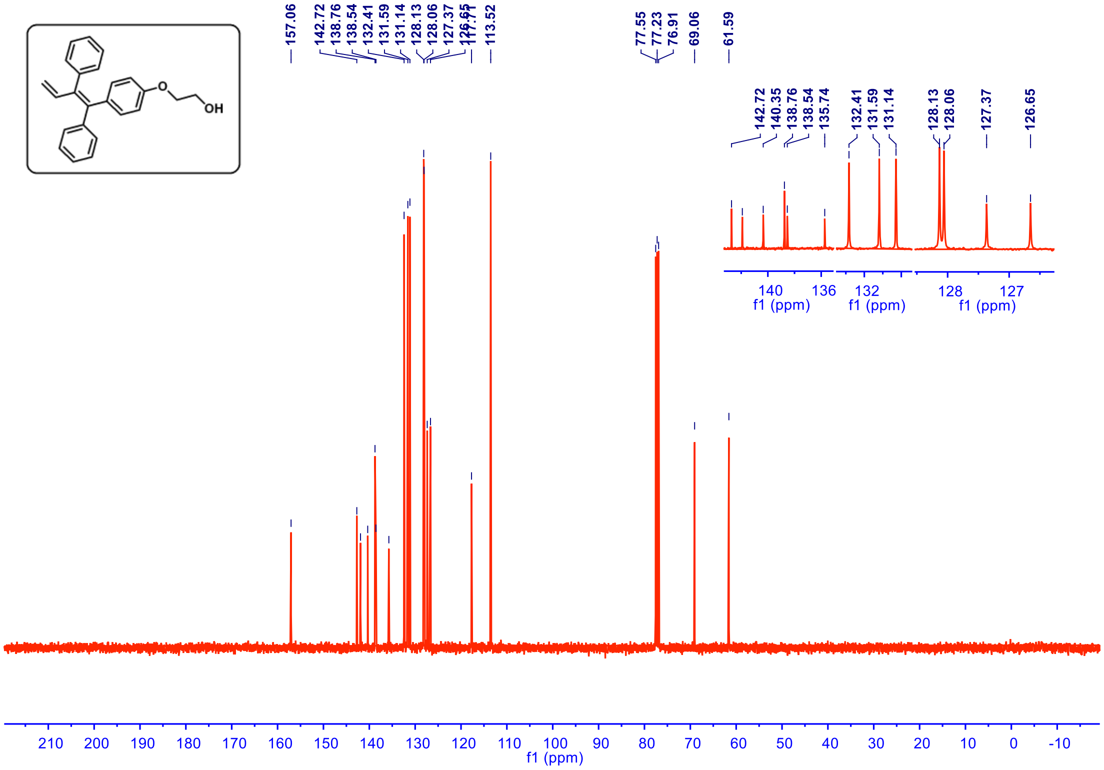

Supplementary Figure 494. ^{13}C NMR spectrum of **(*Z*)-2-(4-(1,2-diphenylbuta-1,3-dien-1-yl)phenoxy)ethan-1-ol** (101 MHz, $CDCl_3$)

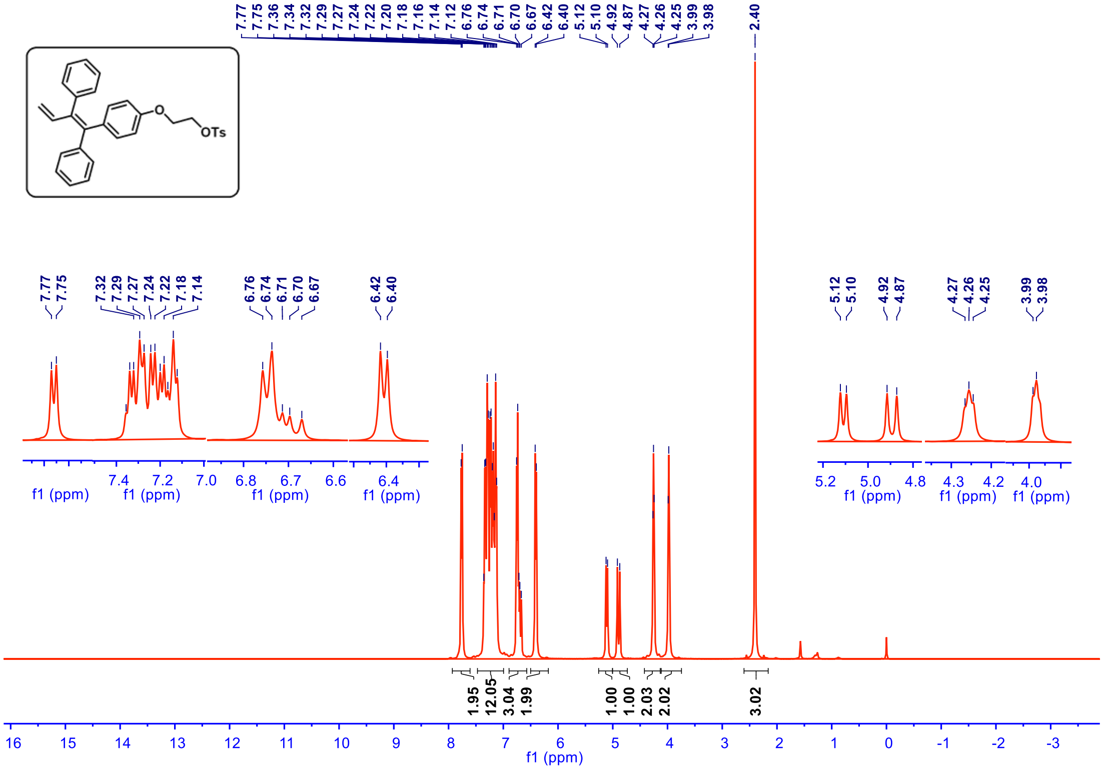

Supplementary Figure 495. ^{1}H NMR spectrum of **(*Z*)-2-(4-(1,2-diphenylbuta-1,3-dien-1-yl)phenoxy)ethyl 4-methylbenzenesulfonate** (400 MHz, $CDCl_3$)

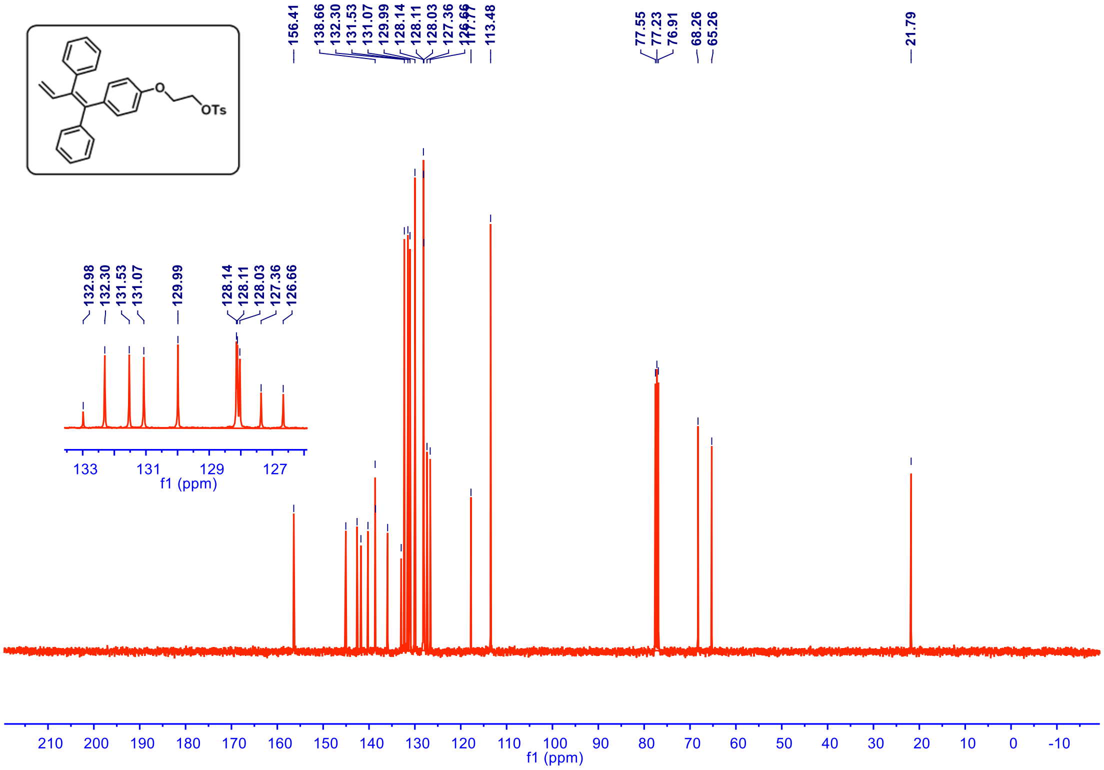

Supplementary Figure 496. ^{13}C NMR spectrum of **(*Z*)-2-(4-(1,2-diphenylbuta-1,3-dien-1-yl)phenoxy)ethyl 4-methylbenzenesulfonate** (101 MHz, $CDCl_3$)

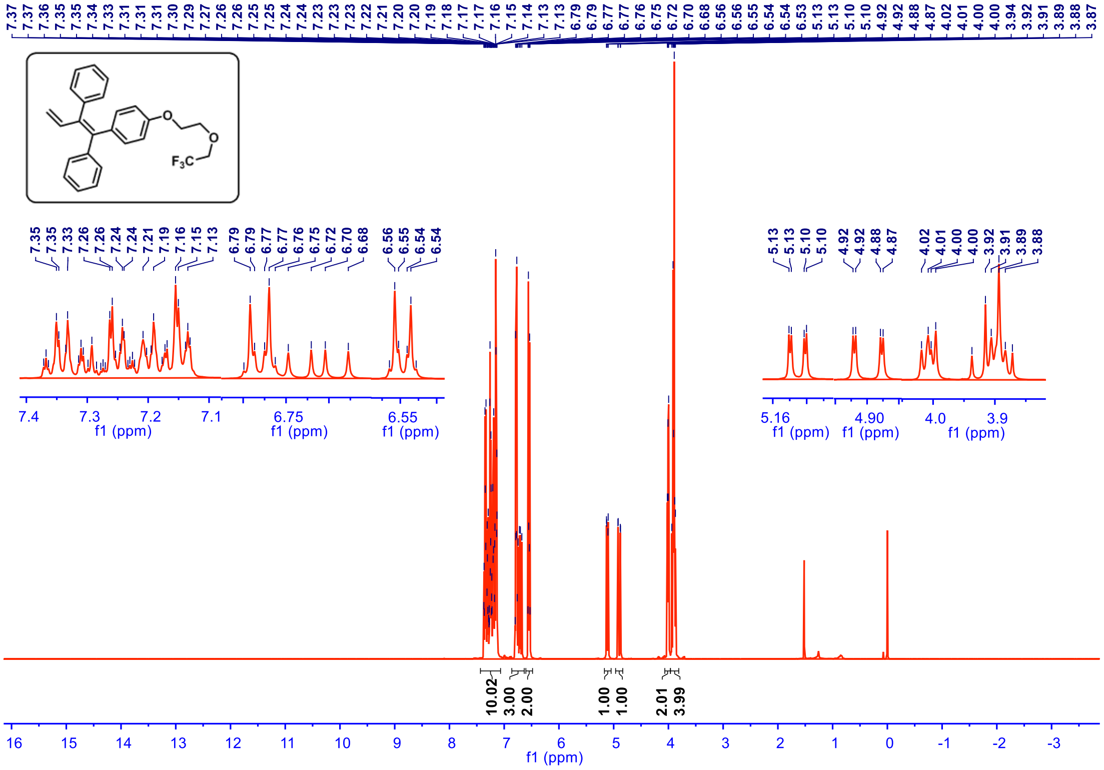

Supplementary Figure 497. ^{1}H NMR spectrum of compound **54** (400 MHz, $CDCl_3$)

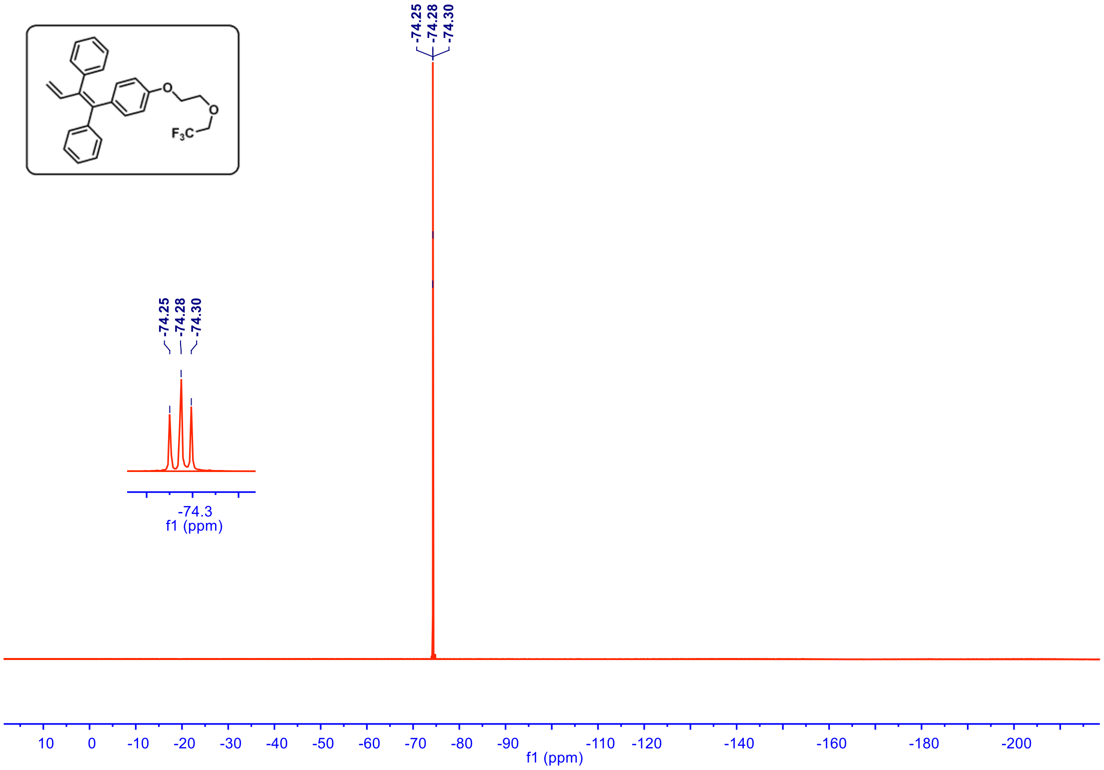

Supplementary Figure 498. ^{19}F NMR spectrum of compound **54** (376 MHz, $CDCl_3$)

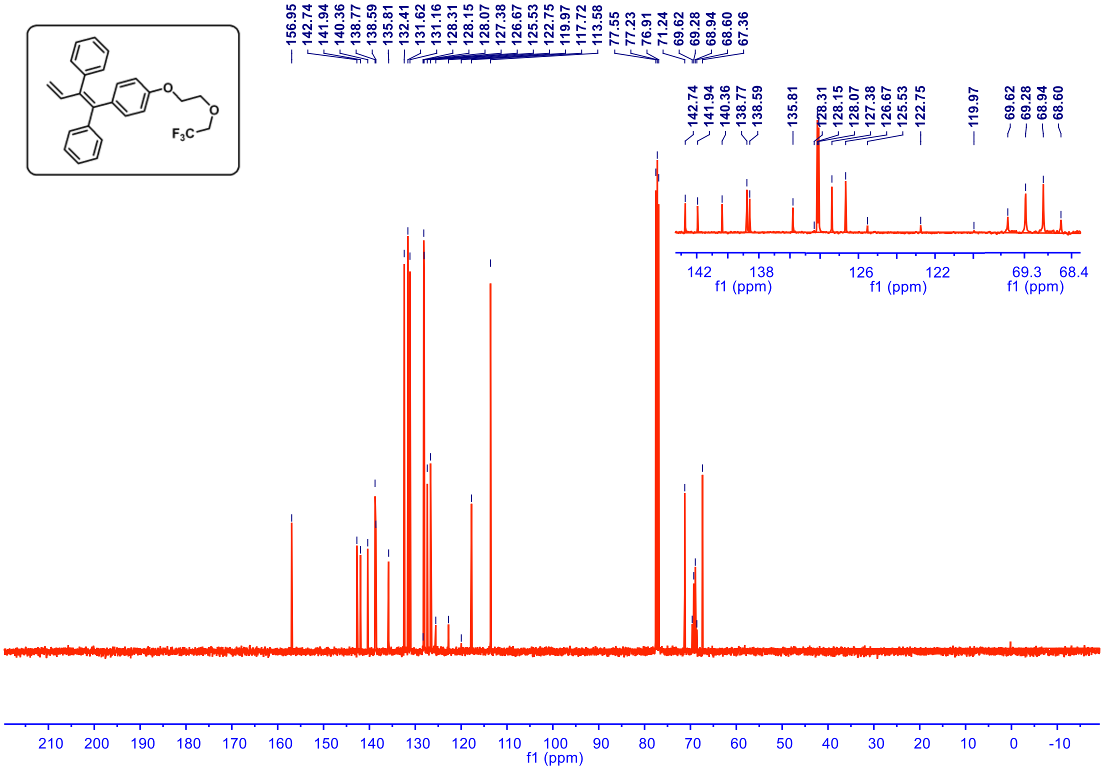

Supplementary Figure 499. ^{13}C NMR spectrum of compound **54** (101 MHz, $CDCl_3$)

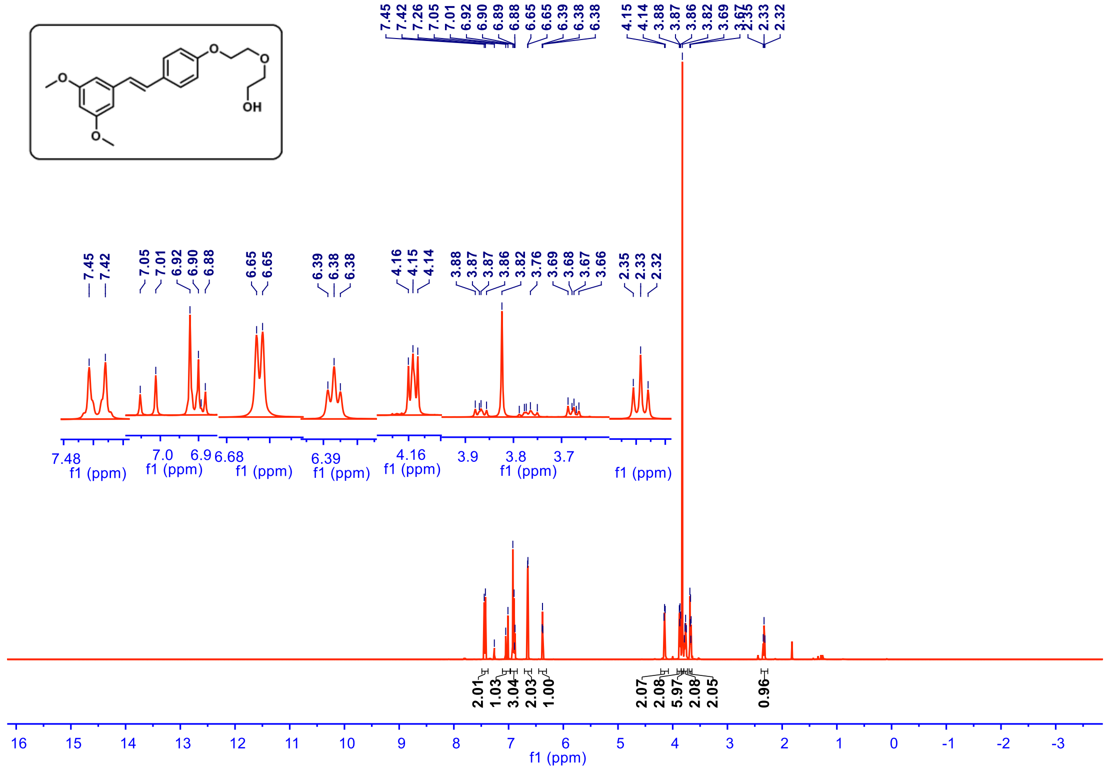

Supplementary Figure 500. ^{1}H NMR spectrum of **(*E*)-2-(2-(4-(3,5-dimethoxystyryl)phenoxy)ethoxy)ethanol** (400 MHz, $CDCl_3$)

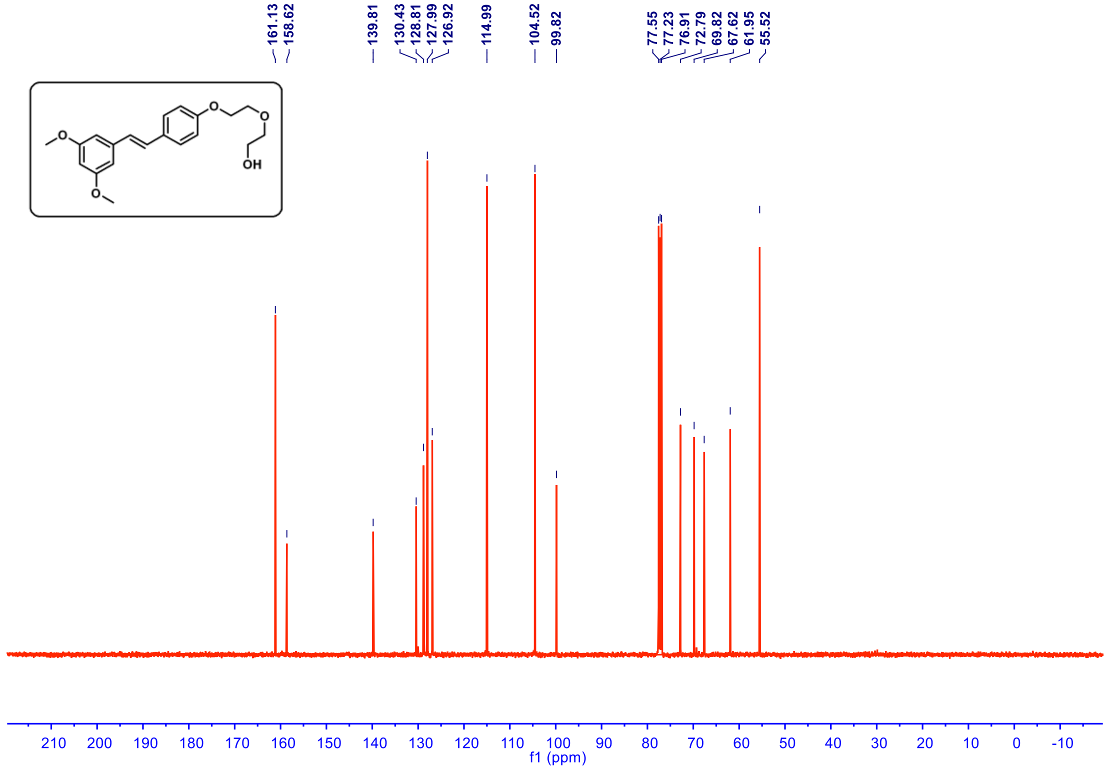

Supplementary Figure 501. ^{13}C NMR spectrum of **(*E*)-2-(2-(4-(3,5-dimethoxystyryl)phenoxy)ethoxy)ethanol** (101 MHz, $CDCl_3$)

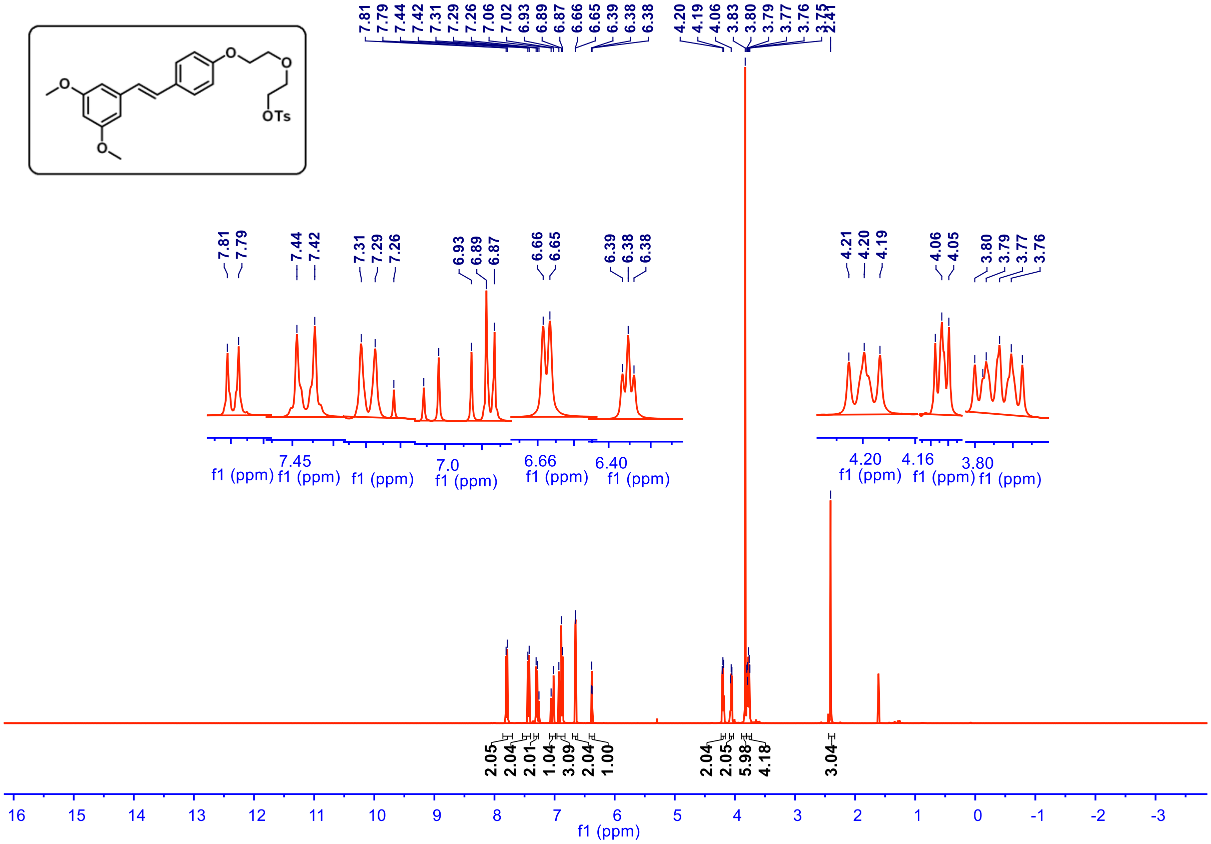

Supplementary Figure 502. ^{1}H NMR spectrum of **(*E*)-2-(2-(4-(3,5-dimethoxystyryl)phenoxy)ethoxy)ethyl 4-methylbenzenesulfonate** (400 MHz, $CDCl_3$)

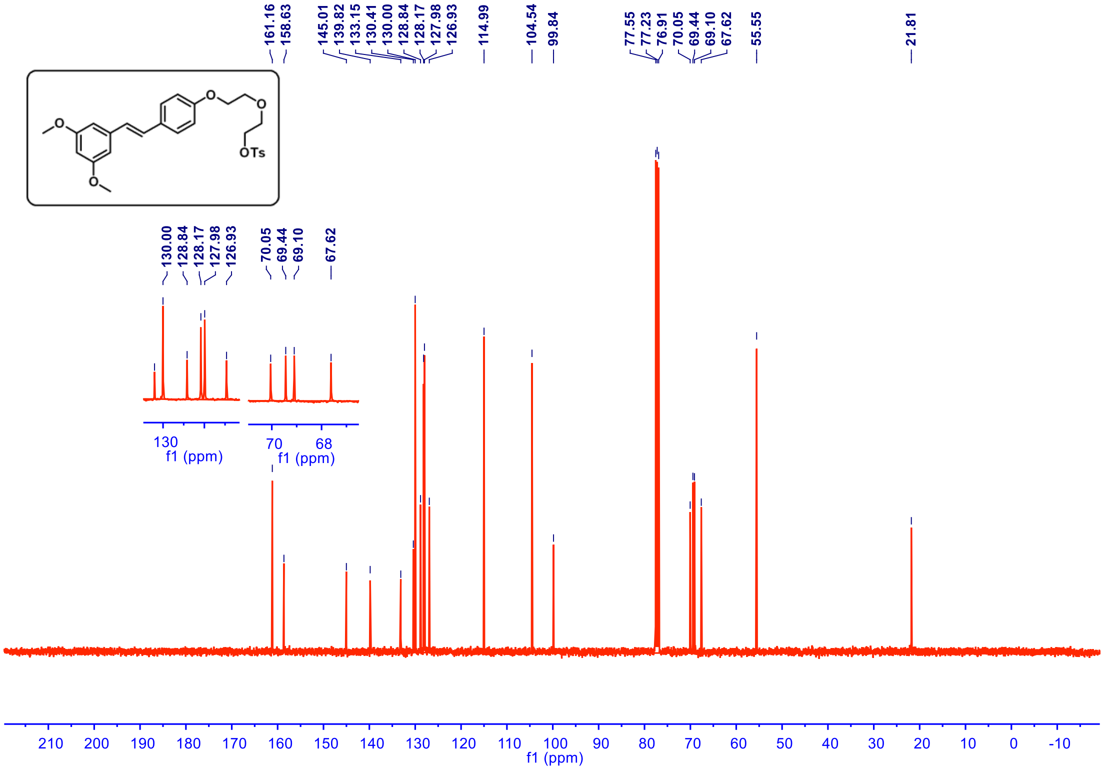

Supplementary Figure 503. ^{13}C NMR spectrum of **(*E*)-2-(2-(4-(3,5-dimethoxystyryl)phenoxy)ethoxy)ethyl 4-methylbenzenesulfonate** (101 MHz, $CDCl_3$)

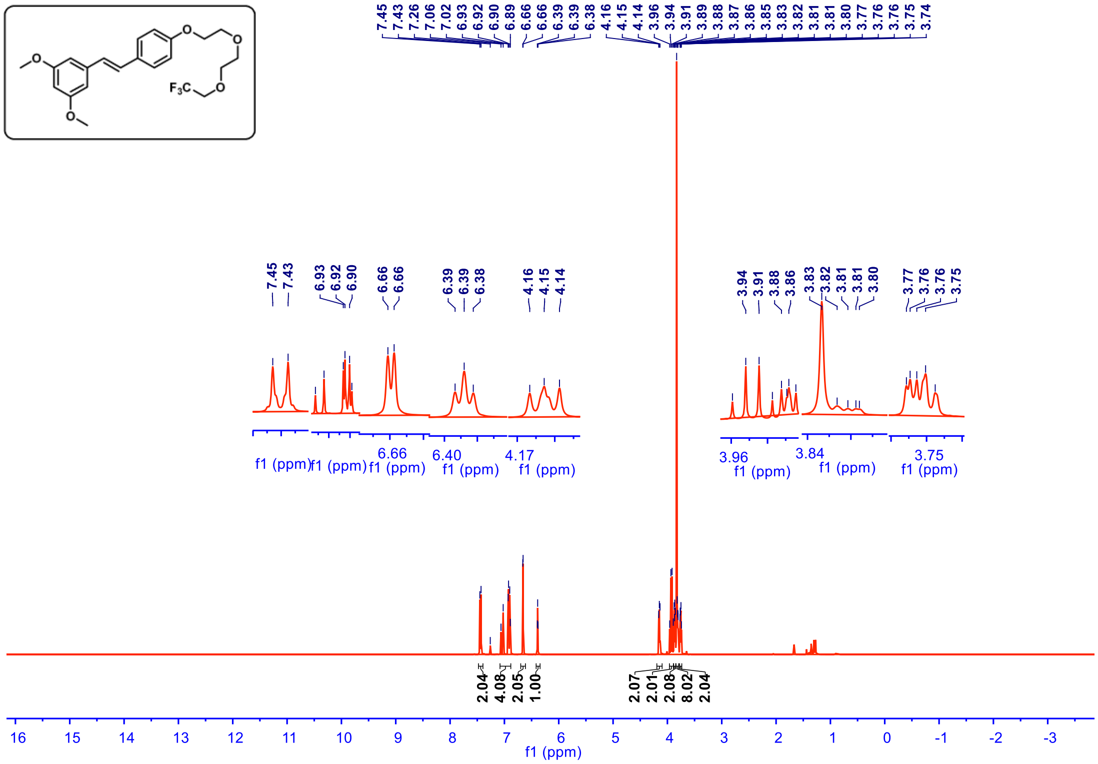

Supplementary Figure 504. ^{1}H NMR spectrum of compound **55** (400 MHz, $CDCl_3$)

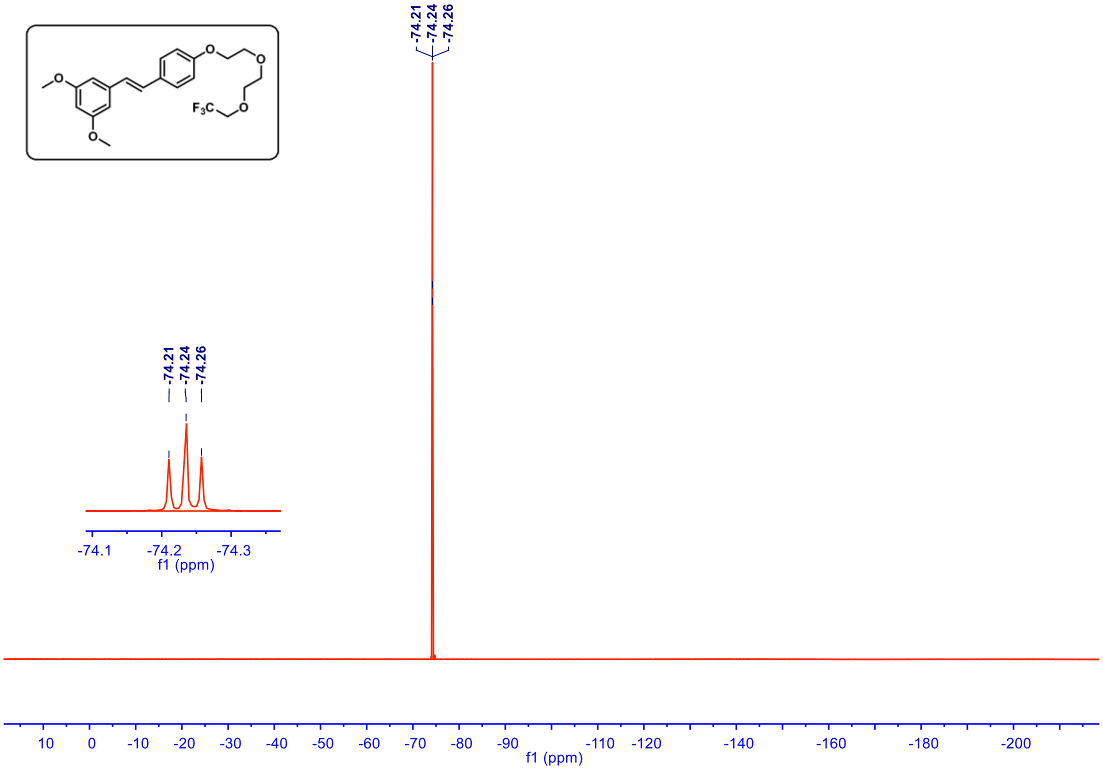

Supplementary Figure 505. ^{19}F NMR spectrum of compound **55** (376 MHz, $CDCl_3$)

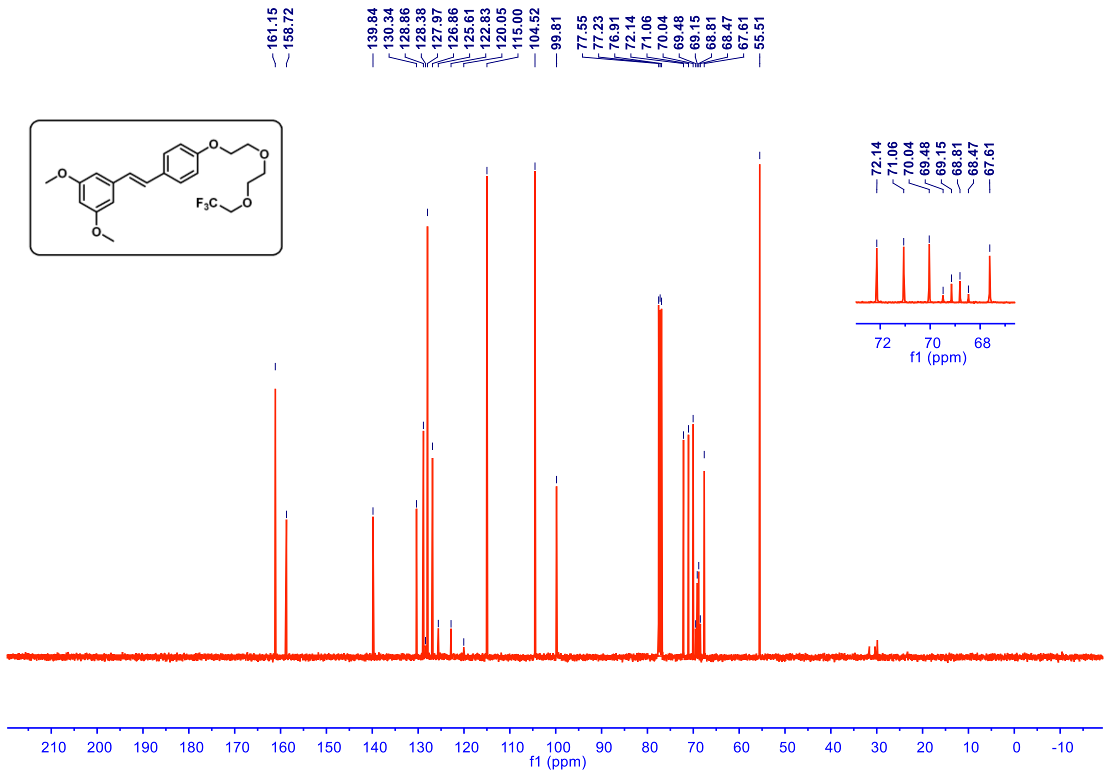

Supplementary Figure 506. ^{13}C NMR spectrum of compound **55** (101 MHz, $CDCl_3$)

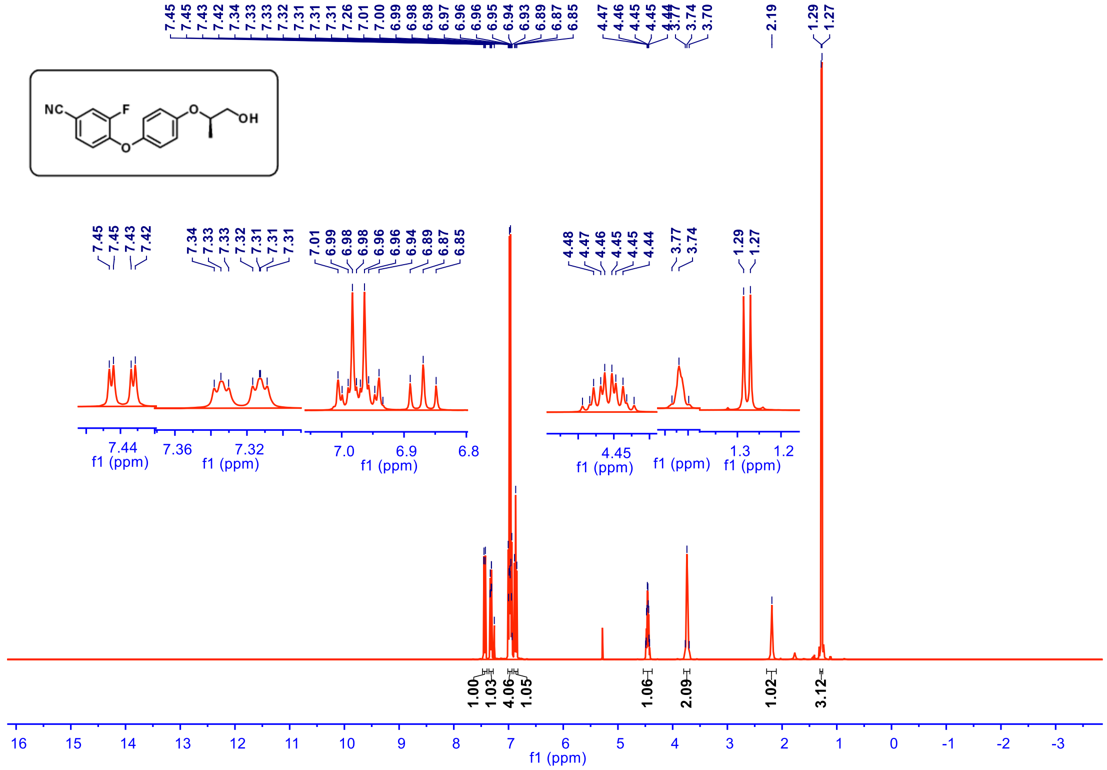

Supplementary Figure 507. ^{1}H NMR spectrum of **(*R*)-3-fluoro-4-(4-(1-hydroxypropan-2-yloxy)phenoxy)benzonitrile** (400 MHz, $CDCl_3$)

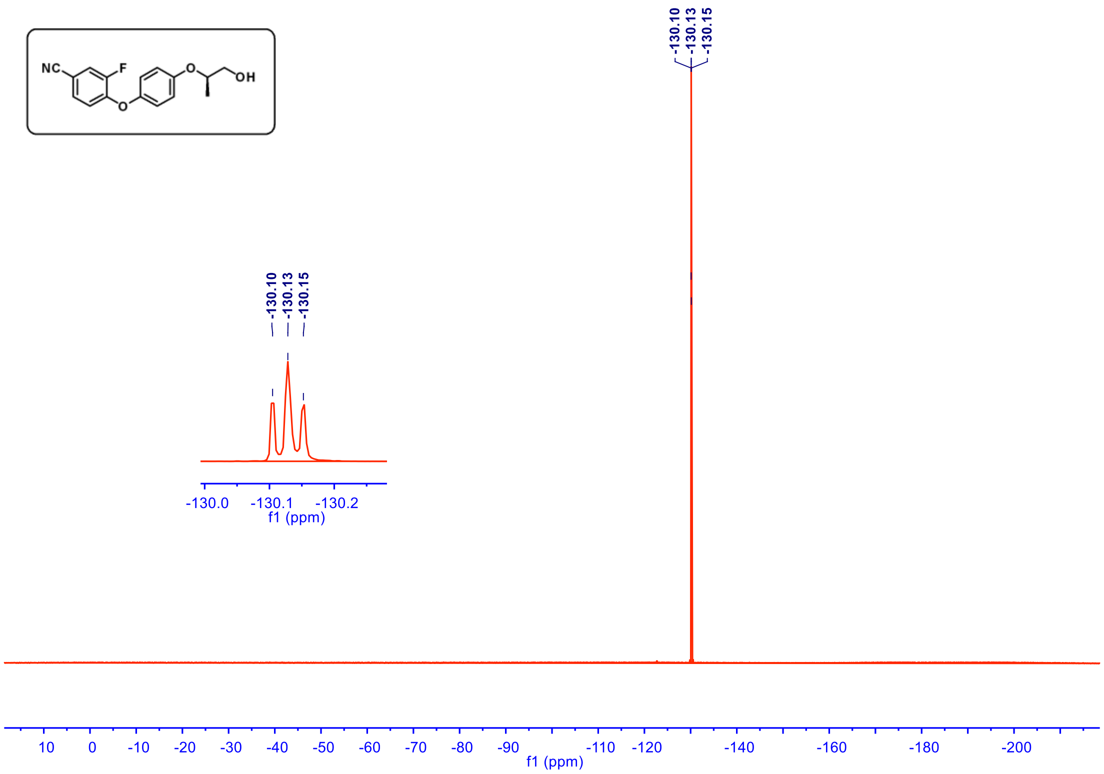

Supplementary Figure 508. ^{19}F NMR spectrum of **(*R*)-3-fluoro-4-(4-(1-hydroxypropan-2-yloxy)phenoxy)benzonitrile** (376 MHz, $CDCl_3$)

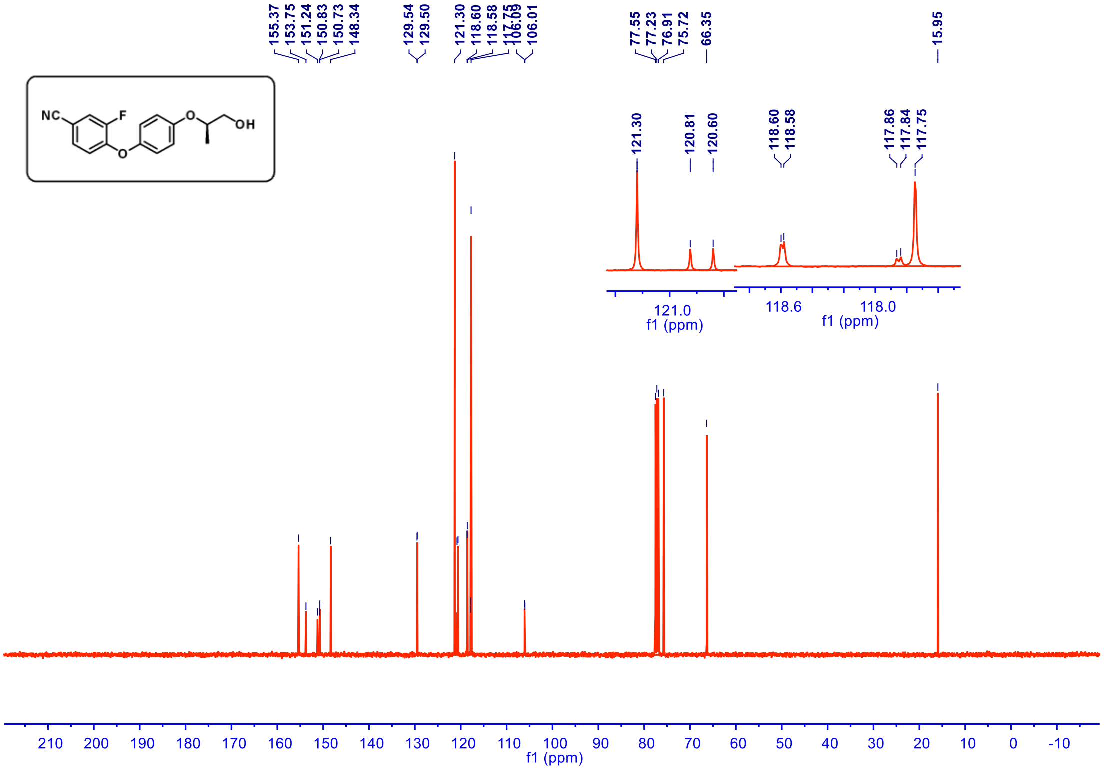

Supplementary Figure 509. ^{13}C NMR spectrum of **(*R*)-3-fluoro-4-(4-(1-hydroxypropan-2-yloxy)phenoxy)benzonitrile** (101 MHz, $CDCl_3$)

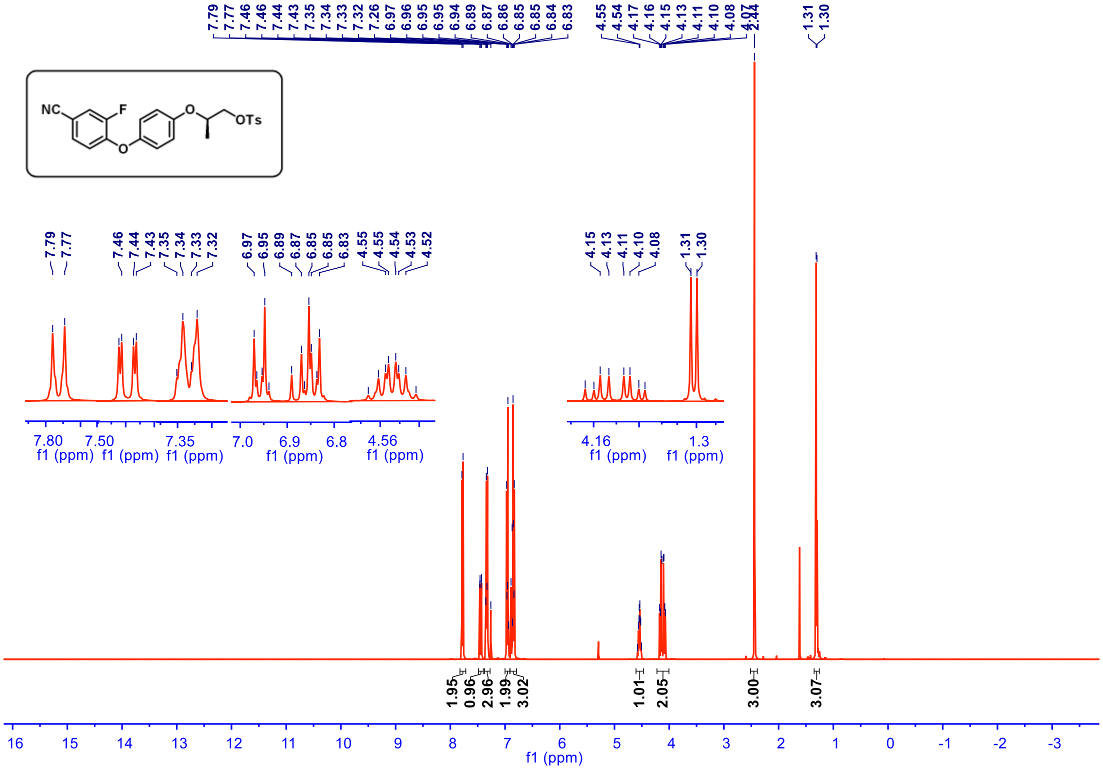

Supplementary Figure 510. ^{1}H NMR spectrum of **(*R*)-2-(4-(4-Cyano-2-fluorophenoxy)phenoxy)propyl 4-methylbenzenesulfonate** (400 MHz, $CDCl_3$)

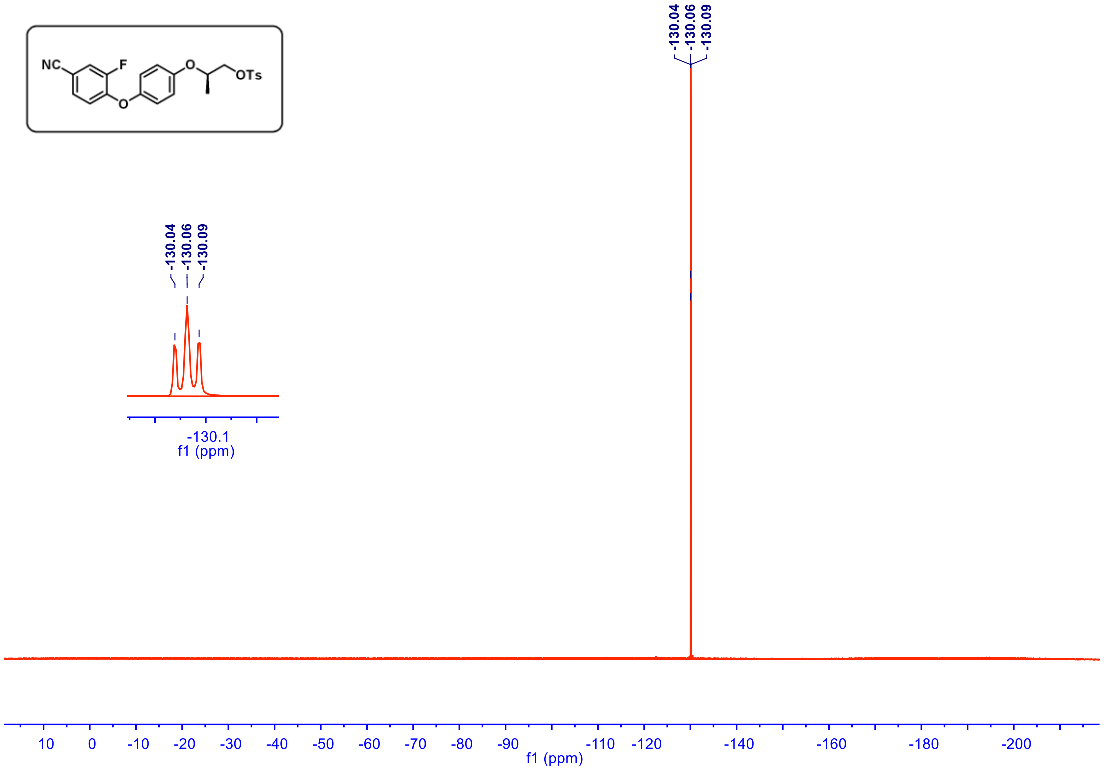

Supplementary Figure 511. ^{19}F NMR spectrum of **(*R*)-2-(4-(4-Cyano-2-fluorophenoxy)phenoxy)propyl 4-methylbenzenesulfonate** (376 MHz, $CDCl_3$)

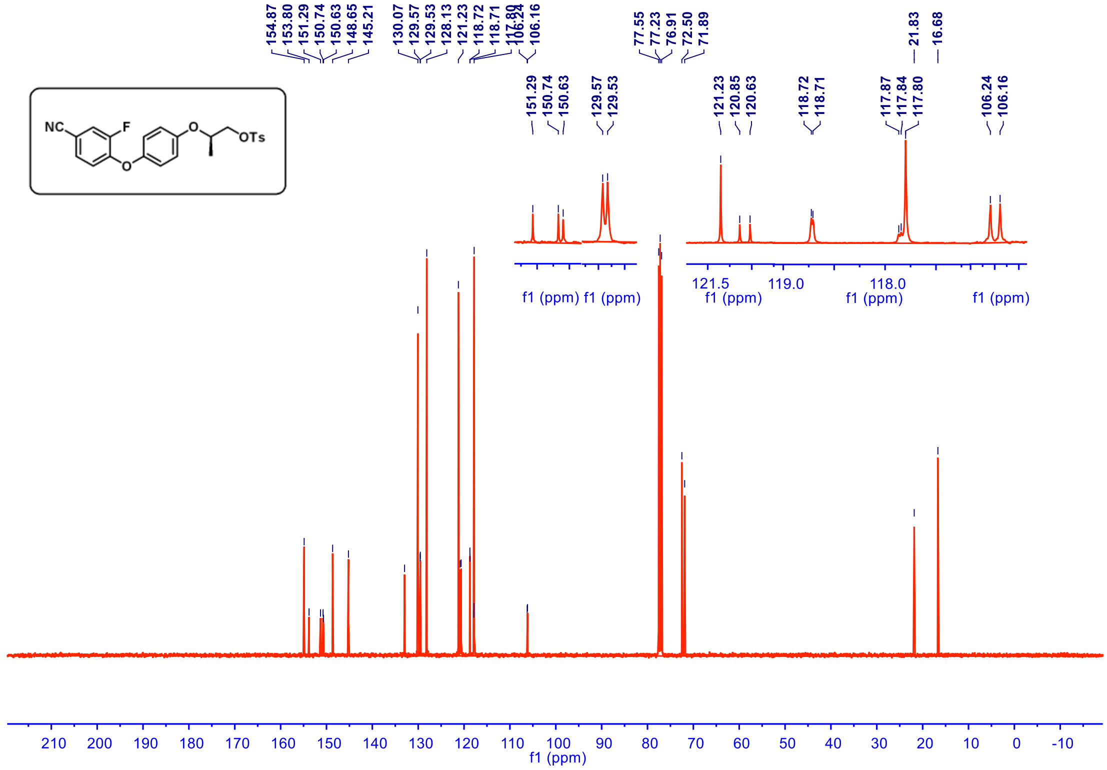

Supplementary Figure 512. ^{13}C NMR spectrum of **(*R*)-2-(4-(4-Cyano-2-fluorophenoxy)phenoxy)propyl 4-methylbenzenesulfonate** (101 MHz, $CDCl_3$)

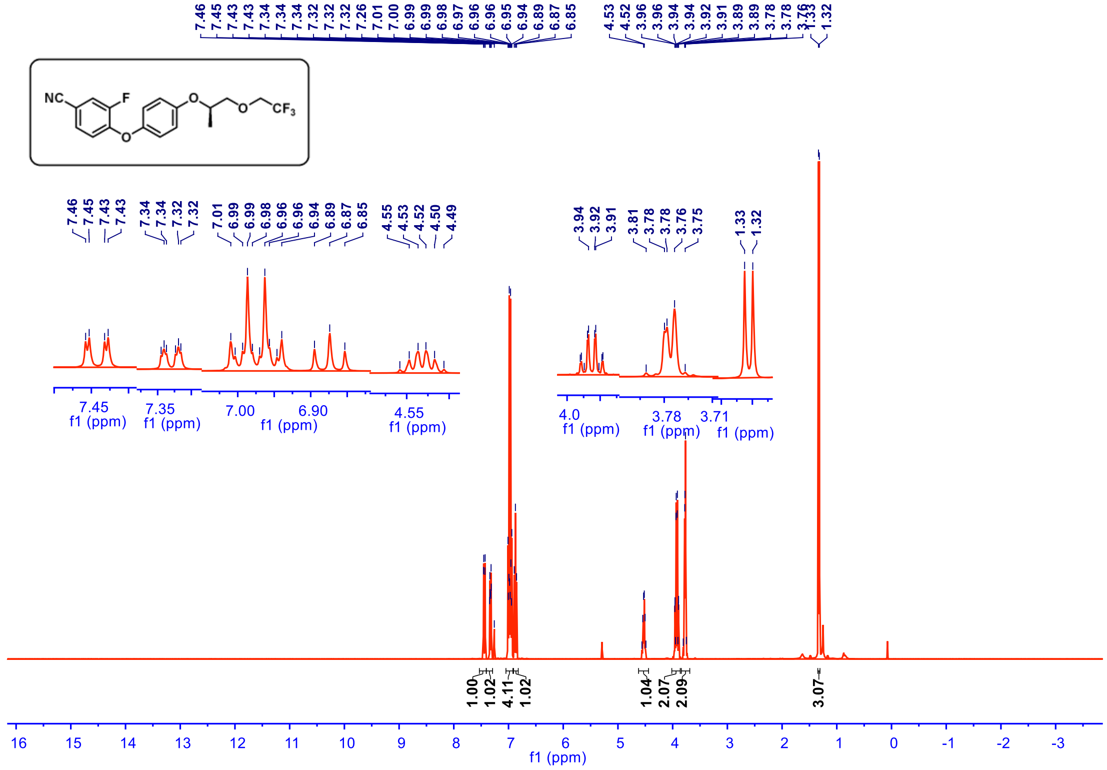

Supplementary Figure 513. ^{1}H NMR spectrum of compound **56** (400 MHz, $CDCl_3$)

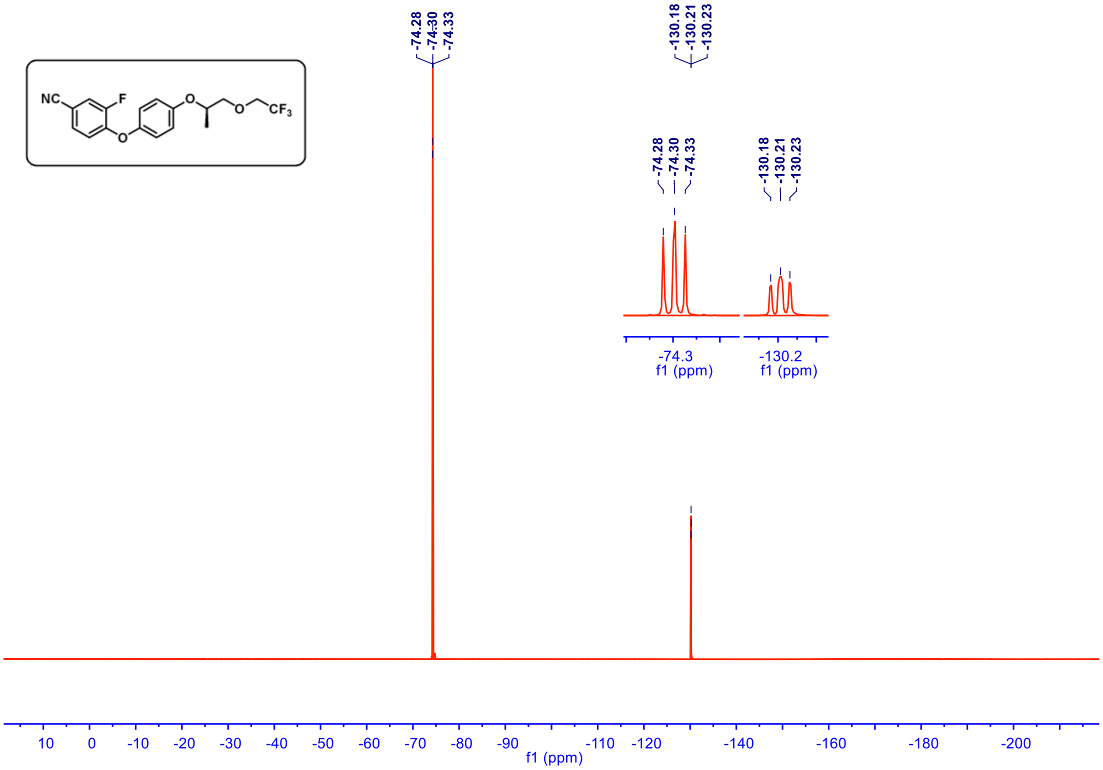

Supplementary Figure 514. ^{19}F NMR spectrum of compound **56** (376 MHz, $CDCl_3$)

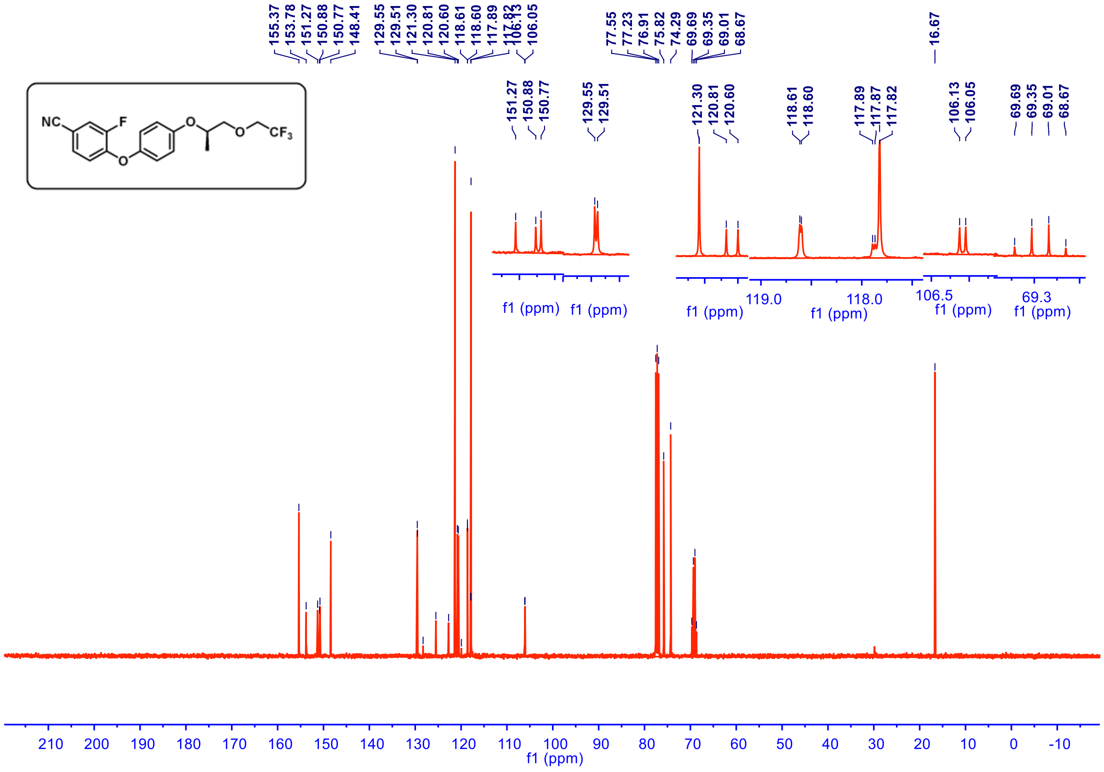

Supplementary Figure 515. ^{13}C NMR spectrum of compound **56** (101 MHz, $CDCl_3$)

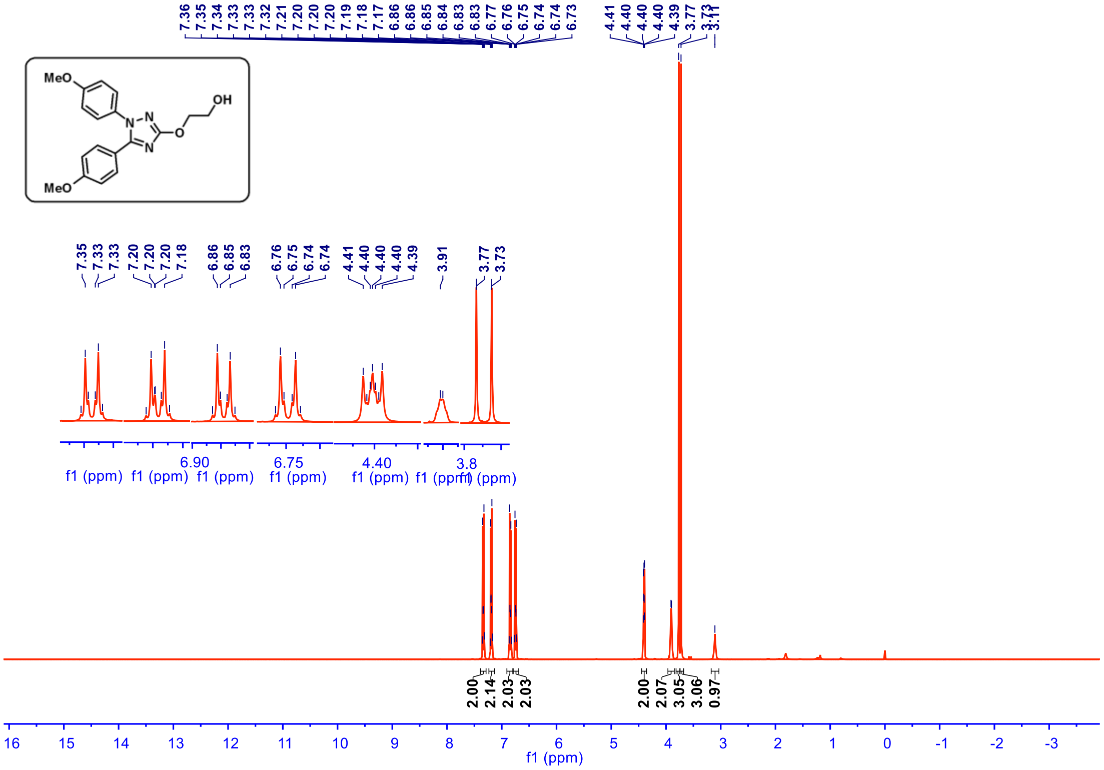

Supplementary Figure 516. ^{1}H NMR spectrum of **2-(1,5-bis(4-methoxyphenyl)-1*H*-1,2,4-triazol-3-yloxy)ethanol** (400 MHz, $CDCl_3$)

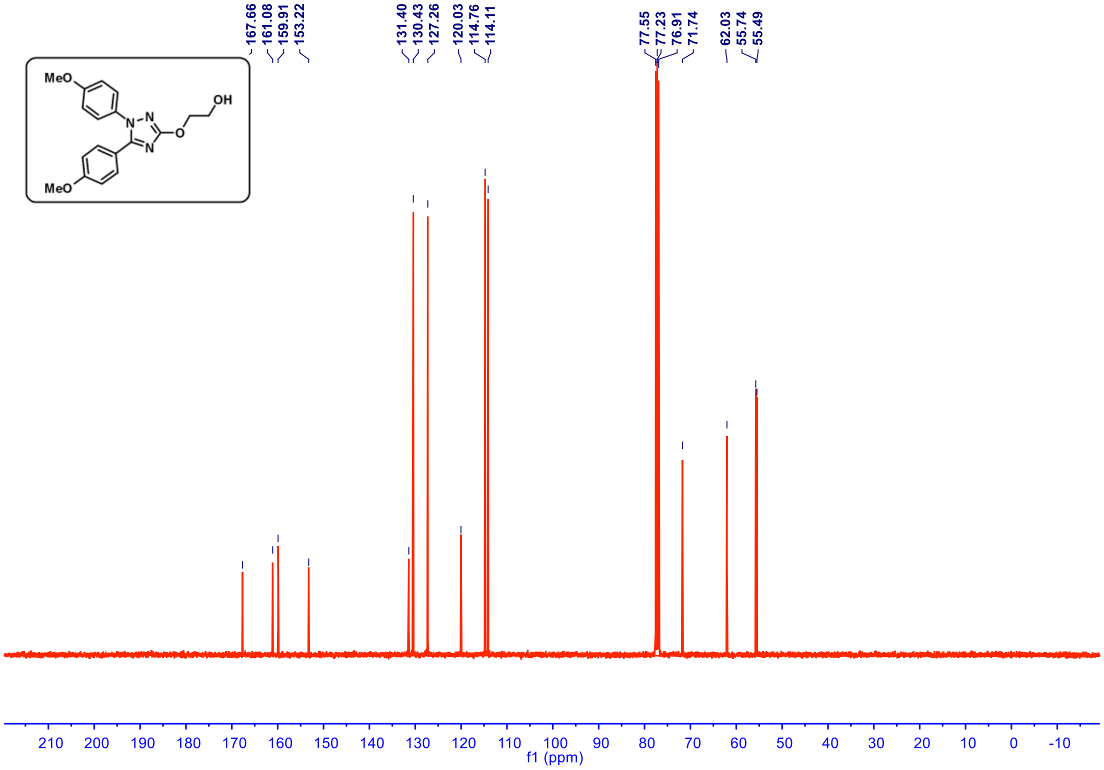

Supplementary Figure 517. ^{13}C NMR spectrum of **2-(1,5-bis(4-methoxyphenyl)-1*H*-1,2,4-triazol-3-yloxy)ethanol** (101 MHz, $CDCl_3$)

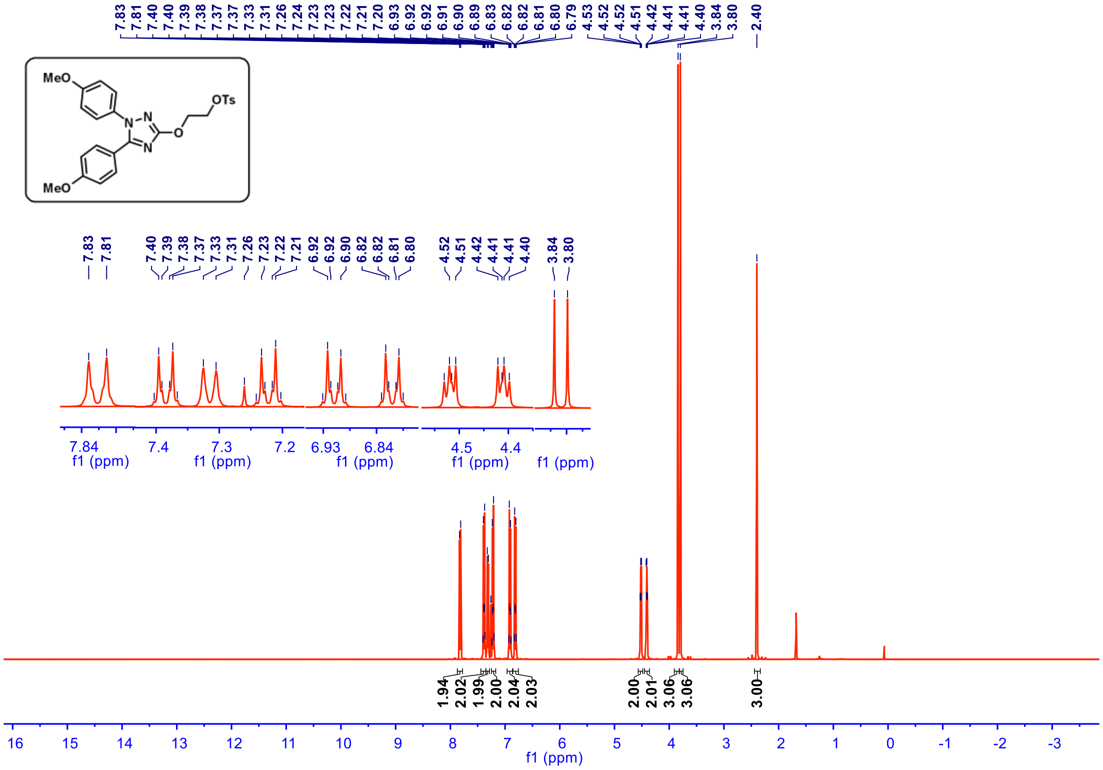

Supplementary Figure 518. ^{1}H NMR spectrum of **2-(1,5-bis(4-methoxyphenyl)-1*H*-1,2,4-triazol-3-yloxy)ethyl 4-methylbenzenesulfonate** (400 MHz, $CDCl_3$)

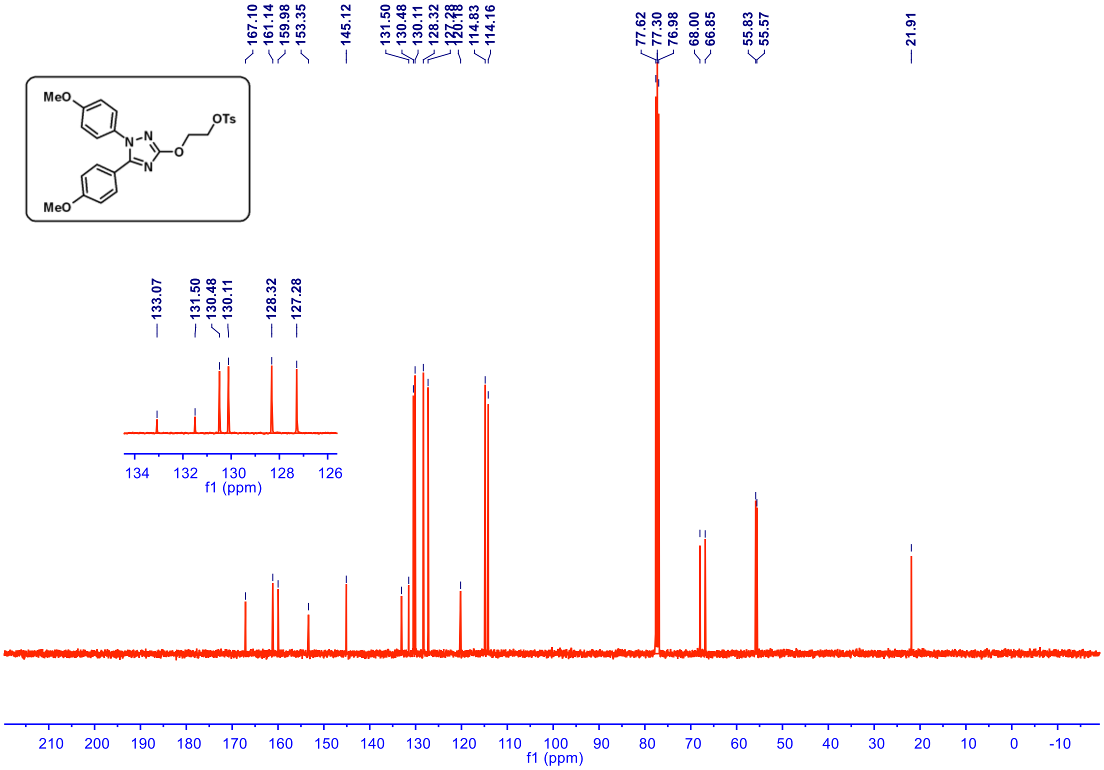

Supplementary Figure 519. ^{13}C NMR spectrum of **2-(1,5-bis(4-methoxyphenyl)-1*H*-1,2,4-triazol-3-yloxy)ethyl 4-methylbenzenesulfonate** (101 MHz, $CDCl_3$)

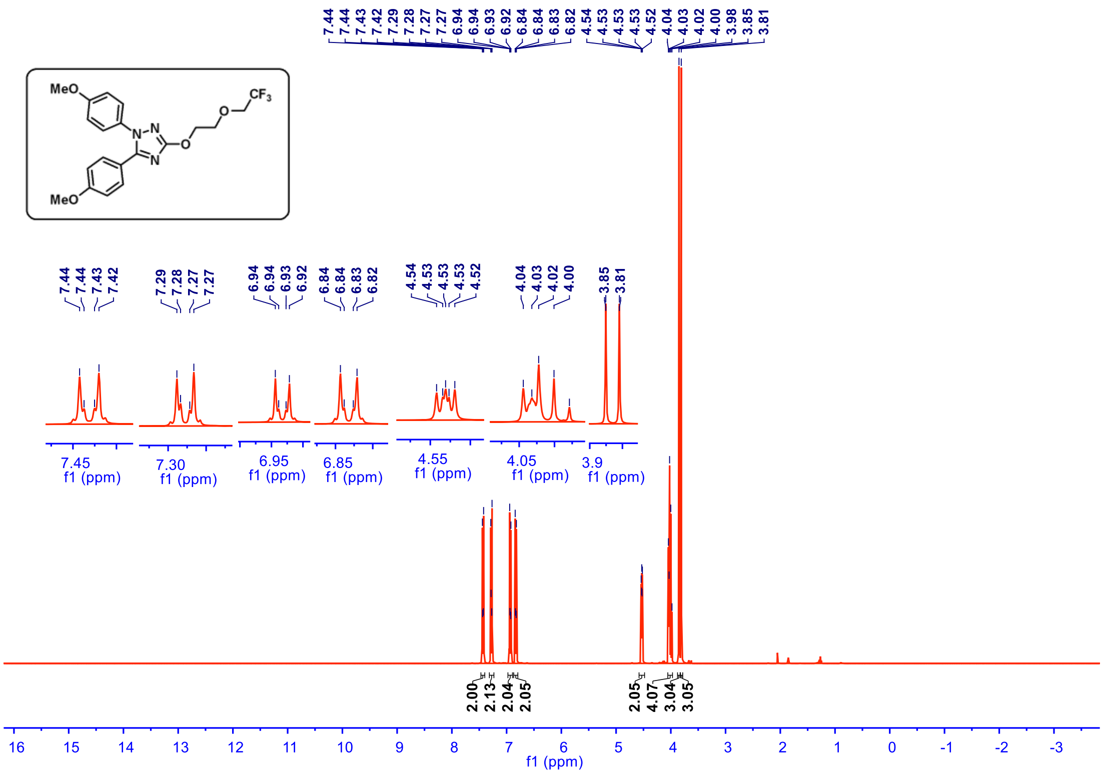

Supplementary Figure 520. ^{1}H NMR spectrum of compound **57** (400 MHz, $CDCl_3$)

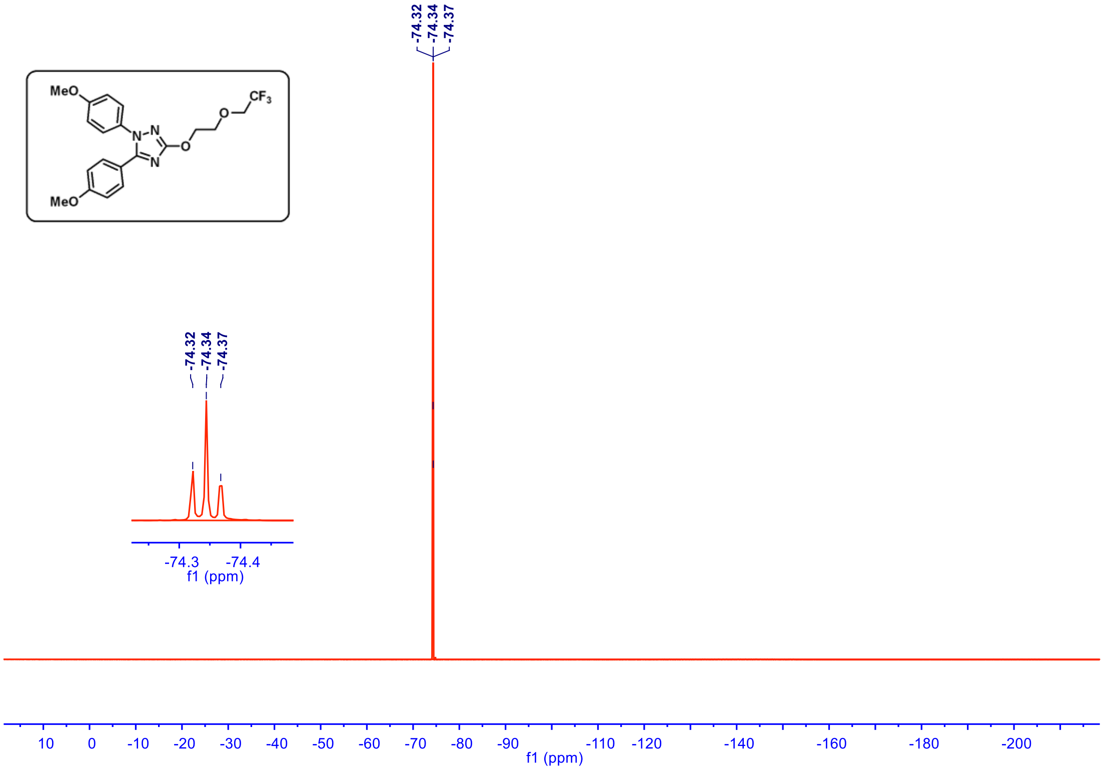

Supplementary Figure 521. ^{19}F NMR spectrum of compound **57** (376 MHz, $CDCl_3$)

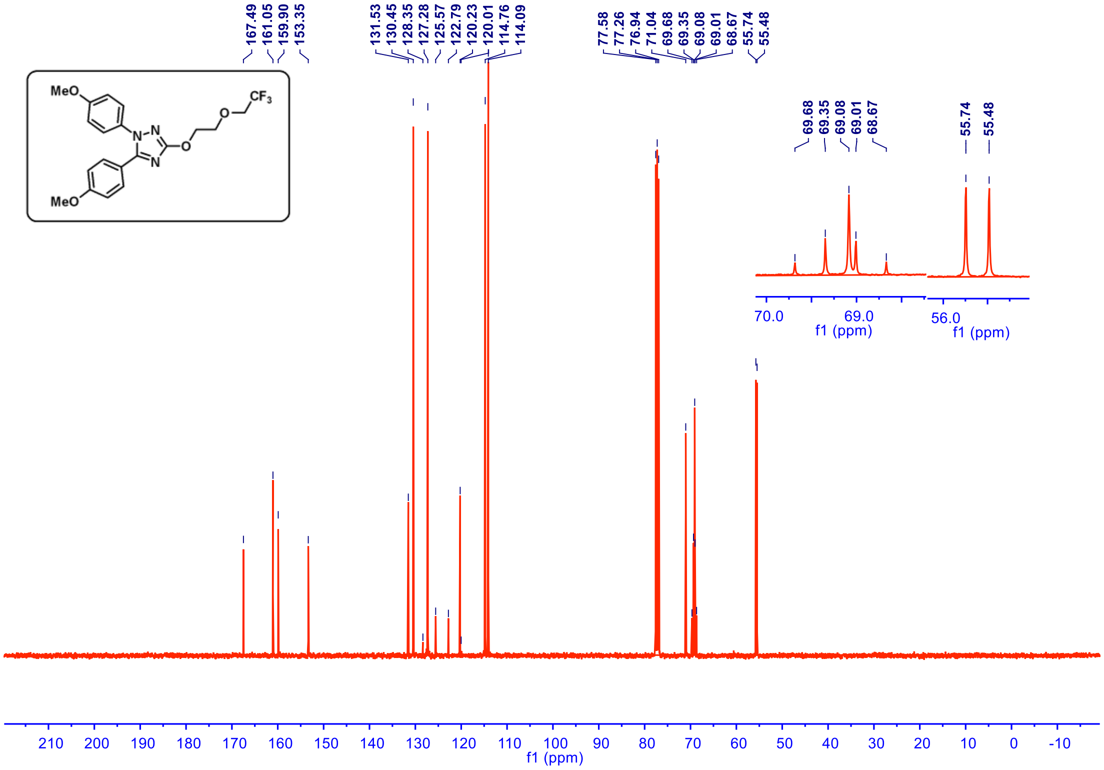

Supplementary Figure 522. ^{13}C NMR spectrum of compound **57** (101 MHz, $CDCl_3$)

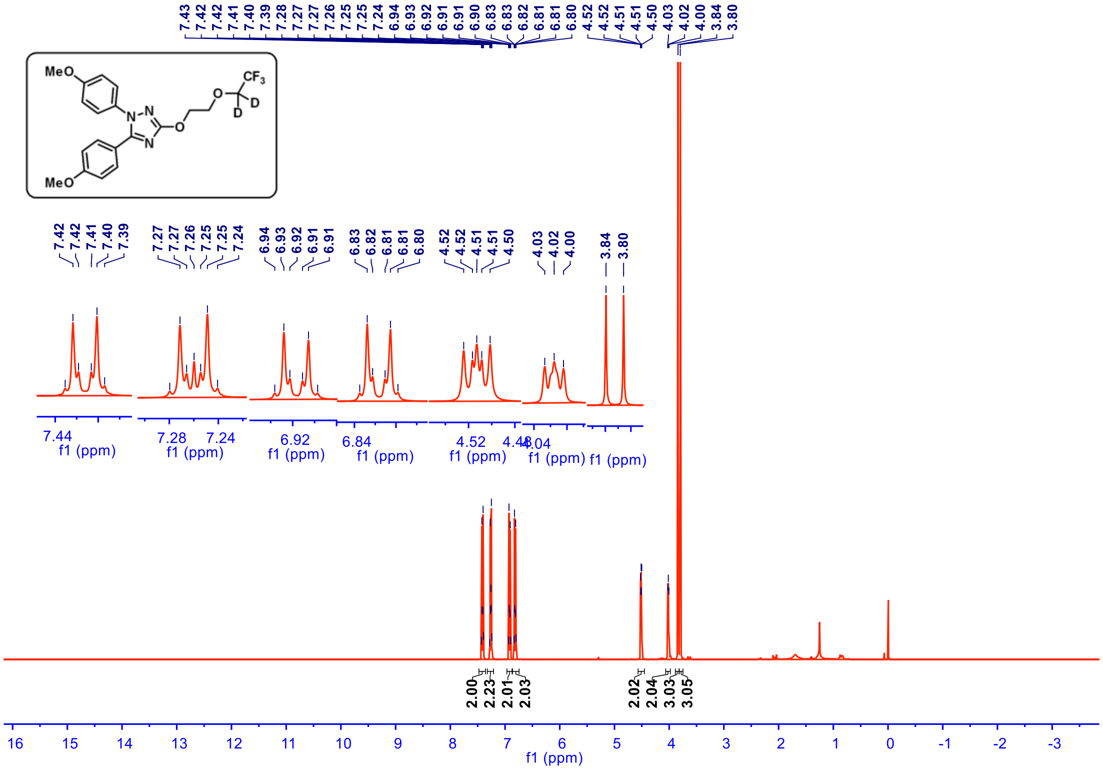

Supplementary Figure 523. ^{1}H NMR spectrum of **1,5-bis(4-methoxyphenyl)-3-(2-(2,2,2-trifluoroethoxy-1,1-*d*$_2$)ethoxy)-1*H*-1,2,4-triazole** (400 MHz, $CDCl_3$)

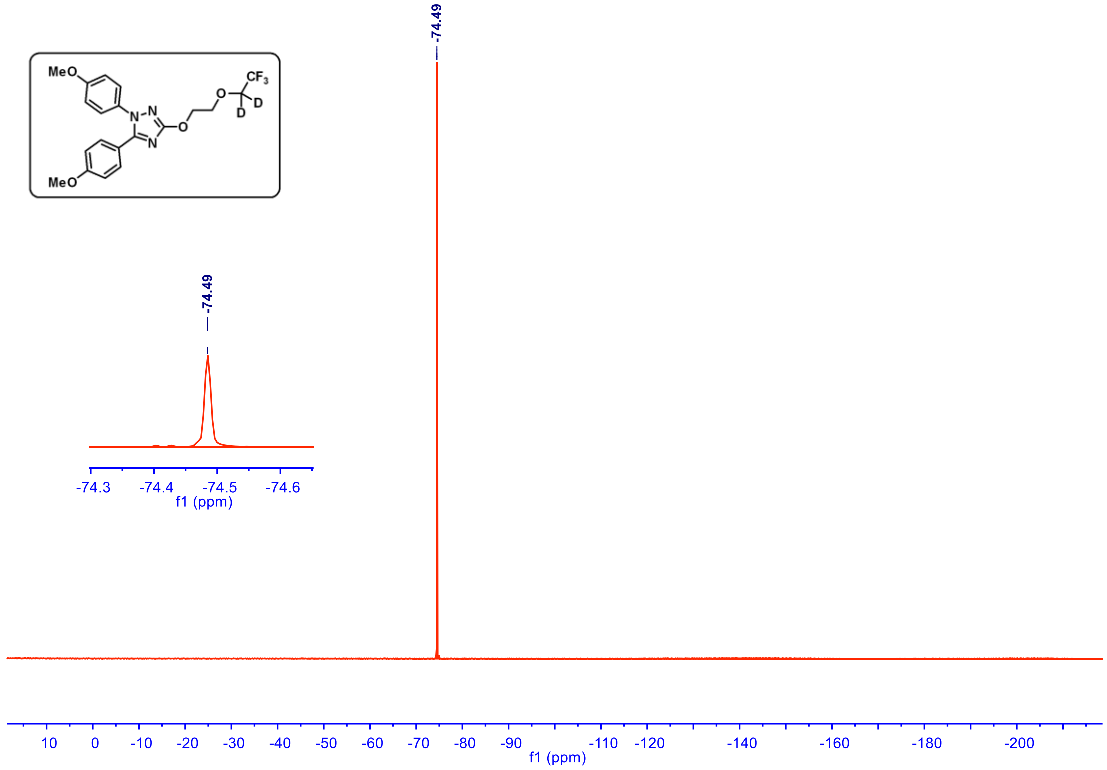

Supplementary Figure 524. ^{19}F NMR spectrum of **1,5-bis(4-methoxyphenyl)-3-(2-(2,2,2-trifluoroethoxy-1,1-*d*$_2$)ethoxy)-1*H*-1,2,4-triazole** (376 MHz, $CDCl_3$)

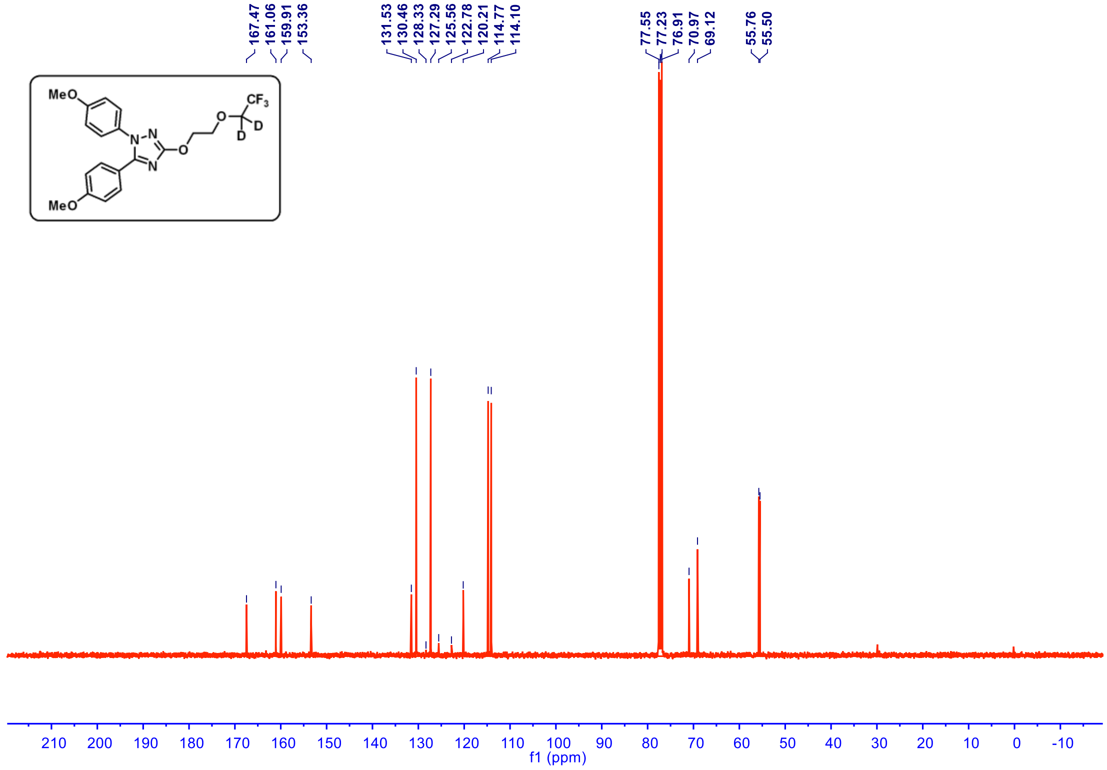

Supplementary Figure 525. ^{13}C NMR spectrum of **1,5-bis(4-methoxyphenyl)-3-(2-(2,2,2-trifluoroethoxy-1,1-*d*$_2$)ethoxy)-1*H*-1,2,4-triazole** (101 MHz, $CDCl_3$)

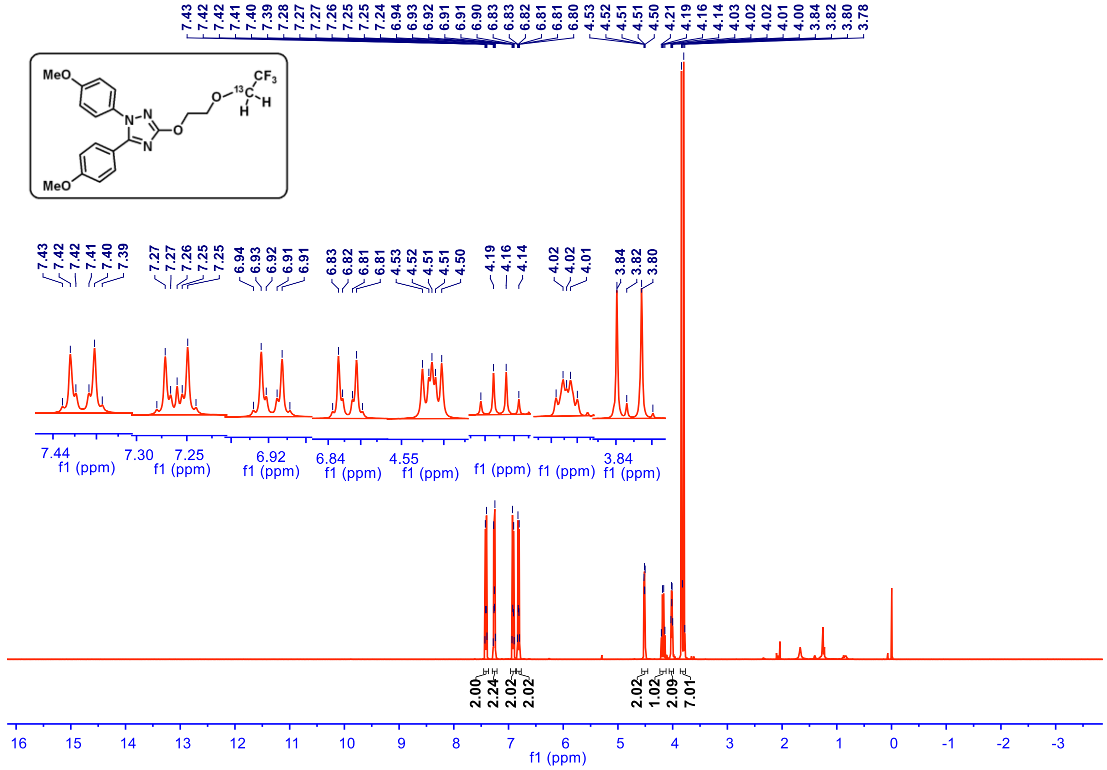

Supplementary Figure 526. ^{1}H NMR spectrum of **1,5-bis(4-methoxyphenyl)-3-(2-(2,2,2-trifluoroethoxy-1-^{13}C)ethoxy)-1*H*-1,2,4-triazole** (400 MHz, $CDCl_3$)

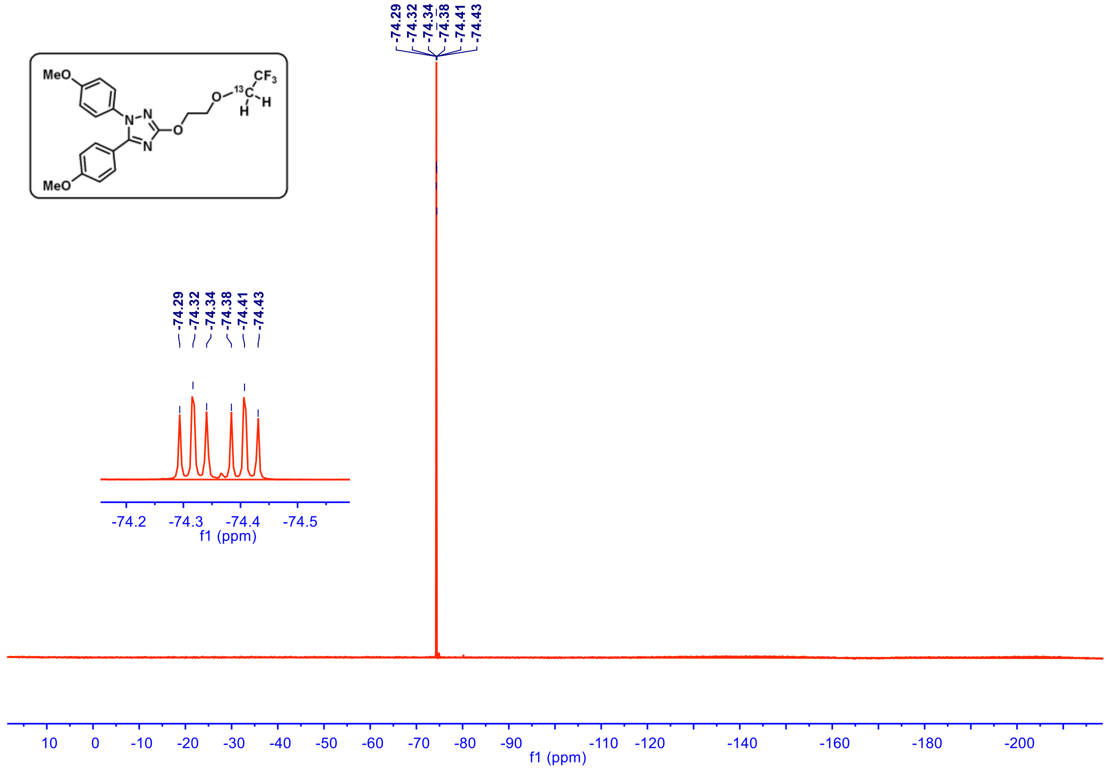

Supplementary Figure 527. ^{19}F NMR spectrum of **1,5-bis(4-methoxyphenyl)-3-(2-(2,2,2-trifluoroethoxy-1-^{13}C)ethoxy)-1*H*-1,2,4-triazole** (376 MHz, $CDCl_3$)

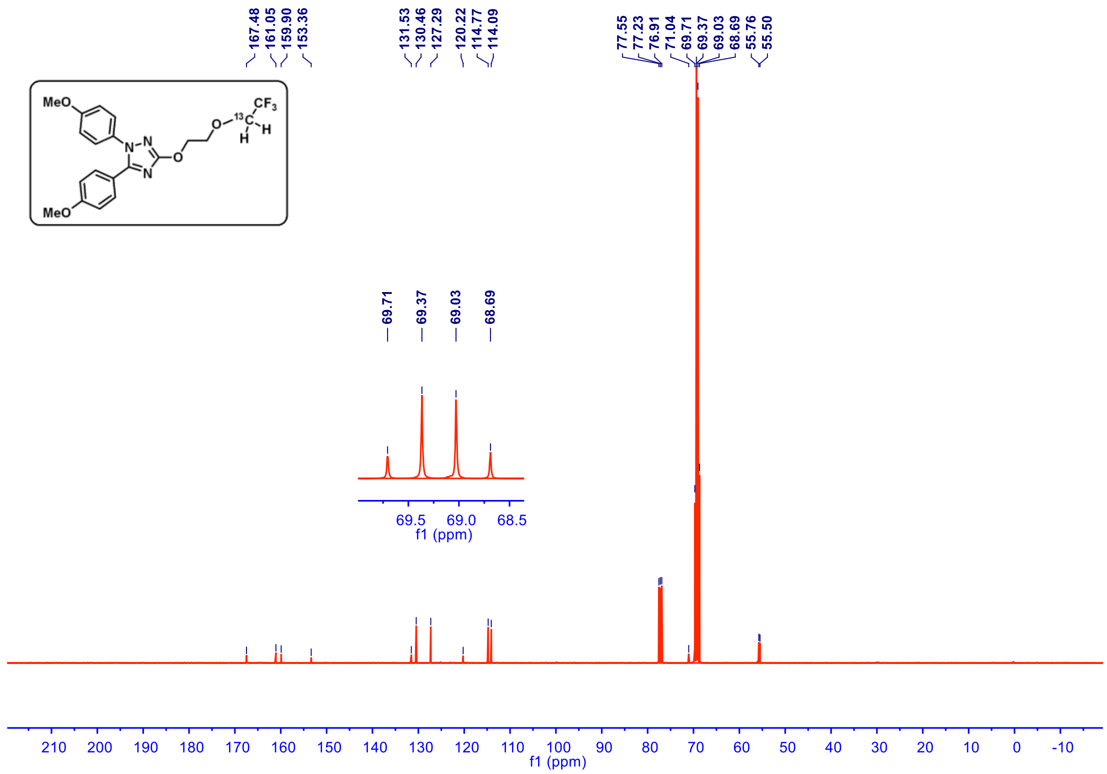

Supplementary Figure 528. ^{13}C NMR spectrum of **1,5-bis(4-methoxyphenyl)-3-(2-(2,2,2-trifluoroethoxy-1-^{13}C)ethoxy)-1*H*-1,2,4-triazole** (101 MHz, $CDCl_3$)

Supplementary References

1. Andrew, G., Brian, M., Gabriel, M. B., Kiran, R. & Marion, W. *U.S. Patent* 62677903, (2018).
2. Nilova, A., Metze, B. & Stuart, D. R. Aryl(TMP)iodonium tosylate reagents as a strategic entry point to diverse aryl intermediates: selective access to arynes. *Org. Lett.* **23**, *4813* (2021).
3. Rotstein, B. H., Wang, Lu., Liu, R. Y., Patteson, J., Kwan, E. E., Vasdev, N. & Liang, S. H. Mechanistic studies and radiofluorination of structurally diverse pharmaceuticals with spirocyclic iodonium(iii) ylides. *Chem. Sci.* **7**, *4407* (2016).
4. Chen, Z. X., Jiang, Y. W., Zhang, L., Guo, Y. L. & Ma, D. W. Oxalic diamides and *tert*-butoxide: two types of ligands enabling practical access to alkyl aryl ethers via Cu-catalyzed coupling reaction *J. Am. Chem. Soc.* **141**, *3541* (2019).
5. Yang, Q. L. & Njardarson, J. T. Base mediated deprotection strategies for trifluoroethyl (TFE) ethers, a new alcohol protecting group. *Tetrahedron Lett.* **54**, *7080* (2013).
6. Kamal, A., Pratap, T. B., Ramana, K. V., Ramana, A. V. & Babu, A. H. Facile and efficient synthesis of fluoroalkyl aryl ethers. *Tetrahedron Lett.* **43**, *7353* (2002).
7. Tan, X. Q., Liu, Z. L., Shen, H. G., Zhang, P., Zhang, Z. Z. & Li, C. Z. Trifluoromethylation of alkyl radicals in aqueous solution. *J. Am. Chem. Soc.* **139**, *12430* (2017).
8. Chiu, G., Li, S. J., Connolly, P. J., Pulito, V., Liu, J. C. & Middleton, S. A. (Phenylpiperidinyl)cyclohexylsulfonamides: development of $\alpha_{1a/1d}$-selective adrenergic receptor antagonists for the treatment of benign prostatic hyperplasia/lower urinary tract symptoms (BPH/LUTS). *Bioorg. Med. Chem. Lett.* **17**, 3930 (2007).
9. Zhang, K., Xu, X. H. & Qing, F. L. Copper-catalyzed oxidative trifluoroethoxylation of aryl boronic acids with CF_3CH_2OH. *J. Fluorine Chem.* **196**, 24 (2017).
10. Huang, R. L., Huang, Y. J., Lin, X. X., Rong, M. G. & Weng, Z. Q. Well-defined copper(I) fluoroalkoxide complexes for trifluoroethoxylation of aryl and heteroaryl bromides. *Angew. Chem. Int. Ed.* **54**, *5736* (2015).

11. Idoux, J. P., Madenwald, M. L., Garcia, B. S., Chu, D. L. & Gupton, J. T. Aromatic fluoroalkoxylation via direct displacement of a nitro or fluoro group. *J. Org. Chem.* **50**, *1876* (1985).
12. Scott D. L., Richard, N., Kim, H., Dylan, K. & Erika Mathes, L. *U.S. Patent* 16376349, (2019).
13. Haskali, M. B. & Pike, V. W. [^{11}C]Fluoroform, a breakthrough for versatile labeling of PET radiotracer trifluoromethyl groups in high molar activity. *Chem. Eur. J.* **23**, *8156* (2017).
14. van der Born, D., Sewing, C., Herscheid, J. D. M., Windhorst, A. D., Orru, R. V. & Vugts, D. J. A universal procedure for the [^{18}F]trifluoromethylation of aryl iodides and aryl boronic acids with highly improved specific activity. *Angew. Chem. Int. Ed.* **53**, *11046* (2014).
